# Excess deaths from non-COVID-19-related causes in Japan and 47 prefectures from January 2020 through May 2021 by place of death

**DOI:** 10.1016/j.ssmph.2022.101196

**Published:** 2022-08-06

**Authors:** Shuhei Nomura, Akifumi Eguchi, Cyrus Ghaznavi, Yuta Tanoue, Takayuki Kawashima, Daisuke Yoneoka, Lisa Yamasaki, Motoi Suzuki, Masahiro Hashizume

**Affiliations:** a Department of Health Policy and Management, School of Medicine, Keio University, Tokyo, Japan; b Department of Global Health Policy, Graduate School of Medicine, The University of Tokyo, Tokyo, Japan; c Tokyo Foundation for Policy Research, Tokyo, Japan; d Department of Sustainable Health Science, Center for Preventive Medical Sciences, Chiba University, Chiba, Japan; e Medical Education Program, Washington University School of Medicine in St Louis, Saint Louis, United States; f Institute for Business and Finance, Waseda University, Tokyo, Japan; g Department of Mathematical and Computing Science, Tokyo Institute of Technology, Tokyo, Japan; h Infectious Disease Surveillance Center at the National Institute of Infectious Diseases, Tokyo, Japan; i School of Medicine, Nagasaki University, Nagasaki, Japan

**Keywords:** COVID-19, Japan, Excess deaths, Non-COVID-19-related deaths, Place of death

## Abstract

Excess deaths, including all-causes mortality, were confirmed for the first time in Japan in April 2021. However, little is known about the indirect effects of COVID-19 on the number of non-COVID-19-related deaths. We then estimated the excess deaths from non-COVID-19-related causes in Japan and its 47 prefectures from January 2020 through May 2021 by place of death. Vital statistical data on deaths were obtained from the Ministry of Health, Labour and Welfare. Using quasi-Poisson regression models, we estimated the expected weekly number of deaths due to all-causes excluding COVID-19 (non-COVID-19) and due to respiratory disease, circulatory disease, malignant neoplasms, and senility. Estimates were made separately for deaths in all locations, as well as for deaths in hospitals and clinics, in nursing homes and elderly care facilities, and at home. We defined a week with excess deaths as one in which the observed number of deaths exceeded the upper bound of the two-sided 95% prediction interval. Excess death was expressed as a range of differences between the observed and expected number of deaths and the 95% upper bound of the two-sided predictive interval. The excess percentage was calculated as the number of excess deaths divided by the expected number of deaths. At the national level, excess deaths from non-COVID-19-related all-causes were observed during April 19 to May 16, 2021. The largest excess percentage was 2.73–8.58% (excess deaths 689–2161) in the week of May 3–9. Similar trends were observed for all four cause categories. The cause-of-death categories which contributed to the excesses showed heterogeneity among prefectures. When stratified by place of death, excess mortality tended to be observed in nursing homes and elderly care facilities for all categories, in hospitals and clinics for circulatory disease, and at home for respiratory disease, malignant neoplasms, and senility. A caution is necessary that for the lastest three months (March–May 2021), adjusted data were used to account for possible reporting delays.

## Highlights

• There are few studies on excess deaths due to non-COVID-19-related causes.
• Since April 2021, excess deaths from all causes other than COVID-19 have been observed in Japan.
• Excess mortality tended to be observed in nursing homes and elderly care facilities for all categories.
• These excesses have lasted for multiple, consecutive weeks, aligning with the fourth wave of COVID-19.

## 1 Introduction

In Japan, the number of deaths per population due to coronavirus disease 2019 (COVID-19) is relatively low compared to many high-income countries (World Health Organization, 2020) as of May 2021; no excess deaths (a positive difference between the observed and expected number of deaths) were reported in Japan during 2020 (Onozuka et al., 2021; Sanmarchi et al., 2021). However, since the start of the fourth wave of COVID-19 in April 2021 (with a peak in mid-May), excess deaths were reported for the first time in Japan (National Institute of Infectious Diseases, 2021). The number of excess deaths was larger than the reported number of COVID-19 deaths; therefore, the excess may include non-COVID-19-related deaths associated with COVID-19-induced healthcare system strain (NHK WORLD-JAPAN, 2021).

The objective of this study was to examine the indirect effects of COVID-19 on the number of non-COVID-19-related deaths by estimating excess weekly non-COVID-19-related deaths in Japan from the start of 2020 through May 30, 2021, by place of death.

## 2 Material and methods

### 2.1 Data

We obtained mortality data from Vital Statistics of the Ministry of Health, Labour and Welfare (MHLW). Data from 2012 (including the last few days of 2011 for weekly analysis purposes) through May 2021 were employed in this analysis, which contained information on cause of death, place of residence (prefecture), and place of death (including hospitals and clinics, nursing homes and elderly care facilities, home, and others) for all people who have residency cards and died in Japan, regardless of nationality. We excluded those who died abroad, those who were staying in Japan for a short period of time (people without residency cards), as well as those whose place of residence or date of birth were unknown.

### 2.2 Target causes of deaths

All-cause deaths except those due to COVID-19 (U7.1, in accordance with the 10th revision of the International Statistical Classification of Disease and Related Health Problems (ICD-10)) and the following four causes were considered: respiratory disease (including influenza and pneumonia [J09–J18], chronic lower respiratory diseases [J40–J47], and other diseases of the respiratory system [J00–J06, J20–J39, J60–J70, J80–J86, J90–J96, J97–J99, R09.2, and U04]), circulatory disease (hypertensive diseases [I10–I15], ischemic heart disease [I20–I25], heart failure [I50], cerebrovascular diseases [I60–I69], and other disease of the circulatory system [I00–I09, I26–I49, I51, I52, and I70–I99]), malignant neoplasms (C00–C97), and senility (R54). These cause groups and ICD-10 codes were selected based on previous analyses of comorbid conditions reported on death certificates where COVID-19 was listed as a cause of death (Centers for Disease Control and Prevention, 2020b) as well as the magnitude of the contribution to mortality burden in Japan: according to the MHLW, of deaths not due to COVID-19, these accounted for approximately 27%, 23%, 10%, and 9% of all deaths in 2019 in Japan, respectively (Ministry of Health Labour and Welfare, 2020).

### 2.3 Estimation of expected number of deaths

To estimate the expected weekly number of deaths, we utilized the Farrington algorithm (Farrington et al., 1996), which is commonly used by the epidemiology community, such as the Centers for Disease Control and Prevention of the United States as well as Japan's National Institute of Infectious Diseases, to estimate excess mortality associated with COVID-19 (Centers for Disease Control and Prevention, 2020a; Kawashima et al., 2021).

The Farrington algorithm computes a quasi-Poisson regression model, which is a generalized linear model accounting for overdispersion. It is designed to limit the data used for estimation: the expected number of deaths at a certain week t is estimated using the data during t−w and t+w weeks of years h−b and h−1, where w and b are pre-determined parameters and h is the year of t, referred to as the reference period. The regression model, thus, is given by:

[1] $$\log\left(E\left(Y_{t}\right)\right)=\alpha +\beta t+f^{T}\left(t\right)\gamma_{f\left(t\right)}\text{,}$$

where $Y_{t}$ refers to the number of deaths at a certain week t and is assumed to follow the quasi-Poisson distribution with dispersion parameter; α and β indicate regression parameters; $\gamma_{f\left(t\right)}$ is a regression parameter vector representing seasonality; and f(t) is a vector of dummy variables that equally divides the time points outside the reference period. In the present study, data for a period of one year that is not included in the reference period was divided into nine periods to control seasonality, in line with a previous study (Centers for Disease Control and Prevention, 2020a). We considered data up to five years prior (b=5) and used data of three weeks w=3 before and after a certain point as the reference period, in line with previous studies (Bedubourg & Le Strat, 2017; Centers for Disease Control and Prevention, 2020a). Since it was visually determined that the model did not fit well only for respiratory system diseases, *b* was modified to b=3 for this cause of death category only. More details regarding the Farrington algorithm can be found in elsewhere (Farrington et al., 1996; Noufaily et al., 2013). It must be noted that this study adopted exactly the same methodology as the estimation of excess all-cause mortality in Japan (Kawashima et al., 2021), where the robustness of the above parameter sets, as well as adjustments for possible reporting delay for provisional mortality data in the latest 3 months (March–May 2021) was also tested and confirmed (Kawashima et al., 2021). We specially note that we omitted one specific condition in the original Farrington algorithm such that the linear trend term βt is dropped when the predicted value is not larger than any observed value. The details of the methods are documented in the Appendix.

### 2.4 Definitions of excess and exiguous deaths

We defined a week with excess and exiguous deaths as one in which the observed number of deaths exceeded the upper bound or fell below the lower bound of the two-sided 95% prediction interval, respectively. Excess/exiguous deaths were expressed as a range that included the difference between the observed number of deaths and the expected number of deaths in addition to that between the observed number of deaths and the upper/lower bounds of the two-sided 95% prediction interval. The excess/exiguous percentage was also calculated as the number of excess/exiguous deaths divided by the expected number of deaths.

## 3 Results

**Fig. 1:**
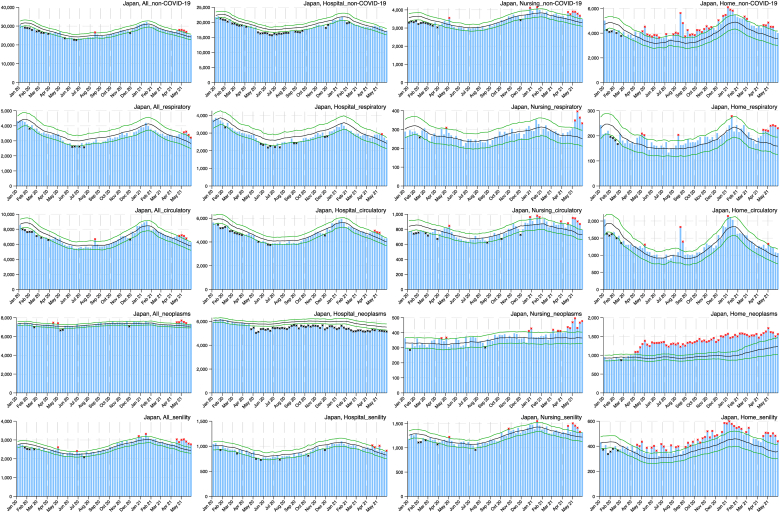
Weekly observed and 95% upper/lower bounds of the expected weekly number of deaths in Japan from January 2020 through June 2021 by cause categories and by place of death.

Between December 30, 2019, and May 30, 2021, 1990379 people in Japan died of non-COVID-19-related causes; no excess deaths from non-COVID-19-related all-causes were observed during consecutive weeks in 2020, but in 2021, excess deaths from non-COVID-19-related all-causes were observed between April 19 and May 16 (Fig. 1). The largest excess percentage was 2.73–8.58% (689–2161) during the week of May 3–9 (Table A.1 in the Appendix). A similar trend was observed for the four cause categories of death: excess deaths due to respiratory disease occurred between May 3 and 30, 2021, with the largest excess percentage of 6.17–19.38% (185–581) being observed during the week of May 10–16. Excess deaths due to circulatory disease occurred between April 19 and May 16, 2021, with the largest excess percentage of 5.00–14.39% (308–886) being observed during the week of May 3–9. Excess deaths due to malignant neoplasms occurred between April 12 and May 16, 2021, with the largest excess percentage of 3.71–6.59% (265–471) being observed during the week of April 26 to May 2. Excess deaths due to senility occurred between April 12 and May 30, 2021, with the largest excess percentage of 13.08–19.36% (329–487) being observed during the week of May 3–9.

Table 1 shows the cumulative number of excess deaths since April 2021, when excess deaths were observed during consecutive weeks for the first time, and for the same period in 2020, by cause categories and place of death. As can be visually inferred from Fig. 1, excess deaths tended to be observed in nursing homes and elderly care facilities for all four categories, in hospitals and clinics for circulatory disease, and at home for respiratory disease, malignant neoplasms, and senility. Since approximately April 2020, exiguous deaths due to malignant neoplasms have been observed in hospitals and clinics while excess deaths have been observed at home. The presence or absence of excess deaths varied among the prefectures, but there was a general tendency for them to occur after April 2021, and the place of death also exhibited similar trends among all prefectures. Figure A in the Appendix presents the weekly observed and 95% upper and lower bounds of the expected weekly number of deaths in the 47 prefectures from January 2020 through May 2021 for non-COVID-19-related all-causes and the four cause categories, by place of death. Their exact values can be found in the Appendix.

**Table 1:** Cumulative number of observed and excess/exiguous deaths in Japan from non-COVID-19-related causes and the four cause categories by place of death from April through May 2020 and 2021.

Non-COVID-19-related causes	Weeks	Observed	Excess deaths	Exiguous deaths
All locations				
	Apr 6 to May 31, 2020	201439	0–1225	72–5569
	Apr 5 to May 30, 2021	214444	1995–11387	0–0
Hospitals and clinics				
	Apr 6 to May 31, 2020	140522	0–0	2750–8976
	Apr 5 to May 30, 2021	144953	0–3179	0–481
Nursing homes and elderly care facilities				
	Apr 6 to May 31, 2020	25688	134–567	0–303
	Apr 5 to May 30, 2021	29737	1026–2529	0–0
Home				
	Apr 6 to May 31, 2020	31558	1228–4389	0–0
	Apr 5 to May 30, 2021	35543	981–5490	0–0
Respiratory disease	Weeks	Observed	Excess deaths	Exiguous deaths
All locations				
	Apr 6 to May 31, 2020	25490	0–71	0–1265
	Apr 5 to May 30, 2021	27095	348–2552	0–78
Hospitals and clinics				
	Apr 6 to May 31, 2020	21641	0–0	65–1551
	Apr 5 to May 30, 2021	22511	5–1518	0–103
Nursing homes and elderly care facilities				
	Apr 6 to May 31, 2020	2221	3–147	0–3
	Apr 5 to May 30, 2021	2580	218–479	0–4
Home				
	Apr 6 to May 31, 2020	1467	18–193	0–13
	Apr 5 to May 30, 2021	1802	185–460	0–0
Circulatory disease	Weeks	Observed	Excess deaths	Exiguous deaths
All locations				
	Apr 6 to May 31, 2020	50779	0–513	0–1139
	Apr 5 to May 30, 2021	54694	709–4875	0–0
Hospitals and clinics				
	Apr 6 to May 31, 2020	34824	0–0	17–1595
	Apr 5 to May 30, 2021	37124	244–2616	0–0
Nursing homes and elderly care facilities				
	Apr 6 to May 31, 2020	6087	32–249	0–60
	Apr 5 to May 30, 2021	6976	367–869	0–0
Home				
	Apr 6 to May 31, 2020	9180	52–823	0–0
	Apr 5 to May 30, 2021	9659	22–1143	0–0
Malignant neoplasms	Weeks	Observed	Excess deaths	Exiguous deaths
All locations				
	Apr 6 to May 31, 2020	56818	84–703	239–649
	Apr 5 to May 30, 2021	59132	571–1960	0–0
Hospitals and clinics				
	Apr 6 to May 31, 2020	43532	0–0	1627–2893
	Apr 5 to May 30, 2021	42219	0–0	524–2476
Nursing homes and elderly care facilities				
	Apr 6 to May 31, 2020	2741	8–169	0–23
	Apr 5 to May 30, 2021	3582	350–659	0–0
Home				
	Apr 6 to May 31, 2020	10096	2073–2704	0–0
	Apr 5 to May 30, 2021	12654	1343–3085	0–0
Senility	Weeks	Observed	Excess deaths	Exiguous deaths
All locations				
	Apr 6 to May 31, 2020	19031	81–451	0–152
	Apr 5 to May 30, 2021	23001	1388–2601	0–0
Hospitals and clinics				
	Apr 6 to May 31, 2020	6573	0–78	15–280
	Apr 5 to May 30, 2021	7539	74–518	0–0
Nursing homes and elderly care facilities				
	Apr 6 to May 31, 2020	8979	10–169	0–143
	Apr 5 to May 30, 2021	11028	408–1030	0–0
Home				
	Apr 6 to May 31, 2020	3047	187–487	0–0
	Apr 5 to May 30, 2021	3802	371–830	0–0

Note: The cumulative number of excess/exiguous deaths was calculated by summing up the weekly excess deaths during the period.

## 4 Discussion

In 2020, exiguous deaths in hospitals and clinics and excess deaths at home were observed for malignant neoplasm-related deaths starting in April, which coincided with the first declaration of a state of emergency against COVID-19 in Japan. At that time, it was necessary to concentrate limited medical resources on patients with severe COVID-19 and potentially infected patients, and patients receiving long-term inpatient care were encouraged to be transferred to nursing homes, elderly care facilities, or home care (Makiyama et al., 2021). There are also reports that the number of terminal patients choosing to end their lives at home has increased due to the risk of infection in hospitals and restrictions on family visits to prevent nosocomial infections (Tsukamoto, 2020). Although non-COVID-19 patients were required to postpone non-urgent surgeries, change treatment methods, and suspend outpatient visits (Mori et al., 2020), there were no significant excesses in malignant neoplasms-related deaths (and non-COVID-19-related all-deaths) regardless of place of death by the end of 2020, suggesting that there was little health impact from restrictions on access to non-emergent care during this period.

In 2021, excess deaths from non-COVID-19-related all-causes were observed during the fourth wave of COVID-19 that began in April. Excess deaths were observed simultaneously for all major causes of deaths in Japan. As opposed to the long-term health effects of restrictions on access to non-urgent medical care and changes in lifestyle, it is possible that the strain on healthcare systems caused by the rapid increase in COVID-19 patients has affected acute and end-of-life care (Honda, 2021). In particular, the excess deaths secondary to all four cause categories observed in nursing homes and elderly care facilities and those due to all categories except for circulatory disease at home would indicate that it was extremely difficult for medical institutions to accept critically ill patients.

The limitations of this study lie in its reliance on provisional data in the latest 3 months, March–May 2021, (although attempts were made to adjust for reporting delays) and in the assumptions applied to the model. In addition, the Farrington algorithm for estimating excess/exiguous deaths only evaluates whether there are more or fewer deaths than in previous years; therefore, we did not directly assess causality between the COVID-19 pandemic and the number of deaths in any given category. In other words, the fact that excess deaths were observed does not allow us to conclude that they were the result of indirect effects of the pandemic. It is possible that our estimates also include the effects of unobserved contingencies (e.g., temperature) unrelated to the pandemic.

It is important to note that due to the nature of the methodology used in this study, data from 2020 was used to estimate 2021. In some locations and at some times, exiguous deaths were observed during 2020, which may have exerted an effect on the expected number of deaths (and the associated 95% prediction interval) around the same time in 2021. However, our estimates in 2021 depend not only on data from 2020, but also on data from before 2020 (2016–2019), and in this sense 2020 alone is unlikely to have a significant impact on current estimates of excess/exiguous deaths. The alternative, namely excluding 2020 data when calculating 2021 estimates, would jeopardize the estimation of longitudinal time trends and seasonality, which we believe would constitute an inappropriate mathematical approach to estimating excess deaths during the pandemic.

## 5 Conclusions

Using vital statistics data on deaths from January 2012 to May 2021 obtained from MHLW, we estimated excess deaths from all causes other than COVID-19 (i.e. non-COVID-19-related all-cause deaths), as well as deaths from respiratory disease, circulatory disease, malignant neoplasms, and senility, on a weekly basis during 2020–2021 compared to previous years. We found that since April 2021, excess deaths lasting multiple, consecutive weeks have been observed for the first time in Japan for all cause categories, coinciding with the onset of the fourth wave of COVID-19. In addition, when stratified by place of death, excess deaths tended to occur in nursing homes and elderly care facilities for all categories, in hospitals and clinics for circulatory disease, and at home for respiratory disease, malignant neoplasms, and senility. These findings may indicate that COVID-19-associated healthcare system strain has had profound consequences for acute and end-of-life care. A caution is necessary that for the lastest three months (March–May 2021), adjusted data were used to account for possible reporting delays.

## Ethics statement

Ethical approval was granted by the ethics committee of the National Institute of Infectious Diseases, under authorization number 1174.

## Funding

The present work was supported in part by grants from the 10.13039/501100003478Ministry of Health, Labour and Welfare of Japan and the 10.13039/501100001700Ministry of Education, Culture, Sports, Science and Technology of Japan (21H03203), and by 10.13039/501100009023Precursory Research for Embryonic Science and Technology from the 10.13039/501100002241Japan Science and Technology Agency (JPMJPR21RC). The funders had no role in the study design, data collection and analysis, decision to publish, or preparation of the manuscript.

## Consent to participate

Not applicable.

## Availability of data and material

The mortality data have been obtained through a restricted data-use agreement with the Ministry of Health, Labour and Welfare, Japan, and are therefore not available for public dissemination.

## Code availability

Not applicable.

## CRediT authorship contribution statement

**Shuhei Nomura:** Conceptualization, Data curation, Formal analysis, Funding acquisition, Investigation, Methodology, Project administration, Resources, Validation, Writing – original draft, Writing – review & editing. **Akifumi Eguchi:** Conceptualization, Data curation, Formal analysis, Investigation, Methodology, Validation, Writing – original draft, Writing – review & editing. **Cyrus Ghaznavi:** Investigation, Writing – original draft, Writing – review & editing. **Yuta Tanoue:** Data curation, Formal analysis, Investigation, Methodology, Validation, Writing – review & editing. **Takayuki Kawashima:** Data curation, Formal analysis, Investigation, Methodology, Validation, Writing – review & editing. **Daisuke Yoneoka:** Data curation, Formal analysis, Funding acquisition, Investigation, Methodology, Validation, Writing – review & editing. **Lisa Yamasaki:** Investigation, Writing – review & editing. **Motoi Suzuki:** Data curation, Investigation, Project administration, Resources, Writing – review & editing. **Masahiro Hashizume:** Conceptualization, Data curation, Funding acquisition, Investigation, Project administration, Resources, Supervision, Writing – review & editing.

## Declaration of competing interest

The authors declare that they have no competing interests.
