# Supplementary material for: Excess deaths from non-COVID-19-related causes in Japan and 47 prefectures from January 2020 through May 2021 by place of death
Source: SSM Popul Health. 2022 Aug 6;19:101196. doi: 10.1016/j.ssmph.2022.101196 (PMC9356762; doi:10.1016/j.ssmph.2022.101196)
Supplement: Multimedia component 1 [file mmc1.pdf]

Appendix

Figure A: Weekly observed and 95% upper/lower bounds of the expected weekly number of deaths in 47 prefectures from January 2020 through June 2021 by cause categories and by place of death.

Blue bar indicates the observed number of deaths, black line indicates the expected number of deaths, green lines indicate the 95% upper and lower bounds of the expected number of deaths, red asterisk indicates the observed count above the upper bound, and black asterisk indicates the observed count below the upper bound.

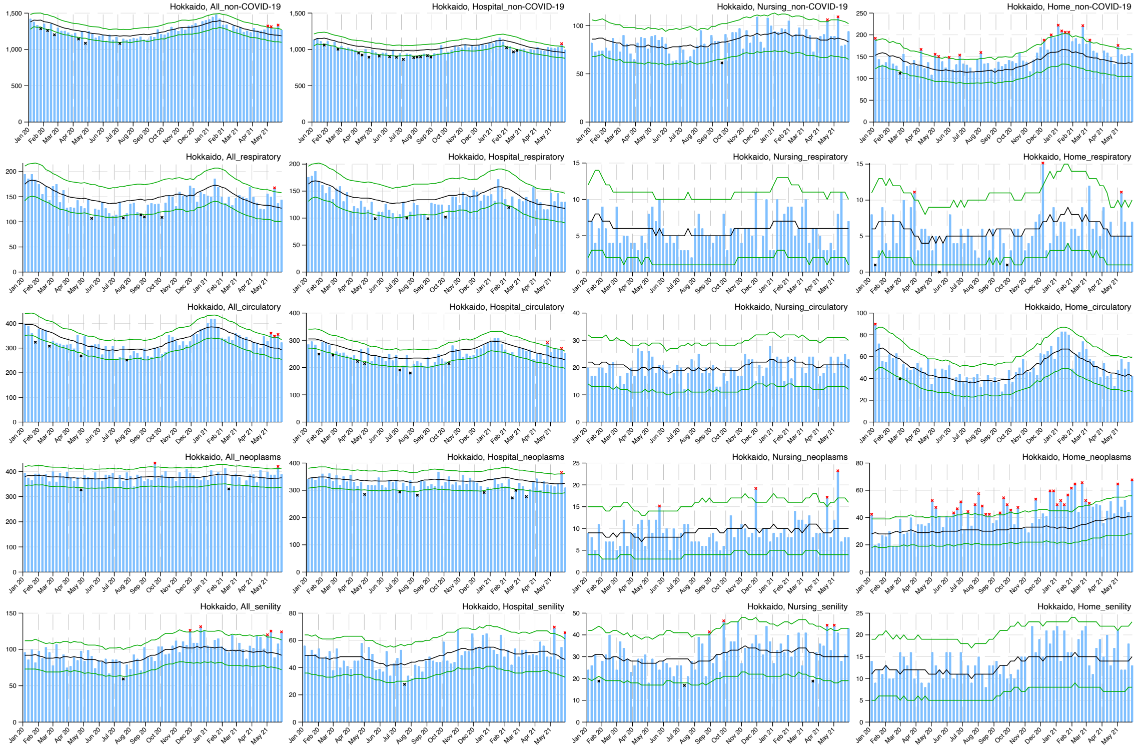
Hokkaido, All_non-COVID-19
Hokkaido, Hospital_non-COVID-19
Hokkaido, Nursing_non-COVID-19
Hokkaido, Home_non-COVID-19
Hokkaido, All_respiratory
Hokkaido, Hospital_respiratory
Hokkaido, Nursing_respiratory
Hokkaido, Home_respiratory
Hokkaido, All_circulatory
Hokkaido, Hospital_circulatory
Hokkaido, Nursing_circulatory
Hokkaido, Home_circulatory
Hokkaido, All_neoplasms
Hokkaido, Hospital_neoplasms
Hokkaido, Nursing_neoplasms
Hokkaido, Home_neoplasms
Hokkaido, All_senility
Hokkaido, Hospital_senility
Hokkaido, Nursing_senility
Hokkaido, Home_senility
Jan 20
Feb 20
Mar 20
Apr 20
May 20
Jun 20
Jul 20
Aug 20
Sep 20
Oct 20
Nov 20
Dec 20
Jan 21
Feb 21
Mar 21
Apr 21
May 21

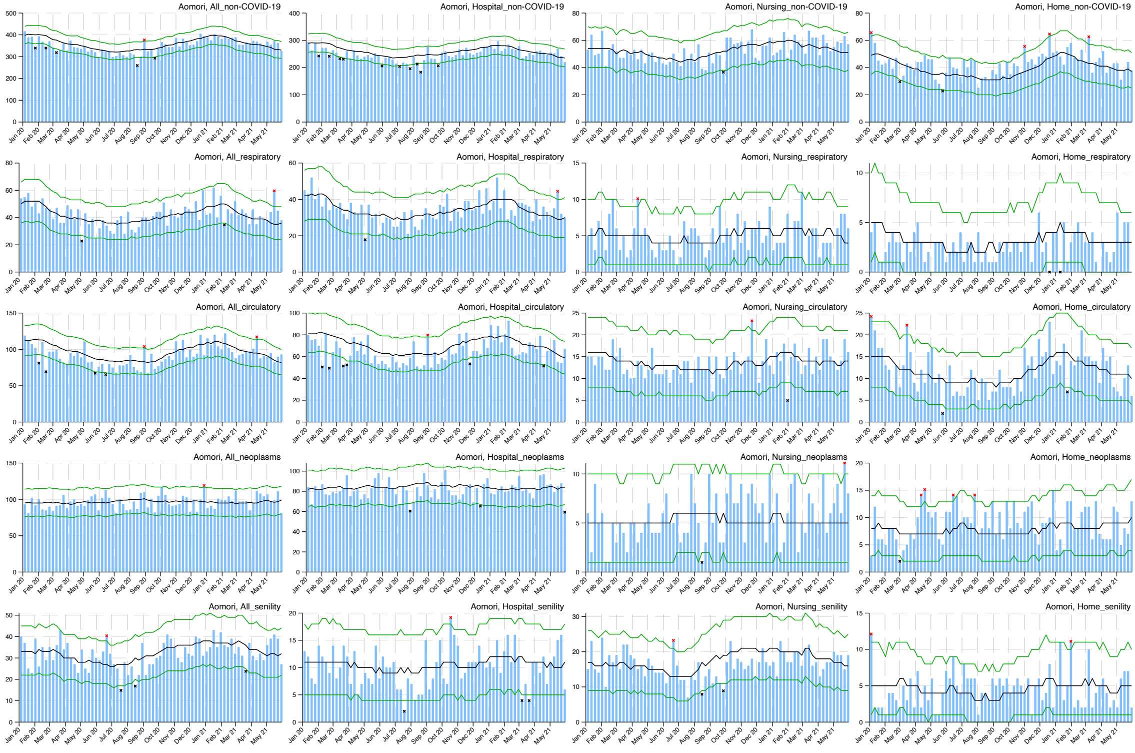
Aomori, All_non-COVID-19
Aomori, Hospital_non-COVID-19
Aomori, Nursing_non-COVID-19
Aomori, Home_non-COVID-19
Aomori, All_respiratory
Aomori, Hospital_respiratory
Aomori, Nursing_respiratory
Aomori, Home_respiratory
Aomori, All_circulatory
Aomori, Hospital_circulatory
Aomori, Nursing_circulatory
Aomori, Home_circulatory
Aomori, All_neoplasms
Aomori, Hospital_neoplasms
Aomori, Nursing_neoplasms
Aomori, Home_neoplasms
Aomori, All_senility
Aomori, Hospital_senility
Aomori, Nursing_senility
Aomori, Home_senility
Jan 20
Feb 20
Mar 20
Apr 20
May 20
Jun 20
Jul 20
Aug 20
Sep 20
Oct 20
Nov 20
Dec 20
Jan 21
Feb 21
Mar 21
Apr 21
May 21

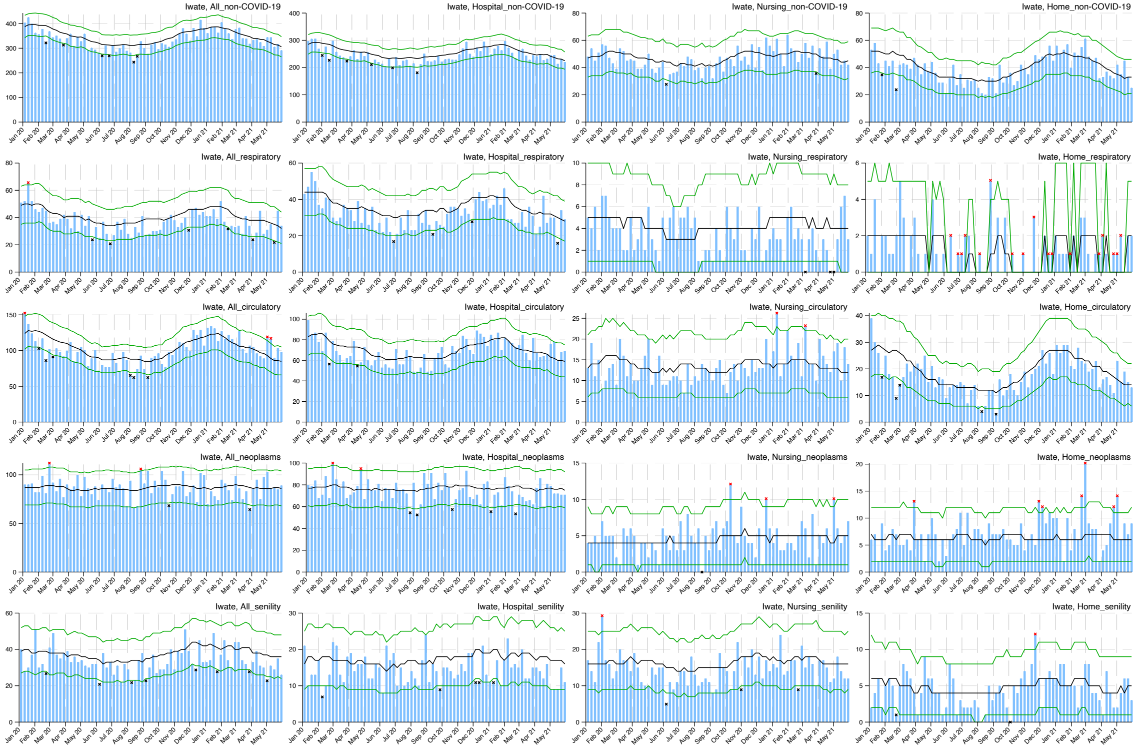

Iwate, All_non-COVID-19
Iwate, Hospital_non-COVID-19
Iwate, Nursing_non-COVID-19
Iwate, Home_non-COVID-19
Iwate, All_respiratory
Iwate, Hospital_respiratory
Iwate, Nursing_respiratory
Iwate, Home_respiratory
Iwate, All_circulatory
Iwate, Hospital_circulatory
Iwate, Nursing_circulatory
Iwate, Home_circulatory
Iwate, All_neoplasms
Iwate, Hospital_neoplasms
Iwate, Nursing_neoplasms
Iwate, Home_neoplasms
Iwate, All_senility
Iwate, Hospital_senility
Iwate, Nursing_senility
Iwate, Home_senility
Jan 20
Feb 20
Mar 20
Apr 20
May 20
Jun 20
Jul 20
Aug 20
Sep 20
Oct 20
Nov 20
Dec 20
Jan 21
Feb 21
Mar 21
Apr 21
May 21

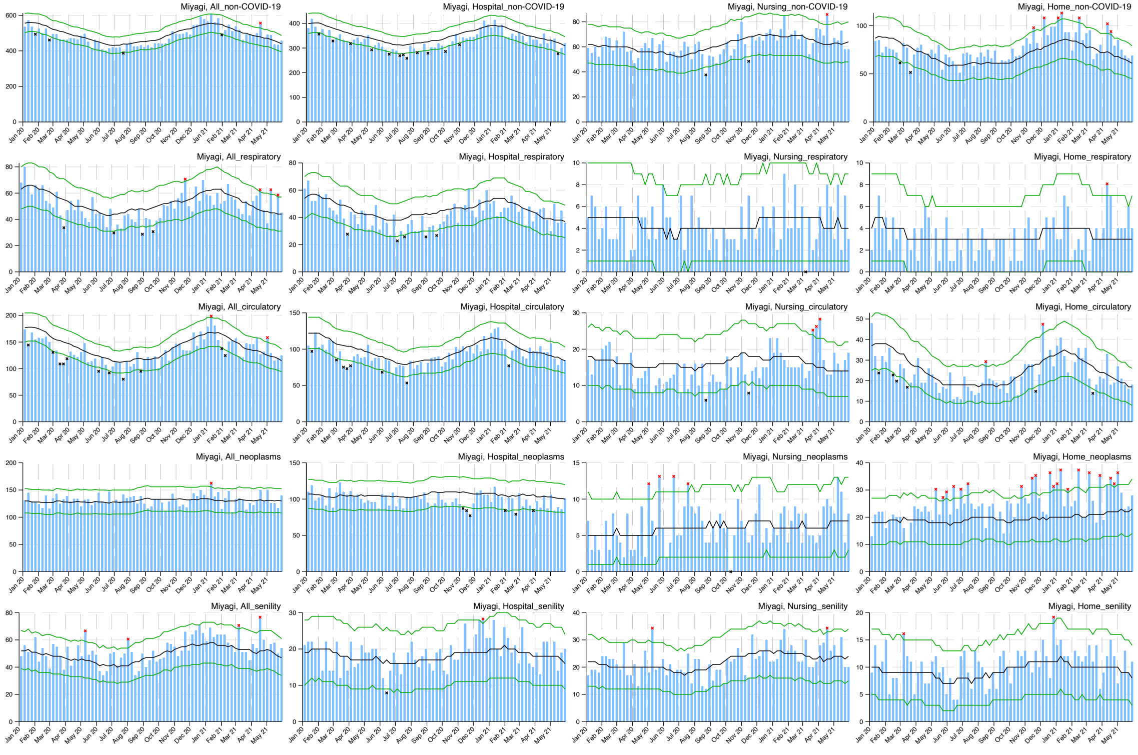
Miyagi, All_non-COVID-19
Miyagi, Hospital_non-COVID-19
Miyagi, Nursing_non-COVID-19
Miyagi, Home_non-COVID-19
Miyagi, All_respiratory
Miyagi, Hospital_respiratory
Miyagi, Nursing_respiratory
Miyagi, Home_respiratory
Miyagi, All_circulatory
Miyagi, Hospital_circulatory
Miyagi, Nursing_circulatory
Miyagi, Home_circulatory
Miyagi, All_neoplasms
Miyagi, Hospital_neoplasms
Miyagi, Nursing_neoplasms
Miyagi, Home_neoplasms
Miyagi, All_senility
Miyagi, Hospital_senility
Miyagi, Nursing_senility
Miyagi, Home_senility

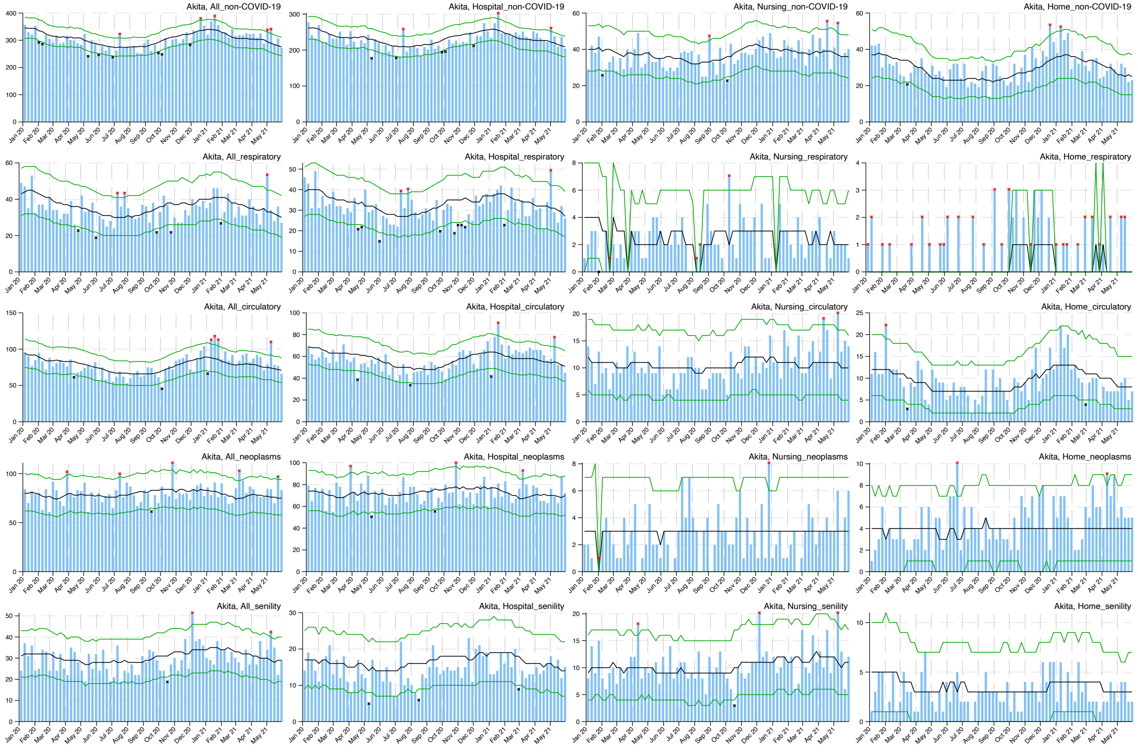
Akita, All_non-COVID-19
Akita, Hospital_non-COVID-19
Akita, Nursing_non-COVID-19
Akita, Home_non-COVID-19
Akita, All_respiratory
Akita, Hospital_respiratory
Akita, Nursing_respiratory
Akita, Home_respiratory
Akita, All_circulatory
Akita, Hospital_circulatory
Akita, Nursing_circulatory
Akita, Home_circulatory
Akita, All_neoplasms
Akita, Hospital_neoplasms
Akita, Nursing_neoplasms
Akita, Home_neoplasms
Akita, All_senility
Akita, Hospital_senility
Akita, Nursing_senility
Akita, Home_senility

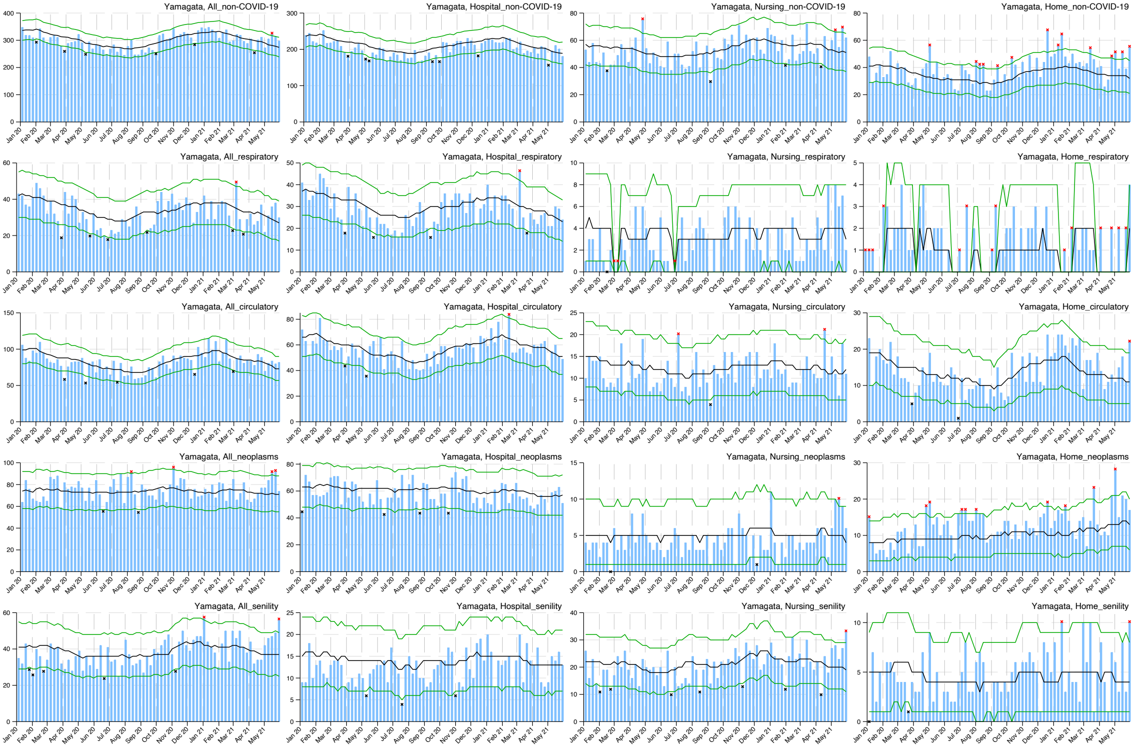
Yamagata, All_non-COVID-19
Yamagata, Hospital_non-COVID-19
Yamagata, Nursing_non-COVID-19
Yamagata, Home_non-COVID-19
Yamagata, All_respiratory
Yamagata, Hospital_respiratory
Yamagata, Nursing_respiratory
Yamagata, Home_respiratory
Yamagata, All_circulatory
Yamagata, Hospital_circulatory
Yamagata, Nursing_circulatory
Yamagata, Home_circulatory
Yamagata, All_neoplasms
Yamagata, Hospital_neoplasms
Yamagata, Nursing_neoplasms
Yamagata, Home_neoplasms
Yamagata, All_senility
Yamagata, Hospital_senility
Yamagata, Nursing_senility
Yamagata, Home_senility

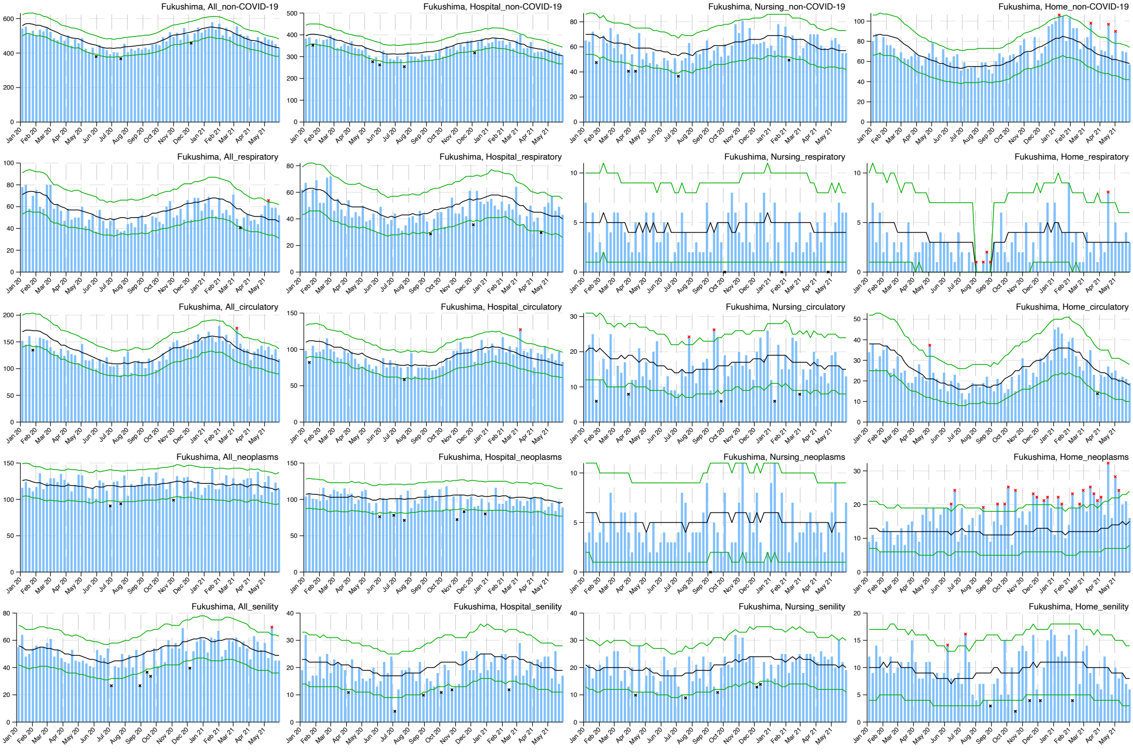
Fukushima, All_non-COVID-19
Fukushima, Hospital_non-COVID-19
Fukushima, Nursing_non-COVID-19
Fukushima, Home_non-COVID-19
Fukushima, All_respiratory
Fukushima, Hospital_respiratory
Fukushima, Nursing_respiratory
Fukushima, Home_respiratory
Fukushima, All_circulatory
Fukushima, Hospital_circulatory
Fukushima, Nursing_circulatory
Fukushima, Home_circulatory
Fukushima, All_neoplasms
Fukushima, Hospital_neoplasms
Fukushima, Nursing_neoplasms
Fukushima, Home_neoplasms
Fukushima, All_senility
Fukushima, Hospital_senility
Fukushima, Nursing_senility
Fukushima, Home_senility
Jan 20
Feb 20
Mar 20
Apr 20
May 20
Jun 20
Jul 20
Aug 20
Sep 20
Oct 20
Nov 20
Dec 20
Jan 21
Feb 21
Mar 21
Apr 21
May 21

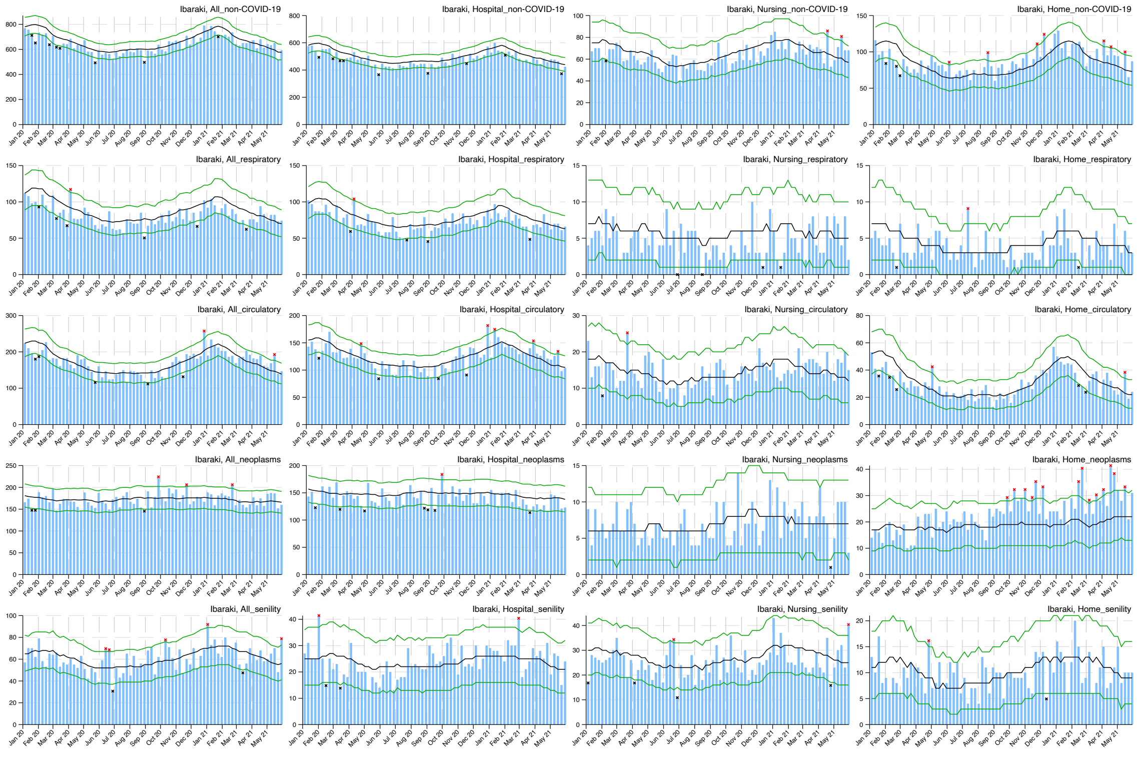

Ibaraki, All_non-COVID-19
0 200 400 600 800
Ibaraki, Hospital_non-COVID-19
0 200 400 600 800
Ibaraki, Nursing_non-COVID-19
0 20 40 60 80 100
Ibaraki, Home_non-COVID-19
0 50 100 150
Ibaraki, All_respiratory
0 50 100 150
Ibaraki, Hospital_respiratory
0 50 100 150
Ibaraki, Nursing_respiratory
0 5 10 15
Ibaraki, Home_respiratory
0 5 10 15
Ibaraki, All_circulatory
0 100 200 300
Ibaraki, Hospital_circulatory
0 50 100 150 200
Ibaraki, Nursing_circulatory
0 10 20 30
Ibaraki, Home_circulatory
0 20 40 60 80
Ibaraki, All_neoplasms
0 50 100 150 200 250
Ibaraki, Hospital_neoplasms
0 50 100 150 200
Ibaraki, Nursing_neoplasms
0 5 10 15
Ibaraki, Home_neoplasms
0 10 20 30 40
Ibaraki, All_senility
0 20 40 60 80 100
Ibaraki, Hospital_senility
0 10 20 30 40
Ibaraki, Nursing_senility
0 10 20 30 40
Ibaraki, Home_senility
0 5 10 15 20
Jan 20 Feb 20 Mar 20 Apr 20 May 20 Jun 20 Jul 20 Aug 20 Sep 20 Oct 20 Nov 20 Dec 20 Jan 21 Feb 21 Mar 21 Apr 21 May 21

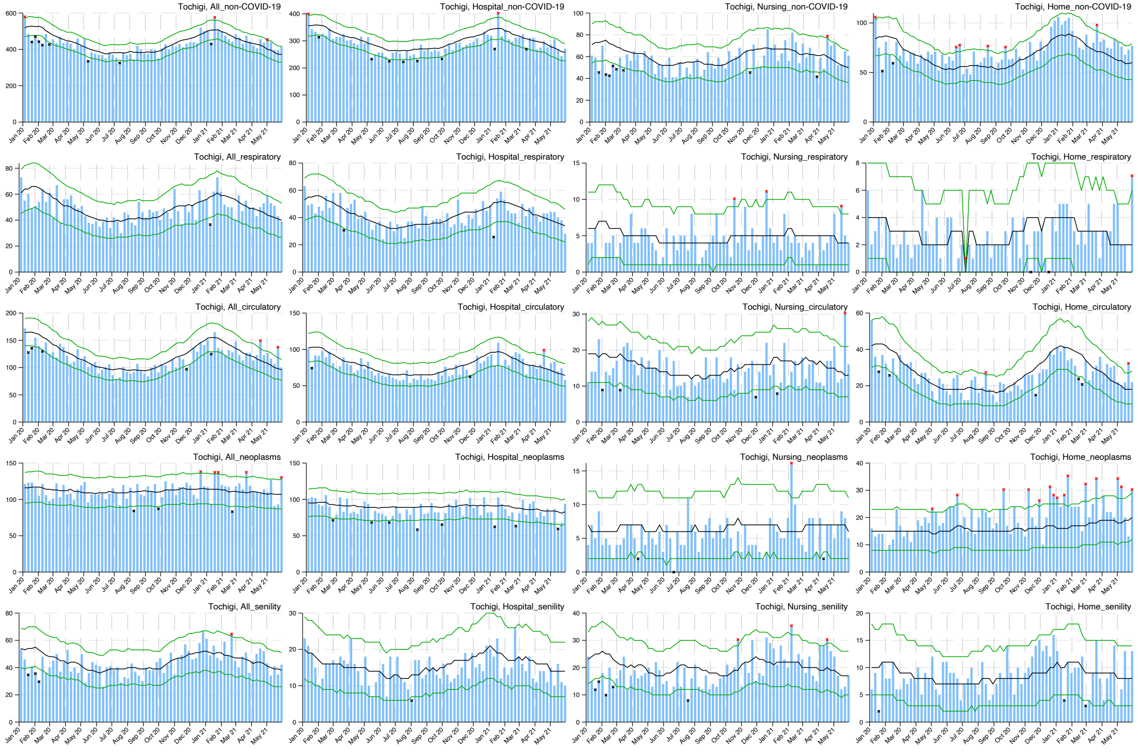
Tochigi, All_non-COVID-19
Tochigi, Hospital_non-COVID-19
Tochigi, Nursing_non-COVID-19
Tochigi, Home_non-COVID-19
Tochigi, All_respiratory
Tochigi, Hospital_respiratory
Tochigi, Nursing_respiratory
Tochigi, Home_respiratory
Tochigi, All_circulatory
Tochigi, Hospital_circulatory
Tochigi, Nursing_circulatory
Tochigi, Home_circulatory
Tochigi, All_neoplasms
Tochigi, Hospital_neoplasms
Tochigi, Nursing_neoplasms
Tochigi, Home_neoplasms
Tochigi, All_senility
Tochigi, Hospital_senility
Tochigi, Nursing_senility
Tochigi, Home_senility

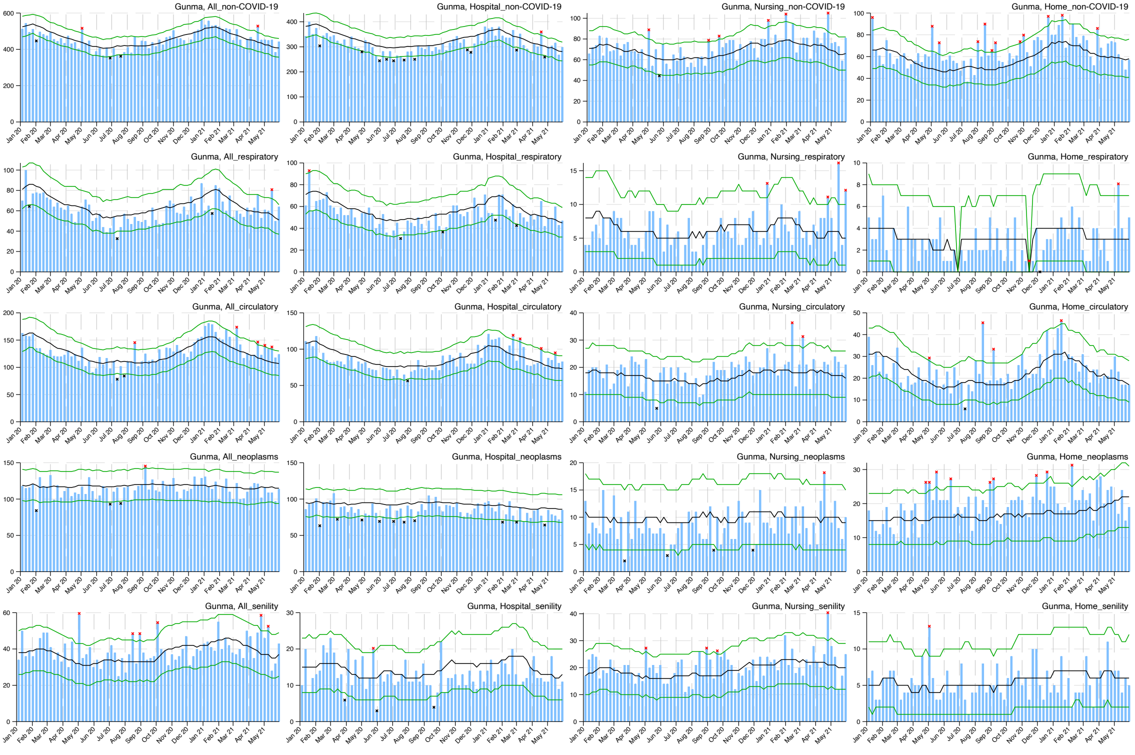

Gunma, All_non-COVID-19
0 200 400 600
Gunma, Hospital_non-COVID-19
0 100 200 300 400
Gunma, Nursing_non-COVID-19
0 20 40 60 80 100
Gunma, Home_non-COVID-19
0 20 40 60 80 100
Gunma, All_respiratory
0 20 40 60 80 100
Gunma, Hospital_respiratory
0 20 40 60 80 100
Gunma, Nursing_respiratory
0 5 10 15
Gunma, Home_respiratory
0 2 4 6 8 10
Gunma, All_circulatory
0 50 100 150 200
Gunma, Hospital_circulatory
0 50 100 150
Gunma, Nursing_circulatory
0 10 20 30 40
Gunma, Home_circulatory
0 10 20 30 40 50
Gunma, All_neoplasms
0 50 100 150
Gunma, Hospital_neoplasms
0 50 100 150
Gunma, Nursing_neoplasms
0 5 10 15 20
Gunma, Home_neoplasms
0 10 20 30
Gunma, All_senility
0 20 40 60
Gunma, Hospital_senility
0 10 20 30
Gunma, Nursing_senility
0 10 20 30 40
Gunma, Home_senility
0 5 10 15
Jan 20 Feb 20 Mar 20 Apr 20 May 20 Jun 20 Jul 20 Aug 20 Sep 20 Oct 20 Nov 20 Dec 20 Jan 21 Feb 21 Mar 21 Apr 21 May 21

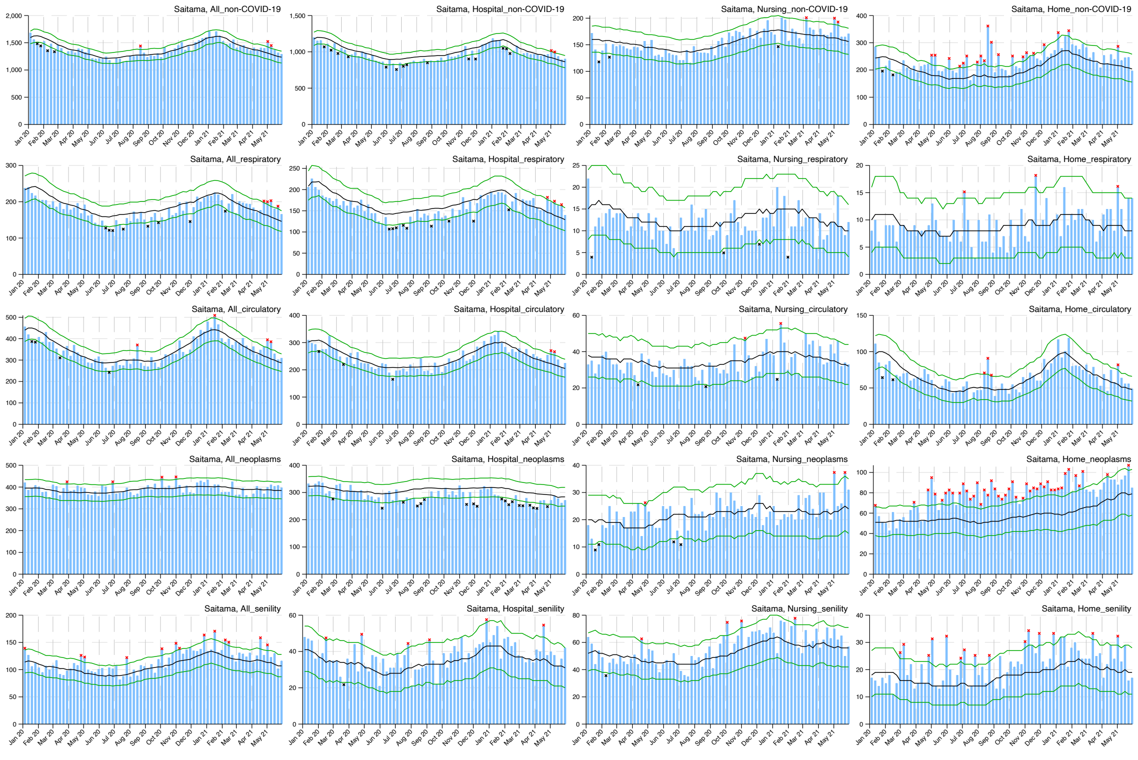
Saitama, All_non-COVID-19
Saitama, Hospital_non-COVID-19
Saitama, Nursing_non-COVID-19
Saitama, Home_non-COVID-19
Saitama, All_respiratory
Saitama, Hospital_respiratory
Saitama, Nursing_respiratory
Saitama, Home_respiratory
Saitama, All_circulatory
Saitama, Hospital_circulatory
Saitama, Nursing_circulatory
Saitama, Home_circulatory
Saitama, All_neoplasms
Saitama, Hospital_neoplasms
Saitama, Nursing_neoplasms
Saitama, Home_neoplasms
Saitama, All_senility
Saitama, Hospital_senility
Saitama, Nursing_senility
Saitama, Home_senility
Jan 20 Feb 20 Mar 20 Apr 20 May 20 Jun 20 Jul 20 Aug 20 Sep 20 Oct 20 Nov 20 Dec 20 Jan 21 Feb 21 Mar 21 Apr 21 May 21

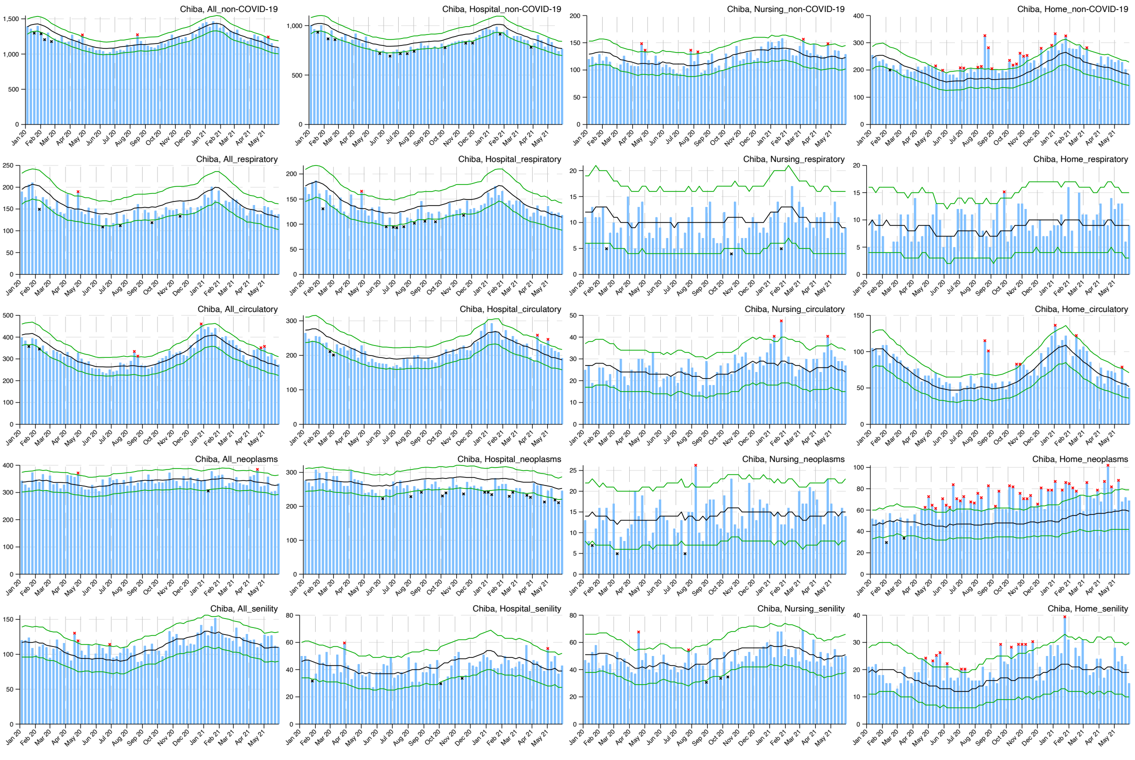

Chiba, All_non-COVID-19
Chiba, Hospital_non-COVID-19
Chiba, Nursing_non-COVID-19
Chiba, Home_non-COVID-19
Chiba, All_respiratory
Chiba, Hospital_respiratory
Chiba, Nursing_respiratory
Chiba, Home_respiratory
Chiba, All_circulatory
Chiba, Hospital_circulatory
Chiba, Nursing_circulatory
Chiba, Home_circulatory
Chiba, All_neoplasms
Chiba, Hospital_neoplasms
Chiba, Nursing_neoplasms
Chiba, Home_neoplasms
Chiba, All_senility
Chiba, Hospital_senility
Chiba, Nursing_senility
Chiba, Home_senility
Jan 20 Feb 20 Mar 20 Apr 20 May 20 Jun 20 Jul 20 Aug 20 Sep 20 Oct 20 Nov 20 Dec 20 Jan 21 Feb 21 Mar 21 Apr 21 May 21

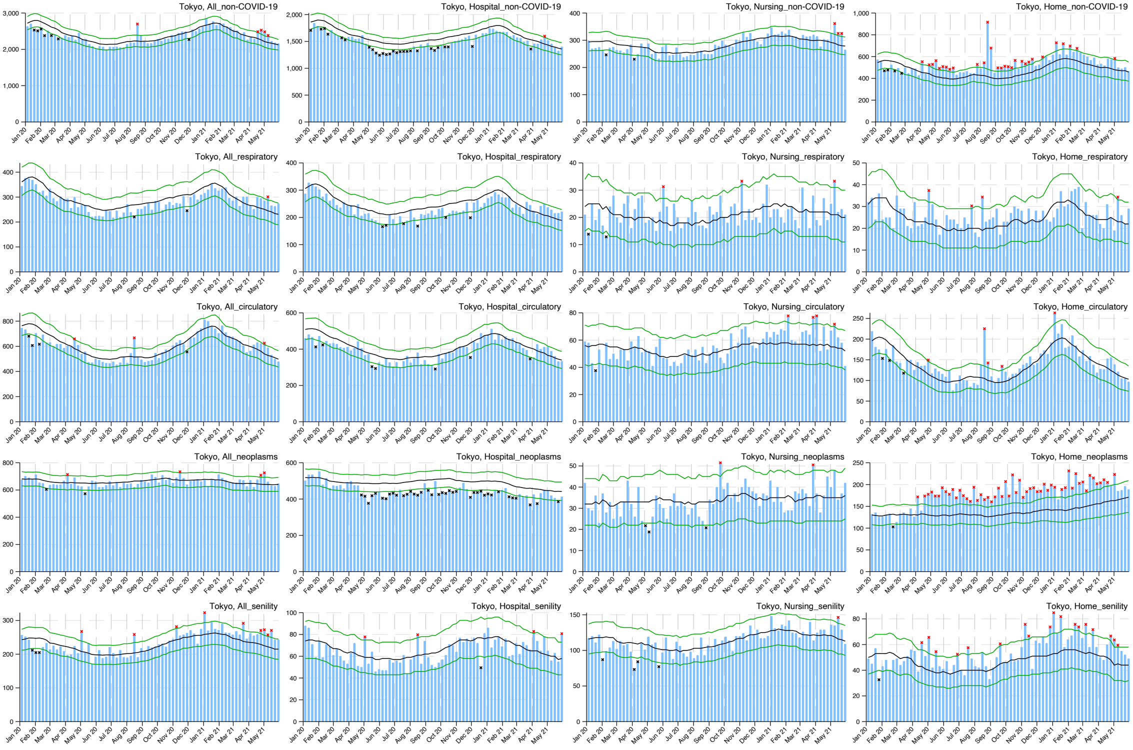
Tokyo, All_non-COVID-19
Tokyo, Hospital_non-COVID-19
Tokyo, Nursing_non-COVID-19
Tokyo, Home_non-COVID-19
Tokyo, All_respiratory
Tokyo, Hospital_respiratory
Tokyo, Nursing_respiratory
Tokyo, Home_respiratory
Tokyo, All_circulatory
Tokyo, Hospital_circulatory
Tokyo, Nursing_circulatory
Tokyo, Home_circulatory
Tokyo, All_neoplasms
Tokyo, Hospital_neoplasms
Tokyo, Nursing_neoplasms
Tokyo, Home_neoplasms
Tokyo, All_senility
Tokyo, Hospital_senility
Tokyo, Nursing_senility
Tokyo, Home_senility

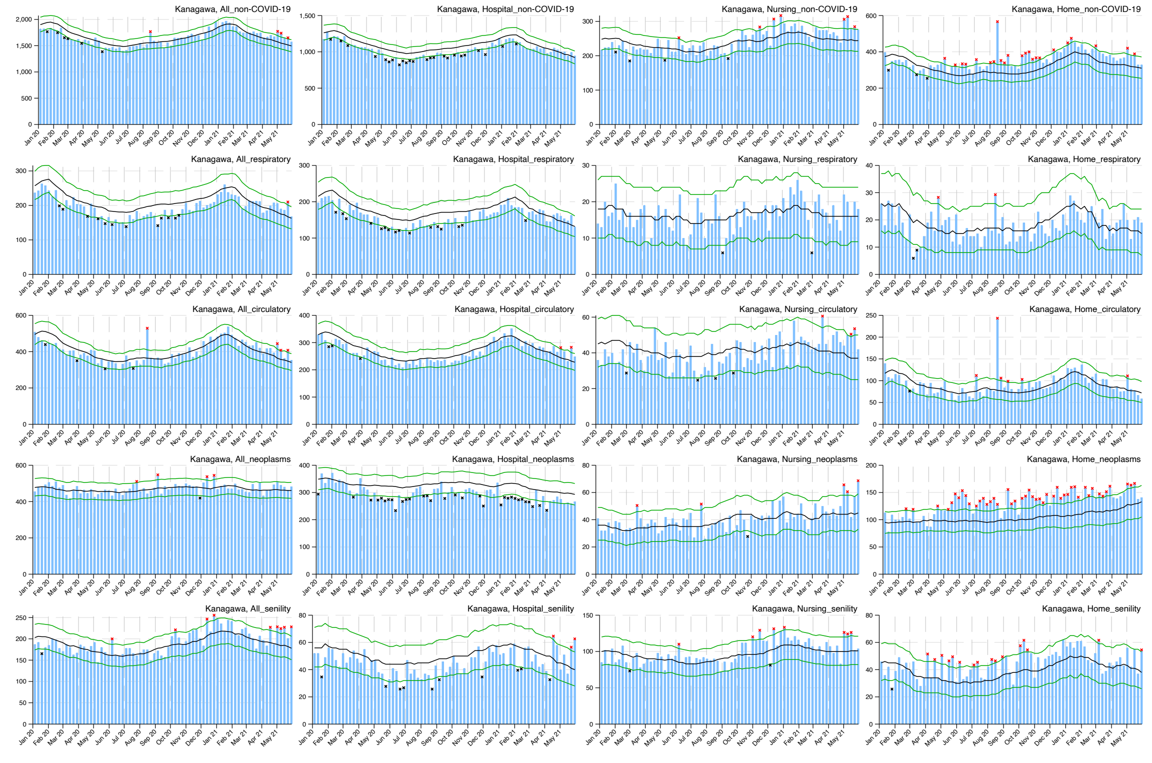
Kanagawa, All_non-COVID-19
Kanagawa, Hospital_non-COVID-19
Kanagawa, Nursing_non-COVID-19
Kanagawa, Home_non-COVID-19
Kanagawa, All_respiratory
Kanagawa, Hospital_respiratory
Kanagawa, Nursing_respiratory
Kanagawa, Home_respiratory
Kanagawa, All_circulatory
Kanagawa, Hospital_circulatory
Kanagawa, Nursing_circulatory
Kanagawa, Home_circulatory
Kanagawa, All_neoplasms
Kanagawa, Hospital_neoplasms
Kanagawa, Nursing_neoplasms
Kanagawa, Home_neoplasms
Kanagawa, All_senility
Kanagawa, Hospital_senility
Kanagawa, Nursing_senility
Kanagawa, Home_senility

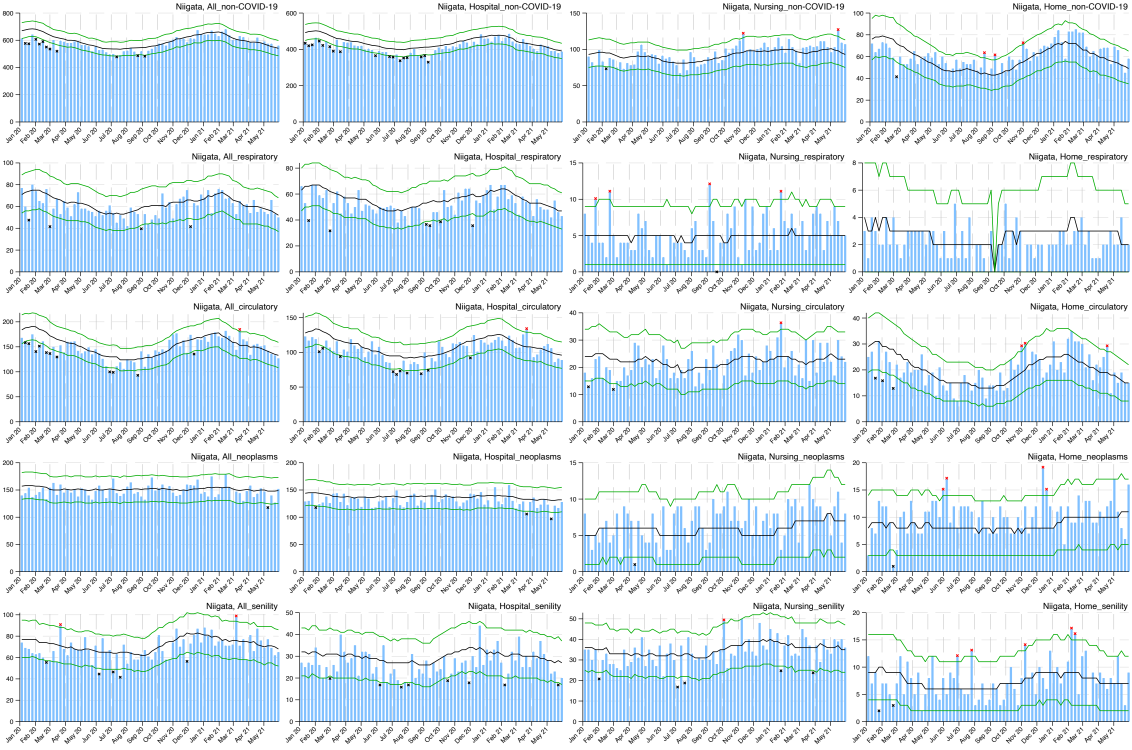
Niigata, All_non-COVID-19
Niigata, Hospital_non-COVID-19
Niigata, Nursing_non-COVID-19
Niigata, Home_non-COVID-19
Niigata, All_respiratory
Niigata, Hospital_respiratory
Niigata, Nursing_respiratory
Niigata, Home_respiratory
Niigata, All_circulatory
Niigata, Hospital_circulatory
Niigata, Nursing_circulatory
Niigata, Home_circulatory
Niigata, All_neoplasms
Niigata, Hospital_neoplasms
Niigata, Nursing_neoplasms
Niigata, Home_neoplasms
Niigata, All_senility
Niigata, Hospital_senility
Niigata, Nursing_senility
Niigata, Home_senility

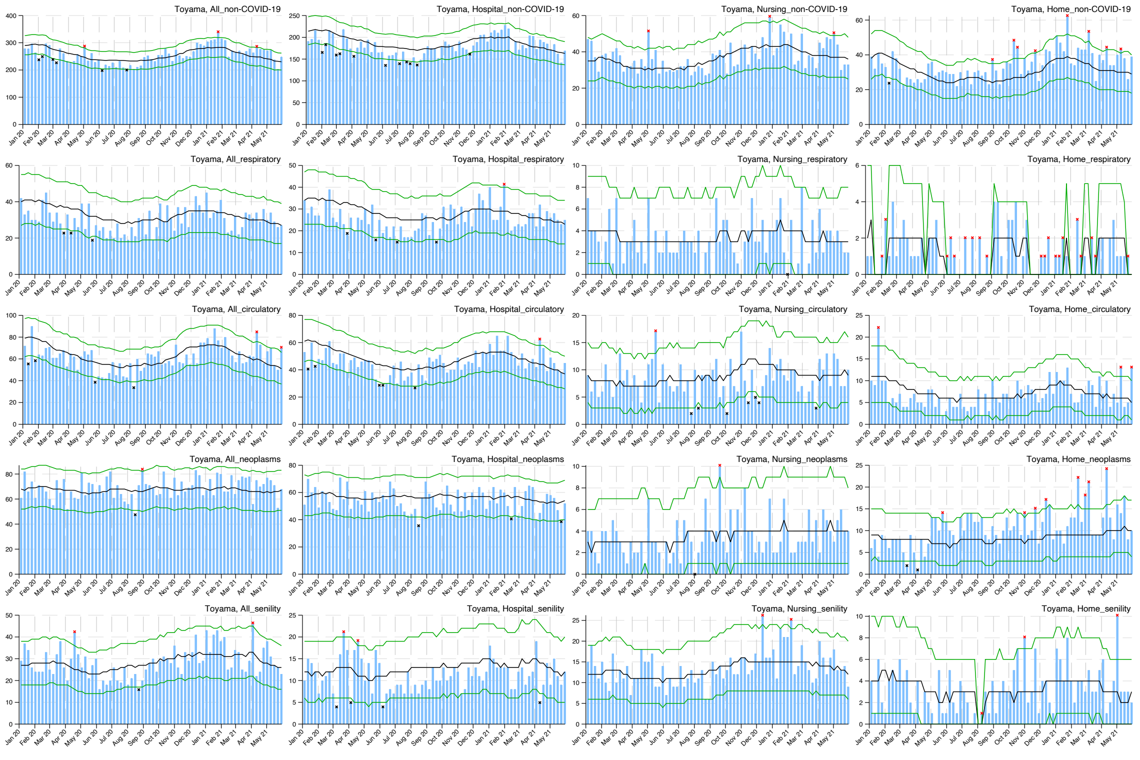
Toyama, All_non-COVID-19
Toyama, Hospital_non-COVID-19
Toyama, Nursing_non-COVID-19
Toyama, Home_non-COVID-19
Toyama, All_respiratory
Toyama, Hospital_respiratory
Toyama, Nursing_respiratory
Toyama, Home_respiratory
Toyama, All_circulatory
Toyama, Hospital_circulatory
Toyama, Nursing_circulatory
Toyama, Home_circulatory
Toyama, All_neoplasms
Toyama, Hospital_neoplasms
Toyama, Nursing_neoplasms
Toyama, Home_neoplasms
Toyama, All_senility
Toyama, Hospital_senility
Toyama, Nursing_senility
Toyama, Home_senility

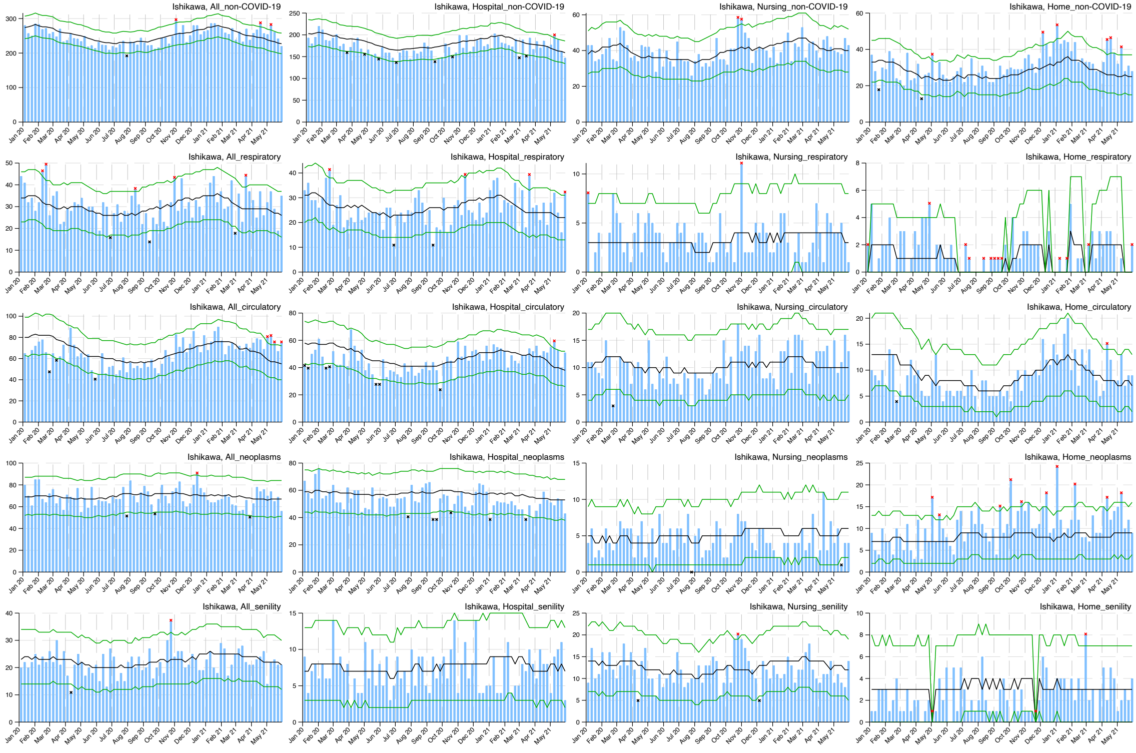
Ishikawa, All_non-COVID-19
Ishikawa, Hospital_non-COVID-19
Ishikawa, Nursing_non-COVID-19
Ishikawa, Home_non-COVID-19
Ishikawa, All_respiratory
Ishikawa, Hospital_respiratory
Ishikawa, Nursing_respiratory
Ishikawa, Home_respiratory
Ishikawa, All_circulatory
Ishikawa, Hospital_circulatory
Ishikawa, Nursing_circulatory
Ishikawa, Home_circulatory
Ishikawa, All_neoplasms
Ishikawa, Hospital_neoplasms
Ishikawa, Nursing_neoplasms
Ishikawa, Home_neoplasms
Ishikawa, All_senility
Ishikawa, Hospital_senility
Ishikawa, Nursing_senility
Ishikawa, Home_senility
Jan 20
Feb 20
Mar 20
Apr 20
May 20
Jun 20
Jul 20
Aug 20
Sep 20
Oct 20
Nov 20
Dec 20
Jan 21
Feb 21
Mar 21
Apr 21
May 21

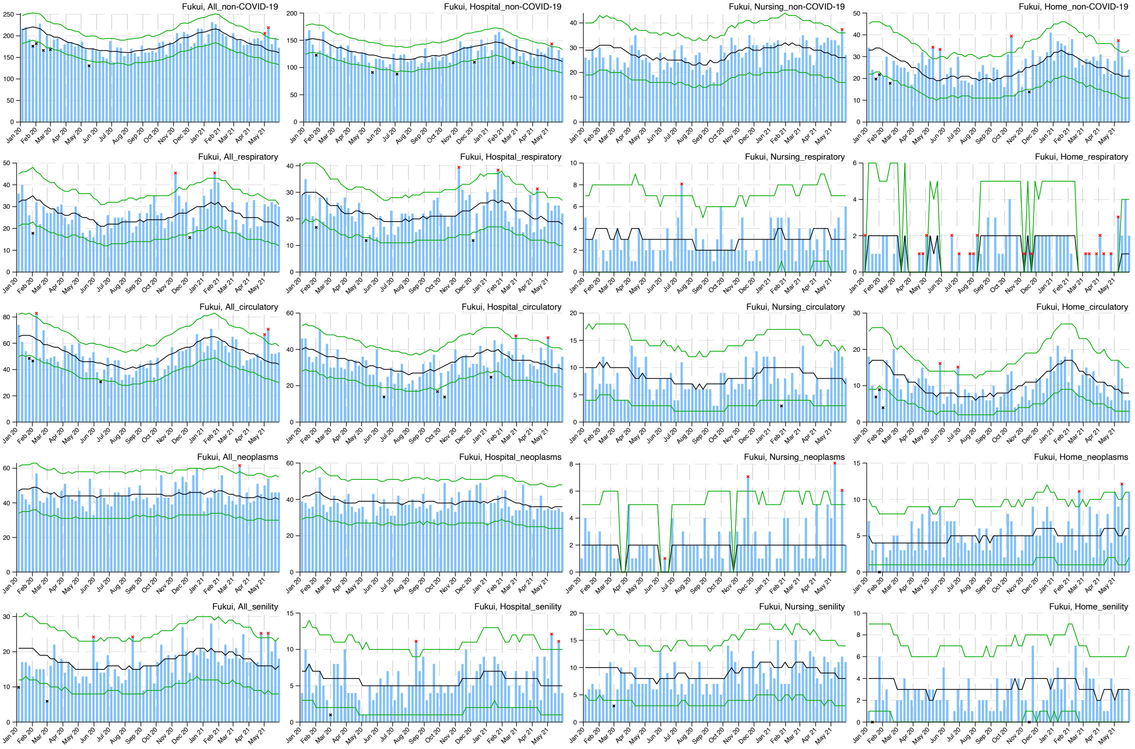
Fukui, All_non-COVID-19
Fukui, Hospital_non-COVID-19
Fukui, Nursing_non-COVID-19
Fukui, Home_non-COVID-19
Fukui, All_respiratory
Fukui, Hospital_respiratory
Fukui, Nursing_respiratory
Fukui, Home_respiratory
Fukui, All_circulatory
Fukui, Hospital_circulatory
Fukui, Nursing_circulatory
Fukui, Home_circulatory
Fukui, All_neoplasms
Fukui, Hospital_neoplasms
Fukui, Nursing_neoplasms
Fukui, Home_neoplasms
Fukui, All_senility
Fukui, Hospital_senility
Fukui, Nursing_senility
Fukui, Home_senility

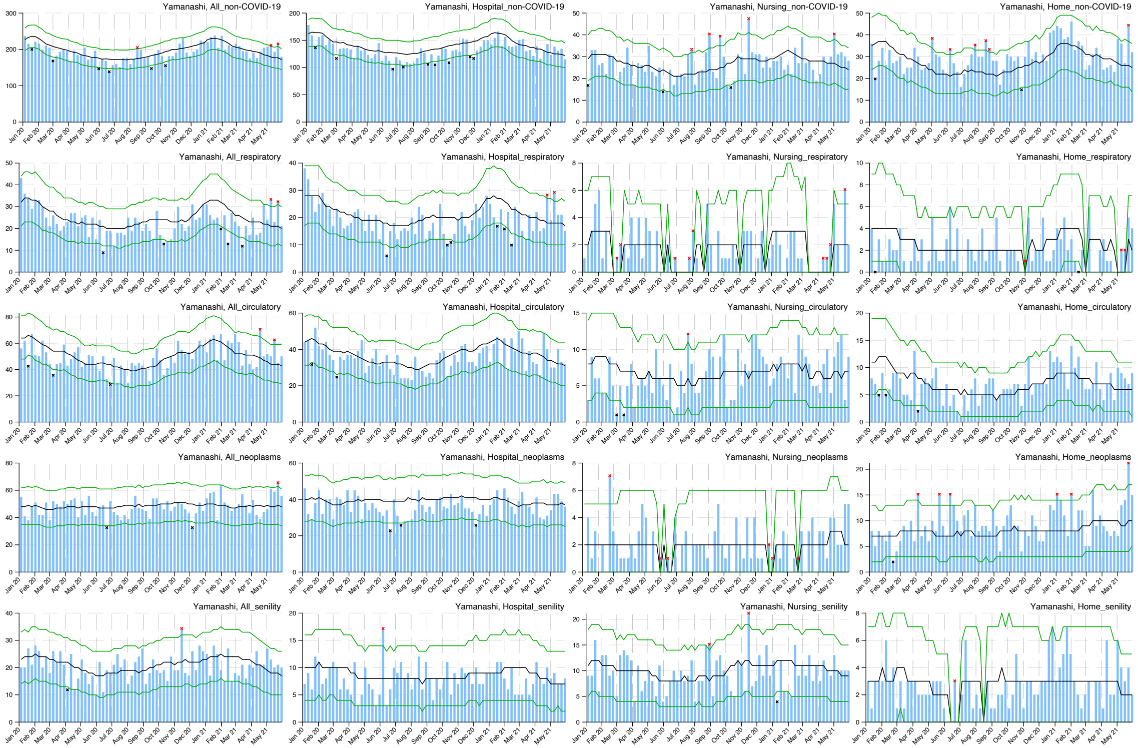
Yamanashi, All_non-COVID-19
Yamanashi, Hospital_non-COVID-19
Yamanashi, Nursing_non-COVID-19
Yamanashi, Home_non-COVID-19
Yamanashi, All_respiratory
Yamanashi, Hospital_respiratory
Yamanashi, Nursing_respiratory
Yamanashi, Home_respiratory
Yamanashi, All_circulatory
Yamanashi, Hospital_circulatory
Yamanashi, Nursing_circulatory
Yamanashi, Home_circulatory
Yamanashi, All_neoplasms
Yamanashi, Hospital_neoplasms
Yamanashi, Nursing_neoplasms
Yamanashi, Home_neoplasms
Yamanashi, All_senility
Yamanashi, Hospital_senility
Yamanashi, Nursing_senility
Yamanashi, Home_senility

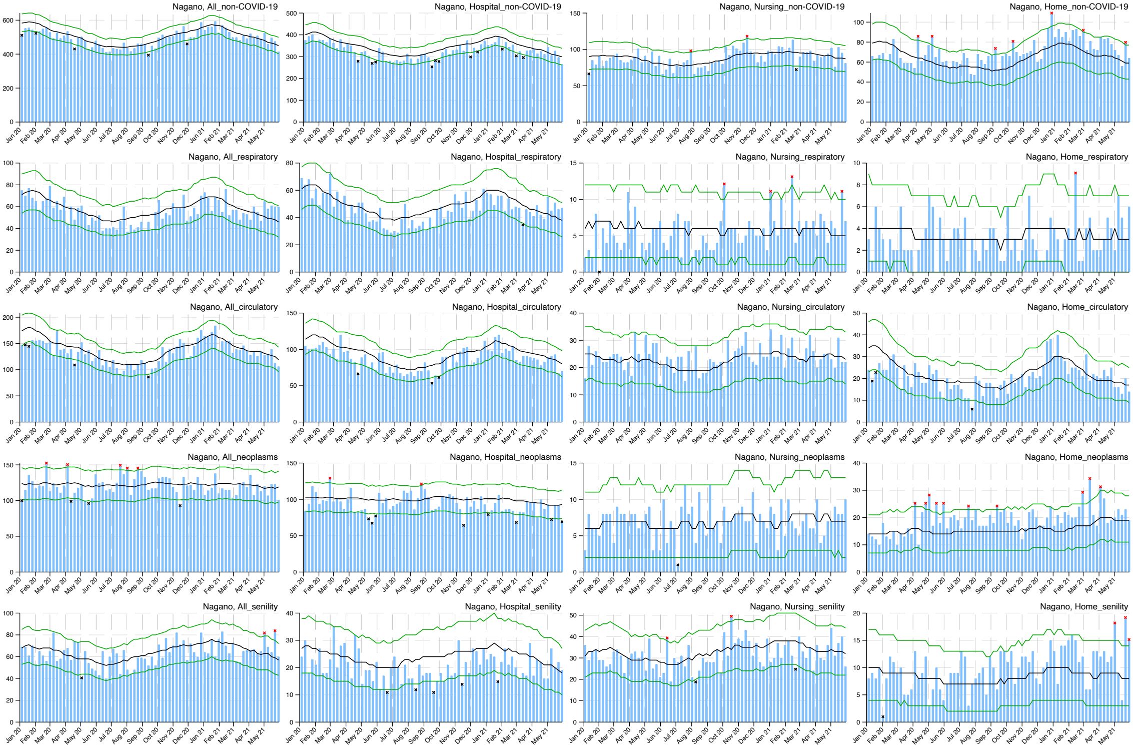
Nagano, All_non-COVID-19
Nagano, Hospital_non-COVID-19
Nagano, Nursing_non-COVID-19
Nagano, Home_non-COVID-19
Nagano, All_respiratory
Nagano, Hospital_respiratory
Nagano, Nursing_respiratory
Nagano, Home_respiratory
Nagano, All_circulatory
Nagano, Hospital_circulatory
Nagano, Nursing_circulatory
Nagano, Home_circulatory
Nagano, All_neoplasms
Nagano, Hospital_neoplasms
Nagano, Nursing_neoplasms
Nagano, Home_neoplasms
Nagano, All_senility
Nagano, Hospital_senility
Nagano, Nursing_senility
Nagano, Home_senility
Jan 20 Feb 20 Mar 20 Apr 20 May 20 Jun 20 Jul 20 Aug 20 Sep 20 Oct 20 Nov 20 Dec 20 Jan 21 Feb 21 Mar 21 Apr 21 May 21

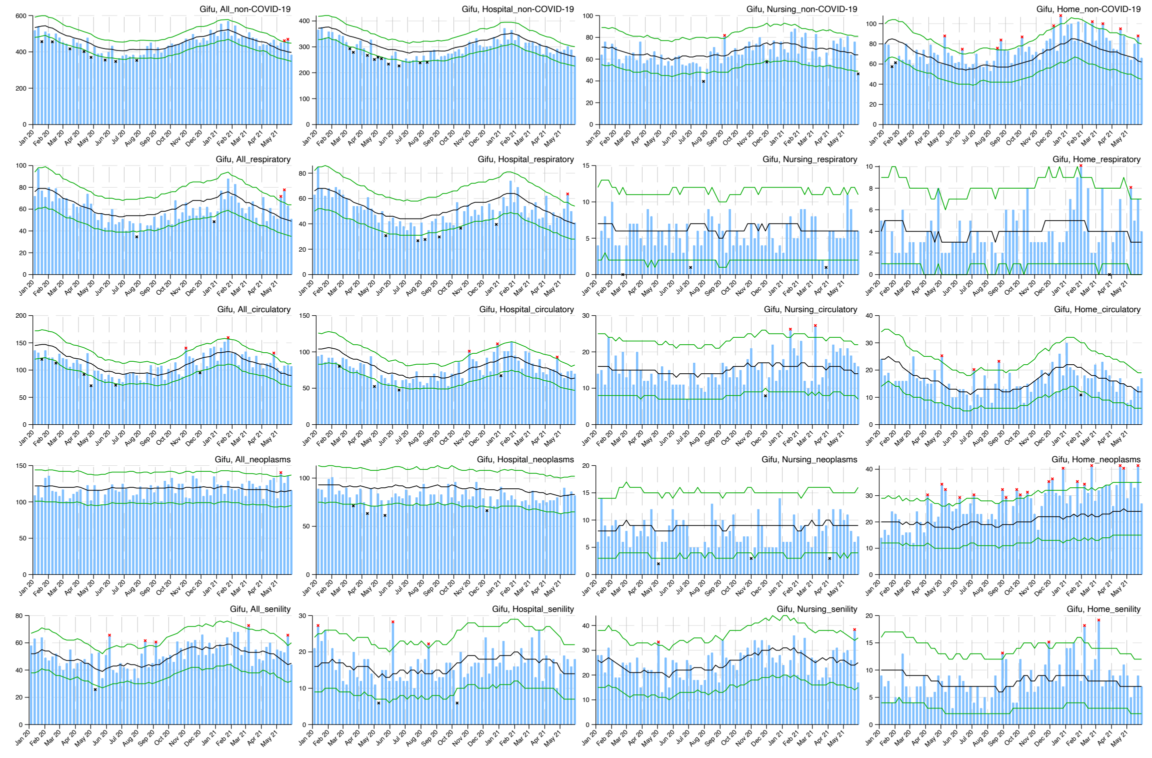
Gifu, All_non-COVID-19
Gifu, Hospital_non-COVID-19
Gifu, Nursing_non-COVID-19
Gifu, Home_non-COVID-19
Gifu, All_respiratory
Gifu, Hospital_respiratory
Gifu, Nursing_respiratory
Gifu, Home_respiratory
Gifu, All_circulatory
Gifu, Hospital_circulatory
Gifu, Nursing_circulatory
Gifu, Home_circulatory
Gifu, All_neoplasms
Gifu, Hospital_neoplasms
Gifu, Nursing_neoplasms
Gifu, Home_neoplasms
Gifu, All_senility
Gifu, Hospital_senility
Gifu, Nursing_senility
Gifu, Home_senility
Jan 20 Feb 20 Mar 20 Apr 20 May 20 Jun 20 Jul 20 Aug 20 Sep 20 Oct 20 Nov 20 Dec 20 Jan 21 Feb 21 Mar 21 Apr 21 May 21

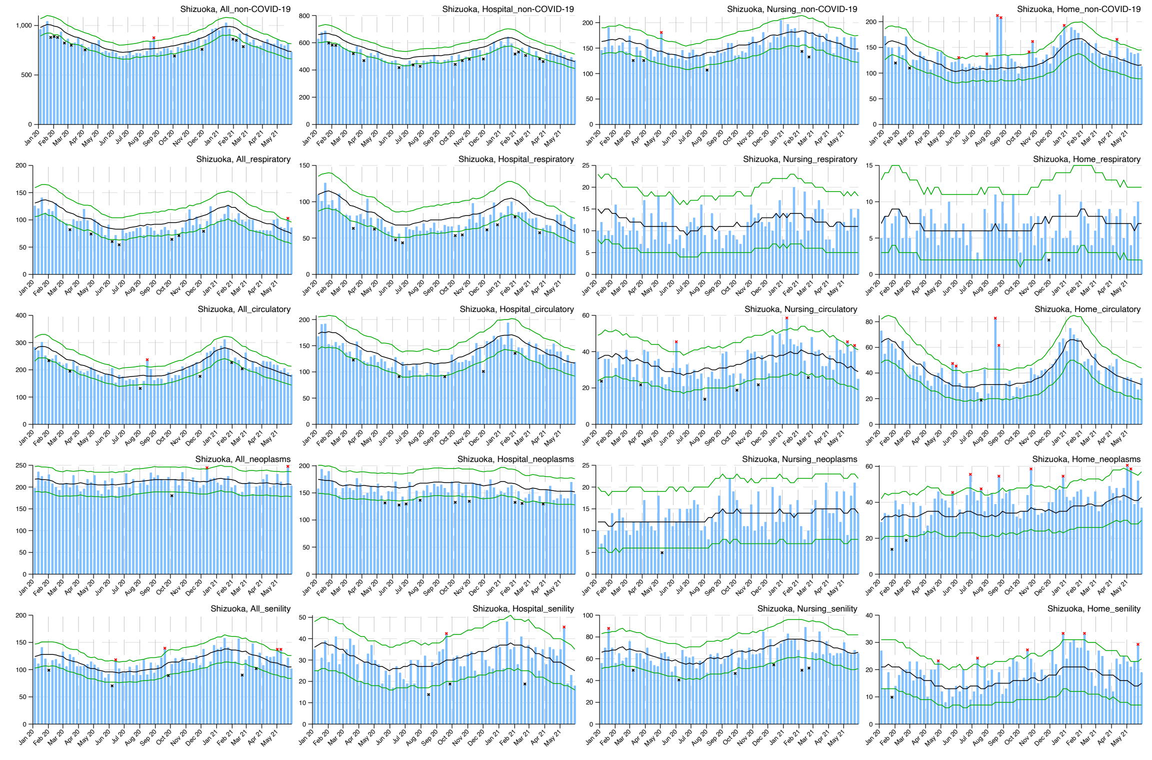
Shizuoka, All_non-COVID-19
Shizuoka, Hospital_non-COVID-19
Shizuoka, Nursing_non-COVID-19
Shizuoka, Home_non-COVID-19
Shizuoka, All_respiratory
Shizuoka, Hospital_respiratory
Shizuoka, Nursing_respiratory
Shizuoka, Home_respiratory
Shizuoka, All_circulatory
Shizuoka, Hospital_circulatory
Shizuoka, Nursing_circulatory
Shizuoka, Home_circulatory
Shizuoka, All_neoplasms
Shizuoka, Hospital_neoplasms
Shizuoka, Nursing_neoplasms
Shizuoka, Home_neoplasms
Shizuoka, All_senility
Shizuoka, Hospital_senility
Shizuoka, Nursing_senility
Shizuoka, Home_senility

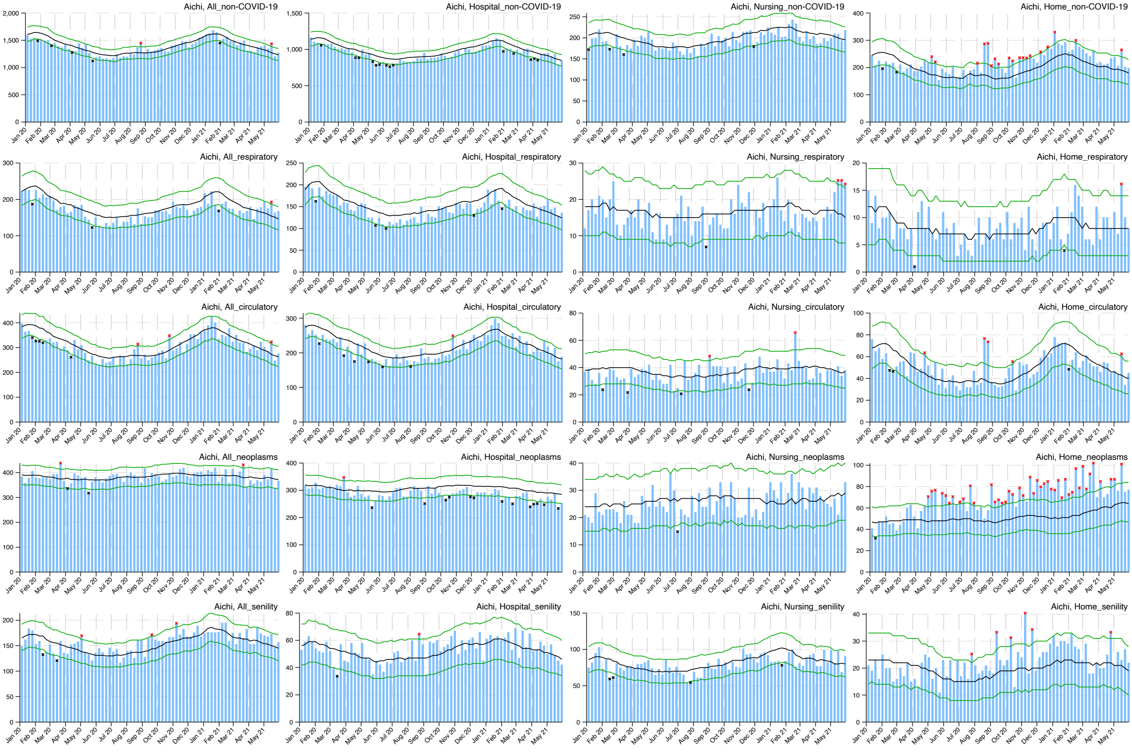
Aichi, All_non-COVID-19
Aichi, Hospital_non-COVID-19
Aichi, Nursing_non-COVID-19
Aichi, Home_non-COVID-19
Aichi, All_respiratory
Aichi, Hospital_respiratory
Aichi, Nursing_respiratory
Aichi, Home_respiratory
Aichi, All_circulatory
Aichi, Hospital_circulatory
Aichi, Nursing_circulatory
Aichi, Home_circulatory
Aichi, All_neoplasms
Aichi, Hospital_neoplasms
Aichi, Nursing_neoplasms
Aichi, Home_neoplasms
Aichi, All_senility
Aichi, Hospital_senility
Aichi, Nursing_senility
Aichi, Home_senility

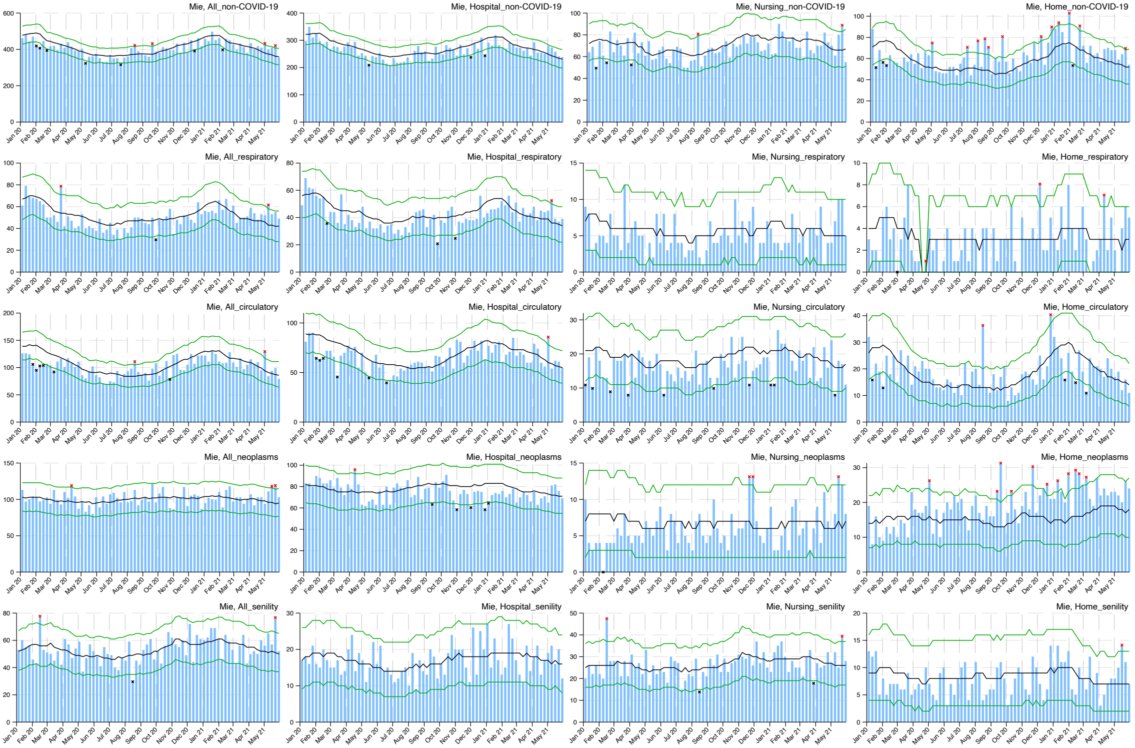
Mie, All_non-COVID-19
Mie, Hospital_non-COVID-19
Mie, Nursing_non-COVID-19
Mie, Home_non-COVID-19
Mie, All_respiratory
Mie, Hospital_respiratory
Mie, Nursing_respiratory
Mie, Home_respiratory
Mie, All_circulatory
Mie, Hospital_circulatory
Mie, Nursing_circulatory
Mie, Home_circulatory
Mie, All_neoplasms
Mie, Hospital_neoplasms
Mie, Nursing_neoplasms
Mie, Home_neoplasms
Mie, All_senility
Mie, Hospital_senility
Mie, Nursing_senility
Mie, Home_senility
Jan 20
Feb 20
Mar 20
Apr 20
May 20
Jun 20
Jul 20
Aug 20
Sep 20
Oct 20
Nov 20
Dec 20
Jan 21
Feb 21
Mar 21
Apr 21
May 21

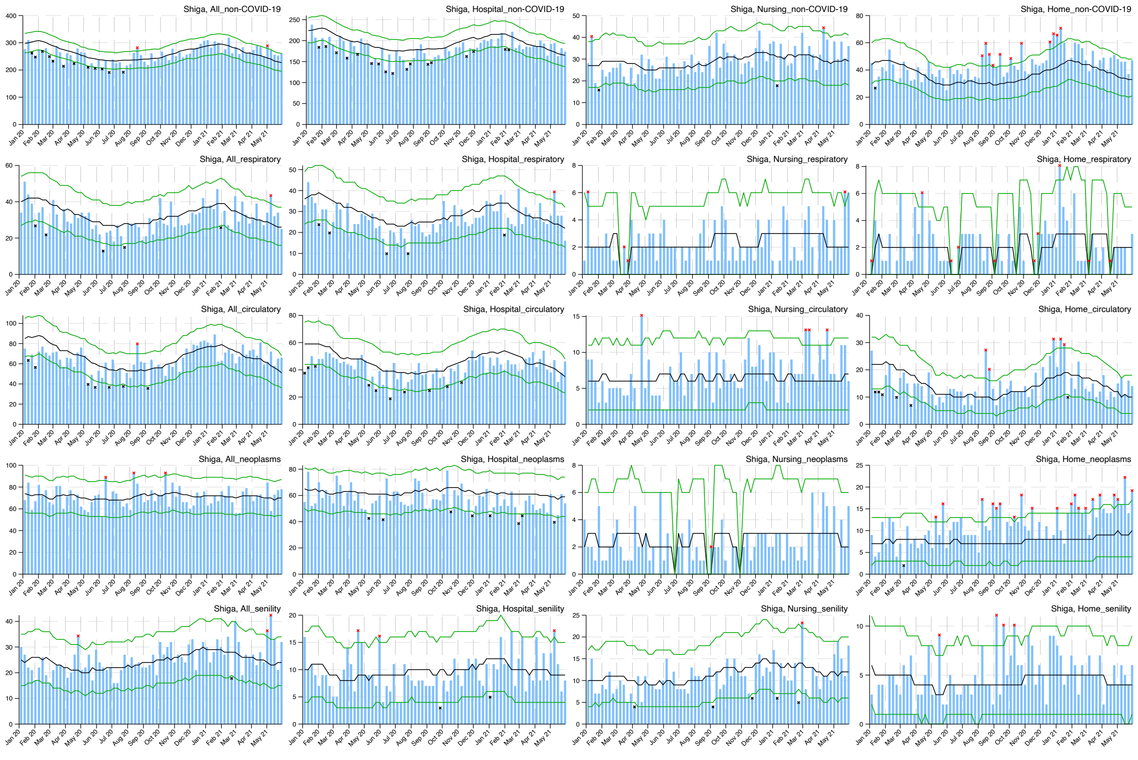
Shiga, All_non-COVID-19
Shiga, Hospital_non-COVID-19
Shiga, Nursing_non-COVID-19
Shiga, Home_non-COVID-19
Shiga, All_respiratory
Shiga, Hospital_respiratory
Shiga, Nursing_respiratory
Shiga, Home_respiratory
Shiga, All_circulatory
Shiga, Hospital_circulatory
Shiga, Nursing_circulatory
Shiga, Home_circulatory
Shiga, All_neoplasms
Shiga, Hospital_neoplasms
Shiga, Nursing_neoplasms
Shiga, Home_neoplasms
Shiga, All_senility
Shiga, Hospital_senility
Shiga, Nursing_senility
Shiga, Home_senility
Jan 20 Feb 20 Mar 20 Apr 20 May 20 Jun 20 Jul 20 Aug 20 Sep 20 Oct 20 Nov 20 Dec 20 Jan 21 Feb 21 Mar 21 Apr 21 May 21

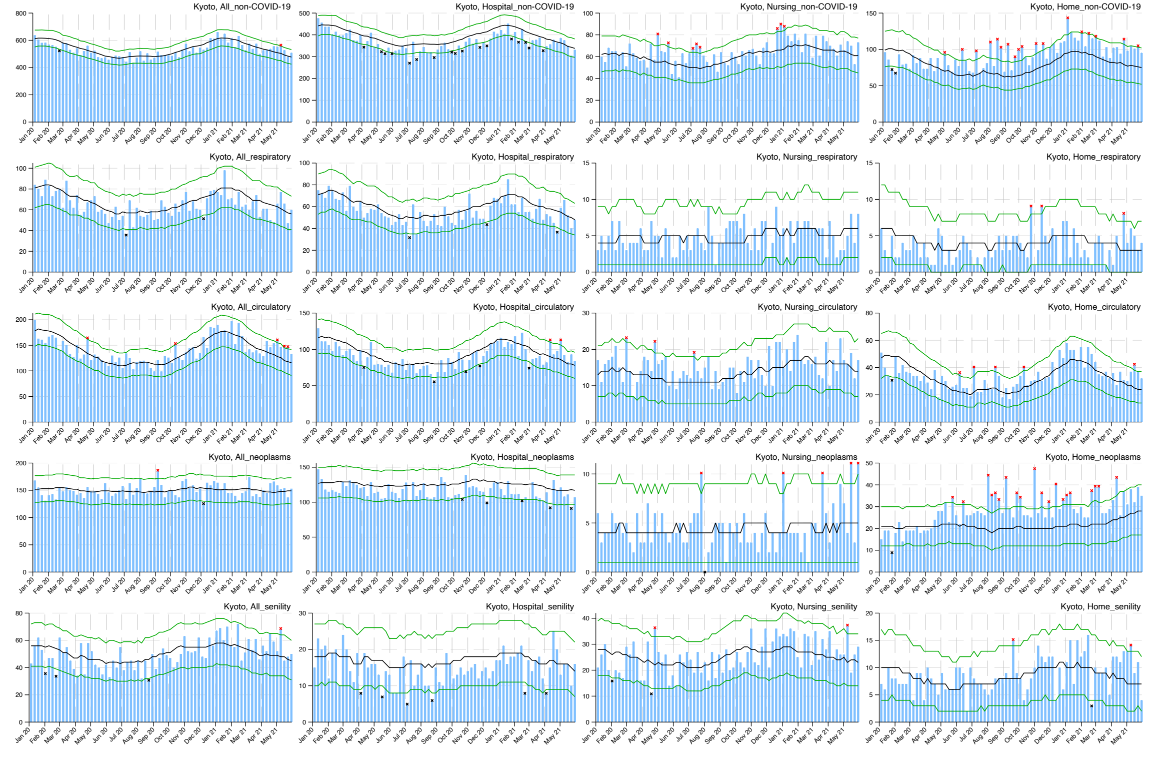
Kyoto, All_non-COVID-19
Kyoto, Hospital_non-COVID-19
Kyoto, Nursing_non-COVID-19
Kyoto, Home_non-COVID-19
Kyoto, All_respiratory
Kyoto, Hospital_respiratory
Kyoto, Nursing_respiratory
Kyoto, Home_respiratory
Kyoto, All_circulatory
Kyoto, Hospital_circulatory
Kyoto, Nursing_circulatory
Kyoto, Home_circulatory
Kyoto, All_neoplasms
Kyoto, Hospital_neoplasms
Kyoto, Nursing_neoplasms
Kyoto, Home_neoplasms
Kyoto, All_senility
Kyoto, Hospital_senility
Kyoto, Nursing_senility
Kyoto, Home_senility
Jan 20 Feb 20 Mar 20 Apr 20 May 20 Jun 20 Jul 20 Aug 20 Sep 20 Oct 20 Nov 20 Dec 20 Jan 21 Feb 21 Mar 21 Apr 21 May 21

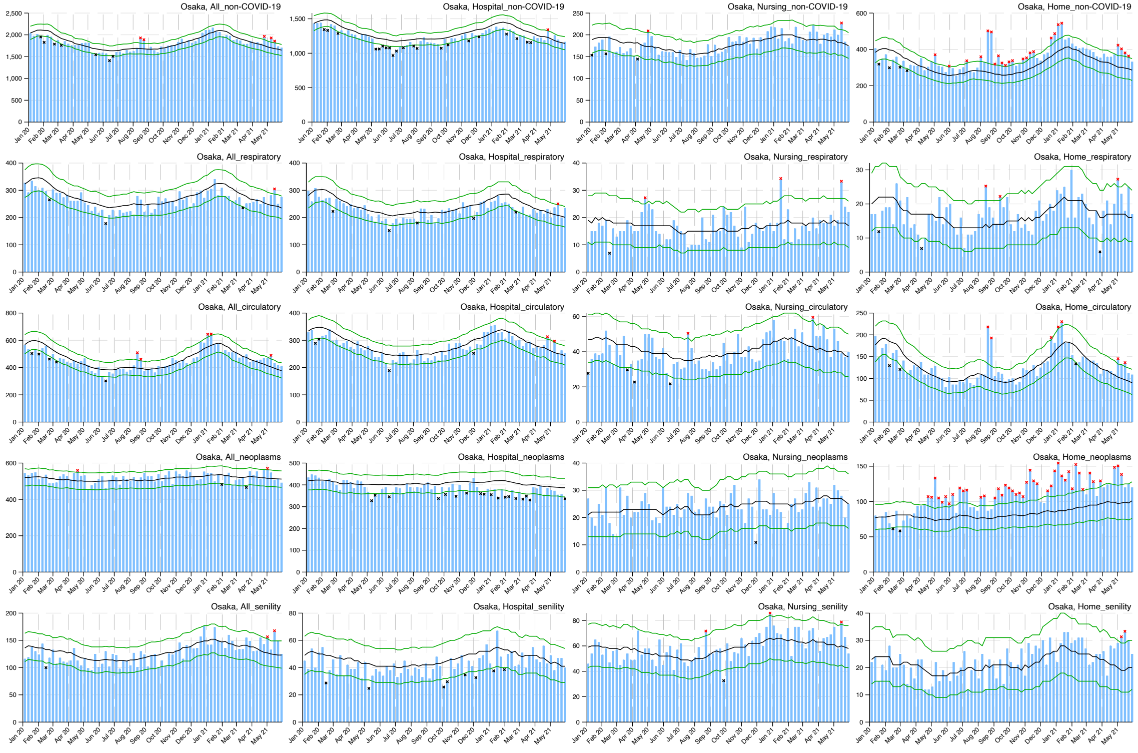
Osaka, All_non-COVID-19
Osaka, Hospital_non-COVID-19
Osaka, Nursing_non-COVID-19
Osaka, Home_non-COVID-19
Osaka, All_respiratory
Osaka, Hospital_respiratory
Osaka, Nursing_respiratory
Osaka, Home_respiratory
Osaka, All_circulatory
Osaka, Hospital_circulatory
Osaka, Nursing_circulatory
Osaka, Home_circulatory
Osaka, All_neoplasms
Osaka, Hospital_neoplasms
Osaka, Nursing_neoplasms
Osaka, Home_neoplasms
Osaka, All_senility
Osaka, Hospital_senility
Osaka, Nursing_senility
Osaka, Home_senility

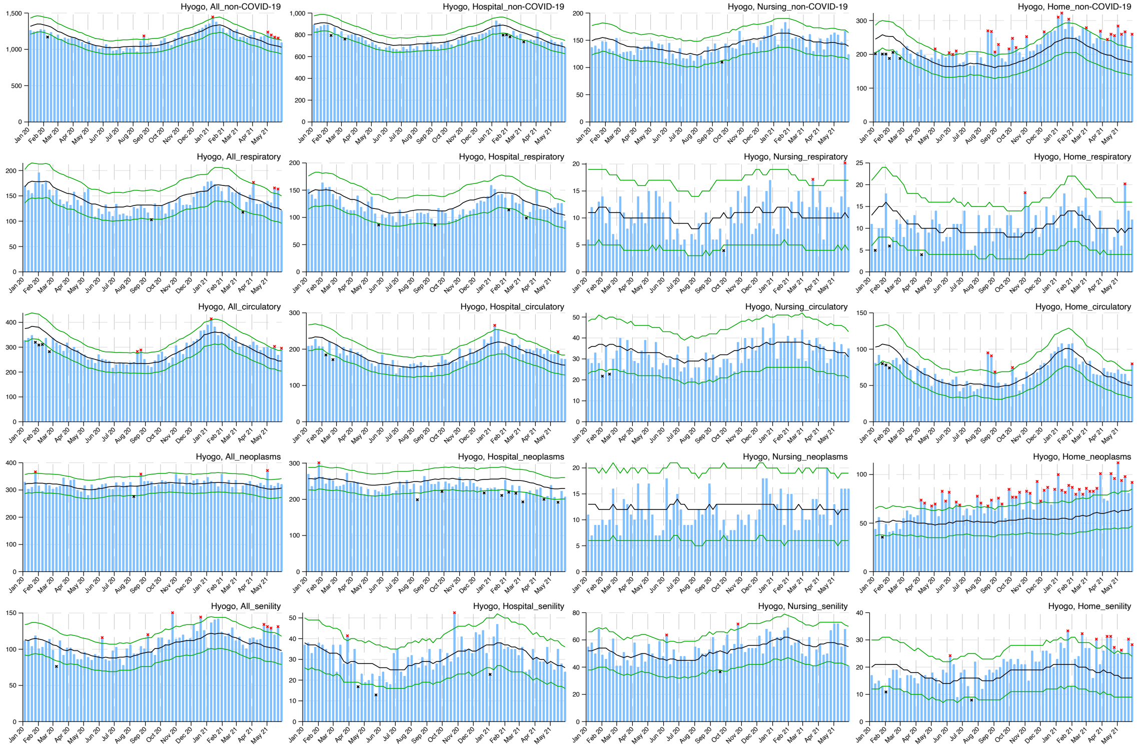
Hyogo, All_non-COVID-19
Hyogo, Hospital_non-COVID-19
Hyogo, Nursing_non-COVID-19
Hyogo, Home_non-COVID-19
Hyogo, All_respiratory
Hyogo, Hospital_respiratory
Hyogo, Nursing_respiratory
Hyogo, Home_respiratory
Hyogo, All_circulatory
Hyogo, Hospital_circulatory
Hyogo, Nursing_circulatory
Hyogo, Home_circulatory
Hyogo, All_neoplasms
Hyogo, Hospital_neoplasms
Hyogo, Nursing_neoplasms
Hyogo, Home_neoplasms
Hyogo, All_senility
Hyogo, Hospital_senility
Hyogo, Nursing_senility
Hyogo, Home_senility

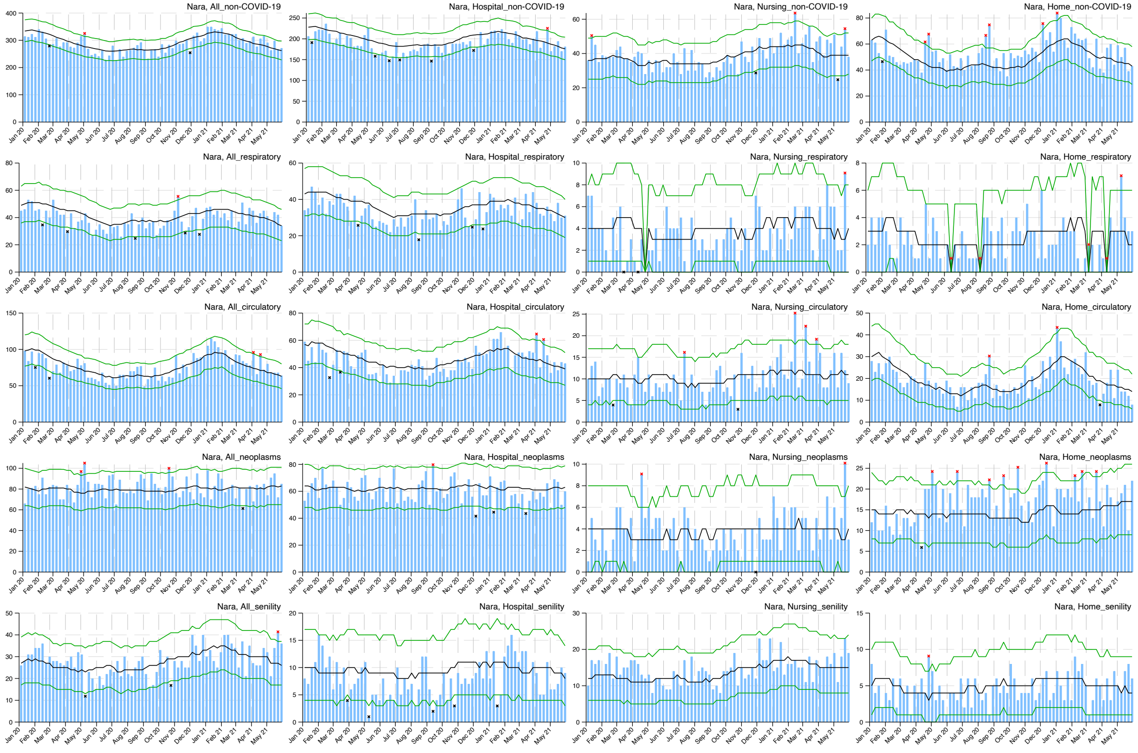
Nara, All_non-COVID-19
Nara, Hospital_non-COVID-19
Nara, Nursing_non-COVID-19
Nara, Home_non-COVID-19
Nara, All_respiratory
Nara, Hospital_respiratory
Nara, Nursing_respiratory
Nara, Home_respiratory
Nara, All_circulatory
Nara, Hospital_circulatory
Nara, Nursing_circulatory
Nara, Home_circulatory
Nara, All_neoplasms
Nara, Hospital_neoplasms
Nara, Nursing_neoplasms
Nara, Home_neoplasms
Nara, All_senility
Nara, Hospital_senility
Nara, Nursing_senility
Nara, Home_senility

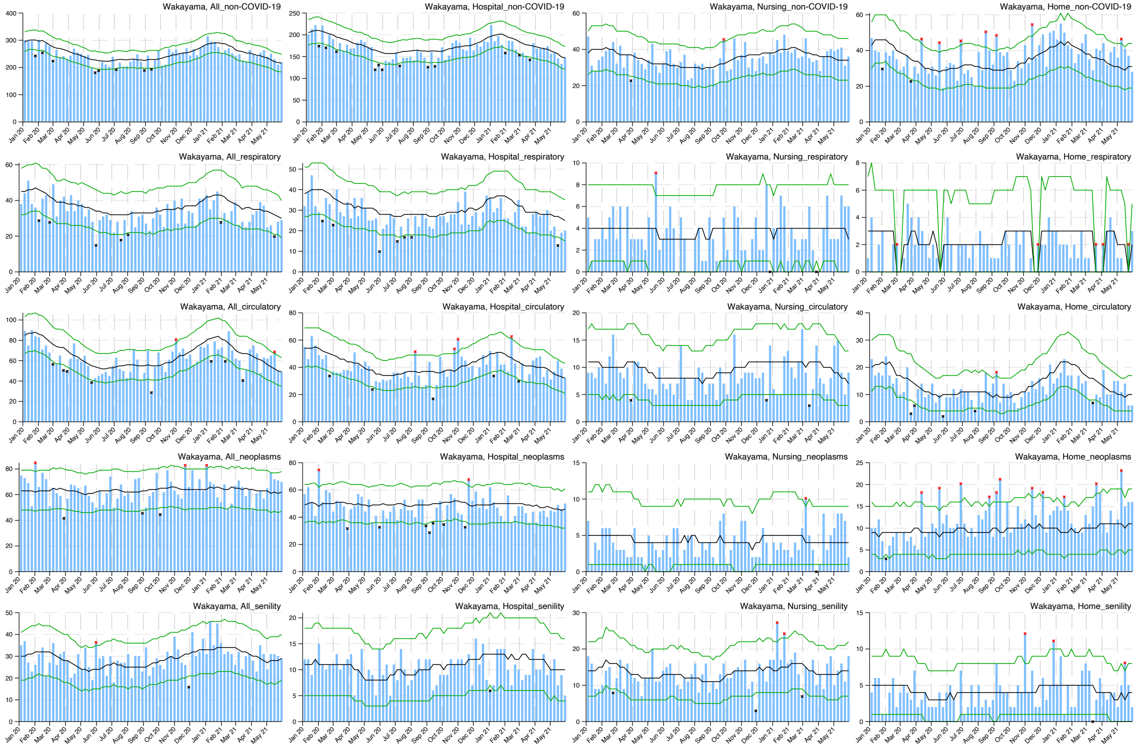
Wakayama, All_non-COVID-19
Wakayama, Hospital_non-COVID-19
Wakayama, Nursing_non-COVID-19
Wakayama, Home_non-COVID-19
Wakayama, All_respiratory
Wakayama, Hospital_respiratory
Wakayama, Nursing_respiratory
Wakayama, Home_respiratory
Wakayama, All_circulatory
Wakayama, Hospital_circulatory
Wakayama, Nursing_circulatory
Wakayama, Home_circulatory
Wakayama, All_neoplasms
Wakayama, Hospital_neoplasms
Wakayama, Nursing_neoplasms
Wakayama, Home_neoplasms
Wakayama, All_senility
Wakayama, Hospital_senility
Wakayama, Nursing_senility
Wakayama, Home_senility
Jan 20
Feb 20
Mar 20
Apr 20
May 20
Jun 20
Jul 20
Aug 20
Sep 20
Oct 20
Nov 20
Dec 20
Jan 21
Feb 21
Mar 21
Apr 21
May 21

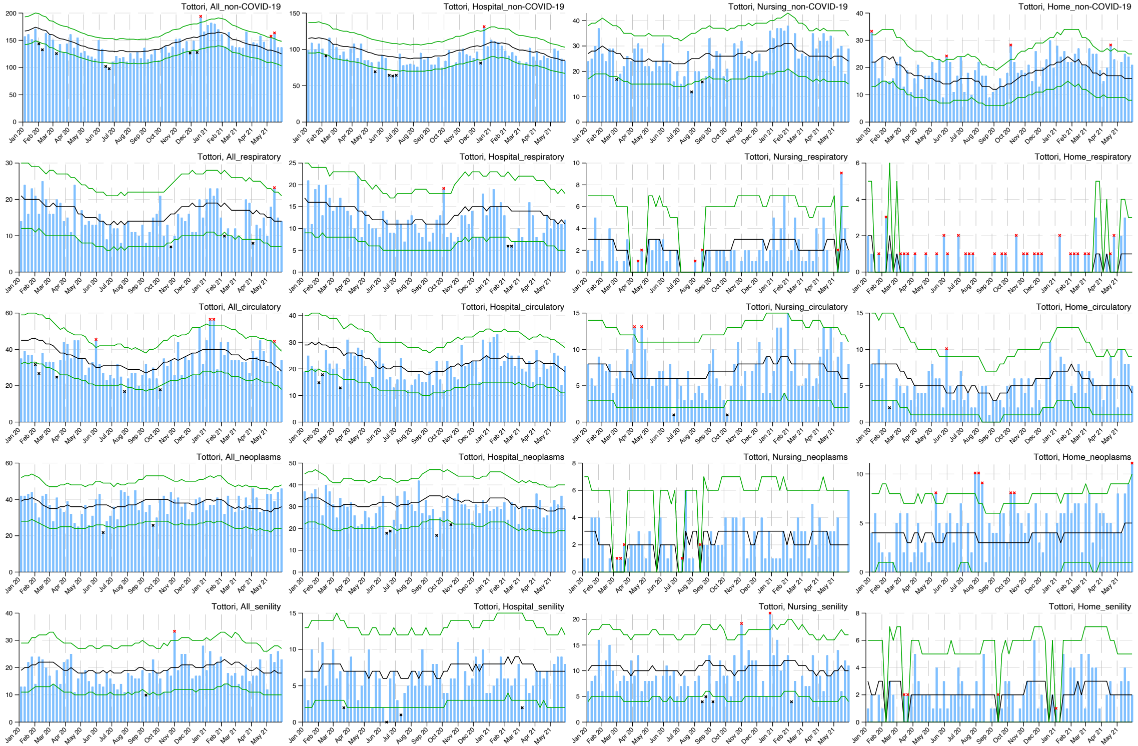
Tottori, All_non-COVID-19
Tottori, Hospital_non-COVID-19
Tottori, Nursing_non-COVID-19
Tottori, Home_non-COVID-19
Tottori, All_respiratory
Tottori, Hospital_respiratory
Tottori, Nursing_respiratory
Tottori, Home_respiratory
Tottori, All_circulatory
Tottori, Hospital_circulatory
Tottori, Nursing_circulatory
Tottori, Home_circulatory
Tottori, All_neoplasms
Tottori, Hospital_neoplasms
Tottori, Nursing_neoplasms
Tottori, Home_neoplasms
Tottori, All_senility
Tottori, Hospital_senility
Tottori, Nursing_senility
Tottori, Home_senility

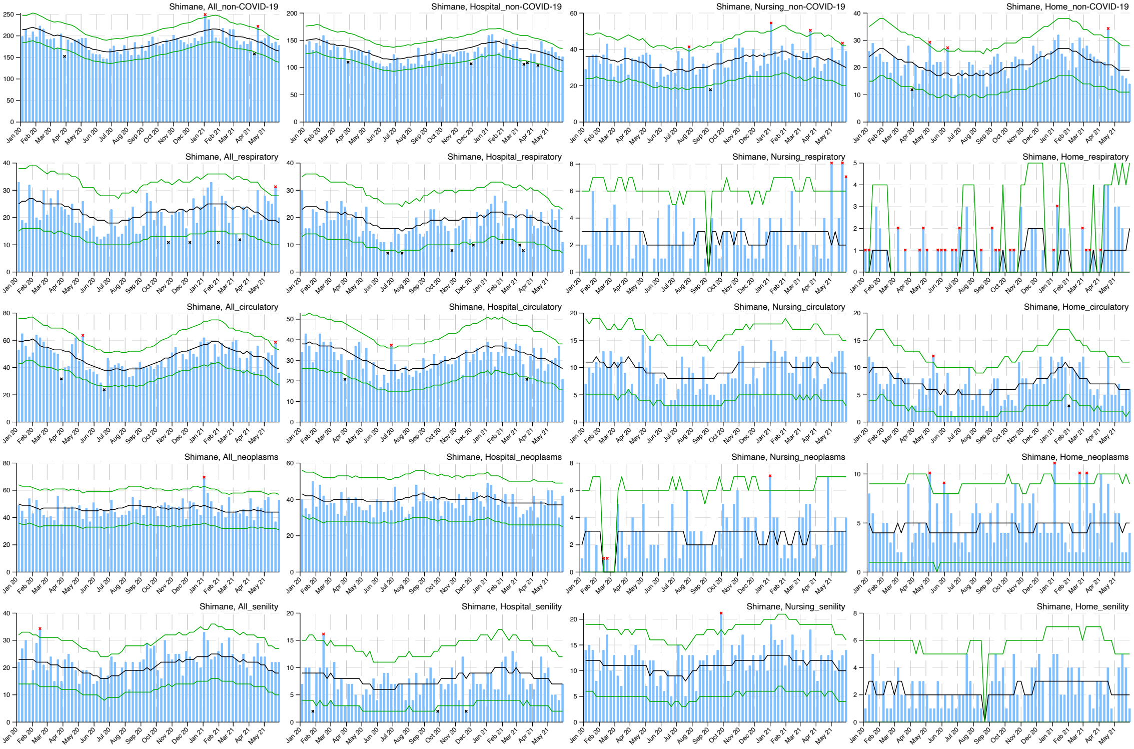
Shimane, All_non-COVID-19
Shimane, Hospital_non-COVID-19
Shimane, Nursing_non-COVID-19
Shimane, Home_non-COVID-19
Shimane, All_respiratory
Shimane, Hospital_respiratory
Shimane, Nursing_respiratory
Shimane, Home_respiratory
Shimane, All_circulatory
Shimane, Hospital_circulatory
Shimane, Nursing_circulatory
Shimane, Home_circulatory
Shimane, All_neoplasms
Shimane, Hospital_neoplasms
Shimane, Nursing_neoplasms
Shimane, Home_neoplasms
Shimane, All_senility
Shimane, Hospital_senility
Shimane, Nursing_senility
Shimane, Home_senility

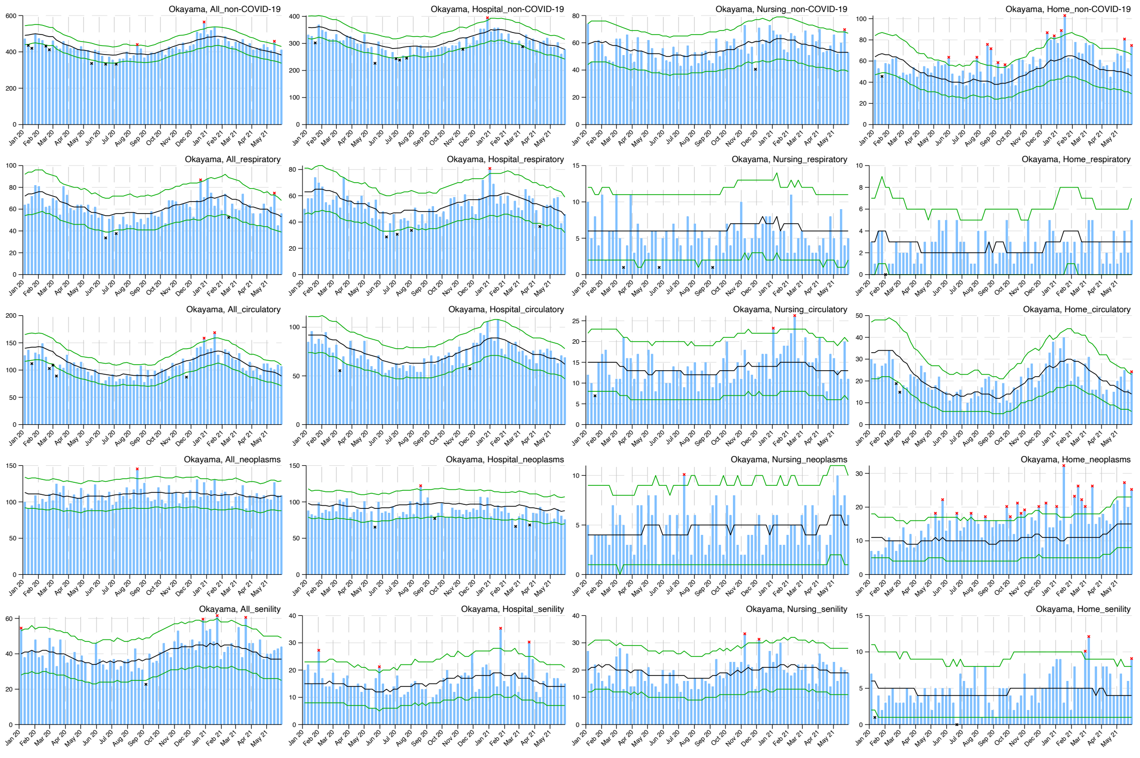
Okayama, All_non-COVID-19
Okayama, Hospital_non-COVID-19
Okayama, Nursing_non-COVID-19
Okayama, Home_non-COVID-19
Okayama, All_respiratory
Okayama, Hospital_respiratory
Okayama, Nursing_respiratory
Okayama, Home_respiratory
Okayama, All_circulatory
Okayama, Hospital_circulatory
Okayama, Nursing_circulatory
Okayama, Home_circulatory
Okayama, All_neoplasms
Okayama, Hospital_neoplasms
Okayama, Nursing_neoplasms
Okayama, Home_neoplasms
Okayama, All_senility
Okayama, Hospital_senility
Okayama, Nursing_senility
Okayama, Home_senility
Jan 20
Feb 20
Mar 20
Apr 20
May 20
Jun 20
Jul 20
Aug 20
Sep 20
Oct 20
Nov 20
Dec 20
Jan 21
Feb 21
Mar 21
Apr 21
May 21

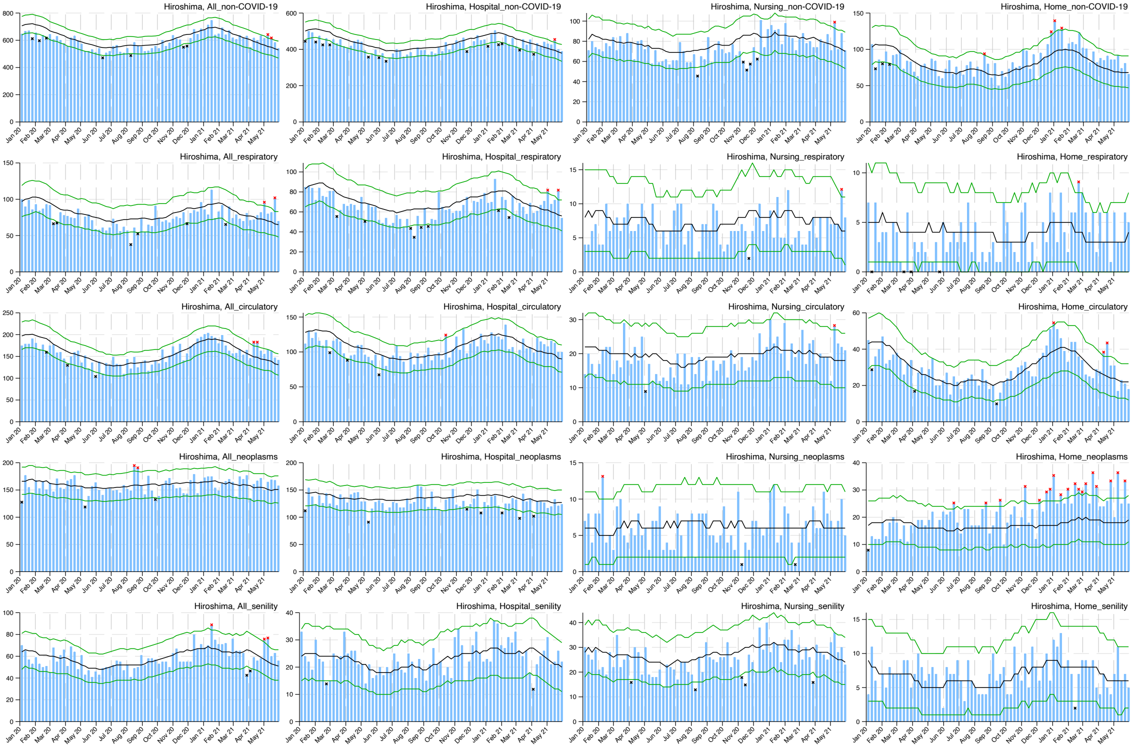

Hiroshima, All_non-COVID-19
Hiroshima, Hospital_non-COVID-19
Hiroshima, Nursing_non-COVID-19
Hiroshima, Home_non-COVID-19
Hiroshima, All_respiratory
Hiroshima, Hospital_respiratory
Hiroshima, Nursing_respiratory
Hiroshima, Home_respiratory
Hiroshima, All_circulatory
Hiroshima, Hospital_circulatory
Hiroshima, Nursing_circulatory
Hiroshima, Home_circulatory
Hiroshima, All_neoplasms
Hiroshima, Hospital_neoplasms
Hiroshima, Nursing_neoplasms
Hiroshima, Home_neoplasms
Hiroshima, All_senility
Hiroshima, Hospital_senility
Hiroshima, Nursing_senility
Hiroshima, Home_senility
Jan 20
Feb 20
Mar 20
Apr 20
May 20
Jun 20
Jul 20
Aug 20
Sep 20
Oct 20
Nov 20
Dec 20
Jan 21
Feb 21
Mar 21
Apr 21
May 21

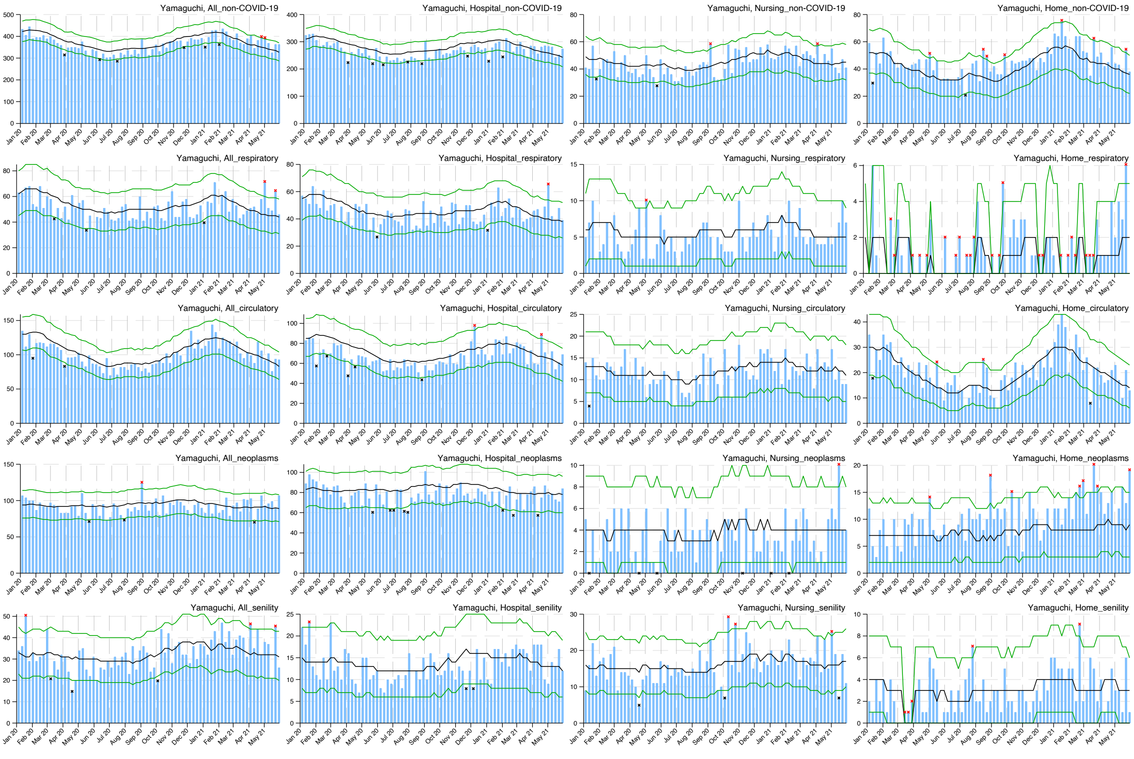
Yamaguchi, All_non-COVID-19
Yamaguchi, Hospital_non-COVID-19
Yamaguchi, Nursing_non-COVID-19
Yamaguchi, Home_non-COVID-19
Yamaguchi, All_respiratory
Yamaguchi, Hospital_respiratory
Yamaguchi, Nursing_respiratory
Yamaguchi, Home_respiratory
Yamaguchi, All_circulatory
Yamaguchi, Hospital_circulatory
Yamaguchi, Nursing_circulatory
Yamaguchi, Home_circulatory
Yamaguchi, All_neoplasms
Yamaguchi, Hospital_neoplasms
Yamaguchi, Nursing_neoplasms
Yamaguchi, Home_neoplasms
Yamaguchi, All_senility
Yamaguchi, Hospital_senility
Yamaguchi, Nursing_senility
Yamaguchi, Home_senility

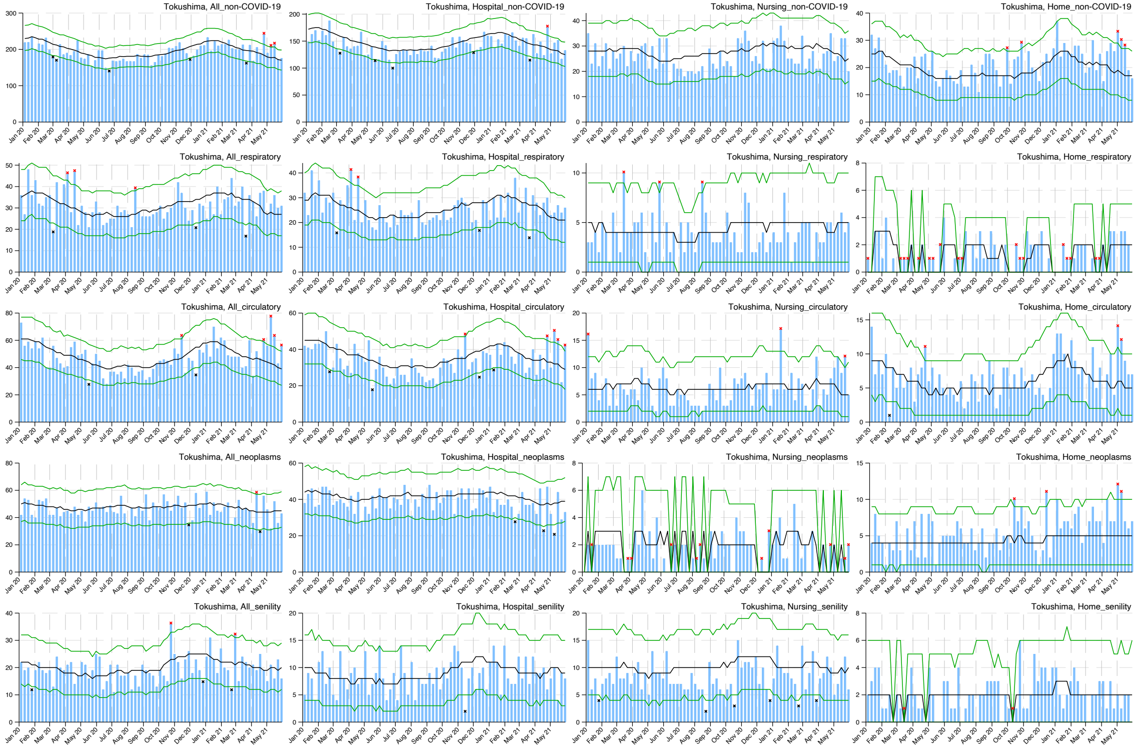
Tokushima, All_non-COVID-19
Tokushima, Hospital_non-COVID-19
Tokushima, Nursing_non-COVID-19
Tokushima, Home_non-COVID-19
Tokushima, All_respiratory
Tokushima, Hospital_respiratory
Tokushima, Nursing_respiratory
Tokushima, Home_respiratory
Tokushima, All_circulatory
Tokushima, Hospital_circulatory
Tokushima, Nursing_circulatory
Tokushima, Home_circulatory
Tokushima, All_neoplasms
Tokushima, Hospital_neoplasms
Tokushima, Nursing_neoplasms
Tokushima, Home_neoplasms
Tokushima, All_senility
Tokushima, Hospital_senility
Tokushima, Nursing_senility
Tokushima, Home_senility

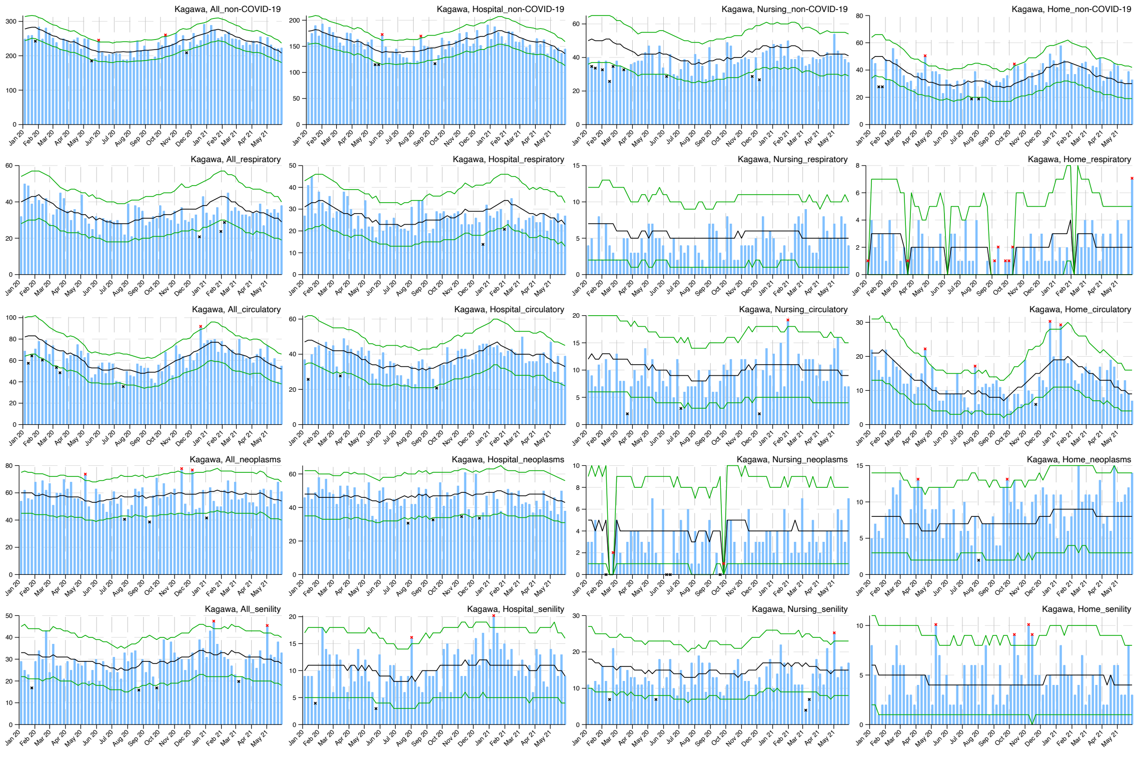
Kagawa, All_non-COVID-19
Kagawa, Hospital_non-COVID-19
Kagawa, Nursing_non-COVID-19
Kagawa, Home_non-COVID-19
Kagawa, All_respiratory
Kagawa, Hospital_respiratory
Kagawa, Nursing_respiratory
Kagawa, Home_respiratory
Kagawa, All_circulatory
Kagawa, Hospital_circulatory
Kagawa, Nursing_circulatory
Kagawa, Home_circulatory
Kagawa, All_neoplasms
Kagawa, Hospital_neoplasms
Kagawa, Nursing_neoplasms
Kagawa, Home_neoplasms
Kagawa, All_senility
Kagawa, Hospital_senility
Kagawa, Nursing_senility
Kagawa, Home_senility
Jan 20 Feb 20 Mar 20 Apr 20 May 20 Jun 20 Jul 20 Aug 20 Sep 20 Oct 20 Nov 20 Dec 20 Jan 21 Feb 21 Mar 21 Apr 21 May 21

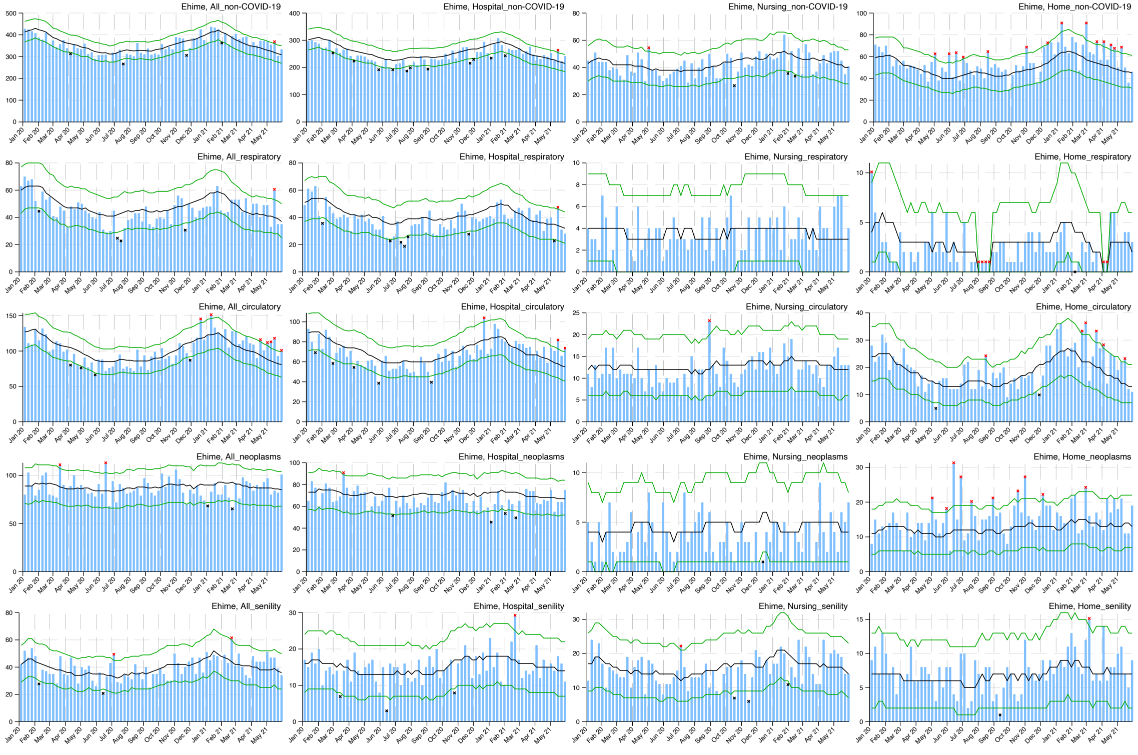
Ehime, All_non-COVID-19
Ehime, Hospital_non-COVID-19
Ehime, Nursing_non-COVID-19
Ehime, Home_non-COVID-19
Ehime, All_respiratory
Ehime, Hospital_respiratory
Ehime, Nursing_respiratory
Ehime, Home_respiratory
Ehime, All_circulatory
Ehime, Hospital_circulatory
Ehime, Nursing_circulatory
Ehime, Home_circulatory
Ehime, All_neoplasms
Ehime, Hospital_neoplasms
Ehime, Nursing_neoplasms
Ehime, Home_neoplasms
Ehime, All_senility
Ehime, Hospital_senility
Ehime, Nursing_senility
Ehime, Home_senility
Jan 20
Feb 20
Mar 20
Apr 20
May 20
Jun 20
Jul 20
Aug 20
Sep 20
Oct 20
Nov 20
Dec 20
Jan 21
Feb 21
Mar 21
Apr 21
May 21

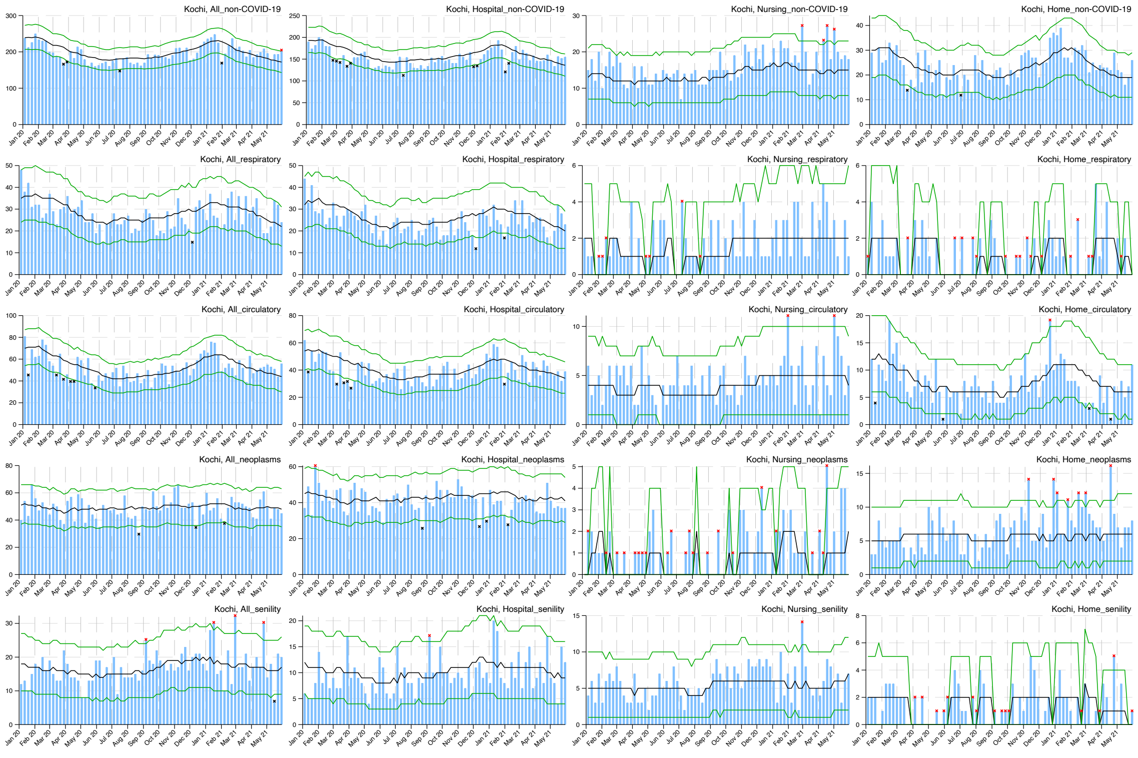
Kochi, All_non-COVID-19
Kochi, Hospital_non-COVID-19
Kochi, Nursing_non-COVID-19
Kochi, Home_non-COVID-19
Kochi, All_respiratory
Kochi, Hospital_respiratory
Kochi, Nursing_respiratory
Kochi, Home_respiratory
Kochi, All_circulatory
Kochi, Hospital_circulatory
Kochi, Nursing_circulatory
Kochi, Home_circulatory
Kochi, All_neoplasms
Kochi, Hospital_neoplasms
Kochi, Nursing_neoplasms
Kochi, Home_neoplasms
Kochi, All_senility
Kochi, Hospital_senility
Kochi, Nursing_senility
Kochi, Home_senility

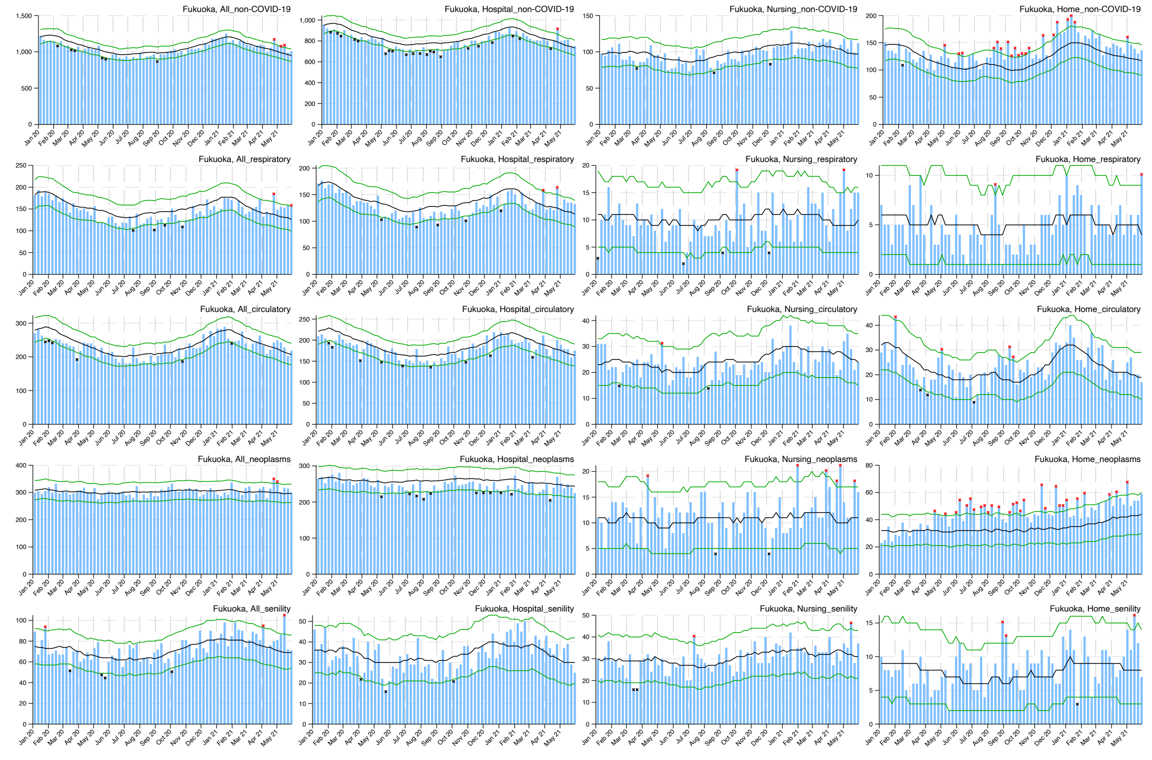
Fukuoka, All_non-COVID-19
Fukuoka, Hospital_non-COVID-19
Fukuoka, Nursing_non-COVID-19
Fukuoka, Home_non-COVID-19
Fukuoka, All_respiratory
Fukuoka, Hospital_respiratory
Fukuoka, Nursing_respiratory
Fukuoka, Home_respiratory
Fukuoka, All_circulatory
Fukuoka, Hospital_circulatory
Fukuoka, Nursing_circulatory
Fukuoka, Home_circulatory
Fukuoka, All_neoplasms
Fukuoka, Hospital_neoplasms
Fukuoka, Nursing_neoplasms
Fukuoka, Home_neoplasms
Fukuoka, All_senility
Fukuoka, Hospital_senility
Fukuoka, Nursing_senility
Fukuoka, Home_senility

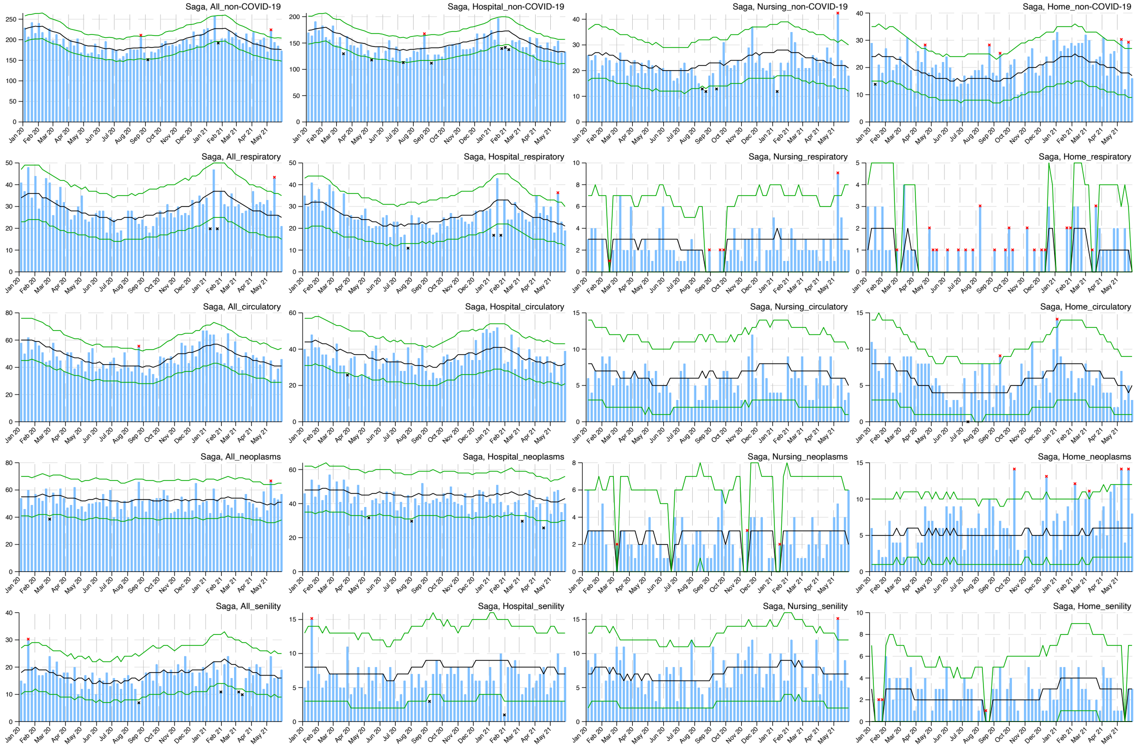
Saga, All_non-COVID-19
Saga, Hospital_non-COVID-19
Saga, Nursing_non-COVID-19
Saga, Home_non-COVID-19
Saga, All_respiratory
Saga, Hospital_respiratory
Saga, Nursing_respiratory
Saga, Home_respiratory
Saga, All_circulatory
Saga, Hospital_circulatory
Saga, Nursing_circulatory
Saga, Home_circulatory
Saga, All_neoplasms
Saga, Hospital_neoplasms
Saga, Nursing_neoplasms
Saga, Home_neoplasms
Saga, All_senility
Saga, Hospital_senility
Saga, Nursing_senility
Saga, Home_senility
Jan 20
Feb 20
Mar 20
Apr 20
May 20
Jun 20
Jul 20
Aug 20
Sep 20
Oct 20
Nov 20
Dec 20
Jan 21
Feb 21
Mar 21
Apr 21
May 21

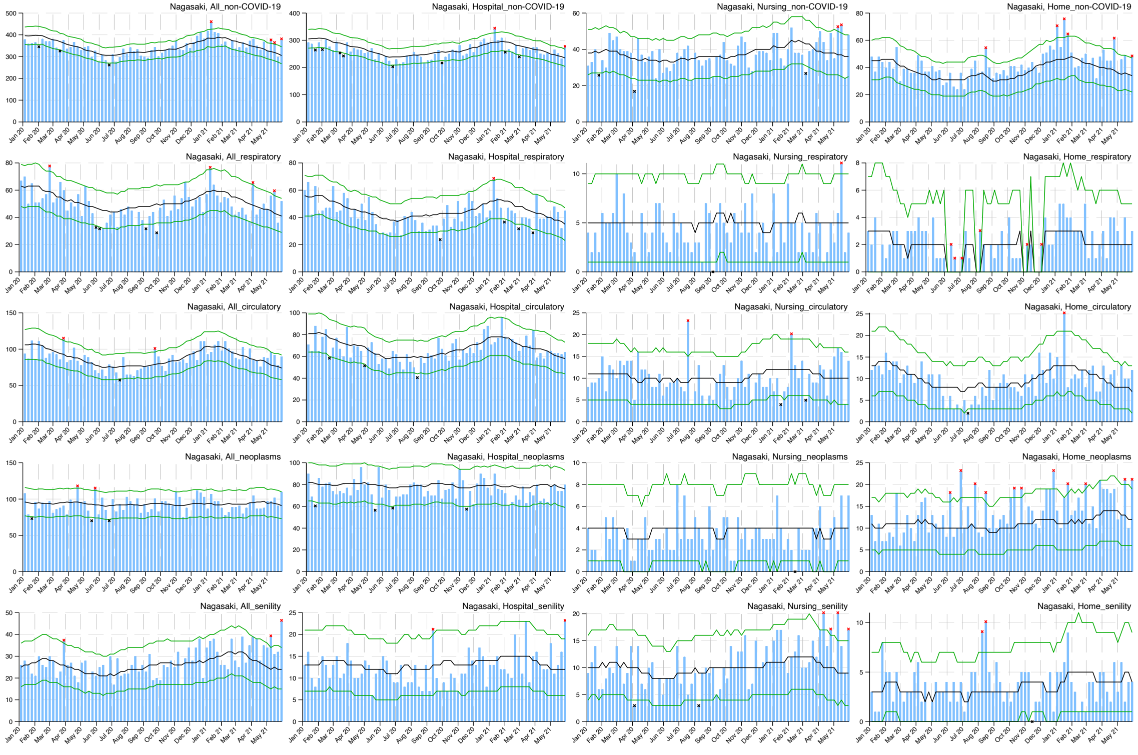
Nagasaki, All_non-COVID-19
Nagasaki, Hospital_non-COVID-19
Nagasaki, Nursing_non-COVID-19
Nagasaki, Home_non-COVID-19
Nagasaki, All_respiratory
Nagasaki, Hospital_respiratory
Nagasaki, Nursing_respiratory
Nagasaki, Home_respiratory
Nagasaki, All_circulatory
Nagasaki, Hospital_circulatory
Nagasaki, Nursing_circulatory
Nagasaki, Home_circulatory
Nagasaki, All_neoplasms
Nagasaki, Hospital_neoplasms
Nagasaki, Nursing_neoplasms
Nagasaki, Home_neoplasms
Nagasaki, All_senility
Nagasaki, Hospital_senility
Nagasaki, Nursing_senility
Nagasaki, Home_senility
Jan 20
Feb 20
Mar 20
Apr 20
May 20
Jun 20
Jul 20
Aug 20
Sep 20
Oct 20
Nov 20
Dec 20
Jan 21
Feb 21
Mar 21
Apr 21
May 21

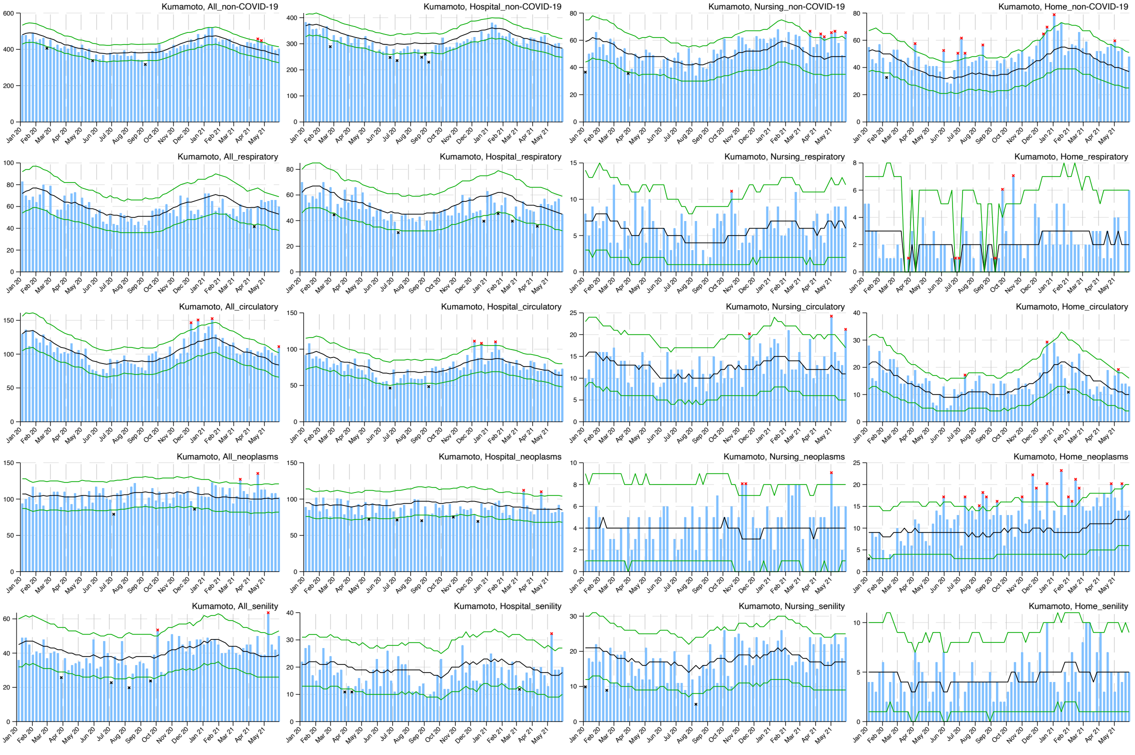
Kumamoto, All_non-COVID-19
Kumamoto, Hospital_non-COVID-19
Kumamoto, Nursing_non-COVID-19
Kumamoto, Home_non-COVID-19
Kumamoto, All_respiratory
Kumamoto, Hospital_respiratory
Kumamoto, Nursing_respiratory
Kumamoto, Home_respiratory
Kumamoto, All_circulatory
Kumamoto, Hospital_circulatory
Kumamoto, Nursing_circulatory
Kumamoto, Home_circulatory
Kumamoto, All_neoplasms
Kumamoto, Hospital_neoplasms
Kumamoto, Nursing_neoplasms
Kumamoto, Home_neoplasms
Kumamoto, All_senility
Kumamoto, Hospital_senility
Kumamoto, Nursing_senility
Kumamoto, Home_senility
Jan 20
Feb 20
Mar 20
Apr 20
May 20
Jun 20
Jul 20
Aug 20
Sep 20
Oct 20
Nov 20
Dec 20
Jan 21
Feb 21
Mar 21
Apr 21
May 21

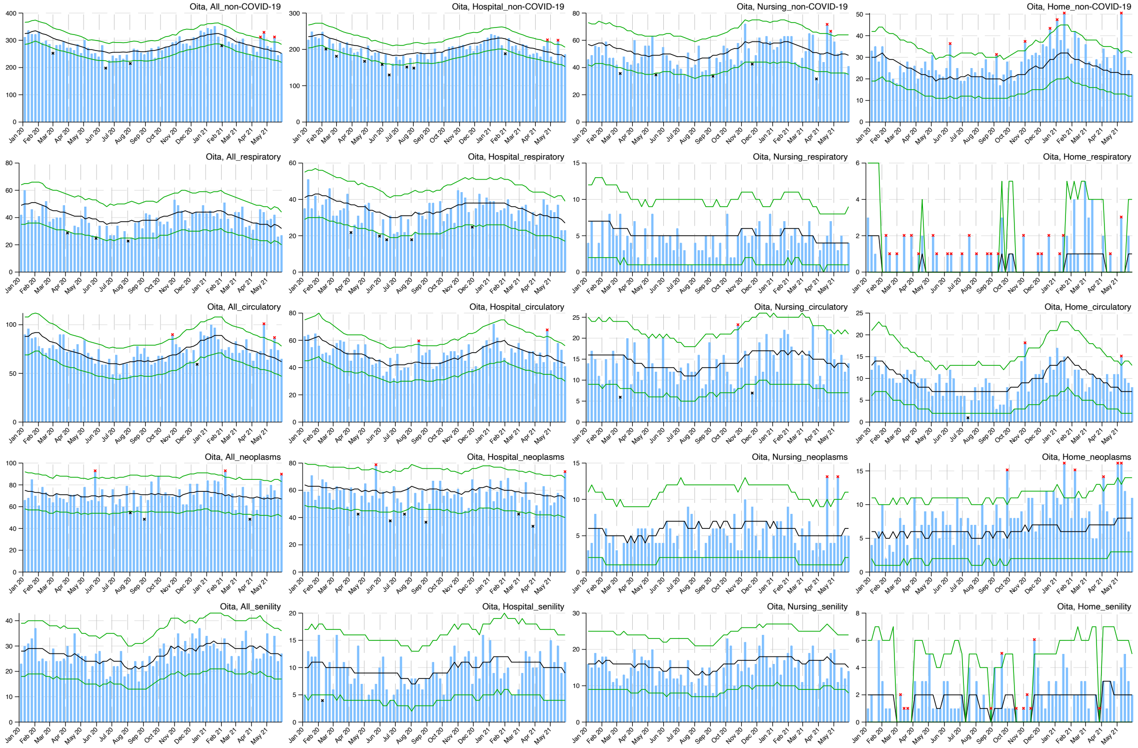
Oita, All_non-COVID-19
Oita, Hospital_non-COVID-19
Oita, Nursing_non-COVID-19
Oita, Home_non-COVID-19
Oita, All_respiratory
Oita, Hospital_respiratory
Oita, Nursing_respiratory
Oita, Home_respiratory
Oita, All_circulatory
Oita, Hospital_circulatory
Oita, Nursing_circulatory
Oita, Home_circulatory
Oita, All_neoplasms
Oita, Hospital_neoplasms
Oita, Nursing_neoplasms
Oita, Home_neoplasms
Oita, All_senility
Oita, Hospital_senility
Oita, Nursing_senility
Oita, Home_senility

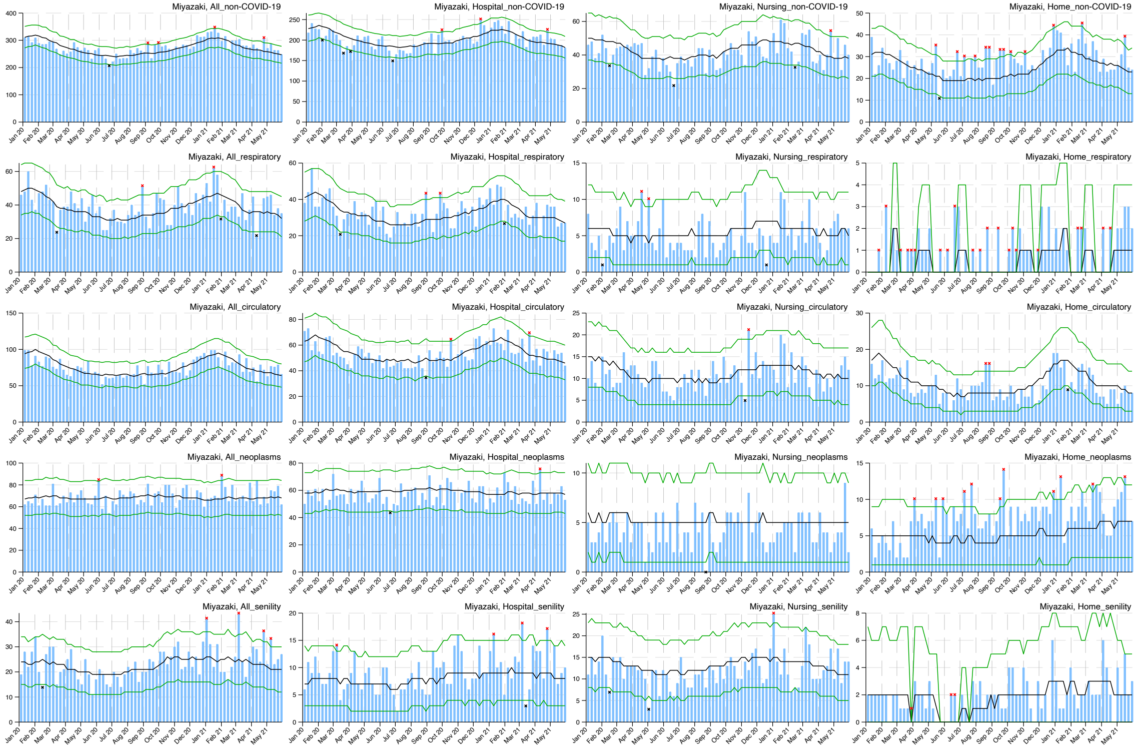
Miyazaki, All_non-COVID-19
Miyazaki, Hospital_non-COVID-19
Miyazaki, Nursing_non-COVID-19
Miyazaki, Home_non-COVID-19
Miyazaki, All_respiratory
Miyazaki, Hospital_respiratory
Miyazaki, Nursing_respiratory
Miyazaki, Home_respiratory
Miyazaki, All_circulatory
Miyazaki, Hospital_circulatory
Miyazaki, Nursing_circulatory
Miyazaki, Home_circulatory
Miyazaki, All_neoplasms
Miyazaki, Hospital_neoplasms
Miyazaki, Nursing_neoplasms
Miyazaki, Home_neoplasms
Miyazaki, All_senility
Miyazaki, Hospital_senility
Miyazaki, Nursing_senility
Miyazaki, Home_senility
Jan 20
Feb 20
Mar 20
Apr 20
May 20
Jun 20
Jul 20
Aug 20
Sep 20
Oct 20
Nov 20
Dec 20
Jan 21
Feb 21
Mar 21
Apr 21
May 21

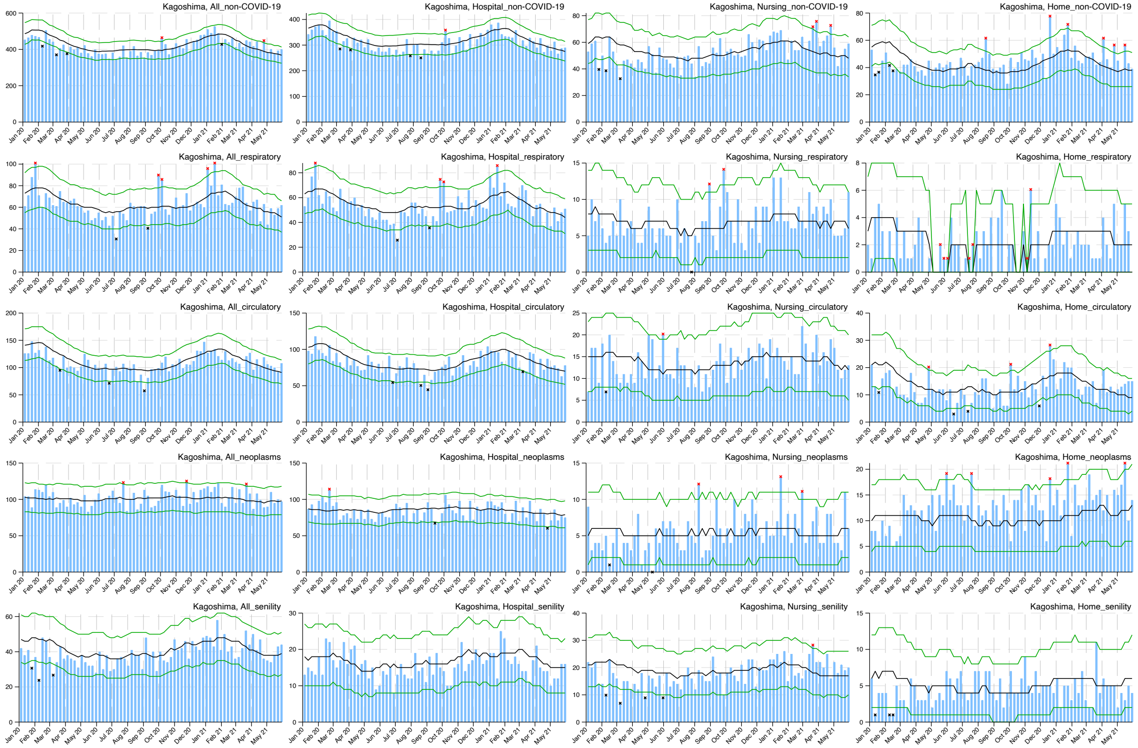
Kagoshima, All_non-COVID-19
Kagoshima, Hospital_non-COVID-19
Kagoshima, Nursing_non-COVID-19
Kagoshima, Home_non-COVID-19
Kagoshima, All_respiratory
Kagoshima, Hospital_respiratory
Kagoshima, Nursing_respiratory
Kagoshima, Home_respiratory
Kagoshima, All_circulatory
Kagoshima, Hospital_circulatory
Kagoshima, Nursing_circulatory
Kagoshima, Home_circulatory
Kagoshima, All_neoplasms
Kagoshima, Hospital_neoplasms
Kagoshima, Nursing_neoplasms
Kagoshima, Home_neoplasms
Kagoshima, All_senility
Kagoshima, Hospital_senility
Kagoshima, Nursing_senility
Kagoshima, Home_senility
Jan 20
Feb 20
Mar 20
Apr 20
May 20
Jun 20
Jul 20
Aug 20
Sep 20
Oct 20
Nov 20
Dec 20
Jan 21
Feb 21
Mar 21
Apr 21
May 21

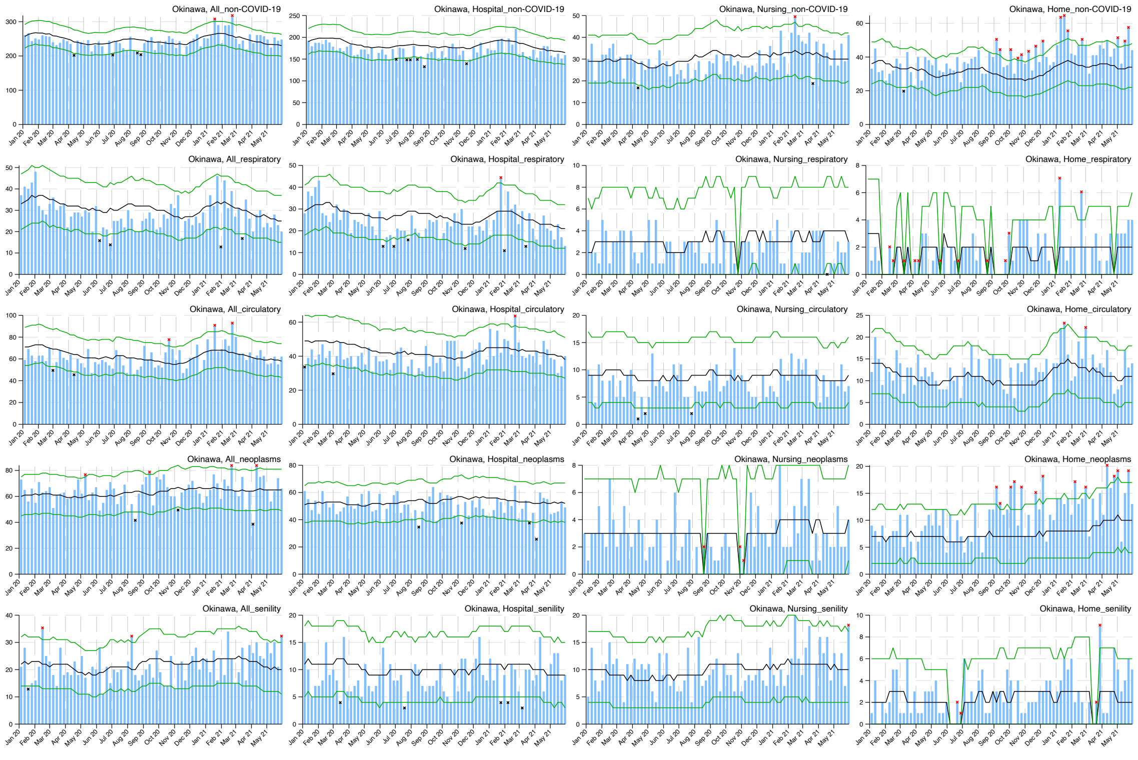
Okinawa, All_non-COVID-19
Okinawa, Hospital_non-COVID-19
Okinawa, Nursing_non-COVID-19
Okinawa, Home_non-COVID-19
Okinawa, All_respiratory
Okinawa, Hospital_respiratory
Okinawa, Nursing_respiratory
Okinawa, Home_respiratory
Okinawa, All_circulatory
Okinawa, Hospital_circulatory
Okinawa, Nursing_circulatory
Okinawa, Home_circulatory
Okinawa, All_neoplasms
Okinawa, Hospital_neoplasms
Okinawa, Nursing_neoplasms
Okinawa, Home_neoplasms
Okinawa, All_senility
Okinawa, Hospital_senility
Okinawa, Nursing_senility
Okinawa, Home_senility

Table A.1: Weekly number of observed and excess/exiguous deaths in Japan and 47 prefectures for non-COVID-19-related deaths in all places from January 2020 through May 2021.

Prefecture	Week	Observed	Excess deaths	Percent excess	Exiguous deaths	Percent exiguous
Japan						
	Apr 5 to Apr 11, 2021	26703	0–63	0.00–0.24	0–0	0.00–0.00
	Apr 12 to Apr 18, 2021	27626	0–1317	0.00–5.01	0–0	0.00–0.00
	Apr 19 to Apr 25, 2021	27710	155–1759	0.60–6.78	0–0	0.00–0.00
	Apr 26 to May 2, 2021	27710	690–2166	2.70–8.48	0–0	0.00–0.00
	May 3 to May 9, 2021	27353	689–2161	2.73–8.58	0–0	0.00–0.00
	May 10 to May 16, 2021	26698	461–1939	1.86–7.83	0–0	0.00–0.00
	May 17 to May 23, 2021	25443	0–997	0.00–4.08	0–0	0.00–0.00
	May 24 to May 30, 2021	25201	0–985	0.00–4.07	0–0	0.00–0.00
Hokkaido						
	Apr 5 to Apr 11, 2021	1257	0–8	0.00–0.64	0–0	0.00–0.00
	Apr 12 to Apr 18, 2021	1256	0–24	0.00–1.95	0–0	0.00–0.00
	Apr 19 to Apr 25, 2021	1274	0–46	0.00–3.75	0–0	0.00–0.00
	Apr 26 to May 2, 2021	1310	5–98	0.41–8.09	0–0	0.00–0.00
	May 3 to May 9, 2021	1300	3–95	0.25–7.88	0–0	0.00–0.00
	May 10 to May 16, 2021	1279	0–81	0.00–6.76	0–0	0.00–0.00
	May 17 to May 23, 2021	1322	32–126	2.68–10.54	0–0	0.00–0.00
	May 24 to May 30, 2021	1268	0–82	0.00–6.91	0–0	0.00–0.00
Aomori						
	Apr 5 to Apr 11, 2021	369	0–14	0.00–3.94	0–0	0.00–0.00
	Apr 12 to Apr 18, 2021	360	0–6	0.00–1.69	0–0	0.00–0.00
	Apr 19 to Apr 25, 2021	352	0–2	0.00–0.57	0–0	0.00–0.00
	Apr 26 to May 2, 2021	368	0–22	0.00–6.36	0–0	0.00–0.00
	May 3 to May 9, 2021	354	0–12	0.00–3.51	0–0	0.00–0.00
	May 10 to May 16, 2021	371	0–37	0.00–11.08	0–0	0.00–0.00
	May 17 to May 23, 2021	362	0–29	0.00–8.71	0–0	0.00–0.00
	May 24 to May 30, 2021	325	0–0	0.00–0.00	0–5	0.00–1.52
Iwate						
	Apr 5 to Apr 11, 2021	336	0–0	0.00–0.00	0–4	0.00–1.18
	Apr 12 to Apr 18, 2021	324	0–0	0.00–0.00	0–12	0.00–3.57
	Apr 19 to Apr 25, 2021	309	0–0	0.00–0.00	0–26	0.00–7.76
	Apr 26 to May 2, 2021	344	0–16	0.00–4.88	0–0	0.00–0.00
	May 3 to May 9, 2021	344	0–22	0.00–6.83	0–0	0.00–0.00
	May 10 to May 16, 2021	305	0–0	0.00–0.00	0–10	0.00–3.17
	May 17 to May 23, 2021	317	0–4	0.00–1.28	0–0	0.00–0.00
	May 24 to May 30, 2021	291	0–0	0.00–0.00	0–15	0.00–4.90
Miyagi						
	Apr 5 to Apr 11, 2021	499	0–17	0.00–3.53	0–0	0.00–0.00
	Apr 12 to Apr 18, 2021	551	22–72	4.59–15.03	0–0	0.00–0.00
	Apr 19 to Apr 25, 2021	469	0–0	0.00–0.00	0–3	0.00–0.64
	Apr 26 to May 2, 2021	492	0–21	0.00–4.46	0–0	0.00–0.00
	May 3 to May 9, 2021	489	0–25	0.00–5.39	0–0	0.00–0.00

	May 10 to May 16, 2021	436	0–0	0.00–0.00	0–21	0.00–4.60
	May 17 to May 23, 2021	434	0–0	0.00–0.00	0–15	0.00–3.34
	May 24 to May 30, 2021	457	0–17	0.00–3.86	0–0	0.00–0.00
Akita						
	Apr 5 to Apr 11, 2021	309	0–6	0.00–1.98	0–0	0.00–0.00
	Apr 12 to Apr 18, 2021	325	0–21	0.00–6.91	0–0	0.00–0.00
	Apr 19 to Apr 25, 2021	288	0–0	0.00–0.00	0–14	0.00–4.64
	Apr 26 to May 2, 2021	335	5–41	1.70–13.95	0–0	0.00–0.00
	May 3 to May 9, 2021	338	14–49	4.84–16.96	0–0	0.00–0.00
	May 10 to May 16, 2021	286	0–2	0.00–0.70	0–0	0.00–0.00
	May 17 to May 23, 2021	306	0–26	0.00–9.29	0–0	0.00–0.00
	May 24 to May 30, 2021	272	0–0	0.00–0.00	0–4	0.00–1.45
Yamagata						
	Apr 5 to Apr 11, 2021	257	0–0	0.00–0.00	6–42	2.01–14.05
	Apr 12 to Apr 18, 2021	302	0–6	0.00–2.03	0–0	0.00–0.00
	Apr 19 to Apr 25, 2021	312	0–17	0.00–5.76	0–0	0.00–0.00
	Apr 26 to May 2, 2021	273	0–0	0.00–0.00	0–17	0.00–5.86
	May 3 to May 9, 2021	305	0–22	0.00–7.77	0–0	0.00–0.00
	May 10 to May 16, 2021	323	6–42	2.14–14.95	0–0	0.00–0.00
	May 17 to May 23, 2021	314	0–36	0.00–12.95	0–0	0.00–0.00
	May 24 to May 30, 2021	298	0–24	0.00–8.76	0–0	0.00–0.00
Fukushima						
	Apr 5 to Apr 11, 2021	463	0–0	0.00–0.00	0–9	0.00–1.91
	Apr 12 to Apr 18, 2021	492	0–24	0.00–5.13	0–0	0.00–0.00
	Apr 19 to Apr 25, 2021	468	0–8	0.00–1.74	0–0	0.00–0.00
	Apr 26 to May 2, 2021	491	0–40	0.00–8.87	0–0	0.00–0.00
	May 3 to May 9, 2021	478	0–33	0.00–7.42	0–0	0.00–0.00
	May 10 to May 16, 2021	472	0–32	0.00–7.27	0–0	0.00–0.00
	May 17 to May 23, 2021	451	0–17	0.00–3.92	0–0	0.00–0.00
	May 24 to May 30, 2021	431	0–0	0.00–0.00	0–0	0.00–0.00
Ibaraki						
	Apr 5 to Apr 11, 2021	607	0–0	0.00–0.00	0–39	0.00–6.04
	Apr 12 to Apr 18, 2021	679	0–46	0.00–7.27	0–0	0.00–0.00
	Apr 19 to Apr 25, 2021	613	0–0	0.00–0.00	0–14	0.00–2.23
	Apr 26 to May 2, 2021	642	0–27	0.00–4.39	0–0	0.00–0.00
	May 3 to May 9, 2021	632	0–28	0.00–4.64	0–0	0.00–0.00
	May 10 to May 16, 2021	650	0–54	0.00–9.06	0–0	0.00–0.00
	May 17 to May 23, 2021	519	0–0	0.00–0.00	0–59	0.00–10.21
	May 24 to May 30, 2021	592	0–15	0.00–2.60	0–0	0.00–0.00
Tochigi						
	Apr 5 to Apr 11, 2021	406	0–0	0.00–0.00	0–22	0.00–5.14
	Apr 12 to Apr 18, 2021	456	0–33	0.00–7.80	0–0	0.00–0.00
	Apr 19 to Apr 25, 2021	436	0–22	0.00–5.31	0–0	0.00–0.00
	Apr 26 to May 2, 2021	450	2–50	0.50–12.50	0–0	0.00–0.00
	May 3 to May 9, 2021	440	0–44	0.00–11.11	0–0	0.00–0.00
	May 10 to May 16, 2021	397	0–14	0.00–3.66	0–0	0.00–0.00

	May 17 to May 23, 2021	395	0–21	0.00–5.61	0–0	0.00–0.00
	May 24 to May 30, 2021	416	0–44	0.00–11.83	0–0	0.00–0.00
Gunma						
	Apr 5 to Apr 11, 2021	447	0–0	0.00–0.00	0–2	0.00–0.45
	Apr 12 to Apr 18, 2021	523	31–84	7.06–19.13	0–0	0.00–0.00
	Apr 19 to Apr 25, 2021	452	0–17	0.00–3.91	0–0	0.00–0.00
	Apr 26 to May 2, 2021	454	0–29	0.00–6.82	0–0	0.00–0.00
	May 3 to May 9, 2021	450	0–26	0.00–6.13	0–0	0.00–0.00
	May 10 to May 16, 2021	453	0–39	0.00–9.42	0–0	0.00–0.00
	May 17 to May 23, 2021	385	0–0	0.00–0.00	0–24	0.00–5.87
	May 24 to May 30, 2021	443	0–35	0.00–8.58	0–0	0.00–0.00
Saitama						
	Apr 5 to Apr 11, 2021	1358	0–0	0.00–0.00	0–10	0.00–0.73
	Apr 12 to Apr 18, 2021	1408	0–60	0.00–4.45	0–0	0.00–0.00
	Apr 19 to Apr 25, 2021	1401	0–73	0.00–5.50	0–0	0.00–0.00
	Apr 26 to May 2, 2021	1508	84–198	6.41–15.11	0–0	0.00–0.00
	May 3 to May 9, 2021	1435	39–146	3.03–11.33	0–0	0.00–0.00
	May 10 to May 16, 2021	1371	0–104	0.00–8.21	0–0	0.00–0.00
	May 17 to May 23, 2021	1319	0–71	0.00–5.69	0–0	0.00–0.00
	May 24 to May 30, 2021	1293	0–57	0.00–4.61	0–0	0.00–0.00
Chiba						
	Apr 5 to Apr 11, 2021	1278	0–76	0.00–6.32	0–0	0.00–0.00
	Apr 12 to Apr 18, 2021	1251	0–63	0.00–5.30	0–0	0.00–0.00
	Apr 19 to Apr 25, 2021	1198	0–23	0.00–1.96	0–0	0.00–0.00
	Apr 26 to May 2, 2021	1249	0–96	0.00–8.33	0–0	0.00–0.00
	May 3 to May 9, 2021	1230	5–100	0.44–8.85	0–0	0.00–0.00
	May 10 to May 16, 2021	1155	0–43	0.00–3.87	0–0	0.00–0.00
	May 17 to May 23, 2021	1083	0–0	0.00–0.00	0–18	0.00–1.63
	May 24 to May 30, 2021	1095	0–1	0.00–0.09	0–0	0.00–0.00
Tokyo						
	Apr 5 to Apr 11, 2021	2288	0–0	0.00–0.00	0–41	0.00–1.76
	Apr 12 to Apr 18, 2021	2463	1–166	0.04–7.23	0–0	0.00–0.00
	Apr 19 to Apr 25, 2021	2504	85–244	3.76–10.80	0–0	0.00–0.00
	Apr 26 to May 2, 2021	2459	88–246	3.98–11.12	0–0	0.00–0.00
	May 3 to May 9, 2021	2360	10–173	0.46–7.91	0–0	0.00–0.00
	May 10 to May 16, 2021	2227	0–58	0.00–2.67	0–0	0.00–0.00
	May 17 to May 23, 2021	2178	0–30	0.00–1.40	0–0	0.00–0.00
	May 24 to May 30, 2021	2162	0–37	0.00–1.74	0–0	0.00–0.00
Kanagawa						
	Apr 5 to Apr 11, 2021	1666	0–40	0.00–2.46	0–0	0.00–0.00
	Apr 12 to Apr 18, 2021	1702	0–85	0.00–5.26	0–0	0.00–0.00
	Apr 19 to Apr 25, 2021	1675	0–81	0.00–5.08	0–0	0.00–0.00
	Apr 26 to May 2, 2021	1755	65–182	4.13–11.57	0–0	0.00–0.00
	May 3 to May 9, 2021	1722	59–174	3.81–11.24	0–0	0.00–0.00
	May 10 to May 16, 2021	1647	0–114	0.00–7.44	0–0	0.00–0.00
	May 17 to May 23, 2021	1634	6–120	0.40–7.93	0–0	0.00–0.00

	May 24 to May 30, 2021	1576	0–80	0.00–5.35	0–0	0.00–0.00
Niigata						
	Apr 5 to Apr 11, 2021	581	0–0	0.00–0.00	0–6	0.00–1.02
	Apr 12 to Apr 18, 2021	619	0–34	0.00–5.81	0–0	0.00–0.00
	Apr 19 to Apr 25, 2021	579	0–0	0.00–0.00	0–2	0.00–0.34
	Apr 26 to May 2, 2021	591	0–19	0.00–3.32	0–0	0.00–0.00
	May 3 to May 9, 2021	574	0–11	0.00–1.95	0–0	0.00–0.00
	May 10 to May 16, 2021	573	0–20	0.00–3.62	0–0	0.00–0.00
	May 17 to May 23, 2021	542	0–0	0.00–0.00	0–6	0.00–1.09
	May 24 to May 30, 2021	560	0–19	0.00–3.51	0–0	0.00–0.00
Toyama						
	Apr 5 to Apr 11, 2021	284	1–36	0.40–14.52	0–0	0.00–0.00
	Apr 12 to Apr 18, 2021	277	0–29	0.00–11.69	0–0	0.00–0.00
	Apr 19 to Apr 25, 2021	274	0–31	0.00–12.76	0–0	0.00–0.00
	Apr 26 to May 2, 2021	271	0–30	0.00–12.45	0–0	0.00–0.00
	May 3 to May 9, 2021	273	0–34	0.00–14.23	0–0	0.00–0.00
	May 10 to May 16, 2021	238	0–5	0.00–2.15	0–0	0.00–0.00
	May 17 to May 23, 2021	203	0–0	0.00–0.00	0–29	0.00–12.50
	May 24 to May 30, 2021	248	0–18	0.00–7.83	0–0	0.00–0.00
Ishikawa						
	Apr 5 to Apr 11, 2021	268	0–22	0.00–8.94	0–0	0.00–0.00
	Apr 12 to Apr 18, 2021	285	9–40	3.67–16.33	0–0	0.00–0.00
	Apr 19 to Apr 25, 2021	257	0–14	0.00–5.76	0–0	0.00–0.00
	Apr 26 to May 2, 2021	254	0–16	0.00–6.72	0–0	0.00–0.00
	May 3 to May 9, 2021	280	13–45	5.53–19.15	0–0	0.00–0.00
	May 10 to May 16, 2021	261	0–28	0.00–12.02	0–0	0.00–0.00
	May 17 to May 23, 2021	242	0–13	0.00–5.68	0–0	0.00–0.00
	May 24 to May 30, 2021	220	0–0	0.00–0.00	0–7	0.00–3.08
Fukui						
	Apr 5 to Apr 11, 2021	193	0–14	0.00–7.82	0–0	0.00–0.00
	Apr 12 to Apr 18, 2021	194	0–15	0.00–8.38	0–0	0.00–0.00
	Apr 19 to Apr 25, 2021	189	0–12	0.00–6.78	0–0	0.00–0.00
	Apr 26 to May 2, 2021	204	3–33	1.75–19.30	0–0	0.00–0.00
	May 3 to May 9, 2021	217	21–50	12.57–29.94	0–0	0.00–0.00
	May 10 to May 16, 2021	181	0–16	0.00–9.70	0–0	0.00–0.00
	May 17 to May 23, 2021	193	0–29	0.00–17.68	0–0	0.00–0.00
	May 24 to May 30, 2021	172	0–11	0.00–6.83	0–0	0.00–0.00
Yamanashi						
	Apr 5 to Apr 11, 2021	176	0–0	0.00–0.00	0–17	0.00–8.81
	Apr 12 to Apr 18, 2021	194	0–4	0.00–2.11	0–0	0.00–0.00
	Apr 19 to Apr 25, 2021	205	0–20	0.00–10.81	0–0	0.00–0.00
	Apr 26 to May 2, 2021	207	0–24	0.00–13.11	0–0	0.00–0.00
	May 3 to May 9, 2021	209	1–30	0.56–16.76	0–0	0.00–0.00
	May 10 to May 16, 2021	193	0–16	0.00–9.04	0–0	0.00–0.00
	May 17 to May 23, 2021	213	5–36	2.82–20.34	0–0	0.00–0.00
	May 24 to May 30, 2021	179	0–7	0.00–4.07	0–0	0.00–0.00

Nagano						
	Apr 5 to Apr 11, 2021	495	0–0	0.00–0.00	0–5	0.00–1.00
	Apr 12 to Apr 18, 2021	508	0–15	0.00–3.04	0–0	0.00–0.00
	Apr 19 to Apr 25, 2021	490	0–3	0.00–0.62	0–0	0.00–0.00
	Apr 26 to May 2, 2021	525	0–44	0.00–9.15	0–0	0.00–0.00
	May 3 to May 9, 2021	459	0–0	0.00–0.00	0–10	0.00–2.13
	May 10 to May 16, 2021	497	0–32	0.00–6.88	0–0	0.00–0.00
	May 17 to May 23, 2021	475	0–16	0.00–3.49	0–0	0.00–0.00
	May 24 to May 30, 2021	415	0–0	0.00–0.00	0–35	0.00–7.78
Gifu						
	Apr 5 to Apr 11, 2021	438	0–0	0.00–0.00	0–12	0.00–2.67
	Apr 12 to Apr 18, 2021	449	0–14	0.00–3.22	0–0	0.00–0.00
	Apr 19 to Apr 25, 2021	466	0–37	0.00–8.62	0–0	0.00–0.00
	Apr 26 to May 2, 2021	435	0–17	0.00–4.07	0–0	0.00–0.00
	May 3 to May 9, 2021	451	0–41	0.00–10.00	0–0	0.00–0.00
	May 10 to May 16, 2021	457	2–51	0.49–12.56	0–0	0.00–0.00
	May 17 to May 23, 2021	465	15–65	3.75–16.25	0–0	0.00–0.00
	May 24 to May 30, 2021	384	0–0	0.00–0.00	0–13	0.00–3.27
Shizuoka						
	Apr 5 to Apr 11, 2021	860	0–37	0.00–4.50	0–0	0.00–0.00
	Apr 12 to Apr 18, 2021	815	0–6	0.00–0.74	0–0	0.00–0.00
	Apr 19 to Apr 25, 2021	815	0–19	0.00–2.39	0–0	0.00–0.00
	Apr 26 to May 2, 2021	858	0–76	0.00–9.72	0–0	0.00–0.00
	May 3 to May 9, 2021	813	0–45	0.00–5.86	0–0	0.00–0.00
	May 10 to May 16, 2021	798	0–41	0.00–5.42	0–0	0.00–0.00
	May 17 to May 23, 2021	815	0–74	0.00–9.99	0–0	0.00–0.00
	May 24 to May 30, 2021	734	0–0	0.00–0.00	0–4	0.00–0.54
Aichi						
	Apr 5 to Apr 11, 2021	1278	0–0	0.00–0.00	0–97	0.00–7.05
	Apr 12 to Apr 18, 2021	1368	0–11	0.00–0.81	0–0	0.00–0.00
	Apr 19 to Apr 25, 2021	1389	0–50	0.00–3.73	0–0	0.00–0.00
	Apr 26 to May 2, 2021	1405	0–83	0.00–6.28	0–0	0.00–0.00
	May 3 to May 9, 2021	1344	0–41	0.00–3.15	0–0	0.00–0.00
	May 10 to May 16, 2021	1422	40–152	3.15–11.97	0–0	0.00–0.00
	May 17 to May 23, 2021	1206	0–0	0.00–0.00	0–45	0.00–3.60
	May 24 to May 30, 2021	1274	0–39	0.00–3.16	0–0	0.00–0.00
Mie						
	Apr 5 to Apr 11, 2021	401	0–0	0.00–0.00	0–6	0.00–1.47
	Apr 12 to Apr 18, 2021	418	0–19	0.00–4.76	0–0	0.00–0.00
	Apr 19 to Apr 25, 2021	409	0–21	0.00–5.41	0–0	0.00–0.00
	Apr 26 to May 2, 2021	429	5–49	1.32–12.89	0–0	0.00–0.00
	May 3 to May 9, 2021	400	0–26	0.00–6.95	0–0	0.00–0.00
	May 10 to May 16, 2021	410	0–40	0.00–10.81	0–0	0.00–0.00
	May 17 to May 23, 2021	417	10–55	2.76–15.19	0–0	0.00–0.00
	May 24 to May 30, 2021	356	0–0	0.00–0.00	0–4	0.00–1.11
Shiga						

	Apr 5 to Apr 11, 2021	289	0–34	0.00–13.33	0–0	0.00–0.00
	Apr 12 to Apr 18, 2021	285	0–32	0.00–12.65	0–0	0.00–0.00
	Apr 19 to Apr 25, 2021	250	0–2	0.00–0.81	0–0	0.00–0.00
	Apr 26 to May 2, 2021	286	7–42	2.87–17.21	0–0	0.00–0.00
	May 3 to May 9, 2021	273	0–33	0.00–13.75	0–0	0.00–0.00
	May 10 to May 16, 2021	256	0–23	0.00–9.87	0–0	0.00–0.00
	May 17 to May 23, 2021	252	0–22	0.00–9.57	0–0	0.00–0.00
	May 24 to May 30, 2021	261	0–34	0.00–14.98	0–0	0.00–0.00
Kyoto						
	Apr 5 to Apr 11, 2021	531	0–1	0.00–0.19	0–0	0.00–0.00
	Apr 12 to Apr 18, 2021	536	0–13	0.00–2.49	0–0	0.00–0.00
	Apr 19 to Apr 25, 2021	556	0–41	0.00–7.96	0–0	0.00–0.00
	Apr 26 to May 2, 2021	552	0–43	0.00–8.45	0–0	0.00–0.00
	May 3 to May 9, 2021	559	1–55	0.20–10.91	0–0	0.00–0.00
	May 10 to May 16, 2021	527	0–35	0.00–7.11	0–0	0.00–0.00
	May 17 to May 23, 2021	479	0–0	0.00–0.00	0–5	0.00–1.03
	May 24 to May 30, 2021	508	0–31	0.00–6.50	0–0	0.00–0.00
Osaka						
	Apr 5 to Apr 11, 2021	1830	0–35	0.00–1.95	0–0	0.00–0.00
	Apr 12 to Apr 18, 2021	1792	0–13	0.00–0.73	0–0	0.00–0.00
	Apr 19 to Apr 25, 2021	1946	55–194	3.14–11.07	0–0	0.00–0.00
	Apr 26 to May 2, 2021	1853	0–131	0.00–7.61	0–0	0.00–0.00
	May 3 to May 9, 2021	1910	80–208	4.70–12.22	0–0	0.00–0.00
	May 10 to May 16, 2021	1838	23–155	1.37–9.21	0–0	0.00–0.00
	May 17 to May 23, 2021	1735	0–63	0.00–3.77	0–0	0.00–0.00
	May 24 to May 30, 2021	1707	0–58	0.00–3.52	0–0	0.00–0.00
Hyogo						
	Apr 5 to Apr 11, 2021	1145	0–11	0.00–0.97	0–0	0.00–0.00
	Apr 12 to Apr 18, 2021	1173	0–52	0.00–4.64	0–0	0.00–0.00
	Apr 19 to Apr 25, 2021	1185	0–86	0.00–7.83	0–0	0.00–0.00
	Apr 26 to May 2, 2021	1226	52–150	4.83–13.94	0–0	0.00–0.00
	May 3 to May 9, 2021	1186	26–123	2.45–11.57	0–0	0.00–0.00
	May 10 to May 16, 2021	1157	12–104	1.14–9.88	0–0	0.00–0.00
	May 17 to May 23, 2021	1144	5–100	0.48–9.58	0–0	0.00–0.00
	May 24 to May 30, 2021	1095	0–66	0.00–6.41	0–0	0.00–0.00
Nara						
	Apr 5 to Apr 11, 2021	277	0–0	0.00–0.00	0–9	0.00–3.15
	Apr 12 to Apr 18, 2021	306	0–22	0.00–7.75	0–0	0.00–0.00
	Apr 19 to Apr 25, 2021	316	0–35	0.00–12.46	0–0	0.00–0.00
	Apr 26 to May 2, 2021	291	0–14	0.00–5.05	0–0	0.00–0.00
	May 3 to May 9, 2021	263	0–0	0.00–0.00	0–9	0.00–3.31
	May 10 to May 16, 2021	298	0–29	0.00–10.78	0–0	0.00–0.00
	May 17 to May 23, 2021	267	0–2	0.00–0.75	0–0	0.00–0.00
	May 24 to May 30, 2021	271	0–9	0.00–3.44	0–0	0.00–0.00
Wakayama						
	Apr 5 to Apr 11, 2021	241	0–0	0.00–0.00	0–4	0.00–1.63

	Apr 12 to Apr 18, 2021	254	0–11	0.00–4.53	0–0	0.00–0.00
	Apr 19 to Apr 25, 2021	263	0–25	0.00–10.50	0–0	0.00–0.00
	Apr 26 to May 2, 2021	239	0–4	0.00–1.70	0–0	0.00–0.00
	May 3 to May 9, 2021	250	0–22	0.00–9.65	0–0	0.00–0.00
	May 10 to May 16, 2021	230	0–9	0.00–4.07	0–0	0.00–0.00
	May 17 to May 23, 2021	210	0–0	0.00–0.00	0–8	0.00–3.67
	May 24 to May 30, 2021	219	0–3	0.00–1.39	0–0	0.00–0.00
Tottori						
	Apr 5 to Apr 11, 2021	144	0–4	0.00–2.86	0–0	0.00–0.00
	Apr 12 to Apr 18, 2021	158	0–20	0.00–14.49	0–0	0.00–0.00
	Apr 19 to Apr 25, 2021	147	0–12	0.00–8.89	0–0	0.00–0.00
	Apr 26 to May 2, 2021	132	0–0	0.00–0.00	0–0	0.00–0.00
	May 3 to May 9, 2021	156	1–24	0.76–18.18	0–0	0.00–0.00
	May 10 to May 16, 2021	162	9–32	6.92–24.62	0–0	0.00–0.00
	May 17 to May 23, 2021	137	0–9	0.00–7.03	0–0	0.00–0.00
	May 24 to May 30, 2021	137	0–12	0.00–9.60	0–0	0.00–0.00
Shimane						
	Apr 5 to Apr 11, 2021	160	0–0	0.00–0.00	3–30	1.58–15.79
	Apr 12 to Apr 18, 2021	220	5–34	2.69–18.28	0–0	0.00–0.00
	Apr 19 to Apr 25, 2021	189	0–4	0.00–2.16	0–0	0.00–0.00
	Apr 26 to May 2, 2021	194	0–14	0.00–7.78	0–0	0.00–0.00
	May 3 to May 9, 2021	202	0–27	0.00–15.43	0–0	0.00–0.00
	May 10 to May 16, 2021	183	0–12	0.00–7.02	0–0	0.00–0.00
	May 17 to May 23, 2021	186	0–19	0.00–11.38	0–0	0.00–0.00
	May 24 to May 30, 2021	178	0–13	0.00–7.88	0–0	0.00–0.00
Okayama						
	Apr 5 to Apr 11, 2021	421	0–2	0.00–0.48	0–0	0.00–0.00
	Apr 12 to Apr 18, 2021	421	0–10	0.00–2.43	0–0	0.00–0.00
	Apr 19 to Apr 25, 2021	441	0–33	0.00–8.09	0–0	0.00–0.00
	Apr 26 to May 2, 2021	426	0–22	0.00–5.45	0–0	0.00–0.00
	May 3 to May 9, 2021	409	0–10	0.00–2.51	0–0	0.00–0.00
	May 10 to May 16, 2021	454	11–58	2.78–14.65	0–0	0.00–0.00
	May 17 to May 23, 2021	390	0–0	0.00–0.00	0–0	0.00–0.00
	May 24 to May 30, 2021	412	0–29	0.00–7.57	0–0	0.00–0.00
Hiroshima						
	Apr 5 to Apr 11, 2021	617	0–26	0.00–4.40	0–0	0.00–0.00
	Apr 12 to Apr 18, 2021	599	0–15	0.00–2.57	0–0	0.00–0.00
	Apr 19 to Apr 25, 2021	635	0–63	0.00–11.01	0–0	0.00–0.00
	Apr 26 to May 2, 2021	614	0–49	0.00–8.67	0–0	0.00–0.00
	May 3 to May 9, 2021	636	20–82	3.61–14.80	0–0	0.00–0.00
	May 10 to May 16, 2021	611	1–62	0.18–11.29	0–0	0.00–0.00
	May 17 to May 23, 2021	584	0–46	0.00–8.55	0–0	0.00–0.00
	May 24 to May 30, 2021	541	0–12	0.00–2.27	0–0	0.00–0.00
Yamaguchi						
	Apr 5 to Apr 11, 2021	346	0–0	0.00–0.00	0–15	0.00–4.16
	Apr 12 to Apr 18, 2021	382	0–28	0.00–7.91	0–0	0.00–0.00

	Apr 19 to Apr 25, 2021	395	2–45	0.57–12.86	0–0	0.00–0.00
	Apr 26 to May 2, 2021	390	2–43	0.58–12.39	0–0	0.00–0.00
	May 3 to May 9, 2021	369	0–28	0.00–8.21	0–0	0.00–0.00
	May 10 to May 16, 2021	325	0–0	0.00–0.00	0–13	0.00–3.85
	May 17 to May 23, 2021	363	0–28	0.00–8.36	0–0	0.00–0.00
	May 24 to May 30, 2021	363	0–36	0.00–11.01	0–0	0.00–0.00
Tokushima						
	Apr 5 to Apr 11, 2021	206	0–15	0.00–7.85	0–0	0.00–0.00
	Apr 12 to Apr 18, 2021	188	0–0	0.00–0.00	0–1	0.00–0.53
	Apr 19 to Apr 25, 2021	242	28–58	15.22–31.52	0–0	0.00–0.00
	Apr 26 to May 2, 2021	190	0–11	0.00–6.15	0–0	0.00–0.00
	May 3 to May 9, 2021	209	3–32	1.69–18.08	0–0	0.00–0.00
	May 10 to May 16, 2021	215	8–37	4.49–20.79	0–0	0.00–0.00
	May 17 to May 23, 2021	173	0–1	0.00–0.58	0–0	0.00–0.00
	May 24 to May 30, 2021	176	0–6	0.00–3.53	0–0	0.00–0.00
Kagawa						
	Apr 5 to Apr 11, 2021	233	0–0	0.00–0.00	0–8	0.00–3.32
	Apr 12 to Apr 18, 2021	256	0–18	0.00–7.56	0–0	0.00–0.00
	Apr 19 to Apr 25, 2021	227	0–0	0.00–0.00	0–7	0.00–2.99
	Apr 26 to May 2, 2021	254	0–25	0.00–10.92	0–0	0.00–0.00
	May 3 to May 9, 2021	232	0–7	0.00–3.11	0–0	0.00–0.00
	May 10 to May 16, 2021	223	0–5	0.00–2.29	0–0	0.00–0.00
	May 17 to May 23, 2021	221	0–5	0.00–2.31	0–0	0.00–0.00
	May 24 to May 30, 2021	223	0–15	0.00–7.21	0–0	0.00–0.00
Ehime						
	Apr 5 to Apr 11, 2021	350	0–6	0.00–1.74	0–0	0.00–0.00
	Apr 12 to Apr 18, 2021	372	0–32	0.00–9.41	0–0	0.00–0.00
	Apr 19 to Apr 25, 2021	372	0–38	0.00–11.38	0–0	0.00–0.00
	Apr 26 to May 2, 2021	368	0–39	0.00–11.85	0–0	0.00–0.00
	May 3 to May 9, 2021	355	0–30	0.00–9.23	0–0	0.00–0.00
	May 10 to May 16, 2021	365	5–46	1.57–14.42	0–0	0.00–0.00
	May 17 to May 23, 2021	303	0–0	0.00–0.00	0–10	0.00–3.19
	May 24 to May 30, 2021	333	0–24	0.00–7.77	0–0	0.00–0.00
Kochi						
	Apr 5 to Apr 11, 2021	193	0–5	0.00–2.66	0–0	0.00–0.00
	Apr 12 to Apr 18, 2021	199	0–12	0.00–6.42	0–0	0.00–0.00
	Apr 19 to Apr 25, 2021	209	0–25	0.00–13.59	0–0	0.00–0.00
	Apr 26 to May 2, 2021	196	0–14	0.00–7.69	0–0	0.00–0.00
	May 3 to May 9, 2021	177	0–0	0.00–0.00	0–1	0.00–0.56
	May 10 to May 16, 2021	193	0–16	0.00–9.04	0–0	0.00–0.00
	May 17 to May 23, 2021	194	0–20	0.00–11.49	0–0	0.00–0.00
	May 24 to May 30, 2021	203	1–31	0.58–18.02	0–0	0.00–0.00
Fukuoka						
	Apr 5 to Apr 11, 2021	1001	0–0	0.00–0.00	0–44	0.00–4.21
	Apr 12 to Apr 18, 2021	1058	0–25	0.00–2.42	0–0	0.00–0.00
	Apr 19 to Apr 25, 2021	1161	54–142	5.30–13.94	0–0	0.00–0.00

	Apr 26 to May 2, 2021	1087	0–82	0.00–8.16	0–0	0.00–0.00
	May 3 to May 9, 2021	1068	3–82	0.30–8.32	0–0	0.00–0.00
	May 10 to May 16, 2021	1082	19–105	1.94–10.75	0–0	0.00–0.00
	May 17 to May 23, 2021	991	0–30	0.00–3.12	0–0	0.00–0.00
	May 24 to May 30, 2021	1010	0–58	0.00–6.09	0–0	0.00–0.00
Saga						
	Apr 5 to Apr 11, 2021	193	0–4	0.00–2.12	0–0	0.00–0.00
	Apr 12 to Apr 18, 2021	199	0–13	0.00–6.99	0–0	0.00–0.00
	Apr 19 to Apr 25, 2021	205	0–21	0.00–11.41	0–0	0.00–0.00
	Apr 26 to May 2, 2021	171	0–0	0.00–0.00	0–10	0.00–5.52
	May 3 to May 9, 2021	222	16–44	8.99–24.72	0–0	0.00–0.00
	May 10 to May 16, 2021	194	0–17	0.00–9.60	0–0	0.00–0.00
	May 17 to May 23, 2021	185	0–8	0.00–4.52	0–0	0.00–0.00
	May 24 to May 30, 2021	173	0–0	0.00–0.00	0–3	0.00–1.70
Nagasaki						
	Apr 5 to Apr 11, 2021	350	0–7	0.00–2.04	0–0	0.00–0.00
	Apr 12 to Apr 18, 2021	331	0–0	0.00–0.00	0–9	0.00–2.65
	Apr 19 to Apr 25, 2021	369	0–34	0.00–10.15	0–0	0.00–0.00
	Apr 26 to May 2, 2021	328	0–2	0.00–0.61	0–0	0.00–0.00
	May 3 to May 9, 2021	373	12–50	3.72–15.48	0–0	0.00–0.00
	May 10 to May 16, 2021	361	4–42	1.25–13.17	0–0	0.00–0.00
	May 17 to May 23, 2021	299	0–0	0.00–0.00	0–14	0.00–4.47
	May 24 to May 30, 2021	378	33–73	10.82–23.93	0–0	0.00–0.00
Kumamoto						
	Apr 5 to Apr 11, 2021	430	0–26	0.00–6.44	0–0	0.00–0.00
	Apr 12 to Apr 18, 2021	453	9–54	2.26–13.53	0–0	0.00–0.00
	Apr 19 to Apr 25, 2021	444	6–50	1.52–12.69	0–0	0.00–0.00
	Apr 26 to May 2, 2021	422	0–30	0.00–7.65	0–0	0.00–0.00
	May 3 to May 9, 2021	422	0–38	0.00–9.90	0–0	0.00–0.00
	May 10 to May 16, 2021	418	0–40	0.00–10.58	0–0	0.00–0.00
	May 17 to May 23, 2021	395	0–21	0.00–5.61	0–0	0.00–0.00
	May 24 to May 30, 2021	404	0–35	0.00–9.49	0–0	0.00–0.00
Oita						
	Apr 5 to Apr 11, 2021	257	0–0	0.00–0.00	0–18	0.00–6.55
	Apr 12 to Apr 18, 2021	309	1–37	0.37–13.60	0–0	0.00–0.00
	Apr 19 to Apr 25, 2021	326	24–58	8.96–21.64	0–0	0.00–0.00
	Apr 26 to May 2, 2021	279	0–12	0.00–4.49	0–0	0.00–0.00
	May 3 to May 9, 2021	272	0–11	0.00–4.21	0–0	0.00–0.00
	May 10 to May 16, 2021	309	15–49	5.77–18.85	0–0	0.00–0.00
	May 17 to May 23, 2021	245	0–0	0.00–0.00	0–13	0.00–5.04
	May 24 to May 30, 2021	252	0–0	0.00–0.00	0–0	0.00–0.00
Miyazaki						
	Apr 5 to Apr 11, 2021	271	0–6	0.00–2.26	0–0	0.00–0.00
	Apr 12 to Apr 18, 2021	262	0–0	0.00–0.00	0–2	0.00–0.76
	Apr 19 to Apr 25, 2021	307	16–48	6.18–18.53	0–0	0.00–0.00
	Apr 26 to May 2, 2021	271	0–13	0.00–5.04	0–0	0.00–0.00

	May 3 to May 9, 2021	288	0–32	0.00–12.50	0–0	0.00–0.00
	May 10 to May 16, 2021	264	0–12	0.00–4.76	0–0	0.00–0.00
	May 17 to May 23, 2021	263	0–13	0.00–5.20	0–0	0.00–0.00
	May 24 to May 30, 2021	248	0–1	0.00–0.40	0–0	0.00–0.00
Kagoshima						
	Apr 5 to Apr 11, 2021	443	0–41	0.00–10.20	0–0	0.00–0.00
	Apr 12 to Apr 18, 2021	393	0–0	0.00–0.00	0–3	0.00–0.76
	Apr 19 to Apr 25, 2021	444	8–55	2.06–14.14	0–0	0.00–0.00
	Apr 26 to May 2, 2021	384	0–1	0.00–0.26	0–0	0.00–0.00
	May 3 to May 9, 2021	401	0–20	0.00–5.25	0–0	0.00–0.00
	May 10 to May 16, 2021	377	0–0	0.00–0.00	0–0	0.00–0.00
	May 17 to May 23, 2021	393	0–19	0.00–5.08	0–0	0.00–0.00
	May 24 to May 30, 2021	400	0–32	0.00–8.70	0–0	0.00–0.00
Okinawa						
	Apr 5 to Apr 11, 2021	261	0–20	0.00–8.30	0–0	0.00–0.00
	Apr 12 to Apr 18, 2021	261	0–20	0.00–8.30	0–0	0.00–0.00
	Apr 19 to Apr 25, 2021	257	0–21	0.00–8.90	0–0	0.00–0.00
	Apr 26 to May 2, 2021	248	0–14	0.00–5.98	0–0	0.00–0.00
	May 3 to May 9, 2021	224	0–0	0.00–0.00	0–10	0.00–4.27
	May 10 to May 16, 2021	254	0–21	0.00–9.01	0–0	0.00–0.00
	May 17 to May 23, 2021	244	0–11	0.00–4.72	0–0	0.00–0.00
	May 24 to May 30, 2021	248	0–18	0.00–7.83	0–0	0.00–0.00

Table A.2: Weekly number of observed and excess/exiguous deaths in Japan and 47 prefectures for non-COVID-19-related deaths in hospitals and clinics from January 2020 through May 2021.

Prefecture	Week	Observed	Excess deaths	Percent excess	Exiguous deaths	Percent exiguous
Japan						
	Apr 5 to Apr 11, 2021	18186	0–0	0.00–0.00	0–481	0.00–2.58
	Apr 12 to Apr 18, 2021	18622	0–135	0.00–0.73	0–0	0.00–0.00
	Apr 19 to Apr 25, 2021	18851	0–620	0.00–3.40	0–0	0.00–0.00
	Apr 26 to May 2, 2021	18497	0–521	0.00–2.90	0–0	0.00–0.00
	May 3 to May 9, 2021	18452	0–884	0.00–5.03	0–0	0.00–0.00
	May 10 to May 16, 2021	17985	0–597	0.00–3.43	0–0	0.00–0.00
	May 17 to May 23, 2021	17130	0–41	0.00–0.24	0–0	0.00–0.00
	May 24 to May 30, 2021	17230	0–381	0.00–2.26	0–0	0.00–0.00
Hokkaido						
	Apr 5 to Apr 11, 2021	986	0–0	0.00–0.00	0–19	0.00–1.89
	Apr 12 to Apr 18, 2021	978	0–0	0.00–0.00	0–16	0.00–1.61
	Apr 19 to Apr 25, 2021	1032	0–44	0.00–4.45	0–0	0.00–0.00
	Apr 26 to May 2, 2021	1021	0–44	0.00–4.50	0–0	0.00–0.00
	May 3 to May 9, 2021	1015	0–45	0.00–4.64	0–0	0.00–0.00
	May 10 to May 16, 2021	1031	0–65	0.00–6.73	0–0	0.00–0.00
	May 17 to May 23, 2021	1068	27–106	2.81–11.02	0–0	0.00–0.00
	May 24 to May 30, 2021	997	0–44	0.00–4.62	0–0	0.00–0.00
Aomori						
	Apr 5 to Apr 11, 2021	262	0–10	0.00–3.97	0–0	0.00–0.00
	Apr 12 to Apr 18, 2021	255	0–3	0.00–1.19	0–0	0.00–0.00
	Apr 19 to Apr 25, 2021	246	0–0	0.00–0.00	0–4	0.00–1.60
	Apr 26 to May 2, 2021	255	0–6	0.00–2.41	0–0	0.00–0.00
	May 3 to May 9, 2021	252	0–8	0.00–3.28	0–0	0.00–0.00
	May 10 to May 16, 2021	268	0–30	0.00–12.61	0–0	0.00–0.00
	May 17 to May 23, 2021	242	0–5	0.00–2.11	0–0	0.00–0.00
	May 24 to May 30, 2021	220	0–0	0.00–0.00	0–15	0.00–6.38
Iwate						
	Apr 5 to Apr 11, 2021	248	0–0	0.00–0.00	0–0	0.00–0.00
	Apr 12 to Apr 18, 2021	227	0–0	0.00–0.00	0–20	0.00–8.10
	Apr 19 to Apr 25, 2021	232	0–0	0.00–0.00	0–16	0.00–6.45
	Apr 26 to May 2, 2021	245	0–4	0.00–1.66	0–0	0.00–0.00
	May 3 to May 9, 2021	246	0–9	0.00–3.80	0–0	0.00–0.00
	May 10 to May 16, 2021	213	0–0	0.00–0.00	0–19	0.00–8.19
	May 17 to May 23, 2021	231	0–0	0.00–0.00	0–0	0.00–0.00
	May 24 to May 30, 2021	220	0–0	0.00–0.00	0–5	0.00–2.22
Miyagi						
	Apr 5 to Apr 11, 2021	317	0–0	0.00–0.00	0–22	0.00–6.49
	Apr 12 to Apr 18, 2021	359	0–22	0.00–6.53	0–0	0.00–0.00
	Apr 19 to Apr 25, 2021	319	0–0	0.00–0.00	0–13	0.00–3.92
	Apr 26 to May 2, 2021	332	0–0	0.00–0.00	0–0	0.00–0.00
	May 3 to May 9, 2021	338	0–12	0.00–3.68	0–0	0.00–0.00

	May 10 to May 16, 2021	280	0–0	0.00–0.00	4–42	1.24–13.04
	May 17 to May 23, 2021	299	0–0	0.00–0.00	0–17	0.00–5.38
	May 24 to May 30, 2021	320	0–11	0.00–3.56	0–0	0.00–0.00
Akita						
	Apr 5 to Apr 11, 2021	233	0–7	0.00–3.10	0–0	0.00–0.00
	Apr 12 to Apr 18, 2021	227	0–0	0.00–0.00	0–0	0.00–0.00
	Apr 19 to Apr 25, 2021	222	0–0	0.00–0.00	0–4	0.00–1.77
	Apr 26 to May 2, 2021	259	7–37	3.15–16.67	0–0	0.00–0.00
	May 3 to May 9, 2021	238	0–18	0.00–8.18	0–0	0.00–0.00
	May 10 to May 16, 2021	209	0–0	0.00–0.00	0–6	0.00–2.79
	May 17 to May 23, 2021	236	0–24	0.00–11.32	0–0	0.00–0.00
	May 24 to May 30, 2021	204	0–0	0.00–0.00	0–5	0.00–2.39
Yamagata						
	Apr 5 to Apr 11, 2021	188	0–0	0.00–0.00	0–15	0.00–7.39
	Apr 12 to Apr 18, 2021	208	0–4	0.00–1.96	0–0	0.00–0.00
	Apr 19 to Apr 25, 2021	205	0–2	0.00–0.99	0–0	0.00–0.00
	Apr 26 to May 2, 2021	158	0–0	0.00–0.00	12–41	6.03–20.60
	May 3 to May 9, 2021	196	0–2	0.00–1.03	0–0	0.00–0.00
	May 10 to May 16, 2021	211	0–19	0.00–9.90	0–0	0.00–0.00
	May 17 to May 23, 2021	201	0–11	0.00–5.79	0–0	0.00–0.00
	May 24 to May 30, 2021	181	0–0	0.00–0.00	0–8	0.00–4.23
Fukushima						
	Apr 5 to Apr 11, 2021	322	0–0	0.00–0.00	0–14	0.00–4.17
	Apr 12 to Apr 18, 2021	322	0–0	0.00–0.00	0–12	0.00–3.59
	Apr 19 to Apr 25, 2021	333	0–3	0.00–0.91	0–0	0.00–0.00
	Apr 26 to May 2, 2021	331	0–9	0.00–2.80	0–0	0.00–0.00
	May 3 to May 9, 2021	337	0–19	0.00–5.97	0–0	0.00–0.00
	May 10 to May 16, 2021	326	0–14	0.00–4.49	0–0	0.00–0.00
	May 17 to May 23, 2021	317	0–10	0.00–3.26	0–0	0.00–0.00
	May 24 to May 30, 2021	305	0–0	0.00–0.00	0–0	0.00–0.00
Ibaraki						
	Apr 5 to Apr 11, 2021	444	0–0	0.00–0.00	0–43	0.00–8.83
	Apr 12 to Apr 18, 2021	484	0–8	0.00–1.68	0–0	0.00–0.00
	Apr 19 to Apr 25, 2021	457	0–0	0.00–0.00	0–12	0.00–2.56
	Apr 26 to May 2, 2021	478	0–16	0.00–3.46	0–0	0.00–0.00
	May 3 to May 9, 2021	476	0–19	0.00–4.16	0–0	0.00–0.00
	May 10 to May 16, 2021	464	0–11	0.00–2.43	0–0	0.00–0.00
	May 17 to May 23, 2021	377	0–0	0.00–0.00	18–67	4.05–15.09
	May 24 to May 30, 2021	423	0–0	0.00–0.00	0–17	0.00–3.86
Tochigi						
	Apr 5 to Apr 11, 2021	275	0–0	0.00–0.00	0–20	0.00–6.78
	Apr 12 to Apr 18, 2021	298	0–5	0.00–1.71	0–0	0.00–0.00
	Apr 19 to Apr 25, 2021	276	0–0	0.00–0.00	0–10	0.00–3.50
	Apr 26 to May 2, 2021	285	0–7	0.00–2.52	0–0	0.00–0.00
	May 3 to May 9, 2021	306	0–32	0.00–11.68	0–0	0.00–0.00
	May 10 to May 16, 2021	269	0–1	0.00–0.37	0–0	0.00–0.00

	May 17 to May 23, 2021	244	0–0	0.00–0.00	0–16	0.00–6.15
	May 24 to May 30, 2021	270	0–12	0.00–4.65	0–0	0.00–0.00
Gunma						
	Apr 5 to Apr 11, 2021	301	0–0	0.00–0.00	0–10	0.00–3.22
	Apr 12 to Apr 18, 2021	356	10–50	3.27–16.34	0–0	0.00–0.00
	Apr 19 to Apr 25, 2021	262	0–0	0.00–0.00	4–41	1.32–13.53
	Apr 26 to May 2, 2021	300	0–4	0.00–1.35	0–0	0.00–0.00
	May 3 to May 9, 2021	304	0–7	0.00–2.36	0–0	0.00–0.00
	May 10 to May 16, 2021	316	0–26	0.00–8.97	0–0	0.00–0.00
	May 17 to May 23, 2021	268	0–0	0.00–0.00	0–15	0.00–5.30
	May 24 to May 30, 2021	299	0–18	0.00–6.41	0–0	0.00–0.00
Saitama						
	Apr 5 to Apr 11, 2021	943	0–0	0.00–0.00	0–20	0.00–2.08
	Apr 12 to Apr 18, 2021	958	0–10	0.00–1.05	0–0	0.00–0.00
	Apr 19 to Apr 25, 2021	968	0–39	0.00–4.20	0–0	0.00–0.00
	Apr 26 to May 2, 2021	1008	3–88	0.33–9.57	0–0	0.00–0.00
	May 3 to May 9, 2021	989	4–83	0.44–9.16	0–0	0.00–0.00
	May 10 to May 16, 2021	940	0–50	0.00–5.62	0–0	0.00–0.00
	May 17 to May 23, 2021	894	0–18	0.00–2.05	0–0	0.00–0.00
	May 24 to May 30, 2021	904	0–41	0.00–4.75	0–0	0.00–0.00
Chiba						
	Apr 5 to Apr 11, 2021	905	0–56	0.00–6.60	0–0	0.00–0.00
	Apr 12 to Apr 18, 2021	852	0–17	0.00–2.04	0–0	0.00–0.00
	Apr 19 to Apr 25, 2021	787	0–0	0.00–0.00	0–36	0.00–4.37
	Apr 26 to May 2, 2021	874	0–67	0.00–8.30	0–0	0.00–0.00
	May 3 to May 9, 2021	833	0–37	0.00–4.65	0–0	0.00–0.00
	May 10 to May 16, 2021	780	0–0	0.00–0.00	0–4	0.00–0.51
	May 17 to May 23, 2021	742	0–0	0.00–0.00	0–30	0.00–3.89
	May 24 to May 30, 2021	766	0–0	0.00–0.00	0–3	0.00–0.39
Tokyo						
	Apr 5 to Apr 11, 2021	1455	0–0	0.00–0.00	0–45	0.00–3.00
	Apr 12 to Apr 18, 2021	1536	0–62	0.00–4.21	0–0	0.00–0.00
	Apr 19 to Apr 25, 2021	1583	8–131	0.55–9.02	0–0	0.00–0.00
	Apr 26 to May 2, 2021	1520	0–87	0.00–6.07	0–0	0.00–0.00
	May 3 to May 9, 2021	1453	0–44	0.00–3.12	0–0	0.00–0.00
	May 10 to May 16, 2021	1388	0–0	0.00–0.00	0–7	0.00–0.50
	May 17 to May 23, 2021	1323	0–0	0.00–0.00	0–51	0.00–3.71
	May 24 to May 30, 2021	1402	0–42	0.00–3.09	0–0	0.00–0.00
Kanagawa						
	Apr 5 to Apr 11, 2021	998	0–0	0.00–0.00	0–34	0.00–3.29
	Apr 12 to Apr 18, 2021	1054	0–33	0.00–3.23	0–0	0.00–0.00
	Apr 19 to Apr 25, 2021	1031	0–27	0.00–2.69	0–0	0.00–0.00
	Apr 26 to May 2, 2021	1002	0–14	0.00–1.42	0–0	0.00–0.00
	May 3 to May 9, 2021	1002	0–37	0.00–3.83	0–0	0.00–0.00
	May 10 to May 16, 2021	944	0–0	0.00–0.00	0–17	0.00–1.77
	May 17 to May 23, 2021	994	0–53	0.00–5.63	0–0	0.00–0.00

	May 24 to May 30, 2021	946	0–25	0.00–2.71	0–0	0.00–0.00
Niigata						
	Apr 5 to Apr 11, 2021	390	0–0	0.00–0.00	0–32	0.00–7.58
	Apr 12 to Apr 18, 2021	426	0–5	0.00–1.19	0–0	0.00–0.00
	Apr 19 to Apr 25, 2021	415	0–0	0.00–0.00	0–2	0.00–0.48
	Apr 26 to May 2, 2021	417	0–7	0.00–1.71	0–0	0.00–0.00
	May 3 to May 9, 2021	391	0–0	0.00–0.00	0–14	0.00–3.46
	May 10 to May 16, 2021	390	0–0	0.00–0.00	0–8	0.00–2.01
	May 17 to May 23, 2021	379	0–0	0.00–0.00	0–16	0.00–4.05
	May 24 to May 30, 2021	387	0–0	0.00–0.00	0–4	0.00–1.02
Toyama						
	Apr 5 to Apr 11, 2021	194	0–18	0.00–10.23	0–0	0.00–0.00
	Apr 12 to Apr 18, 2021	189	0–13	0.00–7.39	0–0	0.00–0.00
	Apr 19 to Apr 25, 2021	185	0–13	0.00–7.56	0–0	0.00–0.00
	Apr 26 to May 2, 2021	172	0–1	0.00–0.58	0–0	0.00–0.00
	May 3 to May 9, 2021	184	0–15	0.00–8.88	0–0	0.00–0.00
	May 10 to May 16, 2021	162	0–0	0.00–0.00	0–4	0.00–2.41
	May 17 to May 23, 2021	142	0–0	0.00–0.00	0–23	0.00–13.94
	May 24 to May 30, 2021	169	0–6	0.00–3.68	0–0	0.00–0.00
Ishikawa						
	Apr 5 to Apr 11, 2021	170	0–0	0.00–0.00	0–6	0.00–3.41
	Apr 12 to Apr 18, 2021	186	0–11	0.00–6.29	0–0	0.00–0.00
	Apr 19 to Apr 25, 2021	174	0–1	0.00–0.58	0–0	0.00–0.00
	Apr 26 to May 2, 2021	177	0–8	0.00–4.73	0–0	0.00–0.00
	May 3 to May 9, 2021	198	5–32	3.01–19.28	0–0	0.00–0.00
	May 10 to May 16, 2021	189	0–26	0.00–15.95	0–0	0.00–0.00
	May 17 to May 23, 2021	162	0–0	0.00–0.00	0–0	0.00–0.00
	May 24 to May 30, 2021	147	0–0	0.00–0.00	0–12	0.00–7.55
Fukui						
	Apr 5 to Apr 11, 2021	134	0–12	0.00–9.84	0–0	0.00–0.00
	Apr 12 to Apr 18, 2021	128	0–6	0.00–4.92	0–0	0.00–0.00
	Apr 19 to Apr 25, 2021	125	0–4	0.00–3.31	0–0	0.00–0.00
	Apr 26 to May 2, 2021	137	0–19	0.00–16.10	0–0	0.00–0.00
	May 3 to May 9, 2021	142	4–26	3.45–22.41	0–0	0.00–0.00
	May 10 to May 16, 2021	115	0–0	0.00–0.00	0–1	0.00–0.86
	May 17 to May 23, 2021	132	0–18	0.00–15.79	0–0	0.00–0.00
	May 24 to May 30, 2021	118	0–7	0.00–6.31	0–0	0.00–0.00
Yamanashi						
	Apr 5 to Apr 11, 2021	116	0–0	0.00–0.00	0–18	0.00–13.43
	Apr 12 to Apr 18, 2021	132	0–1	0.00–0.76	0–0	0.00–0.00
	Apr 19 to Apr 25, 2021	143	0–15	0.00–11.72	0–0	0.00–0.00
	Apr 26 to May 2, 2021	123	0–0	0.00–0.00	0–4	0.00–3.15
	May 3 to May 9, 2021	138	0–13	0.00–10.40	0–0	0.00–0.00
	May 10 to May 16, 2021	131	0–6	0.00–4.80	0–0	0.00–0.00
	May 17 to May 23, 2021	134	0–10	0.00–8.06	0–0	0.00–0.00
	May 24 to May 30, 2021	115	0–0	0.00–0.00	0–7	0.00–5.74

Nagano						
	Apr 5 to Apr 11, 2021	314	0–0	0.00–0.00	0–19	0.00–5.71
	Apr 12 to Apr 18, 2021	324	0–0	0.00–0.00	0–8	0.00–2.41
	Apr 19 to Apr 25, 2021	311	0–0	0.00–0.00	0–15	0.00–4.60
	Apr 26 to May 2, 2021	343	0–24	0.00–7.52	0–0	0.00–0.00
	May 3 to May 9, 2021	306	0–0	0.00–0.00	0–5	0.00–1.61
	May 10 to May 16, 2021	326	0–18	0.00–5.84	0–0	0.00–0.00
	May 17 to May 23, 2021	296	0–0	0.00–0.00	0–8	0.00–2.63
	May 24 to May 30, 2021	264	0–0	0.00–0.00	0–35	0.00–11.71
Gifu						
	Apr 5 to Apr 11, 2021	278	0–0	0.00–0.00	0–17	0.00–5.76
	Apr 12 to Apr 18, 2021	272	0–0	0.00–0.00	0–17	0.00–5.88
	Apr 19 to Apr 25, 2021	292	0–10	0.00–3.55	0–0	0.00–0.00
	Apr 26 to May 2, 2021	282	0–6	0.00–2.17	0–0	0.00–0.00
	May 3 to May 9, 2021	289	0–16	0.00–5.86	0–0	0.00–0.00
	May 10 to May 16, 2021	302	0–33	0.00–12.27	0–0	0.00–0.00
	May 17 to May 23, 2021	289	0–24	0.00–9.06	0–0	0.00–0.00
	May 24 to May 30, 2021	264	0–1	0.00–0.38	0–0	0.00–0.00
Shizuoka						
	Apr 5 to Apr 11, 2021	539	0–18	0.00–3.45	0–0	0.00–0.00
	Apr 12 to Apr 18, 2021	507	0–0	0.00–0.00	0–8	0.00–1.55
	Apr 19 to Apr 25, 2021	513	0–9	0.00–1.79	0–0	0.00–0.00
	Apr 26 to May 2, 2021	526	0–29	0.00–5.84	0–0	0.00–0.00
	May 3 to May 9, 2021	508	0–20	0.00–4.10	0–0	0.00–0.00
	May 10 to May 16, 2021	481	0–4	0.00–0.84	0–0	0.00–0.00
	May 17 to May 23, 2021	494	0–24	0.00–5.11	0–0	0.00–0.00
	May 24 to May 30, 2021	470	0–8	0.00–1.73	0–0	0.00–0.00
Aichi						
	Apr 5 to Apr 11, 2021	861	0–0	0.00–0.00	8–85	0.85–8.99
	Apr 12 to Apr 18, 2021	942	0–11	0.00–1.18	0–0	0.00–0.00
	Apr 19 to Apr 25, 2021	945	0–23	0.00–2.49	0–0	0.00–0.00
	Apr 26 to May 2, 2021	947	0–34	0.00–3.72	0–0	0.00–0.00
	May 3 to May 9, 2021	904	0–10	0.00–1.12	0–0	0.00–0.00
	May 10 to May 16, 2021	932	0–58	0.00–6.64	0–0	0.00–0.00
	May 17 to May 23, 2021	790	0–0	0.00–0.00	0–71	0.00–8.25
	May 24 to May 30, 2021	841	0–0	0.00–0.00	0–5	0.00–0.59
Mie						
	Apr 5 to Apr 11, 2021	255	0–0	0.00–0.00	0–9	0.00–3.41
	Apr 12 to Apr 18, 2021	254	0–0	0.00–0.00	0–5	0.00–1.93
	Apr 19 to Apr 25, 2021	251	0–0	0.00–0.00	0–4	0.00–1.57
	Apr 26 to May 2, 2021	279	0–30	0.00–12.05	0–0	0.00–0.00
	May 3 to May 9, 2021	273	0–30	0.00–12.35	0–0	0.00–0.00
	May 10 to May 16, 2021	258	0–19	0.00–7.95	0–0	0.00–0.00
	May 17 to May 23, 2021	241	0–7	0.00–2.99	0–0	0.00–0.00
	May 24 to May 30, 2021	238	0–7	0.00–3.03	0–0	0.00–0.00
Shiga						

	Apr 5 to Apr 11, 2021	200	0–14	0.00–7.53	0–0	0.00–0.00
	Apr 12 to Apr 18, 2021	193	0–9	0.00–4.89	0–0	0.00–0.00
	Apr 19 to Apr 25, 2021	164	0–0	0.00–0.00	0–15	0.00–8.38
	Apr 26 to May 2, 2021	192	0–16	0.00–9.09	0–0	0.00–0.00
	May 3 to May 9, 2021	194	0–21	0.00–12.14	0–0	0.00–0.00
	May 10 to May 16, 2021	163	0–0	0.00–0.00	0–6	0.00–3.55
	May 17 to May 23, 2021	182	0–15	0.00–8.98	0–0	0.00–0.00
	May 24 to May 30, 2021	174	0–9	0.00–5.45	0–0	0.00–0.00
Kyoto						
	Apr 5 to Apr 11, 2021	356	0–0	0.00–0.00	0–22	0.00–5.82
	Apr 12 to Apr 18, 2021	377	0–5	0.00–1.34	0–0	0.00–0.00
	Apr 19 to Apr 25, 2021	363	0–0	0.00–0.00	0–4	0.00–1.09
	Apr 26 to May 2, 2021	385	0–22	0.00–6.06	0–0	0.00–0.00
	May 3 to May 9, 2021	375	0–18	0.00–5.04	0–0	0.00–0.00
	May 10 to May 16, 2021	349	0–2	0.00–0.58	0–0	0.00–0.00
	May 17 to May 23, 2021	305	0–0	0.00–0.00	0–38	0.00–11.08
	May 24 to May 30, 2021	331	0–0	0.00–0.00	0–7	0.00–2.07
Osaka						
	Apr 5 to Apr 11, 2021	1226	0–0	0.00–0.00	0–17	0.00–1.37
	Apr 12 to Apr 18, 2021	1187	0–0	0.00–0.00	0–54	0.00–4.35
	Apr 19 to Apr 25, 2021	1329	2–99	0.16–8.05	0–0	0.00–0.00
	Apr 26 to May 2, 2021	1189	0–0	0.00–0.00	0–18	0.00–1.49
	May 3 to May 9, 2021	1259	0–76	0.00–6.42	0–0	0.00–0.00
	May 10 to May 16, 2021	1199	0–33	0.00–2.83	0–0	0.00–0.00
	May 17 to May 23, 2021	1147	0–0	0.00–0.00	0–7	0.00–0.61
	May 24 to May 30, 2021	1165	0–22	0.00–1.92	0–0	0.00–0.00
Hyogo						
	Apr 5 to Apr 11, 2021	742	0–0	0.00–0.00	0–22	0.00–2.88
	Apr 12 to Apr 18, 2021	735	0–0	0.00–0.00	0–13	0.00–1.74
	Apr 19 to Apr 25, 2021	743	0–9	0.00–1.23	0–0	0.00–0.00
	Apr 26 to May 2, 2021	753	0–33	0.00–4.58	0–0	0.00–0.00
	May 3 to May 9, 2021	734	0–24	0.00–3.38	0–0	0.00–0.00
	May 10 to May 16, 2021	713	0–9	0.00–1.28	0–0	0.00–0.00
	May 17 to May 23, 2021	731	0–35	0.00–5.03	0–0	0.00–0.00
	May 24 to May 30, 2021	685	0–0	0.00–0.00	0–1	0.00–0.15
Nara						
	Apr 5 to Apr 11, 2021	178	0–0	0.00–0.00	0–17	0.00–8.72
	Apr 12 to Apr 18, 2021	193	0–0	0.00–0.00	0–0	0.00–0.00
	Apr 19 to Apr 25, 2021	223	3–33	1.58–17.37	0–0	0.00–0.00
	Apr 26 to May 2, 2021	170	0–0	0.00–0.00	0–17	0.00–9.09
	May 3 to May 9, 2021	182	0–0	0.00–0.00	0–3	0.00–1.62
	May 10 to May 16, 2021	194	0–12	0.00–6.59	0–0	0.00–0.00
	May 17 to May 23, 2021	170	0–0	0.00–0.00	0–8	0.00–4.49
	May 24 to May 30, 2021	181	0–5	0.00–2.84	0–0	0.00–0.00
Wakayama						
	Apr 5 to Apr 11, 2021	168	0–0	0.00–0.00	0–2	0.00–1.18

	Apr 12 to Apr 18, 2021	170	0–2	0.00–1.19	0–0	0.00–0.00
	Apr 19 to Apr 25, 2021	174	0–7	0.00–4.19	0–0	0.00–0.00
	Apr 26 to May 2, 2021	156	0–0	0.00–0.00	0–7	0.00–4.29
	May 3 to May 9, 2021	159	0–0	0.00–0.00	0–1	0.00–0.63
	May 10 to May 16, 2021	146	0–0	0.00–0.00	0–10	0.00–6.41
	May 17 to May 23, 2021	133	0–0	0.00–0.00	0–16	0.00–10.74
	May 24 to May 30, 2021	148	0–1	0.00–0.68	0–0	0.00–0.00
Tottori						
	Apr 5 to Apr 11, 2021	85	0–0	0.00–0.00	0–10	0.00–10.53
	Apr 12 to Apr 18, 2021	91	0–0	0.00–0.00	0–3	0.00–3.19
	Apr 19 to Apr 25, 2021	88	0–0	0.00–0.00	0–3	0.00–3.30
	Apr 26 to May 2, 2021	87	0–0	0.00–0.00	0–2	0.00–2.25
	May 3 to May 9, 2021	101	0–13	0.00–14.77	0–0	0.00–0.00
	May 10 to May 16, 2021	98	0–11	0.00–12.64	0–0	0.00–0.00
	May 17 to May 23, 2021	87	0–2	0.00–2.35	0–0	0.00–0.00
	May 24 to May 30, 2021	84	0–0	0.00–0.00	0–1	0.00–1.18
Shimane						
	Apr 5 to Apr 11, 2021	105	0–0	0.00–0.00	2–25	1.54–19.23
	Apr 12 to Apr 18, 2021	148	0–20	0.00–15.63	0–0	0.00–0.00
	Apr 19 to Apr 25, 2021	133	0–7	0.00–5.56	0–0	0.00–0.00
	Apr 26 to May 2, 2021	130	0–6	0.00–4.84	0–0	0.00–0.00
	May 3 to May 9, 2021	137	0–15	0.00–12.30	0–0	0.00–0.00
	May 10 to May 16, 2021	128	0–11	0.00–9.40	0–0	0.00–0.00
	May 17 to May 23, 2021	121	0–6	0.00–5.22	0–0	0.00–0.00
	May 24 to May 30, 2021	120	0–7	0.00–6.19	0–0	0.00–0.00
Okayama						
	Apr 5 to Apr 11, 2021	304	0–0	0.00–0.00	0–0	0.00–0.00
	Apr 12 to Apr 18, 2021	310	0–11	0.00–3.68	0–0	0.00–0.00
	Apr 19 to Apr 25, 2021	320	0–26	0.00–8.84	0–0	0.00–0.00
	Apr 26 to May 2, 2021	287	0–0	0.00–0.00	0–6	0.00–2.05
	May 3 to May 9, 2021	287	0–0	0.00–0.00	0–5	0.00–1.71
	May 10 to May 16, 2021	304	0–15	0.00–5.19	0–0	0.00–0.00
	May 17 to May 23, 2021	261	0–0	0.00–0.00	0–22	0.00–7.77
	May 24 to May 30, 2021	278	0–0	0.00–0.00	0–0	0.00–0.00
Hiroshima						
	Apr 5 to Apr 11, 2021	433	0–9	0.00–2.12	0–0	0.00–0.00
	Apr 12 to Apr 18, 2021	416	0–0	0.00–0.00	0–2	0.00–0.48
	Apr 19 to Apr 25, 2021	441	0–30	0.00–7.30	0–0	0.00–0.00
	Apr 26 to May 2, 2021	431	0–25	0.00–6.16	0–0	0.00–0.00
	May 3 to May 9, 2021	426	0–27	0.00–6.77	0–0	0.00–0.00
	May 10 to May 16, 2021	450	11–55	2.78–13.92	0–0	0.00–0.00
	May 17 to May 23, 2021	400	0–14	0.00–3.63	0–0	0.00–0.00
	May 24 to May 30, 2021	389	0–10	0.00–2.64	0–0	0.00–0.00
Yamaguchi						
	Apr 5 to Apr 11, 2021	256	0–0	0.00–0.00	0–11	0.00–4.12
	Apr 12 to Apr 18, 2021	281	0–18	0.00–6.84	0–0	0.00–0.00

	Apr 19 to Apr 25, 2021	286	0–25	0.00–9.58	0–0	0.00–0.00
	Apr 26 to May 2, 2021	283	0–24	0.00–9.27	0–0	0.00–0.00
	May 3 to May 9, 2021	281	0–26	0.00–10.20	0–0	0.00–0.00
	May 10 to May 16, 2021	243	0–0	0.00–0.00	0–9	0.00–3.57
	May 17 to May 23, 2021	252	0–3	0.00–1.20	0–0	0.00–0.00
	May 24 to May 30, 2021	274	0–31	0.00–12.76	0–0	0.00–0.00
Tokushima						
	Apr 5 to Apr 11, 2021	149	0–8	0.00–5.67	0–0	0.00–0.00
	Apr 12 to Apr 18, 2021	124	0–0	0.00–0.00	0–16	0.00–11.43
	Apr 19 to Apr 25, 2021	176	15–39	10.95–28.47	0–0	0.00–0.00
	Apr 26 to May 2, 2021	127	0–0	0.00–0.00	0–7	0.00–5.22
	May 3 to May 9, 2021	147	0–14	0.00–10.53	0–0	0.00–0.00
	May 10 to May 16, 2021	152	0–20	0.00–15.15	0–0	0.00–0.00
	May 17 to May 23, 2021	117	0–0	0.00–0.00	0–10	0.00–7.87
	May 24 to May 30, 2021	133	0–8	0.00–6.40	0–0	0.00–0.00
Kagawa						
	Apr 5 to Apr 11, 2021	154	0–0	0.00–0.00	0–5	0.00–3.14
	Apr 12 to Apr 18, 2021	163	0–5	0.00–3.16	0–0	0.00–0.00
	Apr 19 to Apr 25, 2021	136	0–0	0.00–0.00	0–19	0.00–12.26
	Apr 26 to May 2, 2021	151	0–0	0.00–0.00	0–1	0.00–0.66
	May 3 to May 9, 2021	144	0–0	0.00–0.00	0–6	0.00–4.00
	May 10 to May 16, 2021	147	0–4	0.00–2.80	0–0	0.00–0.00
	May 17 to May 23, 2021	137	0–0	0.00–0.00	0–4	0.00–2.84
	May 24 to May 30, 2021	145	0–9	0.00–6.62	0–0	0.00–0.00
Ehime						
	Apr 5 to Apr 11, 2021	231	0–0	0.00–0.00	0–9	0.00–3.75
	Apr 12 to Apr 18, 2021	246	0–9	0.00–3.80	0–0	0.00–0.00
	Apr 19 to Apr 25, 2021	239	0–5	0.00–2.14	0–0	0.00–0.00
	Apr 26 to May 2, 2021	253	0–23	0.00–10.00	0–0	0.00–0.00
	May 3 to May 9, 2021	226	0–0	0.00–0.00	0–2	0.00–0.88
	May 10 to May 16, 2021	261	7–39	3.15–17.57	0–0	0.00–0.00
	May 17 to May 23, 2021	223	0–4	0.00–1.83	0–0	0.00–0.00
	May 24 to May 30, 2021	242	0–27	0.00–12.56	0–0	0.00–0.00
Kochi						
	Apr 5 to Apr 11, 2021	145	0–0	0.00–0.00	0–5	0.00–3.33
	Apr 12 to Apr 18, 2021	147	0–0	0.00–0.00	0–4	0.00–2.65
	Apr 19 to Apr 25, 2021	160	0–12	0.00–8.11	0–0	0.00–0.00
	Apr 26 to May 2, 2021	146	0–0	0.00–0.00	0–1	0.00–0.68
	May 3 to May 9, 2021	134	0–0	0.00–0.00	0–9	0.00–6.29
	May 10 to May 16, 2021	156	0–15	0.00–10.64	0–0	0.00–0.00
	May 17 to May 23, 2021	152	0–14	0.00–10.14	0–0	0.00–0.00
	May 24 to May 30, 2021	155	0–19	0.00–13.97	0–0	0.00–0.00
Fukuoka						
	Apr 5 to Apr 11, 2021	733	0–0	0.00–0.00	4–76	0.49–9.39
	Apr 12 to Apr 18, 2021	804	0–2	0.00–0.25	0–0	0.00–0.00
	Apr 19 to Apr 25, 2021	907	44–118	5.58–14.96	0–0	0.00–0.00

	Apr 26 to May 2, 2021	792	0–16	0.00–2.06	0–0	0.00–0.00
	May 3 to May 9, 2021	805	0–44	0.00–5.78	0–0	0.00–0.00
	May 10 to May 16, 2021	812	0–60	0.00–7.98	0–0	0.00–0.00
	May 17 to May 23, 2021	743	0–0	0.00–0.00	0–2	0.00–0.27
	May 24 to May 30, 2021	751	0–19	0.00–2.60	0–0	0.00–0.00
Saga						
	Apr 5 to Apr 11, 2021	146	0–4	0.00–2.82	0–0	0.00–0.00
	Apr 12 to Apr 18, 2021	139	0–0	0.00–0.00	0–0	0.00–0.00
	Apr 19 to Apr 25, 2021	151	0–12	0.00–8.63	0–0	0.00–0.00
	Apr 26 to May 2, 2021	133	0–0	0.00–0.00	0–4	0.00–2.92
	May 3 to May 9, 2021	144	0–9	0.00–6.67	0–0	0.00–0.00
	May 10 to May 16, 2021	154	0–21	0.00–15.79	0–0	0.00–0.00
	May 17 to May 23, 2021	132	0–0	0.00–0.00	0–2	0.00–1.49
	May 24 to May 30, 2021	133	0–0	0.00–0.00	0–0	0.00–0.00
Nagasaki						
	Apr 5 to Apr 11, 2021	251	0–0	0.00–0.00	0–13	0.00–4.92
	Apr 12 to Apr 18, 2021	247	0–0	0.00–0.00	0–13	0.00–5.00
	Apr 19 to Apr 25, 2021	253	0–0	0.00–0.00	0–2	0.00–0.78
	Apr 26 to May 2, 2021	248	0–0	0.00–0.00	0–1	0.00–0.40
	May 3 to May 9, 2021	266	0–19	0.00–7.69	0–0	0.00–0.00
	May 10 to May 16, 2021	252	0–9	0.00–3.70	0–0	0.00–0.00
	May 17 to May 23, 2021	233	0–0	0.00–0.00	0–7	0.00–2.92
	May 24 to May 30, 2021	275	8–40	3.40–17.02	0–0	0.00–0.00
Kumamoto						
	Apr 5 to Apr 11, 2021	308	0–0	0.00–0.00	0–1	0.00–0.32
	Apr 12 to Apr 18, 2021	327	0–21	0.00–6.86	0–0	0.00–0.00
	Apr 19 to Apr 25, 2021	333	0–30	0.00–9.90	0–0	0.00–0.00
	Apr 26 to May 2, 2021	295	0–0	0.00–0.00	0–5	0.00–1.67
	May 3 to May 9, 2021	296	0–2	0.00–0.68	0–0	0.00–0.00
	May 10 to May 16, 2021	300	0–12	0.00–4.17	0–0	0.00–0.00
	May 17 to May 23, 2021	303	0–17	0.00–5.94	0–0	0.00–0.00
	May 24 to May 30, 2021	281	0–0	0.00–0.00	0–2	0.00–0.71
Oita						
	Apr 5 to Apr 11, 2021	180	0–0	0.00–0.00	0–18	0.00–9.09
	Apr 12 to Apr 18, 2021	209	0–14	0.00–7.18	0–0	0.00–0.00
	Apr 19 to Apr 25, 2021	224	3–31	1.55–16.06	0–0	0.00–0.00
	Apr 26 to May 2, 2021	180	0–0	0.00–0.00	0–12	0.00–6.25
	May 3 to May 9, 2021	168	0–0	0.00–0.00	0–19	0.00–10.16
	May 10 to May 16, 2021	223	9–37	4.84–19.89	0–0	0.00–0.00
	May 17 to May 23, 2021	181	0–0	0.00–0.00	0–4	0.00–2.16
	May 24 to May 30, 2021	186	0–7	0.00–3.91	0–0	0.00–0.00
Miyazaki						
	Apr 5 to Apr 11, 2021	213	0–18	0.00–9.23	0–0	0.00–0.00
	Apr 12 to Apr 18, 2021	188	0–0	0.00–0.00	0–6	0.00–3.09
	Apr 19 to Apr 25, 2021	224	5–32	2.60–16.67	0–0	0.00–0.00
	Apr 26 to May 2, 2021	203	0–12	0.00–6.28	0–0	0.00–0.00

	May 3 to May 9, 2021	202	0–12	0.00–6.32	0–0	0.00–0.00
	May 10 to May 16, 2021	188	0–0	0.00–0.00	0–0	0.00–0.00
	May 17 to May 23, 2021	187	0–1	0.00–0.54	0–0	0.00–0.00
	May 24 to May 30, 2021	181	0–0	0.00–0.00	0–1	0.00–0.55
Kagoshima						
	Apr 5 to Apr 11, 2021	327	0–24	0.00–7.92	0–0	0.00–0.00
	Apr 12 to Apr 18, 2021	273	0–0	0.00–0.00	0–26	0.00–8.70
	Apr 19 to Apr 25, 2021	298	0–5	0.00–1.71	0–0	0.00–0.00
	Apr 26 to May 2, 2021	277	0–0	0.00–0.00	0–13	0.00–4.48
	May 3 to May 9, 2021	313	0–26	0.00–9.06	0–0	0.00–0.00
	May 10 to May 16, 2021	266	0–0	0.00–0.00	0–16	0.00–5.67
	May 17 to May 23, 2021	287	0–7	0.00–2.50	0–0	0.00–0.00
	May 24 to May 30, 2021	290	0–15	0.00–5.45	0–0	0.00–0.00
Okinawa						
	Apr 5 to Apr 11, 2021	168	0–0	0.00–0.00	0–5	0.00–2.89
	Apr 12 to Apr 18, 2021	180	0–7	0.00–4.05	0–0	0.00–0.00
	Apr 19 to Apr 25, 2021	179	0–10	0.00–5.92	0–0	0.00–0.00
	Apr 26 to May 2, 2021	158	0–0	0.00–0.00	0–12	0.00–7.06
	May 3 to May 9, 2021	154	0–0	0.00–0.00	0–14	0.00–8.33
	May 10 to May 16, 2021	162	0–0	0.00–0.00	0–6	0.00–3.57
	May 17 to May 23, 2021	151	0–0	0.00–0.00	0–16	0.00–9.58
	May 24 to May 30, 2021	159	0–0	0.00–0.00	0–6	0.00–3.64

Table A.3: Weekly number of observed and excess/exiguous deaths in Japan and 47 prefectures for non-COVID-19-related deaths in nursing homes and elderly care facilities from January 2020 through May 2021.

Prefecture	Week	Observed	Excess deaths	Percent excess	Exiguous deaths	Percent exiguous
Japan						
	Apr 5 to Apr 11, 2021	3565	0–47	0.00–1.34	0–0	0.00–0.00
	Apr 12 to Apr 18, 2021	3840	148–361	4.25–10.38	0–0	0.00–0.00
	Apr 19 to Apr 25, 2021	3725	48–274	1.39–7.94	0–0	0.00–0.00
	Apr 26 to May 2, 2021	3863	217–439	6.34–12.82	0–0	0.00–0.00
	May 3 to May 9, 2021	3856	278–485	8.25–14.39	0–0	0.00–0.00
	May 10 to May 16, 2021	3753	202–405	6.03–12.10	0–0	0.00–0.00
	May 17 to May 23, 2021	3641	133–328	4.01–9.90	0–0	0.00–0.00
	May 24 to May 30, 2021	3494	0–190	0.00–5.75	0–0	0.00–0.00
Hokkaido						
	Apr 5 to Apr 11, 2021	91	0–6	0.00–7.06	0–0	0.00–0.00
	Apr 12 to Apr 18, 2021	105	1–20	1.18–23.53	0–0	0.00–0.00
	Apr 19 to Apr 25, 2021	89	0–2	0.00–2.30	0–0	0.00–0.00
	Apr 26 to May 2, 2021	89	0–3	0.00–3.49	0–0	0.00–0.00
	May 3 to May 9, 2021	108	2–21	2.30–24.14	0–0	0.00–0.00
	May 10 to May 16, 2021	79	0–0	0.00–0.00	0–7	0.00–8.14
	May 17 to May 23, 2021	80	0–0	0.00–0.00	0–5	0.00–5.88
	May 24 to May 30, 2021	94	0–11	0.00–13.25	0–0	0.00–0.00
Aomori						
	Apr 5 to Apr 11, 2021	64	0–9	0.00–16.36	0–0	0.00–0.00
	Apr 12 to Apr 18, 2021	58	0–3	0.00–5.45	0–0	0.00–0.00
	Apr 19 to Apr 25, 2021	52	0–0	0.00–0.00	0–2	0.00–3.70
	Apr 26 to May 2, 2021	55	0–2	0.00–3.77	0–0	0.00–0.00
	May 3 to May 9, 2021	59	0–7	0.00–13.46	0–0	0.00–0.00
	May 10 to May 16, 2021	58	0–7	0.00–13.73	0–0	0.00–0.00
	May 17 to May 23, 2021	63	0–12	0.00–23.53	0–0	0.00–0.00
	May 24 to May 30, 2021	57	0–6	0.00–11.76	0–0	0.00–0.00
Iwate						
	Apr 5 to Apr 11, 2021	48	0–1	0.00–2.13	0–0	0.00–0.00
	Apr 12 to Apr 18, 2021	59	0–12	0.00–25.53	0–0	0.00–0.00
	Apr 19 to Apr 25, 2021	39	0–0	0.00–0.00	0–8	0.00–17.02
	Apr 26 to May 2, 2021	50	0–4	0.00–8.70	0–0	0.00–0.00
	May 3 to May 9, 2021	53	0–7	0.00–15.22	0–0	0.00–0.00
	May 10 to May 16, 2021	42	0–0	0.00–0.00	0–2	0.00–4.55
	May 17 to May 23, 2021	45	0–1	0.00–2.27	0–0	0.00–0.00
	May 24 to May 30, 2021	42	0–0	0.00–0.00	0–3	0.00–6.67
Miyagi						
	Apr 5 to Apr 11, 2021	69	0–7	0.00–11.29	0–0	0.00–0.00
	Apr 12 to Apr 18, 2021	85	7–23	11.29–37.10	0–0	0.00–0.00
	Apr 19 to Apr 25, 2021	63	0–1	0.00–1.61	0–0	0.00–0.00
	Apr 26 to May 2, 2021	67	0–4	0.00–6.35	0–0	0.00–0.00
	May 3 to May 9, 2021	65	0–2	0.00–3.17	0–0	0.00–0.00

	May 10 to May 16, 2021	73	0–11	0.00–17.74	0–0	0.00–0.00
	May 17 to May 23, 2021	58	0–0	0.00–0.00	0–5	0.00–7.94
	May 24 to May 30, 2021	58	0–0	0.00–0.00	0–6	0.00–9.38
Akita						
	Apr 5 to Apr 11, 2021	40	0–1	0.00–2.56	0–0	0.00–0.00
	Apr 12 to Apr 18, 2021	55	4–16	10.26–41.03	0–0	0.00–0.00
	Apr 19 to Apr 25, 2021	29	0–0	0.00–0.00	0–10	0.00–25.64
	Apr 26 to May 2, 2021	41	0–3	0.00–7.89	0–0	0.00–0.00
	May 3 to May 9, 2021	54	5–17	13.51–45.95	0–0	0.00–0.00
	May 10 to May 16, 2021	36	0–0	0.00–0.00	0–0	0.00–0.00
	May 17 to May 23, 2021	38	0–2	0.00–5.56	0–0	0.00–0.00
	May 24 to May 30, 2021	40	0–4	0.00–11.11	0–0	0.00–0.00
Yamagata						
	Apr 5 to Apr 11, 2021	41	0–0	0.00–0.00	2–16	3.51–28.07
	Apr 12 to Apr 18, 2021	63	0–8	0.00–14.55	0–0	0.00–0.00
	Apr 19 to Apr 25, 2021	53	0–0	0.00–0.00	0–0	0.00–0.00
	Apr 26 to May 2, 2021	60	0–7	0.00–13.21	0–0	0.00–0.00
	May 3 to May 9, 2021	67	1–15	1.92–28.85	0–0	0.00–0.00
	May 10 to May 16, 2021	55	0–4	0.00–7.84	0–0	0.00–0.00
	May 17 to May 23, 2021	69	3–17	5.77–32.69	0–0	0.00–0.00
	May 24 to May 30, 2021	62	0–11	0.00–21.57	0–0	0.00–0.00
Fukushima						
	Apr 5 to Apr 11, 2021	61	0–3	0.00–5.17	0–0	0.00–0.00
	Apr 12 to Apr 18, 2021	59	0–1	0.00–1.72	0–0	0.00–0.00
	Apr 19 to Apr 25, 2021	58	0–0	0.00–0.00	0–0	0.00–0.00
	Apr 26 to May 2, 2021	65	0–6	0.00–10.17	0–0	0.00–0.00
	May 3 to May 9, 2021	67	0–9	0.00–15.52	0–0	0.00–0.00
	May 10 to May 16, 2021	62	0–5	0.00–8.77	0–0	0.00–0.00
	May 17 to May 23, 2021	55	0–0	0.00–0.00	0–2	0.00–3.51
	May 24 to May 30, 2021	55	0–0	0.00–0.00	0–2	0.00–3.51
Ibaraki						
	Apr 5 to Apr 11, 2021	56	0–0	0.00–0.00	0–11	0.00–16.42
	Apr 12 to Apr 18, 2021	85	4–20	6.15–30.77	0–0	0.00–0.00
	Apr 19 to Apr 25, 2021	53	0–0	0.00–0.00	0–11	0.00–17.19
	Apr 26 to May 2, 2021	59	0–0	0.00–0.00	0–3	0.00–4.84
	May 3 to May 9, 2021	71	0–10	0.00–16.39	0–0	0.00–0.00
	May 10 to May 16, 2021	80	3–19	4.92–31.15	0–0	0.00–0.00
	May 17 to May 23, 2021	68	0–10	0.00–17.24	0–0	0.00–0.00
	May 24 to May 30, 2021	68	0–11	0.00–19.30	0–0	0.00–0.00
Tochigi						
	Apr 5 to Apr 11, 2021	49	0–0	0.00–0.00	0–13	0.00–20.97
	Apr 12 to Apr 18, 2021	78	1–18	1.67–30.00	0–0	0.00–0.00
	Apr 19 to Apr 25, 2021	70	0–12	0.00–20.69	0–0	0.00–0.00
	Apr 26 to May 2, 2021	72	0–16	0.00–28.57	0–0	0.00–0.00
	May 3 to May 9, 2021	52	0–0	0.00–0.00	0–2	0.00–3.70
	May 10 to May 16, 2021	53	0–1	0.00–1.92	0–0	0.00–0.00

	May 17 to May 23, 2021	65	0–14	0.00–27.45	0–0	0.00–0.00
	May 24 to May 30, 2021	61	0–11	0.00–22.00	0–0	0.00–0.00
Gunma						
	Apr 5 to Apr 11, 2021	76	0–2	0.00–2.70	0–0	0.00–0.00
	Apr 12 to Apr 18, 2021	86	0–13	0.00–17.81	0–0	0.00–0.00
	Apr 19 to Apr 25, 2021	104	17–34	24.29–48.57	0–0	0.00–0.00
	Apr 26 to May 2, 2021	76	0–7	0.00–10.14	0–0	0.00–0.00
	May 3 to May 9, 2021	77	0–9	0.00–13.24	0–0	0.00–0.00
	May 10 to May 16, 2021	73	0–8	0.00–12.31	0–0	0.00–0.00
	May 17 to May 23, 2021	59	0–0	0.00–0.00	0–6	0.00–9.23
	May 24 to May 30, 2021	81	0–15	0.00–22.73	0–0	0.00–0.00
Saitama						
	Apr 5 to Apr 11, 2021	169	0–2	0.00–1.20	0–0	0.00–0.00
	Apr 12 to Apr 18, 2021	168	0–5	0.00–3.07	0–0	0.00–0.00
	Apr 19 to Apr 25, 2021	173	0–10	0.00–6.13	0–0	0.00–0.00
	Apr 26 to May 2, 2021	198	10–35	6.13–21.47	0–0	0.00–0.00
	May 3 to May 9, 2021	191	4–29	2.47–17.90	0–0	0.00–0.00
	May 10 to May 16, 2021	165	0–6	0.00–3.77	0–0	0.00–0.00
	May 17 to May 23, 2021	165	0–7	0.00–4.43	0–0	0.00–0.00
	May 24 to May 30, 2021	171	0–15	0.00–9.62	0–0	0.00–0.00
Chiba						
	Apr 5 to Apr 11, 2021	126	0–4	0.00–3.28	0–0	0.00–0.00
	Apr 12 to Apr 18, 2021	124	0–2	0.00–1.64	0–0	0.00–0.00
	Apr 19 to Apr 25, 2021	147	1–23	0.81–18.55	0–0	0.00–0.00
	Apr 26 to May 2, 2021	127	0–2	0.00–1.60	0–0	0.00–0.00
	May 3 to May 9, 2021	136	0–11	0.00–8.80	0–0	0.00–0.00
	May 10 to May 16, 2021	135	0–13	0.00–10.66	0–0	0.00–0.00
	May 17 to May 23, 2021	121	0–0	0.00–0.00	0–0	0.00–0.00
	May 24 to May 30, 2021	129	0–6	0.00–4.88	0–0	0.00–0.00
Tokyo						
	Apr 5 to Apr 11, 2021	277	0–0	0.00–0.00	0–23	0.00–7.67
	Apr 12 to Apr 18, 2021	304	0–6	0.00–2.01	0–0	0.00–0.00
	Apr 19 to Apr 25, 2021	302	0–6	0.00–2.03	0–0	0.00–0.00
	Apr 26 to May 2, 2021	326	0–34	0.00–11.64	0–0	0.00–0.00
	May 3 to May 9, 2021	358	36–69	12.46–23.88	0–0	0.00–0.00
	May 10 to May 16, 2021	322	5–39	1.77–13.78	0–0	0.00–0.00
	May 17 to May 23, 2021	322	8–42	2.86–15.00	0–0	0.00–0.00
	May 24 to May 30, 2021	265	0–0	0.00–0.00	0–14	0.00–5.02
Kanagawa						
	Apr 5 to Apr 11, 2021	268	0–20	0.00–8.06	0–0	0.00–0.00
	Apr 12 to Apr 18, 2021	258	0–12	0.00–4.88	0–0	0.00–0.00
	Apr 19 to Apr 25, 2021	238	0–0	0.00–0.00	0–8	0.00–3.25
	Apr 26 to May 2, 2021	303	23–56	9.31–22.67	0–0	0.00–0.00
	May 3 to May 9, 2021	311	33–66	13.47–26.94	0–0	0.00–0.00
	May 10 to May 16, 2021	278	0–31	0.00–12.55	0–0	0.00–0.00
	May 17 to May 23, 2021	282	5–38	2.05–15.57	0–0	0.00–0.00

	May 24 to May 30, 2021	276	0–32	0.00–13.11	0–0	0.00–0.00
Niigata						
	Apr 5 to Apr 11, 2021	111	0–12	0.00–12.12	0–0	0.00–0.00
	Apr 12 to Apr 18, 2021	111	0–11	0.00–11.00	0–0	0.00–0.00
	Apr 19 to Apr 25, 2021	103	0–3	0.00–3.00	0–0	0.00–0.00
	Apr 26 to May 2, 2021	96	0–0	0.00–0.00	0–5	0.00–4.95
	May 3 to May 9, 2021	103	0–4	0.00–4.04	0–0	0.00–0.00
	May 10 to May 16, 2021	126	8–28	8.16–28.57	0–0	0.00–0.00
	May 17 to May 23, 2021	109	0–14	0.00–14.74	0–0	0.00–0.00
	May 24 to May 30, 2021	107	0–14	0.00–15.05	0–0	0.00–0.00
Toyama						
	Apr 5 to Apr 11, 2021	42	0–4	0.00–10.53	0–0	0.00–0.00
	Apr 12 to Apr 18, 2021	48	0–10	0.00–26.32	0–0	0.00–0.00
	Apr 19 to Apr 25, 2021	47	0–10	0.00–27.03	0–0	0.00–0.00
	Apr 26 to May 2, 2021	50	1–13	2.70–35.14	0–0	0.00–0.00
	May 3 to May 9, 2021	44	0–7	0.00–18.92	0–0	0.00–0.00
	May 10 to May 16, 2021	30	0–0	0.00–0.00	0–7	0.00–18.92
	May 17 to May 23, 2021	33	0–0	0.00–0.00	0–4	0.00–10.81
	May 24 to May 30, 2021	33	0–0	0.00–0.00	0–3	0.00–8.33
Ishikawa						
	Apr 5 to Apr 11, 2021	46	0–5	0.00–12.20	0–0	0.00–0.00
	Apr 12 to Apr 18, 2021	48	0–8	0.00–20.00	0–0	0.00–0.00
	Apr 19 to Apr 25, 2021	42	0–2	0.00–5.00	0–0	0.00–0.00
	Apr 26 to May 2, 2021	40	0–0	0.00–0.00	0–1	0.00–2.44
	May 3 to May 9, 2021	39	0–0	0.00–0.00	0–2	0.00–4.88
	May 10 to May 16, 2021	38	0–0	0.00–0.00	0–3	0.00–7.32
	May 17 to May 23, 2021	47	0–7	0.00–17.50	0–0	0.00–0.00
	May 24 to May 30, 2021	43	0–3	0.00–7.50	0–0	0.00–0.00
Fukui						
	Apr 5 to Apr 11, 2021	31	0–2	0.00–6.90	0–0	0.00–0.00
	Apr 12 to Apr 18, 2021	32	0–4	0.00–14.29	0–0	0.00–0.00
	Apr 19 to Apr 25, 2021	34	0–6	0.00–21.43	0–0	0.00–0.00
	Apr 26 to May 2, 2021	33	0–6	0.00–22.22	0–0	0.00–0.00
	May 3 to May 9, 2021	33	0–6	0.00–22.22	0–0	0.00–0.00
	May 10 to May 16, 2021	31	0–5	0.00–19.23	0–0	0.00–0.00
	May 17 to May 23, 2021	37	1–11	3.85–42.31	0–0	0.00–0.00
	May 24 to May 30, 2021	27	0–1	0.00–3.85	0–0	0.00–0.00
Yamanashi						
	Apr 5 to Apr 11, 2021	30	0–2	0.00–7.14	0–0	0.00–0.00
	Apr 12 to Apr 18, 2021	27	0–0	0.00–0.00	0–0	0.00–0.00
	Apr 19 to Apr 25, 2021	30	0–3	0.00–11.11	0–0	0.00–0.00
	Apr 26 to May 2, 2021	40	2–13	7.41–48.15	0–0	0.00–0.00
	May 3 to May 9, 2021	31	0–5	0.00–19.23	0–0	0.00–0.00
	May 10 to May 16, 2021	32	0–7	0.00–28.00	0–0	0.00–0.00
	May 17 to May 23, 2021	30	0–5	0.00–20.00	0–0	0.00–0.00
	May 24 to May 30, 2021	28	0–4	0.00–16.67	0–0	0.00–0.00

Nagano						
	Apr 5 to Apr 11, 2021	89	0–0	0.00–0.00	0–3	0.00–3.26
	Apr 12 to Apr 18, 2021	91	0–0	0.00–0.00	0–0	0.00–0.00
	Apr 19 to Apr 25, 2021	94	0–2	0.00–2.17	0–0	0.00–0.00
	Apr 26 to May 2, 2021	102	0–11	0.00–12.09	0–0	0.00–0.00
	May 3 to May 9, 2021	76	0–0	0.00–0.00	0–12	0.00–13.64
	May 10 to May 16, 2021	103	0–15	0.00–17.05	0–0	0.00–0.00
	May 17 to May 23, 2021	95	0–7	0.00–7.95	0–0	0.00–0.00
	May 24 to May 30, 2021	81	0–0	0.00–0.00	0–6	0.00–6.90
Gifu						
	Apr 5 to Apr 11, 2021	77	0–8	0.00–11.59	0–0	0.00–0.00
	Apr 12 to Apr 18, 2021	74	0–6	0.00–8.82	0–0	0.00–0.00
	Apr 19 to Apr 25, 2021	79	0–12	0.00–17.91	0–0	0.00–0.00
	Apr 26 to May 2, 2021	71	0–4	0.00–5.97	0–0	0.00–0.00
	May 3 to May 9, 2021	81	0–15	0.00–22.73	0–0	0.00–0.00
	May 10 to May 16, 2021	64	0–0	0.00–0.00	0–2	0.00–3.03
	May 17 to May 23, 2021	76	0–12	0.00–18.75	0–0	0.00–0.00
	May 24 to May 30, 2021	47	0–0	0.00–0.00	2–17	3.13–26.56
Shizuoka						
	Apr 5 to Apr 11, 2021	140	0–0	0.00–0.00	0–22	0.00–13.58
	Apr 12 to Apr 18, 2021	172	0–12	0.00–7.50	0–0	0.00–0.00
	Apr 19 to Apr 25, 2021	161	0–2	0.00–1.26	0–0	0.00–0.00
	Apr 26 to May 2, 2021	171	0–17	0.00–11.04	0–0	0.00–0.00
	May 3 to May 9, 2021	151	0–0	0.00–0.00	0–1	0.00–0.66
	May 10 to May 16, 2021	172	0–23	0.00–15.44	0–0	0.00–0.00
	May 17 to May 23, 2021	171	0–23	0.00–15.54	0–0	0.00–0.00
	May 24 to May 30, 2021	142	0–0	0.00–0.00	0–6	0.00–4.05
Aichi						
	Apr 5 to Apr 11, 2021	187	0–0	0.00–0.00	0–24	0.00–11.37
	Apr 12 to Apr 18, 2021	199	0–0	0.00–0.00	0–10	0.00–4.78
	Apr 19 to Apr 25, 2021	192	0–0	0.00–0.00	0–16	0.00–7.69
	Apr 26 to May 2, 2021	199	0–0	0.00–0.00	0–7	0.00–3.40
	May 3 to May 9, 2021	210	0–7	0.00–3.45	0–0	0.00–0.00
	May 10 to May 16, 2021	209	0–9	0.00–4.50	0–0	0.00–0.00
	May 17 to May 23, 2021	194	0–0	0.00–0.00	0–3	0.00–1.52
	May 24 to May 30, 2021	218	0–22	0.00–11.22	0–0	0.00–0.00
Mie						
	Apr 5 to Apr 11, 2021	70	0–0	0.00–0.00	0–4	0.00–5.41
	Apr 12 to Apr 18, 2021	82	0–10	0.00–13.89	0–0	0.00–0.00
	Apr 19 to Apr 25, 2021	73	0–4	0.00–5.80	0–0	0.00–0.00
	Apr 26 to May 2, 2021	70	0–3	0.00–4.48	0–0	0.00–0.00
	May 3 to May 9, 2021	61	0–0	0.00–0.00	0–5	0.00–7.58
	May 10 to May 16, 2021	80	0–14	0.00–21.21	0–0	0.00–0.00
	May 17 to May 23, 2021	88	5–22	7.58–33.33	0–0	0.00–0.00
	May 24 to May 30, 2021	55	0–0	0.00–0.00	0–12	0.00–17.91
Shiga						

	Apr 5 to Apr 11, 2021	44	2–15	6.90–51.72	0–0	0.00–0.00
	Apr 12 to Apr 18, 2021	39	0–10	0.00–34.48	0–0	0.00–0.00
	Apr 19 to Apr 25, 2021	28	0–0	0.00–0.00	0–0	0.00–0.00
	Apr 26 to May 2, 2021	38	0–9	0.00–31.03	0–0	0.00–0.00
	May 3 to May 9, 2021	30	0–1	0.00–3.45	0–0	0.00–0.00
	May 10 to May 16, 2021	38	0–9	0.00–31.03	0–0	0.00–0.00
	May 17 to May 23, 2021	30	0–0	0.00–0.00	0–0	0.00–0.00
	May 24 to May 30, 2021	36	0–7	0.00–24.14	0–0	0.00–0.00
Kyoto						
	Apr 5 to Apr 11, 2021	67	0–1	0.00–1.52	0–0	0.00–0.00
	Apr 12 to Apr 18, 2021	61	0–0	0.00–0.00	0–3	0.00–4.69
	Apr 19 to Apr 25, 2021	71	0–6	0.00–9.23	0–0	0.00–0.00
	Apr 26 to May 2, 2021	66	0–1	0.00–1.54	0–0	0.00–0.00
	May 3 to May 9, 2021	74	0–10	0.00–15.63	0–0	0.00–0.00
	May 10 to May 16, 2021	70	0–8	0.00–12.90	0–0	0.00–0.00
	May 17 to May 23, 2021	53	0–0	0.00–0.00	0–8	0.00–13.11
	May 24 to May 30, 2021	73	0–12	0.00–19.67	0–0	0.00–0.00
Osaka						
	Apr 5 to Apr 11, 2021	200	0–10	0.00–5.26	0–0	0.00–0.00
	Apr 12 to Apr 18, 2021	204	0–16	0.00–8.51	0–0	0.00–0.00
	Apr 19 to Apr 25, 2021	196	0–10	0.00–5.38	0–0	0.00–0.00
	Apr 26 to May 2, 2021	212	0–24	0.00–12.77	0–0	0.00–0.00
	May 3 to May 9, 2021	200	0–18	0.00–9.89	0–0	0.00–0.00
	May 10 to May 16, 2021	225	13–43	7.14–23.63	0–0	0.00–0.00
	May 17 to May 23, 2021	184	0–5	0.00–2.79	0–0	0.00–0.00
	May 24 to May 30, 2021	174	0–0	0.00–0.00	0–1	0.00–0.57
Hyogo						
	Apr 5 to Apr 11, 2021	133	0–0	0.00–0.00	0–12	0.00–8.28
	Apr 12 to Apr 18, 2021	154	0–9	0.00–6.21	0–0	0.00–0.00
	Apr 19 to Apr 25, 2021	160	0–14	0.00–9.59	0–0	0.00–0.00
	Apr 26 to May 2, 2021	164	0–18	0.00–12.33	0–0	0.00–0.00
	May 3 to May 9, 2021	160	0–16	0.00–11.11	0–0	0.00–0.00
	May 10 to May 16, 2021	147	0–4	0.00–2.80	0–0	0.00–0.00
	May 17 to May 23, 2021	167	0–24	0.00–16.78	0–0	0.00–0.00
	May 24 to May 30, 2021	124	0–0	0.00–0.00	0–15	0.00–10.79
Nara						
	Apr 5 to Apr 11, 2021	39	0–1	0.00–2.63	0–0	0.00–0.00
	Apr 12 to Apr 18, 2021	48	0–10	0.00–26.32	0–0	0.00–0.00
	Apr 19 to Apr 25, 2021	46	0–7	0.00–17.95	0–0	0.00–0.00
	Apr 26 to May 2, 2021	51	0–12	0.00–30.77	0–0	0.00–0.00
	May 3 to May 9, 2021	25	0–0	0.00–0.00	2–14	5.13–35.90
	May 10 to May 16, 2021	44	0–5	0.00–12.82	0–0	0.00–0.00
	May 17 to May 23, 2021	54	2–15	5.13–38.46	0–0	0.00–0.00
	May 24 to May 30, 2021	38	0–0	0.00–0.00	0–1	0.00–2.56
Wakayama						
	Apr 5 to Apr 11, 2021	36	0–0	0.00–0.00	0–0	0.00–0.00

	Apr 12 to Apr 18, 2021	39	0–3	0.00–8.33	0–0	0.00–0.00
	Apr 19 to Apr 25, 2021	40	0–4	0.00–11.11	0–0	0.00–0.00
	Apr 26 to May 2, 2021	39	0–4	0.00–11.43	0–0	0.00–0.00
	May 3 to May 9, 2021	40	0–6	0.00–17.65	0–0	0.00–0.00
	May 10 to May 16, 2021	41	0–7	0.00–20.59	0–0	0.00–0.00
	May 17 to May 23, 2021	31	0–0	0.00–0.00	0–3	0.00–8.82
	May 24 to May 30, 2021	36	0–2	0.00–5.88	0–0	0.00–0.00
Tottori						
	Apr 5 to Apr 11, 2021	35	0–9	0.00–34.62	0–0	0.00–0.00
	Apr 12 to Apr 18, 2021	32	0–6	0.00–23.08	0–0	0.00–0.00
	Apr 19 to Apr 25, 2021	34	0–9	0.00–36.00	0–0	0.00–0.00
	Apr 26 to May 2, 2021	22	0–0	0.00–0.00	0–4	0.00–15.38
	May 3 to May 9, 2021	29	0–3	0.00–11.54	0–0	0.00–0.00
	May 10 to May 16, 2021	30	0–4	0.00–15.38	0–0	0.00–0.00
	May 17 to May 23, 2021	19	0–0	0.00–0.00	0–6	0.00–24.00
	May 24 to May 30, 2021	29	0–5	0.00–20.83	0–0	0.00–0.00
Shimane						
	Apr 5 to Apr 11, 2021	28	0–0	0.00–0.00	0–7	0.00–20.00
	Apr 12 to Apr 18, 2021	32	0–0	0.00–0.00	0–2	0.00–5.88
	Apr 19 to Apr 25, 2021	35	0–0	0.00–0.00	0–0	0.00–0.00
	Apr 26 to May 2, 2021	41	0–7	0.00–20.59	0–0	0.00–0.00
	May 3 to May 9, 2021	34	0–1	0.00–3.03	0–0	0.00–0.00
	May 10 to May 16, 2021	34	0–2	0.00–6.25	0–0	0.00–0.00
	May 17 to May 23, 2021	43	1–12	3.23–38.71	0–0	0.00–0.00
	May 24 to May 30, 2021	39	0–9	0.00–30.00	0–0	0.00–0.00
Okayama						
	Apr 5 to Apr 11, 2021	53	0–0	0.00–0.00	0–3	0.00–5.36
	Apr 12 to Apr 18, 2021	54	0–0	0.00–0.00	0–1	0.00–1.82
	Apr 19 to Apr 25, 2021	58	0–3	0.00–5.45	0–0	0.00–0.00
	Apr 26 to May 2, 2021	66	0–12	0.00–22.22	0–0	0.00–0.00
	May 3 to May 9, 2021	52	0–0	0.00–0.00	0–1	0.00–1.89
	May 10 to May 16, 2021	60	0–7	0.00–13.21	0–0	0.00–0.00
	May 17 to May 23, 2021	69	1–16	1.89–30.19	0–0	0.00–0.00
	May 24 to May 30, 2021	53	0–0	0.00–0.00	0–0	0.00–0.00
Hiroshima						
	Apr 5 to Apr 11, 2021	82	0–2	0.00–2.50	0–0	0.00–0.00
	Apr 12 to Apr 18, 2021	78	0–0	0.00–0.00	0–1	0.00–1.27
	Apr 19 to Apr 25, 2021	87	0–9	0.00–11.54	0–0	0.00–0.00
	Apr 26 to May 2, 2021	71	0–0	0.00–0.00	0–6	0.00–7.79
	May 3 to May 9, 2021	98	5–23	6.67–30.67	0–0	0.00–0.00
	May 10 to May 16, 2021	72	0–0	0.00–0.00	0–2	0.00–2.70
	May 17 to May 23, 2021	88	0–16	0.00–22.22	0–0	0.00–0.00
	May 24 to May 30, 2021	71	0–1	0.00–1.43	0–0	0.00–0.00
Yamaguchi						
	Apr 5 to Apr 11, 2021	44	0–0	0.00–0.00	0–0	0.00–0.00
	Apr 12 to Apr 18, 2021	51	0–7	0.00–15.91	0–0	0.00–0.00

	Apr 19 to Apr 25, 2021	46	0–2	0.00–4.55	0–0	0.00–0.00
	Apr 26 to May 2, 2021	55	0–12	0.00–27.91	0–0	0.00–0.00
	May 3 to May 9, 2021	41	0–0	0.00–0.00	0–3	0.00–6.82
	May 10 to May 16, 2021	37	0–0	0.00–0.00	0–7	0.00–15.91
	May 17 to May 23, 2021	47	0–2	0.00–4.44	0–0	0.00–0.00
	May 24 to May 30, 2021	41	0–0	0.00–0.00	0–4	0.00–8.89
Tokushima						
	Apr 5 to Apr 11, 2021	27	0–0	0.00–0.00	0–2	0.00–6.90
	Apr 12 to Apr 18, 2021	28	0–0	0.00–0.00	0–1	0.00–3.45
	Apr 19 to Apr 25, 2021	35	0–7	0.00–25.00	0–0	0.00–0.00
	Apr 26 to May 2, 2021	24	0–0	0.00–0.00	0–3	0.00–11.11
	May 3 to May 9, 2021	26	0–0	0.00–0.00	0–1	0.00–3.70
	May 10 to May 16, 2021	33	0–7	0.00–26.92	0–0	0.00–0.00
	May 17 to May 23, 2021	33	0–9	0.00–37.50	0–0	0.00–0.00
	May 24 to May 30, 2021	20	0–0	0.00–0.00	0–5	0.00–20.00
Kagawa						
	Apr 5 to Apr 11, 2021	40	0–0	0.00–0.00	0–1	0.00–2.44
	Apr 12 to Apr 18, 2021	43	0–1	0.00–2.38	0–0	0.00–0.00
	Apr 19 to Apr 25, 2021	41	0–0	0.00–0.00	0–1	0.00–2.38
	Apr 26 to May 2, 2021	54	0–12	0.00–28.57	0–0	0.00–0.00
	May 3 to May 9, 2021	44	0–2	0.00–4.76	0–0	0.00–0.00
	May 10 to May 16, 2021	42	0–0	0.00–0.00	0–0	0.00–0.00
	May 17 to May 23, 2021	39	0–0	0.00–0.00	0–3	0.00–7.14
	May 24 to May 30, 2021	37	0–0	0.00–0.00	0–4	0.00–9.76
Ehime						
	Apr 5 to Apr 11, 2021	49	0–3	0.00–6.52	0–0	0.00–0.00
	Apr 12 to Apr 18, 2021	43	0–0	0.00–0.00	0–2	0.00–4.44
	Apr 19 to Apr 25, 2021	51	0–7	0.00–15.91	0–0	0.00–0.00
	Apr 26 to May 2, 2021	52	0–9	0.00–20.93	0–0	0.00–0.00
	May 3 to May 9, 2021	52	0–10	0.00–23.81	0–0	0.00–0.00
	May 10 to May 16, 2021	45	0–3	0.00–7.14	0–0	0.00–0.00
	May 17 to May 23, 2021	35	0–0	0.00–0.00	0–5	0.00–12.50
	May 24 to May 30, 2021	41	0–1	0.00–2.50	0–0	0.00–0.00
Kochi						
	Apr 5 to Apr 11, 2021	23	1–9	7.14–64.29	0–0	0.00–0.00
	Apr 12 to Apr 18, 2021	27	4–12	26.67–80.00	0–0	0.00–0.00
	Apr 19 to Apr 25, 2021	18	0–3	0.00–20.00	0–0	0.00–0.00
	Apr 26 to May 2, 2021	26	4–12	28.57–85.71	0–0	0.00–0.00
	May 3 to May 9, 2021	20	0–5	0.00–33.33	0–0	0.00–0.00
	May 10 to May 16, 2021	18	0–3	0.00–20.00	0–0	0.00–0.00
	May 17 to May 23, 2021	19	0–4	0.00–26.67	0–0	0.00–0.00
	May 24 to May 30, 2021	18	0–3	0.00–20.00	0–0	0.00–0.00
Fukuoka						
	Apr 5 to Apr 11, 2021	102	0–0	0.00–0.00	0–4	0.00–3.77
	Apr 12 to Apr 18, 2021	102	0–0	0.00–0.00	0–2	0.00–1.92
	Apr 19 to Apr 25, 2021	110	0–6	0.00–5.77	0–0	0.00–0.00

	Apr 26 to May 2, 2021	118	0–17	0.00–16.83	0–0	0.00–0.00
	May 3 to May 9, 2021	104	0–6	0.00–6.12	0–0	0.00–0.00
	May 10 to May 16, 2021	116	0–18	0.00–18.37	0–0	0.00–0.00
	May 17 to May 23, 2021	99	0–2	0.00–2.06	0–0	0.00–0.00
	May 24 to May 30, 2021	112	0–15	0.00–15.46	0–0	0.00–0.00
Saga						
	Apr 5 to Apr 11, 2021	24	0–0	0.00–0.00	0–0	0.00–0.00
	Apr 12 to Apr 18, 2021	29	0–6	0.00–26.09	0–0	0.00–0.00
	Apr 19 to Apr 25, 2021	25	0–2	0.00–8.70	0–0	0.00–0.00
	Apr 26 to May 2, 2021	17	0–0	0.00–0.00	0–5	0.00–22.73
	May 3 to May 9, 2021	42	11–20	50.00–90.91	0–0	0.00–0.00
	May 10 to May 16, 2021	24	0–2	0.00–9.09	0–0	0.00–0.00
	May 17 to May 23, 2021	22	0–0	0.00–0.00	0–0	0.00–0.00
	May 24 to May 30, 2021	18	0–0	0.00–0.00	0–3	0.00–14.29
Nagasaki						
	Apr 5 to Apr 11, 2021	44	0–6	0.00–15.79	0–0	0.00–0.00
	Apr 12 to Apr 18, 2021	34	0–0	0.00–0.00	0–4	0.00–10.53
	Apr 19 to Apr 25, 2021	49	0–11	0.00–28.95	0–0	0.00–0.00
	Apr 26 to May 2, 2021	35	0–0	0.00–0.00	0–3	0.00–7.89
	May 3 to May 9, 2021	52	2–15	5.41–40.54	0–0	0.00–0.00
	May 10 to May 16, 2021	53	4–16	10.81–43.24	0–0	0.00–0.00
	May 17 to May 23, 2021	24	0–0	0.00–0.00	0–12	0.00–33.33
	May 24 to May 30, 2021	48	0–12	0.00–33.33	0–0	0.00–0.00
Kumamoto						
	Apr 5 to Apr 11, 2021	64	2–16	4.17–33.33	0–0	0.00–0.00
	Apr 12 to Apr 18, 2021	62	2–16	4.35–34.78	0–0	0.00–0.00
	Apr 19 to Apr 25, 2021	51	0–4	0.00–8.51	0–0	0.00–0.00
	Apr 26 to May 2, 2021	65	3–17	6.25–35.42	0–0	0.00–0.00
	May 3 to May 9, 2021	66	4–18	8.33–37.50	0–0	0.00–0.00
	May 10 to May 16, 2021	58	0–10	0.00–20.83	0–0	0.00–0.00
	May 17 to May 23, 2021	49	0–1	0.00–2.08	0–0	0.00–0.00
	May 24 to May 30, 2021	65	4–17	8.33–35.42	0–0	0.00–0.00
Oita						
	Apr 5 to Apr 11, 2021	44	0–0	0.00–0.00	0–6	0.00–12.00
	Apr 12 to Apr 18, 2021	71	6–20	11.76–39.22	0–0	0.00–0.00
	Apr 19 to Apr 25, 2021	66	2–16	4.00–32.00	0–0	0.00–0.00
	Apr 26 to May 2, 2021	59	0–10	0.00–20.41	0–0	0.00–0.00
	May 3 to May 9, 2021	50	0–1	0.00–2.04	0–0	0.00–0.00
	May 10 to May 16, 2021	52	0–2	0.00–4.00	0–0	0.00–0.00
	May 17 to May 23, 2021	36	0–0	0.00–0.00	0–14	0.00–28.00
	May 24 to May 30, 2021	41	0–0	0.00–0.00	0–8	0.00–16.33
Miyazaki						
	Apr 5 to Apr 11, 2021	31	0–0	0.00–0.00	0–9	0.00–22.50
	Apr 12 to Apr 18, 2021	40	0–1	0.00–2.56	0–0	0.00–0.00
	Apr 19 to Apr 25, 2021	54	3–15	7.69–38.46	0–0	0.00–0.00
	Apr 26 to May 2, 2021	38	0–0	0.00–0.00	0–0	0.00–0.00

	May 3 to May 9, 2021	50	0–12	0.00–31.58	0–0	0.00–0.00
	May 10 to May 16, 2021	31	0–0	0.00–0.00	0–7	0.00–18.42
	May 17 to May 23, 2021	46	0–7	0.00–17.95	0–0	0.00–0.00
	May 24 to May 30, 2021	40	0–2	0.00–5.26	0–0	0.00–0.00
Kagoshima						
	Apr 5 to Apr 11, 2021	61	0–9	0.00–17.31	0–0	0.00–0.00
	Apr 12 to Apr 18, 2021	65	0–13	0.00–25.00	0–0	0.00–0.00
	Apr 19 to Apr 25, 2021	72	6–22	12.00–44.00	0–0	0.00–0.00
	Apr 26 to May 2, 2021	56	0–6	0.00–12.00	0–0	0.00–0.00
	May 3 to May 9, 2021	42	0–0	0.00–0.00	0–7	0.00–14.29
	May 10 to May 16, 2021	50	0–0	0.00–0.00	0–0	0.00–0.00
	May 17 to May 23, 2021	55	0–5	0.00–10.00	0–0	0.00–0.00
	May 24 to May 30, 2021	59	0–11	0.00–22.92	0–0	0.00–0.00
Okinawa						
	Apr 5 to Apr 11, 2021	37	0–6	0.00–19.35	0–0	0.00–0.00
	Apr 12 to Apr 18, 2021	33	0–2	0.00–6.45	0–0	0.00–0.00
	Apr 19 to Apr 25, 2021	27	0–0	0.00–0.00	0–3	0.00–10.00
	Apr 26 to May 2, 2021	34	0–4	0.00–13.33	0–0	0.00–0.00
	May 3 to May 9, 2021	27	0–0	0.00–0.00	0–3	0.00–10.00
	May 10 to May 16, 2021	37	0–7	0.00–23.33	0–0	0.00–0.00
	May 17 to May 23, 2021	29	0–0	0.00–0.00	0–1	0.00–3.33
	May 24 to May 30, 2021	41	0–11	0.00–36.67	0–0	0.00–0.00

Table A.4: Weekly number of observed and excess/exiguous deaths in Japan and 47 prefectures for non-COVID-19-related deaths at homes from January 2020 through May 2021.

Prefecture	Week	Observed	Excess deaths	Percent excess	Exiguous deaths	Percent exiguous
Japan						
	Apr 5 to Apr 11, 2021	4409	0–424	0.00–10.64	0–0	0.00–0.00
	Apr 12 to Apr 18, 2021	4619	67–686	1.70–17.44	0–0	0.00–0.00
	Apr 19 to Apr 25, 2021	4584	100–727	2.59–18.85	0–0	0.00–0.00
	Apr 26 to May 2, 2021	4817	436–1042	11.55–27.60	0–0	0.00–0.00
	May 3 to May 9, 2021	4484	148–759	3.97–20.38	0–0	0.00–0.00
	May 10 to May 16, 2021	4473	230–847	6.34–23.36	0–0	0.00–0.00
	May 17 to May 23, 2021	4147	0–562	0.00–15.68	0–0	0.00–0.00
	May 24 to May 30, 2021	4010	0–443	0.00–12.42	0–0	0.00–0.00
Hokkaido						
	Apr 5 to Apr 11, 2021	159	0–18	0.00–12.77	0–0	0.00–0.00
	Apr 12 to Apr 18, 2021	149	0–10	0.00–7.19	0–0	0.00–0.00
	Apr 19 to Apr 25, 2021	133	0–0	0.00–0.00	0–5	0.00–3.62
	Apr 26 to May 2, 2021	174	6–39	4.44–28.89	0–0	0.00–0.00
	May 3 to May 9, 2021	155	0–20	0.00–14.81	0–0	0.00–0.00
	May 10 to May 16, 2021	151	0–17	0.00–12.69	0–0	0.00–0.00
	May 17 to May 23, 2021	152	0–16	0.00–11.76	0–0	0.00–0.00
	May 24 to May 30, 2021	158	0–24	0.00–17.91	0–0	0.00–0.00
Aomori						
	Apr 5 to Apr 11, 2021	36	0–0	0.00–0.00	0–5	0.00–12.20
	Apr 12 to Apr 18, 2021	40	0–0	0.00–0.00	0–0	0.00–0.00
	Apr 19 to Apr 25, 2021	43	0–4	0.00–10.26	0–0	0.00–0.00
	Apr 26 to May 2, 2021	43	0–5	0.00–13.16	0–0	0.00–0.00
	May 3 to May 9, 2021	31	0–0	0.00–0.00	0–7	0.00–18.42
	May 10 to May 16, 2021	38	0–0	0.00–0.00	0–0	0.00–0.00
	May 17 to May 23, 2021	44	0–5	0.00–12.82	0–0	0.00–0.00
	May 24 to May 30, 2021	38	0–1	0.00–2.70	0–0	0.00–0.00
Iwate						
	Apr 5 to Apr 11, 2021	37	0–0	0.00–0.00	0–2	0.00–5.13
	Apr 12 to Apr 18, 2021	34	0–0	0.00–0.00	0–4	0.00–10.53
	Apr 19 to Apr 25, 2021	32	0–0	0.00–0.00	0–4	0.00–11.11
	Apr 26 to May 2, 2021	42	0–7	0.00–20.00	0–0	0.00–0.00
	May 3 to May 9, 2021	35	0–1	0.00–2.94	0–0	0.00–0.00
	May 10 to May 16, 2021	45	0–13	0.00–40.63	0–0	0.00–0.00
	May 17 to May 23, 2021	33	0–0	0.00–0.00	0–0	0.00–0.00
	May 24 to May 30, 2021	25	0–0	0.00–0.00	0–8	0.00–24.24
Miyagi						
	Apr 5 to Apr 11, 2021	101	9–28	12.33–38.36	0–0	0.00–0.00
	Apr 12 to Apr 18, 2021	93	2–22	2.82–30.99	0–0	0.00–0.00
	Apr 19 to Apr 25, 2021	79	0–11	0.00–16.18	0–0	0.00–0.00
	Apr 26 to May 2, 2021	85	0–17	0.00–25.00	0–0	0.00–0.00
	May 3 to May 9, 2021	75	0–9	0.00–13.64	0–0	0.00–0.00

	May 10 to May 16, 2021	69	0–4	0.00–6.15	0–0	0.00–0.00
	May 17 to May 23, 2021	66	0–3	0.00–4.76	0–0	0.00–0.00
	May 24 to May 30, 2021	69	0–8	0.00–13.11	0–0	0.00–0.00
Akita						
	Apr 5 to Apr 11, 2021	28	0–0	0.00–0.00	0–2	0.00–6.67
	Apr 12 to Apr 18, 2021	32	0–2	0.00–6.67	0–0	0.00–0.00
	Apr 19 to Apr 25, 2021	29	0–1	0.00–3.57	0–0	0.00–0.00
	Apr 26 to May 2, 2021	26	0–0	0.00–0.00	0–0	0.00–0.00
	May 3 to May 9, 2021	32	0–6	0.00–23.08	0–0	0.00–0.00
	May 10 to May 16, 2021	30	0–5	0.00–20.00	0–0	0.00–0.00
	May 17 to May 23, 2021	22	0–0	0.00–0.00	0–4	0.00–15.38
	May 24 to May 30, 2021	23	0–0	0.00–0.00	0–2	0.00–8.00
Yamagata						
	Apr 5 to Apr 11, 2021	26	0–0	0.00–0.00	0–9	0.00–25.71
	Apr 12 to Apr 18, 2021	31	0–0	0.00–0.00	0–3	0.00–8.82
	Apr 19 to Apr 25, 2021	48	1–14	2.94–41.18	0–0	0.00–0.00
	Apr 26 to May 2, 2021	51	5–17	14.71–50.00	0–0	0.00–0.00
	May 3 to May 9, 2021	39	0–5	0.00–14.71	0–0	0.00–0.00
	May 10 to May 16, 2021	51	5–17	14.71–50.00	0–0	0.00–0.00
	May 17 to May 23, 2021	39	0–5	0.00–14.71	0–0	0.00–0.00
	May 24 to May 30, 2021	55	10–23	31.25–71.88	0–0	0.00–0.00
Fukushima						
	Apr 5 to Apr 11, 2021	70	0–5	0.00–7.69	0–0	0.00–0.00
	Apr 12 to Apr 18, 2021	96	14–32	21.88–50.00	0–0	0.00–0.00
	Apr 19 to Apr 25, 2021	66	0–4	0.00–6.45	0–0	0.00–0.00
	Apr 26 to May 2, 2021	89	11–27	17.74–43.55	0–0	0.00–0.00
	May 3 to May 9, 2021	60	0–0	0.00–0.00	0–1	0.00–1.64
	May 10 to May 16, 2021	70	0–10	0.00–16.67	0–0	0.00–0.00
	May 17 to May 23, 2021	69	0–10	0.00–16.95	0–0	0.00–0.00
	May 24 to May 30, 2021	57	0–0	0.00–0.00	0–1	0.00–1.72
Ibaraki						
	Apr 5 to Apr 11, 2021	95	0–10	0.00–11.76	0–0	0.00–0.00
	Apr 12 to Apr 18, 2021	106	1–23	1.20–27.71	0–0	0.00–0.00
	Apr 19 to Apr 25, 2021	95	0–13	0.00–15.85	0–0	0.00–0.00
	Apr 26 to May 2, 2021	94	0–14	0.00–17.50	0–0	0.00–0.00
	May 3 to May 9, 2021	76	0–0	0.00–0.00	0–2	0.00–2.56
	May 10 to May 16, 2021	99	3–24	4.00–32.00	0–0	0.00–0.00
	May 17 to May 23, 2021	65	0–0	0.00–0.00	0–9	0.00–12.16
	May 24 to May 30, 2021	87	0–14	0.00–19.18	0–0	0.00–0.00
Tochigi						
	Apr 5 to Apr 11, 2021	75	0–7	0.00–10.29	0–0	0.00–0.00
	Apr 12 to Apr 18, 2021	74	0–8	0.00–12.12	0–0	0.00–0.00
	Apr 19 to Apr 25, 2021	84	0–20	0.00–31.25	0–0	0.00–0.00
	Apr 26 to May 2, 2021	81	0–19	0.00–30.65	0–0	0.00–0.00
	May 3 to May 9, 2021	76	0–14	0.00–22.58	0–0	0.00–0.00
	May 10 to May 16, 2021	68	0–9	0.00–15.25	0–0	0.00–0.00

	May 17 to May 23, 2021	73	0–14	0.00–23.73	0–0	0.00–0.00
	May 24 to May 30, 2021	77	0–17	0.00–28.33	0–0	0.00–0.00
Gunma						
	Apr 5 to Apr 11, 2021	61	0–1	0.00–1.67	0–0	0.00–0.00
	Apr 12 to Apr 18, 2021	72	0–13	0.00–22.03	0–0	0.00–0.00
	Apr 19 to Apr 25, 2021	74	0–14	0.00–23.33	0–0	0.00–0.00
	Apr 26 to May 2, 2021	73	0–14	0.00–23.73	0–0	0.00–0.00
	May 3 to May 9, 2021	62	0–3	0.00–5.08	0–0	0.00–0.00
	May 10 to May 16, 2021	57	0–0	0.00–0.00	0–1	0.00–1.72
	May 17 to May 23, 2021	48	0–0	0.00–0.00	0–9	0.00–15.79
	May 24 to May 30, 2021	57	0–0	0.00–0.00	0–1	0.00–1.72
Saitama						
	Apr 5 to Apr 11, 2021	224	0–1	0.00–0.45	0–0	0.00–0.00
	Apr 12 to Apr 18, 2021	257	0–37	0.00–16.82	0–0	0.00–0.00
	Apr 19 to Apr 25, 2021	239	0–21	0.00–9.63	0–0	0.00–0.00
	Apr 26 to May 2, 2021	284	14–68	6.48–31.48	0–0	0.00–0.00
	May 3 to May 9, 2021	235	0–20	0.00–9.30	0–0	0.00–0.00
	May 10 to May 16, 2021	246	0–36	0.00–17.14	0–0	0.00–0.00
	May 17 to May 23, 2021	247	0–39	0.00–18.75	0–0	0.00–0.00
	May 24 to May 30, 2021	197	0–0	0.00–0.00	0–7	0.00–3.43
Chiba						
	Apr 5 to Apr 11, 2021	220	0–10	0.00–4.76	0–0	0.00–0.00
	Apr 12 to Apr 18, 2021	248	0–38	0.00–18.10	0–0	0.00–0.00
	Apr 19 to Apr 25, 2021	238	0–32	0.00–15.53	0–0	0.00–0.00
	Apr 26 to May 2, 2021	229	0–29	0.00–14.50	0–0	0.00–0.00
	May 3 to May 9, 2021	236	0–40	0.00–20.41	0–0	0.00–0.00
	May 10 to May 16, 2021	229	0–39	0.00–20.53	0–0	0.00–0.00
	May 17 to May 23, 2021	202	0–14	0.00–7.45	0–0	0.00–0.00
	May 24 to May 30, 2021	183	0–0	0.00–0.00	0–1	0.00–0.54
Tokyo						
	Apr 5 to Apr 11, 2021	518	0–16	0.00–3.19	0–0	0.00–0.00
	Apr 12 to Apr 18, 2021	581	0–83	0.00–16.67	0–0	0.00–0.00
	Apr 19 to Apr 25, 2021	574	0–85	0.00–17.38	0–0	0.00–0.00
	Apr 26 to May 2, 2021	578	7–99	1.46–20.67	0–0	0.00–0.00
	May 3 to May 9, 2021	500	0–26	0.00–5.49	0–0	0.00–0.00
	May 10 to May 16, 2021	487	0–17	0.00–3.62	0–0	0.00–0.00
	May 17 to May 23, 2021	501	0–30	0.00–6.37	0–0	0.00–0.00
	May 24 to May 30, 2021	466	0–8	0.00–1.75	0–0	0.00–0.00
Kanagawa						
	Apr 5 to Apr 11, 2021	351	0–21	0.00–6.36	0–0	0.00–0.00
	Apr 12 to Apr 18, 2021	363	0–34	0.00–10.33	0–0	0.00–0.00
	Apr 19 to Apr 25, 2021	369	0–39	0.00–11.82	0–0	0.00–0.00
	Apr 26 to May 2, 2021	416	28–91	8.62–28.00	0–0	0.00–0.00
	May 3 to May 9, 2021	378	0–59	0.00–18.50	0–0	0.00–0.00
	May 10 to May 16, 2021	385	7–68	2.21–21.45	0–0	0.00–0.00
	May 17 to May 23, 2021	329	0–15	0.00–4.78	0–0	0.00–0.00

	May 24 to May 30, 2021	329	0–19	0.00–6.13	0–0	0.00–0.00
Niigata						
	Apr 5 to Apr 11, 2021	68	0–9	0.00–15.25	0–0	0.00–0.00
	Apr 12 to Apr 18, 2021	68	0–11	0.00–19.30	0–0	0.00–0.00
	Apr 19 to Apr 25, 2021	53	0–0	0.00–0.00	0–3	0.00–5.36
	Apr 26 to May 2, 2021	69	0–14	0.00–25.45	0–0	0.00–0.00
	May 3 to May 9, 2021	66	0–12	0.00–22.22	0–0	0.00–0.00
	May 10 to May 16, 2021	53	0–1	0.00–1.92	0–0	0.00–0.00
	May 17 to May 23, 2021	45	0–0	0.00–0.00	0–6	0.00–11.76
	May 24 to May 30, 2021	58	0–9	0.00–18.37	0–0	0.00–0.00
Toyama						
	Apr 5 to Apr 11, 2021	44	1–13	3.23–41.94	0–0	0.00–0.00
	Apr 12 to Apr 18, 2021	34	0–3	0.00–9.68	0–0	0.00–0.00
	Apr 19 to Apr 25, 2021	34	0–3	0.00–9.68	0–0	0.00–0.00
	Apr 26 to May 2, 2021	40	0–10	0.00–33.33	0–0	0.00–0.00
	May 3 to May 9, 2021	43	2–13	6.67–43.33	0–0	0.00–0.00
	May 10 to May 16, 2021	38	0–8	0.00–26.67	0–0	0.00–0.00
	May 17 to May 23, 2021	26	0–0	0.00–0.00	0–4	0.00–13.33
	May 24 to May 30, 2021	39	0–10	0.00–34.48	0–0	0.00–0.00
Ishikawa						
	Apr 5 to Apr 11, 2021	45	8–19	30.77–73.08	0–0	0.00–0.00
	Apr 12 to Apr 18, 2021	46	8–20	30.77–76.92	0–0	0.00–0.00
	Apr 19 to Apr 25, 2021	38	0–12	0.00–46.15	0–0	0.00–0.00
	Apr 26 to May 2, 2021	32	0–7	0.00–28.00	0–0	0.00–0.00
	May 3 to May 9, 2021	41	4–16	16.00–64.00	0–0	0.00–0.00
	May 10 to May 16, 2021	28	0–2	0.00–7.69	0–0	0.00–0.00
	May 17 to May 23, 2021	31	0–6	0.00–24.00	0–0	0.00–0.00
	May 24 to May 30, 2021	28	0–3	0.00–12.00	0–0	0.00–0.00
Fukui						
	Apr 5 to Apr 11, 2021	22	0–0	0.00–0.00	0–3	0.00–12.00
	Apr 12 to Apr 18, 2021	29	0–5	0.00–20.83	0–0	0.00–0.00
	Apr 19 to Apr 25, 2021	22	0–0	0.00–0.00	0–1	0.00–4.35
	Apr 26 to May 2, 2021	28	0–6	0.00–27.27	0–0	0.00–0.00
	May 3 to May 9, 2021	37	4–15	18.18–68.18	0–0	0.00–0.00
	May 10 to May 16, 2021	30	0–9	0.00–42.86	0–0	0.00–0.00
	May 17 to May 23, 2021	21	0–0	0.00–0.00	0–0	0.00–0.00
	May 24 to May 30, 2021	24	0–3	0.00–14.29	0–0	0.00–0.00
Yamanashi						
	Apr 5 to Apr 11, 2021	25	0–0	0.00–0.00	0–4	0.00–13.79
	Apr 12 to Apr 18, 2021	26	0–0	0.00–0.00	0–3	0.00–10.34
	Apr 19 to Apr 25, 2021	23	0–0	0.00–0.00	0–5	0.00–17.86
	Apr 26 to May 2, 2021	38	0–11	0.00–40.74	0–0	0.00–0.00
	May 3 to May 9, 2021	36	0–10	0.00–38.46	0–0	0.00–0.00
	May 10 to May 16, 2021	27	0–1	0.00–3.85	0–0	0.00–0.00
	May 17 to May 23, 2021	44	7–18	26.92–69.23	0–0	0.00–0.00
	May 24 to May 30, 2021	32	0–7	0.00–28.00	0–0	0.00–0.00

Nagano						
	Apr 5 to Apr 11, 2021	83	0–16	0.00–23.88	0–0	0.00–0.00
	Apr 12 to Apr 18, 2021	84	0–18	0.00–27.27	0–0	0.00–0.00
	Apr 19 to Apr 25, 2021	78	0–12	0.00–18.18	0–0	0.00–0.00
	Apr 26 to May 2, 2021	72	0–8	0.00–12.50	0–0	0.00–0.00
	May 3 to May 9, 2021	67	0–4	0.00–6.35	0–0	0.00–0.00
	May 10 to May 16, 2021	56	0–0	0.00–0.00	0–5	0.00–8.20
	May 17 to May 23, 2021	79	2–20	3.39–33.90	0–0	0.00–0.00
	May 24 to May 30, 2021	64	0–5	0.00–8.47	0–0	0.00–0.00
Gifu						
	Apr 5 to Apr 11, 2021	74	0–2	0.00–2.78	0–0	0.00–0.00
	Apr 12 to Apr 18, 2021	94	5–24	7.14–34.29	0–0	0.00–0.00
	Apr 19 to Apr 25, 2021	88	0–19	0.00–27.54	0–0	0.00–0.00
	Apr 26 to May 2, 2021	67	0–0	0.00–0.00	0–1	0.00–1.47
	May 3 to May 9, 2021	64	0–0	0.00–0.00	0–3	0.00–4.48
	May 10 to May 16, 2021	80	0–14	0.00–21.21	0–0	0.00–0.00
	May 17 to May 23, 2021	87	6–24	9.52–38.10	0–0	0.00–0.00
	May 24 to May 30, 2021	66	0–4	0.00–6.45	0–0	0.00–0.00
Shizuoka						
	Apr 5 to Apr 11, 2021	164	3–34	2.31–26.15	0–0	0.00–0.00
	Apr 12 to Apr 18, 2021	122	0–0	0.00–0.00	0–4	0.00–3.17
	Apr 19 to Apr 25, 2021	129	0–4	0.00–3.20	0–0	0.00–0.00
	Apr 26 to May 2, 2021	148	0–26	0.00–21.31	0–0	0.00–0.00
	May 3 to May 9, 2021	140	0–21	0.00–17.65	0–0	0.00–0.00
	May 10 to May 16, 2021	137	0–20	0.00–17.09	0–0	0.00–0.00
	May 17 to May 23, 2021	139	0–24	0.00–20.87	0–0	0.00–0.00
	May 24 to May 30, 2021	113	0–0	0.00–0.00	0–3	0.00–2.59
Aichi						
	Apr 5 to Apr 11, 2021	210	0–6	0.00–2.94	0–0	0.00–0.00
	Apr 12 to Apr 18, 2021	214	0–11	0.00–5.42	0–0	0.00–0.00
	Apr 19 to Apr 25, 2021	234	0–39	0.00–20.00	0–0	0.00–0.00
	Apr 26 to May 2, 2021	239	0–46	0.00–23.83	0–0	0.00–0.00
	May 3 to May 9, 2021	206	0–12	0.00–6.19	0–0	0.00–0.00
	May 10 to May 16, 2021	262	23–72	12.11–37.89	0–0	0.00–0.00
	May 17 to May 23, 2021	202	0–16	0.00–8.60	0–0	0.00–0.00
	May 24 to May 30, 2021	198	0–18	0.00–10.00	0–0	0.00–0.00
Mie						
	Apr 5 to Apr 11, 2021	69	0–10	0.00–16.95	0–0	0.00–0.00
	Apr 12 to Apr 18, 2021	70	0–13	0.00–22.81	0–0	0.00–0.00
	Apr 19 to Apr 25, 2021	68	0–11	0.00–19.30	0–0	0.00–0.00
	Apr 26 to May 2, 2021	68	0–13	0.00–23.64	0–0	0.00–0.00
	May 3 to May 9, 2021	59	0–4	0.00–7.27	0–0	0.00–0.00
	May 10 to May 16, 2021	64	0–10	0.00–18.52	0–0	0.00–0.00
	May 17 to May 23, 2021	69	1–17	1.92–32.69	0–0	0.00–0.00
	May 24 to May 30, 2021	54	0–2	0.00–3.85	0–0	0.00–0.00
Shiga						

	Apr 5 to Apr 11, 2021	40	0–2	0.00–5.26	0–0	0.00–0.00
	Apr 12 to Apr 18, 2021	49	0–11	0.00–28.95	0–0	0.00–0.00
	Apr 19 to Apr 25, 2021	51	0–14	0.00–37.84	0–0	0.00–0.00
	Apr 26 to May 2, 2021	48	0–13	0.00–37.14	0–0	0.00–0.00
	May 3 to May 9, 2021	46	0–12	0.00–35.29	0–0	0.00–0.00
	May 10 to May 16, 2021	46	0–12	0.00–35.29	0–0	0.00–0.00
	May 17 to May 23, 2021	37	0–4	0.00–12.12	0–0	0.00–0.00
	May 24 to May 30, 2021	47	0–14	0.00–42.42	0–0	0.00–0.00
Kyoto						
	Apr 5 to Apr 11, 2021	103	0–22	0.00–27.16	0–0	0.00–0.00
	Apr 12 to Apr 18, 2021	94	0–12	0.00–14.63	0–0	0.00–0.00
	Apr 19 to Apr 25, 2021	113	6–34	7.59–43.04	0–0	0.00–0.00
	Apr 26 to May 2, 2021	98	0–21	0.00–27.27	0–0	0.00–0.00
	May 3 to May 9, 2021	105	0–27	0.00–34.62	0–0	0.00–0.00
	May 10 to May 16, 2021	101	0–24	0.00–31.17	0–0	0.00–0.00
	May 17 to May 23, 2021	104	1–28	1.32–36.84	0–0	0.00–0.00
	May 24 to May 30, 2021	95	0–20	0.00–26.67	0–0	0.00–0.00
Osaka						
	Apr 5 to Apr 11, 2021	358	0–29	0.00–8.81	0–0	0.00–0.00
	Apr 12 to Apr 18, 2021	359	0–38	0.00–11.84	0–0	0.00–0.00
	Apr 19 to Apr 25, 2021	373	0–61	0.00–19.55	0–0	0.00–0.00
	Apr 26 to May 2, 2021	419	54–117	17.88–38.74	0–0	0.00–0.00
	May 3 to May 9, 2021	399	34–98	11.30–32.56	0–0	0.00–0.00
	May 10 to May 16, 2021	377	15–80	5.05–26.94	0–0	0.00–0.00
	May 17 to May 23, 2021	360	4–67	1.37–22.87	0–0	0.00–0.00
	May 24 to May 30, 2021	332	0–46	0.00–16.08	0–0	0.00–0.00
Hyogo						
	Apr 5 to Apr 11, 2021	242	1–44	0.51–22.22	0–0	0.00–0.00
	Apr 12 to Apr 18, 2021	257	16–60	8.12–30.46	0–0	0.00–0.00
	Apr 19 to Apr 25, 2021	253	18–62	9.42–32.46	0–0	0.00–0.00
	Apr 26 to May 2, 2021	281	53–95	28.49–51.08	0–0	0.00–0.00
	May 3 to May 9, 2021	257	30–73	16.30–39.67	0–0	0.00–0.00
	May 10 to May 16, 2021	264	40–82	21.98–45.05	0–0	0.00–0.00
	May 17 to May 23, 2021	215	0–36	0.00–20.11	0–0	0.00–0.00
	May 24 to May 30, 2021	257	38–80	21.47–45.20	0–0	0.00–0.00
Nara						
	Apr 5 to Apr 11, 2021	57	0–8	0.00–16.33	0–0	0.00–0.00
	Apr 12 to Apr 18, 2021	60	0–11	0.00–22.45	0–0	0.00–0.00
	Apr 19 to Apr 25, 2021	44	0–0	0.00–0.00	0–3	0.00–6.38
	Apr 26 to May 2, 2021	59	0–13	0.00–28.26	0–0	0.00–0.00
	May 3 to May 9, 2021	50	0–4	0.00–8.70	0–0	0.00–0.00
	May 10 to May 16, 2021	57	0–12	0.00–26.67	0–0	0.00–0.00
	May 17 to May 23, 2021	39	0–0	0.00–0.00	0–6	0.00–13.33
	May 24 to May 30, 2021	43	0–0	0.00–0.00	0–0	0.00–0.00
Wakayama						
	Apr 5 to Apr 11, 2021	32	0–0	0.00–0.00	0–1	0.00–3.03

Apr 12 to Apr 18, 2021	39	0–7	0.00–21.88	0–0	0.00–0.00
Apr 19 to Apr 25, 2021	42	0–11	0.00–35.48	0–0	0.00–0.00
Apr 26 to May 2, 2021	36	0–5	0.00–16.13	0–0	0.00–0.00
May 3 to May 9, 2021	46	2–16	6.67–53.33	0–0	0.00–0.00
May 10 to May 16, 2021	41	0–12	0.00–41.38	0–0	0.00–0.00
May 17 to May 23, 2021	37	0–6	0.00–19.35	0–0	0.00–0.00
May 24 to May 30, 2021	28	0–0	0.00–0.00	0–3	0.00–9.68
Tottori					
Apr 5 to Apr 11, 2021	20	0–3	0.00–17.65	0–0	0.00–0.00
Apr 12 to Apr 18, 2021	28	2–11	11.76–64.71	0–0	0.00–0.00
Apr 19 to Apr 25, 2021	23	0–6	0.00–35.29	0–0	0.00–0.00
Apr 26 to May 2, 2021	17	0–0	0.00–0.00	0–0	0.00–0.00
May 3 to May 9, 2021	22	0–5	0.00–29.41	0–0	0.00–0.00
May 10 to May 16, 2021	25	0–9	0.00–56.25	0–0	0.00–0.00
May 17 to May 23, 2021	24	0–8	0.00–50.00	0–0	0.00–0.00
May 24 to May 30, 2021	21	0–5	0.00–31.25	0–0	0.00–0.00
Shimane					
Apr 5 to Apr 11, 2021	22	0–1	0.00–4.76	0–0	0.00–0.00
Apr 12 to Apr 18, 2021	34	3–13	14.29–61.90	0–0	0.00–0.00
Apr 19 to Apr 25, 2021	17	0–0	0.00–0.00	0–4	0.00–19.05
Apr 26 to May 2, 2021	20	0–0	0.00–0.00	0–0	0.00–0.00
May 3 to May 9, 2021	29	0–10	0.00–52.63	0–0	0.00–0.00
May 10 to May 16, 2021	17	0–0	0.00–0.00	0–2	0.00–10.53
May 17 to May 23, 2021	16	0–0	0.00–0.00	0–3	0.00–15.79
May 24 to May 30, 2021	14	0–0	0.00–0.00	0–5	0.00–26.32
Okayama					
Apr 5 to Apr 11, 2021	56	0–5	0.00–9.80	0–0	0.00–0.00
Apr 12 to Apr 18, 2021	45	0–0	0.00–0.00	0–6	0.00–11.76
Apr 19 to Apr 25, 2021	59	0–8	0.00–15.69	0–0	0.00–0.00
Apr 26 to May 2, 2021	67	0–17	0.00–34.00	0–0	0.00–0.00
May 3 to May 9, 2021	65	0–15	0.00–30.00	0–0	0.00–0.00
May 10 to May 16, 2021	80	11–31	22.45–63.27	0–0	0.00–0.00
May 17 to May 23, 2021	53	0–5	0.00–10.42	0–0	0.00–0.00
May 24 to May 30, 2021	74	8–28	17.39–60.87	0–0	0.00–0.00
Hiroshima					
Apr 5 to Apr 11, 2021	85	0–11	0.00–14.86	0–0	0.00–0.00
Apr 12 to Apr 18, 2021	91	0–18	0.00–24.66	0–0	0.00–0.00
Apr 19 to Apr 25, 2021	86	0–15	0.00–21.13	0–0	0.00–0.00
Apr 26 to May 2, 2021	86	0–17	0.00–24.64	0–0	0.00–0.00
May 3 to May 9, 2021	92	0–23	0.00–33.33	0–0	0.00–0.00
May 10 to May 16, 2021	74	0–6	0.00–8.82	0–0	0.00–0.00
May 17 to May 23, 2021	81	0–13	0.00–19.12	0–0	0.00–0.00
May 24 to May 30, 2021	66	0–0	0.00–0.00	0–2	0.00–2.94
Yamaguchi					
Apr 5 to Apr 11, 2021	42	0–0	0.00–0.00	0–2	0.00–4.55
Apr 12 to Apr 18, 2021	45	0–2	0.00–4.65	0–0	0.00–0.00

	Apr 19 to Apr 25, 2021	56	0–15	0.00–36.59	0–0	0.00–0.00
	Apr 26 to May 2, 2021	44	0–4	0.00–10.00	0–0	0.00–0.00
	May 3 to May 9, 2021	43	0–3	0.00–7.50	0–0	0.00–0.00
	May 10 to May 16, 2021	39	0–1	0.00–2.63	0–0	0.00–0.00
	May 17 to May 23, 2021	54	2–17	5.41–45.95	0–0	0.00–0.00
	May 24 to May 30, 2021	38	0–2	0.00–5.56	0–0	0.00–0.00
Tokushima						
	Apr 5 to Apr 11, 2021	26	0–5	0.00–23.81	0–0	0.00–0.00
	Apr 12 to Apr 18, 2021	29	0–10	0.00–52.63	0–0	0.00–0.00
	Apr 19 to Apr 25, 2021	26	0–8	0.00–44.44	0–0	0.00–0.00
	Apr 26 to May 2, 2021	33	4–14	21.05–73.68	0–0	0.00–0.00
	May 3 to May 9, 2021	30	3–12	16.67–66.67	0–0	0.00–0.00
	May 10 to May 16, 2021	28	1–11	5.88–64.71	0–0	0.00–0.00
	May 17 to May 23, 2021	19	0–2	0.00–11.76	0–0	0.00–0.00
	May 24 to May 30, 2021	16	0–0	0.00–0.00	0–1	0.00–5.88
Kagawa						
	Apr 5 to Apr 11, 2021	35	0–1	0.00–2.94	0–0	0.00–0.00
	Apr 12 to Apr 18, 2021	39	0–5	0.00–14.71	0–0	0.00–0.00
	Apr 19 to Apr 25, 2021	45	0–12	0.00–36.36	0–0	0.00–0.00
	Apr 26 to May 2, 2021	43	0–12	0.00–38.71	0–0	0.00–0.00
	May 3 to May 9, 2021	33	0–2	0.00–6.45	0–0	0.00–0.00
	May 10 to May 16, 2021	30	0–0	0.00–0.00	0–0	0.00–0.00
	May 17 to May 23, 2021	39	0–9	0.00–30.00	0–0	0.00–0.00
	May 24 to May 30, 2021	33	0–3	0.00–10.00	0–0	0.00–0.00
Ehime						
	Apr 5 to Apr 11, 2021	57	0–5	0.00–9.62	0–0	0.00–0.00
	Apr 12 to Apr 18, 2021	70	4–20	8.00–40.00	0–0	0.00–0.00
	Apr 19 to Apr 25, 2021	67	2–18	4.08–36.73	0–0	0.00–0.00
	Apr 26 to May 2, 2021	50	0–2	0.00–4.17	0–0	0.00–0.00
	May 3 to May 9, 2021	68	5–21	10.64–44.68	0–0	0.00–0.00
	May 10 to May 16, 2021	50	0–4	0.00–8.70	0–0	0.00–0.00
	May 17 to May 23, 2021	36	0–0	0.00–0.00	0–10	0.00–21.74
	May 24 to May 30, 2021	46	0–1	0.00–2.22	0–0	0.00–0.00
Kochi						
	Apr 5 to Apr 11, 2021	23	0–2	0.00–9.52	0–0	0.00–0.00
	Apr 12 to Apr 18, 2021	22	0–2	0.00–10.00	0–0	0.00–0.00
	Apr 19 to Apr 25, 2021	29	0–9	0.00–45.00	0–0	0.00–0.00
	Apr 26 to May 2, 2021	22	0–3	0.00–15.79	0–0	0.00–0.00
	May 3 to May 9, 2021	21	0–2	0.00–10.53	0–0	0.00–0.00
	May 10 to May 16, 2021	16	0–0	0.00–0.00	0–3	0.00–15.79
	May 17 to May 23, 2021	19	0–0	0.00–0.00	0–0	0.00–0.00
	May 24 to May 30, 2021	26	0–7	0.00–36.84	0–0	0.00–0.00
Fukuoka						
	Apr 5 to Apr 11, 2021	145	0–19	0.00–15.08	0–0	0.00–0.00
	Apr 12 to Apr 18, 2021	141	0–16	0.00–12.80	0–0	0.00–0.00
	Apr 19 to Apr 25, 2021	130	0–6	0.00–4.84	0–0	0.00–0.00

	Apr 26 to May 2, 2021	159	7–37	5.74–30.33	0–0	0.00–0.00
	May 3 to May 9, 2021	146	0–25	0.00–20.66	0–0	0.00–0.00
	May 10 to May 16, 2021	139	0–19	0.00–15.83	0–0	0.00–0.00
	May 17 to May 23, 2021	130	0–11	0.00–9.24	0–0	0.00–0.00
	May 24 to May 30, 2021	136	0–19	0.00–16.24	0–0	0.00–0.00
Saga						
	Apr 5 to Apr 11, 2021	17	0–0	0.00–0.00	0–3	0.00–15.00
	Apr 12 to Apr 18, 2021	25	0–5	0.00–25.00	0–0	0.00–0.00
	Apr 19 to Apr 25, 2021	26	0–6	0.00–30.00	0–0	0.00–0.00
	Apr 26 to May 2, 2021	17	0–0	0.00–0.00	0–2	0.00–10.53
	May 3 to May 9, 2021	30	2–12	11.11–66.67	0–0	0.00–0.00
	May 10 to May 16, 2021	12	0–0	0.00–0.00	0–6	0.00–33.33
	May 17 to May 23, 2021	29	2–11	11.11–61.11	0–0	0.00–0.00
	May 24 to May 30, 2021	16	0–0	0.00–0.00	0–1	0.00–5.88
Nagasaki						
	Apr 5 to Apr 11, 2021	45	0–6	0.00–15.38	0–0	0.00–0.00
	Apr 12 to Apr 18, 2021	45	0–6	0.00–15.38	0–0	0.00–0.00
	Apr 19 to Apr 25, 2021	61	8–23	21.05–60.53	0–0	0.00–0.00
	Apr 26 to May 2, 2021	37	0–1	0.00–2.78	0–0	0.00–0.00
	May 3 to May 9, 2021	46	0–11	0.00–31.43	0–0	0.00–0.00
	May 10 to May 16, 2021	49	0–13	0.00–36.11	0–0	0.00–0.00
	May 17 to May 23, 2021	35	0–0	0.00–0.00	0–0	0.00–0.00
	May 24 to May 30, 2021	48	1–14	2.94–41.18	0–0	0.00–0.00
Kumamoto						
	Apr 5 to Apr 11, 2021	50	0–6	0.00–13.64	0–0	0.00–0.00
	Apr 12 to Apr 18, 2021	55	0–13	0.00–30.95	0–0	0.00–0.00
	Apr 19 to Apr 25, 2021	57	0–15	0.00–35.71	0–0	0.00–0.00
	Apr 26 to May 2, 2021	59	4–19	10.00–47.50	0–0	0.00–0.00
	May 3 to May 9, 2021	52	0–12	0.00–30.00	0–0	0.00–0.00
	May 10 to May 16, 2021	54	0–15	0.00–38.46	0–0	0.00–0.00
	May 17 to May 23, 2021	38	0–0	0.00–0.00	0–0	0.00–0.00
	May 24 to May 30, 2021	48	0–11	0.00–29.73	0–0	0.00–0.00
Oita						
	Apr 5 to Apr 11, 2021	30	0–6	0.00–25.00	0–0	0.00–0.00
	Apr 12 to Apr 18, 2021	25	0–2	0.00–8.70	0–0	0.00–0.00
	Apr 19 to Apr 25, 2021	31	0–8	0.00–34.78	0–0	0.00–0.00
	Apr 26 to May 2, 2021	34	0–11	0.00–47.83	0–0	0.00–0.00
	May 3 to May 9, 2021	50	18–28	81.82–127.27	0–0	0.00–0.00
	May 10 to May 16, 2021	30	0–8	0.00–36.36	0–0	0.00–0.00
	May 17 to May 23, 2021	24	0–2	0.00–9.09	0–0	0.00–0.00
	May 24 to May 30, 2021	22	0–0	0.00–0.00	0–0	0.00–0.00
Miyazaki						
	Apr 5 to Apr 11, 2021	23	0–0	0.00–0.00	0–3	0.00–11.54
	Apr 12 to Apr 18, 2021	24	0–0	0.00–0.00	0–3	0.00–11.11
	Apr 19 to Apr 25, 2021	24	0–0	0.00–0.00	0–2	0.00–7.69
	Apr 26 to May 2, 2021	26	0–0	0.00–0.00	0–0	0.00–0.00

	May 3 to May 9, 2021	31	0–6	0.00–24.00	0–0	0.00–0.00
	May 10 to May 16, 2021	39	4–15	16.67–62.50	0–0	0.00–0.00
	May 17 to May 23, 2021	25	0–2	0.00–8.70	0–0	0.00–0.00
	May 24 to May 30, 2021	24	0–1	0.00–4.35	0–0	0.00–0.00
Kagoshima						
	Apr 5 to Apr 11, 2021	46	0–6	0.00–15.00	0–0	0.00–0.00
	Apr 12 to Apr 18, 2021	40	0–1	0.00–2.56	0–0	0.00–0.00
	Apr 19 to Apr 25, 2021	56	5–18	13.16–47.37	0–0	0.00–0.00
	Apr 26 to May 2, 2021	45	0–8	0.00–21.62	0–0	0.00–0.00
	May 3 to May 9, 2021	38	0–0	0.00–0.00	0–0	0.00–0.00
	May 10 to May 16, 2021	56	4–17	10.26–43.59	0–0	0.00–0.00
	May 17 to May 23, 2021	43	0–5	0.00–13.16	0–0	0.00–0.00
	May 24 to May 30, 2021	39	0–1	0.00–2.63	0–0	0.00–0.00
Okinawa						
	Apr 5 to Apr 11, 2021	50	0–14	0.00–38.89	0–0	0.00–0.00
	Apr 12 to Apr 18, 2021	40	0–5	0.00–14.29	0–0	0.00–0.00
	Apr 19 to Apr 25, 2021	45	0–10	0.00–28.57	0–0	0.00–0.00
	Apr 26 to May 2, 2021	51	5–18	15.15–54.55	0–0	0.00–0.00
	May 3 to May 9, 2021	36	0–3	0.00–9.09	0–0	0.00–0.00
	May 10 to May 16, 2021	49	2–16	6.06–48.48	0–0	0.00–0.00
	May 17 to May 23, 2021	57	10–23	29.41–67.65	0–0	0.00–0.00
	May 24 to May 30, 2021	44	0–10	0.00–29.41	0–0	0.00–0.00

Table A.5: Weekly number of observed and excess/exiguous deaths in Japan and 47 prefectures for respiratory disease-related deaths in all places from January 2020 through May 2021.

Prefecture	Week	Observed	Excess deaths	Percent excess	Exiguous deaths	Percent exiguous
Japan						
	Apr 5 to Apr 11, 2021	3207	0–0	0.00–0.00	0–78	0.00–2.37
	Apr 12 to Apr 18, 2021	3330	0–90	0.00–2.78	0–0	0.00–0.00
	Apr 19 to Apr 25, 2021	3441	0–254	0.00–7.97	0–0	0.00–0.00
	Apr 26 to May 2, 2021	3461	0–340	0.00–10.89	0–0	0.00–0.00
	May 3 to May 9, 2021	3528	92–474	3.01–15.52	0–0	0.00–0.00
	May 10 to May 16, 2021	3579	185–581	6.17–19.38	0–0	0.00–0.00
	May 17 to May 23, 2021	3366	66–452	2.26–15.51	0–0	0.00–0.00
	May 24 to May 30, 2021	3183	5–361	0.18–12.79	0–0	0.00–0.00
Hokkaido						
	Apr 5 to Apr 11, 2021	138	0–3	0.00–2.22	0–0	0.00–0.00
	Apr 12 to Apr 18, 2021	126	0–0	0.00–0.00	0–8	0.00–5.97
	Apr 19 to Apr 25, 2021	125	0–0	0.00–0.00	0–8	0.00–6.02
	Apr 26 to May 2, 2021	156	0–23	0.00–17.29	0–0	0.00–0.00
	May 3 to May 9, 2021	150	0–18	0.00–13.64	0–0	0.00–0.00
	May 10 to May 16, 2021	166	6–36	4.62–27.69	0–0	0.00–0.00
	May 17 to May 23, 2021	137	0–8	0.00–6.20	0–0	0.00–0.00
	May 24 to May 30, 2021	144	0–17	0.00–13.39	0–0	0.00–0.00
Aomori						
	Apr 5 to Apr 11, 2021	35	0–0	0.00–0.00	0–4	0.00–10.26
	Apr 12 to Apr 18, 2021	41	0–2	0.00–5.13	0–0	0.00–0.00
	Apr 19 to Apr 25, 2021	39	0–0	0.00–0.00	0–0	0.00–0.00
	Apr 26 to May 2, 2021	38	0–0	0.00–0.00	0–0	0.00–0.00
	May 3 to May 9, 2021	44	0–8	0.00–22.22	0–0	0.00–0.00
	May 10 to May 16, 2021	59	11–24	31.43–68.57	0–0	0.00–0.00
	May 17 to May 23, 2021	45	0–10	0.00–28.57	0–0	0.00–0.00
	May 24 to May 30, 2021	38	0–2	0.00–5.56	0–0	0.00–0.00
Iwate						
	Apr 5 to Apr 11, 2021	29	0–0	0.00–0.00	0–9	0.00–23.68
	Apr 12 to Apr 18, 2021	45	0–7	0.00–18.42	0–0	0.00–0.00
	Apr 19 to Apr 25, 2021	28	0–0	0.00–0.00	0–10	0.00–26.32
	Apr 26 to May 2, 2021	31	0–0	0.00–0.00	0–6	0.00–16.22
	May 3 to May 9, 2021	35	0–0	0.00–0.00	0–1	0.00–2.78
	May 10 to May 16, 2021	22	0–0	0.00–0.00	1–13	2.86–37.14
	May 17 to May 23, 2021	45	0–11	0.00–32.35	0–0	0.00–0.00
	May 24 to May 30, 2021	34	0–2	0.00–6.25	0–0	0.00–0.00
Miyagi						
	Apr 5 to Apr 11, 2021	58	0–10	0.00–20.83	0–0	0.00–0.00
	Apr 12 to Apr 18, 2021	62	1–15	2.13–31.91	0–0	0.00–0.00
	Apr 19 to Apr 25, 2021	47	0–1	0.00–2.17	0–0	0.00–0.00
	Apr 26 to May 2, 2021	37	0–0	0.00–0.00	0–9	0.00–19.57
	May 3 to May 9, 2021	62	3–17	6.67–37.78	0–0	0.00–0.00

	May 10 to May 16, 2021	44	0–0	0.00–0.00	0–1	0.00–2.22
	May 17 to May 23, 2021	58	1–14	2.27–31.82	0–0	0.00–0.00
	May 24 to May 30, 2021	45	0–1	0.00–2.27	0–0	0.00–0.00
Akita						
	Apr 5 to Apr 11, 2021	32	0–0	0.00–0.00	0–2	0.00–5.88
	Apr 12 to Apr 18, 2021	26	0–0	0.00–0.00	0–8	0.00–23.53
	Apr 19 to Apr 25, 2021	35	0–0	0.00–0.00	0–0	0.00–0.00
	Apr 26 to May 2, 2021	53	8–20	24.24–60.61	0–0	0.00–0.00
	May 3 to May 9, 2021	34	0–1	0.00–3.03	0–0	0.00–0.00
	May 10 to May 16, 2021	28	0–0	0.00–0.00	0–4	0.00–12.50
	May 17 to May 23, 2021	36	0–5	0.00–16.13	0–0	0.00–0.00
	May 24 to May 30, 2021	27	0–0	0.00–0.00	0–3	0.00–10.00
Yamagata						
	Apr 5 to Apr 11, 2021	26	0–0	0.00–0.00	0–6	0.00–18.75
	Apr 12 to Apr 18, 2021	28	0–0	0.00–0.00	0–5	0.00–15.15
	Apr 19 to Apr 25, 2021	37	0–5	0.00–15.63	0–0	0.00–0.00
	Apr 26 to May 2, 2021	22	0–0	0.00–0.00	0–9	0.00–29.03
	May 3 to May 9, 2021	31	0–1	0.00–3.33	0–0	0.00–0.00
	May 10 to May 16, 2021	36	0–7	0.00–24.14	0–0	0.00–0.00
	May 17 to May 23, 2021	38	0–10	0.00–35.71	0–0	0.00–0.00
	May 24 to May 30, 2021	30	0–3	0.00–11.11	0–0	0.00–0.00
Fukushima						
	Apr 5 to Apr 11, 2021	48	0–0	0.00–0.00	0–3	0.00–5.88
	Apr 12 to Apr 18, 2021	44	0–0	0.00–0.00	0–7	0.00–13.73
	Apr 19 to Apr 25, 2021	53	0–2	0.00–3.92	0–0	0.00–0.00
	Apr 26 to May 2, 2021	61	0–11	0.00–22.00	0–0	0.00–0.00
	May 3 to May 9, 2021	65	1–17	2.08–35.42	0–0	0.00–0.00
	May 10 to May 16, 2021	59	0–11	0.00–22.92	0–0	0.00–0.00
	May 17 to May 23, 2021	59	0–11	0.00–22.92	0–0	0.00–0.00
	May 24 to May 30, 2021	52	0–6	0.00–13.04	0–0	0.00–0.00
Ibaraki						
	Apr 5 to Apr 11, 2021	72	0–0	0.00–0.00	0–9	0.00–11.11
	Apr 12 to Apr 18, 2021	94	0–13	0.00–16.05	0–0	0.00–0.00
	Apr 19 to Apr 25, 2021	83	0–3	0.00–3.75	0–0	0.00–0.00
	Apr 26 to May 2, 2021	74	0–0	0.00–0.00	0–3	0.00–3.90
	May 3 to May 9, 2021	82	0–7	0.00–9.33	0–0	0.00–0.00
	May 10 to May 16, 2021	82	0–8	0.00–10.81	0–0	0.00–0.00
	May 17 to May 23, 2021	73	0–2	0.00–2.82	0–0	0.00–0.00
	May 24 to May 30, 2021	74	0–4	0.00–5.71	0–0	0.00–0.00
Tochigi						
	Apr 5 to Apr 11, 2021	43	0–0	0.00–0.00	0–5	0.00–10.42
	Apr 12 to Apr 18, 2021	50	0–3	0.00–6.38	0–0	0.00–0.00
	Apr 19 to Apr 25, 2021	53	0–7	0.00–15.22	0–0	0.00–0.00
	Apr 26 to May 2, 2021	59	0–15	0.00–34.09	0–0	0.00–0.00
	May 3 to May 9, 2021	53	0–10	0.00–23.26	0–0	0.00–0.00
	May 10 to May 16, 2021	51	0–9	0.00–21.43	0–0	0.00–0.00

	May 17 to May 23, 2021	43	0–2	0.00–4.88	0–0	0.00–0.00
	May 24 to May 30, 2021	39	0–0	0.00–0.00	0–1	0.00–2.50
Gunma						
	Apr 5 to Apr 11, 2021	63	0–4	0.00–6.78	0–0	0.00–0.00
	Apr 12 to Apr 18, 2021	76	0–17	0.00–28.81	0–0	0.00–0.00
	Apr 19 to Apr 25, 2021	53	0–0	0.00–0.00	0–5	0.00–8.62
	Apr 26 to May 2, 2021	61	0–3	0.00–5.17	0–0	0.00–0.00
	May 3 to May 9, 2021	60	0–2	0.00–3.45	0–0	0.00–0.00
	May 10 to May 16, 2021	80	8–24	14.29–42.86	0–0	0.00–0.00
	May 17 to May 23, 2021	50	0–0	0.00–0.00	0–3	0.00–5.66
	May 24 to May 30, 2021	64	0–13	0.00–25.49	0–0	0.00–0.00
Saitama						
	Apr 5 to Apr 11, 2021	171	0–0	0.00–0.00	0–4	0.00–2.29
	Apr 12 to Apr 18, 2021	177	0–7	0.00–4.12	0–0	0.00–0.00
	Apr 19 to Apr 25, 2021	200	3–35	1.82–21.21	0–0	0.00–0.00
	Apr 26 to May 2, 2021	198	5–35	3.07–21.47	0–0	0.00–0.00
	May 3 to May 9, 2021	201	14–43	8.86–27.22	0–0	0.00–0.00
	May 10 to May 16, 2021	181	0–27	0.00–17.53	0–0	0.00–0.00
	May 17 to May 23, 2021	186	5–36	3.33–24.00	0–0	0.00–0.00
	May 24 to May 30, 2021	166	0–20	0.00–13.70	0–0	0.00–0.00
Chiba						
	Apr 5 to Apr 11, 2021	159	0–11	0.00–7.43	0–0	0.00–0.00
	Apr 12 to Apr 18, 2021	138	0–0	0.00–0.00	0–7	0.00–4.83
	Apr 19 to Apr 25, 2021	138	0–0	0.00–0.00	0–7	0.00–4.83
	Apr 26 to May 2, 2021	157	0–17	0.00–12.14	0–0	0.00–0.00
	May 3 to May 9, 2021	154	0–17	0.00–12.41	0–0	0.00–0.00
	May 10 to May 16, 2021	147	0–11	0.00–8.09	0–0	0.00–0.00
	May 17 to May 23, 2021	137	0–4	0.00–3.01	0–0	0.00–0.00
	May 24 to May 30, 2021	138	0–7	0.00–5.34	0–0	0.00–0.00
Tokyo						
	Apr 5 to Apr 11, 2021	252	0–0	0.00–0.00	0–13	0.00–4.91
	Apr 12 to Apr 18, 2021	300	0–41	0.00–15.83	0–0	0.00–0.00
	Apr 19 to Apr 25, 2021	295	0–40	0.00–15.69	0–0	0.00–0.00
	Apr 26 to May 2, 2021	285	0–34	0.00–13.55	0–0	0.00–0.00
	May 3 to May 9, 2021	298	10–53	4.08–21.63	0–0	0.00–0.00
	May 10 to May 16, 2021	266	0–25	0.00–10.37	0–0	0.00–0.00
	May 17 to May 23, 2021	263	0–29	0.00–12.39	0–0	0.00–0.00
	May 24 to May 30, 2021	274	0–43	0.00–18.61	0–0	0.00–0.00
Kanagawa						
	Apr 5 to Apr 11, 2021	185	0–0	0.00–0.00	0–7	0.00–3.65
	Apr 12 to Apr 18, 2021	199	0–10	0.00–5.29	0–0	0.00–0.00
	Apr 19 to Apr 25, 2021	208	0–23	0.00–12.43	0–0	0.00–0.00
	Apr 26 to May 2, 2021	206	0–26	0.00–14.44	0–0	0.00–0.00
	May 3 to May 9, 2021	190	0–15	0.00–8.57	0–0	0.00–0.00
	May 10 to May 16, 2021	187	0–14	0.00–8.09	0–0	0.00–0.00
	May 17 to May 23, 2021	208	7–41	4.19–24.55	0–0	0.00–0.00

	May 24 to May 30, 2021	168	0–5	0.00–3.07	0–0	0.00–0.00
Niigata						
	Apr 5 to Apr 11, 2021	66	0–6	0.00–10.00	0–0	0.00–0.00
	Apr 12 to Apr 18, 2021	55	0–0	0.00–0.00	0–5	0.00–8.33
	Apr 19 to Apr 25, 2021	58	0–0	0.00–0.00	0–1	0.00–1.69
	Apr 26 to May 2, 2021	55	0–0	0.00–0.00	0–3	0.00–5.17
	May 3 to May 9, 2021	53	0–0	0.00–0.00	0–4	0.00–7.02
	May 10 to May 16, 2021	66	0–10	0.00–17.86	0–0	0.00–0.00
	May 17 to May 23, 2021	53	0–0	0.00–0.00	0–1	0.00–1.85
	May 24 to May 30, 2021	50	0–0	0.00–0.00	0–2	0.00–3.85
Toyama						
	Apr 5 to Apr 11, 2021	34	0–4	0.00–13.33	0–0	0.00–0.00
	Apr 12 to Apr 18, 2021	29	0–0	0.00–0.00	0–1	0.00–3.33
	Apr 19 to Apr 25, 2021	36	0–6	0.00–20.00	0–0	0.00–0.00
	Apr 26 to May 2, 2021	30	0–1	0.00–3.45	0–0	0.00–0.00
	May 3 to May 9, 2021	34	0–6	0.00–21.43	0–0	0.00–0.00
	May 10 to May 16, 2021	29	0–1	0.00–3.57	0–0	0.00–0.00
	May 17 to May 23, 2021	26	0–0	0.00–0.00	0–2	0.00–7.14
	May 24 to May 30, 2021	27	0–0	0.00–0.00	0–0	0.00–0.00
Ishikawa						
	Apr 5 to Apr 11, 2021	25	0–0	0.00–0.00	0–4	0.00–13.79
	Apr 12 to Apr 18, 2021	37	0–8	0.00–27.59	0–0	0.00–0.00
	Apr 19 to Apr 25, 2021	26	0–0	0.00–0.00	0–3	0.00–10.34
	Apr 26 to May 2, 2021	32	0–3	0.00–10.34	0–0	0.00–0.00
	May 3 to May 9, 2021	37	0–9	0.00–32.14	0–0	0.00–0.00
	May 10 to May 16, 2021	29	0–2	0.00–7.41	0–0	0.00–0.00
	May 17 to May 23, 2021	19	0–0	0.00–0.00	0–8	0.00–29.63
	May 24 to May 30, 2021	35	0–9	0.00–34.62	0–0	0.00–0.00
Fukui						
	Apr 5 to Apr 11, 2021	33	0–8	0.00–32.00	0–0	0.00–0.00
	Apr 12 to Apr 18, 2021	21	0–0	0.00–0.00	0–4	0.00–16.00
	Apr 19 to Apr 25, 2021	21	0–0	0.00–0.00	0–3	0.00–12.50
	Apr 26 to May 2, 2021	31	0–8	0.00–34.78	0–0	0.00–0.00
	May 3 to May 9, 2021	30	0–7	0.00–30.43	0–0	0.00–0.00
	May 10 to May 16, 2021	32	0–9	0.00–39.13	0–0	0.00–0.00
	May 17 to May 23, 2021	31	0–9	0.00–40.91	0–0	0.00–0.00
	May 24 to May 30, 2021	30	0–9	0.00–42.86	0–0	0.00–0.00
Yamanashi						
	Apr 5 to Apr 11, 2021	17	0–0	0.00–0.00	0–6	0.00–26.09
	Apr 12 to Apr 18, 2021	18	0–0	0.00–0.00	0–5	0.00–21.74
	Apr 19 to Apr 25, 2021	31	0–9	0.00–40.91	0–0	0.00–0.00
	Apr 26 to May 2, 2021	24	0–2	0.00–9.09	0–0	0.00–0.00
	May 3 to May 9, 2021	33	3–12	14.29–57.14	0–0	0.00–0.00
	May 10 to May 16, 2021	23	0–2	0.00–9.52	0–0	0.00–0.00
	May 17 to May 23, 2021	32	1–11	4.76–52.38	0–0	0.00–0.00
	May 24 to May 30, 2021	21	0–1	0.00–5.00	0–0	0.00–0.00

Nagano						
	Apr 5 to Apr 11, 2021	60	0–5	0.00–9.09	0–0	0.00–0.00
	Apr 12 to Apr 18, 2021	55	0–2	0.00–3.77	0–0	0.00–0.00
	Apr 19 to Apr 25, 2021	47	0–0	0.00–0.00	0–5	0.00–9.62
	Apr 26 to May 2, 2021	65	0–14	0.00–27.45	0–0	0.00–0.00
	May 3 to May 9, 2021	58	0–9	0.00–18.37	0–0	0.00–0.00
	May 10 to May 16, 2021	61	0–12	0.00–24.49	0–0	0.00–0.00
	May 17 to May 23, 2021	60	0–12	0.00–25.00	0–0	0.00–0.00
	May 24 to May 30, 2021	60	0–14	0.00–30.43	0–0	0.00–0.00
Gifu						
	Apr 5 to Apr 11, 2021	49	0–0	0.00–0.00	0–10	0.00–16.95
	Apr 12 to Apr 18, 2021	61	0–4	0.00–7.02	0–0	0.00–0.00
	Apr 19 to Apr 25, 2021	54	0–0	0.00–0.00	0–2	0.00–3.57
	Apr 26 to May 2, 2021	51	0–0	0.00–0.00	0–3	0.00–5.56
	May 3 to May 9, 2021	71	4–19	7.69–36.54	0–0	0.00–0.00
	May 10 to May 16, 2021	77	11–26	21.57–50.98	0–0	0.00–0.00
	May 17 to May 23, 2021	63	0–13	0.00–26.00	0–0	0.00–0.00
	May 24 to May 30, 2021	51	0–2	0.00–4.08	0–0	0.00–0.00
Shizuoka						
	Apr 5 to Apr 11, 2021	81	0–0	0.00–0.00	0–12	0.00–12.90
	Apr 12 to Apr 18, 2021	80	0–0	0.00–0.00	0–13	0.00–13.98
	Apr 19 to Apr 25, 2021	100	0–9	0.00–9.89	0–0	0.00–0.00
	Apr 26 to May 2, 2021	102	0–16	0.00–18.60	0–0	0.00–0.00
	May 3 to May 9, 2021	87	0–4	0.00–4.82	0–0	0.00–0.00
	May 10 to May 16, 2021	85	0–5	0.00–6.25	0–0	0.00–0.00
	May 17 to May 23, 2021	102	3–24	3.85–30.77	0–0	0.00–0.00
	May 24 to May 30, 2021	86	0–11	0.00–14.67	0–0	0.00–0.00
Aichi						
	Apr 5 to Apr 11, 2021	146	0–0	0.00–0.00	0–25	0.00–14.62
	Apr 12 to Apr 18, 2021	174	0–6	0.00–3.57	0–0	0.00–0.00
	Apr 19 to Apr 25, 2021	178	0–13	0.00–7.88	0–0	0.00–0.00
	Apr 26 to May 2, 2021	194	0–31	0.00–19.02	0–0	0.00–0.00
	May 3 to May 9, 2021	175	0–16	0.00–10.06	0–0	0.00–0.00
	May 10 to May 16, 2021	191	5–37	3.25–24.03	0–0	0.00–0.00
	May 17 to May 23, 2021	164	0–13	0.00–8.61	0–0	0.00–0.00
	May 24 to May 30, 2021	168	0–22	0.00–15.07	0–0	0.00–0.00
Mie						
	Apr 5 to Apr 11, 2021	54	0–7	0.00–14.89	0–0	0.00–0.00
	Apr 12 to Apr 18, 2021	48	0–1	0.00–2.13	0–0	0.00–0.00
	Apr 19 to Apr 25, 2021	53	0–6	0.00–12.77	0–0	0.00–0.00
	Apr 26 to May 2, 2021	52	0–6	0.00–13.04	0–0	0.00–0.00
	May 3 to May 9, 2021	61	3–18	6.98–41.86	0–0	0.00–0.00
	May 10 to May 16, 2021	53	0–10	0.00–23.26	0–0	0.00–0.00
	May 17 to May 23, 2021	55	0–13	0.00–30.95	0–0	0.00–0.00
	May 24 to May 30, 2021	49	0–7	0.00–16.67	0–0	0.00–0.00
Shiga						

	Apr 5 to Apr 11, 2021	39	0–9	0.00–30.00	0–0	0.00–0.00
	Apr 12 to Apr 18, 2021	30	0–0	0.00–0.00	0–0	0.00–0.00
	Apr 19 to Apr 25, 2021	27	0–0	0.00–0.00	0–2	0.00–6.90
	Apr 26 to May 2, 2021	34	0–5	0.00–17.24	0–0	0.00–0.00
	May 3 to May 9, 2021	43	4–15	14.29–53.57	0–0	0.00–0.00
	May 10 to May 16, 2021	32	0–5	0.00–18.52	0–0	0.00–0.00
	May 17 to May 23, 2021	34	0–8	0.00–30.77	0–0	0.00–0.00
	May 24 to May 30, 2021	25	0–0	0.00–0.00	0–1	0.00–3.85
Kyoto						
	Apr 5 to Apr 11, 2021	61	0–0	0.00–0.00	0–5	0.00–7.58
	Apr 12 to Apr 18, 2021	50	0–0	0.00–0.00	0–15	0.00–23.08
	Apr 19 to Apr 25, 2021	48	0–0	0.00–0.00	0–16	0.00–25.00
	Apr 26 to May 2, 2021	66	0–3	0.00–4.76	0–0	0.00–0.00
	May 3 to May 9, 2021	77	0–16	0.00–26.23	0–0	0.00–0.00
	May 10 to May 16, 2021	66	0–7	0.00–11.86	0–0	0.00–0.00
	May 17 to May 23, 2021	47	0–0	0.00–0.00	0–10	0.00–17.54
	May 24 to May 30, 2021	60	0–4	0.00–7.14	0–0	0.00–0.00
Osaka						
	Apr 5 to Apr 11, 2021	267	0–4	0.00–1.52	0–0	0.00–0.00
	Apr 12 to Apr 18, 2021	249	0–0	0.00–0.00	0–12	0.00–4.60
	Apr 19 to Apr 25, 2021	275	0–21	0.00–8.27	0–0	0.00–0.00
	Apr 26 to May 2, 2021	245	0–0	0.00–0.00	0–4	0.00–1.61
	May 3 to May 9, 2021	282	0–37	0.00–15.10	0–0	0.00–0.00
	May 10 to May 16, 2021	302	16–59	6.58–24.28	0–0	0.00–0.00
	May 17 to May 23, 2021	250	0–9	0.00–3.73	0–0	0.00–0.00
	May 24 to May 30, 2021	276	0–41	0.00–17.45	0–0	0.00–0.00
Hyogo						
	Apr 5 to Apr 11, 2021	134	0–0	0.00–0.00	0–8	0.00–5.63
	Apr 12 to Apr 18, 2021	134	0–0	0.00–0.00	0–6	0.00–4.29
	Apr 19 to Apr 25, 2021	136	0–0	0.00–0.00	0–1	0.00–0.73
	Apr 26 to May 2, 2021	140	0–7	0.00–5.26	0–0	0.00–0.00
	May 3 to May 9, 2021	137	0–8	0.00–6.20	0–0	0.00–0.00
	May 10 to May 16, 2021	164	9–37	7.09–29.13	0–0	0.00–0.00
	May 17 to May 23, 2021	162	8–36	6.35–28.57	0–0	0.00–0.00
	May 24 to May 30, 2021	121	0–0	0.00–0.00	0–1	0.00–0.82
Nara						
	Apr 5 to Apr 11, 2021	36	0–0	0.00–0.00	0–5	0.00–12.20
	Apr 12 to Apr 18, 2021	43	0–3	0.00–7.50	0–0	0.00–0.00
	Apr 19 to Apr 25, 2021	45	0–5	0.00–12.50	0–0	0.00–0.00
	Apr 26 to May 2, 2021	41	0–2	0.00–5.13	0–0	0.00–0.00
	May 3 to May 9, 2021	37	0–0	0.00–0.00	0–1	0.00–2.63
	May 10 to May 16, 2021	44	0–7	0.00–18.92	0–0	0.00–0.00
	May 17 to May 23, 2021	42	0–7	0.00–20.00	0–0	0.00–0.00
	May 24 to May 30, 2021	34	0–0	0.00–0.00	0–0	0.00–0.00
Wakayama						
	Apr 5 to Apr 11, 2021	34	0–0	0.00–0.00	0–1	0.00–2.86

	Apr 12 to Apr 18, 2021	39	0–4	0.00–11.43	0–0	0.00–0.00
	Apr 19 to Apr 25, 2021	37	0–3	0.00–8.82	0–0	0.00–0.00
	Apr 26 to May 2, 2021	25	0–0	0.00–0.00	0–9	0.00–26.47
	May 3 to May 9, 2021	26	0–0	0.00–0.00	0–7	0.00–21.21
	May 10 to May 16, 2021	20	0–0	0.00–0.00	1–12	3.13–37.50
	May 17 to May 23, 2021	28	0–0	0.00–0.00	0–3	0.00–9.68
	May 24 to May 30, 2021	29	0–0	0.00–0.00	0–1	0.00–3.33
Tottori						
	Apr 5 to Apr 11, 2021	13	0–0	0.00–0.00	0–4	0.00–23.53
	Apr 12 to Apr 18, 2021	12	0–0	0.00–0.00	0–4	0.00–25.00
	Apr 19 to Apr 25, 2021	15	0–0	0.00–0.00	0–1	0.00–6.25
	Apr 26 to May 2, 2021	10	0–0	0.00–0.00	0–5	0.00–33.33
	May 3 to May 9, 2021	18	0–3	0.00–20.00	0–0	0.00–0.00
	May 10 to May 16, 2021	23	1–9	7.14–64.29	0–0	0.00–0.00
	May 17 to May 23, 2021	15	0–1	0.00–7.14	0–0	0.00–0.00
	May 24 to May 30, 2021	14	0–0	0.00–0.00	0–0	0.00–0.00
Shimane						
	Apr 5 to Apr 11, 2021	21	0–0	0.00–0.00	0–1	0.00–4.55
	Apr 12 to Apr 18, 2021	30	0–8	0.00–36.36	0–0	0.00–0.00
	Apr 19 to Apr 25, 2021	19	0–0	0.00–0.00	0–2	0.00–9.52
	Apr 26 to May 2, 2021	28	0–8	0.00–40.00	0–0	0.00–0.00
	May 3 to May 9, 2021	26	0–7	0.00–36.84	0–0	0.00–0.00
	May 10 to May 16, 2021	25	0–6	0.00–31.58	0–0	0.00–0.00
	May 17 to May 23, 2021	31	3–12	15.79–63.16	0–0	0.00–0.00
	May 24 to May 30, 2021	20	0–2	0.00–11.11	0–0	0.00–0.00
Okayama						
	Apr 5 to Apr 11, 2021	45	0–0	0.00–0.00	0–16	0.00–26.23
	Apr 12 to Apr 18, 2021	56	0–0	0.00–0.00	0–4	0.00–6.67
	Apr 19 to Apr 25, 2021	65	0–5	0.00–8.33	0–0	0.00–0.00
	Apr 26 to May 2, 2021	59	0–0	0.00–0.00	0–1	0.00–1.67
	May 3 to May 9, 2021	62	0–5	0.00–8.77	0–0	0.00–0.00
	May 10 to May 16, 2021	74	1–18	1.79–32.14	0–0	0.00–0.00
	May 17 to May 23, 2021	45	0–0	0.00–0.00	0–10	0.00–18.18
	May 24 to May 30, 2021	56	0–3	0.00–5.66	0–0	0.00–0.00
Hiroshima						
	Apr 5 to Apr 11, 2021	72	0–0	0.00–0.00	0–4	0.00–5.26
	Apr 12 to Apr 18, 2021	78	0–5	0.00–6.85	0–0	0.00–0.00
	Apr 19 to Apr 25, 2021	82	0–9	0.00–12.33	0–0	0.00–0.00
	Apr 26 to May 2, 2021	95	4–23	5.56–31.94	0–0	0.00–0.00
	May 3 to May 9, 2021	80	0–10	0.00–14.29	0–0	0.00–0.00
	May 10 to May 16, 2021	82	0–13	0.00–18.84	0–0	0.00–0.00
	May 17 to May 23, 2021	101	18–35	27.27–53.03	0–0	0.00–0.00
	May 24 to May 30, 2021	70	0–5	0.00–7.69	0–0	0.00–0.00
Yamaguchi						
	Apr 5 to Apr 11, 2021	52	0–3	0.00–6.12	0–0	0.00–0.00
	Apr 12 to Apr 18, 2021	47	0–0	0.00–0.00	0–1	0.00–2.08

	Apr 19 to Apr 25, 2021	58	0–12	0.00–26.09	0–0	0.00–0.00
	Apr 26 to May 2, 2021	71	11–25	23.91–54.35	0–0	0.00–0.00
	May 3 to May 9, 2021	51	0–6	0.00–13.33	0–0	0.00–0.00
	May 10 to May 16, 2021	49	0–4	0.00–8.89	0–0	0.00–0.00
	May 17 to May 23, 2021	64	5–19	11.11–42.22	0–0	0.00–0.00
	May 24 to May 30, 2021	46	0–2	0.00–4.55	0–0	0.00–0.00
Tokushima						
	Apr 5 to Apr 11, 2021	26	0–0	0.00–0.00	0–6	0.00–18.75
	Apr 12 to Apr 18, 2021	37	0–6	0.00–19.35	0–0	0.00–0.00
	Apr 19 to Apr 25, 2021	38	0–10	0.00–35.71	0–0	0.00–0.00
	Apr 26 to May 2, 2021	27	0–0	0.00–0.00	0–0	0.00–0.00
	May 3 to May 9, 2021	32	0–4	0.00–14.29	0–0	0.00–0.00
	May 10 to May 16, 2021	36	0–9	0.00–33.33	0–0	0.00–0.00
	May 17 to May 23, 2021	30	0–3	0.00–11.11	0–0	0.00–0.00
	May 24 to May 30, 2021	31	0–4	0.00–14.81	0–0	0.00–0.00
Kagawa						
	Apr 5 to Apr 11, 2021	34	0–0	0.00–0.00	0–1	0.00–2.86
	Apr 12 to Apr 18, 2021	39	0–5	0.00–14.71	0–0	0.00–0.00
	Apr 19 to Apr 25, 2021	27	0–0	0.00–0.00	0–7	0.00–20.59
	Apr 26 to May 2, 2021	36	0–3	0.00–9.09	0–0	0.00–0.00
	May 3 to May 9, 2021	34	0–2	0.00–6.25	0–0	0.00–0.00
	May 10 to May 16, 2021	36	0–5	0.00–16.13	0–0	0.00–0.00
	May 17 to May 23, 2021	34	0–3	0.00–9.68	0–0	0.00–0.00
	May 24 to May 30, 2021	38	0–9	0.00–31.03	0–0	0.00–0.00
Ehime						
	Apr 5 to Apr 11, 2021	50	0–7	0.00–16.28	0–0	0.00–0.00
	Apr 12 to Apr 18, 2021	40	0–0	0.00–0.00	0–2	0.00–4.76
	Apr 19 to Apr 25, 2021	49	0–7	0.00–16.67	0–0	0.00–0.00
	Apr 26 to May 2, 2021	49	0–8	0.00–19.51	0–0	0.00–0.00
	May 3 to May 9, 2021	33	0–0	0.00–0.00	0–8	0.00–19.51
	May 10 to May 16, 2021	60	7–20	17.50–50.00	0–0	0.00–0.00
	May 17 to May 23, 2021	35	0–0	0.00–0.00	0–4	0.00–10.26
	May 24 to May 30, 2021	35	0–0	0.00–0.00	0–2	0.00–5.41
Kochi						
	Apr 5 to Apr 11, 2021	35	0–8	0.00–29.63	0–0	0.00–0.00
	Apr 12 to Apr 18, 2021	26	0–0	0.00–0.00	0–1	0.00–3.70
	Apr 19 to Apr 25, 2021	19	0–0	0.00–0.00	0–7	0.00–26.92
	Apr 26 to May 2, 2021	19	0–0	0.00–0.00	0–6	0.00–24.00
	May 3 to May 9, 2021	22	0–0	0.00–0.00	0–2	0.00–8.33
	May 10 to May 16, 2021	34	0–10	0.00–41.67	0–0	0.00–0.00
	May 17 to May 23, 2021	32	0–9	0.00–39.13	0–0	0.00–0.00
	May 24 to May 30, 2021	24	0–2	0.00–9.09	0–0	0.00–0.00
Fukuoka						
	Apr 5 to Apr 11, 2021	144	0–3	0.00–2.13	0–0	0.00–0.00
	Apr 12 to Apr 18, 2021	139	0–0	0.00–0.00	0–0	0.00–0.00
	Apr 19 to Apr 25, 2021	183	16–45	11.59–32.61	0–0	0.00–0.00

	Apr 26 to May 2, 2021	148	0–13	0.00–9.63	0–0	0.00–0.00
	May 3 to May 9, 2021	155	0–23	0.00–17.42	0–0	0.00–0.00
	May 10 to May 16, 2021	153	0–22	0.00–16.79	0–0	0.00–0.00
	May 17 to May 23, 2021	159	0–29	0.00–22.31	0–0	0.00–0.00
	May 24 to May 30, 2021	157	1–31	0.79–24.60	0–0	0.00–0.00
Saga						
	Apr 5 to Apr 11, 2021	31	0–3	0.00–10.71	0–0	0.00–0.00
	Apr 12 to Apr 18, 2021	32	0–4	0.00–14.29	0–0	0.00–0.00
	Apr 19 to Apr 25, 2021	31	0–4	0.00–14.81	0–0	0.00–0.00
	Apr 26 to May 2, 2021	33	0–7	0.00–26.92	0–0	0.00–0.00
	May 3 to May 9, 2021	28	0–2	0.00–7.69	0–0	0.00–0.00
	May 10 to May 16, 2021	43	7–17	26.92–65.38	0–0	0.00–0.00
	May 17 to May 23, 2021	26	0–0	0.00–0.00	0–0	0.00–0.00
	May 24 to May 30, 2021	21	0–0	0.00–0.00	0–4	0.00–16.00
Nagasaki						
	Apr 5 to Apr 11, 2021	42	0–0	0.00–0.00	0–6	0.00–12.50
	Apr 12 to Apr 18, 2021	46	0–0	0.00–0.00	0–1	0.00–2.13
	Apr 19 to Apr 25, 2021	50	0–4	0.00–8.70	0–0	0.00–0.00
	Apr 26 to May 2, 2021	46	0–0	0.00–0.00	0–0	0.00–0.00
	May 3 to May 9, 2021	56	0–11	0.00–24.44	0–0	0.00–0.00
	May 10 to May 16, 2021	59	3–16	6.98–37.21	0–0	0.00–0.00
	May 17 to May 23, 2021	36	0–0	0.00–0.00	0–6	0.00–14.29
	May 24 to May 30, 2021	52	0–11	0.00–26.83	0–0	0.00–0.00
Kumamoto						
	Apr 5 to Apr 11, 2021	42	0–0	0.00–0.00	1–17	1.69–28.81
	Apr 12 to Apr 18, 2021	60	0–0	0.00–0.00	0–0	0.00–0.00
	Apr 19 to Apr 25, 2021	66	0–8	0.00–13.79	0–0	0.00–0.00
	Apr 26 to May 2, 2021	64	0–6	0.00–10.34	0–0	0.00–0.00
	May 3 to May 9, 2021	65	0–9	0.00–16.07	0–0	0.00–0.00
	May 10 to May 16, 2021	66	0–11	0.00–20.00	0–0	0.00–0.00
	May 17 to May 23, 2021	66	0–12	0.00–22.22	0–0	0.00–0.00
	May 24 to May 30, 2021	60	0–7	0.00–13.21	0–0	0.00–0.00
Oita						
	Apr 5 to Apr 11, 2021	36	0–0	0.00–0.00	0–0	0.00–0.00
	Apr 12 to Apr 18, 2021	46	0–11	0.00–31.43	0–0	0.00–0.00
	Apr 19 to Apr 25, 2021	44	0–9	0.00–25.71	0–0	0.00–0.00
	Apr 26 to May 2, 2021	38	0–3	0.00–8.57	0–0	0.00–0.00
	May 3 to May 9, 2021	39	0–6	0.00–18.18	0–0	0.00–0.00
	May 10 to May 16, 2021	42	0–7	0.00–20.00	0–0	0.00–0.00
	May 17 to May 23, 2021	26	0–0	0.00–0.00	0–8	0.00–23.53
	May 24 to May 30, 2021	27	0–0	0.00–0.00	0–5	0.00–15.63
Miyazaki						
	Apr 5 to Apr 11, 2021	22	0–0	0.00–0.00	2–14	5.56–38.89
	Apr 12 to Apr 18, 2021	36	0–0	0.00–0.00	0–0	0.00–0.00
	Apr 19 to Apr 25, 2021	44	0–9	0.00–25.71	0–0	0.00–0.00
	Apr 26 to May 2, 2021	45	0–9	0.00–25.00	0–0	0.00–0.00

	May 3 to May 9, 2021	46	0–11	0.00–31.43	0–0	0.00–0.00
	May 10 to May 16, 2021	34	0–0	0.00–0.00	0–1	0.00–2.86
	May 17 to May 23, 2021	38	0–4	0.00–11.76	0–0	0.00–0.00
	May 24 to May 30, 2021	35	0–3	0.00–9.38	0–0	0.00–0.00
Kagoshima						
	Apr 5 to Apr 11, 2021	67	0–8	0.00–13.56	0–0	0.00–0.00
	Apr 12 to Apr 18, 2021	47	0–0	0.00–0.00	0–12	0.00–20.34
	Apr 19 to Apr 25, 2021	63	0–7	0.00–12.50	0–0	0.00–0.00
	Apr 26 to May 2, 2021	44	0–0	0.00–0.00	0–12	0.00–21.43
	May 3 to May 9, 2021	59	0–4	0.00–7.27	0–0	0.00–0.00
	May 10 to May 16, 2021	53	0–0	0.00–0.00	0–2	0.00–3.64
	May 17 to May 23, 2021	59	0–6	0.00–11.32	0–0	0.00–0.00
	May 24 to May 30, 2021	62	0–11	0.00–21.57	0–0	0.00–0.00
Okinawa						
	Apr 5 to Apr 11, 2021	28	0–1	0.00–3.70	0–0	0.00–0.00
	Apr 12 to Apr 18, 2021	30	0–3	0.00–11.11	0–0	0.00–0.00
	Apr 19 to Apr 25, 2021	22	0–0	0.00–0.00	0–6	0.00–21.43
	Apr 26 to May 2, 2021	24	0–0	0.00–0.00	0–3	0.00–11.11
	May 3 to May 9, 2021	22	0–0	0.00–0.00	0–4	0.00–15.38
	May 10 to May 16, 2021	28	0–2	0.00–7.69	0–0	0.00–0.00
	May 17 to May 23, 2021	23	0–0	0.00–0.00	0–2	0.00–8.00
	May 24 to May 30, 2021	20	0–0	0.00–0.00	0–5	0.00–20.00

Table A.6: Weekly number of observed and excess/exiguous deaths in Japan and 47 prefectures for respiratory disease-related deaths in hospitals and clinics from January 2020 through May 2021.

Prefecture	Week	Observed	Excess deaths	Percent excess	Exiguous deaths	Percent exiguous
Japan						
	Apr 5 to Apr 11, 2021	2728	0–0	0.00–0.00	0–103	0.00–3.64
	Apr 12 to Apr 18, 2021	2793	0–9	0.00–0.32	0–0	0.00–0.00
	Apr 19 to Apr 25, 2021	2906	0–168	0.00–6.14	0–0	0.00–0.00
	Apr 26 to May 2, 2021	2916	0–242	0.00–9.05	0–0	0.00–0.00
	May 3 to May 9, 2021	2916	0–305	0.00–11.68	0–0	0.00–0.00
	May 10 to May 16, 2021	2907	5–358	0.20–14.04	0–0	0.00–0.00
	May 17 to May 23, 2021	2742	0–252	0.00–10.12	0–0	0.00–0.00
	May 24 to May 30, 2021	2603	0–184	0.00–7.61	0–0	0.00–0.00
Hokkaido						
	Apr 5 to Apr 11, 2021	129	0–4	0.00–3.20	0–0	0.00–0.00
	Apr 12 to Apr 18, 2021	116	0–0	0.00–0.00	0–9	0.00–7.20
	Apr 19 to Apr 25, 2021	116	0–0	0.00–0.00	0–8	0.00–6.45
	Apr 26 to May 2, 2021	147	0–24	0.00–19.51	0–0	0.00–0.00
	May 3 to May 9, 2021	131	0–9	0.00–7.38	0–0	0.00–0.00
	May 10 to May 16, 2021	146	0–26	0.00–21.67	0–0	0.00–0.00
	May 17 to May 23, 2021	130	0–11	0.00–9.24	0–0	0.00–0.00
	May 24 to May 30, 2021	130	0–12	0.00–10.17	0–0	0.00–0.00
Aomori						
	Apr 5 to Apr 11, 2021	29	0–0	0.00–0.00	0–2	0.00–6.45
	Apr 12 to Apr 18, 2021	36	0–5	0.00–16.13	0–0	0.00–0.00
	Apr 19 to Apr 25, 2021	33	0–2	0.00–6.45	0–0	0.00–0.00
	Apr 26 to May 2, 2021	31	0–0	0.00–0.00	0–0	0.00–0.00
	May 3 to May 9, 2021	35	0–5	0.00–16.67	0–0	0.00–0.00
	May 10 to May 16, 2021	44	4–15	13.79–51.72	0–0	0.00–0.00
	May 17 to May 23, 2021	32	0–3	0.00–10.34	0–0	0.00–0.00
	May 24 to May 30, 2021	30	0–0	0.00–0.00	0–0	0.00–0.00
Iwate						
	Apr 5 to Apr 11, 2021	25	0–0	0.00–0.00	0–8	0.00–24.24
	Apr 12 to Apr 18, 2021	42	0–10	0.00–31.25	0–0	0.00–0.00
	Apr 19 to Apr 25, 2021	27	0–0	0.00–0.00	0–5	0.00–15.63
	Apr 26 to May 2, 2021	30	0–0	0.00–0.00	0–2	0.00–6.25
	May 3 to May 9, 2021	30	0–0	0.00–0.00	0–1	0.00–3.23
	May 10 to May 16, 2021	16	0–0	0.00–0.00	3–14	10.00–46.67
	May 17 to May 23, 2021	34	0–5	0.00–17.24	0–0	0.00–0.00
	May 24 to May 30, 2021	29	0–1	0.00–3.57	0–0	0.00–0.00
Miyagi						
	Apr 5 to Apr 11, 2021	46	0–5	0.00–12.20	0–0	0.00–0.00
	Apr 12 to Apr 18, 2021	47	0–8	0.00–20.51	0–0	0.00–0.00
	Apr 19 to Apr 25, 2021	38	0–0	0.00–0.00	0–1	0.00–2.56
	Apr 26 to May 2, 2021	30	0–0	0.00–0.00	0–9	0.00–23.08
	May 3 to May 9, 2021	50	0–12	0.00–31.58	0–0	0.00–0.00

	May 10 to May 16, 2021	36	0–0	0.00–0.00	0–2	0.00–5.26
	May 17 to May 23, 2021	45	0–7	0.00–18.42	0–0	0.00–0.00
	May 24 to May 30, 2021	36	0–0	0.00–0.00	0–1	0.00–2.70
Akita						
	Apr 5 to Apr 11, 2021	31	0–0	0.00–0.00	0–1	0.00–3.13
	Apr 12 to Apr 18, 2021	21	0–0	0.00–0.00	0–11	0.00–34.38
	Apr 19 to Apr 25, 2021	34	0–2	0.00–6.25	0–0	0.00–0.00
	Apr 26 to May 2, 2021	49	7–18	22.58–58.06	0–0	0.00–0.00
	May 3 to May 9, 2021	29	0–0	0.00–0.00	0–2	0.00–6.45
	May 10 to May 16, 2021	24	0–0	0.00–0.00	0–6	0.00–20.00
	May 17 to May 23, 2021	32	0–3	0.00–10.34	0–0	0.00–0.00
	May 24 to May 30, 2021	26	0–0	0.00–0.00	0–1	0.00–3.70
Yamagata						
	Apr 5 to Apr 11, 2021	22	0–0	0.00–0.00	0–6	0.00–21.43
	Apr 12 to Apr 18, 2021	26	0–0	0.00–0.00	0–2	0.00–7.14
	Apr 19 to Apr 25, 2021	27	0–0	0.00–0.00	0–1	0.00–3.57
	Apr 26 to May 2, 2021	21	0–0	0.00–0.00	0–5	0.00–19.23
	May 3 to May 9, 2021	21	0–0	0.00–0.00	0–5	0.00–19.23
	May 10 to May 16, 2021	30	0–5	0.00–20.00	0–0	0.00–0.00
	May 17 to May 23, 2021	29	0–5	0.00–20.83	0–0	0.00–0.00
	May 24 to May 30, 2021	24	0–1	0.00–4.35	0–0	0.00–0.00
Fukushima						
	Apr 5 to Apr 11, 2021	41	0–0	0.00–0.00	0–4	0.00–8.89
	Apr 12 to Apr 18, 2021	30	0–0	0.00–0.00	1–15	2.22–33.33
	Apr 19 to Apr 25, 2021	49	0–5	0.00–11.36	0–0	0.00–0.00
	Apr 26 to May 2, 2021	52	0–9	0.00–20.93	0–0	0.00–0.00
	May 3 to May 9, 2021	57	0–15	0.00–35.71	0–0	0.00–0.00
	May 10 to May 16, 2021	46	0–4	0.00–9.52	0–0	0.00–0.00
	May 17 to May 23, 2021	49	0–7	0.00–16.67	0–0	0.00–0.00
	May 24 to May 30, 2021	43	0–3	0.00–7.50	0–0	0.00–0.00
Ibaraki						
	Apr 5 to Apr 11, 2021	65	0–0	0.00–0.00	0–8	0.00–10.96
	Apr 12 to Apr 18, 2021	83	0–12	0.00–16.90	0–0	0.00–0.00
	Apr 19 to Apr 25, 2021	71	0–1	0.00–1.43	0–0	0.00–0.00
	Apr 26 to May 2, 2021	62	0–0	0.00–0.00	0–6	0.00–8.82
	May 3 to May 9, 2021	70	0–3	0.00–4.48	0–0	0.00–0.00
	May 10 to May 16, 2021	70	0–4	0.00–6.06	0–0	0.00–0.00
	May 17 to May 23, 2021	61	0–0	0.00–0.00	0–3	0.00–4.69
	May 24 to May 30, 2021	66	0–3	0.00–4.76	0–0	0.00–0.00
Tochigi						
	Apr 5 to Apr 11, 2021	37	0–0	0.00–0.00	0–4	0.00–9.76
	Apr 12 to Apr 18, 2021	43	0–3	0.00–7.50	0–0	0.00–0.00
	Apr 19 to Apr 25, 2021	43	0–4	0.00–10.26	0–0	0.00–0.00
	Apr 26 to May 2, 2021	44	0–6	0.00–15.79	0–0	0.00–0.00
	May 3 to May 9, 2021	44	0–7	0.00–18.92	0–0	0.00–0.00
	May 10 to May 16, 2021	40	0–4	0.00–11.11	0–0	0.00–0.00

	May 17 to May 23, 2021	38	0–3	0.00–8.57	0–0	0.00–0.00
	May 24 to May 30, 2021	28	0–0	0.00–0.00	0–6	0.00–17.65
Gunma						
	Apr 5 to Apr 11, 2021	52	0–0	0.00–0.00	0–0	0.00–0.00
	Apr 12 to Apr 18, 2021	65	0–14	0.00–27.45	0–0	0.00–0.00
	Apr 19 to Apr 25, 2021	42	0–0	0.00–0.00	0–9	0.00–17.65
	Apr 26 to May 2, 2021	49	0–0	0.00–0.00	0–0	0.00–0.00
	May 3 to May 9, 2021	49	0–0	0.00–0.00	0–0	0.00–0.00
	May 10 to May 16, 2021	60	0–12	0.00–25.00	0–0	0.00–0.00
	May 17 to May 23, 2021	43	0–0	0.00–0.00	0–3	0.00–6.52
	May 24 to May 30, 2021	47	0–2	0.00–4.44	0–0	0.00–0.00
Saitama						
	Apr 5 to Apr 11, 2021	151	0–0	0.00–0.00	0–5	0.00–3.21
	Apr 12 to Apr 18, 2021	152	0–1	0.00–0.66	0–0	0.00–0.00
	Apr 19 to Apr 25, 2021	180	4–34	2.74–23.29	0–0	0.00–0.00
	Apr 26 to May 2, 2021	170	0–26	0.00–18.06	0–0	0.00–0.00
	May 3 to May 9, 2021	171	4–32	2.88–23.02	0–0	0.00–0.00
	May 10 to May 16, 2021	163	0–27	0.00–19.85	0–0	0.00–0.00
	May 17 to May 23, 2021	163	1–30	0.75–22.56	0–0	0.00–0.00
	May 24 to May 30, 2021	140	0–11	0.00–8.53	0–0	0.00–0.00
Chiba						
	Apr 5 to Apr 11, 2021	137	0–7	0.00–5.38	0–0	0.00–0.00
	Apr 12 to Apr 18, 2021	117	0–0	0.00–0.00	0–9	0.00–7.14
	Apr 19 to Apr 25, 2021	115	0–0	0.00–0.00	0–11	0.00–8.73
	Apr 26 to May 2, 2021	136	0–13	0.00–10.57	0–0	0.00–0.00
	May 3 to May 9, 2021	125	0–6	0.00–5.04	0–0	0.00–0.00
	May 10 to May 16, 2021	121	0–2	0.00–1.68	0–0	0.00–0.00
	May 17 to May 23, 2021	123	0–7	0.00–6.03	0–0	0.00–0.00
	May 24 to May 30, 2021	118	0–4	0.00–3.51	0–0	0.00–0.00
Tokyo						
	Apr 5 to Apr 11, 2021	213	0–0	0.00–0.00	0–6	0.00–2.74
	Apr 12 to Apr 18, 2021	242	0–29	0.00–13.62	0–0	0.00–0.00
	Apr 19 to Apr 25, 2021	249	0–41	0.00–19.71	0–0	0.00–0.00
	Apr 26 to May 2, 2021	237	0–31	0.00–15.05	0–0	0.00–0.00
	May 3 to May 9, 2021	228	0–27	0.00–13.43	0–0	0.00–0.00
	May 10 to May 16, 2021	215	0–18	0.00–9.14	0–0	0.00–0.00
	May 17 to May 23, 2021	216	0–24	0.00–12.50	0–0	0.00–0.00
	May 24 to May 30, 2021	221	0–32	0.00–16.93	0–0	0.00–0.00
Kanagawa						
	Apr 5 to Apr 11, 2021	145	0–0	0.00–0.00	0–15	0.00–9.38
	Apr 12 to Apr 18, 2021	164	0–8	0.00–5.13	0–0	0.00–0.00
	Apr 19 to Apr 25, 2021	168	0–19	0.00–12.75	0–0	0.00–0.00
	Apr 26 to May 2, 2021	161	0–14	0.00–9.52	0–0	0.00–0.00
	May 3 to May 9, 2021	154	0–11	0.00–7.69	0–0	0.00–0.00
	May 10 to May 16, 2021	148	0–7	0.00–4.96	0–0	0.00–0.00
	May 17 to May 23, 2021	163	0–27	0.00–19.85	0–0	0.00–0.00

	May 24 to May 30, 2021	131	0–0	0.00–0.00	0–1	0.00–0.76
Niigata						
	Apr 5 to Apr 11, 2021	56	0–3	0.00–5.66	0–0	0.00–0.00
	Apr 12 to Apr 18, 2021	50	0–0	0.00–0.00	0–3	0.00–5.66
	Apr 19 to Apr 25, 2021	48	0–0	0.00–0.00	0–4	0.00–7.69
	Apr 26 to May 2, 2021	48	0–0	0.00–0.00	0–3	0.00–5.88
	May 3 to May 9, 2021	42	0–0	0.00–0.00	0–8	0.00–16.00
	May 10 to May 16, 2021	55	0–6	0.00–12.24	0–0	0.00–0.00
	May 17 to May 23, 2021	46	0–0	0.00–0.00	0–1	0.00–2.13
	May 24 to May 30, 2021	43	0–0	0.00–0.00	0–4	0.00–8.51
Toyama						
	Apr 5 to Apr 11, 2021	32	0–6	0.00–23.08	0–0	0.00–0.00
	Apr 12 to Apr 18, 2021	22	0–0	0.00–0.00	0–3	0.00–12.00
	Apr 19 to Apr 25, 2021	29	0–4	0.00–16.00	0–0	0.00–0.00
	Apr 26 to May 2, 2021	26	0–1	0.00–4.00	0–0	0.00–0.00
	May 3 to May 9, 2021	28	0–4	0.00–16.67	0–0	0.00–0.00
	May 10 to May 16, 2021	25	0–1	0.00–4.17	0–0	0.00–0.00
	May 17 to May 23, 2021	23	0–0	0.00–0.00	0–1	0.00–4.17
	May 24 to May 30, 2021	25	0–2	0.00–8.70	0–0	0.00–0.00
Ishikawa						
	Apr 5 to Apr 11, 2021	21	0–0	0.00–0.00	0–3	0.00–12.50
	Apr 12 to Apr 18, 2021	28	0–4	0.00–16.67	0–0	0.00–0.00
	Apr 19 to Apr 25, 2021	19	0–0	0.00–0.00	0–5	0.00–20.83
	Apr 26 to May 2, 2021	28	0–4	0.00–16.67	0–0	0.00–0.00
	May 3 to May 9, 2021	32	0–9	0.00–39.13	0–0	0.00–0.00
	May 10 to May 16, 2021	24	0–2	0.00–9.09	0–0	0.00–0.00
	May 17 to May 23, 2021	16	0–0	0.00–0.00	0–6	0.00–27.27
	May 24 to May 30, 2021	32	1–10	4.55–45.45	0–0	0.00–0.00
Fukui						
	Apr 5 to Apr 11, 2021	31	1–11	5.00–55.00	0–0	0.00–0.00
	Apr 12 to Apr 18, 2021	16	0–0	0.00–0.00	0–5	0.00–23.81
	Apr 19 to Apr 25, 2021	17	0–0	0.00–0.00	0–3	0.00–15.00
	Apr 26 to May 2, 2021	26	0–7	0.00–36.84	0–0	0.00–0.00
	May 3 to May 9, 2021	23	0–4	0.00–21.05	0–0	0.00–0.00
	May 10 to May 16, 2021	25	0–6	0.00–31.58	0–0	0.00–0.00
	May 17 to May 23, 2021	25	0–6	0.00–31.58	0–0	0.00–0.00
	May 24 to May 30, 2021	22	0–4	0.00–22.22	0–0	0.00–0.00
Yamanashi						
	Apr 5 to Apr 11, 2021	15	0–0	0.00–0.00	0–4	0.00–21.05
	Apr 12 to Apr 18, 2021	16	0–0	0.00–0.00	0–3	0.00–15.79
	Apr 19 to Apr 25, 2021	28	1–10	5.56–55.56	0–0	0.00–0.00
	Apr 26 to May 2, 2021	19	0–1	0.00–5.56	0–0	0.00–0.00
	May 3 to May 9, 2021	29	3–11	16.67–61.11	0–0	0.00–0.00
	May 10 to May 16, 2021	21	0–3	0.00–16.67	0–0	0.00–0.00
	May 17 to May 23, 2021	21	0–3	0.00–16.67	0–0	0.00–0.00
	May 24 to May 30, 2021	15	0–0	0.00–0.00	0–2	0.00–11.76

Nagano						
	Apr 5 to Apr 11, 2021	49	0–4	0.00–8.89	0–0	0.00–0.00
	Apr 12 to Apr 18, 2021	42	0–0	0.00–0.00	0–2	0.00–4.55
	Apr 19 to Apr 25, 2021	39	0–0	0.00–0.00	0–4	0.00–9.30
	Apr 26 to May 2, 2021	55	0–12	0.00–27.91	0–0	0.00–0.00
	May 3 to May 9, 2021	45	0–4	0.00–9.76	0–0	0.00–0.00
	May 10 to May 16, 2021	51	0–10	0.00–24.39	0–0	0.00–0.00
	May 17 to May 23, 2021	46	0–7	0.00–17.95	0–0	0.00–0.00
	May 24 to May 30, 2021	47	0–9	0.00–23.68	0–0	0.00–0.00
Gifu						
	Apr 5 to Apr 11, 2021	39	0–0	0.00–0.00	0–10	0.00–20.41
	Apr 12 to Apr 18, 2021	49	0–2	0.00–4.26	0–0	0.00–0.00
	Apr 19 to Apr 25, 2021	45	0–0	0.00–0.00	0–1	0.00–2.17
	Apr 26 to May 2, 2021	45	0–0	0.00–0.00	0–0	0.00–0.00
	May 3 to May 9, 2021	52	0–9	0.00–20.93	0–0	0.00–0.00
	May 10 to May 16, 2021	63	6–21	14.29–50.00	0–0	0.00–0.00
	May 17 to May 23, 2021	50	0–9	0.00–21.95	0–0	0.00–0.00
	May 24 to May 30, 2021	41	0–1	0.00–2.50	0–0	0.00–0.00
Shizuoka						
	Apr 5 to Apr 11, 2021	67	0–0	0.00–0.00	0–9	0.00–11.84
	Apr 12 to Apr 18, 2021	58	0–0	0.00–0.00	0–17	0.00–22.67
	Apr 19 to Apr 25, 2021	82	0–10	0.00–13.89	0–0	0.00–0.00
	Apr 26 to May 2, 2021	82	0–14	0.00–20.59	0–0	0.00–0.00
	May 3 to May 9, 2021	73	0–6	0.00–8.96	0–0	0.00–0.00
	May 10 to May 16, 2021	62	0–0	0.00–0.00	0–2	0.00–3.13
	May 17 to May 23, 2021	79	0–18	0.00–29.51	0–0	0.00–0.00
	May 24 to May 30, 2021	69	0–10	0.00–16.95	0–0	0.00–0.00
Aichi						
	Apr 5 to Apr 11, 2021	127	0–0	0.00–0.00	0–20	0.00–13.61
	Apr 12 to Apr 18, 2021	149	0–6	0.00–4.20	0–0	0.00–0.00
	Apr 19 to Apr 25, 2021	152	0–11	0.00–7.80	0–0	0.00–0.00
	Apr 26 to May 2, 2021	165	0–27	0.00–19.57	0–0	0.00–0.00
	May 3 to May 9, 2021	144	0–9	0.00–6.67	0–0	0.00–0.00
	May 10 to May 16, 2021	148	0–17	0.00–12.98	0–0	0.00–0.00
	May 17 to May 23, 2021	129	0–1	0.00–0.78	0–0	0.00–0.00
	May 24 to May 30, 2021	136	0–12	0.00–9.68	0–0	0.00–0.00
Mie						
	Apr 5 to Apr 11, 2021	38	0–0	0.00–0.00	0–2	0.00–5.00
	Apr 12 to Apr 18, 2021	45	0–6	0.00–15.38	0–0	0.00–0.00
	Apr 19 to Apr 25, 2021	44	0–5	0.00–12.82	0–0	0.00–0.00
	Apr 26 to May 2, 2021	45	0–6	0.00–15.38	0–0	0.00–0.00
	May 3 to May 9, 2021	52	2–16	5.56–44.44	0–0	0.00–0.00
	May 10 to May 16, 2021	39	0–3	0.00–8.33	0–0	0.00–0.00
	May 17 to May 23, 2021	37	0–2	0.00–5.71	0–0	0.00–0.00
	May 24 to May 30, 2021	39	0–5	0.00–14.71	0–0	0.00–0.00
Shiga						

	Apr 5 to Apr 11, 2021	34	0–7	0.00–25.93	0–0	0.00–0.00
	Apr 12 to Apr 18, 2021	25	0–0	0.00–0.00	0–1	0.00–3.85
	Apr 19 to Apr 25, 2021	24	0–0	0.00–0.00	0–2	0.00–7.69
	Apr 26 to May 2, 2021	28	0–3	0.00–12.00	0–0	0.00–0.00
	May 3 to May 9, 2021	39	4–15	16.67–62.50	0–0	0.00–0.00
	May 10 to May 16, 2021	28	0–4	0.00–16.67	0–0	0.00–0.00
	May 17 to May 23, 2021	28	0–5	0.00–21.74	0–0	0.00–0.00
	May 24 to May 30, 2021	16	0–0	0.00–0.00	0–6	0.00–27.27
Kyoto						
	Apr 5 to Apr 11, 2021	51	0–0	0.00–0.00	0–7	0.00–12.07
	Apr 12 to Apr 18, 2021	45	0–0	0.00–0.00	0–12	0.00–21.05
	Apr 19 to Apr 25, 2021	37	0–0	0.00–0.00	4–19	7.14–33.93
	Apr 26 to May 2, 2021	59	0–4	0.00–7.27	0–0	0.00–0.00
	May 3 to May 9, 2021	66	0–13	0.00–24.53	0–0	0.00–0.00
	May 10 to May 16, 2021	52	0–1	0.00–1.96	0–0	0.00–0.00
	May 17 to May 23, 2021	40	0–0	0.00–0.00	0–10	0.00–20.00
	May 24 to May 30, 2021	48	0–0	0.00–0.00	0–0	0.00–0.00
Osaka						
	Apr 5 to Apr 11, 2021	226	0–0	0.00–0.00	0–0	0.00–0.00
	Apr 12 to Apr 18, 2021	214	0–0	0.00–0.00	0–11	0.00–4.89
	Apr 19 to Apr 25, 2021	231	0–12	0.00–5.48	0–0	0.00–0.00
	Apr 26 to May 2, 2021	200	0–0	0.00–0.00	0–15	0.00–6.98
	May 3 to May 9, 2021	237	0–26	0.00–12.32	0–0	0.00–0.00
	May 10 to May 16, 2021	248	1–40	0.48–19.23	0–0	0.00–0.00
	May 17 to May 23, 2021	199	0–0	0.00–0.00	0–6	0.00–2.93
	May 24 to May 30, 2021	235	0–34	0.00–16.92	0–0	0.00–0.00
Hyogo						
	Apr 5 to Apr 11, 2021	116	0–0	0.00–0.00	0–5	0.00–4.13
	Apr 12 to Apr 18, 2021	114	0–0	0.00–0.00	0–6	0.00–5.00
	Apr 19 to Apr 25, 2021	115	0–0	0.00–0.00	0–3	0.00–2.54
	Apr 26 to May 2, 2021	117	0–3	0.00–2.63	0–0	0.00–0.00
	May 3 to May 9, 2021	118	0–9	0.00–8.26	0–0	0.00–0.00
	May 10 to May 16, 2021	126	0–19	0.00–17.76	0–0	0.00–0.00
	May 17 to May 23, 2021	126	0–20	0.00–18.87	0–0	0.00–0.00
	May 24 to May 30, 2021	94	0–0	0.00–0.00	0–10	0.00–9.62
Nara						
	Apr 5 to Apr 11, 2021	32	0–0	0.00–0.00	0–3	0.00–8.57
	Apr 12 to Apr 18, 2021	31	0–0	0.00–0.00	0–4	0.00–11.43
	Apr 19 to Apr 25, 2021	36	0–2	0.00–5.88	0–0	0.00–0.00
	Apr 26 to May 2, 2021	32	0–0	0.00–0.00	0–2	0.00–5.88
	May 3 to May 9, 2021	28	0–0	0.00–0.00	0–5	0.00–15.15
	May 10 to May 16, 2021	38	0–6	0.00–18.75	0–0	0.00–0.00
	May 17 to May 23, 2021	30	0–0	0.00–0.00	0–1	0.00–3.23
	May 24 to May 30, 2021	31	0–1	0.00–3.33	0–0	0.00–0.00
Wakayama						
	Apr 5 to Apr 11, 2021	27	0–0	0.00–0.00	0–1	0.00–3.57

	Apr 12 to Apr 18, 2021	28	0–0	0.00–0.00	0–0	0.00–0.00
	Apr 19 to Apr 25, 2021	30	0–2	0.00–7.14	0–0	0.00–0.00
	Apr 26 to May 2, 2021	20	0–0	0.00–0.00	0–8	0.00–28.57
	May 3 to May 9, 2021	22	0–0	0.00–0.00	0–5	0.00–18.52
	May 10 to May 16, 2021	13	0–0	0.00–0.00	4–14	14.81–51.85
	May 17 to May 23, 2021	19	0–0	0.00–0.00	0–7	0.00–26.92
	May 24 to May 30, 2021	20	0–0	0.00–0.00	0–5	0.00–20.00
Tottori						
	Apr 5 to Apr 11, 2021	7	0–0	0.00–0.00	0–7	0.00–50.00
	Apr 12 to Apr 18, 2021	9	0–0	0.00–0.00	0–4	0.00–30.77
	Apr 19 to Apr 25, 2021	13	0–0	0.00–0.00	0–0	0.00–0.00
	Apr 26 to May 2, 2021	10	0–0	0.00–0.00	0–2	0.00–16.67
	May 3 to May 9, 2021	12	0–0	0.00–0.00	0–0	0.00–0.00
	May 10 to May 16, 2021	11	0–0	0.00–0.00	0–0	0.00–0.00
	May 17 to May 23, 2021	11	0–0	0.00–0.00	0–1	0.00–8.33
	May 24 to May 30, 2021	12	0–1	0.00–9.09	0–0	0.00–0.00
Shimane						
	Apr 5 to Apr 11, 2021	16	0–0	0.00–0.00	0–3	0.00–15.79
	Apr 12 to Apr 18, 2021	22	0–3	0.00–15.79	0–0	0.00–0.00
	Apr 19 to Apr 25, 2021	17	0–0	0.00–0.00	0–1	0.00–5.56
	Apr 26 to May 2, 2021	17	0–0	0.00–0.00	0–1	0.00–5.56
	May 3 to May 9, 2021	23	0–7	0.00–43.75	0–0	0.00–0.00
	May 10 to May 16, 2021	19	0–3	0.00–18.75	0–0	0.00–0.00
	May 17 to May 23, 2021	23	0–8	0.00–53.33	0–0	0.00–0.00
	May 24 to May 30, 2021	11	0–0	0.00–0.00	0–4	0.00–26.67
Okayama						
	Apr 5 to Apr 11, 2021	37	0–0	0.00–0.00	2–16	3.77–30.19
	Apr 12 to Apr 18, 2021	52	0–0	0.00–0.00	0–0	0.00–0.00
	Apr 19 to Apr 25, 2021	53	0–2	0.00–3.92	0–0	0.00–0.00
	Apr 26 to May 2, 2021	53	0–1	0.00–1.92	0–0	0.00–0.00
	May 3 to May 9, 2021	58	0–8	0.00–16.00	0–0	0.00–0.00
	May 10 to May 16, 2021	59	0–10	0.00–20.41	0–0	0.00–0.00
	May 17 to May 23, 2021	39	0–0	0.00–0.00	0–9	0.00–18.75
	May 24 to May 30, 2021	46	0–1	0.00–2.22	0–0	0.00–0.00
Hiroshima						
	Apr 5 to Apr 11, 2021	61	0–0	0.00–0.00	0–4	0.00–6.15
	Apr 12 to Apr 18, 2021	67	0–4	0.00–6.35	0–0	0.00–0.00
	Apr 19 to Apr 25, 2021	71	0–9	0.00–14.52	0–0	0.00–0.00
	Apr 26 to May 2, 2021	81	2–19	3.23–30.65	0–0	0.00–0.00
	May 3 to May 9, 2021	68	0–8	0.00–13.33	0–0	0.00–0.00
	May 10 to May 16, 2021	72	0–13	0.00–22.03	0–0	0.00–0.00
	May 17 to May 23, 2021	81	9–24	15.79–42.11	0–0	0.00–0.00
	May 24 to May 30, 2021	54	0–0	0.00–0.00	0–2	0.00–3.57
Yamaguchi						
	Apr 5 to Apr 11, 2021	47	0–5	0.00–11.90	0–0	0.00–0.00
	Apr 12 to Apr 18, 2021	41	0–0	0.00–0.00	0–0	0.00–0.00

	Apr 19 to Apr 25, 2021	49	0–9	0.00–22.50	0–0	0.00–0.00
	Apr 26 to May 2, 2021	65	11–25	27.50–62.50	0–0	0.00–0.00
	May 3 to May 9, 2021	42	0–3	0.00–7.69	0–0	0.00–0.00
	May 10 to May 16, 2021	39	0–0	0.00–0.00	0–0	0.00–0.00
	May 17 to May 23, 2021	48	0–9	0.00–23.08	0–0	0.00–0.00
	May 24 to May 30, 2021	39	0–1	0.00–2.63	0–0	0.00–0.00
Tokushima						
	Apr 5 to Apr 11, 2021	23	0–0	0.00–0.00	0–3	0.00–11.54
	Apr 12 to Apr 18, 2021	28	0–3	0.00–12.00	0–0	0.00–0.00
	Apr 19 to Apr 25, 2021	30	0–7	0.00–30.43	0–0	0.00–0.00
	Apr 26 to May 2, 2021	23	0–1	0.00–4.55	0–0	0.00–0.00
	May 3 to May 9, 2021	24	0–2	0.00–9.09	0–0	0.00–0.00
	May 10 to May 16, 2021	27	0–6	0.00–28.57	0–0	0.00–0.00
	May 17 to May 23, 2021	24	0–3	0.00–14.29	0–0	0.00–0.00
	May 24 to May 30, 2021	26	0–5	0.00–23.81	0–0	0.00–0.00
Kagawa						
	Apr 5 to Apr 11, 2021	24	0–0	0.00–0.00	0–4	0.00–14.29
	Apr 12 to Apr 18, 2021	28	0–0	0.00–0.00	0–0	0.00–0.00
	Apr 19 to Apr 25, 2021	20	0–0	0.00–0.00	0–7	0.00–25.93
	Apr 26 to May 2, 2021	25	0–0	0.00–0.00	0–1	0.00–3.85
	May 3 to May 9, 2021	26	0–0	0.00–0.00	0–0	0.00–0.00
	May 10 to May 16, 2021	28	0–4	0.00–16.67	0–0	0.00–0.00
	May 17 to May 23, 2021	23	0–0	0.00–0.00	0–2	0.00–8.00
	May 24 to May 30, 2021	27	0–4	0.00–17.39	0–0	0.00–0.00
Ehime						
	Apr 5 to Apr 11, 2021	45	0–9	0.00–25.00	0–0	0.00–0.00
	Apr 12 to Apr 18, 2021	30	0–0	0.00–0.00	0–6	0.00–16.67
	Apr 19 to Apr 25, 2021	39	0–4	0.00–11.43	0–0	0.00–0.00
	Apr 26 to May 2, 2021	40	0–5	0.00–14.29	0–0	0.00–0.00
	May 3 to May 9, 2021	23	0–0	0.00–0.00	1–12	2.86–34.29
	May 10 to May 16, 2021	47	1–13	2.94–38.24	0–0	0.00–0.00
	May 17 to May 23, 2021	31	0–0	0.00–0.00	0–2	0.00–6.06
	May 24 to May 30, 2021	28	0–0	0.00–0.00	0–4	0.00–12.50
Kochi						
	Apr 5 to Apr 11, 2021	26	0–1	0.00–4.00	0–0	0.00–0.00
	Apr 12 to Apr 18, 2021	22	0–0	0.00–0.00	0–3	0.00–12.00
	Apr 19 to Apr 25, 2021	17	0–0	0.00–0.00	0–7	0.00–29.17
	Apr 26 to May 2, 2021	14	0–0	0.00–0.00	0–9	0.00–39.13
	May 3 to May 9, 2021	20	0–0	0.00–0.00	0–2	0.00–9.09
	May 10 to May 16, 2021	32	0–10	0.00–45.45	0–0	0.00–0.00
	May 17 to May 23, 2021	28	0–7	0.00–33.33	0–0	0.00–0.00
	May 24 to May 30, 2021	23	0–3	0.00–15.00	0–0	0.00–0.00
Fukuoka						
	Apr 5 to Apr 11, 2021	126	0–0	0.00–0.00	0–2	0.00–1.56
	Apr 12 to Apr 18, 2021	121	0–0	0.00–0.00	0–5	0.00–3.97
	Apr 19 to Apr 25, 2021	162	11–37	8.80–29.60	0–0	0.00–0.00

	Apr 26 to May 2, 2021	124	0–2	0.00–1.64	0–0	0.00–0.00
	May 3 to May 9, 2021	141	0–22	0.00–18.49	0–0	0.00–0.00
	May 10 to May 16, 2021	135	0–17	0.00–14.41	0–0	0.00–0.00
	May 17 to May 23, 2021	135	0–18	0.00–15.38	0–0	0.00–0.00
	May 24 to May 30, 2021	132	0–19	0.00–16.81	0–0	0.00–0.00
Saga						
	Apr 5 to Apr 11, 2021	27	0–2	0.00–8.00	0–0	0.00–0.00
	Apr 12 to Apr 18, 2021	30	0–6	0.00–25.00	0–0	0.00–0.00
	Apr 19 to Apr 25, 2021	26	0–2	0.00–8.33	0–0	0.00–0.00
	Apr 26 to May 2, 2021	30	0–7	0.00–30.43	0–0	0.00–0.00
	May 3 to May 9, 2021	18	0–0	0.00–0.00	0–5	0.00–21.74
	May 10 to May 16, 2021	36	4–14	18.18–63.64	0–0	0.00–0.00
	May 17 to May 23, 2021	23	0–1	0.00–4.55	0–0	0.00–0.00
	May 24 to May 30, 2021	19	0–0	0.00–0.00	0–2	0.00–9.52
Nagasaki						
	Apr 5 to Apr 11, 2021	39	0–0	0.00–0.00	0–4	0.00–9.30
	Apr 12 to Apr 18, 2021	39	0–0	0.00–0.00	0–3	0.00–7.14
	Apr 19 to Apr 25, 2021	41	0–1	0.00–2.50	0–0	0.00–0.00
	Apr 26 to May 2, 2021	40	0–0	0.00–0.00	0–0	0.00–0.00
	May 3 to May 9, 2021	48	0–10	0.00–26.32	0–0	0.00–0.00
	May 10 to May 16, 2021	44	0–6	0.00–15.79	0–0	0.00–0.00
	May 17 to May 23, 2021	32	0–0	0.00–0.00	0–5	0.00–13.51
	May 24 to May 30, 2021	45	0–10	0.00–28.57	0–0	0.00–0.00
Kumamoto						
	Apr 5 to Apr 11, 2021	36	0–0	0.00–0.00	1–15	1.96–29.41
	Apr 12 to Apr 18, 2021	53	0–2	0.00–3.92	0–0	0.00–0.00
	Apr 19 to Apr 25, 2021	57	0–7	0.00–14.00	0–0	0.00–0.00
	Apr 26 to May 2, 2021	53	0–4	0.00–8.16	0–0	0.00–0.00
	May 3 to May 9, 2021	52	0–4	0.00–8.33	0–0	0.00–0.00
	May 10 to May 16, 2021	55	0–8	0.00–17.02	0–0	0.00–0.00
	May 17 to May 23, 2021	57	0–11	0.00–23.91	0–0	0.00–0.00
	May 24 to May 30, 2021	45	0–0	0.00–0.00	0–0	0.00–0.00
Oita						
	Apr 5 to Apr 11, 2021	32	0–0	0.00–0.00	0–0	0.00–0.00
	Apr 12 to Apr 18, 2021	40	0–9	0.00–29.03	0–0	0.00–0.00
	Apr 19 to Apr 25, 2021	37	0–6	0.00–19.35	0–0	0.00–0.00
	Apr 26 to May 2, 2021	34	0–4	0.00–13.33	0–0	0.00–0.00
	May 3 to May 9, 2021	33	0–3	0.00–10.00	0–0	0.00–0.00
	May 10 to May 16, 2021	37	0–7	0.00–23.33	0–0	0.00–0.00
	May 17 to May 23, 2021	23	0–0	0.00–0.00	0–6	0.00–20.69
	May 24 to May 30, 2021	23	0–0	0.00–0.00	0–4	0.00–14.81
Miyazaki						
	Apr 5 to Apr 11, 2021	20	0–0	0.00–0.00	0–10	0.00–33.33
	Apr 12 to Apr 18, 2021	31	0–1	0.00–3.33	0–0	0.00–0.00
	Apr 19 to Apr 25, 2021	37	0–7	0.00–23.33	0–0	0.00–0.00
	Apr 26 to May 2, 2021	36	0–6	0.00–20.00	0–0	0.00–0.00

	May 3 to May 9, 2021	36	0–6	0.00–20.00	0–0	0.00–0.00
	May 10 to May 16, 2021	25	0–0	0.00–0.00	0–4	0.00–13.79
	May 17 to May 23, 2021	27	0–0	0.00–0.00	0–1	0.00–3.57
	May 24 to May 30, 2021	27	0–0	0.00–0.00	0–0	0.00–0.00
Kagoshima						
	Apr 5 to Apr 11, 2021	55	0–3	0.00–5.77	0–0	0.00–0.00
	Apr 12 to Apr 18, 2021	37	0–0	0.00–0.00	0–14	0.00–27.45
	Apr 19 to Apr 25, 2021	48	0–0	0.00–0.00	0–1	0.00–2.04
	Apr 26 to May 2, 2021	37	0–0	0.00–0.00	0–11	0.00–22.92
	May 3 to May 9, 2021	52	0–4	0.00–8.33	0–0	0.00–0.00
	May 10 to May 16, 2021	41	0–0	0.00–0.00	0–6	0.00–12.77
	May 17 to May 23, 2021	48	0–2	0.00–4.35	0–0	0.00–0.00
	May 24 to May 30, 2021	51	0–7	0.00–15.91	0–0	0.00–0.00
Okinawa						
	Apr 5 to Apr 11, 2021	23	0–0	0.00–0.00	0–0	0.00–0.00
	Apr 12 to Apr 18, 2021	27	0–4	0.00–17.39	0–0	0.00–0.00
	Apr 19 to Apr 25, 2021	21	0–0	0.00–0.00	0–2	0.00–8.70
	Apr 26 to May 2, 2021	20	0–0	0.00–0.00	0–3	0.00–13.04
	May 3 to May 9, 2021	16	0–0	0.00–0.00	0–6	0.00–27.27
	May 10 to May 16, 2021	23	0–1	0.00–4.55	0–0	0.00–0.00
	May 17 to May 23, 2021	17	0–0	0.00–0.00	0–4	0.00–19.05
	May 24 to May 30, 2021	13	0–0	0.00–0.00	0–8	0.00–38.10

Table A.7: Weekly number of observed and excess/exiguous deaths in Japan and 47 prefectures for respiratory disease-related deaths in nursing homes and elderly care facilities from January 2020 through May 2021.

Prefecture	Week	Observed	Excess deaths	Percent excess	Exiguous deaths	Percent exiguous
Japan						
	Apr 5 to Apr 11, 2021	265	0–0	0.00–0.00	0–4	0.00–1.49
	Apr 12 to Apr 18, 2021	286	0–17	0.00–6.32	0–0	0.00–0.00
	Apr 19 to Apr 25, 2021	295	0–27	0.00–10.07	0–0	0.00–0.00
	Apr 26 to May 2, 2021	306	0–41	0.00–15.47	0–0	0.00–0.00
	May 3 to May 9, 2021	345	36–81	13.64–30.68	0–0	0.00–0.00
	May 10 to May 16, 2021	393	87–131	33.21–50.00	0–0	0.00–0.00
	May 17 to May 23, 2021	360	61–104	23.83–40.63	0–0	0.00–0.00
	May 24 to May 30, 2021	330	34–78	13.49–30.95	0–0	0.00–0.00
Hokkaido						
	Apr 5 to Apr 11, 2021	3	0–0	0.00–0.00	0–3	0.00–50.00
	Apr 12 to Apr 18, 2021	3	0–0	0.00–0.00	0–3	0.00–50.00
	Apr 19 to Apr 25, 2021	4	0–0	0.00–0.00	0–2	0.00–33.33
	Apr 26 to May 2, 2021	3	0–0	0.00–0.00	0–3	0.00–50.00
	May 3 to May 9, 2021	8	0–2	0.00–33.33	0–0	0.00–0.00
	May 10 to May 16, 2021	11	0–5	0.00–83.33	0–0	0.00–0.00
	May 17 to May 23, 2021	2	0–0	0.00–0.00	0–4	0.00–66.67
	May 24 to May 30, 2021	7	0–1	0.00–16.67	0–0	0.00–0.00
Aomori						
	Apr 5 to Apr 11, 2021	4	0–0	0.00–0.00	0–1	0.00–20.00
	Apr 12 to Apr 18, 2021	4	0–0	0.00–0.00	0–2	0.00–33.33
	Apr 19 to Apr 25, 2021	4	0–0	0.00–0.00	0–2	0.00–33.33
	Apr 26 to May 2, 2021	1	0–0	0.00–0.00	0–4	0.00–80.00
	May 3 to May 9, 2021	6	0–1	0.00–20.00	0–0	0.00–0.00
	May 10 to May 16, 2021	8	0–3	0.00–60.00	0–0	0.00–0.00
	May 17 to May 23, 2021	8	0–4	0.00–100.00	0–0	0.00–0.00
	May 24 to May 30, 2021	6	0–2	0.00–50.00	0–0	0.00–0.00
Iwate						
	Apr 5 to Apr 11, 2021	3	0–0	0.00–0.00	0–1	0.00–25.00
	Apr 12 to Apr 18, 2021	2	0–0	0.00–0.00	0–3	0.00–60.00
	Apr 19 to Apr 25, 2021	0	0–0	0.00–0.00	1–4	25.00–100.00
	Apr 26 to May 2, 2021	0	0–0	0.00–0.00	1–4	25.00–100.00
	May 3 to May 9, 2021	3	0–0	0.00–0.00	0–1	0.00–25.00
	May 10 to May 16, 2021	6	0–2	0.00–50.00	0–0	0.00–0.00
	May 17 to May 23, 2021	7	0–3	0.00–75.00	0–0	0.00–0.00
	May 24 to May 30, 2021	3	0–0	0.00–0.00	0–1	0.00–25.00
Miyagi						
	Apr 5 to Apr 11, 2021	4	0–0	0.00–0.00	0–0	0.00–0.00
	Apr 12 to Apr 18, 2021	8	0–4	0.00–100.00	0–0	0.00–0.00
	Apr 19 to Apr 25, 2021	6	0–2	0.00–50.00	0–0	0.00–0.00
	Apr 26 to May 2, 2021	3	0–0	0.00–0.00	0–1	0.00–25.00

	May 3 to May 9, 2021	8	0–3	0.00–60.00	0–0	0.00–0.00
	May 10 to May 16, 2021	2	0–0	0.00–0.00	0–2	0.00–50.00
	May 17 to May 23, 2021	7	0–3	0.00–75.00	0–0	0.00–0.00
	May 24 to May 30, 2021	3	0–0	0.00–0.00	0–1	0.00–25.00
Akita						
	Apr 5 to Apr 11, 2021	0	0–0	0.00–0.00	0–2	0.00–100.00
	Apr 12 to Apr 18, 2021	2	0–0	0.00–0.00	0–0	0.00–0.00
	Apr 19 to Apr 25, 2021	1	0–0	0.00–0.00	0–2	0.00–66.67
	Apr 26 to May 2, 2021	3	0–1	0.00–50.00	0–0	0.00–0.00
	May 3 to May 9, 2021	2	0–0	0.00–0.00	0–0	0.00–0.00
	May 10 to May 16, 2021	2	0–0	0.00–0.00	0–0	0.00–0.00
	May 17 to May 23, 2021	2	0–0	0.00–0.00	0–0	0.00–0.00
	May 24 to May 30, 2021	1	0–0	0.00–0.00	0–1	0.00–50.00
Yamagata						
	Apr 5 to Apr 11, 2021	4	0–1	0.00–33.33	0–0	0.00–0.00
	Apr 12 to Apr 18, 2021	2	0–0	0.00–0.00	0–2	0.00–50.00
	Apr 19 to Apr 25, 2021	8	0–4	0.00–100.00	0–0	0.00–0.00
	Apr 26 to May 2, 2021	1	0–0	0.00–0.00	0–3	0.00–75.00
	May 3 to May 9, 2021	8	0–4	0.00–100.00	0–0	0.00–0.00
	May 10 to May 16, 2021	6	0–2	0.00–50.00	0–0	0.00–0.00
	May 17 to May 23, 2021	7	0–3	0.00–75.00	0–0	0.00–0.00
	May 24 to May 30, 2021	2	0–0	0.00–0.00	0–1	0.00–33.33
Fukushima						
	Apr 5 to Apr 11, 2021	3	0–0	0.00–0.00	0–1	0.00–25.00
	Apr 12 to Apr 18, 2021	6	0–2	0.00–50.00	0–0	0.00–0.00
	Apr 19 to Apr 25, 2021	0	0–0	0.00–0.00	1–4	25.00–100.00
	Apr 26 to May 2, 2021	4	0–0	0.00–0.00	0–0	0.00–0.00
	May 3 to May 9, 2021	5	0–1	0.00–25.00	0–0	0.00–0.00
	May 10 to May 16, 2021	7	0–3	0.00–75.00	0–0	0.00–0.00
	May 17 to May 23, 2021	6	0–2	0.00–50.00	0–0	0.00–0.00
	May 24 to May 30, 2021	6	0–2	0.00–50.00	0–0	0.00–0.00
Ibaraki						
	Apr 5 to Apr 11, 2021	3	0–0	0.00–0.00	0–3	0.00–50.00
	Apr 12 to Apr 18, 2021	8	0–2	0.00–33.33	0–0	0.00–0.00
	Apr 19 to Apr 25, 2021	5	0–0	0.00–0.00	0–1	0.00–16.67
	Apr 26 to May 2, 2021	8	0–3	0.00–60.00	0–0	0.00–0.00
	May 3 to May 9, 2021	7	0–2	0.00–40.00	0–0	0.00–0.00
	May 10 to May 16, 2021	4	0–0	0.00–0.00	0–1	0.00–20.00
	May 17 to May 23, 2021	8	0–3	0.00–60.00	0–0	0.00–0.00
	May 24 to May 30, 2021	2	0–0	0.00–0.00	0–3	0.00–60.00
Tochigi						
	Apr 5 to Apr 11, 2021	3	0–0	0.00–0.00	0–2	0.00–40.00
	Apr 12 to Apr 18, 2021	4	0–0	0.00–0.00	0–1	0.00–20.00
	Apr 19 to Apr 25, 2021	5	0–0	0.00–0.00	0–0	0.00–0.00
	Apr 26 to May 2, 2021	8	0–3	0.00–60.00	0–0	0.00–0.00
	May 3 to May 9, 2021	4	0–0	0.00–0.00	0–0	0.00–0.00

	May 10 to May 16, 2021	9	1–5	25.00–125.00	0–0	0.00–0.00
	May 17 to May 23, 2021	5	0–1	0.00–25.00	0–0	0.00–0.00
	May 24 to May 30, 2021	4	0–0	0.00–0.00	0–0	0.00–0.00
Gunma						
	Apr 5 to Apr 11, 2021	8	0–3	0.00–60.00	0–0	0.00–0.00
	Apr 12 to Apr 18, 2021	8	0–3	0.00–60.00	0–0	0.00–0.00
	Apr 19 to Apr 25, 2021	11	1–5	16.67–83.33	0–0	0.00–0.00
	Apr 26 to May 2, 2021	8	0–2	0.00–33.33	0–0	0.00–0.00
	May 3 to May 9, 2021	3	0–0	0.00–0.00	0–3	0.00–50.00
	May 10 to May 16, 2021	16	6–10	100.00–166.67	0–0	0.00–0.00
	May 17 to May 23, 2021	4	0–0	0.00–0.00	0–1	0.00–20.00
	May 24 to May 30, 2021	12	2–7	40.00–140.00	0–0	0.00–0.00
Saitama						
	Apr 5 to Apr 11, 2021	8	0–0	0.00–0.00	0–4	0.00–33.33
	Apr 12 to Apr 18, 2021	9	0–0	0.00–0.00	0–2	0.00–18.18
	Apr 19 to Apr 25, 2021	12	0–0	0.00–0.00	0–0	0.00–0.00
	Apr 26 to May 2, 2021	12	0–0	0.00–0.00	0–0	0.00–0.00
	May 3 to May 9, 2021	18	0–7	0.00–63.64	0–0	0.00–0.00
	May 10 to May 16, 2021	11	0–0	0.00–0.00	0–0	0.00–0.00
	May 17 to May 23, 2021	9	0–0	0.00–0.00	0–1	0.00–10.00
	May 24 to May 30, 2021	12	0–2	0.00–20.00	0–0	0.00–0.00
Chiba						
	Apr 5 to Apr 11, 2021	11	0–1	0.00–10.00	0–0	0.00–0.00
	Apr 12 to Apr 18, 2021	12	0–2	0.00–20.00	0–0	0.00–0.00
	Apr 19 to Apr 25, 2021	7	0–0	0.00–0.00	0–3	0.00–30.00
	Apr 26 to May 2, 2021	9	0–0	0.00–0.00	0–1	0.00–10.00
	May 3 to May 9, 2021	14	0–4	0.00–40.00	0–0	0.00–0.00
	May 10 to May 16, 2021	11	0–1	0.00–10.00	0–0	0.00–0.00
	May 17 to May 23, 2021	8	0–0	0.00–0.00	0–1	0.00–11.11
	May 24 to May 30, 2021	9	0–0	0.00–0.00	0–0	0.00–0.00
Tokyo						
	Apr 5 to Apr 11, 2021	17	0–0	0.00–0.00	0–5	0.00–22.73
	Apr 12 to Apr 18, 2021	27	0–5	0.00–22.73	0–0	0.00–0.00
	Apr 19 to Apr 25, 2021	19	0–0	0.00–0.00	0–3	0.00–13.64
	Apr 26 to May 2, 2021	25	0–4	0.00–19.05	0–0	0.00–0.00
	May 3 to May 9, 2021	33	2–12	9.52–57.14	0–0	0.00–0.00
	May 10 to May 16, 2021	22	0–1	0.00–4.76	0–0	0.00–0.00
	May 17 to May 23, 2021	23	0–3	0.00–15.00	0–0	0.00–0.00
	May 24 to May 30, 2021	21	0–1	0.00–5.00	0–0	0.00–0.00
Kanagawa						
	Apr 5 to Apr 11, 2021	19	0–3	0.00–18.75	0–0	0.00–0.00
	Apr 12 to Apr 18, 2021	15	0–0	0.00–0.00	0–1	0.00–6.25
	Apr 19 to Apr 25, 2021	12	0–0	0.00–0.00	0–4	0.00–25.00
	Apr 26 to May 2, 2021	22	0–6	0.00–37.50	0–0	0.00–0.00
	May 3 to May 9, 2021	20	0–4	0.00–25.00	0–0	0.00–0.00
	May 10 to May 16, 2021	16	0–0	0.00–0.00	0–0	0.00–0.00

	May 17 to May 23, 2021	20	0–4	0.00–25.00	0–0	0.00–0.00
	May 24 to May 30, 2021	18	0–2	0.00–12.50	0–0	0.00–0.00
Niigata						
	Apr 5 to Apr 11, 2021	8	0–3	0.00–60.00	0–0	0.00–0.00
	Apr 12 to Apr 18, 2021	3	0–0	0.00–0.00	0–2	0.00–40.00
	Apr 19 to Apr 25, 2021	7	0–2	0.00–40.00	0–0	0.00–0.00
	Apr 26 to May 2, 2021	5	0–0	0.00–0.00	0–0	0.00–0.00
	May 3 to May 9, 2021	9	0–4	0.00–80.00	0–0	0.00–0.00
	May 10 to May 16, 2021	7	0–2	0.00–40.00	0–0	0.00–0.00
	May 17 to May 23, 2021	5	0–0	0.00–0.00	0–0	0.00–0.00
	May 24 to May 30, 2021	5	0–0	0.00–0.00	0–0	0.00–0.00
Toyama						
	Apr 5 to Apr 11, 2021	2	0–0	0.00–0.00	0–2	0.00–50.00
	Apr 12 to Apr 18, 2021	4	0–1	0.00–33.33	0–0	0.00–0.00
	Apr 19 to Apr 25, 2021	4	0–1	0.00–33.33	0–0	0.00–0.00
	Apr 26 to May 2, 2021	3	0–0	0.00–0.00	0–0	0.00–0.00
	May 3 to May 9, 2021	4	0–1	0.00–33.33	0–0	0.00–0.00
	May 10 to May 16, 2021	3	0–0	0.00–0.00	0–0	0.00–0.00
	May 17 to May 23, 2021	2	0–0	0.00–0.00	0–1	0.00–33.33
	May 24 to May 30, 2021	2	0–0	0.00–0.00	0–1	0.00–33.33
Ishikawa						
	Apr 5 to Apr 11, 2021	1	0–0	0.00–0.00	0–3	0.00–75.00
	Apr 12 to Apr 18, 2021	6	0–2	0.00–50.00	0–0	0.00–0.00
	Apr 19 to Apr 25, 2021	5	0–1	0.00–25.00	0–0	0.00–0.00
	Apr 26 to May 2, 2021	4	0–0	0.00–0.00	0–0	0.00–0.00
	May 3 to May 9, 2021	4	0–0	0.00–0.00	0–0	0.00–0.00
	May 10 to May 16, 2021	5	0–1	0.00–25.00	0–0	0.00–0.00
	May 17 to May 23, 2021	3	0–0	0.00–0.00	0–0	0.00–0.00
	May 24 to May 30, 2021	1	0–0	0.00–0.00	0–2	0.00–66.67
Fukui						
	Apr 5 to Apr 11, 2021	1	0–0	0.00–0.00	0–3	0.00–75.00
	Apr 12 to Apr 18, 2021	4	0–0	0.00–0.00	0–0	0.00–0.00
	Apr 19 to Apr 25, 2021	2	0–0	0.00–0.00	0–2	0.00–50.00
	Apr 26 to May 2, 2021	3	0–0	0.00–0.00	0–0	0.00–0.00
	May 3 to May 9, 2021	4	0–1	0.00–33.33	0–0	0.00–0.00
	May 10 to May 16, 2021	5	0–2	0.00–66.67	0–0	0.00–0.00
	May 17 to May 23, 2021	2	0–0	0.00–0.00	0–1	0.00–33.33
	May 24 to May 30, 2021	6	0–3	0.00–100.00	0–0	0.00–0.00
Yamanashi						
	Apr 5 to Apr 11, 2021	1	NA	NA	NA	NA
	Apr 12 to Apr 18, 2021	1	NA	NA	NA	NA
	Apr 19 to Apr 25, 2021	2	NA	NA	NA	NA
	Apr 26 to May 2, 2021	5	0–3	0.00–150.00	0–0	0.00–0.00
	May 3 to May 9, 2021	2	0–0	0.00–0.00	0–0	0.00–0.00
	May 10 to May 16, 2021	0	0–0	0.00–0.00	0–2	0.00–100.00
	May 17 to May 23, 2021	6	1–4	50.00–200.00	0–0	0.00–0.00

	May 24 to May 30, 2021	2	0–0	0.00–0.00	0–0	0.00–0.00
Nagano						
	Apr 5 to Apr 11, 2021	8	0–2	0.00–33.33	0–0	0.00–0.00
	Apr 12 to Apr 18, 2021	7	0–1	0.00–16.67	0–0	0.00–0.00
	Apr 19 to Apr 25, 2021	4	0–0	0.00–0.00	0–2	0.00–33.33
	Apr 26 to May 2, 2021	7	0–2	0.00–40.00	0–0	0.00–0.00
	May 3 to May 9, 2021	6	0–1	0.00–20.00	0–0	0.00–0.00
	May 10 to May 16, 2021	5	0–0	0.00–0.00	0–0	0.00–0.00
	May 17 to May 23, 2021	11	1–6	20.00–120.00	0–0	0.00–0.00
	May 24 to May 30, 2021	7	0–2	0.00–40.00	0–0	0.00–0.00
Gifu						
	Apr 5 to Apr 11, 2021	6	0–0	0.00–0.00	0–0	0.00–0.00
	Apr 12 to Apr 18, 2021	5	0–0	0.00–0.00	0–1	0.00–16.67
	Apr 19 to Apr 25, 2021	5	0–0	0.00–0.00	0–1	0.00–16.67
	Apr 26 to May 2, 2021	5	0–0	0.00–0.00	0–1	0.00–16.67
	May 3 to May 9, 2021	11	0–5	0.00–83.33	0–0	0.00–0.00
	May 10 to May 16, 2021	9	0–3	0.00–50.00	0–0	0.00–0.00
	May 17 to May 23, 2021	6	0–0	0.00–0.00	0–0	0.00–0.00
	May 24 to May 30, 2021	6	0–0	0.00–0.00	0–0	0.00–0.00
Shizuoka						
	Apr 5 to Apr 11, 2021	6	0–0	0.00–0.00	0–5	0.00–45.45
	Apr 12 to Apr 18, 2021	12	0–0	0.00–0.00	0–0	0.00–0.00
	Apr 19 to Apr 25, 2021	12	0–0	0.00–0.00	0–0	0.00–0.00
	Apr 26 to May 2, 2021	12	0–1	0.00–9.09	0–0	0.00–0.00
	May 3 to May 9, 2021	10	0–0	0.00–0.00	0–1	0.00–9.09
	May 10 to May 16, 2021	15	0–4	0.00–36.36	0–0	0.00–0.00
	May 17 to May 23, 2021	13	0–2	0.00–18.18	0–0	0.00–0.00
	May 24 to May 30, 2021	15	0–4	0.00–36.36	0–0	0.00–0.00
Aichi						
	Apr 5 to Apr 11, 2021	9	0–0	0.00–0.00	0–8	0.00–47.06
	Apr 12 to Apr 18, 2021	15	0–0	0.00–0.00	0–2	0.00–11.76
	Apr 19 to Apr 25, 2021	18	0–1	0.00–5.88	0–0	0.00–0.00
	Apr 26 to May 2, 2021	16	0–0	0.00–0.00	0–1	0.00–5.88
	May 3 to May 9, 2021	22	0–5	0.00–29.41	0–0	0.00–0.00
	May 10 to May 16, 2021	25	1–9	6.25–56.25	0–0	0.00–0.00
	May 17 to May 23, 2021	25	1–9	6.25–56.25	0–0	0.00–0.00
	May 24 to May 30, 2021	24	1–9	6.67–60.00	0–0	0.00–0.00
Mie						
	Apr 5 to Apr 11, 2021	9	0–3	0.00–50.00	0–0	0.00–0.00
	Apr 12 to Apr 18, 2021	1	0–0	0.00–0.00	0–4	0.00–80.00
	Apr 19 to Apr 25, 2021	4	0–0	0.00–0.00	0–1	0.00–20.00
	Apr 26 to May 2, 2021	4	0–0	0.00–0.00	0–1	0.00–20.00
	May 3 to May 9, 2021	7	0–2	0.00–40.00	0–0	0.00–0.00
	May 10 to May 16, 2021	10	0–5	0.00–100.00	0–0	0.00–0.00
	May 17 to May 23, 2021	10	0–5	0.00–100.00	0–0	0.00–0.00
	May 24 to May 30, 2021	5	0–0	0.00–0.00	0–0	0.00–0.00

Shiga						
	Apr 5 to Apr 11, 2021	5	0–2	0.00–66.67	0–0	0.00–0.00
	Apr 12 to Apr 18, 2021	4	0–2	0.00–100.00	0–0	0.00–0.00
	Apr 19 to Apr 25, 2021	1	0–0	0.00–0.00	0–1	0.00–50.00
	Apr 26 to May 2, 2021	4	0–2	0.00–100.00	0–0	0.00–0.00
	May 3 to May 9, 2021	2	0–0	0.00–0.00	0–0	0.00–0.00
	May 10 to May 16, 2021	2	0–0	0.00–0.00	0–0	0.00–0.00
	May 17 to May 23, 2021	6	1–4	50.00–200.00	0–0	0.00–0.00
	May 24 to May 30, 2021	6	0–4	0.00–200.00	0–0	0.00–0.00
Kyoto						
	Apr 5 to Apr 11, 2021	5	0–0	0.00–0.00	0–0	0.00–0.00
	Apr 12 to Apr 18, 2021	2	0–0	0.00–0.00	0–3	0.00–60.00
	Apr 19 to Apr 25, 2021	3	0–0	0.00–0.00	0–2	0.00–40.00
	Apr 26 to May 2, 2021	3	0–0	0.00–0.00	0–3	0.00–50.00
	May 3 to May 9, 2021	5	0–0	0.00–0.00	0–1	0.00–16.67
	May 10 to May 16, 2021	8	0–2	0.00–33.33	0–0	0.00–0.00
	May 17 to May 23, 2021	4	0–0	0.00–0.00	0–2	0.00–33.33
	May 24 to May 30, 2021	8	0–2	0.00–33.33	0–0	0.00–0.00
Osaka						
	Apr 5 to Apr 11, 2021	18	0–0	0.00–0.00	0–1	0.00–5.26
	Apr 12 to Apr 18, 2021	18	0–0	0.00–0.00	0–0	0.00–0.00
	Apr 19 to Apr 25, 2021	21	0–3	0.00–16.67	0–0	0.00–0.00
	Apr 26 to May 2, 2021	17	0–0	0.00–0.00	0–2	0.00–10.53
	May 3 to May 9, 2021	19	0–1	0.00–5.56	0–0	0.00–0.00
	May 10 to May 16, 2021	33	6–15	33.33–83.33	0–0	0.00–0.00
	May 17 to May 23, 2021	24	0–6	0.00–33.33	0–0	0.00–0.00
	May 24 to May 30, 2021	22	0–5	0.00–29.41	0–0	0.00–0.00
Hyogo						
	Apr 5 to Apr 11, 2021	6	0–0	0.00–0.00	0–4	0.00–40.00
	Apr 12 to Apr 18, 2021	12	0–2	0.00–20.00	0–0	0.00–0.00
	Apr 19 to Apr 25, 2021	12	0–2	0.00–20.00	0–0	0.00–0.00
	Apr 26 to May 2, 2021	11	0–1	0.00–10.00	0–0	0.00–0.00
	May 3 to May 9, 2021	11	0–1	0.00–10.00	0–0	0.00–0.00
	May 10 to May 16, 2021	14	0–4	0.00–40.00	0–0	0.00–0.00
	May 17 to May 23, 2021	20	3–9	27.27–81.82	0–0	0.00–0.00
	May 24 to May 30, 2021	12	0–2	0.00–20.00	0–0	0.00–0.00
Nara						
	Apr 5 to Apr 11, 2021	3	0–0	0.00–0.00	0–1	0.00–25.00
	Apr 12 to Apr 18, 2021	8	0–4	0.00–100.00	0–0	0.00–0.00
	Apr 19 to Apr 25, 2021	6	0–2	0.00–50.00	0–0	0.00–0.00
	Apr 26 to May 2, 2021	6	0–3	0.00–100.00	0–0	0.00–0.00
	May 3 to May 9, 2021	2	0–0	0.00–0.00	0–2	0.00–50.00
	May 10 to May 16, 2021	2	0–0	0.00–0.00	0–1	0.00–33.33
	May 17 to May 23, 2021	9	1–6	33.33–200.00	0–0	0.00–0.00
	May 24 to May 30, 2021	0	0–0	0.00–0.00	0–4	0.00–100.00
Wakayama						

	Apr 5 to Apr 11, 2021	4	0–0	0.00–0.00	0–0	0.00–0.00
	Apr 12 to Apr 18, 2021	7	0–3	0.00–75.00	0–0	0.00–0.00
	Apr 19 to Apr 25, 2021	3	0–0	0.00–0.00	0–1	0.00–25.00
	Apr 26 to May 2, 2021	3	0–0	0.00–0.00	0–1	0.00–25.00
	May 3 to May 9, 2021	3	0–0	0.00–0.00	0–1	0.00–25.00
	May 10 to May 16, 2021	7	0–3	0.00–75.00	0–0	0.00–0.00
	May 17 to May 23, 2021	6	0–2	0.00–50.00	0–0	0.00–0.00
	May 24 to May 30, 2021	6	0–3	0.00–100.00	0–0	0.00–0.00
Tottori						
	Apr 5 to Apr 11, 2021	5	0–3	0.00–150.00	0–0	0.00–0.00
	Apr 12 to Apr 18, 2021	2	0–0	0.00–0.00	0–1	0.00–33.33
	Apr 19 to Apr 25, 2021	0	0–0	0.00–0.00	0–3	0.00–100.00
	Apr 26 to May 2, 2021	0	0–0	0.00–0.00	0–3	0.00–100.00
	May 3 to May 9, 2021	2	NA	NA	NA	NA
	May 10 to May 16, 2021	9	3–6	100.00–200.00	0–0	0.00–0.00
	May 17 to May 23, 2021	4	0–1	0.00–33.33	0–0	0.00–0.00
	May 24 to May 30, 2021	2	0–0	0.00–0.00	0–0	0.00–0.00
Shimane						
	Apr 5 to Apr 11, 2021	1	0–0	0.00–0.00	0–2	0.00–66.67
	Apr 12 to Apr 18, 2021	4	0–1	0.00–33.33	0–0	0.00–0.00
	Apr 19 to Apr 25, 2021	1	0–0	0.00–0.00	0–2	0.00–66.67
	Apr 26 to May 2, 2021	8	2–6	100.00–300.00	0–0	0.00–0.00
	May 3 to May 9, 2021	0	0–0	0.00–0.00	0–3	0.00–100.00
	May 10 to May 16, 2021	4	0–2	0.00–100.00	0–0	0.00–0.00
	May 17 to May 23, 2021	8	2–6	100.00–300.00	0–0	0.00–0.00
	May 24 to May 30, 2021	7	1–5	50.00–250.00	0–0	0.00–0.00
Okayama						
	Apr 5 to Apr 11, 2021	5	0–0	0.00–0.00	0–1	0.00–16.67
	Apr 12 to Apr 18, 2021	3	0–0	0.00–0.00	0–3	0.00–50.00
	Apr 19 to Apr 25, 2021	8	0–2	0.00–33.33	0–0	0.00–0.00
	Apr 26 to May 2, 2021	5	0–0	0.00–0.00	0–1	0.00–16.67
	May 3 to May 9, 2021	2	0–0	0.00–0.00	0–4	0.00–66.67
	May 10 to May 16, 2021	9	0–3	0.00–50.00	0–0	0.00–0.00
	May 17 to May 23, 2021	4	0–0	0.00–0.00	0–2	0.00–33.33
	May 24 to May 30, 2021	5	0–0	0.00–0.00	0–1	0.00–16.67
Hiroshima						
	Apr 5 to Apr 11, 2021	8	0–0	0.00–0.00	0–0	0.00–0.00
	Apr 12 to Apr 18, 2021	3	0–0	0.00–0.00	0–5	0.00–62.50
	Apr 19 to Apr 25, 2021	8	0–0	0.00–0.00	0–0	0.00–0.00
	Apr 26 to May 2, 2021	7	0–0	0.00–0.00	0–1	0.00–12.50
	May 3 to May 9, 2021	7	0–0	0.00–0.00	0–0	0.00–0.00
	May 10 to May 16, 2021	7	0–0	0.00–0.00	0–0	0.00–0.00
	May 17 to May 23, 2021	12	1–6	16.67–100.00	0–0	0.00–0.00
	May 24 to May 30, 2021	8	0–2	0.00–33.33	0–0	0.00–0.00
Yamaguchi						
	Apr 5 to Apr 11, 2021	4	0–0	0.00–0.00	0–1	0.00–20.00

	Apr 12 to Apr 18, 2021	4	0–0	0.00–0.00	0–1	0.00–20.00
	Apr 19 to Apr 25, 2021	5	0–0	0.00–0.00	0–0	0.00–0.00
	Apr 26 to May 2, 2021	4	0–0	0.00–0.00	0–1	0.00–20.00
	May 3 to May 9, 2021	5	0–0	0.00–0.00	0–0	0.00–0.00
	May 10 to May 16, 2021	7	0–2	0.00–40.00	0–0	0.00–0.00
	May 17 to May 23, 2021	10	0–5	0.00–100.00	0–0	0.00–0.00
	May 24 to May 30, 2021	7	0–2	0.00–40.00	0–0	0.00–0.00
Tokushima						
	Apr 5 to Apr 11, 2021	3	0–0	0.00–0.00	0–2	0.00–40.00
	Apr 12 to Apr 18, 2021	5	0–1	0.00–25.00	0–0	0.00–0.00
	Apr 19 to Apr 25, 2021	5	0–1	0.00–25.00	0–0	0.00–0.00
	Apr 26 to May 2, 2021	4	0–0	0.00–0.00	0–0	0.00–0.00
	May 3 to May 9, 2021	5	0–0	0.00–0.00	0–0	0.00–0.00
	May 10 to May 16, 2021	6	0–1	0.00–20.00	0–0	0.00–0.00
	May 17 to May 23, 2021	4	0–0	0.00–0.00	0–1	0.00–20.00
	May 24 to May 30, 2021	5	0–0	0.00–0.00	0–0	0.00–0.00
Kagawa						
	Apr 5 to Apr 11, 2021	8	0–3	0.00–60.00	0–0	0.00–0.00
	Apr 12 to Apr 18, 2021	6	0–1	0.00–20.00	0–0	0.00–0.00
	Apr 19 to Apr 25, 2021	5	0–0	0.00–0.00	0–0	0.00–0.00
	Apr 26 to May 2, 2021	7	0–2	0.00–40.00	0–0	0.00–0.00
	May 3 to May 9, 2021	5	0–0	0.00–0.00	0–0	0.00–0.00
	May 10 to May 16, 2021	8	0–3	0.00–60.00	0–0	0.00–0.00
	May 17 to May 23, 2021	7	0–2	0.00–40.00	0–0	0.00–0.00
	May 24 to May 30, 2021	4	0–0	0.00–0.00	0–1	0.00–20.00
Ehime						
	Apr 5 to Apr 11, 2021	4	0–1	0.00–33.33	0–0	0.00–0.00
	Apr 12 to Apr 18, 2021	4	0–1	0.00–33.33	0–0	0.00–0.00
	Apr 19 to Apr 25, 2021	3	0–0	0.00–0.00	0–0	0.00–0.00
	Apr 26 to May 2, 2021	6	0–3	0.00–100.00	0–0	0.00–0.00
	May 3 to May 9, 2021	7	0–4	0.00–133.33	0–0	0.00–0.00
	May 10 to May 16, 2021	7	0–4	0.00–133.33	0–0	0.00–0.00
	May 17 to May 23, 2021	0	0–0	0.00–0.00	0–3	0.00–100.00
	May 24 to May 30, 2021	4	0–1	0.00–33.33	0–0	0.00–0.00
Kochi						
	Apr 5 to Apr 11, 2021	5	0–3	0.00–150.00	0–0	0.00–0.00
	Apr 12 to Apr 18, 2021	4	0–2	0.00–100.00	0–0	0.00–0.00
	Apr 19 to Apr 25, 2021	2	0–0	0.00–0.00	0–0	0.00–0.00
	Apr 26 to May 2, 2021	3	0–1	0.00–50.00	0–0	0.00–0.00
	May 3 to May 9, 2021	1	0–0	0.00–0.00	0–1	0.00–50.00
	May 10 to May 16, 2021	0	0–0	0.00–0.00	0–2	0.00–100.00
	May 17 to May 23, 2021	3	0–1	0.00–50.00	0–0	0.00–0.00
	May 24 to May 30, 2021	1	0–0	0.00–0.00	0–1	0.00–50.00
Fukuoka						
	Apr 5 to Apr 11, 2021	12	0–3	0.00–33.33	0–0	0.00–0.00
	Apr 12 to Apr 18, 2021	9	0–0	0.00–0.00	0–0	0.00–0.00

	Apr 19 to Apr 25, 2021	15	0–6	0.00–66.67	0–0	0.00–0.00
	Apr 26 to May 2, 2021	19	4–10	44.44–111.11	0–0	0.00–0.00
	May 3 to May 9, 2021	8	0–0	0.00–0.00	0–1	0.00–11.11
	May 10 to May 16, 2021	12	0–3	0.00–33.33	0–0	0.00–0.00
	May 17 to May 23, 2021	15	0–6	0.00–66.67	0–0	0.00–0.00
	May 24 to May 30, 2021	15	0–5	0.00–50.00	0–0	0.00–0.00
Saga						
	Apr 5 to Apr 11, 2021	3	0–0	0.00–0.00	0–0	0.00–0.00
	Apr 12 to Apr 18, 2021	1	0–0	0.00–0.00	0–2	0.00–66.67
	Apr 19 to Apr 25, 2021	3	0–0	0.00–0.00	0–0	0.00–0.00
	Apr 26 to May 2, 2021	1	0–0	0.00–0.00	0–2	0.00–66.67
	May 3 to May 9, 2021	9	2–6	66.67–200.00	0–0	0.00–0.00
	May 10 to May 16, 2021	5	0–2	0.00–66.67	0–0	0.00–0.00
	May 17 to May 23, 2021	2	0–0	0.00–0.00	0–1	0.00–33.33
	May 24 to May 30, 2021	2	0–0	0.00–0.00	0–1	0.00–33.33
Nagasaki						
	Apr 5 to Apr 11, 2021	1	0–0	0.00–0.00	0–4	0.00–80.00
	Apr 12 to Apr 18, 2021	2	0–0	0.00–0.00	0–3	0.00–60.00
	Apr 19 to Apr 25, 2021	5	0–0	0.00–0.00	0–0	0.00–0.00
	Apr 26 to May 2, 2021	3	0–0	0.00–0.00	0–2	0.00–40.00
	May 3 to May 9, 2021	6	0–1	0.00–20.00	0–0	0.00–0.00
	May 10 to May 16, 2021	11	1–6	20.00–120.00	0–0	0.00–0.00
	May 17 to May 23, 2021	2	0–0	0.00–0.00	0–3	0.00–60.00
	May 24 to May 30, 2021	4	0–0	0.00–0.00	0–1	0.00–20.00
Kumamoto						
	Apr 5 to Apr 11, 2021	5	0–0	0.00–0.00	0–1	0.00–16.67
	Apr 12 to Apr 18, 2021	4	0–0	0.00–0.00	0–2	0.00–33.33
	Apr 19 to Apr 25, 2021	8	0–1	0.00–14.29	0–0	0.00–0.00
	Apr 26 to May 2, 2021	7	0–0	0.00–0.00	0–0	0.00–0.00
	May 3 to May 9, 2021	9	0–3	0.00–50.00	0–0	0.00–0.00
	May 10 to May 16, 2021	9	0–2	0.00–28.57	0–0	0.00–0.00
	May 17 to May 23, 2021	6	0–0	0.00–0.00	0–1	0.00–14.29
	May 24 to May 30, 2021	9	0–3	0.00–50.00	0–0	0.00–0.00
Oita						
	Apr 5 to Apr 11, 2021	4	0–0	0.00–0.00	0–0	0.00–0.00
	Apr 12 to Apr 18, 2021	5	0–1	0.00–25.00	0–0	0.00–0.00
	Apr 19 to Apr 25, 2021	7	0–3	0.00–75.00	0–0	0.00–0.00
	Apr 26 to May 2, 2021	4	0–0	0.00–0.00	0–0	0.00–0.00
	May 3 to May 9, 2021	3	0–0	0.00–0.00	0–1	0.00–25.00
	May 10 to May 16, 2021	5	0–1	0.00–25.00	0–0	0.00–0.00
	May 17 to May 23, 2021	1	0–0	0.00–0.00	0–3	0.00–75.00
	May 24 to May 30, 2021	4	0–0	0.00–0.00	0–0	0.00–0.00
Miyazaki						
	Apr 5 to Apr 11, 2021	2	0–0	0.00–0.00	0–4	0.00–66.67
	Apr 12 to Apr 18, 2021	3	0–0	0.00–0.00	0–2	0.00–40.00
	Apr 19 to Apr 25, 2021	6	0–1	0.00–20.00	0–0	0.00–0.00

	Apr 26 to May 2, 2021	7	0–2	0.00–40.00	0–0	0.00–0.00
	May 3 to May 9, 2021	8	0–3	0.00–60.00	0–0	0.00–0.00
	May 10 to May 16, 2021	6	0–0	0.00–0.00	0–0	0.00–0.00
	May 17 to May 23, 2021	6	0–0	0.00–0.00	0–0	0.00–0.00
	May 24 to May 30, 2021	6	0–1	0.00–20.00	0–0	0.00–0.00
Kagoshima						
	Apr 5 to Apr 11, 2021	9	0–2	0.00–28.57	0–0	0.00–0.00
	Apr 12 to Apr 18, 2021	8	0–1	0.00–14.29	0–0	0.00–0.00
	Apr 19 to Apr 25, 2021	10	0–4	0.00–66.67	0–0	0.00–0.00
	Apr 26 to May 2, 2021	5	0–0	0.00–0.00	0–2	0.00–28.57
	May 3 to May 9, 2021	5	0–0	0.00–0.00	0–2	0.00–28.57
	May 10 to May 16, 2021	5	0–0	0.00–0.00	0–2	0.00–28.57
	May 17 to May 23, 2021	6	0–0	0.00–0.00	0–1	0.00–14.29
	May 24 to May 30, 2021	11	0–5	0.00–83.33	0–0	0.00–0.00
Okinawa						
	Apr 5 to Apr 11, 2021	4	0–0	0.00–0.00	0–0	0.00–0.00
	Apr 12 to Apr 18, 2021	0	0–0	0.00–0.00	1–4	25.00–100.00
	Apr 19 to Apr 25, 2021	1	0–0	0.00–0.00	0–3	0.00–75.00
	Apr 26 to May 2, 2021	1	0–0	0.00–0.00	0–3	0.00–75.00
	May 3 to May 9, 2021	3	0–0	0.00–0.00	0–1	0.00–25.00
	May 10 to May 16, 2021	2	0–0	0.00–0.00	0–2	0.00–50.00
	May 17 to May 23, 2021	2	0–0	0.00–0.00	0–2	0.00–50.00
	May 24 to May 30, 2021	3	0–0	0.00–0.00	0–0	0.00–0.00

Table A.8: Weekly number of observed and excess/exiguous deaths in Japan and 47 prefectures for respiratory disease-related deaths at homes from January 2020 through May 2021.

Prefecture	Week	Observed	Excess deaths	Percent excess	Exiguous deaths	Percent exiguous
Japan						
	Apr 5 to Apr 11, 2021	199	0–25	0.00–14.37	0–0	0.00–0.00
	Apr 12 to Apr 18, 2021	222	13–50	7.56–29.07	0–0	0.00–0.00
	Apr 19 to Apr 25, 2021	220	8–46	4.60–26.44	0–0	0.00–0.00
	Apr 26 to May 2, 2021	218	11–47	6.43–27.49	0–0	0.00–0.00
	May 3 to May 9, 2021	238	36–69	21.30–40.83	0–0	0.00–0.00
	May 10 to May 16, 2021	240	42–77	25.77–47.24	0–0	0.00–0.00
	May 17 to May 23, 2021	237	42–77	26.25–48.13	0–0	0.00–0.00
	May 24 to May 30, 2021	228	33–69	20.75–43.40	0–0	0.00–0.00
Hokkaido						
	Apr 5 to Apr 11, 2021	6	0–0	0.00–0.00	0–0	0.00–0.00
	Apr 12 to Apr 18, 2021	7	0–2	0.00–40.00	0–0	0.00–0.00
	Apr 19 to Apr 25, 2021	5	0–0	0.00–0.00	0–0	0.00–0.00
	Apr 26 to May 2, 2021	5	0–0	0.00–0.00	0–0	0.00–0.00
	May 3 to May 9, 2021	11	2–6	40.00–120.00	0–0	0.00–0.00
	May 10 to May 16, 2021	7	0–2	0.00–40.00	0–0	0.00–0.00
	May 17 to May 23, 2021	5	0–0	0.00–0.00	0–0	0.00–0.00
	May 24 to May 30, 2021	7	0–2	0.00–40.00	0–0	0.00–0.00
Aomori						
	Apr 5 to Apr 11, 2021	2	0–0	0.00–0.00	0–1	0.00–33.33
	Apr 12 to Apr 18, 2021	1	0–0	0.00–0.00	0–2	0.00–66.67
	Apr 19 to Apr 25, 2021	2	0–0	0.00–0.00	0–1	0.00–33.33
	Apr 26 to May 2, 2021	6	0–3	0.00–100.00	0–0	0.00–0.00
	May 3 to May 9, 2021	3	0–0	0.00–0.00	0–0	0.00–0.00
	May 10 to May 16, 2021	5	0–2	0.00–66.67	0–0	0.00–0.00
	May 17 to May 23, 2021	5	0–2	0.00–66.67	0–0	0.00–0.00
	May 24 to May 30, 2021	0	0–0	0.00–0.00	0–3	0.00–100.00
Iwate						
	Apr 5 to Apr 11, 2021	1	0–0	0.00–0.00	0–1	0.00–50.00
	Apr 12 to Apr 18, 2021	0	NA	NA	NA	NA
	Apr 19 to Apr 25, 2021	1	NA	NA	NA	NA
	Apr 26 to May 2, 2021	1	NA	NA	NA	NA
	May 3 to May 9, 2021	2	NA	NA	NA	NA
	May 10 to May 16, 2021	0	NA	NA	NA	NA
	May 17 to May 23, 2021	2	0–0	0.00–0.00	0–0	0.00–0.00
	May 24 to May 30, 2021	2	0–0	0.00–0.00	0–0	0.00–0.00
Miyagi						
	Apr 5 to Apr 11, 2021	8	1–5	33.33–166.67	0–0	0.00–0.00
	Apr 12 to Apr 18, 2021	7	0–4	0.00–133.33	0–0	0.00–0.00
	Apr 19 to Apr 25, 2021	3	0–0	0.00–0.00	0–0	0.00–0.00
	Apr 26 to May 2, 2021	4	0–1	0.00–33.33	0–0	0.00–0.00
	May 3 to May 9, 2021	4	0–1	0.00–33.33	0–0	0.00–0.00

	May 10 to May 16, 2021	4	0–1	0.00–33.33	0–0	0.00–0.00
	May 17 to May 23, 2021	6	0–3	0.00–100.00	0–0	0.00–0.00
	May 24 to May 30, 2021	4	0–1	0.00–33.33	0–0	0.00–0.00
Akita						
	Apr 5 to Apr 11, 2021	0	NA	NA	NA	NA
	Apr 12 to Apr 18, 2021	2	NA	NA	NA	NA
	Apr 19 to Apr 25, 2021	0	NA	NA	NA	NA
	Apr 26 to May 2, 2021	0	NA	NA	NA	NA
	May 3 to May 9, 2021	2	NA	NA	NA	NA
	May 10 to May 16, 2021	2	NA	NA	NA	NA
	May 17 to May 23, 2021	0	NA	NA	NA	NA
	May 24 to May 30, 2021	0	NA	NA	NA	NA
Yamagata						
	Apr 5 to Apr 11, 2021	0	NA	NA	NA	NA
	Apr 12 to Apr 18, 2021	0	NA	NA	NA	NA
	Apr 19 to Apr 25, 2021	2	NA	NA	NA	NA
	Apr 26 to May 2, 2021	0	NA	NA	NA	NA
	May 3 to May 9, 2021	2	NA	NA	NA	NA
	May 10 to May 16, 2021	0	NA	NA	NA	NA
	May 17 to May 23, 2021	2	NA	NA	NA	NA
	May 24 to May 30, 2021	4	0–2	0.00–100.00	0–0	0.00–0.00
Fukushima						
	Apr 5 to Apr 11, 2021	2	0–0	0.00–0.00	0–1	0.00–33.33
	Apr 12 to Apr 18, 2021	8	1–5	33.33–166.67	0–0	0.00–0.00
	Apr 19 to Apr 25, 2021	3	0–0	0.00–0.00	0–0	0.00–0.00
	Apr 26 to May 2, 2021	5	0–2	0.00–66.67	0–0	0.00–0.00
	May 3 to May 9, 2021	3	0–0	0.00–0.00	0–0	0.00–0.00
	May 10 to May 16, 2021	3	0–0	0.00–0.00	0–0	0.00–0.00
	May 17 to May 23, 2021	4	0–1	0.00–33.33	0–0	0.00–0.00
	May 24 to May 30, 2021	3	0–0	0.00–0.00	0–0	0.00–0.00
Ibaraki						
	Apr 5 to Apr 11, 2021	4	0–0	0.00–0.00	0–0	0.00–0.00
	Apr 12 to Apr 18, 2021	2	0–0	0.00–0.00	0–2	0.00–50.00
	Apr 19 to Apr 25, 2021	7	0–3	0.00–75.00	0–0	0.00–0.00
	Apr 26 to May 2, 2021	4	0–0	0.00–0.00	0–0	0.00–0.00
	May 3 to May 9, 2021	3	0–0	0.00–0.00	0–1	0.00–25.00
	May 10 to May 16, 2021	6	0–2	0.00–50.00	0–0	0.00–0.00
	May 17 to May 23, 2021	4	0–1	0.00–33.33	0–0	0.00–0.00
	May 24 to May 30, 2021	3	0–0	0.00–0.00	0–0	0.00–0.00
Tochigi						
	Apr 5 to Apr 11, 2021	3	0–0	0.00–0.00	0–0	0.00–0.00
	Apr 12 to Apr 18, 2021	3	0–1	0.00–50.00	0–0	0.00–0.00
	Apr 19 to Apr 25, 2021	5	0–3	0.00–150.00	0–0	0.00–0.00
	Apr 26 to May 2, 2021	5	0–3	0.00–150.00	0–0	0.00–0.00
	May 3 to May 9, 2021	2	0–0	0.00–0.00	0–0	0.00–0.00
	May 10 to May 16, 2021	2	0–0	0.00–0.00	0–0	0.00–0.00

	May 17 to May 23, 2021	0	0–0	0.00–0.00	0–2	0.00–100.00
	May 24 to May 30, 2021	7	1–5	50.00–250.00	0–0	0.00–0.00
Gunma						
	Apr 5 to Apr 11, 2021	3	0–0	0.00–0.00	0–0	0.00–0.00
	Apr 12 to Apr 18, 2021	1	0–0	0.00–0.00	0–2	0.00–66.67
	Apr 19 to Apr 25, 2021	0	0–0	0.00–0.00	0–3	0.00–100.00
	Apr 26 to May 2, 2021	4	0–1	0.00–33.33	0–0	0.00–0.00
	May 3 to May 9, 2021	8	1–5	33.33–166.67	0–0	0.00–0.00
	May 10 to May 16, 2021	4	0–1	0.00–33.33	0–0	0.00–0.00
	May 17 to May 23, 2021	3	0–0	0.00–0.00	0–0	0.00–0.00
	May 24 to May 30, 2021	5	0–2	0.00–66.67	0–0	0.00–0.00
Saitama						
	Apr 5 to Apr 11, 2021	12	0–3	0.00–33.33	0–0	0.00–0.00
	Apr 12 to Apr 18, 2021	12	0–3	0.00–33.33	0–0	0.00–0.00
	Apr 19 to Apr 25, 2021	8	0–0	0.00–0.00	0–1	0.00–11.11
	Apr 26 to May 2, 2021	16	1–7	11.11–77.78	0–0	0.00–0.00
	May 3 to May 9, 2021	12	0–3	0.00–33.33	0–0	0.00–0.00
	May 10 to May 16, 2021	7	0–0	0.00–0.00	0–1	0.00–12.50
	May 17 to May 23, 2021	14	0–6	0.00–75.00	0–0	0.00–0.00
	May 24 to May 30, 2021	14	0–6	0.00–75.00	0–0	0.00–0.00
Chiba						
	Apr 5 to Apr 11, 2021	10	0–1	0.00–11.11	0–0	0.00–0.00
	Apr 12 to Apr 18, 2021	9	0–0	0.00–0.00	0–1	0.00–10.00
	Apr 19 to Apr 25, 2021	14	0–4	0.00–40.00	0–0	0.00–0.00
	Apr 26 to May 2, 2021	12	0–3	0.00–33.33	0–0	0.00–0.00
	May 3 to May 9, 2021	13	0–4	0.00–44.44	0–0	0.00–0.00
	May 10 to May 16, 2021	13	0–4	0.00–44.44	0–0	0.00–0.00
	May 17 to May 23, 2021	6	0–0	0.00–0.00	0–3	0.00–33.33
	May 24 to May 30, 2021	9	0–0	0.00–0.00	0–0	0.00–0.00
Tokyo						
	Apr 5 to Apr 11, 2021	20	0–0	0.00–0.00	0–4	0.00–16.67
	Apr 12 to Apr 18, 2021	31	0–7	0.00–29.17	0–0	0.00–0.00
	Apr 19 to Apr 25, 2021	25	0–0	0.00–0.00	0–0	0.00–0.00
	Apr 26 to May 2, 2021	19	0–0	0.00–0.00	0–5	0.00–20.83
	May 3 to May 9, 2021	34	1–11	4.35–47.83	0–0	0.00–0.00
	May 10 to May 16, 2021	26	0–3	0.00–13.04	0–0	0.00–0.00
	May 17 to May 23, 2021	22	0–0	0.00–0.00	0–0	0.00–0.00
	May 24 to May 30, 2021	29	0–7	0.00–31.82	0–0	0.00–0.00
Kanagawa						
	Apr 5 to Apr 11, 2021	19	0–3	0.00–18.75	0–0	0.00–0.00
	Apr 12 to Apr 18, 2021	20	0–3	0.00–17.65	0–0	0.00–0.00
	Apr 19 to Apr 25, 2021	26	0–9	0.00–52.94	0–0	0.00–0.00
	Apr 26 to May 2, 2021	20	0–3	0.00–17.65	0–0	0.00–0.00
	May 3 to May 9, 2021	13	0–0	0.00–0.00	0–3	0.00–18.75
	May 10 to May 16, 2021	20	0–4	0.00–25.00	0–0	0.00–0.00
	May 17 to May 23, 2021	21	0–5	0.00–31.25	0–0	0.00–0.00

	May 24 to May 30, 2021	19	0–4	0.00–26.67	0–0	0.00–0.00
Niigata						
	Apr 5 to Apr 11, 2021	2	0–0	0.00–0.00	0–1	0.00–33.33
	Apr 12 to Apr 18, 2021	2	0–0	0.00–0.00	0–1	0.00–33.33
	Apr 19 to Apr 25, 2021	3	0–0	0.00–0.00	0–0	0.00–0.00
	Apr 26 to May 2, 2021	2	0–0	0.00–0.00	0–1	0.00–33.33
	May 3 to May 9, 2021	2	0–0	0.00–0.00	0–1	0.00–33.33
	May 10 to May 16, 2021	4	0–2	0.00–100.00	0–0	0.00–0.00
	May 17 to May 23, 2021	2	0–0	0.00–0.00	0–0	0.00–0.00
	May 24 to May 30, 2021	2	0–0	0.00–0.00	0–0	0.00–0.00
Toyama						
	Apr 5 to Apr 11, 2021	0	0–0	0.00–0.00	0–2	0.00–100.00
	Apr 12 to Apr 18, 2021	2	0–0	0.00–0.00	0–0	0.00–0.00
	Apr 19 to Apr 25, 2021	3	0–1	0.00–50.00	0–0	0.00–0.00
	Apr 26 to May 2, 2021	1	0–0	0.00–0.00	0–1	0.00–50.00
	May 3 to May 9, 2021	1	0–0	0.00–0.00	0–1	0.00–50.00
	May 10 to May 16, 2021	1	0–0	0.00–0.00	0–1	0.00–50.00
	May 17 to May 23, 2021	1	NA	NA	NA	NA
	May 24 to May 30, 2021	0	NA	NA	NA	NA
Ishikawa						
	Apr 5 to Apr 11, 2021	3	0–1	0.00–50.00	0–0	0.00–0.00
	Apr 12 to Apr 18, 2021	3	0–1	0.00–50.00	0–0	0.00–0.00
	Apr 19 to Apr 25, 2021	2	0–0	0.00–0.00	0–0	0.00–0.00
	Apr 26 to May 2, 2021	0	0–0	0.00–0.00	0–2	0.00–100.00
	May 3 to May 9, 2021	1	0–0	0.00–0.00	0–1	0.00–50.00
	May 10 to May 16, 2021	0	NA	NA	NA	NA
	May 17 to May 23, 2021	0	NA	NA	NA	NA
	May 24 to May 30, 2021	2	NA	NA	NA	NA
Fukui						
	Apr 5 to Apr 11, 2021	1	NA	NA	NA	NA
	Apr 12 to Apr 18, 2021	0	NA	NA	NA	NA
	Apr 19 to Apr 25, 2021	1	NA	NA	NA	NA
	Apr 26 to May 2, 2021	0	NA	NA	NA	NA
	May 3 to May 9, 2021	3	NA	NA	NA	NA
	May 10 to May 16, 2021	2	0–1	0.00–100.00	0–0	0.00–0.00
	May 17 to May 23, 2021	4	0–3	0.00–300.00	0–0	0.00–0.00
	May 24 to May 30, 2021	2	0–1	0.00–100.00	0–0	0.00–0.00
Yamanashi						
	Apr 5 to Apr 11, 2021	1	0–0	0.00–0.00	0–1	0.00–50.00
	Apr 12 to Apr 18, 2021	1	0–0	0.00–0.00	0–2	0.00–66.67
	Apr 19 to Apr 25, 2021	0	0–0	0.00–0.00	0–2	0.00–100.00
	Apr 26 to May 2, 2021	0	NA	NA	NA	NA
	May 3 to May 9, 2021	2	NA	NA	NA	NA
	May 10 to May 16, 2021	2	NA	NA	NA	NA
	May 17 to May 23, 2021	5	0–2	0.00–66.67	0–0	0.00–0.00
	May 24 to May 30, 2021	4	0–2	0.00–100.00	0–0	0.00–0.00

Nagano						
	Apr 5 to Apr 11, 2021	3	0–0	0.00–0.00	0–0	0.00–0.00
	Apr 12 to Apr 18, 2021	5	0–2	0.00–66.67	0–0	0.00–0.00
	Apr 19 to Apr 25, 2021	4	0–1	0.00–33.33	0–0	0.00–0.00
	Apr 26 to May 2, 2021	2	0–0	0.00–0.00	0–2	0.00–50.00
	May 3 to May 9, 2021	7	0–4	0.00–133.33	0–0	0.00–0.00
	May 10 to May 16, 2021	5	0–2	0.00–66.67	0–0	0.00–0.00
	May 17 to May 23, 2021	3	0–0	0.00–0.00	0–0	0.00–0.00
	May 24 to May 30, 2021	6	0–3	0.00–100.00	0–0	0.00–0.00
Gifu						
	Apr 5 to Apr 11, 2021	4	0–0	0.00–0.00	0–0	0.00–0.00
	Apr 12 to Apr 18, 2021	7	0–3	0.00–75.00	0–0	0.00–0.00
	Apr 19 to Apr 25, 2021	4	0–0	0.00–0.00	0–0	0.00–0.00
	Apr 26 to May 2, 2021	1	0–0	0.00–0.00	0–3	0.00–75.00
	May 3 to May 9, 2021	8	1–5	33.33–166.67	0–0	0.00–0.00
	May 10 to May 16, 2021	5	0–2	0.00–66.67	0–0	0.00–0.00
	May 17 to May 23, 2021	7	0–4	0.00–133.33	0–0	0.00–0.00
	May 24 to May 30, 2021	4	0–1	0.00–33.33	0–0	0.00–0.00
Shizuoka						
	Apr 5 to Apr 11, 2021	8	0–1	0.00–14.29	0–0	0.00–0.00
	Apr 12 to Apr 18, 2021	9	0–2	0.00–28.57	0–0	0.00–0.00
	Apr 19 to Apr 25, 2021	5	0–0	0.00–0.00	0–2	0.00–28.57
	Apr 26 to May 2, 2021	7	0–0	0.00–0.00	0–0	0.00–0.00
	May 3 to May 9, 2021	4	0–0	0.00–0.00	0–3	0.00–42.86
	May 10 to May 16, 2021	8	0–2	0.00–33.33	0–0	0.00–0.00
	May 17 to May 23, 2021	10	0–3	0.00–42.86	0–0	0.00–0.00
	May 24 to May 30, 2021	2	0–0	0.00–0.00	0–5	0.00–71.43
Aichi						
	Apr 5 to Apr 11, 2021	10	0–2	0.00–25.00	0–0	0.00–0.00
	Apr 12 to Apr 18, 2021	10	0–2	0.00–25.00	0–0	0.00–0.00
	Apr 19 to Apr 25, 2021	8	0–0	0.00–0.00	0–0	0.00–0.00
	Apr 26 to May 2, 2021	11	0–3	0.00–37.50	0–0	0.00–0.00
	May 3 to May 9, 2021	7	0–0	0.00–0.00	0–1	0.00–12.50
	May 10 to May 16, 2021	16	2–8	25.00–100.00	0–0	0.00–0.00
	May 17 to May 23, 2021	10	0–2	0.00–25.00	0–0	0.00–0.00
	May 24 to May 30, 2021	8	0–0	0.00–0.00	0–0	0.00–0.00
Mie						
	Apr 5 to Apr 11, 2021	7	1–4	33.33–133.33	0–0	0.00–0.00
	Apr 12 to Apr 18, 2021	2	0–0	0.00–0.00	0–1	0.00–33.33
	Apr 19 to Apr 25, 2021	5	0–2	0.00–66.67	0–0	0.00–0.00
	Apr 26 to May 2, 2021	2	0–0	0.00–0.00	0–1	0.00–33.33
	May 3 to May 9, 2021	0	0–0	0.00–0.00	0–3	0.00–100.00
	May 10 to May 16, 2021	4	0–2	0.00–100.00	0–0	0.00–0.00
	May 17 to May 23, 2021	6	0–3	0.00–100.00	0–0	0.00–0.00
	May 24 to May 30, 2021	5	0–2	0.00–66.67	0–0	0.00–0.00
Shiga						

	Apr 5 to Apr 11, 2021	0	0–0	0.00–0.00	0–3	0.00–100.00
	Apr 12 to Apr 18, 2021	1	NA	NA	NA	NA
	Apr 19 to Apr 25, 2021	2	0–0	0.00–0.00	0–0	0.00–0.00
	Apr 26 to May 2, 2021	2	0–0	0.00–0.00	0–0	0.00–0.00
	May 3 to May 9, 2021	2	0–0	0.00–0.00	0–0	0.00–0.00
	May 10 to May 16, 2021	2	0–0	0.00–0.00	0–0	0.00–0.00
	May 17 to May 23, 2021	0	0–0	0.00–0.00	0–2	0.00–100.00
	May 24 to May 30, 2021	3	0–1	0.00–50.00	0–0	0.00–0.00
Kyoto						
	Apr 5 to Apr 11, 2021	5	0–1	0.00–25.00	0–0	0.00–0.00
	Apr 12 to Apr 18, 2021	3	0–0	0.00–0.00	0–0	0.00–0.00
	Apr 19 to Apr 25, 2021	8	1–5	33.33–166.67	0–0	0.00–0.00
	Apr 26 to May 2, 2021	4	0–1	0.00–33.33	0–0	0.00–0.00
	May 3 to May 9, 2021	6	0–3	0.00–100.00	0–0	0.00–0.00
	May 10 to May 16, 2021	5	0–2	0.00–66.67	0–0	0.00–0.00
	May 17 to May 23, 2021	3	0–0	0.00–0.00	0–0	0.00–0.00
	May 24 to May 30, 2021	4	0–1	0.00–33.33	0–0	0.00–0.00
Osaka						
	Apr 5 to Apr 11, 2021	21	0–4	0.00–23.53	0–0	0.00–0.00
	Apr 12 to Apr 18, 2021	14	0–0	0.00–0.00	0–3	0.00–17.65
	Apr 19 to Apr 25, 2021	20	0–3	0.00–17.65	0–0	0.00–0.00
	Apr 26 to May 2, 2021	27	2–10	11.76–58.82	0–0	0.00–0.00
	May 3 to May 9, 2021	23	0–6	0.00–35.29	0–0	0.00–0.00
	May 10 to May 16, 2021	18	0–1	0.00–5.88	0–0	0.00–0.00
	May 17 to May 23, 2021	25	0–9	0.00–56.25	0–0	0.00–0.00
	May 24 to May 30, 2021	17	0–1	0.00–6.25	0–0	0.00–0.00
Hyogo						
	Apr 5 to Apr 11, 2021	10	0–0	0.00–0.00	0–0	0.00–0.00
	Apr 12 to Apr 18, 2021	5	0–0	0.00–0.00	0–5	0.00–50.00
	Apr 19 to Apr 25, 2021	8	0–0	0.00–0.00	0–2	0.00–20.00
	Apr 26 to May 2, 2021	12	0–3	0.00–33.33	0–0	0.00–0.00
	May 3 to May 9, 2021	6	0–0	0.00–0.00	0–4	0.00–40.00
	May 10 to May 16, 2021	20	4–11	44.44–122.22	0–0	0.00–0.00
	May 17 to May 23, 2021	14	0–4	0.00–40.00	0–0	0.00–0.00
	May 24 to May 30, 2021	12	0–2	0.00–20.00	0–0	0.00–0.00
Nara						
	Apr 5 to Apr 11, 2021	1	NA	NA	NA	NA
	Apr 12 to Apr 18, 2021	4	0–1	0.00–33.33	0–0	0.00–0.00
	Apr 19 to Apr 25, 2021	3	0–0	0.00–0.00	0–0	0.00–0.00
	Apr 26 to May 2, 2021	3	0–0	0.00–0.00	0–0	0.00–0.00
	May 3 to May 9, 2021	7	1–4	33.33–133.33	0–0	0.00–0.00
	May 10 to May 16, 2021	4	0–1	0.00–33.33	0–0	0.00–0.00
	May 17 to May 23, 2021	3	0–1	0.00–50.00	0–0	0.00–0.00
	May 24 to May 30, 2021	3	0–1	0.00–50.00	0–0	0.00–0.00
Wakayama						
	Apr 5 to Apr 11, 2021	3	0–0	0.00–0.00	0–0	0.00–0.00

	Apr 12 to Apr 18, 2021	4	0–1	0.00–33.33	0–0	0.00–0.00
	Apr 19 to Apr 25, 2021	3	0–0	0.00–0.00	0–0	0.00–0.00
	Apr 26 to May 2, 2021	1	0–0	0.00–0.00	0–1	0.00–50.00
	May 3 to May 9, 2021	1	0–0	0.00–0.00	0–1	0.00–50.00
	May 10 to May 16, 2021	0	0–0	0.00–0.00	0–2	0.00–100.00
	May 17 to May 23, 2021	2	NA	NA	NA	NA
	May 24 to May 30, 2021	3	0–1	0.00–50.00	0–0	0.00–0.00
Tottori						
	Apr 5 to Apr 11, 2021	1	0–0	0.00–0.00	0–0	0.00–0.00
	Apr 12 to Apr 18, 2021	1	NA	NA	NA	NA
	Apr 19 to Apr 25, 2021	2	NA	NA	NA	NA
	Apr 26 to May 2, 2021	0	NA	NA	NA	NA
	May 3 to May 9, 2021	2	0–1	0.00–100.00	0–0	0.00–0.00
	May 10 to May 16, 2021	3	0–2	0.00–200.00	0–0	0.00–0.00
	May 17 to May 23, 2021	0	0–0	0.00–0.00	0–1	0.00–100.00
	May 24 to May 30, 2021	0	0–0	0.00–0.00	0–1	0.00–100.00
Shimane						
	Apr 5 to Apr 11, 2021	4	0–3	0.00–300.00	0–0	0.00–0.00
	Apr 12 to Apr 18, 2021	4	0–3	0.00–300.00	0–0	0.00–0.00
	Apr 19 to Apr 25, 2021	1	0–0	0.00–0.00	0–0	0.00–0.00
	Apr 26 to May 2, 2021	3	0–2	0.00–200.00	0–0	0.00–0.00
	May 3 to May 9, 2021	3	0–2	0.00–200.00	0–0	0.00–0.00
	May 10 to May 16, 2021	2	0–1	0.00–100.00	0–0	0.00–0.00
	May 17 to May 23, 2021	0	0–0	0.00–0.00	0–1	0.00–100.00
	May 24 to May 30, 2021	0	0–0	0.00–0.00	0–2	0.00–100.00
Okayama						
	Apr 5 to Apr 11, 2021	2	0–0	0.00–0.00	0–1	0.00–33.33
	Apr 12 to Apr 18, 2021	1	0–0	0.00–0.00	0–2	0.00–66.67
	Apr 19 to Apr 25, 2021	4	0–1	0.00–33.33	0–0	0.00–0.00
	Apr 26 to May 2, 2021	1	0–0	0.00–0.00	0–2	0.00–66.67
	May 3 to May 9, 2021	2	0–0	0.00–0.00	0–1	0.00–33.33
	May 10 to May 16, 2021	4	0–1	0.00–33.33	0–0	0.00–0.00
	May 17 to May 23, 2021	2	0–0	0.00–0.00	0–1	0.00–33.33
	May 24 to May 30, 2021	5	0–2	0.00–66.67	0–0	0.00–0.00
Hiroshima						
	Apr 5 to Apr 11, 2021	3	0–0	0.00–0.00	0–0	0.00–0.00
	Apr 12 to Apr 18, 2021	6	0–3	0.00–100.00	0–0	0.00–0.00
	Apr 19 to Apr 25, 2021	1	0–0	0.00–0.00	0–2	0.00–66.67
	Apr 26 to May 2, 2021	6	0–3	0.00–100.00	0–0	0.00–0.00
	May 3 to May 9, 2021	3	0–0	0.00–0.00	0–0	0.00–0.00
	May 10 to May 16, 2021	3	0–0	0.00–0.00	0–0	0.00–0.00
	May 17 to May 23, 2021	6	0–3	0.00–100.00	0–0	0.00–0.00
	May 24 to May 30, 2021	5	0–1	0.00–25.00	0–0	0.00–0.00
Yamaguchi						
	Apr 5 to Apr 11, 2021	1	0–0	0.00–0.00	0–0	0.00–0.00
	Apr 12 to Apr 18, 2021	1	0–0	0.00–0.00	0–0	0.00–0.00

	Apr 19 to Apr 25, 2021	4	0–3	0.00–300.00	0–0	0.00–0.00
	Apr 26 to May 2, 2021	2	0–1	0.00–100.00	0–0	0.00–0.00
	May 3 to May 9, 2021	4	0–3	0.00–300.00	0–0	0.00–0.00
	May 10 to May 16, 2021	3	0–1	0.00–50.00	0–0	0.00–0.00
	May 17 to May 23, 2021	6	1–4	50.00–200.00	0–0	0.00–0.00
	May 24 to May 30, 2021	0	0–0	0.00–0.00	0–2	0.00–100.00
Tokushima						
	Apr 5 to Apr 11, 2021	0	NA	NA	NA	NA
	Apr 12 to Apr 18, 2021	3	0–1	0.00–50.00	0–0	0.00–0.00
	Apr 19 to Apr 25, 2021	3	0–1	0.00–50.00	0–0	0.00–0.00
	Apr 26 to May 2, 2021	0	0–0	0.00–0.00	0–2	0.00–100.00
	May 3 to May 9, 2021	3	0–1	0.00–50.00	0–0	0.00–0.00
	May 10 to May 16, 2021	3	0–1	0.00–50.00	0–0	0.00–0.00
	May 17 to May 23, 2021	2	0–0	0.00–0.00	0–0	0.00–0.00
	May 24 to May 30, 2021	0	0–0	0.00–0.00	0–2	0.00–100.00
Kagawa						
	Apr 5 to Apr 11, 2021	2	0–0	0.00–0.00	0–0	0.00–0.00
	Apr 12 to Apr 18, 2021	3	0–1	0.00–50.00	0–0	0.00–0.00
	Apr 19 to Apr 25, 2021	2	0–0	0.00–0.00	0–0	0.00–0.00
	Apr 26 to May 2, 2021	4	0–2	0.00–100.00	0–0	0.00–0.00
	May 3 to May 9, 2021	3	0–1	0.00–50.00	0–0	0.00–0.00
	May 10 to May 16, 2021	0	0–0	0.00–0.00	0–2	0.00–100.00
	May 17 to May 23, 2021	4	0–2	0.00–100.00	0–0	0.00–0.00
	May 24 to May 30, 2021	7	2–5	100.00–250.00	0–0	0.00–0.00
Ehime						
	Apr 5 to Apr 11, 2021	1	NA	NA	NA	NA
	Apr 12 to Apr 18, 2021	6	0–3	0.00–100.00	0–0	0.00–0.00
	Apr 19 to Apr 25, 2021	6	0–3	0.00–100.00	0–0	0.00–0.00
	Apr 26 to May 2, 2021	3	0–0	0.00–0.00	0–0	0.00–0.00
	May 3 to May 9, 2021	3	0–0	0.00–0.00	0–0	0.00–0.00
	May 10 to May 16, 2021	4	0–1	0.00–33.33	0–0	0.00–0.00
	May 17 to May 23, 2021	4	0–1	0.00–33.33	0–0	0.00–0.00
	May 24 to May 30, 2021	3	0–1	0.00–50.00	0–0	0.00–0.00
Kochi						
	Apr 5 to Apr 11, 2021	4	0–2	0.00–100.00	0–0	0.00–0.00
	Apr 12 to Apr 18, 2021	0	0–0	0.00–0.00	0–2	0.00–100.00
	Apr 19 to Apr 25, 2021	0	0–0	0.00–0.00	0–2	0.00–100.00
	Apr 26 to May 2, 2021	2	0–1	0.00–100.00	0–0	0.00–0.00
	May 3 to May 9, 2021	1	NA	NA	NA	NA
	May 10 to May 16, 2021	2	0–1	0.00–100.00	0–0	0.00–0.00
	May 17 to May 23, 2021	0	0–0	0.00–0.00	0–1	0.00–100.00
	May 24 to May 30, 2021	0	NA	NA	NA	NA
Fukuoka						
	Apr 5 to Apr 11, 2021	6	0–1	0.00–20.00	0–0	0.00–0.00
	Apr 12 to Apr 18, 2021	7	0–2	0.00–40.00	0–0	0.00–0.00
	Apr 19 to Apr 25, 2021	4	0–0	0.00–0.00	0–1	0.00–20.00

	Apr 26 to May 2, 2021	5	0–0	0.00–0.00	0–0	0.00–0.00
	May 3 to May 9, 2021	6	0–1	0.00–20.00	0–0	0.00–0.00
	May 10 to May 16, 2021	4	0–0	0.00–0.00	0–1	0.00–20.00
	May 17 to May 23, 2021	6	0–1	0.00–20.00	0–0	0.00–0.00
	May 24 to May 30, 2021	10	1–6	25.00–150.00	0–0	0.00–0.00
Saga						
	Apr 5 to Apr 11, 2021	1	0–0	0.00–0.00	0–0	0.00–0.00
	Apr 12 to Apr 18, 2021	1	0–0	0.00–0.00	0–0	0.00–0.00
	Apr 19 to Apr 25, 2021	2	0–1	0.00–100.00	0–0	0.00–0.00
	Apr 26 to May 2, 2021	2	0–1	0.00–100.00	0–0	0.00–0.00
	May 3 to May 9, 2021	0	0–0	0.00–0.00	0–1	0.00–100.00
	May 10 to May 16, 2021	2	0–1	0.00–100.00	0–0	0.00–0.00
	May 17 to May 23, 2021	1	0–0	0.00–0.00	0–0	0.00–0.00
	May 24 to May 30, 2021	0	NA	NA	NA	NA
Nagasaki						
	Apr 5 to Apr 11, 2021	1	0–0	0.00–0.00	0–1	0.00–50.00
	Apr 12 to Apr 18, 2021	4	0–2	0.00–100.00	0–0	0.00–0.00
	Apr 19 to Apr 25, 2021	4	0–2	0.00–100.00	0–0	0.00–0.00
	Apr 26 to May 2, 2021	3	0–1	0.00–50.00	0–0	0.00–0.00
	May 3 to May 9, 2021	2	0–0	0.00–0.00	0–0	0.00–0.00
	May 10 to May 16, 2021	4	0–2	0.00–100.00	0–0	0.00–0.00
	May 17 to May 23, 2021	2	0–0	0.00–0.00	0–0	0.00–0.00
	May 24 to May 30, 2021	3	0–1	0.00–50.00	0–0	0.00–0.00
Kumamoto						
	Apr 5 to Apr 11, 2021	1	0–0	0.00–0.00	0–1	0.00–50.00
	Apr 12 to Apr 18, 2021	3	0–0	0.00–0.00	0–0	0.00–0.00
	Apr 19 to Apr 25, 2021	1	0–0	0.00–0.00	0–1	0.00–50.00
	Apr 26 to May 2, 2021	4	0–2	0.00–100.00	0–0	0.00–0.00
	May 3 to May 9, 2021	4	0–1	0.00–33.33	0–0	0.00–0.00
	May 10 to May 16, 2021	0	0–0	0.00–0.00	0–2	0.00–100.00
	May 17 to May 23, 2021	3	0–1	0.00–50.00	0–0	0.00–0.00
	May 24 to May 30, 2021	6	0–4	0.00–200.00	0–0	0.00–0.00
Oita						
	Apr 5 to Apr 11, 2021	0	NA	NA	NA	NA
	Apr 12 to Apr 18, 2021	1	NA	NA	NA	NA
	Apr 19 to Apr 25, 2021	0	NA	NA	NA	NA
	Apr 26 to May 2, 2021	0	NA	NA	NA	NA
	May 3 to May 9, 2021	3	NA	NA	NA	NA
	May 10 to May 16, 2021	0	NA	NA	NA	NA
	May 17 to May 23, 2021	2	0–1	0.00–100.00	0–0	0.00–0.00
	May 24 to May 30, 2021	0	0–0	0.00–0.00	0–1	0.00–100.00
Miyazaki						
	Apr 5 to Apr 11, 2021	0	NA	NA	NA	NA
	Apr 12 to Apr 18, 2021	2	NA	NA	NA	NA
	Apr 19 to Apr 25, 2021	1	0–0	0.00–0.00	0–0	0.00–0.00
	Apr 26 to May 2, 2021	2	0–1	0.00–100.00	0–0	0.00–0.00

	May 3 to May 9, 2021	2	0–1	0.00–100.00	0–0	0.00–0.00
	May 10 to May 16, 2021	3	0–2	0.00–200.00	0–0	0.00–0.00
	May 17 to May 23, 2021	3	0–2	0.00–200.00	0–0	0.00–0.00
	May 24 to May 30, 2021	2	0–1	0.00–100.00	0–0	0.00–0.00
Kagoshima						
	Apr 5 to Apr 11, 2021	2	0–0	0.00–0.00	0–1	0.00–33.33
	Apr 12 to Apr 18, 2021	1	0–0	0.00–0.00	0–2	0.00–66.67
	Apr 19 to Apr 25, 2021	5	0–3	0.00–150.00	0–0	0.00–0.00
	Apr 26 to May 2, 2021	2	0–0	0.00–0.00	0–0	0.00–0.00
	May 3 to May 9, 2021	2	0–0	0.00–0.00	0–0	0.00–0.00
	May 10 to May 16, 2021	5	0–3	0.00–150.00	0–0	0.00–0.00
	May 17 to May 23, 2021	3	0–1	0.00–50.00	0–0	0.00–0.00
	May 24 to May 30, 2021	0	0–0	0.00–0.00	0–2	0.00–100.00
Okinawa						
	Apr 5 to Apr 11, 2021	1	0–0	0.00–0.00	0–1	0.00–50.00
	Apr 12 to Apr 18, 2021	3	0–1	0.00–50.00	0–0	0.00–0.00
	Apr 19 to Apr 25, 2021	0	NA	NA	NA	NA
	Apr 26 to May 2, 2021	3	0–1	0.00–50.00	0–0	0.00–0.00
	May 3 to May 9, 2021	3	0–1	0.00–50.00	0–0	0.00–0.00
	May 10 to May 16, 2021	3	0–1	0.00–50.00	0–0	0.00–0.00
	May 17 to May 23, 2021	4	0–2	0.00–100.00	0–0	0.00–0.00
	May 24 to May 30, 2021	4	0–2	0.00–100.00	0–0	0.00–0.00

Table A.9: Weekly number of observed and excess/exiguous deaths in Japan and 47 prefectures for circulatory disease-related deaths in all places from January 2020 through May 2021.

Prefecture	Week	Observed	Excess deaths	Percent excess	Exiguous deaths	Percent exiguous
Japan						
	Apr 5 to Apr 11, 2021	6986	0–271	0.00–4.04	0–0	0.00–0.00
	Apr 12 to Apr 18, 2021	7108	0–534	0.00–8.12	0–0	0.00–0.00
	Apr 19 to Apr 25, 2021	7062	27–638	0.42–9.93	0–0	0.00–0.00
	Apr 26 to May 2, 2021	7135	253–839	4.02–13.33	0–0	0.00–0.00
	May 3 to May 9, 2021	7042	308–886	5.00–14.39	0–0	0.00–0.00
	May 10 to May 16, 2021	6726	121–704	2.01–11.69	0–0	0.00–0.00
	May 17 to May 23, 2021	6356	0–499	0.00–8.52	0–0	0.00–0.00
	May 24 to May 30, 2021	6279	0–504	0.00–8.73	0–0	0.00–0.00
Hokkaido						
	Apr 5 to Apr 11, 2021	320	0–0	0.00–0.00	0–0	0.00–0.00
	Apr 12 to Apr 18, 2021	301	0–0	0.00–0.00	0–14	0.00–4.44
	Apr 19 to Apr 25, 2021	350	0–36	0.00–11.46	0–0	0.00–0.00
	Apr 26 to May 2, 2021	328	0–20	0.00–6.49	0–0	0.00–0.00
	May 3 to May 9, 2021	357	12–55	3.97–18.21	0–0	0.00–0.00
	May 10 to May 16, 2021	345	3–45	1.00–15.00	0–0	0.00–0.00
	May 17 to May 23, 2021	352	9–52	3.00–17.33	0–0	0.00–0.00
	May 24 to May 30, 2021	323	0–30	0.00–10.24	0–0	0.00–0.00
Aomori						
	Apr 5 to Apr 11, 2021	116	2–21	2.11–22.11	0–0	0.00–0.00
	Apr 12 to Apr 18, 2021	88	0–0	0.00–0.00	0–6	0.00–6.38
	Apr 19 to Apr 25, 2021	91	0–0	0.00–0.00	0–2	0.00–2.15
	Apr 26 to May 2, 2021	90	0–0	0.00–0.00	0–1	0.00–1.10
	May 3 to May 9, 2021	95	0–7	0.00–7.95	0–0	0.00–0.00
	May 10 to May 16, 2021	82	0–0	0.00–0.00	0–3	0.00–3.53
	May 17 to May 23, 2021	90	0–7	0.00–8.43	0–0	0.00–0.00
	May 24 to May 30, 2021	93	0–11	0.00–13.41	0–0	0.00–0.00
Iwate						
	Apr 5 to Apr 11, 2021	96	0–0	0.00–0.00	0–4	0.00–4.00
	Apr 12 to Apr 18, 2021	92	0–0	0.00–0.00	0–5	0.00–5.15
	Apr 19 to Apr 25, 2021	89	0–0	0.00–0.00	0–7	0.00–7.29
	Apr 26 to May 2, 2021	118	4–25	4.30–26.88	0–0	0.00–0.00
	May 3 to May 9, 2021	116	6–27	6.74–30.34	0–0	0.00–0.00
	May 10 to May 16, 2021	90	0–4	0.00–4.65	0–0	0.00–0.00
	May 17 to May 23, 2021	104	0–18	0.00–20.93	0–0	0.00–0.00
	May 24 to May 30, 2021	98	0–13	0.00–15.29	0–0	0.00–0.00
Miyagi						
	Apr 5 to Apr 11, 2021	158	0–24	0.00–17.91	0–0	0.00–0.00
	Apr 12 to Apr 18, 2021	127	0–0	0.00–0.00	0–5	0.00–3.79
	Apr 19 to Apr 25, 2021	132	0–4	0.00–3.13	0–0	0.00–0.00
	Apr 26 to May 2, 2021	157	7–32	5.60–25.60	0–0	0.00–0.00
	May 3 to May 9, 2021	129	0–6	0.00–4.88	0–0	0.00–0.00

	May 10 to May 16, 2021	115	0–0	0.00–0.00	0–7	0.00–5.74
	May 17 to May 23, 2021	120	0–1	0.00–0.84	0–0	0.00–0.00
	May 24 to May 30, 2021	125	0–10	0.00–8.70	0–0	0.00–0.00
Akita						
	Apr 5 to Apr 11, 2021	92	0–14	0.00–17.95	0–0	0.00–0.00
	Apr 12 to Apr 18, 2021	89	0–13	0.00–17.11	0–0	0.00–0.00
	Apr 19 to Apr 25, 2021	71	0–0	0.00–0.00	0–4	0.00–5.33
	Apr 26 to May 2, 2021	79	0–4	0.00–5.33	0–0	0.00–0.00
	May 3 to May 9, 2021	109	17–35	22.97–47.30	0–0	0.00–0.00
	May 10 to May 16, 2021	78	0–5	0.00–6.85	0–0	0.00–0.00
	May 17 to May 23, 2021	77	0–5	0.00–6.94	0–0	0.00–0.00
	May 24 to May 30, 2021	66	0–0	0.00–0.00	0–5	0.00–7.04
Yamagata						
	Apr 5 to Apr 11, 2021	78	0–0	0.00–0.00	0–5	0.00–6.02
	Apr 12 to Apr 18, 2021	93	0–9	0.00–10.71	0–0	0.00–0.00
	Apr 19 to Apr 25, 2021	89	0–6	0.00–7.23	0–0	0.00–0.00
	Apr 26 to May 2, 2021	70	0–0	0.00–0.00	0–11	0.00–13.58
	May 3 to May 9, 2021	81	0–2	0.00–2.53	0–0	0.00–0.00
	May 10 to May 16, 2021	84	0–7	0.00–9.09	0–0	0.00–0.00
	May 17 to May 23, 2021	80	0–7	0.00–9.59	0–0	0.00–0.00
	May 24 to May 30, 2021	82	0–9	0.00–12.33	0–0	0.00–0.00
Fukushima						
	Apr 5 to Apr 11, 2021	132	0–5	0.00–3.94	0–0	0.00–0.00
	Apr 12 to Apr 18, 2021	137	0–11	0.00–8.73	0–0	0.00–0.00
	Apr 19 to Apr 25, 2021	143	0–19	0.00–15.32	0–0	0.00–0.00
	Apr 26 to May 2, 2021	134	0–12	0.00–9.84	0–0	0.00–0.00
	May 3 to May 9, 2021	141	0–22	0.00–18.49	0–0	0.00–0.00
	May 10 to May 16, 2021	126	0–8	0.00–6.78	0–0	0.00–0.00
	May 17 to May 23, 2021	136	0–20	0.00–17.24	0–0	0.00–0.00
	May 24 to May 30, 2021	118	0–5	0.00–4.42	0–0	0.00–0.00
Ibaraki						
	Apr 5 to Apr 11, 2021	180	0–13	0.00–7.78	0–0	0.00–0.00
	Apr 12 to Apr 18, 2021	190	0–28	0.00–17.28	0–0	0.00–0.00
	Apr 19 to Apr 25, 2021	162	0–5	0.00–3.18	0–0	0.00–0.00
	Apr 26 to May 2, 2021	158	0–6	0.00–3.95	0–0	0.00–0.00
	May 3 to May 9, 2021	169	0–21	0.00–14.19	0–0	0.00–0.00
	May 10 to May 16, 2021	191	14–44	9.52–29.93	0–0	0.00–0.00
	May 17 to May 23, 2021	140	0–0	0.00–0.00	0–3	0.00–2.10
	May 24 to May 30, 2021	147	0–8	0.00–5.76	0–0	0.00–0.00
Tochigi						
	Apr 5 to Apr 11, 2021	123	0–7	0.00–6.03	0–0	0.00–0.00
	Apr 12 to Apr 18, 2021	148	14–35	12.39–30.97	0–0	0.00–0.00
	Apr 19 to Apr 25, 2021	123	0–13	0.00–11.82	0–0	0.00–0.00
	Apr 26 to May 2, 2021	127	0–19	0.00–17.59	0–0	0.00–0.00
	May 3 to May 9, 2021	116	0–12	0.00–11.54	0–0	0.00–0.00
	May 10 to May 16, 2021	107	0–7	0.00–7.00	0–0	0.00–0.00

	May 17 to May 23, 2021	136	18–38	18.37–38.78	0–0	0.00–0.00
	May 24 to May 30, 2021	100	0–4	0.00–4.17	0–0	0.00–0.00
Gunma						
	Apr 5 to Apr 11, 2021	123	0–1	0.00–0.82	0–0	0.00–0.00
	Apr 12 to Apr 18, 2021	145	4–28	3.42–23.93	0–0	0.00–0.00
	Apr 19 to Apr 25, 2021	123	0–7	0.00–6.03	0–0	0.00–0.00
	Apr 26 to May 2, 2021	139	1–26	0.88–23.01	0–0	0.00–0.00
	May 3 to May 9, 2021	134	0–23	0.00–20.72	0–0	0.00–0.00
	May 10 to May 16, 2021	136	3–27	2.75–24.77	0–0	0.00–0.00
	May 17 to May 23, 2021	118	0–10	0.00–9.26	0–0	0.00–0.00
	May 24 to May 30, 2021	124	0–17	0.00–15.89	0–0	0.00–0.00
Saitama						
	Apr 5 to Apr 11, 2021	327	0–0	0.00–0.00	0–7	0.00–2.10
	Apr 12 to Apr 18, 2021	348	0–21	0.00–6.42	0–0	0.00–0.00
	Apr 19 to Apr 25, 2021	362	0–44	0.00–13.84	0–0	0.00–0.00
	Apr 26 to May 2, 2021	391	32–78	10.22–24.92	0–0	0.00–0.00
	May 3 to May 9, 2021	382	33–77	10.82–25.25	0–0	0.00–0.00
	May 10 to May 16, 2021	330	0–30	0.00–10.00	0–0	0.00–0.00
	May 17 to May 23, 2021	303	0–11	0.00–3.77	0–0	0.00–0.00
	May 24 to May 30, 2021	310	0–23	0.00–8.01	0–0	0.00–0.00
Chiba						
	Apr 5 to Apr 11, 2021	341	0–30	0.00–9.65	0–0	0.00–0.00
	Apr 12 to Apr 18, 2021	346	0–42	0.00–13.82	0–0	0.00–0.00
	Apr 19 to Apr 25, 2021	348	8–55	2.73–18.77	0–0	0.00–0.00
	Apr 26 to May 2, 2021	354	18–65	6.23–22.49	0–0	0.00–0.00
	May 3 to May 9, 2021	312	0–32	0.00–11.43	0–0	0.00–0.00
	May 10 to May 16, 2021	323	0–45	0.00–16.19	0–0	0.00–0.00
	May 17 to May 23, 2021	297	0–25	0.00–9.19	0–0	0.00–0.00
	May 24 to May 30, 2021	274	0–8	0.00–3.01	0–0	0.00–0.00
Tokyo						
	Apr 5 to Apr 11, 2021	613	0–24	0.00–4.07	0–0	0.00–0.00
	Apr 12 to Apr 18, 2021	631	0–55	0.00–9.55	0–0	0.00–0.00
	Apr 19 to Apr 25, 2021	623	0–62	0.00–11.05	0–0	0.00–0.00
	Apr 26 to May 2, 2021	619	7–79	1.30–14.63	0–0	0.00–0.00
	May 3 to May 9, 2021	589	0–61	0.00–11.55	0–0	0.00–0.00
	May 10 to May 16, 2021	532	0–12	0.00–2.31	0–0	0.00–0.00
	May 17 to May 23, 2021	500	0–0	0.00–0.00	0–9	0.00–1.77
	May 24 to May 30, 2021	479	0–0	0.00–0.00	0–19	0.00–3.82
Kanagawa						
	Apr 5 to Apr 11, 2021	418	0–27	0.00–6.91	0–0	0.00–0.00
	Apr 12 to Apr 18, 2021	420	0–36	0.00–9.38	0–0	0.00–0.00
	Apr 19 to Apr 25, 2021	395	0–17	0.00–4.50	0–0	0.00–0.00
	Apr 26 to May 2, 2021	440	26–76	7.14–20.88	0–0	0.00–0.00
	May 3 to May 9, 2021	404	1–47	0.28–13.17	0–0	0.00–0.00
	May 10 to May 16, 2021	364	0–10	0.00–2.82	0–0	0.00–0.00
	May 17 to May 23, 2021	404	9–56	2.59–16.09	0–0	0.00–0.00

	May 24 to May 30, 2021	354	0–13	0.00–3.81	0–0	0.00–0.00
Niigata						
	Apr 5 to Apr 11, 2021	154	0–4	0.00–2.67	0–0	0.00–0.00
	Apr 12 to Apr 18, 2021	156	0–7	0.00–4.70	0–0	0.00–0.00
	Apr 19 to Apr 25, 2021	151	0–4	0.00–2.72	0–0	0.00–0.00
	Apr 26 to May 2, 2021	153	0–9	0.00–6.25	0–0	0.00–0.00
	May 3 to May 9, 2021	142	0–1	0.00–0.71	0–0	0.00–0.00
	May 10 to May 16, 2021	136	0–0	0.00–0.00	0–2	0.00–1.45
	May 17 to May 23, 2021	133	0–0	0.00–0.00	0–2	0.00–1.48
	May 24 to May 30, 2021	127	0–0	0.00–0.00	0–6	0.00–4.51
Toyama						
	Apr 5 to Apr 11, 2021	84	8–24	13.33–40.00	0–0	0.00–0.00
	Apr 12 to Apr 18, 2021	73	0–14	0.00–23.73	0–0	0.00–0.00
	Apr 19 to Apr 25, 2021	56	0–0	0.00–0.00	0–1	0.00–1.75
	Apr 26 to May 2, 2021	67	0–12	0.00–21.82	0–0	0.00–0.00
	May 3 to May 9, 2021	70	0–16	0.00–29.63	0–0	0.00–0.00
	May 10 to May 16, 2021	54	0–0	0.00–0.00	0–0	0.00–0.00
	May 17 to May 23, 2021	50	0–0	0.00–0.00	0–3	0.00–5.66
	May 24 to May 30, 2021	70	4–19	7.84–37.25	0–0	0.00–0.00
Ishikawa						
	Apr 5 to Apr 11, 2021	79	0–13	0.00–19.70	0–0	0.00–0.00
	Apr 12 to Apr 18, 2021	78	0–13	0.00–20.00	0–0	0.00–0.00
	Apr 19 to Apr 25, 2021	69	0–7	0.00–11.29	0–0	0.00–0.00
	Apr 26 to May 2, 2021	80	5–21	8.47–35.59	0–0	0.00–0.00
	May 3 to May 9, 2021	81	8–24	14.04–42.11	0–0	0.00–0.00
	May 10 to May 16, 2021	75	2–18	3.51–31.58	0–0	0.00–0.00
	May 17 to May 23, 2021	67	0–11	0.00–19.64	0–0	0.00–0.00
	May 24 to May 30, 2021	75	3–20	5.45–36.36	0–0	0.00–0.00
Fukui						
	Apr 5 to Apr 11, 2021	49	0–0	0.00–0.00	0–3	0.00–5.77
	Apr 12 to Apr 18, 2021	52	0–2	0.00–4.00	0–0	0.00–0.00
	Apr 19 to Apr 25, 2021	47	0–0	0.00–0.00	0–2	0.00–4.08
	Apr 26 to May 2, 2021	66	3–19	6.38–40.43	0–0	0.00–0.00
	May 3 to May 9, 2021	70	10–24	21.74–52.17	0–0	0.00–0.00
	May 10 to May 16, 2021	52	0–7	0.00–15.56	0–0	0.00–0.00
	May 17 to May 23, 2021	52	0–7	0.00–15.56	0–0	0.00–0.00
	May 24 to May 30, 2021	53	0–9	0.00–20.45	0–0	0.00–0.00
Yamanashi						
	Apr 5 to Apr 11, 2021	42	0–0	0.00–0.00	0–8	0.00–16.00
	Apr 12 to Apr 18, 2021	70	5–21	10.20–42.86	0–0	0.00–0.00
	Apr 19 to Apr 25, 2021	50	0–3	0.00–6.38	0–0	0.00–0.00
	Apr 26 to May 2, 2021	52	0–6	0.00–13.04	0–0	0.00–0.00
	May 3 to May 9, 2021	50	0–6	0.00–13.64	0–0	0.00–0.00
	May 10 to May 16, 2021	62	3–18	6.82–40.91	0–0	0.00–0.00
	May 17 to May 23, 2021	45	0–1	0.00–2.27	0–0	0.00–0.00
	May 24 to May 30, 2021	50	0–7	0.00–16.28	0–0	0.00–0.00

Nagano						
	Apr 5 to Apr 11, 2021	133	0–0	0.00–0.00	0–2	0.00–1.48
	Apr 12 to Apr 18, 2021	126	0–0	0.00–0.00	0–9	0.00–6.67
	Apr 19 to Apr 25, 2021	136	0–2	0.00–1.49	0–0	0.00–0.00
	Apr 26 to May 2, 2021	128	0–0	0.00–0.00	0–3	0.00–2.29
	May 3 to May 9, 2021	130	0–1	0.00–0.78	0–0	0.00–0.00
	May 10 to May 16, 2021	139	0–12	0.00–9.45	0–0	0.00–0.00
	May 17 to May 23, 2021	113	0–0	0.00–0.00	0–12	0.00–9.60
	May 24 to May 30, 2021	106	0–0	0.00–0.00	0–13	0.00–10.92
Gifu						
	Apr 5 to Apr 11, 2021	112	0–3	0.00–2.75	0–0	0.00–0.00
	Apr 12 to Apr 18, 2021	113	0–6	0.00–5.61	0–0	0.00–0.00
	Apr 19 to Apr 25, 2021	130	4–26	3.85–25.00	0–0	0.00–0.00
	Apr 26 to May 2, 2021	104	0–4	0.00–4.00	0–0	0.00–0.00
	May 3 to May 9, 2021	98	0–4	0.00–4.26	0–0	0.00–0.00
	May 10 to May 16, 2021	108	0–14	0.00–14.89	0–0	0.00–0.00
	May 17 to May 23, 2021	109	0–18	0.00–19.78	0–0	0.00–0.00
	May 24 to May 30, 2021	107	0–17	0.00–18.89	0–0	0.00–0.00
Shizuoka						
	Apr 5 to Apr 11, 2021	230	0–22	0.00–10.58	0–0	0.00–0.00
	Apr 12 to Apr 18, 2021	230	0–26	0.00–12.75	0–0	0.00–0.00
	Apr 19 to Apr 25, 2021	219	0–20	0.00–10.05	0–0	0.00–0.00
	Apr 26 to May 2, 2021	217	0–23	0.00–11.86	0–0	0.00–0.00
	May 3 to May 9, 2021	198	0–9	0.00–4.76	0–0	0.00–0.00
	May 10 to May 16, 2021	206	0–20	0.00–10.75	0–0	0.00–0.00
	May 17 to May 23, 2021	190	0–10	0.00–5.56	0–0	0.00–0.00
	May 24 to May 30, 2021	182	0–5	0.00–2.82	0–0	0.00–0.00
Aichi						
	Apr 5 to Apr 11, 2021	309	0–2	0.00–0.65	0–0	0.00–0.00
	Apr 12 to Apr 18, 2021	290	0–0	0.00–0.00	0–12	0.00–3.97
	Apr 19 to Apr 25, 2021	323	0–29	0.00–9.86	0–0	0.00–0.00
	Apr 26 to May 2, 2021	303	0–14	0.00–4.84	0–0	0.00–0.00
	May 3 to May 9, 2021	285	0–1	0.00–0.35	0–0	0.00–0.00
	May 10 to May 16, 2021	320	6–48	2.21–17.65	0–0	0.00–0.00
	May 17 to May 23, 2021	248	0–0	0.00–0.00	0–19	0.00–7.12
	May 24 to May 30, 2021	276	0–13	0.00–4.94	0–0	0.00–0.00
Mie						
	Apr 5 to Apr 11, 2021	106	0–0	0.00–0.00	0–0	0.00–0.00
	Apr 12 to Apr 18, 2021	110	0–8	0.00–7.84	0–0	0.00–0.00
	Apr 19 to Apr 25, 2021	95	0–0	0.00–0.00	0–4	0.00–4.04
	Apr 26 to May 2, 2021	128	11–34	11.70–36.17	0–0	0.00–0.00
	May 3 to May 9, 2021	84	0–0	0.00–0.00	0–7	0.00–7.69
	May 10 to May 16, 2021	94	0–5	0.00–5.62	0–0	0.00–0.00
	May 17 to May 23, 2021	99	0–12	0.00–13.79	0–0	0.00–0.00
	May 24 to May 30, 2021	79	0–0	0.00–0.00	0–7	0.00–8.14
Shiga						

	Apr 5 to Apr 11, 2021	68	0–3	0.00–4.62	0–0	0.00–0.00
	Apr 12 to Apr 18, 2021	81	0–16	0.00–24.62	0–0	0.00–0.00
	Apr 19 to Apr 25, 2021	59	0–0	0.00–0.00	0–3	0.00–4.84
	Apr 26 to May 2, 2021	62	0–1	0.00–1.64	0–0	0.00–0.00
	May 3 to May 9, 2021	72	0–14	0.00–24.14	0–0	0.00–0.00
	May 10 to May 16, 2021	58	0–2	0.00–3.57	0–0	0.00–0.00
	May 17 to May 23, 2021	65	0–11	0.00–20.37	0–0	0.00–0.00
	May 24 to May 30, 2021	66	0–15	0.00–29.41	0–0	0.00–0.00
Kyoto						
	Apr 5 to Apr 11, 2021	158	0–21	0.00–15.33	0–0	0.00–0.00
	Apr 12 to Apr 18, 2021	146	0–12	0.00–8.96	0–0	0.00–0.00
	Apr 19 to Apr 25, 2021	154	0–22	0.00–16.67	0–0	0.00–0.00
	Apr 26 to May 2, 2021	159	4–31	3.13–24.22	0–0	0.00–0.00
	May 3 to May 9, 2021	147	0–21	0.00–16.67	0–0	0.00–0.00
	May 10 to May 16, 2021	147	2–27	1.67–22.50	0–0	0.00–0.00
	May 17 to May 23, 2021	146	3–28	2.54–23.73	0–0	0.00–0.00
	May 24 to May 30, 2021	133	0–17	0.00–14.66	0–0	0.00–0.00
Osaka						
	Apr 5 to Apr 11, 2021	457	0–14	0.00–3.16	0–0	0.00–0.00
	Apr 12 to Apr 18, 2021	458	0–25	0.00–5.77	0–0	0.00–0.00
	Apr 19 to Apr 25, 2021	469	0–44	0.00–10.35	0–0	0.00–0.00
	Apr 26 to May 2, 2021	469	0–55	0.00–13.29	0–0	0.00–0.00
	May 3 to May 9, 2021	485	22–80	5.43–19.75	0–0	0.00–0.00
	May 10 to May 16, 2021	433	0–38	0.00–9.62	0–0	0.00–0.00
	May 17 to May 23, 2021	420	0–35	0.00–9.09	0–0	0.00–0.00
	May 24 to May 30, 2021	412	0–34	0.00–8.99	0–0	0.00–0.00
Hyogo						
	Apr 5 to Apr 11, 2021	309	0–28	0.00–9.96	0–0	0.00–0.00
	Apr 12 to Apr 18, 2021	288	0–12	0.00–4.35	0–0	0.00–0.00
	Apr 19 to Apr 25, 2021	299	0–29	0.00–10.74	0–0	0.00–0.00
	Apr 26 to May 2, 2021	301	0–41	0.00–15.77	0–0	0.00–0.00
	May 3 to May 9, 2021	294	0–40	0.00–15.75	0–0	0.00–0.00
	May 10 to May 16, 2021	300	7–48	2.78–19.05	0–0	0.00–0.00
	May 17 to May 23, 2021	269	0–24	0.00–9.80	0–0	0.00–0.00
	May 24 to May 30, 2021	293	6–49	2.46–20.08	0–0	0.00–0.00
Nara						
	Apr 5 to Apr 11, 2021	66	0–0	0.00–0.00	0–7	0.00–9.59
	Apr 12 to Apr 18, 2021	92	2–21	2.82–29.58	0–0	0.00–0.00
	Apr 19 to Apr 25, 2021	72	0–1	0.00–1.41	0–0	0.00–0.00
	Apr 26 to May 2, 2021	80	0–12	0.00–17.65	0–0	0.00–0.00
	May 3 to May 9, 2021	68	0–0	0.00–0.00	0–0	0.00–0.00
	May 10 to May 16, 2021	68	0–2	0.00–3.03	0–0	0.00–0.00
	May 17 to May 23, 2021	68	0–3	0.00–4.62	0–0	0.00–0.00
	May 24 to May 30, 2021	64	0–1	0.00–1.59	0–0	0.00–0.00
Wakayama						
	Apr 5 to Apr 11, 2021	67	0–5	0.00–8.06	0–0	0.00–0.00

	Apr 12 to Apr 18, 2021	48	0–0	0.00–0.00	0–13	0.00–21.31
	Apr 19 to Apr 25, 2021	63	0–4	0.00–6.78	0–0	0.00–0.00
	Apr 26 to May 2, 2021	62	0–6	0.00–10.71	0–0	0.00–0.00
	May 3 to May 9, 2021	65	0–11	0.00–20.37	0–0	0.00–0.00
	May 10 to May 16, 2021	68	1–16	1.92–30.77	0–0	0.00–0.00
	May 17 to May 23, 2021	50	0–0	0.00–0.00	0–0	0.00–0.00
	May 24 to May 30, 2021	49	0–0	0.00–0.00	0–0	0.00–0.00
Tottori						
	Apr 5 to Apr 11, 2021	36	0–1	0.00–2.86	0–0	0.00–0.00
	Apr 12 to Apr 18, 2021	45	0–11	0.00–32.35	0–0	0.00–0.00
	Apr 19 to Apr 25, 2021	39	0–5	0.00–14.71	0–0	0.00–0.00
	Apr 26 to May 2, 2021	31	0–0	0.00–0.00	0–2	0.00–6.06
	May 3 to May 9, 2021	45	0–12	0.00–36.36	0–0	0.00–0.00
	May 10 to May 16, 2021	44	1–13	3.23–41.94	0–0	0.00–0.00
	May 17 to May 23, 2021	28	0–0	0.00–0.00	0–2	0.00–6.67
	May 24 to May 30, 2021	34	0–6	0.00–21.43	0–0	0.00–0.00
Shimane						
	Apr 5 to Apr 11, 2021	37	0–0	0.00–0.00	0–11	0.00–22.92
	Apr 12 to Apr 18, 2021	56	0–8	0.00–16.67	0–0	0.00–0.00
	Apr 19 to Apr 25, 2021	38	0–0	0.00–0.00	0–10	0.00–20.83
	Apr 26 to May 2, 2021	46	0–0	0.00–0.00	0–1	0.00–2.13
	May 3 to May 9, 2021	51	0–6	0.00–13.33	0–0	0.00–0.00
	May 10 to May 16, 2021	49	0–7	0.00–16.67	0–0	0.00–0.00
	May 17 to May 23, 2021	58	4–18	10.00–45.00	0–0	0.00–0.00
	May 24 to May 30, 2021	36	0–0	0.00–0.00	0–3	0.00–7.69
Okayama						
	Apr 5 to Apr 11, 2021	104	0–0	0.00–0.00	0–1	0.00–0.95
	Apr 12 to Apr 18, 2021	91	0–0	0.00–0.00	0–11	0.00–10.78
	Apr 19 to Apr 25, 2021	112	0–13	0.00–13.13	0–0	0.00–0.00
	Apr 26 to May 2, 2021	112	0–14	0.00–14.29	0–0	0.00–0.00
	May 3 to May 9, 2021	104	0–7	0.00–7.22	0–0	0.00–0.00
	May 10 to May 16, 2021	101	0–5	0.00–5.21	0–0	0.00–0.00
	May 17 to May 23, 2021	110	0–17	0.00–18.28	0–0	0.00–0.00
	May 24 to May 30, 2021	107	0–18	0.00–20.22	0–0	0.00–0.00
Hiroshima						
	Apr 5 to Apr 11, 2021	181	7–32	4.70–21.48	0–0	0.00–0.00
	Apr 12 to Apr 18, 2021	181	8–33	5.41–22.30	0–0	0.00–0.00
	Apr 19 to Apr 25, 2021	163	0–19	0.00–13.19	0–0	0.00–0.00
	Apr 26 to May 2, 2021	156	0–13	0.00–9.09	0–0	0.00–0.00
	May 3 to May 9, 2021	166	0–25	0.00–17.73	0–0	0.00–0.00
	May 10 to May 16, 2021	163	0–24	0.00–17.27	0–0	0.00–0.00
	May 17 to May 23, 2021	147	0–14	0.00–10.53	0–0	0.00–0.00
	May 24 to May 30, 2021	142	0–11	0.00–8.40	0–0	0.00–0.00
Yamaguchi						
	Apr 5 to Apr 11, 2021	88	0–0	0.00–0.00	0–12	0.00–12.00
	Apr 12 to Apr 18, 2021	119	0–20	0.00–20.20	0–0	0.00–0.00

	Apr 19 to Apr 25, 2021	106	0–10	0.00–10.42	0–0	0.00–0.00
	Apr 26 to May 2, 2021	92	0–0	0.00–0.00	0–2	0.00–2.13
	May 3 to May 9, 2021	102	0–10	0.00–10.87	0–0	0.00–0.00
	May 10 to May 16, 2021	76	0–0	0.00–0.00	0–13	0.00–14.61
	May 17 to May 23, 2021	94	0–7	0.00–8.05	0–0	0.00–0.00
	May 24 to May 30, 2021	93	0–10	0.00–12.05	0–0	0.00–0.00
Tokushima						
	Apr 5 to Apr 11, 2021	52	0–5	0.00–10.64	0–0	0.00–0.00
	Apr 12 to Apr 18, 2021	47	0–1	0.00–2.17	0–0	0.00–0.00
	Apr 19 to Apr 25, 2021	60	1–15	2.22–33.33	0–0	0.00–0.00
	Apr 26 to May 2, 2021	56	0–12	0.00–27.27	0–0	0.00–0.00
	May 3 to May 9, 2021	77	21–34	48.84–79.07	0–0	0.00–0.00
	May 10 to May 16, 2021	63	8–21	19.05–50.00	0–0	0.00–0.00
	May 17 to May 23, 2021	43	0–3	0.00–7.50	0–0	0.00–0.00
	May 24 to May 30, 2021	56	4–17	10.26–43.59	0–0	0.00–0.00
Kagawa						
	Apr 5 to Apr 11, 2021	66	0–2	0.00–3.13	0–0	0.00–0.00
	Apr 12 to Apr 18, 2021	71	0–8	0.00–12.70	0–0	0.00–0.00
	Apr 19 to Apr 25, 2021	63	0–3	0.00–5.00	0–0	0.00–0.00
	Apr 26 to May 2, 2021	74	0–16	0.00–27.59	0–0	0.00–0.00
	May 3 to May 9, 2021	58	0–2	0.00–3.57	0–0	0.00–0.00
	May 10 to May 16, 2021	62	0–7	0.00–12.73	0–0	0.00–0.00
	May 17 to May 23, 2021	40	0–0	0.00–0.00	0–13	0.00–24.53
	May 24 to May 30, 2021	55	0–3	0.00–5.77	0–0	0.00–0.00
Ehime						
	Apr 5 to Apr 11, 2021	90	0–0	0.00–0.00	0–7	0.00–7.22
	Apr 12 to Apr 18, 2021	115	2–21	2.13–22.34	0–0	0.00–0.00
	Apr 19 to Apr 25, 2021	101	0–10	0.00–10.99	0–0	0.00–0.00
	Apr 26 to May 2, 2021	111	3–23	3.41–26.14	0–0	0.00–0.00
	May 3 to May 9, 2021	112	8–26	9.30–30.23	0–0	0.00–0.00
	May 10 to May 16, 2021	117	15–33	17.86–39.29	0–0	0.00–0.00
	May 17 to May 23, 2021	93	0–11	0.00–13.41	0–0	0.00–0.00
	May 24 to May 30, 2021	100	1–19	1.23–23.46	0–0	0.00–0.00
Kochi						
	Apr 5 to Apr 11, 2021	50	0–1	0.00–2.04	0–0	0.00–0.00
	Apr 12 to Apr 18, 2021	52	0–2	0.00–4.00	0–0	0.00–0.00
	Apr 19 to Apr 25, 2021	53	0–5	0.00–10.42	0–0	0.00–0.00
	Apr 26 to May 2, 2021	55	0–8	0.00–17.02	0–0	0.00–0.00
	May 3 to May 9, 2021	53	0–6	0.00–12.77	0–0	0.00–0.00
	May 10 to May 16, 2021	51	0–5	0.00–10.87	0–0	0.00–0.00
	May 17 to May 23, 2021	44	0–0	0.00–0.00	0–1	0.00–2.22
	May 24 to May 30, 2021	56	0–12	0.00–27.27	0–0	0.00–0.00
Fukuoka						
	Apr 5 to Apr 11, 2021	209	0–0	0.00–0.00	0–24	0.00–10.30
	Apr 12 to Apr 18, 2021	221	0–0	0.00–0.00	0–9	0.00–3.91
	Apr 19 to Apr 25, 2021	242	0–15	0.00–6.61	0–0	0.00–0.00

	Apr 26 to May 2, 2021	249	0–25	0.00–11.16	0–0	0.00–0.00
	May 3 to May 9, 2021	243	0–25	0.00–11.47	0–0	0.00–0.00
	May 10 to May 16, 2021	228	0–14	0.00–6.54	0–0	0.00–0.00
	May 17 to May 23, 2021	203	0–0	0.00–0.00	0–7	0.00–3.33
	May 24 to May 30, 2021	218	0–11	0.00–5.31	0–0	0.00–0.00
Saga						
	Apr 5 to Apr 11, 2021	47	0–2	0.00–4.44	0–0	0.00–0.00
	Apr 12 to Apr 18, 2021	42	0–0	0.00–0.00	0–2	0.00–4.55
	Apr 19 to Apr 25, 2021	48	0–4	0.00–9.09	0–0	0.00–0.00
	Apr 26 to May 2, 2021	31	0–0	0.00–0.00	0–12	0.00–27.91
	May 3 to May 9, 2021	45	0–3	0.00–7.14	0–0	0.00–0.00
	May 10 to May 16, 2021	31	0–0	0.00–0.00	0–10	0.00–24.39
	May 17 to May 23, 2021	39	0–0	0.00–0.00	0–2	0.00–4.88
	May 24 to May 30, 2021	46	0–5	0.00–12.20	0–0	0.00–0.00
Nagasaki						
	Apr 5 to Apr 11, 2021	84	0–0	0.00–0.00	0–0	0.00–0.00
	Apr 12 to Apr 18, 2021	76	0–0	0.00–0.00	0–9	0.00–10.59
	Apr 19 to Apr 25, 2021	89	0–5	0.00–5.95	0–0	0.00–0.00
	Apr 26 to May 2, 2021	88	0–7	0.00–8.64	0–0	0.00–0.00
	May 3 to May 9, 2021	96	0–18	0.00–23.08	0–0	0.00–0.00
	May 10 to May 16, 2021	92	0–15	0.00–19.48	0–0	0.00–0.00
	May 17 to May 23, 2021	78	0–2	0.00–2.63	0–0	0.00–0.00
	May 24 to May 30, 2021	90	0–16	0.00–21.62	0–0	0.00–0.00
Kumamoto						
	Apr 5 to Apr 11, 2021	97	0–0	0.00–0.00	0–3	0.00–3.00
	Apr 12 to Apr 18, 2021	104	0–7	0.00–7.22	0–0	0.00–0.00
	Apr 19 to Apr 25, 2021	102	0–6	0.00–6.25	0–0	0.00–0.00
	Apr 26 to May 2, 2021	111	0–16	0.00–16.84	0–0	0.00–0.00
	May 3 to May 9, 2021	106	0–14	0.00–15.22	0–0	0.00–0.00
	May 10 to May 16, 2021	102	0–15	0.00–17.24	0–0	0.00–0.00
	May 17 to May 23, 2021	98	0–13	0.00–15.29	0–0	0.00–0.00
	May 24 to May 30, 2021	110	8–26	9.52–30.95	0–0	0.00–0.00
Oita						
	Apr 5 to Apr 11, 2021	71	0–0	0.00–0.00	0–1	0.00–1.39
	Apr 12 to Apr 18, 2021	73	0–3	0.00–4.29	0–0	0.00–0.00
	Apr 19 to Apr 25, 2021	100	13–30	18.57–42.86	0–0	0.00–0.00
	Apr 26 to May 2, 2021	64	0–0	0.00–0.00	0–4	0.00–5.88
	May 3 to May 9, 2021	75	0–8	0.00–11.94	0–0	0.00–0.00
	May 10 to May 16, 2021	86	4–20	6.06–30.30	0–0	0.00–0.00
	May 17 to May 23, 2021	74	0–10	0.00–15.63	0–0	0.00–0.00
	May 24 to May 30, 2021	65	0–3	0.00–4.84	0–0	0.00–0.00
Miyazaki						
	Apr 5 to Apr 11, 2021	56	0–0	0.00–0.00	0–14	0.00–20.00
	Apr 12 to Apr 18, 2021	79	0–9	0.00–12.86	0–0	0.00–0.00
	Apr 19 to Apr 25, 2021	72	0–4	0.00–5.88	0–0	0.00–0.00
	Apr 26 to May 2, 2021	63	0–0	0.00–0.00	0–5	0.00–7.35

	May 3 to May 9, 2021	77	0–9	0.00–13.24	0–0	0.00–0.00
	May 10 to May 16, 2021	76	0–10	0.00–15.15	0–0	0.00–0.00
	May 17 to May 23, 2021	79	0–14	0.00–21.54	0–0	0.00–0.00
	May 24 to May 30, 2021	66	0–2	0.00–3.13	0–0	0.00–0.00
Kagoshima						
	Apr 5 to Apr 11, 2021	115	0–10	0.00–9.52	0–0	0.00–0.00
	Apr 12 to Apr 18, 2021	102	0–0	0.00–0.00	0–1	0.00–0.97
	Apr 19 to Apr 25, 2021	119	0–19	0.00–19.00	0–0	0.00–0.00
	Apr 26 to May 2, 2021	106	0–8	0.00–8.16	0–0	0.00–0.00
	May 3 to May 9, 2021	103	0–7	0.00–7.29	0–0	0.00–0.00
	May 10 to May 16, 2021	100	0–6	0.00–6.38	0–0	0.00–0.00
	May 17 to May 23, 2021	92	0–0	0.00–0.00	0–1	0.00–1.08
	May 24 to May 30, 2021	108	0–17	0.00–18.68	0–0	0.00–0.00
Okinawa						
	Apr 5 to Apr 11, 2021	63	0–3	0.00–5.00	0–0	0.00–0.00
	Apr 12 to Apr 18, 2021	68	0–8	0.00–13.33	0–0	0.00–0.00
	Apr 19 to Apr 25, 2021	62	0–3	0.00–5.08	0–0	0.00–0.00
	Apr 26 to May 2, 2021	55	0–0	0.00–0.00	0–4	0.00–6.78
	May 3 to May 9, 2021	56	0–0	0.00–0.00	0–4	0.00–6.67
	May 10 to May 16, 2021	62	0–3	0.00–5.08	0–0	0.00–0.00
	May 17 to May 23, 2021	55	0–0	0.00–0.00	0–4	0.00–6.78
	May 24 to May 30, 2021	62	0–4	0.00–6.90	0–0	0.00–0.00

Table A.10: Weekly number of observed and excess/exiguous deaths in Japan and 47 prefectures for circulatory disease-related deaths in hospitals and clinics from January 2020 through May 2021.

Prefecture	Week	Observed	Excess deaths	Percent excess	Exiguous deaths	Percent exiguous
Japan						
	Apr 5 to Apr 11, 2021	4799	0–176	0.00–3.81	0–0	0.00–0.00
	Apr 12 to Apr 18, 2021	4818	0–295	0.00–6.52	0–0	0.00–0.00
	Apr 19 to Apr 25, 2021	4903	99–456	2.23–10.25	0–0	0.00–0.00
	Apr 26 to May 2, 2021	4770	46–406	1.05–9.30	0–0	0.00–0.00
	May 3 to May 9, 2021	4736	99–454	2.31–10.60	0–0	0.00–0.00
	May 10 to May 16, 2021	4497	0–308	0.00–7.35	0–0	0.00–0.00
	May 17 to May 23, 2021	4290	0–213	0.00–5.22	0–0	0.00–0.00
	May 24 to May 30, 2021	4311	0–308	0.00–7.69	0–0	0.00–0.00
Hokkaido						
	Apr 5 to Apr 11, 2021	252	0–0	0.00–0.00	0–0	0.00–0.00
	Apr 12 to Apr 18, 2021	230	0–0	0.00–0.00	0–17	0.00–6.88
	Apr 19 to Apr 25, 2021	289	10–43	4.07–17.48	0–0	0.00–0.00
	Apr 26 to May 2, 2021	257	0–17	0.00–7.08	0–0	0.00–0.00
	May 3 to May 9, 2021	270	0–34	0.00–14.41	0–0	0.00–0.00
	May 10 to May 16, 2021	267	0–32	0.00–13.62	0–0	0.00–0.00
	May 17 to May 23, 2021	268	1–35	0.43–15.02	0–0	0.00–0.00
	May 24 to May 30, 2021	254	0–26	0.00–11.40	0–0	0.00–0.00
Aomori						
	Apr 5 to Apr 11, 2021	79	0–10	0.00–14.49	0–0	0.00–0.00
	Apr 12 to Apr 18, 2021	52	0–0	0.00–0.00	1–17	1.45–24.64
	Apr 19 to Apr 25, 2021	65	0–0	0.00–0.00	0–3	0.00–4.41
	Apr 26 to May 2, 2021	66	0–0	0.00–0.00	0–0	0.00–0.00
	May 3 to May 9, 2021	75	0–11	0.00–17.19	0–0	0.00–0.00
	May 10 to May 16, 2021	55	0–0	0.00–0.00	0–7	0.00–11.29
	May 17 to May 23, 2021	54	0–0	0.00–0.00	0–6	0.00–10.00
	May 24 to May 30, 2021	67	0–8	0.00–13.56	0–0	0.00–0.00
Iwate						
	Apr 5 to Apr 11, 2021	63	0–0	0.00–0.00	0–5	0.00–7.35
	Apr 12 to Apr 18, 2021	64	0–0	0.00–0.00	0–3	0.00–4.48
	Apr 19 to Apr 25, 2021	66	0–0	0.00–0.00	0–0	0.00–0.00
	Apr 26 to May 2, 2021	76	0–13	0.00–20.63	0–0	0.00–0.00
	May 3 to May 9, 2021	78	0–16	0.00–25.81	0–0	0.00–0.00
	May 10 to May 16, 2021	59	0–0	0.00–0.00	0–1	0.00–1.67
	May 17 to May 23, 2021	68	0–8	0.00–13.33	0–0	0.00–0.00
	May 24 to May 30, 2021	69	0–10	0.00–16.95	0–0	0.00–0.00
Miyagi						
	Apr 5 to Apr 11, 2021	104	0–9	0.00–9.47	0–0	0.00–0.00
	Apr 12 to Apr 18, 2021	96	0–2	0.00–2.13	0–0	0.00–0.00
	Apr 19 to Apr 25, 2021	96	0–4	0.00–4.35	0–0	0.00–0.00
	Apr 26 to May 2, 2021	108	0–17	0.00–18.68	0–0	0.00–0.00
	May 3 to May 9, 2021	87	0–0	0.00–0.00	0–2	0.00–2.25

	May 10 to May 16, 2021	78	0–0	0.00–0.00	0–11	0.00–12.36
	May 17 to May 23, 2021	85	0–0	0.00–0.00	0–1	0.00–1.16
	May 24 to May 30, 2021	85	0–1	0.00–1.19	0–0	0.00–0.00
Akita						
	Apr 5 to Apr 11, 2021	66	0–10	0.00–17.86	0–0	0.00–0.00
	Apr 12 to Apr 18, 2021	61	0–6	0.00–10.91	0–0	0.00–0.00
	Apr 19 to Apr 25, 2021	56	0–1	0.00–1.82	0–0	0.00–0.00
	Apr 26 to May 2, 2021	58	0–4	0.00–7.41	0–0	0.00–0.00
	May 3 to May 9, 2021	77	8–22	14.55–40.00	0–0	0.00–0.00
	May 10 to May 16, 2021	52	0–0	0.00–0.00	0–2	0.00–3.70
	May 17 to May 23, 2021	53	0–1	0.00–1.92	0–0	0.00–0.00
	May 24 to May 30, 2021	45	0–0	0.00–0.00	0–6	0.00–11.76
Yamagata						
	Apr 5 to Apr 11, 2021	59	0–1	0.00–1.72	0–0	0.00–0.00
	Apr 12 to Apr 18, 2021	61	0–3	0.00–5.17	0–0	0.00–0.00
	Apr 19 to Apr 25, 2021	63	0–5	0.00–8.62	0–0	0.00–0.00
	Apr 26 to May 2, 2021	43	0–0	0.00–0.00	0–13	0.00–23.21
	May 3 to May 9, 2021	55	0–1	0.00–1.85	0–0	0.00–0.00
	May 10 to May 16, 2021	60	0–7	0.00–13.21	0–0	0.00–0.00
	May 17 to May 23, 2021	51	0–0	0.00–0.00	0–0	0.00–0.00
	May 24 to May 30, 2021	49	0–0	0.00–0.00	0–2	0.00–3.92
Fukushima						
	Apr 5 to Apr 11, 2021	95	0–9	0.00–10.47	0–0	0.00–0.00
	Apr 12 to Apr 18, 2021	93	0–8	0.00–9.41	0–0	0.00–0.00
	Apr 19 to Apr 25, 2021	104	0–19	0.00–22.35	0–0	0.00–0.00
	Apr 26 to May 2, 2021	88	0–5	0.00–6.02	0–0	0.00–0.00
	May 3 to May 9, 2021	94	0–12	0.00–14.63	0–0	0.00–0.00
	May 10 to May 16, 2021	85	0–5	0.00–6.25	0–0	0.00–0.00
	May 17 to May 23, 2021	97	0–18	0.00–22.78	0–0	0.00–0.00
	May 24 to May 30, 2021	83	0–5	0.00–6.41	0–0	0.00–0.00
Ibaraki						
	Apr 5 to Apr 11, 2021	126	0–5	0.00–4.13	0–0	0.00–0.00
	Apr 12 to Apr 18, 2021	136	0–19	0.00–16.24	0–0	0.00–0.00
	Apr 19 to Apr 25, 2021	121	0–8	0.00–7.08	0–0	0.00–0.00
	Apr 26 to May 2, 2021	119	0–10	0.00–9.17	0–0	0.00–0.00
	May 3 to May 9, 2021	126	0–18	0.00–16.67	0–0	0.00–0.00
	May 10 to May 16, 2021	133	3–24	2.75–22.02	0–0	0.00–0.00
	May 17 to May 23, 2021	98	0–0	0.00–0.00	0–8	0.00–7.55
	May 24 to May 30, 2021	104	0–0	0.00–0.00	0–1	0.00–0.95
Tochigi						
	Apr 5 to Apr 11, 2021	79	0–3	0.00–3.95	0–0	0.00–0.00
	Apr 12 to Apr 18, 2021	98	7–24	9.46–32.43	0–0	0.00–0.00
	Apr 19 to Apr 25, 2021	73	0–0	0.00–0.00	0–0	0.00–0.00
	Apr 26 to May 2, 2021	81	0–10	0.00–14.08	0–0	0.00–0.00
	May 3 to May 9, 2021	83	0–14	0.00–20.29	0–0	0.00–0.00
	May 10 to May 16, 2021	70	0–3	0.00–4.48	0–0	0.00–0.00

	May 17 to May 23, 2021	74	0–8	0.00–12.12	0–0	0.00–0.00
	May 24 to May 30, 2021	58	0–0	0.00–0.00	0–6	0.00–9.38
Gunma						
	Apr 5 to Apr 11, 2021	81	0–0	0.00–0.00	0–2	0.00–2.41
	Apr 12 to Apr 18, 2021	100	1–19	1.23–23.46	0–0	0.00–0.00
	Apr 19 to Apr 25, 2021	76	0–0	0.00–0.00	0–2	0.00–2.56
	Apr 26 to May 2, 2021	88	0–11	0.00–14.29	0–0	0.00–0.00
	May 3 to May 9, 2021	85	0–10	0.00–13.33	0–0	0.00–0.00
	May 10 to May 16, 2021	94	2–20	2.70–27.03	0–0	0.00–0.00
	May 17 to May 23, 2021	81	0–7	0.00–9.46	0–0	0.00–0.00
	May 24 to May 30, 2021	83	0–10	0.00–13.70	0–0	0.00–0.00
Saitama						
	Apr 5 to Apr 11, 2021	243	0–8	0.00–3.40	0–0	0.00–0.00
	Apr 12 to Apr 18, 2021	235	0–5	0.00–2.17	0–0	0.00–0.00
	Apr 19 to Apr 25, 2021	255	0–29	0.00–12.83	0–0	0.00–0.00
	Apr 26 to May 2, 2021	268	11–45	4.93–20.18	0–0	0.00–0.00
	May 3 to May 9, 2021	264	10–46	4.59–21.10	0–0	0.00–0.00
	May 10 to May 16, 2021	238	0–24	0.00–11.21	0–0	0.00–0.00
	May 17 to May 23, 2021	208	0–0	0.00–0.00	0–1	0.00–0.48
	May 24 to May 30, 2021	225	0–20	0.00–9.76	0–0	0.00–0.00
Chiba						
	Apr 5 to Apr 11, 2021	256	8–41	3.72–19.07	0–0	0.00–0.00
	Apr 12 to Apr 18, 2021	233	0–25	0.00–12.02	0–0	0.00–0.00
	Apr 19 to Apr 25, 2021	228	0–25	0.00–12.32	0–0	0.00–0.00
	Apr 26 to May 2, 2021	244	12–44	6.00–22.00	0–0	0.00–0.00
	May 3 to May 9, 2021	221	0–27	0.00–13.92	0–0	0.00–0.00
	May 10 to May 16, 2021	213	0–20	0.00–10.36	0–0	0.00–0.00
	May 17 to May 23, 2021	209	0–19	0.00–10.00	0–0	0.00–0.00
	May 24 to May 30, 2021	192	0–5	0.00–2.67	0–0	0.00–0.00
Tokyo						
	Apr 5 to Apr 11, 2021	427	0–36	0.00–9.21	0–0	0.00–0.00
	Apr 12 to Apr 18, 2021	400	0–16	0.00–4.17	0–0	0.00–0.00
	Apr 19 to Apr 25, 2021	412	0–33	0.00–8.71	0–0	0.00–0.00
	Apr 26 to May 2, 2021	413	0–47	0.00–12.84	0–0	0.00–0.00
	May 3 to May 9, 2021	389	0–28	0.00–7.76	0–0	0.00–0.00
	May 10 to May 16, 2021	361	0–9	0.00–2.56	0–0	0.00–0.00
	May 17 to May 23, 2021	333	0–0	0.00–0.00	0–12	0.00–3.48
	May 24 to May 30, 2021	334	0–0	0.00–0.00	0–6	0.00–1.76
Kanagawa						
	Apr 5 to Apr 11, 2021	269	0–6	0.00–2.28	0–0	0.00–0.00
	Apr 12 to Apr 18, 2021	287	0–31	0.00–12.11	0–0	0.00–0.00
	Apr 19 to Apr 25, 2021	266	0–16	0.00–6.40	0–0	0.00–0.00
	Apr 26 to May 2, 2021	279	3–35	1.23–14.34	0–0	0.00–0.00
	May 3 to May 9, 2021	268	0–28	0.00–11.67	0–0	0.00–0.00
	May 10 to May 16, 2021	232	0–0	0.00–0.00	0–6	0.00–2.52
	May 17 to May 23, 2021	280	17–48	7.33–20.69	0–0	0.00–0.00

	May 24 to May 30, 2021	249	0–21	0.00–9.21	0–0	0.00–0.00
Niigata						
	Apr 5 to Apr 11, 2021	97	0–0	0.00–0.00	0–9	0.00–8.49
	Apr 12 to Apr 18, 2021	104	0–0	0.00–0.00	0–2	0.00–1.89
	Apr 19 to Apr 25, 2021	109	0–5	0.00–4.81	0–0	0.00–0.00
	Apr 26 to May 2, 2021	112	0–10	0.00–9.80	0–0	0.00–0.00
	May 3 to May 9, 2021	106	0–6	0.00–6.00	0–0	0.00–0.00
	May 10 to May 16, 2021	86	0–0	0.00–0.00	0–12	0.00–12.24
	May 17 to May 23, 2021	91	0–0	0.00–0.00	0–6	0.00–6.19
	May 24 to May 30, 2021	89	0–0	0.00–0.00	0–7	0.00–7.29
Toyama						
	Apr 5 to Apr 11, 2021	62	4–18	9.09–40.91	0–0	0.00–0.00
	Apr 12 to Apr 18, 2021	56	0–12	0.00–27.27	0–0	0.00–0.00
	Apr 19 to Apr 25, 2021	39	0–0	0.00–0.00	0–3	0.00–7.14
	Apr 26 to May 2, 2021	49	0–8	0.00–19.51	0–0	0.00–0.00
	May 3 to May 9, 2021	45	0–5	0.00–12.50	0–0	0.00–0.00
	May 10 to May 16, 2021	40	0–0	0.00–0.00	0–0	0.00–0.00
	May 17 to May 23, 2021	38	0–0	0.00–0.00	0–0	0.00–0.00
	May 24 to May 30, 2021	45	0–8	0.00–21.62	0–0	0.00–0.00
Ishikawa						
	Apr 5 to Apr 11, 2021	49	0–1	0.00–2.08	0–0	0.00–0.00
	Apr 12 to Apr 18, 2021	52	0–5	0.00–10.64	0–0	0.00–0.00
	Apr 19 to Apr 25, 2021	51	0–6	0.00–13.33	0–0	0.00–0.00
	Apr 26 to May 2, 2021	55	0–13	0.00–30.95	0–0	0.00–0.00
	May 3 to May 9, 2021	59	5–19	12.50–47.50	0–0	0.00–0.00
	May 10 to May 16, 2021	53	0–13	0.00–32.50	0–0	0.00–0.00
	May 17 to May 23, 2021	40	0–1	0.00–2.56	0–0	0.00–0.00
	May 24 to May 30, 2021	51	0–13	0.00–34.21	0–0	0.00–0.00
Fukui						
	Apr 5 to Apr 11, 2021	29	0–0	0.00–0.00	0–4	0.00–12.12
	Apr 12 to Apr 18, 2021	41	0–9	0.00–28.13	0–0	0.00–0.00
	Apr 19 to Apr 25, 2021	34	0–2	0.00–6.25	0–0	0.00–0.00
	Apr 26 to May 2, 2021	46	4–15	12.90–48.39	0–0	0.00–0.00
	May 3 to May 9, 2021	40	0–10	0.00–33.33	0–0	0.00–0.00
	May 10 to May 16, 2021	27	0–0	0.00–0.00	0–3	0.00–10.00
	May 17 to May 23, 2021	32	0–2	0.00–6.67	0–0	0.00–0.00
	May 24 to May 30, 2021	36	0–7	0.00–24.14	0–0	0.00–0.00
Yamanashi						
	Apr 5 to Apr 11, 2021	26	0–0	0.00–0.00	0–11	0.00–29.73
	Apr 12 to Apr 18, 2021	49	0–13	0.00–36.11	0–0	0.00–0.00
	Apr 19 to Apr 25, 2021	40	0–6	0.00–17.65	0–0	0.00–0.00
	Apr 26 to May 2, 2021	30	0–0	0.00–0.00	0–4	0.00–11.76
	May 3 to May 9, 2021	30	0–0	0.00–0.00	0–3	0.00–9.09
	May 10 to May 16, 2021	40	0–8	0.00–25.00	0–0	0.00–0.00
	May 17 to May 23, 2021	33	0–1	0.00–3.13	0–0	0.00–0.00
	May 24 to May 30, 2021	32	0–1	0.00–3.23	0–0	0.00–0.00

Nagano						
	Apr 5 to Apr 11, 2021	86	0–0	0.00–0.00	0–7	0.00–7.53
	Apr 12 to Apr 18, 2021	78	0–0	0.00–0.00	0–14	0.00–15.22
	Apr 19 to Apr 25, 2021	83	0–0	0.00–0.00	0–8	0.00–8.79
	Apr 26 to May 2, 2021	89	0–1	0.00–1.14	0–0	0.00–0.00
	May 3 to May 9, 2021	91	0–4	0.00–4.60	0–0	0.00–0.00
	May 10 to May 16, 2021	93	0–7	0.00–8.14	0–0	0.00–0.00
	May 17 to May 23, 2021	67	0–0	0.00–0.00	0–16	0.00–19.28
	May 24 to May 30, 2021	70	0–0	0.00–0.00	0–10	0.00–12.50
Gifu						
	Apr 5 to Apr 11, 2021	76	0–0	0.00–0.00	0–0	0.00–0.00
	Apr 12 to Apr 18, 2021	76	0–2	0.00–2.70	0–0	0.00–0.00
	Apr 19 to Apr 25, 2021	92	1–20	1.39–27.78	0–0	0.00–0.00
	Apr 26 to May 2, 2021	71	0–3	0.00–4.41	0–0	0.00–0.00
	May 3 to May 9, 2021	64	0–0	0.00–0.00	0–3	0.00–4.48
	May 10 to May 16, 2021	73	0–8	0.00–12.31	0–0	0.00–0.00
	May 17 to May 23, 2021	74	0–11	0.00–17.46	0–0	0.00–0.00
	May 24 to May 30, 2021	70	0–6	0.00–9.38	0–0	0.00–0.00
Shizuoka						
	Apr 5 to Apr 11, 2021	143	0–11	0.00–8.33	0–0	0.00–0.00
	Apr 12 to Apr 18, 2021	154	0–22	0.00–16.67	0–0	0.00–0.00
	Apr 19 to Apr 25, 2021	139	0–10	0.00–7.75	0–0	0.00–0.00
	Apr 26 to May 2, 2021	139	0–13	0.00–10.32	0–0	0.00–0.00
	May 3 to May 9, 2021	117	0–0	0.00–0.00	0–5	0.00–4.10
	May 10 to May 16, 2021	132	0–12	0.00–10.00	0–0	0.00–0.00
	May 17 to May 23, 2021	116	0–0	0.00–0.00	0–2	0.00–1.69
	May 24 to May 30, 2021	119	0–2	0.00–1.71	0–0	0.00–0.00
Aichi						
	Apr 5 to Apr 11, 2021	214	0–2	0.00–0.94	0–0	0.00–0.00
	Apr 12 to Apr 18, 2021	203	0–0	0.00–0.00	0–5	0.00–2.40
	Apr 19 to Apr 25, 2021	235	0–30	0.00–14.63	0–0	0.00–0.00
	Apr 26 to May 2, 2021	218	0–18	0.00–9.00	0–0	0.00–0.00
	May 3 to May 9, 2021	198	0–1	0.00–0.51	0–0	0.00–0.00
	May 10 to May 16, 2021	214	0–24	0.00–12.63	0–0	0.00–0.00
	May 17 to May 23, 2021	176	0–0	0.00–0.00	0–9	0.00–4.86
	May 24 to May 30, 2021	189	0–7	0.00–3.85	0–0	0.00–0.00
Mie						
	Apr 5 to Apr 11, 2021	74	0–6	0.00–8.82	0–0	0.00–0.00
	Apr 12 to Apr 18, 2021	60	0–0	0.00–0.00	0–5	0.00–7.69
	Apr 19 to Apr 25, 2021	56	0–0	0.00–0.00	0–7	0.00–11.11
	Apr 26 to May 2, 2021	85	7–25	11.67–41.67	0–0	0.00–0.00
	May 3 to May 9, 2021	54	0–0	0.00–0.00	0–5	0.00–8.47
	May 10 to May 16, 2021	62	0–5	0.00–8.77	0–0	0.00–0.00
	May 17 to May 23, 2021	61	0–5	0.00–8.93	0–0	0.00–0.00
	May 24 to May 30, 2021	55	0–0	0.00–0.00	0–0	0.00–0.00
Shiga						

	Apr 5 to Apr 11, 2021	48	0–3	0.00–6.67	0–0	0.00–0.00
	Apr 12 to Apr 18, 2021	54	0–10	0.00–22.73	0–0	0.00–0.00
	Apr 19 to Apr 25, 2021	40	0–0	0.00–0.00	0–3	0.00–6.98
	Apr 26 to May 2, 2021	38	0–0	0.00–0.00	0–4	0.00–9.52
	May 3 to May 9, 2021	47	0–6	0.00–14.63	0–0	0.00–0.00
	May 10 to May 16, 2021	31	0–0	0.00–0.00	0–8	0.00–20.51
	May 17 to May 23, 2021	38	0–1	0.00–2.70	0–0	0.00–0.00
	May 24 to May 30, 2021	46	0–11	0.00–31.43	0–0	0.00–0.00
Kyoto						
	Apr 5 to Apr 11, 2021	112	2–22	2.22–24.44	0–0	0.00–0.00
	Apr 12 to Apr 18, 2021	93	0–5	0.00–5.68	0–0	0.00–0.00
	Apr 19 to Apr 25, 2021	98	0–10	0.00–11.36	0–0	0.00–0.00
	Apr 26 to May 2, 2021	112	6–26	6.98–30.23	0–0	0.00–0.00
	May 3 to May 9, 2021	92	0–9	0.00–10.84	0–0	0.00–0.00
	May 10 to May 16, 2021	83	0–3	0.00–3.75	0–0	0.00–0.00
	May 17 to May 23, 2021	93	0–13	0.00–16.25	0–0	0.00–0.00
	May 24 to May 30, 2021	84	0–6	0.00–7.69	0–0	0.00–0.00
Osaka						
	Apr 5 to Apr 11, 2021	271	0–0	0.00–0.00	0–4	0.00–1.45
	Apr 12 to Apr 18, 2021	282	0–8	0.00–2.92	0–0	0.00–0.00
	Apr 19 to Apr 25, 2021	310	6–43	2.25–16.10	0–0	0.00–0.00
	Apr 26 to May 2, 2021	261	0–0	0.00–0.00	0–0	0.00–0.00
	May 3 to May 9, 2021	294	1–39	0.39–15.29	0–0	0.00–0.00
	May 10 to May 16, 2021	251	0–0	0.00–0.00	0–0	0.00–0.00
	May 17 to May 23, 2021	263	0–14	0.00–5.62	0–0	0.00–0.00
	May 24 to May 30, 2021	253	0–10	0.00–4.12	0–0	0.00–0.00
Hyogo						
	Apr 5 to Apr 11, 2021	200	0–21	0.00–11.73	0–0	0.00–0.00
	Apr 12 to Apr 18, 2021	187	0–12	0.00–6.86	0–0	0.00–0.00
	Apr 19 to Apr 25, 2021	197	0–27	0.00–15.88	0–0	0.00–0.00
	Apr 26 to May 2, 2021	186	0–21	0.00–12.73	0–0	0.00–0.00
	May 3 to May 9, 2021	186	0–24	0.00–14.81	0–0	0.00–0.00
	May 10 to May 16, 2021	191	3–31	1.88–19.38	0–0	0.00–0.00
	May 17 to May 23, 2021	174	0–17	0.00–10.83	0–0	0.00–0.00
	May 24 to May 30, 2021	173	0–17	0.00–10.90	0–0	0.00–0.00
Nara						
	Apr 5 to Apr 11, 2021	40	0–0	0.00–0.00	0–6	0.00–13.04
	Apr 12 to Apr 18, 2021	60	3–16	6.82–36.36	0–0	0.00–0.00
	Apr 19 to Apr 25, 2021	49	0–6	0.00–13.95	0–0	0.00–0.00
	Apr 26 to May 2, 2021	36	0–0	0.00–0.00	0–6	0.00–14.29
	May 3 to May 9, 2021	44	0–3	0.00–7.32	0–0	0.00–0.00
	May 10 to May 16, 2021	38	0–0	0.00–0.00	0–3	0.00–7.32
	May 17 to May 23, 2021	44	0–4	0.00–10.00	0–0	0.00–0.00
	May 24 to May 30, 2021	43	0–4	0.00–10.26	0–0	0.00–0.00
Wakayama						
	Apr 5 to Apr 11, 2021	48	0–8	0.00–20.00	0–0	0.00–0.00

	Apr 12 to Apr 18, 2021	33	0–0	0.00–0.00	0–7	0.00–17.50
	Apr 19 to Apr 25, 2021	38	0–0	0.00–0.00	0–1	0.00–2.56
	Apr 26 to May 2, 2021	31	0–0	0.00–0.00	0–6	0.00–16.22
	May 3 to May 9, 2021	37	0–2	0.00–5.71	0–0	0.00–0.00
	May 10 to May 16, 2021	45	0–11	0.00–32.35	0–0	0.00–0.00
	May 17 to May 23, 2021	39	0–6	0.00–18.18	0–0	0.00–0.00
	May 24 to May 30, 2021	32	0–0	0.00–0.00	0–0	0.00–0.00
Tottori						
	Apr 5 to Apr 11, 2021	17	0–0	0.00–0.00	0–6	0.00–26.09
	Apr 12 to Apr 18, 2021	22	0–0	0.00–0.00	0–0	0.00–0.00
	Apr 19 to Apr 25, 2021	20	0–0	0.00–0.00	0–2	0.00–9.09
	Apr 26 to May 2, 2021	21	0–0	0.00–0.00	0–1	0.00–4.55
	May 3 to May 9, 2021	26	0–4	0.00–18.18	0–0	0.00–0.00
	May 10 to May 16, 2021	25	0–4	0.00–19.05	0–0	0.00–0.00
	May 17 to May 23, 2021	14	0–0	0.00–0.00	0–6	0.00–30.00
	May 24 to May 30, 2021	21	0–2	0.00–10.53	0–0	0.00–0.00
Shimane						
	Apr 5 to Apr 11, 2021	26	0–0	0.00–0.00	0–5	0.00–16.13
	Apr 12 to Apr 18, 2021	36	0–5	0.00–16.13	0–0	0.00–0.00
	Apr 19 to Apr 25, 2021	25	0–0	0.00–0.00	0–6	0.00–19.35
	Apr 26 to May 2, 2021	28	0–0	0.00–0.00	0–2	0.00–6.67
	May 3 to May 9, 2021	30	0–1	0.00–3.45	0–0	0.00–0.00
	May 10 to May 16, 2021	31	0–3	0.00–10.71	0–0	0.00–0.00
	May 17 to May 23, 2021	37	0–10	0.00–37.04	0–0	0.00–0.00
	May 24 to May 30, 2021	21	0–0	0.00–0.00	0–5	0.00–19.23
Okayama						
	Apr 5 to Apr 11, 2021	77	0–5	0.00–6.94	0–0	0.00–0.00
	Apr 12 to Apr 18, 2021	74	0–3	0.00–4.23	0–0	0.00–0.00
	Apr 19 to Apr 25, 2021	78	0–9	0.00–13.04	0–0	0.00–0.00
	Apr 26 to May 2, 2021	76	0–8	0.00–11.76	0–0	0.00–0.00
	May 3 to May 9, 2021	69	0–1	0.00–1.47	0–0	0.00–0.00
	May 10 to May 16, 2021	63	0–0	0.00–0.00	0–4	0.00–5.97
	May 17 to May 23, 2021	71	0–6	0.00–9.23	0–0	0.00–0.00
	May 24 to May 30, 2021	69	0–7	0.00–11.29	0–0	0.00–0.00
Hiroshima						
	Apr 5 to Apr 11, 2021	120	0–19	0.00–18.81	0–0	0.00–0.00
	Apr 12 to Apr 18, 2021	115	0–14	0.00–13.86	0–0	0.00–0.00
	Apr 19 to Apr 25, 2021	111	0–12	0.00–12.12	0–0	0.00–0.00
	Apr 26 to May 2, 2021	108	0–10	0.00–10.20	0–0	0.00–0.00
	May 3 to May 9, 2021	116	0–18	0.00–18.37	0–0	0.00–0.00
	May 10 to May 16, 2021	113	0–17	0.00–17.71	0–0	0.00–0.00
	May 17 to May 23, 2021	100	0–9	0.00–9.89	0–0	0.00–0.00
	May 24 to May 30, 2021	100	0–10	0.00–11.11	0–0	0.00–0.00
Yamaguchi						
	Apr 5 to Apr 11, 2021	59	0–0	0.00–0.00	0–8	0.00–11.94
	Apr 12 to Apr 18, 2021	88	3–21	4.48–31.34	0–0	0.00–0.00

	Apr 19 to Apr 25, 2021	71	0–5	0.00–7.58	0–0	0.00–0.00
	Apr 26 to May 2, 2021	50	0–0	0.00–0.00	0–15	0.00–23.08
	May 3 to May 9, 2021	72	0–8	0.00–12.50	0–0	0.00–0.00
	May 10 to May 16, 2021	54	0–0	0.00–0.00	0–8	0.00–12.90
	May 17 to May 23, 2021	61	0–1	0.00–1.67	0–0	0.00–0.00
	May 24 to May 30, 2021	69	0–11	0.00–18.97	0–0	0.00–0.00
Tokushima						
	Apr 5 to Apr 11, 2021	37	0–3	0.00–8.82	0–0	0.00–0.00
	Apr 12 to Apr 18, 2021	29	0–0	0.00–0.00	0–6	0.00–17.14
	Apr 19 to Apr 25, 2021	47	2–14	6.06–42.42	0–0	0.00–0.00
	Apr 26 to May 2, 2021	30	0–0	0.00–0.00	0–3	0.00–9.09
	May 3 to May 9, 2021	50	6–18	18.75–56.25	0–0	0.00–0.00
	May 10 to May 16, 2021	45	2–13	6.25–40.63	0–0	0.00–0.00
	May 17 to May 23, 2021	22	0–0	0.00–0.00	0–8	0.00–26.67
	May 24 to May 30, 2021	42	3–13	10.34–44.83	0–0	0.00–0.00
Kagawa						
	Apr 5 to Apr 11, 2021	40	0–0	0.00–0.00	0–0	0.00–0.00
	Apr 12 to Apr 18, 2021	41	0–1	0.00–2.50	0–0	0.00–0.00
	Apr 19 to Apr 25, 2021	35	0–0	0.00–0.00	0–3	0.00–7.89
	Apr 26 to May 2, 2021	47	0–10	0.00–27.03	0–0	0.00–0.00
	May 3 to May 9, 2021	30	0–0	0.00–0.00	0–5	0.00–14.29
	May 10 to May 16, 2021	39	0–4	0.00–11.43	0–0	0.00–0.00
	May 17 to May 23, 2021	24	0–0	0.00–0.00	0–10	0.00–29.41
	May 24 to May 30, 2021	39	0–6	0.00–18.18	0–0	0.00–0.00
Ehime						
	Apr 5 to Apr 11, 2021	64	0–0	0.00–0.00	0–1	0.00–1.54
	Apr 12 to Apr 18, 2021	75	0–11	0.00–17.19	0–0	0.00–0.00
	Apr 19 to Apr 25, 2021	59	0–0	0.00–0.00	0–4	0.00–6.35
	Apr 26 to May 2, 2021	74	0–13	0.00–21.31	0–0	0.00–0.00
	May 3 to May 9, 2021	71	0–12	0.00–20.34	0–0	0.00–0.00
	May 10 to May 16, 2021	81	8–23	13.79–39.66	0–0	0.00–0.00
	May 17 to May 23, 2021	66	0–10	0.00–17.86	0–0	0.00–0.00
	May 24 to May 30, 2021	73	3–18	5.45–32.73	0–0	0.00–0.00
Kochi						
	Apr 5 to Apr 11, 2021	36	0–0	0.00–0.00	0–3	0.00–7.69
	Apr 12 to Apr 18, 2021	47	0–8	0.00–20.51	0–0	0.00–0.00
	Apr 19 to Apr 25, 2021	43	0–5	0.00–13.16	0–0	0.00–0.00
	Apr 26 to May 2, 2021	38	0–0	0.00–0.00	0–0	0.00–0.00
	May 3 to May 9, 2021	36	0–0	0.00–0.00	0–1	0.00–2.70
	May 10 to May 16, 2021	39	0–3	0.00–8.33	0–0	0.00–0.00
	May 17 to May 23, 2021	32	0–0	0.00–0.00	0–3	0.00–8.57
	May 24 to May 30, 2021	39	0–5	0.00–14.71	0–0	0.00–0.00
Fukuoka						
	Apr 5 to Apr 11, 2021	163	0–0	0.00–0.00	0–21	0.00–11.41
	Apr 12 to Apr 18, 2021	181	0–1	0.00–0.56	0–0	0.00–0.00
	Apr 19 to Apr 25, 2021	200	0–22	0.00–12.36	0–0	0.00–0.00

	Apr 26 to May 2, 2021	189	0–13	0.00–7.39	0–0	0.00–0.00
	May 3 to May 9, 2021	179	0–7	0.00–4.07	0–0	0.00–0.00
	May 10 to May 16, 2021	179	0–10	0.00–5.92	0–0	0.00–0.00
	May 17 to May 23, 2021	159	0–0	0.00–0.00	0–7	0.00–4.22
	May 24 to May 30, 2021	175	0–11	0.00–6.71	0–0	0.00–0.00
Saga						
	Apr 5 to Apr 11, 2021	36	0–3	0.00–9.09	0–0	0.00–0.00
	Apr 12 to Apr 18, 2021	32	0–0	0.00–0.00	0–0	0.00–0.00
	Apr 19 to Apr 25, 2021	36	0–3	0.00–9.09	0–0	0.00–0.00
	Apr 26 to May 2, 2021	27	0–0	0.00–0.00	0–6	0.00–18.18
	May 3 to May 9, 2021	33	0–1	0.00–3.13	0–0	0.00–0.00
	May 10 to May 16, 2021	22	0–0	0.00–0.00	0–9	0.00–29.03
	May 17 to May 23, 2021	31	0–0	0.00–0.00	0–0	0.00–0.00
	May 24 to May 30, 2021	39	0–7	0.00–21.88	0–0	0.00–0.00
Nagasaki						
	Apr 5 to Apr 11, 2021	63	0–0	0.00–0.00	0–4	0.00–5.97
	Apr 12 to Apr 18, 2021	58	0–0	0.00–0.00	0–8	0.00–12.12
	Apr 19 to Apr 25, 2021	65	0–0	0.00–0.00	0–0	0.00–0.00
	Apr 26 to May 2, 2021	65	0–2	0.00–3.17	0–0	0.00–0.00
	May 3 to May 9, 2021	63	0–2	0.00–3.28	0–0	0.00–0.00
	May 10 to May 16, 2021	63	0–3	0.00–5.00	0–0	0.00–0.00
	May 17 to May 23, 2021	62	0–3	0.00–5.08	0–0	0.00–0.00
	May 24 to May 30, 2021	64	0–6	0.00–10.34	0–0	0.00–0.00
Kumamoto						
	Apr 5 to Apr 11, 2021	70	0–0	0.00–0.00	0–3	0.00–4.11
	Apr 12 to Apr 18, 2021	70	0–0	0.00–0.00	0–2	0.00–2.78
	Apr 19 to Apr 25, 2021	80	0–9	0.00–12.68	0–0	0.00–0.00
	Apr 26 to May 2, 2021	70	0–0	0.00–0.00	0–1	0.00–1.41
	May 3 to May 9, 2021	69	0–1	0.00–1.47	0–0	0.00–0.00
	May 10 to May 16, 2021	72	0–7	0.00–10.77	0–0	0.00–0.00
	May 17 to May 23, 2021	69	0–5	0.00–7.81	0–0	0.00–0.00
	May 24 to May 30, 2021	73	0–9	0.00–14.06	0–0	0.00–0.00
Oita						
	Apr 5 to Apr 11, 2021	51	0–2	0.00–4.08	0–0	0.00–0.00
	Apr 12 to Apr 18, 2021	44	0–0	0.00–0.00	0–4	0.00–8.33
	Apr 19 to Apr 25, 2021	67	4–18	8.16–36.73	0–0	0.00–0.00
	Apr 26 to May 2, 2021	38	0–0	0.00–0.00	0–10	0.00–20.83
	May 3 to May 9, 2021	44	0–0	0.00–0.00	0–2	0.00–4.35
	May 10 to May 16, 2021	58	0–13	0.00–28.89	0–0	0.00–0.00
	May 17 to May 23, 2021	53	0–9	0.00–20.45	0–0	0.00–0.00
	May 24 to May 30, 2021	41	0–0	0.00–0.00	0–2	0.00–4.65
Miyazaki						
	Apr 5 to Apr 11, 2021	44	0–0	0.00–0.00	0–7	0.00–13.73
	Apr 12 to Apr 18, 2021	57	0–7	0.00–14.00	0–0	0.00–0.00
	Apr 19 to Apr 25, 2021	55	0–6	0.00–12.24	0–0	0.00–0.00
	Apr 26 to May 2, 2021	48	0–0	0.00–0.00	0–1	0.00–2.04

	May 3 to May 9, 2021	56	0–7	0.00–14.29	0–0	0.00–0.00
	May 10 to May 16, 2021	53	0–5	0.00–10.42	0–0	0.00–0.00
	May 17 to May 23, 2021	54	0–7	0.00–14.89	0–0	0.00–0.00
	May 24 to May 30, 2021	44	0–0	0.00–0.00	0–2	0.00–4.35
Kagoshima						
	Apr 5 to Apr 11, 2021	87	0–7	0.00–8.75	0–0	0.00–0.00
	Apr 12 to Apr 18, 2021	72	0–0	0.00–0.00	0–6	0.00–7.69
	Apr 19 to Apr 25, 2021	87	0–12	0.00–16.00	0–0	0.00–0.00
	Apr 26 to May 2, 2021	76	0–2	0.00–2.70	0–0	0.00–0.00
	May 3 to May 9, 2021	78	0–5	0.00–6.85	0–0	0.00–0.00
	May 10 to May 16, 2021	74	0–2	0.00–2.78	0–0	0.00–0.00
	May 17 to May 23, 2021	63	0–0	0.00–0.00	0–8	0.00–11.27
	May 24 to May 30, 2021	78	0–8	0.00–11.43	0–0	0.00–0.00
Okinawa						
	Apr 5 to Apr 11, 2021	35	0–0	0.00–0.00	0–7	0.00–16.67
	Apr 12 to Apr 18, 2021	49	0–7	0.00–16.67	0–0	0.00–0.00
	Apr 19 to Apr 25, 2021	44	0–3	0.00–7.32	0–0	0.00–0.00
	Apr 26 to May 2, 2021	39	0–0	0.00–0.00	0–2	0.00–4.88
	May 3 to May 9, 2021	38	0–0	0.00–0.00	0–3	0.00–7.32
	May 10 to May 16, 2021	30	0–0	0.00–0.00	0–10	0.00–25.00
	May 17 to May 23, 2021	34	0–0	0.00–0.00	0–6	0.00–15.00
	May 24 to May 30, 2021	40	0–2	0.00–5.26	0–0	0.00–0.00

Table A.11: Weekly number of observed and excess/exiguous deaths in Japan and 47 prefectures for circulatory disease-related deaths in nursing homes and elderly care facilities from January 2020 through May 2021.

Prefecture	Week	Observed	Excess deaths	Percent excess	Exiguous deaths	Percent exiguous
Japan						
	Apr 5 to Apr 11, 2021	821	0–22	0.00–2.75	0–0	0.00–0.00
	Apr 12 to Apr 18, 2021	871	12–82	1.52–10.39	0–0	0.00–0.00
	Apr 19 to Apr 25, 2021	857	0–71	0.00–9.03	0–0	0.00–0.00
	Apr 26 to May 2, 2021	941	98–166	12.65–21.42	0–0	0.00–0.00
	May 3 to May 9, 2021	935	105–175	13.82–23.03	0–0	0.00–0.00
	May 10 to May 16, 2021	896	91–159	12.35–21.57	0–0	0.00–0.00
	May 17 to May 23, 2021	862	61–129	8.32–17.60	0–0	0.00–0.00
	May 24 to May 30, 2021	793	0–65	0.00–8.93	0–0	0.00–0.00
Hokkaido						
	Apr 5 to Apr 11, 2021	18	0–0	0.00–0.00	0–3	0.00–14.29
	Apr 12 to Apr 18, 2021	21	0–0	0.00–0.00	0–0	0.00–0.00
	Apr 19 to Apr 25, 2021	24	0–3	0.00–14.29	0–0	0.00–0.00
	Apr 26 to May 2, 2021	18	0–0	0.00–0.00	0–3	0.00–14.29
	May 3 to May 9, 2021	24	0–3	0.00–14.29	0–0	0.00–0.00
	May 10 to May 16, 2021	22	0–1	0.00–4.76	0–0	0.00–0.00
	May 17 to May 23, 2021	25	0–4	0.00–19.05	0–0	0.00–0.00
	May 24 to May 30, 2021	23	0–3	0.00–15.00	0–0	0.00–0.00
Aomori						
	Apr 5 to Apr 11, 2021	19	0–5	0.00–35.71	0–0	0.00–0.00
	Apr 12 to Apr 18, 2021	19	0–5	0.00–35.71	0–0	0.00–0.00
	Apr 19 to Apr 25, 2021	15	0–1	0.00–7.14	0–0	0.00–0.00
	Apr 26 to May 2, 2021	14	0–0	0.00–0.00	0–0	0.00–0.00
	May 3 to May 9, 2021	12	0–0	0.00–0.00	0–1	0.00–7.69
	May 10 to May 16, 2021	14	0–1	0.00–7.69	0–0	0.00–0.00
	May 17 to May 23, 2021	19	0–5	0.00–35.71	0–0	0.00–0.00
	May 24 to May 30, 2021	16	0–2	0.00–14.29	0–0	0.00–0.00
Iwate						
	Apr 5 to Apr 11, 2021	16	0–3	0.00–23.08	0–0	0.00–0.00
	Apr 12 to Apr 18, 2021	14	0–1	0.00–7.69	0–0	0.00–0.00
	Apr 19 to Apr 25, 2021	12	0–0	0.00–0.00	0–1	0.00–7.69
	Apr 26 to May 2, 2021	17	0–4	0.00–30.77	0–0	0.00–0.00
	May 3 to May 9, 2021	19	0–6	0.00–46.15	0–0	0.00–0.00
	May 10 to May 16, 2021	10	0–0	0.00–0.00	0–2	0.00–16.67
	May 17 to May 23, 2021	18	0–6	0.00–50.00	0–0	0.00–0.00
	May 24 to May 30, 2021	14	0–2	0.00–16.67	0–0	0.00–0.00
Miyagi						
	Apr 5 to Apr 11, 2021	17	0–2	0.00–13.33	0–0	0.00–0.00
	Apr 12 to Apr 18, 2021	13	0–0	0.00–0.00	0–1	0.00–7.14
	Apr 19 to Apr 25, 2021	13	0–0	0.00–0.00	0–1	0.00–7.14
	Apr 26 to May 2, 2021	19	0–5	0.00–35.71	0–0	0.00–0.00

	May 3 to May 9, 2021	16	0–2	0.00–14.29	0–0	0.00–0.00
	May 10 to May 16, 2021	14	0–0	0.00–0.00	0–0	0.00–0.00
	May 17 to May 23, 2021	17	0–3	0.00–21.43	0–0	0.00–0.00
	May 24 to May 30, 2021	19	0–5	0.00–35.71	0–0	0.00–0.00
Akita						
	Apr 5 to Apr 11, 2021	19	1–8	9.09–72.73	0–0	0.00–0.00
	Apr 12 to Apr 18, 2021	17	0–6	0.00–54.55	0–0	0.00–0.00
	Apr 19 to Apr 25, 2021	8	0–0	0.00–0.00	0–3	0.00–27.27
	Apr 26 to May 2, 2021	11	0–0	0.00–0.00	0–0	0.00–0.00
	May 3 to May 9, 2021	20	3–9	27.27–81.82	0–0	0.00–0.00
	May 10 to May 16, 2021	13	0–3	0.00–30.00	0–0	0.00–0.00
	May 17 to May 23, 2021	15	0–5	0.00–50.00	0–0	0.00–0.00
	May 24 to May 30, 2021	14	0–4	0.00–40.00	0–0	0.00–0.00
Yamagata						
	Apr 5 to Apr 11, 2021	10	0–0	0.00–0.00	0–2	0.00–16.67
	Apr 12 to Apr 18, 2021	21	2–9	16.67–75.00	0–0	0.00–0.00
	Apr 19 to Apr 25, 2021	11	0–0	0.00–0.00	0–0	0.00–0.00
	Apr 26 to May 2, 2021	15	0–4	0.00–36.36	0–0	0.00–0.00
	May 3 to May 9, 2021	11	0–0	0.00–0.00	0–1	0.00–8.33
	May 10 to May 16, 2021	6	0–0	0.00–0.00	0–5	0.00–45.45
	May 17 to May 23, 2021	18	0–7	0.00–63.64	0–0	0.00–0.00
	May 24 to May 30, 2021	11	0–0	0.00–0.00	0–1	0.00–8.33
Fukushima						
	Apr 5 to Apr 11, 2021	13	0–0	0.00–0.00	0–3	0.00–18.75
	Apr 12 to Apr 18, 2021	14	0–0	0.00–0.00	0–1	0.00–6.67
	Apr 19 to Apr 25, 2021	11	0–0	0.00–0.00	0–5	0.00–31.25
	Apr 26 to May 2, 2021	19	0–3	0.00–18.75	0–0	0.00–0.00
	May 3 to May 9, 2021	20	0–4	0.00–25.00	0–0	0.00–0.00
	May 10 to May 16, 2021	16	0–0	0.00–0.00	0–0	0.00–0.00
	May 17 to May 23, 2021	15	0–0	0.00–0.00	0–0	0.00–0.00
	May 24 to May 30, 2021	13	0–0	0.00–0.00	0–2	0.00–13.33
Ibaraki						
	Apr 5 to Apr 11, 2021	14	0–0	0.00–0.00	0–1	0.00–6.67
	Apr 12 to Apr 18, 2021	20	0–5	0.00–33.33	0–0	0.00–0.00
	Apr 19 to Apr 25, 2021	14	0–0	0.00–0.00	0–0	0.00–0.00
	Apr 26 to May 2, 2021	12	0–0	0.00–0.00	0–2	0.00–14.29
	May 3 to May 9, 2021	18	0–5	0.00–38.46	0–0	0.00–0.00
	May 10 to May 16, 2021	17	0–4	0.00–30.77	0–0	0.00–0.00
	May 17 to May 23, 2021	20	0–7	0.00–53.85	0–0	0.00–0.00
	May 24 to May 30, 2021	15	0–3	0.00–25.00	0–0	0.00–0.00
Tochigi						
	Apr 5 to Apr 11, 2021	10	0–0	0.00–0.00	0–6	0.00–37.50
	Apr 12 to Apr 18, 2021	17	0–2	0.00–13.33	0–0	0.00–0.00
	Apr 19 to Apr 25, 2021	17	0–2	0.00–13.33	0–0	0.00–0.00
	Apr 26 to May 2, 2021	21	0–6	0.00–40.00	0–0	0.00–0.00
	May 3 to May 9, 2021	11	0–0	0.00–0.00	0–3	0.00–21.43

	May 10 to May 16, 2021	12	0–0	0.00–0.00	0–2	0.00–14.29
	May 17 to May 23, 2021	30	9–17	69.23–130.77	0–0	0.00–0.00
	May 24 to May 30, 2021	16	0–3	0.00–23.08	0–0	0.00–0.00
Gunma						
	Apr 5 to Apr 11, 2021	19	0–0	0.00–0.00	0–0	0.00–0.00
	Apr 12 to Apr 18, 2021	22	0–4	0.00–22.22	0–0	0.00–0.00
	Apr 19 to Apr 25, 2021	21	0–3	0.00–16.67	0–0	0.00–0.00
	Apr 26 to May 2, 2021	18	0–1	0.00–5.88	0–0	0.00–0.00
	May 3 to May 9, 2021	24	0–7	0.00–41.18	0–0	0.00–0.00
	May 10 to May 16, 2021	22	0–5	0.00–29.41	0–0	0.00–0.00
	May 17 to May 23, 2021	18	0–1	0.00–5.88	0–0	0.00–0.00
	May 24 to May 30, 2021	21	0–5	0.00–31.25	0–0	0.00–0.00
Saitama						
	Apr 5 to Apr 11, 2021	33	0–0	0.00–0.00	0–3	0.00–8.33
	Apr 12 to Apr 18, 2021	34	0–0	0.00–0.00	0–2	0.00–5.56
	Apr 19 to Apr 25, 2021	44	0–9	0.00–25.71	0–0	0.00–0.00
	Apr 26 to May 2, 2021	39	0–4	0.00–11.43	0–0	0.00–0.00
	May 3 to May 9, 2021	46	0–12	0.00–35.29	0–0	0.00–0.00
	May 10 to May 16, 2021	32	0–0	0.00–0.00	0–1	0.00–3.03
	May 17 to May 23, 2021	34	0–1	0.00–3.03	0–0	0.00–0.00
	May 24 to May 30, 2021	33	0–1	0.00–3.13	0–0	0.00–0.00
Chiba						
	Apr 5 to Apr 11, 2021	24	0–0	0.00–0.00	0–2	0.00–7.69
	Apr 12 to Apr 18, 2021	33	0–7	0.00–26.92	0–0	0.00–0.00
	Apr 19 to Apr 25, 2021	40	4–14	15.38–53.85	0–0	0.00–0.00
	Apr 26 to May 2, 2021	34	0–7	0.00–25.93	0–0	0.00–0.00
	May 3 to May 9, 2021	31	0–5	0.00–19.23	0–0	0.00–0.00
	May 10 to May 16, 2021	29	0–4	0.00–16.00	0–0	0.00–0.00
	May 17 to May 23, 2021	29	0–4	0.00–16.00	0–0	0.00–0.00
	May 24 to May 30, 2021	27	0–3	0.00–12.50	0–0	0.00–0.00
Tokyo						
	Apr 5 to Apr 11, 2021	52	0–0	0.00–0.00	0–5	0.00–8.77
	Apr 12 to Apr 18, 2021	70	0–14	0.00–25.00	0–0	0.00–0.00
	Apr 19 to Apr 25, 2021	53	0–0	0.00–0.00	0–4	0.00–7.02
	Apr 26 to May 2, 2021	67	0–12	0.00–21.82	0–0	0.00–0.00
	May 3 to May 9, 2021	71	1–16	1.82–29.09	0–0	0.00–0.00
	May 10 to May 16, 2021	62	0–8	0.00–14.81	0–0	0.00–0.00
	May 17 to May 23, 2021	58	0–4	0.00–7.41	0–0	0.00–0.00
	May 24 to May 30, 2021	41	0–0	0.00–0.00	0–11	0.00–21.15
Kanagawa						
	Apr 5 to Apr 11, 2021	50	0–10	0.00–25.00	0–0	0.00–0.00
	Apr 12 to Apr 18, 2021	52	0–12	0.00–30.00	0–0	0.00–0.00
	Apr 19 to Apr 25, 2021	46	0–6	0.00–15.00	0–0	0.00–0.00
	Apr 26 to May 2, 2021	44	0–4	0.00–10.00	0–0	0.00–0.00
	May 3 to May 9, 2021	50	0–12	0.00–31.58	0–0	0.00–0.00
	May 10 to May 16, 2021	50	1–13	2.70–35.14	0–0	0.00–0.00

	May 17 to May 23, 2021	53	3–16	8.11–43.24	0–0	0.00–0.00
	May 24 to May 30, 2021	42	0–5	0.00–13.51	0–0	0.00–0.00
Niigata						
	Apr 5 to Apr 11, 2021	29	0–6	0.00–26.09	0–0	0.00–0.00
	Apr 12 to Apr 18, 2021	22	0–0	0.00–0.00	0–1	0.00–4.35
	Apr 19 to Apr 25, 2021	29	0–5	0.00–20.83	0–0	0.00–0.00
	Apr 26 to May 2, 2021	22	0–0	0.00–0.00	0–3	0.00–12.00
	May 3 to May 9, 2021	17	0–0	0.00–0.00	0–7	0.00–29.17
	May 10 to May 16, 2021	30	0–7	0.00–30.43	0–0	0.00–0.00
	May 17 to May 23, 2021	24	0–1	0.00–4.35	0–0	0.00–0.00
	May 24 to May 30, 2021	22	0–0	0.00–0.00	0–1	0.00–4.35
Toyama						
	Apr 5 to Apr 11, 2021	10	0–1	0.00–11.11	0–0	0.00–0.00
	Apr 12 to Apr 18, 2021	13	0–4	0.00–44.44	0–0	0.00–0.00
	Apr 19 to Apr 25, 2021	7	0–0	0.00–0.00	0–2	0.00–22.22
	Apr 26 to May 2, 2021	13	0–4	0.00–44.44	0–0	0.00–0.00
	May 3 to May 9, 2021	12	0–3	0.00–33.33	0–0	0.00–0.00
	May 10 to May 16, 2021	7	0–0	0.00–0.00	0–2	0.00–22.22
	May 17 to May 23, 2021	7	0–0	0.00–0.00	0–3	0.00–30.00
	May 24 to May 30, 2021	10	0–1	0.00–11.11	0–0	0.00–0.00
Ishikawa						
	Apr 5 to Apr 11, 2021	13	0–3	0.00–30.00	0–0	0.00–0.00
	Apr 12 to Apr 18, 2021	14	0–4	0.00–40.00	0–0	0.00–0.00
	Apr 19 to Apr 25, 2021	10	0–0	0.00–0.00	0–0	0.00–0.00
	Apr 26 to May 2, 2021	15	0–5	0.00–50.00	0–0	0.00–0.00
	May 3 to May 9, 2021	9	0–0	0.00–0.00	0–1	0.00–10.00
	May 10 to May 16, 2021	11	0–1	0.00–10.00	0–0	0.00–0.00
	May 17 to May 23, 2021	16	0–6	0.00–60.00	0–0	0.00–0.00
	May 24 to May 30, 2021	13	0–3	0.00–30.00	0–0	0.00–0.00
Fukui						
	Apr 5 to Apr 11, 2021	6	0–0	0.00–0.00	0–2	0.00–25.00
	Apr 12 to Apr 18, 2021	3	0–0	0.00–0.00	0–5	0.00–62.50
	Apr 19 to Apr 25, 2021	6	0–0	0.00–0.00	0–2	0.00–25.00
	Apr 26 to May 2, 2021	10	0–2	0.00–25.00	0–0	0.00–0.00
	May 3 to May 9, 2021	13	0–5	0.00–62.50	0–0	0.00–0.00
	May 10 to May 16, 2021	13	0–5	0.00–62.50	0–0	0.00–0.00
	May 17 to May 23, 2021	12	0–4	0.00–50.00	0–0	0.00–0.00
	May 24 to May 30, 2021	8	0–1	0.00–14.29	0–0	0.00–0.00
Yamanashi						
	Apr 5 to Apr 11, 2021	8	0–2	0.00–33.33	0–0	0.00–0.00
	Apr 12 to Apr 18, 2021	10	0–4	0.00–66.67	0–0	0.00–0.00
	Apr 19 to Apr 25, 2021	4	0–0	0.00–0.00	0–2	0.00–33.33
	Apr 26 to May 2, 2021	9	0–2	0.00–28.57	0–0	0.00–0.00
	May 3 to May 9, 2021	11	0–4	0.00–57.14	0–0	0.00–0.00
	May 10 to May 16, 2021	12	0–6	0.00–100.00	0–0	0.00–0.00
	May 17 to May 23, 2021	3	0–0	0.00–0.00	0–4	0.00–57.14

	May 24 to May 30, 2021	9	0–2	0.00–28.57	0–0	0.00–0.00
Nagano						
	Apr 5 to Apr 11, 2021	23	0–0	0.00–0.00	0–1	0.00–4.17
	Apr 12 to Apr 18, 2021	25	0–1	0.00–4.17	0–0	0.00–0.00
	Apr 19 to Apr 25, 2021	25	0–0	0.00–0.00	0–0	0.00–0.00
	Apr 26 to May 2, 2021	23	0–0	0.00–0.00	0–2	0.00–8.00
	May 3 to May 9, 2021	20	0–0	0.00–0.00	0–4	0.00–16.67
	May 10 to May 16, 2021	31	0–7	0.00–29.17	0–0	0.00–0.00
	May 17 to May 23, 2021	22	0–0	0.00–0.00	0–2	0.00–8.33
	May 24 to May 30, 2021	22	0–0	0.00–0.00	0–1	0.00–4.35
Gifu						
	Apr 5 to Apr 11, 2021	22	0–6	0.00–37.50	0–0	0.00–0.00
	Apr 12 to Apr 18, 2021	20	0–4	0.00–25.00	0–0	0.00–0.00
	Apr 19 to Apr 25, 2021	21	0–5	0.00–31.25	0–0	0.00–0.00
	Apr 26 to May 2, 2021	19	0–4	0.00–26.67	0–0	0.00–0.00
	May 3 to May 9, 2021	21	0–6	0.00–40.00	0–0	0.00–0.00
	May 10 to May 16, 2021	19	0–4	0.00–26.67	0–0	0.00–0.00
	May 17 to May 23, 2021	17	0–3	0.00–21.43	0–0	0.00–0.00
	May 24 to May 30, 2021	16	0–2	0.00–14.29	0–0	0.00–0.00
Shizuoka						
	Apr 5 to Apr 11, 2021	30	0–0	0.00–0.00	0–6	0.00–16.67
	Apr 12 to Apr 18, 2021	47	0–13	0.00–38.24	0–0	0.00–0.00
	Apr 19 to Apr 25, 2021	41	0–7	0.00–20.59	0–0	0.00–0.00
	Apr 26 to May 2, 2021	39	0–6	0.00–18.18	0–0	0.00–0.00
	May 3 to May 9, 2021	45	2–14	6.45–45.16	0–0	0.00–0.00
	May 10 to May 16, 2021	40	0–8	0.00–25.00	0–0	0.00–0.00
	May 17 to May 23, 2021	43	1–13	3.33–43.33	0–0	0.00–0.00
	May 24 to May 30, 2021	25	0–0	0.00–0.00	0–4	0.00–13.79
Aichi						
	Apr 5 to Apr 11, 2021	36	0–0	0.00–0.00	0–4	0.00–10.00
	Apr 12 to Apr 18, 2021	29	0–0	0.00–0.00	0–11	0.00–27.50
	Apr 19 to Apr 25, 2021	38	0–0	0.00–0.00	0–1	0.00–2.56
	Apr 26 to May 2, 2021	38	0–0	0.00–0.00	0–1	0.00–2.56
	May 3 to May 9, 2021	38	0–0	0.00–0.00	0–0	0.00–0.00
	May 10 to May 16, 2021	41	0–4	0.00–10.81	0–0	0.00–0.00
	May 17 to May 23, 2021	34	0–0	0.00–0.00	0–2	0.00–5.56
	May 24 to May 30, 2021	38	0–1	0.00–2.70	0–0	0.00–0.00
Mie						
	Apr 5 to Apr 11, 2021	13	0–0	0.00–0.00	0–5	0.00–27.78
	Apr 12 to Apr 18, 2021	22	0–4	0.00–22.22	0–0	0.00–0.00
	Apr 19 to Apr 25, 2021	16	0–0	0.00–0.00	0–2	0.00–11.11
	Apr 26 to May 2, 2021	24	0–7	0.00–41.18	0–0	0.00–0.00
	May 3 to May 9, 2021	8	0–0	0.00–0.00	1–8	6.25–50.00
	May 10 to May 16, 2021	18	0–2	0.00–12.50	0–0	0.00–0.00
	May 17 to May 23, 2021	17	0–1	0.00–6.25	0–0	0.00–0.00
	May 24 to May 30, 2021	11	0–0	0.00–0.00	0–6	0.00–35.29

Shiga						
	Apr 5 to Apr 11, 2021	9	0–3	0.00–50.00	0–0	0.00–0.00
	Apr 12 to Apr 18, 2021	13	2–7	33.33–116.67	0–0	0.00–0.00
	Apr 19 to Apr 25, 2021	7	0–1	0.00–16.67	0–0	0.00–0.00
	Apr 26 to May 2, 2021	7	0–1	0.00–16.67	0–0	0.00–0.00
	May 3 to May 9, 2021	6	0–0	0.00–0.00	0–0	0.00–0.00
	May 10 to May 16, 2021	11	0–5	0.00–83.33	0–0	0.00–0.00
	May 17 to May 23, 2021	11	0–4	0.00–57.14	0–0	0.00–0.00
	May 24 to May 30, 2021	6	0–0	0.00–0.00	0–1	0.00–14.29
Kyoto						
	Apr 5 to Apr 11, 2021	17	0–1	0.00–6.25	0–0	0.00–0.00
	Apr 12 to Apr 18, 2021	14	0–0	0.00–0.00	0–2	0.00–12.50
	Apr 19 to Apr 25, 2021	23	0–7	0.00–43.75	0–0	0.00–0.00
	Apr 26 to May 2, 2021	17	0–1	0.00–6.25	0–0	0.00–0.00
	May 3 to May 9, 2021	16	0–0	0.00–0.00	0–0	0.00–0.00
	May 10 to May 16, 2021	19	0–4	0.00–26.67	0–0	0.00–0.00
	May 17 to May 23, 2021	12	0–0	0.00–0.00	0–2	0.00–14.29
	May 24 to May 30, 2021	17	0–3	0.00–21.43	0–0	0.00–0.00
Osaka						
	Apr 5 to Apr 11, 2021	55	0–14	0.00–34.15	0–0	0.00–0.00
	Apr 12 to Apr 18, 2021	47	0–7	0.00–17.50	0–0	0.00–0.00
	Apr 19 to Apr 25, 2021	37	0–0	0.00–0.00	0–3	0.00–7.50
	Apr 26 to May 2, 2021	53	0–12	0.00–29.27	0–0	0.00–0.00
	May 3 to May 9, 2021	46	0–6	0.00–15.00	0–0	0.00–0.00
	May 10 to May 16, 2021	39	0–0	0.00–0.00	0–1	0.00–2.50
	May 17 to May 23, 2021	37	0–0	0.00–0.00	0–1	0.00–2.63
	May 24 to May 30, 2021	40	0–3	0.00–8.11	0–0	0.00–0.00
Hyogo						
	Apr 5 to Apr 11, 2021	36	0–1	0.00–2.86	0–0	0.00–0.00
	Apr 12 to Apr 18, 2021	28	0–0	0.00–0.00	0–6	0.00–17.65
	Apr 19 to Apr 25, 2021	30	0–0	0.00–0.00	0–4	0.00–11.76
	Apr 26 to May 2, 2021	38	0–4	0.00–11.76	0–0	0.00–0.00
	May 3 to May 9, 2021	34	0–1	0.00–3.03	0–0	0.00–0.00
	May 10 to May 16, 2021	37	0–4	0.00–12.12	0–0	0.00–0.00
	May 17 to May 23, 2021	32	0–0	0.00–0.00	0–1	0.00–3.03
	May 24 to May 30, 2021	35	0–4	0.00–12.90	0–0	0.00–0.00
Nara						
	Apr 5 to Apr 11, 2021	8	0–0	0.00–0.00	0–2	0.00–20.00
	Apr 12 to Apr 18, 2021	8	0–0	0.00–0.00	0–2	0.00–20.00
	Apr 19 to Apr 25, 2021	12	0–1	0.00–9.09	0–0	0.00–0.00
	Apr 26 to May 2, 2021	16	0–5	0.00–45.45	0–0	0.00–0.00
	May 3 to May 9, 2021	8	0–0	0.00–0.00	0–3	0.00–27.27
	May 10 to May 16, 2021	16	0–4	0.00–33.33	0–0	0.00–0.00
	May 17 to May 23, 2021	12	0–1	0.00–9.09	0–0	0.00–0.00
	May 24 to May 30, 2021	9	0–0	0.00–0.00	0–2	0.00–18.18
Wakayama						

	Apr 5 to Apr 11, 2021	7	0–0	0.00–0.00	0–3	0.00–30.00
	Apr 12 to Apr 18, 2021	5	0–0	0.00–0.00	0–5	0.00–50.00
	Apr 19 to Apr 25, 2021	8	0–0	0.00–0.00	0–2	0.00–20.00
	Apr 26 to May 2, 2021	14	0–5	0.00–55.56	0–0	0.00–0.00
	May 3 to May 9, 2021	15	0–6	0.00–66.67	0–0	0.00–0.00
	May 10 to May 16, 2021	9	0–1	0.00–12.50	0–0	0.00–0.00
	May 17 to May 23, 2021	5	0–0	0.00–0.00	0–3	0.00–37.50
	May 24 to May 30, 2021	9	0–2	0.00–28.57	0–0	0.00–0.00
Tottori						
	Apr 5 to Apr 11, 2021	13	0–5	0.00–62.50	0–0	0.00–0.00
	Apr 12 to Apr 18, 2021	10	0–3	0.00–42.86	0–0	0.00–0.00
	Apr 19 to Apr 25, 2021	13	0–6	0.00–85.71	0–0	0.00–0.00
	Apr 26 to May 2, 2021	7	0–0	0.00–0.00	0–0	0.00–0.00
	May 3 to May 9, 2021	9	0–2	0.00–28.57	0–0	0.00–0.00
	May 10 to May 16, 2021	11	0–5	0.00–83.33	0–0	0.00–0.00
	May 17 to May 23, 2021	4	0–0	0.00–0.00	0–2	0.00–33.33
	May 24 to May 30, 2021	8	0–2	0.00–33.33	0–0	0.00–0.00
Shimane						
	Apr 5 to Apr 11, 2021	6	0–0	0.00–0.00	0–4	0.00–40.00
	Apr 12 to Apr 18, 2021	7	0–0	0.00–0.00	0–3	0.00–30.00
	Apr 19 to Apr 25, 2021	8	0–0	0.00–0.00	0–2	0.00–20.00
	Apr 26 to May 2, 2021	11	0–2	0.00–22.22	0–0	0.00–0.00
	May 3 to May 9, 2021	12	0–3	0.00–33.33	0–0	0.00–0.00
	May 10 to May 16, 2021	13	0–4	0.00–44.44	0–0	0.00–0.00
	May 17 to May 23, 2021	13	0–4	0.00–44.44	0–0	0.00–0.00
	May 24 to May 30, 2021	9	0–0	0.00–0.00	0–0	0.00–0.00
Okayama						
	Apr 5 to Apr 11, 2021	10	0–0	0.00–0.00	0–3	0.00–23.08
	Apr 12 to Apr 18, 2021	8	0–0	0.00–0.00	0–5	0.00–38.46
	Apr 19 to Apr 25, 2021	17	0–4	0.00–30.77	0–0	0.00–0.00
	Apr 26 to May 2, 2021	16	0–3	0.00–23.08	0–0	0.00–0.00
	May 3 to May 9, 2021	13	0–1	0.00–8.33	0–0	0.00–0.00
	May 10 to May 16, 2021	11	0–0	0.00–0.00	0–2	0.00–15.38
	May 17 to May 23, 2021	20	0–7	0.00–53.85	0–0	0.00–0.00
	May 24 to May 30, 2021	11	0–0	0.00–0.00	0–2	0.00–15.38
Hiroshima						
	Apr 5 to Apr 11, 2021	20	0–1	0.00–5.26	0–0	0.00–0.00
	Apr 12 to Apr 18, 2021	19	0–0	0.00–0.00	0–0	0.00–0.00
	Apr 19 to Apr 25, 2021	19	0–0	0.00–0.00	0–0	0.00–0.00
	Apr 26 to May 2, 2021	12	0–0	0.00–0.00	0–7	0.00–36.84
	May 3 to May 9, 2021	28	1–10	5.56–55.56	0–0	0.00–0.00
	May 10 to May 16, 2021	23	0–5	0.00–27.78	0–0	0.00–0.00
	May 17 to May 23, 2021	22	0–4	0.00–22.22	0–0	0.00–0.00
	May 24 to May 30, 2021	21	0–3	0.00–16.67	0–0	0.00–0.00
Yamaguchi						
	Apr 5 to Apr 11, 2021	13	0–0	0.00–0.00	0–0	0.00–0.00

	Apr 12 to Apr 18, 2021	14	0–2	0.00–16.67	0–0	0.00–0.00
	Apr 19 to Apr 25, 2021	13	0–1	0.00–8.33	0–0	0.00–0.00
	Apr 26 to May 2, 2021	17	0–5	0.00–41.67	0–0	0.00–0.00
	May 3 to May 9, 2021	10	0–0	0.00–0.00	0–3	0.00–23.08
	May 10 to May 16, 2021	12	0–0	0.00–0.00	0–0	0.00–0.00
	May 17 to May 23, 2021	9	0–0	0.00–0.00	0–3	0.00–25.00
	May 24 to May 30, 2021	9	0–0	0.00–0.00	0–2	0.00–18.18
Tokushima						
	Apr 5 to Apr 11, 2021	6	0–0	0.00–0.00	0–1	0.00–14.29
	Apr 12 to Apr 18, 2021	5	0–0	0.00–0.00	0–2	0.00–28.57
	Apr 19 to Apr 25, 2021	8	0–1	0.00–14.29	0–0	0.00–0.00
	Apr 26 to May 2, 2021	10	0–3	0.00–42.86	0–0	0.00–0.00
	May 3 to May 9, 2021	12	0–6	0.00–100.00	0–0	0.00–0.00
	May 10 to May 16, 2021	9	0–4	0.00–80.00	0–0	0.00–0.00
	May 17 to May 23, 2021	12	2–7	40.00–140.00	0–0	0.00–0.00
	May 24 to May 30, 2021	5	0–0	0.00–0.00	0–0	0.00–0.00
Kagawa						
	Apr 5 to Apr 11, 2021	8	0–0	0.00–0.00	0–2	0.00–20.00
	Apr 12 to Apr 18, 2021	8	0–0	0.00–0.00	0–2	0.00–20.00
	Apr 19 to Apr 25, 2021	11	0–1	0.00–10.00	0–0	0.00–0.00
	Apr 26 to May 2, 2021	14	0–4	0.00–40.00	0–0	0.00–0.00
	May 3 to May 9, 2021	16	0–6	0.00–60.00	0–0	0.00–0.00
	May 10 to May 16, 2021	10	0–1	0.00–11.11	0–0	0.00–0.00
	May 17 to May 23, 2021	7	0–0	0.00–0.00	0–2	0.00–22.22
	May 24 to May 30, 2021	7	0–0	0.00–0.00	0–2	0.00–22.22
Ehime						
	Apr 5 to Apr 11, 2021	8	0–0	0.00–0.00	0–5	0.00–38.46
	Apr 12 to Apr 18, 2021	13	0–0	0.00–0.00	0–0	0.00–0.00
	Apr 19 to Apr 25, 2021	13	0–0	0.00–0.00	0–0	0.00–0.00
	Apr 26 to May 2, 2021	15	0–3	0.00–25.00	0–0	0.00–0.00
	May 3 to May 9, 2021	17	0–5	0.00–41.67	0–0	0.00–0.00
	May 10 to May 16, 2021	13	0–1	0.00–8.33	0–0	0.00–0.00
	May 17 to May 23, 2021	13	0–1	0.00–8.33	0–0	0.00–0.00
	May 24 to May 30, 2021	13	0–1	0.00–8.33	0–0	0.00–0.00
Kochi						
	Apr 5 to Apr 11, 2021	7	0–2	0.00–40.00	0–0	0.00–0.00
	Apr 12 to Apr 18, 2021	4	0–0	0.00–0.00	0–1	0.00–20.00
	Apr 19 to Apr 25, 2021	3	0–0	0.00–0.00	0–2	0.00–40.00
	Apr 26 to May 2, 2021	11	1–6	20.00–120.00	0–0	0.00–0.00
	May 3 to May 9, 2021	9	0–4	0.00–80.00	0–0	0.00–0.00
	May 10 to May 16, 2021	7	0–2	0.00–40.00	0–0	0.00–0.00
	May 17 to May 23, 2021	3	0–0	0.00–0.00	0–2	0.00–40.00
	May 24 to May 30, 2021	6	0–2	0.00–50.00	0–0	0.00–0.00
Fukuoka						
	Apr 5 to Apr 11, 2021	24	0–0	0.00–0.00	0–3	0.00–11.11
	Apr 12 to Apr 18, 2021	19	0–0	0.00–0.00	0–9	0.00–32.14

	Apr 19 to Apr 25, 2021	21	0–0	0.00–0.00	0–7	0.00–25.00
	Apr 26 to May 2, 2021	32	0–5	0.00–18.52	0–0	0.00–0.00
	May 3 to May 9, 2021	35	0–9	0.00–34.62	0–0	0.00–0.00
	May 10 to May 16, 2021	29	0–4	0.00–16.00	0–0	0.00–0.00
	May 17 to May 23, 2021	21	0–0	0.00–0.00	0–4	0.00–16.00
	May 24 to May 30, 2021	24	0–0	0.00–0.00	0–0	0.00–0.00
Saga						
	Apr 5 to Apr 11, 2021	5	0–0	0.00–0.00	0–2	0.00–28.57
	Apr 12 to Apr 18, 2021	5	0–0	0.00–0.00	0–2	0.00–28.57
	Apr 19 to Apr 25, 2021	9	0–3	0.00–50.00	0–0	0.00–0.00
	Apr 26 to May 2, 2021	2	0–0	0.00–0.00	0–4	0.00–66.67
	May 3 to May 9, 2021	5	0–0	0.00–0.00	0–1	0.00–16.67
	May 10 to May 16, 2021	6	0–0	0.00–0.00	0–0	0.00–0.00
	May 17 to May 23, 2021	3	0–0	0.00–0.00	0–3	0.00–50.00
	May 24 to May 30, 2021	4	0–0	0.00–0.00	0–1	0.00–20.00
Nagasaki						
	Apr 5 to Apr 11, 2021	12	0–2	0.00–20.00	0–0	0.00–0.00
	Apr 12 to Apr 18, 2021	6	0–0	0.00–0.00	0–4	0.00–40.00
	Apr 19 to Apr 25, 2021	12	0–2	0.00–20.00	0–0	0.00–0.00
	Apr 26 to May 2, 2021	15	0–5	0.00–50.00	0–0	0.00–0.00
	May 3 to May 9, 2021	17	0–7	0.00–70.00	0–0	0.00–0.00
	May 10 to May 16, 2021	16	0–6	0.00–60.00	0–0	0.00–0.00
	May 17 to May 23, 2021	4	0–0	0.00–0.00	0–6	0.00–60.00
	May 24 to May 30, 2021	14	0–4	0.00–40.00	0–0	0.00–0.00
Kumamoto						
	Apr 5 to Apr 11, 2021	12	0–0	0.00–0.00	0–0	0.00–0.00
	Apr 12 to Apr 18, 2021	15	0–3	0.00–25.00	0–0	0.00–0.00
	Apr 19 to Apr 25, 2021	11	0–0	0.00–0.00	0–1	0.00–8.33
	Apr 26 to May 2, 2021	24	4–11	30.77–84.62	0–0	0.00–0.00
	May 3 to May 9, 2021	16	0–4	0.00–33.33	0–0	0.00–0.00
	May 10 to May 16, 2021	14	0–2	0.00–16.67	0–0	0.00–0.00
	May 17 to May 23, 2021	13	0–2	0.00–18.18	0–0	0.00–0.00
	May 24 to May 30, 2021	21	4–10	36.36–90.91	0–0	0.00–0.00
Oita						
	Apr 5 to Apr 11, 2021	9	0–0	0.00–0.00	0–6	0.00–40.00
	Apr 12 to Apr 18, 2021	22	0–8	0.00–57.14	0–0	0.00–0.00
	Apr 19 to Apr 25, 2021	21	0–7	0.00–50.00	0–0	0.00–0.00
	Apr 26 to May 2, 2021	15	0–2	0.00–15.38	0–0	0.00–0.00
	May 3 to May 9, 2021	14	0–0	0.00–0.00	0–0	0.00–0.00
	May 10 to May 16, 2021	16	0–2	0.00–14.29	0–0	0.00–0.00
	May 17 to May 23, 2021	12	0–0	0.00–0.00	0–2	0.00–14.29
	May 24 to May 30, 2021	14	0–1	0.00–7.69	0–0	0.00–0.00
Miyazaki						
	Apr 5 to Apr 11, 2021	7	0–0	0.00–0.00	0–3	0.00–30.00
	Apr 12 to Apr 18, 2021	10	0–0	0.00–0.00	0–1	0.00–9.09
	Apr 19 to Apr 25, 2021	8	0–0	0.00–0.00	0–3	0.00–27.27

	Apr 26 to May 2, 2021	10	0–0	0.00–0.00	0–0	0.00–0.00
	May 3 to May 9, 2021	11	0–0	0.00–0.00	0–0	0.00–0.00
	May 10 to May 16, 2021	13	0–3	0.00–30.00	0–0	0.00–0.00
	May 17 to May 23, 2021	15	0–5	0.00–50.00	0–0	0.00–0.00
	May 24 to May 30, 2021	12	0–2	0.00–20.00	0–0	0.00–0.00
Kagoshima						
	Apr 5 to Apr 11, 2021	16	0–1	0.00–6.67	0–0	0.00–0.00
	Apr 12 to Apr 18, 2021	14	0–0	0.00–0.00	0–1	0.00–6.67
	Apr 19 to Apr 25, 2021	19	0–5	0.00–35.71	0–0	0.00–0.00
	Apr 26 to May 2, 2021	17	0–4	0.00–30.77	0–0	0.00–0.00
	May 3 to May 9, 2021	12	0–0	0.00–0.00	0–1	0.00–7.69
	May 10 to May 16, 2021	10	0–0	0.00–0.00	0–2	0.00–16.67
	May 17 to May 23, 2021	12	0–0	0.00–0.00	0–1	0.00–7.69
	May 24 to May 30, 2021	13	0–1	0.00–8.33	0–0	0.00–0.00
Okinawa						
	Apr 5 to Apr 11, 2021	9	0–1	0.00–12.50	0–0	0.00–0.00
	Apr 12 to Apr 18, 2021	5	0–0	0.00–0.00	0–3	0.00–37.50
	Apr 19 to Apr 25, 2021	8	0–0	0.00–0.00	0–0	0.00–0.00
	Apr 26 to May 2, 2021	8	0–0	0.00–0.00	0–0	0.00–0.00
	May 3 to May 9, 2021	7	0–0	0.00–0.00	0–1	0.00–12.50
	May 10 to May 16, 2021	11	0–3	0.00–37.50	0–0	0.00–0.00
	May 17 to May 23, 2021	6	0–0	0.00–0.00	0–2	0.00–25.00
	May 24 to May 30, 2021	7	0–0	0.00–0.00	0–2	0.00–22.22

Table A.12: Weekly number of observed and excess/exiguous deaths in Japan and 47 prefectures for circulatory disease-related deaths at homes from January 2020 through May 2021.

Prefecture	Week	Observed	Excess deaths	Percent excess	Exiguous deaths	Percent exiguous
Japan						
	Apr 5 to Apr 11, 2021	1242	0–47	0.00–3.93	0–0	0.00–0.00
	Apr 12 to Apr 18, 2021	1304	0–142	0.00–12.22	0–0	0.00–0.00
	Apr 19 to Apr 25, 2021	1193	0–72	0.00–6.42	0–0	0.00–0.00
	Apr 26 to May 2, 2021	1321	22–248	2.05–23.11	0–0	0.00–0.00
	May 3 to May 9, 2021	1236	0–202	0.00–19.54	0–0	0.00–0.00
	May 10 to May 16, 2021	1220	0–218	0.00–21.76	0–0	0.00–0.00
	May 17 to May 23, 2021	1083	0–103	0.00–10.51	0–0	0.00–0.00
	May 24 to May 30, 2021	1060	0–111	0.00–11.70	0–0	0.00–0.00
Hokkaido						
	Apr 5 to Apr 11, 2021	42	0–0	0.00–0.00	0–4	0.00–8.70
	Apr 12 to Apr 18, 2021	44	0–0	0.00–0.00	0–1	0.00–2.22
	Apr 19 to Apr 25, 2021	33	0–0	0.00–0.00	0–12	0.00–26.67
	Apr 26 to May 2, 2021	48	0–4	0.00–9.09	0–0	0.00–0.00
	May 3 to May 9, 2021	58	0–15	0.00–34.88	0–0	0.00–0.00
	May 10 to May 16, 2021	49	0–7	0.00–16.67	0–0	0.00–0.00
	May 17 to May 23, 2021	55	0–11	0.00–25.00	0–0	0.00–0.00
	May 24 to May 30, 2021	42	0–0	0.00–0.00	0–0	0.00–0.00
Aomori						
	Apr 5 to Apr 11, 2021	14	0–2	0.00–16.67	0–0	0.00–0.00
	Apr 12 to Apr 18, 2021	15	0–4	0.00–36.36	0–0	0.00–0.00
	Apr 19 to Apr 25, 2021	8	0–0	0.00–0.00	0–3	0.00–27.27
	Apr 26 to May 2, 2021	7	0–0	0.00–0.00	0–4	0.00–36.36
	May 3 to May 9, 2021	6	0–0	0.00–0.00	0–5	0.00–45.45
	May 10 to May 16, 2021	10	0–0	0.00–0.00	0–1	0.00–9.09
	May 17 to May 23, 2021	13	0–2	0.00–18.18	0–0	0.00–0.00
	May 24 to May 30, 2021	6	0–0	0.00–0.00	0–4	0.00–40.00
Iwate						
	Apr 5 to Apr 11, 2021	17	0–0	0.00–0.00	0–2	0.00–10.53
	Apr 12 to Apr 18, 2021	14	0–0	0.00–0.00	0–4	0.00–22.22
	Apr 19 to Apr 25, 2021	11	0–0	0.00–0.00	0–6	0.00–35.29
	Apr 26 to May 2, 2021	23	0–7	0.00–43.75	0–0	0.00–0.00
	May 3 to May 9, 2021	15	0–0	0.00–0.00	0–0	0.00–0.00
	May 10 to May 16, 2021	19	0–5	0.00–35.71	0–0	0.00–0.00
	May 17 to May 23, 2021	15	0–1	0.00–7.14	0–0	0.00–0.00
	May 24 to May 30, 2021	13	0–0	0.00–0.00	0–1	0.00–7.14
Miyagi						
	Apr 5 to Apr 11, 2021	33	0–10	0.00–43.48	0–0	0.00–0.00
	Apr 12 to Apr 18, 2021	18	0–0	0.00–0.00	0–4	0.00–18.18
	Apr 19 to Apr 25, 2021	19	0–0	0.00–0.00	0–2	0.00–9.52
	Apr 26 to May 2, 2021	27	0–7	0.00–35.00	0–0	0.00–0.00
	May 3 to May 9, 2021	21	0–2	0.00–10.53	0–0	0.00–0.00

	May 10 to May 16, 2021	18	0–0	0.00–0.00	0–1	0.00–5.26
	May 17 to May 23, 2021	16	0–0	0.00–0.00	0–1	0.00–5.88
	May 24 to May 30, 2021	18	0–1	0.00–5.88	0–0	0.00–0.00
Akita						
	Apr 5 to Apr 11, 2021	7	0–0	0.00–0.00	0–3	0.00–30.00
	Apr 12 to Apr 18, 2021	7	0–0	0.00–0.00	0–3	0.00–30.00
	Apr 19 to Apr 25, 2021	7	0–0	0.00–0.00	0–2	0.00–22.22
	Apr 26 to May 2, 2021	7	0–0	0.00–0.00	0–1	0.00–12.50
	May 3 to May 9, 2021	9	0–1	0.00–12.50	0–0	0.00–0.00
	May 10 to May 16, 2021	10	0–2	0.00–25.00	0–0	0.00–0.00
	May 17 to May 23, 2021	5	0–0	0.00–0.00	0–3	0.00–37.50
	May 24 to May 30, 2021	7	0–0	0.00–0.00	0–1	0.00–12.50
Yamagata						
	Apr 5 to Apr 11, 2021	9	0–0	0.00–0.00	0–4	0.00–30.77
	Apr 12 to Apr 18, 2021	11	0–0	0.00–0.00	0–1	0.00–8.33
	Apr 19 to Apr 25, 2021	13	0–1	0.00–8.33	0–0	0.00–0.00
	Apr 26 to May 2, 2021	11	0–0	0.00–0.00	0–1	0.00–8.33
	May 3 to May 9, 2021	15	0–3	0.00–25.00	0–0	0.00–0.00
	May 10 to May 16, 2021	18	0–6	0.00–50.00	0–0	0.00–0.00
	May 17 to May 23, 2021	11	0–0	0.00–0.00	0–0	0.00–0.00
	May 24 to May 30, 2021	22	3–11	27.27–100.00	0–0	0.00–0.00
Fukushima						
	Apr 5 to Apr 11, 2021	22	0–0	0.00–0.00	0–2	0.00–8.33
	Apr 12 to Apr 18, 2021	27	0–4	0.00–17.39	0–0	0.00–0.00
	Apr 19 to Apr 25, 2021	24	0–3	0.00–14.29	0–0	0.00–0.00
	Apr 26 to May 2, 2021	25	0–4	0.00–19.05	0–0	0.00–0.00
	May 3 to May 9, 2021	21	0–1	0.00–5.00	0–0	0.00–0.00
	May 10 to May 16, 2021	22	0–2	0.00–10.00	0–0	0.00–0.00
	May 17 to May 23, 2021	21	0–2	0.00–10.53	0–0	0.00–0.00
	May 24 to May 30, 2021	19	0–1	0.00–5.56	0–0	0.00–0.00
Ibaraki						
	Apr 5 to Apr 11, 2021	38	0–9	0.00–31.03	0–0	0.00–0.00
	Apr 12 to Apr 18, 2021	32	0–4	0.00–14.29	0–0	0.00–0.00
	Apr 19 to Apr 25, 2021	25	0–0	0.00–0.00	0–2	0.00–7.41
	Apr 26 to May 2, 2021	22	0–0	0.00–0.00	0–5	0.00–18.52
	May 3 to May 9, 2021	25	0–0	0.00–0.00	0–0	0.00–0.00
	May 10 to May 16, 2021	38	4–15	17.39–65.22	0–0	0.00–0.00
	May 17 to May 23, 2021	19	0–0	0.00–0.00	0–3	0.00–13.64
	May 24 to May 30, 2021	24	0–2	0.00–9.09	0–0	0.00–0.00
Tochigi						
	Apr 5 to Apr 11, 2021	32	0–7	0.00–28.00	0–0	0.00–0.00
	Apr 12 to Apr 18, 2021	30	0–7	0.00–30.43	0–0	0.00–0.00
	Apr 19 to Apr 25, 2021	31	0–9	0.00–40.91	0–0	0.00–0.00
	Apr 26 to May 2, 2021	22	0–1	0.00–4.76	0–0	0.00–0.00
	May 3 to May 9, 2021	20	0–0	0.00–0.00	0–0	0.00–0.00
	May 10 to May 16, 2021	22	0–4	0.00–22.22	0–0	0.00–0.00

	May 17 to May 23, 2021	32	5–14	27.78–77.78	0–0	0.00–0.00
	May 24 to May 30, 2021	22	0–4	0.00–22.22	0–0	0.00–0.00
Gunma						
	Apr 5 to Apr 11, 2021	21	0–0	0.00–0.00	0–1	0.00–4.55
	Apr 12 to Apr 18, 2021	21	0–1	0.00–5.00	0–0	0.00–0.00
	Apr 19 to Apr 25, 2021	24	0–4	0.00–20.00	0–0	0.00–0.00
	Apr 26 to May 2, 2021	30	0–10	0.00–50.00	0–0	0.00–0.00
	May 3 to May 9, 2021	23	0–4	0.00–21.05	0–0	0.00–0.00
	May 10 to May 16, 2021	17	0–0	0.00–0.00	0–2	0.00–10.53
	May 17 to May 23, 2021	17	0–0	0.00–0.00	0–1	0.00–5.56
	May 24 to May 30, 2021	17	0–0	0.00–0.00	0–0	0.00–0.00
Saitama						
	Apr 5 to Apr 11, 2021	46	0–0	0.00–0.00	0–15	0.00–24.59
	Apr 12 to Apr 18, 2021	75	0–17	0.00–29.31	0–0	0.00–0.00
	Apr 19 to Apr 25, 2021	58	0–1	0.00–1.75	0–0	0.00–0.00
	Apr 26 to May 2, 2021	81	6–26	10.91–47.27	0–0	0.00–0.00
	May 3 to May 9, 2021	64	0–13	0.00–25.49	0–0	0.00–0.00
	May 10 to May 16, 2021	56	0–5	0.00–9.80	0–0	0.00–0.00
	May 17 to May 23, 2021	56	0–7	0.00–14.29	0–0	0.00–0.00
	May 24 to May 30, 2021	49	0–1	0.00–2.08	0–0	0.00–0.00
Chiba						
	Apr 5 to Apr 11, 2021	56	0–0	0.00–0.00	0–10	0.00–15.15
	Apr 12 to Apr 18, 2021	74	0–9	0.00–13.85	0–0	0.00–0.00
	Apr 19 to Apr 25, 2021	73	0–10	0.00–15.87	0–0	0.00–0.00
	Apr 26 to May 2, 2021	71	0–11	0.00–18.33	0–0	0.00–0.00
	May 3 to May 9, 2021	54	0–0	0.00–0.00	0–4	0.00–6.90
	May 10 to May 16, 2021	78	1–22	1.79–39.29	0–0	0.00–0.00
	May 17 to May 23, 2021	57	0–2	0.00–3.64	0–0	0.00–0.00
	May 24 to May 30, 2021	50	0–0	0.00–0.00	0–2	0.00–3.85
Tokyo						
	Apr 5 to Apr 11, 2021	127	0–0	0.00–0.00	0–8	0.00–5.93
	Apr 12 to Apr 18, 2021	153	0–23	0.00–17.69	0–0	0.00–0.00
	Apr 19 to Apr 25, 2021	156	0–32	0.00–25.81	0–0	0.00–0.00
	Apr 26 to May 2, 2021	139	0–20	0.00–16.81	0–0	0.00–0.00
	May 3 to May 9, 2021	121	0–8	0.00–7.08	0–0	0.00–0.00
	May 10 to May 16, 2021	105	0–0	0.00–0.00	0–4	0.00–3.67
	May 17 to May 23, 2021	105	0–0	0.00–0.00	0–1	0.00–0.94
	May 24 to May 30, 2021	97	0–0	0.00–0.00	0–6	0.00–5.83
Kanagawa						
	Apr 5 to Apr 11, 2021	85	0–0	0.00–0.00	0–1	0.00–1.16
	Apr 12 to Apr 18, 2021	75	0–0	0.00–0.00	0–8	0.00–9.64
	Apr 19 to Apr 25, 2021	76	0–0	0.00–0.00	0–7	0.00–8.43
	Apr 26 to May 2, 2021	110	5–32	6.41–41.03	0–0	0.00–0.00
	May 3 to May 9, 2021	81	0–4	0.00–5.19	0–0	0.00–0.00
	May 10 to May 16, 2021	77	0–0	0.00–0.00	0–0	0.00–0.00
	May 17 to May 23, 2021	67	0–0	0.00–0.00	0–8	0.00–10.67

	May 24 to May 30, 2021	59	0–0	0.00–0.00	0–13	0.00–18.06
Niigata						
	Apr 5 to Apr 11, 2021	25	0–6	0.00–31.58	0–0	0.00–0.00
	Apr 12 to Apr 18, 2021	29	2–10	10.53–52.63	0–0	0.00–0.00
	Apr 19 to Apr 25, 2021	11	0–0	0.00–0.00	0–7	0.00–38.89
	Apr 26 to May 2, 2021	19	0–1	0.00–5.56	0–0	0.00–0.00
	May 3 to May 9, 2021	15	0–0	0.00–0.00	0–2	0.00–11.76
	May 10 to May 16, 2021	19	0–3	0.00–18.75	0–0	0.00–0.00
	May 17 to May 23, 2021	15	0–0	0.00–0.00	0–0	0.00–0.00
	May 24 to May 30, 2021	15	0–0	0.00–0.00	0–0	0.00–0.00
Toyama						
	Apr 5 to Apr 11, 2021	10	0–3	0.00–42.86	0–0	0.00–0.00
	Apr 12 to Apr 18, 2021	4	0–0	0.00–0.00	0–2	0.00–33.33
	Apr 19 to Apr 25, 2021	7	0–1	0.00–16.67	0–0	0.00–0.00
	Apr 26 to May 2, 2021	5	0–0	0.00–0.00	0–1	0.00–16.67
	May 3 to May 9, 2021	13	2–7	33.33–116.67	0–0	0.00–0.00
	May 10 to May 16, 2021	4	0–0	0.00–0.00	0–2	0.00–33.33
	May 17 to May 23, 2021	5	0–0	0.00–0.00	0–1	0.00–16.67
	May 24 to May 30, 2021	13	3–8	60.00–160.00	0–0	0.00–0.00
Ishikawa						
	Apr 5 to Apr 11, 2021	15	1–7	12.50–87.50	0–0	0.00–0.00
	Apr 12 to Apr 18, 2021	12	0–4	0.00–50.00	0–0	0.00–0.00
	Apr 19 to Apr 25, 2021	8	0–0	0.00–0.00	0–0	0.00–0.00
	Apr 26 to May 2, 2021	8	0–1	0.00–14.29	0–0	0.00–0.00
	May 3 to May 9, 2021	13	0–6	0.00–85.71	0–0	0.00–0.00
	May 10 to May 16, 2021	7	0–0	0.00–0.00	0–1	0.00–12.50
	May 17 to May 23, 2021	9	0–1	0.00–12.50	0–0	0.00–0.00
	May 24 to May 30, 2021	9	0–2	0.00–28.57	0–0	0.00–0.00
Fukui						
	Apr 5 to Apr 11, 2021	11	0–0	0.00–0.00	0–0	0.00–0.00
	Apr 12 to Apr 18, 2021	7	0–0	0.00–0.00	0–3	0.00–30.00
	Apr 19 to Apr 25, 2021	6	0–0	0.00–0.00	0–4	0.00–40.00
	Apr 26 to May 2, 2021	10	0–1	0.00–11.11	0–0	0.00–0.00
	May 3 to May 9, 2021	17	0–8	0.00–88.89	0–0	0.00–0.00
	May 10 to May 16, 2021	12	0–4	0.00–50.00	0–0	0.00–0.00
	May 17 to May 23, 2021	6	0–0	0.00–0.00	0–2	0.00–25.00
	May 24 to May 30, 2021	6	0–0	0.00–0.00	0–2	0.00–25.00
Yamanashi						
	Apr 5 to Apr 11, 2021	7	0–0	0.00–0.00	0–0	0.00–0.00
	Apr 12 to Apr 18, 2021	9	0–2	0.00–28.57	0–0	0.00–0.00
	Apr 19 to Apr 25, 2021	4	0–0	0.00–0.00	0–2	0.00–33.33
	Apr 26 to May 2, 2021	10	0–4	0.00–66.67	0–0	0.00–0.00
	May 3 to May 9, 2021	9	0–3	0.00–50.00	0–0	0.00–0.00
	May 10 to May 16, 2021	8	0–2	0.00–33.33	0–0	0.00–0.00
	May 17 to May 23, 2021	7	0–1	0.00–16.67	0–0	0.00–0.00
	May 24 to May 30, 2021	9	0–3	0.00–50.00	0–0	0.00–0.00

Nagano						
	Apr 5 to Apr 11, 2021	24	0–5	0.00–26.32	0–0	0.00–0.00
	Apr 12 to Apr 18, 2021	21	0–2	0.00–10.53	0–0	0.00–0.00
	Apr 19 to Apr 25, 2021	25	0–6	0.00–31.58	0–0	0.00–0.00
	Apr 26 to May 2, 2021	16	0–0	0.00–0.00	0–2	0.00–11.11
	May 3 to May 9, 2021	16	0–0	0.00–0.00	0–2	0.00–11.11
	May 10 to May 16, 2021	13	0–0	0.00–0.00	0–5	0.00–27.78
	May 17 to May 23, 2021	20	0–3	0.00–17.65	0–0	0.00–0.00
	May 24 to May 30, 2021	14	0–0	0.00–0.00	0–3	0.00–17.65
Gifu						
	Apr 5 to Apr 11, 2021	12	0–0	0.00–0.00	0–4	0.00–25.00
	Apr 12 to Apr 18, 2021	17	0–2	0.00–13.33	0–0	0.00–0.00
	Apr 19 to Apr 25, 2021	15	0–0	0.00–0.00	0–0	0.00–0.00
	Apr 26 to May 2, 2021	8	0–0	0.00–0.00	0–6	0.00–42.86
	May 3 to May 9, 2021	9	0–0	0.00–0.00	0–5	0.00–35.71
	May 10 to May 16, 2021	13	0–0	0.00–0.00	0–0	0.00–0.00
	May 17 to May 23, 2021	14	0–2	0.00–16.67	0–0	0.00–0.00
	May 24 to May 30, 2021	17	0–5	0.00–41.67	0–0	0.00–0.00
Shizuoka						
	Apr 5 to Apr 11, 2021	51	0–13	0.00–34.21	0–0	0.00–0.00
	Apr 12 to Apr 18, 2021	25	0–0	0.00–0.00	0–12	0.00–32.43
	Apr 19 to Apr 25, 2021	36	0–0	0.00–0.00	0–0	0.00–0.00
	Apr 26 to May 2, 2021	37	0–2	0.00–5.71	0–0	0.00–0.00
	May 3 to May 9, 2021	36	0–2	0.00–5.88	0–0	0.00–0.00
	May 10 to May 16, 2021	32	0–0	0.00–0.00	0–1	0.00–3.03
	May 17 to May 23, 2021	27	0–0	0.00–0.00	0–5	0.00–15.63
	May 24 to May 30, 2021	36	0–5	0.00–16.13	0–0	0.00–0.00
Aichi						
	Apr 5 to Apr 11, 2021	55	0–5	0.00–10.00	0–0	0.00–0.00
	Apr 12 to Apr 18, 2021	54	0–5	0.00–10.20	0–0	0.00–0.00
	Apr 19 to Apr 25, 2021	45	0–0	0.00–0.00	0–1	0.00–2.17
	Apr 26 to May 2, 2021	47	0–1	0.00–2.17	0–0	0.00–0.00
	May 3 to May 9, 2021	44	0–0	0.00–0.00	0–0	0.00–0.00
	May 10 to May 16, 2021	62	2–19	4.65–44.19	0–0	0.00–0.00
	May 17 to May 23, 2021	34	0–0	0.00–0.00	0–7	0.00–17.07
	May 24 to May 30, 2021	45	0–5	0.00–12.50	0–0	0.00–0.00
Mie						
	Apr 5 to Apr 11, 2021	17	0–0	0.00–0.00	0–2	0.00–10.53
	Apr 12 to Apr 18, 2021	24	0–7	0.00–41.18	0–0	0.00–0.00
	Apr 19 to Apr 25, 2021	20	0–3	0.00–17.65	0–0	0.00–0.00
	Apr 26 to May 2, 2021	17	0–1	0.00–6.25	0–0	0.00–0.00
	May 3 to May 9, 2021	19	0–3	0.00–18.75	0–0	0.00–0.00
	May 10 to May 16, 2021	12	0–0	0.00–0.00	0–3	0.00–20.00
	May 17 to May 23, 2021	16	0–1	0.00–6.67	0–0	0.00–0.00
	May 24 to May 30, 2021	11	0–0	0.00–0.00	0–3	0.00–21.43
Shiga						

	Apr 5 to Apr 11, 2021	9	0–0	0.00–0.00	0–5	0.00–35.71
	Apr 12 to Apr 18, 2021	12	0–0	0.00–0.00	0–1	0.00–7.69
	Apr 19 to Apr 25, 2021	10	0–0	0.00–0.00	0–2	0.00–16.67
	Apr 26 to May 2, 2021	15	0–3	0.00–25.00	0–0	0.00–0.00
	May 3 to May 9, 2021	17	0–7	0.00–70.00	0–0	0.00–0.00
	May 10 to May 16, 2021	11	0–0	0.00–0.00	0–0	0.00–0.00
	May 17 to May 23, 2021	16	0–6	0.00–60.00	0–0	0.00–0.00
	May 24 to May 30, 2021	14	0–4	0.00–40.00	0–0	0.00–0.00
Kyoto						
	Apr 5 to Apr 11, 2021	27	0–0	0.00–0.00	0–3	0.00–10.00
	Apr 12 to Apr 18, 2021	37	0–7	0.00–23.33	0–0	0.00–0.00
	Apr 19 to Apr 25, 2021	31	0–2	0.00–6.90	0–0	0.00–0.00
	Apr 26 to May 2, 2021	30	0–3	0.00–11.11	0–0	0.00–0.00
	May 3 to May 9, 2021	37	0–10	0.00–37.04	0–0	0.00–0.00
	May 10 to May 16, 2021	42	4–17	16.00–68.00	0–0	0.00–0.00
	May 17 to May 23, 2021	37	0–13	0.00–54.17	0–0	0.00–0.00
	May 24 to May 30, 2021	32	0–8	0.00–33.33	0–0	0.00–0.00
Osaka						
	Apr 5 to Apr 11, 2021	118	0–0	0.00–0.00	0–0	0.00–0.00
	Apr 12 to Apr 18, 2021	117	0–5	0.00–4.46	0–0	0.00–0.00
	Apr 19 to Apr 25, 2021	111	0–3	0.00–2.78	0–0	0.00–0.00
	Apr 26 to May 2, 2021	144	11–41	10.68–39.81	0–0	0.00–0.00
	May 3 to May 9, 2021	132	0–32	0.00–32.00	0–0	0.00–0.00
	May 10 to May 16, 2021	135	8–38	8.25–39.18	0–0	0.00–0.00
	May 17 to May 23, 2021	113	0–19	0.00–20.21	0–0	0.00–0.00
	May 24 to May 30, 2021	109	0–19	0.00–21.11	0–0	0.00–0.00
Hyogo						
	Apr 5 to Apr 11, 2021	69	0–7	0.00–11.29	0–0	0.00–0.00
	Apr 12 to Apr 18, 2021	68	0–6	0.00–9.68	0–0	0.00–0.00
	Apr 19 to Apr 25, 2021	67	0–7	0.00–11.67	0–0	0.00–0.00
	Apr 26 to May 2, 2021	72	0–15	0.00–26.32	0–0	0.00–0.00
	May 3 to May 9, 2021	66	0–12	0.00–22.22	0–0	0.00–0.00
	May 10 to May 16, 2021	66	0–13	0.00–24.53	0–0	0.00–0.00
	May 17 to May 23, 2021	56	0–5	0.00–9.80	0–0	0.00–0.00
	May 24 to May 30, 2021	79	8–29	16.00–58.00	0–0	0.00–0.00
Nara						
	Apr 5 to Apr 11, 2021	18	0–1	0.00–5.88	0–0	0.00–0.00
	Apr 12 to Apr 18, 2021	24	0–7	0.00–41.18	0–0	0.00–0.00
	Apr 19 to Apr 25, 2021	11	0–0	0.00–0.00	0–6	0.00–35.29
	Apr 26 to May 2, 2021	24	0–8	0.00–50.00	0–0	0.00–0.00
	May 3 to May 9, 2021	14	0–0	0.00–0.00	0–2	0.00–12.50
	May 10 to May 16, 2021	14	0–0	0.00–0.00	0–1	0.00–6.67
	May 17 to May 23, 2021	12	0–0	0.00–0.00	0–3	0.00–20.00
	May 24 to May 30, 2021	8	0–0	0.00–0.00	0–6	0.00–42.86
Wakayama						
	Apr 5 to Apr 11, 2021	10	0–0	0.00–0.00	0–2	0.00–16.67

	Apr 12 to Apr 18, 2021	10	0–0	0.00–0.00	0–1	0.00–9.09
	Apr 19 to Apr 25, 2021	15	0–4	0.00–36.36	0–0	0.00–0.00
	Apr 26 to May 2, 2021	15	0–5	0.00–50.00	0–0	0.00–0.00
	May 3 to May 9, 2021	11	0–1	0.00–10.00	0–0	0.00–0.00
	May 10 to May 16, 2021	14	0–5	0.00–55.56	0–0	0.00–0.00
	May 17 to May 23, 2021	6	0–0	0.00–0.00	0–4	0.00–40.00
	May 24 to May 30, 2021	6	0–0	0.00–0.00	0–4	0.00–40.00
Tottori						
	Apr 5 to Apr 11, 2021	5	0–0	0.00–0.00	0–0	0.00–0.00
	Apr 12 to Apr 18, 2021	9	0–4	0.00–80.00	0–0	0.00–0.00
	Apr 19 to Apr 25, 2021	6	0–1	0.00–20.00	0–0	0.00–0.00
	Apr 26 to May 2, 2021	2	0–0	0.00–0.00	0–3	0.00–60.00
	May 3 to May 9, 2021	10	0–5	0.00–100.00	0–0	0.00–0.00
	May 10 to May 16, 2021	8	0–3	0.00–60.00	0–0	0.00–0.00
	May 17 to May 23, 2021	8	0–3	0.00–60.00	0–0	0.00–0.00
	May 24 to May 30, 2021	5	0–1	0.00–25.00	0–0	0.00–0.00
Shimane						
	Apr 5 to Apr 11, 2021	5	0–0	0.00–0.00	0–2	0.00–28.57
	Apr 12 to Apr 18, 2021	12	0–5	0.00–71.43	0–0	0.00–0.00
	Apr 19 to Apr 25, 2021	5	0–0	0.00–0.00	0–2	0.00–28.57
	Apr 26 to May 2, 2021	5	0–0	0.00–0.00	0–2	0.00–28.57
	May 3 to May 9, 2021	7	0–1	0.00–16.67	0–0	0.00–0.00
	May 10 to May 16, 2021	3	0–0	0.00–0.00	0–3	0.00–50.00
	May 17 to May 23, 2021	6	0–0	0.00–0.00	0–0	0.00–0.00
	May 24 to May 30, 2021	6	0–0	0.00–0.00	0–0	0.00–0.00
Okayama						
	Apr 5 to Apr 11, 2021	16	0–0	0.00–0.00	0–4	0.00–20.00
	Apr 12 to Apr 18, 2021	9	0–0	0.00–0.00	0–9	0.00–50.00
	Apr 19 to Apr 25, 2021	15	0–0	0.00–0.00	0–3	0.00–16.67
	Apr 26 to May 2, 2021	20	0–3	0.00–17.65	0–0	0.00–0.00
	May 3 to May 9, 2021	22	0–6	0.00–37.50	0–0	0.00–0.00
	May 10 to May 16, 2021	25	0–10	0.00–66.67	0–0	0.00–0.00
	May 17 to May 23, 2021	16	0–1	0.00–6.67	0–0	0.00–0.00
	May 24 to May 30, 2021	24	1–10	7.14–71.43	0–0	0.00–0.00
Hiroshima						
	Apr 5 to Apr 11, 2021	38	2–12	7.69–46.15	0–0	0.00–0.00
	Apr 12 to Apr 18, 2021	43	7–18	28.00–72.00	0–0	0.00–0.00
	Apr 19 to Apr 25, 2021	31	0–7	0.00–29.17	0–0	0.00–0.00
	Apr 26 to May 2, 2021	31	0–7	0.00–29.17	0–0	0.00–0.00
	May 3 to May 9, 2021	17	0–0	0.00–0.00	0–6	0.00–26.09
	May 10 to May 16, 2021	23	0–1	0.00–4.55	0–0	0.00–0.00
	May 17 to May 23, 2021	21	0–0	0.00–0.00	0–1	0.00–4.55
	May 24 to May 30, 2021	18	0–0	0.00–0.00	0–4	0.00–18.18
Yamaguchi						
	Apr 5 to Apr 11, 2021	16	0–0	0.00–0.00	0–4	0.00–20.00
	Apr 12 to Apr 18, 2021	17	0–0	0.00–0.00	0–2	0.00–10.53

	Apr 19 to Apr 25, 2021	21	0–3	0.00–16.67	0–0	0.00–0.00
	Apr 26 to May 2, 2021	23	0–6	0.00–35.29	0–0	0.00–0.00
	May 3 to May 9, 2021	17	0–1	0.00–6.25	0–0	0.00–0.00
	May 10 to May 16, 2021	10	0–0	0.00–0.00	0–5	0.00–33.33
	May 17 to May 23, 2021	21	0–6	0.00–40.00	0–0	0.00–0.00
	May 24 to May 30, 2021	13	0–0	0.00–0.00	0–1	0.00–7.14
Tokushima						
	Apr 5 to Apr 11, 2021	7	0–1	0.00–16.67	0–0	0.00–0.00
	Apr 12 to Apr 18, 2021	10	0–5	0.00–100.00	0–0	0.00–0.00
	Apr 19 to Apr 25, 2021	4	0–0	0.00–0.00	0–1	0.00–20.00
	Apr 26 to May 2, 2021	14	3–8	50.00–133.33	0–0	0.00–0.00
	May 3 to May 9, 2021	12	2–6	33.33–100.00	0–0	0.00–0.00
	May 10 to May 16, 2021	9	0–4	0.00–80.00	0–0	0.00–0.00
	May 17 to May 23, 2021	7	0–2	0.00–40.00	0–0	0.00–0.00
	May 24 to May 30, 2021	7	0–2	0.00–40.00	0–0	0.00–0.00
Kagawa						
	Apr 5 to Apr 11, 2021	16	0–3	0.00–23.08	0–0	0.00–0.00
	Apr 12 to Apr 18, 2021	19	0–6	0.00–46.15	0–0	0.00–0.00
	Apr 19 to Apr 25, 2021	17	0–6	0.00–54.55	0–0	0.00–0.00
	Apr 26 to May 2, 2021	13	0–2	0.00–18.18	0–0	0.00–0.00
	May 3 to May 9, 2021	9	0–0	0.00–0.00	0–1	0.00–10.00
	May 10 to May 16, 2021	13	0–3	0.00–30.00	0–0	0.00–0.00
	May 17 to May 23, 2021	9	0–0	0.00–0.00	0–0	0.00–0.00
	May 24 to May 30, 2021	7	0–0	0.00–0.00	0–2	0.00–22.22
Ehime						
	Apr 5 to Apr 11, 2021	14	0–0	0.00–0.00	0–3	0.00–17.65
	Apr 12 to Apr 18, 2021	25	0–8	0.00–47.06	0–0	0.00–0.00
	Apr 19 to Apr 25, 2021	24	0–8	0.00–50.00	0–0	0.00–0.00
	Apr 26 to May 2, 2021	19	0–3	0.00–18.75	0–0	0.00–0.00
	May 3 to May 9, 2021	22	0–8	0.00–57.14	0–0	0.00–0.00
	May 10 to May 16, 2021	23	1–9	7.14–64.29	0–0	0.00–0.00
	May 17 to May 23, 2021	12	0–0	0.00–0.00	0–1	0.00–7.69
	May 24 to May 30, 2021	11	0–0	0.00–0.00	0–2	0.00–15.38
Kochi						
	Apr 5 to Apr 11, 2021	7	0–0	0.00–0.00	0–0	0.00–0.00
	Apr 12 to Apr 18, 2021	1	0–0	0.00–0.00	1–6	14.29–85.71
	Apr 19 to Apr 25, 2021	7	0–1	0.00–16.67	0–0	0.00–0.00
	Apr 26 to May 2, 2021	6	0–0	0.00–0.00	0–0	0.00–0.00
	May 3 to May 9, 2021	8	0–2	0.00–33.33	0–0	0.00–0.00
	May 10 to May 16, 2021	5	0–0	0.00–0.00	0–1	0.00–16.67
	May 17 to May 23, 2021	7	0–1	0.00–16.67	0–0	0.00–0.00
	May 24 to May 30, 2021	11	0–5	0.00–83.33	0–0	0.00–0.00
Fukuoka						
	Apr 5 to Apr 11, 2021	21	0–0	0.00–0.00	0–0	0.00–0.00
	Apr 12 to Apr 18, 2021	18	0–0	0.00–0.00	0–3	0.00–14.29
	Apr 19 to Apr 25, 2021	21	0–0	0.00–0.00	0–0	0.00–0.00

	Apr 26 to May 2, 2021	25	0–4	0.00–19.05	0–0	0.00–0.00
	May 3 to May 9, 2021	27	0–7	0.00–35.00	0–0	0.00–0.00
	May 10 to May 16, 2021	20	0–0	0.00–0.00	0–0	0.00–0.00
	May 17 to May 23, 2021	20	0–1	0.00–5.26	0–0	0.00–0.00
	May 24 to May 30, 2021	17	0–0	0.00–0.00	0–2	0.00–10.53
Saga						
	Apr 5 to Apr 11, 2021	4	0–0	0.00–0.00	0–2	0.00–33.33
	Apr 12 to Apr 18, 2021	4	0–0	0.00–0.00	0–2	0.00–33.33
	Apr 19 to Apr 25, 2021	2	0–0	0.00–0.00	0–4	0.00–66.67
	Apr 26 to May 2, 2021	2	0–0	0.00–0.00	0–3	0.00–60.00
	May 3 to May 9, 2021	7	0–2	0.00–40.00	0–0	0.00–0.00
	May 10 to May 16, 2021	3	0–0	0.00–0.00	0–2	0.00–40.00
	May 17 to May 23, 2021	5	0–1	0.00–25.00	0–0	0.00–0.00
	May 24 to May 30, 2021	3	0–0	0.00–0.00	0–2	0.00–40.00
Nagasaki						
	Apr 5 to Apr 11, 2021	9	0–0	0.00–0.00	0–0	0.00–0.00
	Apr 12 to Apr 18, 2021	11	0–2	0.00–22.22	0–0	0.00–0.00
	Apr 19 to Apr 25, 2021	12	0–4	0.00–50.00	0–0	0.00–0.00
	Apr 26 to May 2, 2021	8	0–0	0.00–0.00	0–0	0.00–0.00
	May 3 to May 9, 2021	13	0–5	0.00–62.50	0–0	0.00–0.00
	May 10 to May 16, 2021	10	0–2	0.00–25.00	0–0	0.00–0.00
	May 17 to May 23, 2021	10	0–2	0.00–25.00	0–0	0.00–0.00
	May 24 to May 30, 2021	12	0–5	0.00–71.43	0–0	0.00–0.00
Kumamoto						
	Apr 5 to Apr 11, 2021	13	0–0	0.00–0.00	0–1	0.00–7.14
	Apr 12 to Apr 18, 2021	19	0–6	0.00–46.15	0–0	0.00–0.00
	Apr 19 to Apr 25, 2021	10	0–0	0.00–0.00	0–2	0.00–16.67
	Apr 26 to May 2, 2021	17	0–5	0.00–41.67	0–0	0.00–0.00
	May 3 to May 9, 2021	19	1–8	9.09–72.73	0–0	0.00–0.00
	May 10 to May 16, 2021	14	0–3	0.00–27.27	0–0	0.00–0.00
	May 17 to May 23, 2021	14	0–4	0.00–40.00	0–0	0.00–0.00
	May 24 to May 30, 2021	13	0–3	0.00–30.00	0–0	0.00–0.00
Oita						
	Apr 5 to Apr 11, 2021	11	0–2	0.00–22.22	0–0	0.00–0.00
	Apr 12 to Apr 18, 2021	7	0–0	0.00–0.00	0–1	0.00–12.50
	Apr 19 to Apr 25, 2021	11	0–3	0.00–37.50	0–0	0.00–0.00
	Apr 26 to May 2, 2021	11	0–3	0.00–37.50	0–0	0.00–0.00
	May 3 to May 9, 2021	15	2–8	28.57–114.29	0–0	0.00–0.00
	May 10 to May 16, 2021	10	0–3	0.00–42.86	0–0	0.00–0.00
	May 17 to May 23, 2021	9	0–2	0.00–28.57	0–0	0.00–0.00
	May 24 to May 30, 2021	8	0–1	0.00–14.29	0–0	0.00–0.00
Miyazaki						
	Apr 5 to Apr 11, 2021	5	0–0	0.00–0.00	0–5	0.00–50.00
	Apr 12 to Apr 18, 2021	9	0–0	0.00–0.00	0–1	0.00–10.00
	Apr 19 to Apr 25, 2021	9	0–0	0.00–0.00	0–1	0.00–10.00
	Apr 26 to May 2, 2021	5	0–0	0.00–0.00	0–5	0.00–50.00

	May 3 to May 9, 2021	8	0–0	0.00–0.00	0–1	0.00–11.11
	May 10 to May 16, 2021	10	0–1	0.00–11.11	0–0	0.00–0.00
	May 17 to May 23, 2021	8	0–0	0.00–0.00	0–0	0.00–0.00
	May 24 to May 30, 2021	8	0–0	0.00–0.00	0–0	0.00–0.00
Kagoshima						
	Apr 5 to Apr 11, 2021	11	0–0	0.00–0.00	0–1	0.00–8.33
	Apr 12 to Apr 18, 2021	13	0–2	0.00–18.18	0–0	0.00–0.00
	Apr 19 to Apr 25, 2021	11	0–0	0.00–0.00	0–0	0.00–0.00
	Apr 26 to May 2, 2021	12	0–2	0.00–20.00	0–0	0.00–0.00
	May 3 to May 9, 2021	13	0–3	0.00–30.00	0–0	0.00–0.00
	May 10 to May 16, 2021	14	0–4	0.00–40.00	0–0	0.00–0.00
	May 17 to May 23, 2021	15	0–6	0.00–66.67	0–0	0.00–0.00
	May 24 to May 30, 2021	15	0–6	0.00–66.67	0–0	0.00–0.00
Okinawa						
	Apr 5 to Apr 11, 2021	17	0–6	0.00–54.55	0–0	0.00–0.00
	Apr 12 to Apr 18, 2021	12	0–1	0.00–9.09	0–0	0.00–0.00
	Apr 19 to Apr 25, 2021	7	0–0	0.00–0.00	0–4	0.00–36.36
	Apr 26 to May 2, 2021	8	0–0	0.00–0.00	0–2	0.00–20.00
	May 3 to May 9, 2021	9	0–0	0.00–0.00	0–1	0.00–10.00
	May 10 to May 16, 2021	17	0–6	0.00–54.55	0–0	0.00–0.00
	May 17 to May 23, 2021	13	0–2	0.00–18.18	0–0	0.00–0.00
	May 24 to May 30, 2021	14	0–3	0.00–27.27	0–0	0.00–0.00

Table A.13: Weekly number of observed and excess/exiguous deaths in Japan and 47 prefectures for malignant neoplasms-related deaths in all places from January 2020 through May 2021.

Prefecture	Week	Observed	Excess deaths	Percent excess	Exiguous deaths	Percent exiguous
Japan						
	Apr 5 to Apr 11, 2021	7221	0–21	0.00–0.29	0–0	0.00–0.00
	Apr 12 to Apr 18, 2021	7439	46–252	0.64–3.51	0–0	0.00–0.00
	Apr 19 to Apr 25, 2021	7439	59–264	0.82–3.68	0–0	0.00–0.00
	Apr 26 to May 2, 2021	7620	265–471	3.71–6.59	0–0	0.00–0.00
	May 3 to May 9, 2021	7474	145–344	2.03–4.82	0–0	0.00–0.00
	May 10 to May 16, 2021	7357	56–249	0.79–3.50	0–0	0.00–0.00
	May 17 to May 23, 2021	7274	0–172	0.00–2.42	0–0	0.00–0.00
	May 24 to May 30, 2021	7308	0–187	0.00–2.63	0–0	0.00–0.00
Hokkaido						
	Apr 5 to Apr 11, 2021	357	0–0	0.00–0.00	0–21	0.00–5.56
	Apr 12 to Apr 18, 2021	405	0–30	0.00–8.00	0–0	0.00–0.00
	Apr 19 to Apr 25, 2021	373	0–0	0.00–0.00	0–2	0.00–0.53
	Apr 26 to May 2, 2021	398	0–25	0.00–6.70	0–0	0.00–0.00
	May 3 to May 9, 2021	387	0–15	0.00–4.03	0–0	0.00–0.00
	May 10 to May 16, 2021	387	0–14	0.00–3.75	0–0	0.00–0.00
	May 17 to May 23, 2021	416	5–43	1.34–11.53	0–0	0.00–0.00
	May 24 to May 30, 2021	389	0–14	0.00–3.73	0–0	0.00–0.00
Aomori						
	Apr 5 to Apr 11, 2021	112	0–17	0.00–17.89	0–0	0.00–0.00
	Apr 12 to Apr 18, 2021	108	0–11	0.00–11.34	0–0	0.00–0.00
	Apr 19 to Apr 25, 2021	104	0–6	0.00–6.12	0–0	0.00–0.00
	Apr 26 to May 2, 2021	107	0–8	0.00–8.08	0–0	0.00–0.00
	May 3 to May 9, 2021	96	0–0	0.00–0.00	0–1	0.00–1.03
	May 10 to May 16, 2021	99	0–3	0.00–3.13	0–0	0.00–0.00
	May 17 to May 23, 2021	111	0–14	0.00–14.43	0–0	0.00–0.00
	May 24 to May 30, 2021	81	0–0	0.00–0.00	0–18	0.00–18.18
Iwate						
	Apr 5 to Apr 11, 2021	95	0–8	0.00–9.20	0–0	0.00–0.00
	Apr 12 to Apr 18, 2021	75	0–0	0.00–0.00	0–11	0.00–12.79
	Apr 19 to Apr 25, 2021	98	0–12	0.00–13.95	0–0	0.00–0.00
	Apr 26 to May 2, 2021	103	0–17	0.00–19.77	0–0	0.00–0.00
	May 3 to May 9, 2021	86	0–0	0.00–0.00	0–0	0.00–0.00
	May 10 to May 16, 2021	87	0–0	0.00–0.00	0–0	0.00–0.00
	May 17 to May 23, 2021	85	0–0	0.00–0.00	0–2	0.00–2.30
	May 24 to May 30, 2021	89	0–3	0.00–3.49	0–0	0.00–0.00
Miyagi						
	Apr 5 to Apr 11, 2021	130	0–0	0.00–0.00	0–1	0.00–0.76
	Apr 12 to Apr 18, 2021	150	0–18	0.00–13.64	0–0	0.00–0.00
	Apr 19 to Apr 25, 2021	130	0–0	0.00–0.00	0–0	0.00–0.00
	Apr 26 to May 2, 2021	150	0–19	0.00–14.50	0–0	0.00–0.00
	May 3 to May 9, 2021	126	0–0	0.00–0.00	0–5	0.00–3.82

	May 10 to May 16, 2021	126	0–0	0.00–0.00	0–4	0.00–3.08
	May 17 to May 23, 2021	117	0–0	0.00–0.00	0–14	0.00–10.69
	May 24 to May 30, 2021	140	0–10	0.00–7.69	0–0	0.00–0.00
Akita						
	Apr 5 to Apr 11, 2021	78	0–0	0.00–0.00	0–0	0.00–0.00
	Apr 12 to Apr 18, 2021	76	0–0	0.00–0.00	0–2	0.00–2.56
	Apr 19 to Apr 25, 2021	76	0–0	0.00–0.00	0–1	0.00–1.30
	Apr 26 to May 2, 2021	84	0–7	0.00–9.09	0–0	0.00–0.00
	May 3 to May 9, 2021	84	0–8	0.00–10.53	0–0	0.00–0.00
	May 10 to May 16, 2021	70	0–0	0.00–0.00	0–6	0.00–7.89
	May 17 to May 23, 2021	96	2–21	2.67–28.00	0–0	0.00–0.00
	May 24 to May 30, 2021	83	0–8	0.00–10.67	0–0	0.00–0.00
Yamagata						
	Apr 5 to Apr 11, 2021	67	0–0	0.00–0.00	0–5	0.00–6.94
	Apr 12 to Apr 18, 2021	72	0–0	0.00–0.00	0–0	0.00–0.00
	Apr 19 to Apr 25, 2021	77	0–5	0.00–6.94	0–0	0.00–0.00
	Apr 26 to May 2, 2021	77	0–6	0.00–8.45	0–0	0.00–0.00
	May 3 to May 9, 2021	84	0–13	0.00–18.31	0–0	0.00–0.00
	May 10 to May 16, 2021	91	2–19	2.78–26.39	0–0	0.00–0.00
	May 17 to May 23, 2021	92	4–21	5.63–29.58	0–0	0.00–0.00
	May 24 to May 30, 2021	74	0–3	0.00–4.23	0–0	0.00–0.00
Fukushima						
	Apr 5 to Apr 11, 2021	107	0–0	0.00–0.00	0–13	0.00–10.83
	Apr 12 to Apr 18, 2021	138	0–18	0.00–15.00	0–0	0.00–0.00
	Apr 19 to Apr 25, 2021	106	0–0	0.00–0.00	0–11	0.00–9.40
	Apr 26 to May 2, 2021	124	0–7	0.00–5.98	0–0	0.00–0.00
	May 3 to May 9, 2021	132	0–16	0.00–13.79	0–0	0.00–0.00
	May 10 to May 16, 2021	110	0–0	0.00–0.00	0–5	0.00–4.35
	May 17 to May 23, 2021	123	0–10	0.00–8.85	0–0	0.00–0.00
	May 24 to May 30, 2021	114	0–0	0.00–0.00	0–1	0.00–0.87
Ibaraki						
	Apr 5 to Apr 11, 2021	159	0–0	0.00–0.00	0–9	0.00–5.36
	Apr 12 to Apr 18, 2021	175	0–9	0.00–5.42	0–0	0.00–0.00
	Apr 19 to Apr 25, 2021	157	0–0	0.00–0.00	0–10	0.00–5.99
	Apr 26 to May 2, 2021	185	0–17	0.00–10.12	0–0	0.00–0.00
	May 3 to May 9, 2021	187	0–18	0.00–10.65	0–0	0.00–0.00
	May 10 to May 16, 2021	186	0–18	0.00–10.71	0–0	0.00–0.00
	May 17 to May 23, 2021	152	0–0	0.00–0.00	0–15	0.00–8.98
	May 24 to May 30, 2021	160	0–0	0.00–0.00	0–6	0.00–3.61
Tochigi						
	Apr 5 to Apr 11, 2021	110	0–0	0.00–0.00	0–0	0.00–0.00
	Apr 12 to Apr 18, 2021	108	0–0	0.00–0.00	0–1	0.00–0.92
	Apr 19 to Apr 25, 2021	114	0–6	0.00–5.56	0–0	0.00–0.00
	Apr 26 to May 2, 2021	110	0–3	0.00–2.80	0–0	0.00–0.00
	May 3 to May 9, 2021	127	0–19	0.00–17.59	0–0	0.00–0.00
	May 10 to May 16, 2021	91	0–0	0.00–0.00	0–16	0.00–14.95

	May 17 to May 23, 2021	93	0–0	0.00–0.00	0–14	0.00–13.08
	May 24 to May 30, 2021	129	1–22	0.93–20.56	0–0	0.00–0.00
Gunma						
	Apr 5 to Apr 11, 2021	122	0–8	0.00–7.02	0–0	0.00–0.00
	Apr 12 to Apr 18, 2021	121	0–6	0.00–5.22	0–0	0.00–0.00
	Apr 19 to Apr 25, 2021	102	0–0	0.00–0.00	0–15	0.00–12.82
	Apr 26 to May 2, 2021	116	0–0	0.00–0.00	0–0	0.00–0.00
	May 3 to May 9, 2021	109	0–0	0.00–0.00	0–7	0.00–6.03
	May 10 to May 16, 2021	109	0–0	0.00–0.00	0–8	0.00–6.84
	May 17 to May 23, 2021	97	0–0	0.00–0.00	0–18	0.00–15.65
	May 24 to May 30, 2021	114	0–0	0.00–0.00	0–1	0.00–0.87
Saitama						
	Apr 5 to Apr 11, 2021	404	0–13	0.00–3.32	0–0	0.00–0.00
	Apr 12 to Apr 18, 2021	394	0–6	0.00–1.55	0–0	0.00–0.00
	Apr 19 to Apr 25, 2021	369	0–0	0.00–0.00	0–18	0.00–4.65
	Apr 26 to May 2, 2021	400	0–12	0.00–3.09	0–0	0.00–0.00
	May 3 to May 9, 2021	411	0–24	0.00–6.20	0–0	0.00–0.00
	May 10 to May 16, 2021	404	0–20	0.00–5.21	0–0	0.00–0.00
	May 17 to May 23, 2021	408	0–23	0.00–5.97	0–0	0.00–0.00
	May 24 to May 30, 2021	398	0–14	0.00–3.65	0–0	0.00–0.00
Chiba						
	Apr 5 to Apr 11, 2021	364	0–24	0.00–7.06	0–0	0.00–0.00
	Apr 12 to Apr 18, 2021	381	6–43	1.78–12.72	0–0	0.00–0.00
	Apr 19 to Apr 25, 2021	322	0–0	0.00–0.00	0–15	0.00–4.45
	Apr 26 to May 2, 2021	325	0–0	0.00–0.00	0–10	0.00–2.99
	May 3 to May 9, 2021	358	0–24	0.00–7.19	0–0	0.00–0.00
	May 10 to May 16, 2021	303	0–0	0.00–0.00	0–25	0.00–7.62
	May 17 to May 23, 2021	306	0–0	0.00–0.00	0–20	0.00–6.13
	May 24 to May 30, 2021	333	0–5	0.00–1.52	0–0	0.00–0.00
Tokyo						
	Apr 5 to Apr 11, 2021	622	0–0	0.00–0.00	0–27	0.00–4.16
	Apr 12 to Apr 18, 2021	676	0–31	0.00–4.81	0–0	0.00–0.00
	Apr 19 to Apr 25, 2021	702	9–60	1.40–9.35	0–0	0.00–0.00
	Apr 26 to May 2, 2021	719	25–78	3.90–12.17	0–0	0.00–0.00
	May 3 to May 9, 2021	661	0–20	0.00–3.12	0–0	0.00–0.00
	May 10 to May 16, 2021	632	0–0	0.00–0.00	0–7	0.00–1.10
	May 17 to May 23, 2021	628	0–0	0.00–0.00	0–11	0.00–1.72
	May 24 to May 30, 2021	648	0–7	0.00–1.09	0–0	0.00–0.00
Kanagawa						
	Apr 5 to Apr 11, 2021	462	0–0	0.00–0.00	0–1	0.00–0.22
	Apr 12 to Apr 18, 2021	466	0–3	0.00–0.65	0–0	0.00–0.00
	Apr 19 to Apr 25, 2021	494	0–28	0.00–6.01	0–0	0.00–0.00
	Apr 26 to May 2, 2021	510	0–44	0.00–9.44	0–0	0.00–0.00
	May 3 to May 9, 2021	491	0–28	0.00–6.05	0–0	0.00–0.00
	May 10 to May 16, 2021	481	0–18	0.00–3.89	0–0	0.00–0.00
	May 17 to May 23, 2021	464	0–3	0.00–0.65	0–0	0.00–0.00

	May 24 to May 30, 2021	483	0–22	0.00–4.77	0–0	0.00–0.00
Niigata						
	Apr 5 to Apr 11, 2021	137	0–0	0.00–0.00	0–11	0.00–7.43
	Apr 12 to Apr 18, 2021	143	0–0	0.00–0.00	0–6	0.00–4.03
	Apr 19 to Apr 25, 2021	153	0–3	0.00–2.00	0–0	0.00–0.00
	Apr 26 to May 2, 2021	147	0–0	0.00–0.00	0–3	0.00–2.00
	May 3 to May 9, 2021	119	0–0	0.00–0.00	6–29	4.05–19.59
	May 10 to May 16, 2021	140	0–0	0.00–0.00	0–8	0.00–5.41
	May 17 to May 23, 2021	127	0–0	0.00–0.00	0–21	0.00–14.19
	May 24 to May 30, 2021	152	0–3	0.00–2.01	0–0	0.00–0.00
Toyama						
	Apr 5 to Apr 11, 2021	75	0–9	0.00–13.64	0–0	0.00–0.00
	Apr 12 to Apr 18, 2021	70	0–4	0.00–6.06	0–0	0.00–0.00
	Apr 19 to Apr 25, 2021	72	0–7	0.00–10.77	0–0	0.00–0.00
	Apr 26 to May 2, 2021	77	0–11	0.00–16.67	0–0	0.00–0.00
	May 3 to May 9, 2021	75	0–10	0.00–15.38	0–0	0.00–0.00
	May 10 to May 16, 2021	71	0–5	0.00–7.58	0–0	0.00–0.00
	May 17 to May 23, 2021	53	0–0	0.00–0.00	0–13	0.00–19.70
	May 24 to May 30, 2021	68	0–1	0.00–1.49	0–0	0.00–0.00
Ishikawa						
	Apr 5 to Apr 11, 2021	78	0–11	0.00–16.42	0–0	0.00–0.00
	Apr 12 to Apr 18, 2021	74	0–7	0.00–10.45	0–0	0.00–0.00
	Apr 19 to Apr 25, 2021	76	0–9	0.00–13.43	0–0	0.00–0.00
	Apr 26 to May 2, 2021	70	0–4	0.00–6.06	0–0	0.00–0.00
	May 3 to May 9, 2021	74	0–7	0.00–10.45	0–0	0.00–0.00
	May 10 to May 16, 2021	65	0–0	0.00–0.00	0–2	0.00–2.99
	May 17 to May 23, 2021	69	0–2	0.00–2.99	0–0	0.00–0.00
	May 24 to May 30, 2021	56	0–0	0.00–0.00	0–11	0.00–16.42
Fukui						
	Apr 5 to Apr 11, 2021	41	0–0	0.00–0.00	0–2	0.00–4.65
	Apr 12 to Apr 18, 2021	51	0–7	0.00–15.91	0–0	0.00–0.00
	Apr 19 to Apr 25, 2021	45	0–2	0.00–4.65	0–0	0.00–0.00
	Apr 26 to May 2, 2021	50	0–7	0.00–16.28	0–0	0.00–0.00
	May 3 to May 9, 2021	54	0–12	0.00–28.57	0–0	0.00–0.00
	May 10 to May 16, 2021	46	0–4	0.00–9.52	0–0	0.00–0.00
	May 17 to May 23, 2021	46	0–3	0.00–6.98	0–0	0.00–0.00
	May 24 to May 30, 2021	46	0–4	0.00–9.52	0–0	0.00–0.00
Yamanashi						
	Apr 5 to Apr 11, 2021	42	0–0	0.00–0.00	0–7	0.00–14.29
	Apr 12 to Apr 18, 2021	39	0–0	0.00–0.00	0–10	0.00–20.41
	Apr 19 to Apr 25, 2021	44	0–0	0.00–0.00	0–4	0.00–8.33
	Apr 26 to May 2, 2021	46	0–0	0.00–0.00	0–3	0.00–6.12
	May 3 to May 9, 2021	61	0–13	0.00–27.08	0–0	0.00–0.00
	May 10 to May 16, 2021	59	0–11	0.00–22.92	0–0	0.00–0.00
	May 17 to May 23, 2021	65	2–16	4.08–32.65	0–0	0.00–0.00
	May 24 to May 30, 2021	56	0–8	0.00–16.67	0–0	0.00–0.00

Nagano						
	Apr 5 to Apr 11, 2021	108	0–0	0.00–0.00	0–13	0.00–10.74
	Apr 12 to Apr 18, 2021	123	0–2	0.00–1.65	0–0	0.00–0.00
	Apr 19 to Apr 25, 2021	113	0–0	0.00–0.00	0–6	0.00–5.04
	Apr 26 to May 2, 2021	120	0–3	0.00–2.56	0–0	0.00–0.00
	May 3 to May 9, 2021	107	0–0	0.00–0.00	0–11	0.00–9.32
	May 10 to May 16, 2021	123	0–4	0.00–3.36	0–0	0.00–0.00
	May 17 to May 23, 2021	123	0–6	0.00–5.13	0–0	0.00–0.00
	May 24 to May 30, 2021	101	0–0	0.00–0.00	0–18	0.00–15.13
Gifu						
	Apr 5 to Apr 11, 2021	123	0–6	0.00–5.13	0–0	0.00–0.00
	Apr 12 to Apr 18, 2021	130	0–15	0.00–13.04	0–0	0.00–0.00
	Apr 19 to Apr 25, 2021	133	0–19	0.00–16.67	0–0	0.00–0.00
	Apr 26 to May 2, 2021	105	0–0	0.00–0.00	0–8	0.00–7.08
	May 3 to May 9, 2021	139	3–24	2.61–20.87	0–0	0.00–0.00
	May 10 to May 16, 2021	126	0–12	0.00–10.53	0–0	0.00–0.00
	May 17 to May 23, 2021	136	0–21	0.00–18.26	0–0	0.00–0.00
	May 24 to May 30, 2021	116	0–0	0.00–0.00	0–0	0.00–0.00
Shizuoka						
	Apr 5 to Apr 11, 2021	229	0–19	0.00–9.05	0–0	0.00–0.00
	Apr 12 to Apr 18, 2021	199	0–0	0.00–0.00	0–9	0.00–4.33
	Apr 19 to Apr 25, 2021	204	0–0	0.00–0.00	0–3	0.00–1.45
	Apr 26 to May 2, 2021	230	0–22	0.00–10.58	0–0	0.00–0.00
	May 3 to May 9, 2021	212	0–6	0.00–2.91	0–0	0.00–0.00
	May 10 to May 16, 2021	200	0–0	0.00–0.00	0–6	0.00–2.91
	May 17 to May 23, 2021	245	9–38	4.35–18.36	0–0	0.00–0.00
	May 24 to May 30, 2021	203	0–0	0.00–0.00	0–3	0.00–1.46
Aichi						
	Apr 5 to Apr 11, 2021	356	0–0	0.00–0.00	0–23	0.00–6.07
	Apr 12 to Apr 18, 2021	369	0–0	0.00–0.00	0–9	0.00–2.38
	Apr 19 to Apr 25, 2021	363	0–0	0.00–0.00	0–13	0.00–3.46
	Apr 26 to May 2, 2021	391	0–13	0.00–3.44	0–0	0.00–0.00
	May 3 to May 9, 2021	373	0–0	0.00–0.00	0–8	0.00–2.10
	May 10 to May 16, 2021	415	0–37	0.00–9.79	0–0	0.00–0.00
	May 17 to May 23, 2021	339	0–0	0.00–0.00	0–35	0.00–9.36
	May 24 to May 30, 2021	367	0–0	0.00–0.00	0–5	0.00–1.34
Mie						
	Apr 5 to Apr 11, 2021	92	0–0	0.00–0.00	0–9	0.00–8.91
	Apr 12 to Apr 18, 2021	103	0–4	0.00–4.04	0–0	0.00–0.00
	Apr 19 to Apr 25, 2021	103	0–5	0.00–5.10	0–0	0.00–0.00
	Apr 26 to May 2, 2021	94	0–0	0.00–0.00	0–4	0.00–4.08
	May 3 to May 9, 2021	111	0–15	0.00–15.63	0–0	0.00–0.00
	May 10 to May 16, 2021	117	1–21	1.04–21.88	0–0	0.00–0.00
	May 17 to May 23, 2021	118	4–24	4.26–25.53	0–0	0.00–0.00
	May 24 to May 30, 2021	103	0–8	0.00–8.42	0–0	0.00–0.00
Shiga						

	Apr 5 to Apr 11, 2021	72	0–0	0.00–0.00	0–0	0.00–0.00
	Apr 12 to Apr 18, 2021	73	0–2	0.00–2.82	0–0	0.00–0.00
	Apr 19 to Apr 25, 2021	69	0–0	0.00–0.00	0–1	0.00–1.43
	Apr 26 to May 2, 2021	84	0–14	0.00–20.00	0–0	0.00–0.00
	May 3 to May 9, 2021	58	0–0	0.00–0.00	0–12	0.00–17.14
	May 10 to May 16, 2021	73	0–5	0.00–7.35	0–0	0.00–0.00
	May 17 to May 23, 2021	77	0–7	0.00–10.00	0–0	0.00–0.00
	May 24 to May 30, 2021	78	0–8	0.00–11.43	0–0	0.00–0.00
Kyoto						
	Apr 5 to Apr 11, 2021	145	0–0	0.00–0.00	0–2	0.00–1.36
	Apr 12 to Apr 18, 2021	163	0–16	0.00–10.88	0–0	0.00–0.00
	Apr 19 to Apr 25, 2021	166	0–20	0.00–13.70	0–0	0.00–0.00
	Apr 26 to May 2, 2021	159	0–12	0.00–8.16	0–0	0.00–0.00
	May 3 to May 9, 2021	152	0–4	0.00–2.70	0–0	0.00–0.00
	May 10 to May 16, 2021	154	0–6	0.00–4.05	0–0	0.00–0.00
	May 17 to May 23, 2021	137	0–0	0.00–0.00	0–12	0.00–8.05
	May 24 to May 30, 2021	153	0–4	0.00–2.68	0–0	0.00–0.00
Osaka						
	Apr 5 to Apr 11, 2021	554	0–31	0.00–5.93	0–0	0.00–0.00
	Apr 12 to Apr 18, 2021	513	0–0	0.00–0.00	0–6	0.00–1.16
	Apr 19 to Apr 25, 2021	562	0–43	0.00–8.29	0–0	0.00–0.00
	Apr 26 to May 2, 2021	565	2–49	0.39–9.50	0–0	0.00–0.00
	May 3 to May 9, 2021	548	0–32	0.00–6.20	0–0	0.00–0.00
	May 10 to May 16, 2021	507	0–0	0.00–0.00	0–7	0.00–1.36
	May 17 to May 23, 2021	514	0–1	0.00–0.19	0–0	0.00–0.00
	May 24 to May 30, 2021	492	0–0	0.00–0.00	0–20	0.00–3.91
Hyogo						
	Apr 5 to Apr 11, 2021	319	0–6	0.00–1.92	0–0	0.00–0.00
	Apr 12 to Apr 18, 2021	326	0–17	0.00–5.50	0–0	0.00–0.00
	Apr 19 to Apr 25, 2021	318	0–11	0.00–3.58	0–0	0.00–0.00
	Apr 26 to May 2, 2021	368	30–64	9.87–21.05	0–0	0.00–0.00
	May 3 to May 9, 2021	318	0–15	0.00–4.95	0–0	0.00–0.00
	May 10 to May 16, 2021	314	0–10	0.00–3.29	0–0	0.00–0.00
	May 17 to May 23, 2021	326	0–21	0.00–6.89	0–0	0.00–0.00
	May 24 to May 30, 2021	321	0–14	0.00–4.56	0–0	0.00–0.00
Nara						
	Apr 5 to Apr 11, 2021	84	0–4	0.00–5.00	0–0	0.00–0.00
	Apr 12 to Apr 18, 2021	82	0–1	0.00–1.23	0–0	0.00–0.00
	Apr 19 to Apr 25, 2021	85	0–5	0.00–6.25	0–0	0.00–0.00
	Apr 26 to May 2, 2021	74	0–0	0.00–0.00	0–8	0.00–9.76
	May 3 to May 9, 2021	91	0–8	0.00–9.64	0–0	0.00–0.00
	May 10 to May 16, 2021	95	0–12	0.00–14.46	0–0	0.00–0.00
	May 17 to May 23, 2021	72	0–0	0.00–0.00	0–10	0.00–12.20
	May 24 to May 30, 2021	85	0–2	0.00–2.41	0–0	0.00–0.00
Wakayama						
	Apr 5 to Apr 11, 2021	61	0–0	0.00–0.00	0–2	0.00–3.17

	Apr 12 to Apr 18, 2021	63	0–0	0.00–0.00	0–0	0.00–0.00
	Apr 19 to Apr 25, 2021	57	0–0	0.00–0.00	0–6	0.00–9.52
	Apr 26 to May 2, 2021	65	0–2	0.00–3.17	0–0	0.00–0.00
	May 3 to May 9, 2021	77	0–16	0.00–26.23	0–0	0.00–0.00
	May 10 to May 16, 2021	72	0–10	0.00–16.13	0–0	0.00–0.00
	May 17 to May 23, 2021	71	0–10	0.00–16.39	0–0	0.00–0.00
	May 24 to May 30, 2021	70	0–8	0.00–12.90	0–0	0.00–0.00
Tottori						
	Apr 5 to Apr 11, 2021	41	0–6	0.00–17.14	0–0	0.00–0.00
	Apr 12 to Apr 18, 2021	33	0–0	0.00–0.00	0–2	0.00–5.71
	Apr 19 to Apr 25, 2021	34	0–0	0.00–0.00	0–0	0.00–0.00
	Apr 26 to May 2, 2021	43	0–9	0.00–26.47	0–0	0.00–0.00
	May 3 to May 9, 2021	43	0–10	0.00–30.30	0–0	0.00–0.00
	May 10 to May 16, 2021	39	0–4	0.00–11.43	0–0	0.00–0.00
	May 17 to May 23, 2021	44	0–9	0.00–25.71	0–0	0.00–0.00
	May 24 to May 30, 2021	46	0–10	0.00–27.78	0–0	0.00–0.00
Shimane						
	Apr 5 to Apr 11, 2021	38	0–0	0.00–0.00	0–8	0.00–17.39
	Apr 12 to Apr 18, 2021	48	0–3	0.00–6.67	0–0	0.00–0.00
	Apr 19 to Apr 25, 2021	47	0–2	0.00–4.44	0–0	0.00–0.00
	Apr 26 to May 2, 2021	53	0–9	0.00–20.45	0–0	0.00–0.00
	May 3 to May 9, 2021	55	0–11	0.00–25.00	0–0	0.00–0.00
	May 10 to May 16, 2021	44	0–0	0.00–0.00	0–0	0.00–0.00
	May 17 to May 23, 2021	37	0–0	0.00–0.00	0–7	0.00–15.91
	May 24 to May 30, 2021	53	0–9	0.00–20.45	0–0	0.00–0.00
Okayama						
	Apr 5 to Apr 11, 2021	112	0–6	0.00–5.66	0–0	0.00–0.00
	Apr 12 to Apr 18, 2021	106	0–1	0.00–0.95	0–0	0.00–0.00
	Apr 19 to Apr 25, 2021	109	0–3	0.00–2.83	0–0	0.00–0.00
	Apr 26 to May 2, 2021	104	0–0	0.00–0.00	0–4	0.00–3.70
	May 3 to May 9, 2021	103	0–0	0.00–0.00	0–6	0.00–5.50
	May 10 to May 16, 2021	127	0–18	0.00–16.51	0–0	0.00–0.00
	May 17 to May 23, 2021	109	0–2	0.00–1.87	0–0	0.00–0.00
	May 24 to May 30, 2021	109	0–1	0.00–0.93	0–0	0.00–0.00
Hiroshima						
	Apr 5 to Apr 11, 2021	161	0–7	0.00–4.55	0–0	0.00–0.00
	Apr 12 to Apr 18, 2021	167	0–12	0.00–7.74	0–0	0.00–0.00
	Apr 19 to Apr 25, 2021	172	0–17	0.00–10.97	0–0	0.00–0.00
	Apr 26 to May 2, 2021	150	0–0	0.00–0.00	0–4	0.00–2.60
	May 3 to May 9, 2021	165	0–14	0.00–9.27	0–0	0.00–0.00
	May 10 to May 16, 2021	169	0–20	0.00–13.42	0–0	0.00–0.00
	May 17 to May 23, 2021	168	0–17	0.00–11.26	0–0	0.00–0.00
	May 24 to May 30, 2021	158	0–7	0.00–4.64	0–0	0.00–0.00
Yamaguchi						
	Apr 5 to Apr 11, 2021	71	0–0	0.00–0.00	2–20	2.20–21.98
	Apr 12 to Apr 18, 2021	89	0–0	0.00–0.00	0–2	0.00–2.20

	Apr 19 to Apr 25, 2021	107	0–16	0.00–17.58	0–0	0.00–0.00
	Apr 26 to May 2, 2021	93	0–1	0.00–1.09	0–0	0.00–0.00
	May 3 to May 9, 2021	96	0–6	0.00–6.67	0–0	0.00–0.00
	May 10 to May 16, 2021	99	0–10	0.00–11.24	0–0	0.00–0.00
	May 17 to May 23, 2021	90	0–0	0.00–0.00	0–0	0.00–0.00
	May 24 to May 30, 2021	107	0–18	0.00–20.22	0–0	0.00–0.00
Tokushima						
	Apr 5 to Apr 11, 2021	58	1–14	2.27–31.82	0–0	0.00–0.00
	Apr 12 to Apr 18, 2021	30	0–0	0.00–0.00	1–14	2.27–31.82
	Apr 19 to Apr 25, 2021	56	0–12	0.00–27.27	0–0	0.00–0.00
	Apr 26 to May 2, 2021	46	0–2	0.00–4.55	0–0	0.00–0.00
	May 3 to May 9, 2021	32	0–0	0.00–0.00	0–12	0.00–27.27
	May 10 to May 16, 2021	52	0–7	0.00–15.56	0–0	0.00–0.00
	May 17 to May 23, 2021	36	0–0	0.00–0.00	0–9	0.00–20.00
	May 24 to May 30, 2021	43	0–0	0.00–0.00	0–2	0.00–4.44
Kagawa						
	Apr 5 to Apr 11, 2021	50	0–0	0.00–0.00	0–9	0.00–15.25
	Apr 12 to Apr 18, 2021	62	0–2	0.00–3.33	0–0	0.00–0.00
	Apr 19 to Apr 25, 2021	63	0–4	0.00–6.78	0–0	0.00–0.00
	Apr 26 to May 2, 2021	50	0–0	0.00–0.00	0–7	0.00–12.28
	May 3 to May 9, 2021	62	0–6	0.00–10.71	0–0	0.00–0.00
	May 10 to May 16, 2021	52	0–0	0.00–0.00	0–3	0.00–5.45
	May 17 to May 23, 2021	68	0–13	0.00–23.64	0–0	0.00–0.00
	May 24 to May 30, 2021	61	0–7	0.00–12.96	0–0	0.00–0.00
Ehime						
	Apr 5 to Apr 11, 2021	90	0–3	0.00–3.45	0–0	0.00–0.00
	Apr 12 to Apr 18, 2021	88	0–1	0.00–1.15	0–0	0.00–0.00
	Apr 19 to Apr 25, 2021	93	0–6	0.00–6.90	0–0	0.00–0.00
	Apr 26 to May 2, 2021	97	0–10	0.00–11.49	0–0	0.00–0.00
	May 3 to May 9, 2021	80	0–0	0.00–0.00	0–7	0.00–8.05
	May 10 to May 16, 2021	84	0–0	0.00–0.00	0–2	0.00–2.33
	May 17 to May 23, 2021	82	0–0	0.00–0.00	0–4	0.00–4.65
	May 24 to May 30, 2021	101	0–16	0.00–18.82	0–0	0.00–0.00
Kochi						
	Apr 5 to Apr 11, 2021	41	0–0	0.00–0.00	0–8	0.00–16.33
	Apr 12 to Apr 18, 2021	55	0–6	0.00–12.24	0–0	0.00–0.00
	Apr 19 to Apr 25, 2021	61	0–12	0.00–24.49	0–0	0.00–0.00
	Apr 26 to May 2, 2021	50	0–0	0.00–0.00	0–0	0.00–0.00
	May 3 to May 9, 2021	41	0–0	0.00–0.00	0–8	0.00–16.33
	May 10 to May 16, 2021	49	0–0	0.00–0.00	0–0	0.00–0.00
	May 17 to May 23, 2021	48	0–0	0.00–0.00	0–1	0.00–2.04
	May 24 to May 30, 2021	45	0–0	0.00–0.00	0–3	0.00–6.25
Fukuoka						
	Apr 5 to Apr 11, 2021	279	0–0	0.00–0.00	0–23	0.00–7.62
	Apr 12 to Apr 18, 2021	314	0–13	0.00–4.32	0–0	0.00–0.00
	Apr 19 to Apr 25, 2021	347	14–48	4.68–16.05	0–0	0.00–0.00

	Apr 26 to May 2, 2021	337	3–37	1.00–12.33	0–0	0.00–0.00
	May 3 to May 9, 2021	302	0–5	0.00–1.68	0–0	0.00–0.00
	May 10 to May 16, 2021	317	0–22	0.00–7.46	0–0	0.00–0.00
	May 17 to May 23, 2021	314	0–17	0.00–5.72	0–0	0.00–0.00
	May 24 to May 30, 2021	300	0–4	0.00–1.35	0–0	0.00–0.00
Saga						
	Apr 5 to Apr 11, 2021	47	0–0	0.00–0.00	0–4	0.00–7.84
	Apr 12 to Apr 18, 2021	40	0–0	0.00–0.00	0–11	0.00–21.57
	Apr 19 to Apr 25, 2021	59	0–9	0.00–18.00	0–0	0.00–0.00
	Apr 26 to May 2, 2021	45	0–0	0.00–0.00	0–4	0.00–8.16
	May 3 to May 9, 2021	66	2–16	4.00–32.00	0–0	0.00–0.00
	May 10 to May 16, 2021	54	0–5	0.00–10.20	0–0	0.00–0.00
	May 17 to May 23, 2021	53	0–2	0.00–3.92	0–0	0.00–0.00
	May 24 to May 30, 2021	57	0–6	0.00–11.76	0–0	0.00–0.00
Nagasaki						
	Apr 5 to Apr 11, 2021	87	0–0	0.00–0.00	0–9	0.00–9.38
	Apr 12 to Apr 18, 2021	87	0–0	0.00–0.00	0–9	0.00–9.38
	Apr 19 to Apr 25, 2021	88	0–0	0.00–0.00	0–9	0.00–9.28
	Apr 26 to May 2, 2021	97	0–3	0.00–3.19	0–0	0.00–0.00
	May 3 to May 9, 2021	97	0–2	0.00–2.11	0–0	0.00–0.00
	May 10 to May 16, 2021	102	0–8	0.00–8.51	0–0	0.00–0.00
	May 17 to May 23, 2021	91	0–0	0.00–0.00	0–2	0.00–2.15
	May 24 to May 30, 2021	110	0–19	0.00–20.88	0–0	0.00–0.00
Kumamoto						
	Apr 5 to Apr 11, 2021	109	0–9	0.00–9.00	0–0	0.00–0.00
	Apr 12 to Apr 18, 2021	134	14–34	14.00–34.00	0–0	0.00–0.00
	Apr 19 to Apr 25, 2021	113	0–13	0.00–13.00	0–0	0.00–0.00
	Apr 26 to May 2, 2021	113	0–12	0.00–11.88	0–0	0.00–0.00
	May 3 to May 9, 2021	103	0–2	0.00–1.98	0–0	0.00–0.00
	May 10 to May 16, 2021	108	0–8	0.00–8.00	0–0	0.00–0.00
	May 17 to May 23, 2021	108	0–7	0.00–6.93	0–0	0.00–0.00
	May 24 to May 30, 2021	102	0–1	0.00–0.99	0–0	0.00–0.00
Oita						
	Apr 5 to Apr 11, 2021	57	0–0	0.00–0.00	0–12	0.00–17.39
	Apr 12 to Apr 18, 2021	84	0–16	0.00–23.53	0–0	0.00–0.00
	Apr 19 to Apr 25, 2021	69	0–2	0.00–2.99	0–0	0.00–0.00
	Apr 26 to May 2, 2021	71	0–3	0.00–4.41	0–0	0.00–0.00
	May 3 to May 9, 2021	80	0–13	0.00–19.40	0–0	0.00–0.00
	May 10 to May 16, 2021	75	0–7	0.00–10.29	0–0	0.00–0.00
	May 17 to May 23, 2021	67	0–0	0.00–0.00	0–1	0.00–1.47
	May 24 to May 30, 2021	89	6–22	8.96–32.84	0–0	0.00–0.00
Miyazaki						
	Apr 5 to Apr 11, 2021	84	0–16	0.00–23.53	0–0	0.00–0.00
	Apr 12 to Apr 18, 2021	62	0–0	0.00–0.00	0–6	0.00–8.82
	Apr 19 to Apr 25, 2021	69	0–1	0.00–1.47	0–0	0.00–0.00
	Apr 26 to May 2, 2021	63	0–0	0.00–0.00	0–5	0.00–7.35

	May 3 to May 9, 2021	75	0–6	0.00–8.70	0–0	0.00–0.00
	May 10 to May 16, 2021	74	0–6	0.00–8.82	0–0	0.00–0.00
	May 17 to May 23, 2021	79	0–10	0.00–14.49	0–0	0.00–0.00
	May 24 to May 30, 2021	62	0–0	0.00–0.00	0–6	0.00–8.82
Kagoshima						
	Apr 5 to Apr 11, 2021	108	0–11	0.00–11.34	0–0	0.00–0.00
	Apr 12 to Apr 18, 2021	99	0–2	0.00–2.06	0–0	0.00–0.00
	Apr 19 to Apr 25, 2021	89	0–0	0.00–0.00	0–6	0.00–6.32
	Apr 26 to May 2, 2021	90	0–0	0.00–0.00	0–7	0.00–7.22
	May 3 to May 9, 2021	110	0–12	0.00–12.24	0–0	0.00–0.00
	May 10 to May 16, 2021	95	0–0	0.00–0.00	0–3	0.00–3.06
	May 17 to May 23, 2021	100	0–3	0.00–3.09	0–0	0.00–0.00
	May 24 to May 30, 2021	97	0–0	0.00–0.00	0–1	0.00–1.02
Okinawa						
	Apr 5 to Apr 11, 2021	83	2–18	3.08–27.69	0–0	0.00–0.00
	Apr 12 to Apr 18, 2021	75	0–9	0.00–13.64	0–0	0.00–0.00
	Apr 19 to Apr 25, 2021	76	0–11	0.00–16.92	0–0	0.00–0.00
	Apr 26 to May 2, 2021	65	0–0	0.00–0.00	0–0	0.00–0.00
	May 3 to May 9, 2021	56	0–0	0.00–0.00	0–9	0.00–13.85
	May 10 to May 16, 2021	64	0–0	0.00–0.00	0–1	0.00–1.54
	May 17 to May 23, 2021	74	0–9	0.00–13.85	0–0	0.00–0.00
	May 24 to May 30, 2021	66	0–1	0.00–1.54	0–0	0.00–0.00

Table A.14: Weekly number of observed and excess/exiguous deaths in Japan and 47 prefectures for malignant neoplasms-related deaths in hospitals and clinics from January 2020 through May 2021.

Prefecture	Week	Observed	Excess deaths	Percent excess	Exiguous deaths	Percent exiguous
Japan						
	Apr 5 to Apr 11, 2021	5211	0–0	0.00–0.00	197–438	3.49–7.75
	Apr 12 to Apr 18, 2021	5362	0–0	0.00–0.00	40–281	0.71–4.98
	Apr 19 to Apr 25, 2021	5314	0–0	0.00–0.00	70–313	1.24–5.56
	Apr 26 to May 2, 2021	5372	0–0	0.00–0.00	0–226	0.00–4.04
	May 3 to May 9, 2021	5273	0–0	0.00–0.00	52–298	0.93–5.35
	May 10 to May 16, 2021	5264	0–0	0.00–0.00	29–277	0.52–5.00
	May 17 to May 23, 2021	5221	0–0	0.00–0.00	69–320	1.25–5.78
	May 24 to May 30, 2021	5202	0–0	0.00–0.00	67–323	1.21–5.85
Hokkaido						
	Apr 5 to Apr 11, 2021	301	0–0	0.00–0.00	0–29	0.00–8.79
	Apr 12 to Apr 18, 2021	333	0–6	0.00–1.83	0–0	0.00–0.00
	Apr 19 to Apr 25, 2021	321	0–0	0.00–0.00	0–6	0.00–1.83
	Apr 26 to May 2, 2021	318	0–0	0.00–0.00	0–7	0.00–2.15
	May 3 to May 9, 2021	314	0–0	0.00–0.00	0–10	0.00–3.09
	May 10 to May 16, 2021	324	0–0	0.00–0.00	0–0	0.00–0.00
	May 17 to May 23, 2021	362	2–38	0.62–11.73	0–0	0.00–0.00
	May 24 to May 30, 2021	310	0–0	0.00–0.00	0–16	0.00–4.91
Aomori						
	Apr 5 to Apr 11, 2021	96	0–14	0.00–17.07	0–0	0.00–0.00
	Apr 12 to Apr 18, 2021	97	0–13	0.00–15.48	0–0	0.00–0.00
	Apr 19 to Apr 25, 2021	86	0–2	0.00–2.38	0–0	0.00–0.00
	Apr 26 to May 2, 2021	87	0–2	0.00–2.35	0–0	0.00–0.00
	May 3 to May 9, 2021	79	0–0	0.00–0.00	0–5	0.00–5.95
	May 10 to May 16, 2021	85	0–3	0.00–3.66	0–0	0.00–0.00
	May 17 to May 23, 2021	88	0–4	0.00–4.76	0–0	0.00–0.00
	May 24 to May 30, 2021	60	0–0	0.00–0.00	7–24	8.33–28.57
Iwate						
	Apr 5 to Apr 11, 2021	86	0–10	0.00–13.16	0–0	0.00–0.00
	Apr 12 to Apr 18, 2021	60	0–0	0.00–0.00	0–16	0.00–21.05
	Apr 19 to Apr 25, 2021	81	0–4	0.00–5.19	0–0	0.00–0.00
	Apr 26 to May 2, 2021	79	0–2	0.00–2.60	0–0	0.00–0.00
	May 3 to May 9, 2021	72	0–0	0.00–0.00	0–4	0.00–5.26
	May 10 to May 16, 2021	72	0–0	0.00–0.00	0–4	0.00–5.26
	May 17 to May 23, 2021	71	0–0	0.00–0.00	0–6	0.00–7.79
	May 24 to May 30, 2021	71	0–0	0.00–0.00	0–4	0.00–5.33
Miyagi						
	Apr 5 to Apr 11, 2021	88	0–0	0.00–0.00	0–15	0.00–14.56
	Apr 12 to Apr 18, 2021	105	0–1	0.00–0.96	0–0	0.00–0.00
	Apr 19 to Apr 25, 2021	87	0–0	0.00–0.00	0–16	0.00–15.53
	Apr 26 to May 2, 2021	106	0–3	0.00–2.91	0–0	0.00–0.00
	May 3 to May 9, 2021	84	0–0	0.00–0.00	0–18	0.00–17.65

	May 10 to May 16, 2021	89	0–0	0.00–0.00	0–12	0.00–11.88
	May 17 to May 23, 2021	86	0–0	0.00–0.00	0–15	0.00–14.85
	May 24 to May 30, 2021	101	0–1	0.00–1.00	0–0	0.00–0.00
Akita						
	Apr 5 to Apr 11, 2021	65	0–0	0.00–0.00	0–5	0.00–7.14
	Apr 12 to Apr 18, 2021	65	0–0	0.00–0.00	0–6	0.00–8.45
	Apr 19 to Apr 25, 2021	63	0–0	0.00–0.00	0–7	0.00–10.00
	Apr 26 to May 2, 2021	76	0–6	0.00–8.57	0–0	0.00–0.00
	May 3 to May 9, 2021	69	0–0	0.00–0.00	0–1	0.00–1.43
	May 10 to May 16, 2021	63	0–0	0.00–0.00	0–6	0.00–8.70
	May 17 to May 23, 2021	87	0–19	0.00–27.94	0–0	0.00–0.00
	May 24 to May 30, 2021	72	0–2	0.00–2.86	0–0	0.00–0.00
Yamagata						
	Apr 5 to Apr 11, 2021	50	0–0	0.00–0.00	0–6	0.00–10.71
	Apr 12 to Apr 18, 2021	57	0–1	0.00–1.79	0–0	0.00–0.00
	Apr 19 to Apr 25, 2021	54	0–0	0.00–0.00	0–2	0.00–3.57
	Apr 26 to May 2, 2021	43	0–0	0.00–0.00	0–13	0.00–23.21
	May 3 to May 9, 2021	59	0–3	0.00–5.36	0–0	0.00–0.00
	May 10 to May 16, 2021	60	0–3	0.00–5.26	0–0	0.00–0.00
	May 17 to May 23, 2021	63	0–7	0.00–12.50	0–0	0.00–0.00
	May 24 to May 30, 2021	51	0–0	0.00–0.00	0–6	0.00–10.53
Fukushima						
	Apr 5 to Apr 11, 2021	85	0–0	0.00–0.00	0–17	0.00–16.67
	Apr 12 to Apr 18, 2021	102	0–0	0.00–0.00	0–0	0.00–0.00
	Apr 19 to Apr 25, 2021	85	0–0	0.00–0.00	0–14	0.00–14.14
	Apr 26 to May 2, 2021	90	0–0	0.00–0.00	0–9	0.00–9.09
	May 3 to May 9, 2021	95	0–0	0.00–0.00	0–3	0.00–3.06
	May 10 to May 16, 2021	84	0–0	0.00–0.00	0–13	0.00–13.40
	May 17 to May 23, 2021	98	0–3	0.00–3.16	0–0	0.00–0.00
	May 24 to May 30, 2021	89	0–0	0.00–0.00	0–7	0.00–7.29
Ibaraki						
	Apr 5 to Apr 11, 2021	120	0–0	0.00–0.00	0–21	0.00–14.89
	Apr 12 to Apr 18, 2021	127	0–0	0.00–0.00	0–12	0.00–8.63
	Apr 19 to Apr 25, 2021	117	0–0	0.00–0.00	0–23	0.00–16.43
	Apr 26 to May 2, 2021	147	0–7	0.00–5.00	0–0	0.00–0.00
	May 3 to May 9, 2021	145	0–4	0.00–2.84	0–0	0.00–0.00
	May 10 to May 16, 2021	141	0–0	0.00–0.00	0–0	0.00–0.00
	May 17 to May 23, 2021	121	0–0	0.00–0.00	0–19	0.00–13.57
	May 24 to May 30, 2021	123	0–0	0.00–0.00	0–15	0.00–10.87
Tochigi						
	Apr 5 to Apr 11, 2021	83	0–0	0.00–0.00	0–2	0.00–2.35
	Apr 12 to Apr 18, 2021	77	0–0	0.00–0.00	0–7	0.00–8.33
	Apr 19 to Apr 25, 2021	87	0–3	0.00–3.57	0–0	0.00–0.00
	Apr 26 to May 2, 2021	68	0–0	0.00–0.00	0–16	0.00–19.05
	May 3 to May 9, 2021	87	0–3	0.00–3.57	0–0	0.00–0.00
	May 10 to May 16, 2021	60	0–0	0.00–0.00	6–23	7.23–27.71

	May 17 to May 23, 2021	68	0–0	0.00–0.00	0–13	0.00–16.05
	May 24 to May 30, 2021	94	0–11	0.00–13.25	0–0	0.00–0.00
Gunma						
	Apr 5 to Apr 11, 2021	86	0–0	0.00–0.00	0–0	0.00–0.00
	Apr 12 to Apr 18, 2021	77	0–0	0.00–0.00	0–11	0.00–12.50
	Apr 19 to Apr 25, 2021	65	0–0	0.00–0.00	4–23	4.55–26.14
	Apr 26 to May 2, 2021	85	0–0	0.00–0.00	0–2	0.00–2.30
	May 3 to May 9, 2021	79	0–0	0.00–0.00	0–8	0.00–9.20
	May 10 to May 16, 2021	77	0–0	0.00–0.00	0–10	0.00–11.49
	May 17 to May 23, 2021	72	0–0	0.00–0.00	0–14	0.00–16.28
	May 24 to May 30, 2021	85	0–0	0.00–0.00	0–1	0.00–1.16
Saitama						
	Apr 5 to Apr 11, 2021	270	0–0	0.00–0.00	0–24	0.00–8.16
	Apr 12 to Apr 18, 2021	272	0–0	0.00–0.00	0–21	0.00–7.17
	Apr 19 to Apr 25, 2021	251	0–0	0.00–0.00	6–41	2.05–14.04
	Apr 26 to May 2, 2021	272	0–0	0.00–0.00	0–19	0.00–6.53
	May 3 to May 9, 2021	287	0–0	0.00–0.00	0–1	0.00–0.35
	May 10 to May 16, 2021	278	0–0	0.00–0.00	0–4	0.00–1.42
	May 17 to May 23, 2021	262	0–0	0.00–0.00	0–22	0.00–7.75
	May 24 to May 30, 2021	272	0–0	0.00–0.00	0–12	0.00–4.23
Chiba						
	Apr 5 to Apr 11, 2021	262	0–0	0.00–0.00	0–4	0.00–1.50
	Apr 12 to Apr 18, 2021	264	0–0	0.00–0.00	0–0	0.00–0.00
	Apr 19 to Apr 25, 2021	215	0–0	0.00–0.00	14–47	5.34–17.94
	Apr 26 to May 2, 2021	246	0–0	0.00–0.00	0–14	0.00–5.38
	May 3 to May 9, 2021	253	0–0	0.00–0.00	0–5	0.00–1.94
	May 10 to May 16, 2021	221	0–0	0.00–0.00	1–33	0.39–12.99
	May 17 to May 23, 2021	213	0–0	0.00–0.00	7–38	2.79–15.14
	May 24 to May 30, 2021	246	0–0	0.00–0.00	0–7	0.00–2.77
Tokyo						
	Apr 5 to Apr 11, 2021	379	0–0	0.00–0.00	25–76	5.49–16.70
	Apr 12 to Apr 18, 2021	432	0–0	0.00–0.00	0–21	0.00–4.64
	Apr 19 to Apr 25, 2021	451	0–3	0.00–0.67	0–0	0.00–0.00
	Apr 26 to May 2, 2021	448	0–1	0.00–0.22	0–0	0.00–0.00
	May 3 to May 9, 2021	420	0–0	0.00–0.00	0–26	0.00–5.83
	May 10 to May 16, 2021	406	0–0	0.00–0.00	0–36	0.00–8.14
	May 17 to May 23, 2021	390	0–0	0.00–0.00	3–53	0.68–11.96
	May 24 to May 30, 2021	413	0–0	0.00–0.00	0–29	0.00–6.56
Kanagawa						
	Apr 5 to Apr 11, 2021	268	0–0	0.00–0.00	0–35	0.00–11.55
	Apr 12 to Apr 18, 2021	272	0–0	0.00–0.00	0–30	0.00–9.93
	Apr 19 to Apr 25, 2021	285	0–0	0.00–0.00	0–17	0.00–5.63
	Apr 26 to May 2, 2021	276	0–0	0.00–0.00	0–23	0.00–7.69
	May 3 to May 9, 2021	260	0–0	0.00–0.00	0–37	0.00–12.46
	May 10 to May 16, 2021	263	0–0	0.00–0.00	0–35	0.00–11.74
	May 17 to May 23, 2021	260	0–0	0.00–0.00	0–36	0.00–12.16

	May 24 to May 30, 2021	267	0–0	0.00–0.00	0–26	0.00–8.87
Niigata						
	Apr 5 to Apr 11, 2021	117	0–0	0.00–0.00	0–14	0.00–10.69
	Apr 12 to Apr 18, 2021	123	0–0	0.00–0.00	0–10	0.00–7.52
	Apr 19 to Apr 25, 2021	132	0–0	0.00–0.00	0–1	0.00–0.75
	Apr 26 to May 2, 2021	123	0–0	0.00–0.00	0–9	0.00–6.82
	May 3 to May 9, 2021	98	0–0	0.00–0.00	11–33	8.40–25.19
	May 10 to May 16, 2021	121	0–0	0.00–0.00	0–10	0.00–7.63
	May 17 to May 23, 2021	117	0–0	0.00–0.00	0–15	0.00–11.36
	May 24 to May 30, 2021	128	0–0	0.00–0.00	0–4	0.00–3.03
Toyama						
	Apr 5 to Apr 11, 2021	45	0–0	0.00–0.00	0–8	0.00–15.09
	Apr 12 to Apr 18, 2021	52	0–0	0.00–0.00	0–1	0.00–1.89
	Apr 19 to Apr 25, 2021	59	0–7	0.00–13.46	0–0	0.00–0.00
	Apr 26 to May 2, 2021	58	0–5	0.00–9.43	0–0	0.00–0.00
	May 3 to May 9, 2021	56	0–3	0.00–5.66	0–0	0.00–0.00
	May 10 to May 16, 2021	47	0–0	0.00–0.00	0–5	0.00–9.62
	May 17 to May 23, 2021	39	0–0	0.00–0.00	1–14	1.89–26.42
	May 24 to May 30, 2021	52	0–0	0.00–0.00	0–2	0.00–3.70
Ishikawa						
	Apr 5 to Apr 11, 2021	50	0–0	0.00–0.00	0–4	0.00–7.41
	Apr 12 to Apr 18, 2021	59	0–5	0.00–9.26	0–0	0.00–0.00
	Apr 19 to Apr 25, 2021	53	0–0	0.00–0.00	0–1	0.00–1.85
	Apr 26 to May 2, 2021	51	0–0	0.00–0.00	0–2	0.00–3.77
	May 3 to May 9, 2021	49	0–0	0.00–0.00	0–4	0.00–7.55
	May 10 to May 16, 2021	54	0–1	0.00–1.89	0–0	0.00–0.00
	May 17 to May 23, 2021	53	0–0	0.00–0.00	0–0	0.00–0.00
	May 24 to May 30, 2021	43	0–0	0.00–0.00	0–10	0.00–18.87
Fukui						
	Apr 5 to Apr 11, 2021	35	0–0	0.00–0.00	0–1	0.00–2.78
	Apr 12 to Apr 18, 2021	42	0–6	0.00–16.67	0–0	0.00–0.00
	Apr 19 to Apr 25, 2021	34	0–0	0.00–0.00	0–2	0.00–5.56
	Apr 26 to May 2, 2021	35	0–0	0.00–0.00	0–1	0.00–2.78
	May 3 to May 9, 2021	34	0–0	0.00–0.00	0–1	0.00–2.86
	May 10 to May 16, 2021	34	0–0	0.00–0.00	0–2	0.00–5.56
	May 17 to May 23, 2021	35	0–0	0.00–0.00	0–1	0.00–2.78
	May 24 to May 30, 2021	33	0–0	0.00–0.00	0–3	0.00–8.33
Yamanashi						
	Apr 5 to Apr 11, 2021	30	0–0	0.00–0.00	0–8	0.00–21.05
	Apr 12 to Apr 18, 2021	29	0–0	0.00–0.00	0–9	0.00–23.68
	Apr 19 to Apr 25, 2021	31	0–0	0.00–0.00	0–6	0.00–16.22
	Apr 26 to May 2, 2021	34	0–0	0.00–0.00	0–3	0.00–8.11
	May 3 to May 9, 2021	43	0–6	0.00–16.22	0–0	0.00–0.00
	May 10 to May 16, 2021	43	0–6	0.00–16.22	0–0	0.00–0.00
	May 17 to May 23, 2021	39	0–1	0.00–2.63	0–0	0.00–0.00
	May 24 to May 30, 2021	36	0–0	0.00–0.00	0–1	0.00–2.70

Nagano						
	Apr 5 to Apr 11, 2021	76	0–0	0.00–0.00	0–19	0.00–20.00
	Apr 12 to Apr 18, 2021	100	0–5	0.00–5.26	0–0	0.00–0.00
	Apr 19 to Apr 25, 2021	87	0–0	0.00–0.00	0–7	0.00–7.45
	Apr 26 to May 2, 2021	93	0–1	0.00–1.09	0–0	0.00–0.00
	May 3 to May 9, 2021	73	0–0	0.00–0.00	1–19	1.09–20.65
	May 10 to May 16, 2021	92	0–0	0.00–0.00	0–0	0.00–0.00
	May 17 to May 23, 2021	92	0–0	0.00–0.00	0–0	0.00–0.00
	May 24 to May 30, 2021	70	0–0	0.00–0.00	4–23	4.30–24.73
Gifu						
	Apr 5 to Apr 11, 2021	77	0–0	0.00–0.00	0–7	0.00–8.33
	Apr 12 to Apr 18, 2021	77	0–0	0.00–0.00	0–6	0.00–7.23
	Apr 19 to Apr 25, 2021	80	0–0	0.00–0.00	0–2	0.00–2.44
	Apr 26 to May 2, 2021	63	0–0	0.00–0.00	0–18	0.00–22.22
	May 3 to May 9, 2021	90	0–8	0.00–9.76	0–0	0.00–0.00
	May 10 to May 16, 2021	83	0–1	0.00–1.22	0–0	0.00–0.00
	May 17 to May 23, 2021	86	0–3	0.00–3.61	0–0	0.00–0.00
	May 24 to May 30, 2021	82	0–0	0.00–0.00	0–1	0.00–1.20
Shizuoka						
	Apr 5 to Apr 11, 2021	163	0–8	0.00–5.16	0–0	0.00–0.00
	Apr 12 to Apr 18, 2021	137	0–0	0.00–0.00	0–17	0.00–11.04
	Apr 19 to Apr 25, 2021	140	0–0	0.00–0.00	0–13	0.00–8.50
	Apr 26 to May 2, 2021	147	0–0	0.00–0.00	0–6	0.00–3.92
	May 3 to May 9, 2021	140	0–0	0.00–0.00	0–13	0.00–8.50
	May 10 to May 16, 2021	140	0–0	0.00–0.00	0–13	0.00–8.50
	May 17 to May 23, 2021	170	0–17	0.00–11.11	0–0	0.00–0.00
	May 24 to May 30, 2021	148	0–0	0.00–0.00	0–4	0.00–2.63
Aichi						
	Apr 5 to Apr 11, 2021	254	0–0	0.00–0.00	4–38	1.37–13.01
	Apr 12 to Apr 18, 2021	268	0–0	0.00–0.00	0–25	0.00–8.53
	Apr 19 to Apr 25, 2021	250	0–0	0.00–0.00	7–41	2.41–14.09
	Apr 26 to May 2, 2021	276	0–0	0.00–0.00	0–14	0.00–4.83
	May 3 to May 9, 2021	267	0–0	0.00–0.00	0–24	0.00–8.25
	May 10 to May 16, 2021	291	0–3	0.00–1.04	0–0	0.00–0.00
	May 17 to May 23, 2021	236	0–0	0.00–0.00	17–51	5.92–17.77
	May 24 to May 30, 2021	254	0–0	0.00–0.00	0–31	0.00–10.88
Mie						
	Apr 5 to Apr 11, 2021	63	0–0	0.00–0.00	0–12	0.00–16.00
	Apr 12 to Apr 18, 2021	69	0–0	0.00–0.00	0–5	0.00–6.76
	Apr 19 to Apr 25, 2021	71	0–0	0.00–0.00	0–3	0.00–4.05
	Apr 26 to May 2, 2021	65	0–0	0.00–0.00	0–8	0.00–10.96
	May 3 to May 9, 2021	81	0–9	0.00–12.50	0–0	0.00–0.00
	May 10 to May 16, 2021	82	0–10	0.00–13.89	0–0	0.00–0.00
	May 17 to May 23, 2021	76	0–5	0.00–7.04	0–0	0.00–0.00
	May 24 to May 30, 2021	69	0–0	0.00–0.00	0–2	0.00–2.82
Shiga						

	Apr 5 to Apr 11, 2021	50	0–0	0.00–0.00	0–10	0.00–16.67
	Apr 12 to Apr 18, 2021	54	0–0	0.00–0.00	0–6	0.00–10.00
	Apr 19 to Apr 25, 2021	47	0–0	0.00–0.00	0–13	0.00–21.67
	Apr 26 to May 2, 2021	61	0–2	0.00–3.39	0–0	0.00–0.00
	May 3 to May 9, 2021	40	0–0	0.00–0.00	4–19	6.78–32.20
	May 10 to May 16, 2021	45	0–0	0.00–0.00	0–12	0.00–21.05
	May 17 to May 23, 2021	61	0–2	0.00–3.39	0–0	0.00–0.00
	May 24 to May 30, 2021	54	0–0	0.00–0.00	0–5	0.00–8.47
Kyoto						
	Apr 5 to Apr 11, 2021	93	0–0	0.00–0.00	5–26	4.20–21.85
	Apr 12 to Apr 18, 2021	132	0–14	0.00–11.86	0–0	0.00–0.00
	Apr 19 to Apr 25, 2021	117	0–0	0.00–0.00	0–0	0.00–0.00
	Apr 26 to May 2, 2021	121	0–4	0.00–3.42	0–0	0.00–0.00
	May 3 to May 9, 2021	108	0–0	0.00–0.00	0–9	0.00–7.69
	May 10 to May 16, 2021	111	0–0	0.00–0.00	0–6	0.00–5.13
	May 17 to May 23, 2021	92	0–0	0.00–0.00	5–26	4.24–22.03
	May 24 to May 30, 2021	107	0–0	0.00–0.00	0–10	0.00–8.55
Osaka						
	Apr 5 to Apr 11, 2021	394	0–0	0.00–0.00	0–1	0.00–0.25
	Apr 12 to Apr 18, 2021	355	0–0	0.00–0.00	0–38	0.00–9.67
	Apr 19 to Apr 25, 2021	385	0–0	0.00–0.00	0–9	0.00–2.28
	Apr 26 to May 2, 2021	376	0–0	0.00–0.00	0–15	0.00–3.84
	May 3 to May 9, 2021	373	0–0	0.00–0.00	0–17	0.00–4.36
	May 10 to May 16, 2021	355	0–0	0.00–0.00	0–34	0.00–8.74
	May 17 to May 23, 2021	357	0–0	0.00–0.00	0–30	0.00–7.75
	May 24 to May 30, 2021	340	0–0	0.00–0.00	5–47	1.29–12.14
Hyogo						
	Apr 5 to Apr 11, 2021	232	0–0	0.00–0.00	0–7	0.00–2.93
	Apr 12 to Apr 18, 2021	202	0–0	0.00–0.00	3–32	1.28–13.68
	Apr 19 to Apr 25, 2021	209	0–0	0.00–0.00	0–24	0.00–10.30
	Apr 26 to May 2, 2021	239	0–9	0.00–3.91	0–0	0.00–0.00
	May 3 to May 9, 2021	206	0–0	0.00–0.00	0–23	0.00–10.04
	May 10 to May 16, 2021	194	0–0	0.00–0.00	6–35	2.62–15.28
	May 17 to May 23, 2021	223	0–0	0.00–0.00	0–7	0.00–3.04
	May 24 to May 30, 2021	207	0–0	0.00–0.00	0–23	0.00–10.00
Nara						
	Apr 5 to Apr 11, 2021	59	0–0	0.00–0.00	0–2	0.00–3.28
	Apr 12 to Apr 18, 2021	56	0–0	0.00–0.00	0–5	0.00–8.20
	Apr 19 to Apr 25, 2021	66	0–5	0.00–8.20	0–0	0.00–0.00
	Apr 26 to May 2, 2021	48	0–0	0.00–0.00	0–14	0.00–22.58
	May 3 to May 9, 2021	69	0–6	0.00–9.52	0–0	0.00–0.00
	May 10 to May 16, 2021	67	0–4	0.00–6.35	0–0	0.00–0.00
	May 17 to May 23, 2021	50	0–0	0.00–0.00	0–12	0.00–19.35
	May 24 to May 30, 2021	60	0–0	0.00–0.00	0–3	0.00–4.76
Wakayama						
	Apr 5 to Apr 11, 2021	44	0–0	0.00–0.00	0–3	0.00–6.38

	Apr 12 to Apr 18, 2021	45	0–0	0.00–0.00	0–2	0.00–4.26
	Apr 19 to Apr 25, 2021	40	0–0	0.00–0.00	0–8	0.00–16.67
	Apr 26 to May 2, 2021	45	0–0	0.00–0.00	0–2	0.00–4.26
	May 3 to May 9, 2021	44	0–0	0.00–0.00	0–2	0.00–4.35
	May 10 to May 16, 2021	49	0–3	0.00–6.52	0–0	0.00–0.00
	May 17 to May 23, 2021	46	0–1	0.00–2.22	0–0	0.00–0.00
	May 24 to May 30, 2021	50	0–4	0.00–8.70	0–0	0.00–0.00
Tottori						
	Apr 5 to Apr 11, 2021	33	0–4	0.00–13.79	0–0	0.00–0.00
	Apr 12 to Apr 18, 2021	25	0–0	0.00–0.00	0–4	0.00–13.79
	Apr 19 to Apr 25, 2021	26	0–0	0.00–0.00	0–2	0.00–7.14
	Apr 26 to May 2, 2021	30	0–2	0.00–7.14	0–0	0.00–0.00
	May 3 to May 9, 2021	33	0–5	0.00–17.86	0–0	0.00–0.00
	May 10 to May 16, 2021	31	0–2	0.00–6.90	0–0	0.00–0.00
	May 17 to May 23, 2021	35	0–6	0.00–20.69	0–0	0.00–0.00
	May 24 to May 30, 2021	29	0–0	0.00–0.00	0–0	0.00–0.00
Shimane						
	Apr 5 to Apr 11, 2021	30	0–0	0.00–0.00	0–8	0.00–21.05
	Apr 12 to Apr 18, 2021	39	0–1	0.00–2.63	0–0	0.00–0.00
	Apr 19 to Apr 25, 2021	37	0–0	0.00–0.00	0–1	0.00–2.63
	Apr 26 to May 2, 2021	45	0–8	0.00–21.62	0–0	0.00–0.00
	May 3 to May 9, 2021	45	0–8	0.00–21.62	0–0	0.00–0.00
	May 10 to May 16, 2021	39	0–2	0.00–5.41	0–0	0.00–0.00
	May 17 to May 23, 2021	30	0–0	0.00–0.00	0–7	0.00–18.92
	May 24 to May 30, 2021	42	0–5	0.00–13.51	0–0	0.00–0.00
Okayama						
	Apr 5 to Apr 11, 2021	93	0–3	0.00–3.33	0–0	0.00–0.00
	Apr 12 to Apr 18, 2021	87	0–0	0.00–0.00	0–1	0.00–1.14
	Apr 19 to Apr 25, 2021	84	0–0	0.00–0.00	0–4	0.00–4.55
	Apr 26 to May 2, 2021	74	0–0	0.00–0.00	0–15	0.00–16.85
	May 3 to May 9, 2021	77	0–0	0.00–0.00	0–14	0.00–15.38
	May 10 to May 16, 2021	90	0–1	0.00–1.12	0–0	0.00–0.00
	May 17 to May 23, 2021	81	0–0	0.00–0.00	0–6	0.00–6.90
	May 24 to May 30, 2021	76	0–0	0.00–0.00	0–12	0.00–13.64
Hiroshima						
	Apr 5 to Apr 11, 2021	130	0–1	0.00–0.78	0–0	0.00–0.00
	Apr 12 to Apr 18, 2021	129	0–0	0.00–0.00	0–1	0.00–0.77
	Apr 19 to Apr 25, 2021	127	0–0	0.00–0.00	0–3	0.00–2.31
	Apr 26 to May 2, 2021	117	0–0	0.00–0.00	0–13	0.00–10.00
	May 3 to May 9, 2021	120	0–0	0.00–0.00	0–7	0.00–5.51
	May 10 to May 16, 2021	133	0–7	0.00–5.56	0–0	0.00–0.00
	May 17 to May 23, 2021	121	0–0	0.00–0.00	0–6	0.00–4.72
	May 24 to May 30, 2021	124	0–0	0.00–0.00	0–3	0.00–2.36
Yamaguchi						
	Apr 5 to Apr 11, 2021	58	0–0	0.00–0.00	3–21	3.80–26.58
	Apr 12 to Apr 18, 2021	75	0–0	0.00–0.00	0–4	0.00–5.06

	Apr 19 to Apr 25, 2021	87	0–8	0.00–10.13	0–0	0.00–0.00
	Apr 26 to May 2, 2021	78	0–0	0.00–0.00	0–2	0.00–2.50
	May 3 to May 9, 2021	79	0–1	0.00–1.28	0–0	0.00–0.00
	May 10 to May 16, 2021	73	0–0	0.00–0.00	0–5	0.00–6.41
	May 17 to May 23, 2021	71	0–0	0.00–0.00	0–8	0.00–10.13
	May 24 to May 30, 2021	84	0–6	0.00–7.69	0–0	0.00–0.00
Tokushima						
	Apr 5 to Apr 11, 2021	46	0–9	0.00–24.32	0–0	0.00–0.00
	Apr 12 to Apr 18, 2021	23	0–0	0.00–0.00	2–14	5.41–37.84
	Apr 19 to Apr 25, 2021	47	0–9	0.00–23.68	0–0	0.00–0.00
	Apr 26 to May 2, 2021	32	0–0	0.00–0.00	0–5	0.00–13.51
	May 3 to May 9, 2021	21	0–0	0.00–0.00	5–17	13.16–44.74
	May 10 to May 16, 2021	44	0–6	0.00–15.79	0–0	0.00–0.00
	May 17 to May 23, 2021	29	0–0	0.00–0.00	0–10	0.00–25.64
	May 24 to May 30, 2021	33	0–0	0.00–0.00	0–6	0.00–15.38
Kagawa						
	Apr 5 to Apr 11, 2021	39	0–0	0.00–0.00	0–9	0.00–18.75
	Apr 12 to Apr 18, 2021	50	0–2	0.00–4.17	0–0	0.00–0.00
	Apr 19 to Apr 25, 2021	44	0–0	0.00–0.00	0–3	0.00–6.38
	Apr 26 to May 2, 2021	38	0–0	0.00–0.00	0–8	0.00–17.39
	May 3 to May 9, 2021	46	0–1	0.00–2.22	0–0	0.00–0.00
	May 10 to May 16, 2021	36	0–0	0.00–0.00	0–8	0.00–18.18
	May 17 to May 23, 2021	50	0–6	0.00–13.64	0–0	0.00–0.00
	May 24 to May 30, 2021	38	0–0	0.00–0.00	0–5	0.00–11.63
Ehime						
	Apr 5 to Apr 11, 2021	62	0–0	0.00–0.00	0–6	0.00–8.82
	Apr 12 to Apr 18, 2021	66	0–0	0.00–0.00	0–2	0.00–2.94
	Apr 19 to Apr 25, 2021	65	0–0	0.00–0.00	0–3	0.00–4.41
	Apr 26 to May 2, 2021	75	0–7	0.00–10.29	0–0	0.00–0.00
	May 3 to May 9, 2021	55	0–0	0.00–0.00	0–13	0.00–19.12
	May 10 to May 16, 2021	64	0–0	0.00–0.00	0–3	0.00–4.48
	May 17 to May 23, 2021	64	0–0	0.00–0.00	0–3	0.00–4.48
	May 24 to May 30, 2021	75	0–8	0.00–11.94	0–0	0.00–0.00
Kochi						
	Apr 5 to Apr 11, 2021	34	0–0	0.00–0.00	0–9	0.00–20.93
	Apr 12 to Apr 18, 2021	34	0–0	0.00–0.00	0–8	0.00–19.05
	Apr 19 to Apr 25, 2021	51	0–9	0.00–21.43	0–0	0.00–0.00
	Apr 26 to May 2, 2021	41	0–0	0.00–0.00	0–2	0.00–4.65
	May 3 to May 9, 2021	37	0–0	0.00–0.00	0–5	0.00–11.90
	May 10 to May 16, 2021	38	0–0	0.00–0.00	0–4	0.00–9.52
	May 17 to May 23, 2021	37	0–0	0.00–0.00	0–6	0.00–13.95
	May 24 to May 30, 2021	37	0–0	0.00–0.00	0–4	0.00–9.76
Fukuoka						
	Apr 5 to Apr 11, 2021	207	0–0	0.00–0.00	14–45	5.56–17.86
	Apr 12 to Apr 18, 2021	240	0–0	0.00–0.00	0–9	0.00–3.61
	Apr 19 to Apr 25, 2021	270	0–21	0.00–8.43	0–0	0.00–0.00

	Apr 26 to May 2, 2021	253	0–5	0.00–2.02	0–0	0.00–0.00
	May 3 to May 9, 2021	238	0–0	0.00–0.00	0–8	0.00–3.25
	May 10 to May 16, 2021	249	0–5	0.00–2.05	0–0	0.00–0.00
	May 17 to May 23, 2021	240	0–0	0.00–0.00	0–5	0.00–2.04
	May 24 to May 30, 2021	225	0–0	0.00–0.00	0–19	0.00–7.79
Saga						
	Apr 5 to Apr 11, 2021	40	0–0	0.00–0.00	0–3	0.00–6.98
	Apr 12 to Apr 18, 2021	26	0–0	0.00–0.00	4–16	9.52–38.10
	Apr 19 to Apr 25, 2021	44	0–2	0.00–4.76	0–0	0.00–0.00
	Apr 26 to May 2, 2021	34	0–0	0.00–0.00	0–7	0.00–17.07
	May 3 to May 9, 2021	47	0–6	0.00–14.63	0–0	0.00–0.00
	May 10 to May 16, 2021	48	0–7	0.00–17.07	0–0	0.00–0.00
	May 17 to May 23, 2021	35	0–0	0.00–0.00	0–7	0.00–16.67
	May 24 to May 30, 2021	40	0–0	0.00–0.00	0–3	0.00–6.98
Nagasaki						
	Apr 5 to Apr 11, 2021	65	0–0	0.00–0.00	0–15	0.00–18.75
	Apr 12 to Apr 18, 2021	67	0–0	0.00–0.00	0–13	0.00–16.25
	Apr 19 to Apr 25, 2021	64	0–0	0.00–0.00	0–16	0.00–20.00
	Apr 26 to May 2, 2021	78	0–0	0.00–0.00	0–0	0.00–0.00
	May 3 to May 9, 2021	80	0–0	0.00–0.00	0–0	0.00–0.00
	May 10 to May 16, 2021	74	0–0	0.00–0.00	0–4	0.00–5.13
	May 17 to May 23, 2021	74	0–0	0.00–0.00	0–3	0.00–3.90
	May 24 to May 30, 2021	80	0–4	0.00–5.26	0–0	0.00–0.00
Kumamoto						
	Apr 5 to Apr 11, 2021	86	0–0	0.00–0.00	0–0	0.00–0.00
	Apr 12 to Apr 18, 2021	109	4–23	4.65–26.74	0–0	0.00–0.00
	Apr 19 to Apr 25, 2021	88	0–2	0.00–2.33	0–0	0.00–0.00
	Apr 26 to May 2, 2021	87	0–1	0.00–1.16	0–0	0.00–0.00
	May 3 to May 9, 2021	81	0–0	0.00–0.00	0–5	0.00–5.81
	May 10 to May 16, 2021	82	0–0	0.00–0.00	0–4	0.00–4.65
	May 17 to May 23, 2021	92	0–5	0.00–5.75	0–0	0.00–0.00
	May 24 to May 30, 2021	82	0–0	0.00–0.00	0–3	0.00–3.53
Oita						
	Apr 5 to Apr 11, 2021	45	0–0	0.00–0.00	0–12	0.00–21.05
	Apr 12 to Apr 18, 2021	63	0–7	0.00–12.50	0–0	0.00–0.00
	Apr 19 to Apr 25, 2021	57	0–1	0.00–1.79	0–0	0.00–0.00
	Apr 26 to May 2, 2021	49	0–0	0.00–0.00	0–7	0.00–12.50
	May 3 to May 9, 2021	51	0–0	0.00–0.00	0–4	0.00–7.27
	May 10 to May 16, 2021	58	0–2	0.00–3.57	0–0	0.00–0.00
	May 17 to May 23, 2021	56	0–0	0.00–0.00	0–0	0.00–0.00
	May 24 to May 30, 2021	73	4–19	7.41–35.19	0–0	0.00–0.00
Miyazaki						
	Apr 5 to Apr 11, 2021	75	2–17	3.45–29.31	0–0	0.00–0.00
	Apr 12 to Apr 18, 2021	53	0–0	0.00–0.00	0–5	0.00–8.62
	Apr 19 to Apr 25, 2021	58	0–0	0.00–0.00	0–0	0.00–0.00
	Apr 26 to May 2, 2021	50	0–0	0.00–0.00	0–8	0.00–13.79

	May 3 to May 9, 2021	58	0–0	0.00–0.00	0–0	0.00–0.00
	May 10 to May 16, 2021	57	0–0	0.00–0.00	0–1	0.00–1.72
	May 17 to May 23, 2021	63	0–5	0.00–8.62	0–0	0.00–0.00
	May 24 to May 30, 2021	53	0–0	0.00–0.00	0–4	0.00–7.02
Kagoshima						
	Apr 5 to Apr 11, 2021	87	0–6	0.00–7.41	0–0	0.00–0.00
	Apr 12 to Apr 18, 2021	80	0–0	0.00–0.00	0–1	0.00–1.23
	Apr 19 to Apr 25, 2021	61	0–0	0.00–0.00	1–19	1.25–23.75
	Apr 26 to May 2, 2021	71	0–0	0.00–0.00	0–10	0.00–12.35
	May 3 to May 9, 2021	86	0–5	0.00–6.17	0–0	0.00–0.00
	May 10 to May 16, 2021	71	0–0	0.00–0.00	0–8	0.00–10.13
	May 17 to May 23, 2021	77	0–0	0.00–0.00	0–1	0.00–1.28
	May 24 to May 30, 2021	75	0–0	0.00–0.00	0–4	0.00–5.06
Okinawa						
	Apr 5 to Apr 11, 2021	60	0–8	0.00–15.38	0–0	0.00–0.00
	Apr 12 to Apr 18, 2021	56	0–3	0.00–5.66	0–0	0.00–0.00
	Apr 19 to Apr 25, 2021	55	0–3	0.00–5.77	0–0	0.00–0.00
	Apr 26 to May 2, 2021	44	0–0	0.00–0.00	0–8	0.00–15.38
	May 3 to May 9, 2021	45	0–0	0.00–0.00	0–8	0.00–15.09
	May 10 to May 16, 2021	46	0–0	0.00–0.00	0–6	0.00–11.54
	May 17 to May 23, 2021	53	0–0	0.00–0.00	0–0	0.00–0.00
	May 24 to May 30, 2021	49	0–0	0.00–0.00	0–3	0.00–5.77

Table A.15: Weekly number of observed and excess/exiguous deaths in Japan and 47 prefectures for malignant neoplasms-related deaths in nursing homes and elderly care facilities from January 2020 through May 2021.

Prefecture	Week	Observed	Excess deaths	Percent excess	Exiguous deaths	Percent exiguous
Japan						
	Apr 5 to Apr 11, 2021	403	0–35	0.00–9.51	0–0	0.00–0.00
	Apr 12 to Apr 18, 2021	433	29–68	7.95–18.63	0–0	0.00–0.00
	Apr 19 to Apr 25, 2021	426	23–63	6.34–17.36	0–0	0.00–0.00
	Apr 26 to May 2, 2021	465	62–101	17.03–27.75	0–0	0.00–0.00
	May 3 to May 9, 2021	491	88–127	24.18–34.89	0–0	0.00–0.00
	May 10 to May 16, 2021	428	21–60	5.71–16.30	0–0	0.00–0.00
	May 17 to May 23, 2021	463	58–97	15.85–26.50	0–0	0.00–0.00
	May 24 to May 30, 2021	473	69–108	18.90–29.59	0–0	0.00–0.00
Hokkaido						
	Apr 5 to Apr 11, 2021	9	0–0	0.00–0.00	0–0	0.00–0.00
	Apr 12 to Apr 18, 2021	17	2–8	22.22–88.89	0–0	0.00–0.00
	Apr 19 to Apr 25, 2021	8	0–0	0.00–0.00	0–1	0.00–11.11
	Apr 26 to May 2, 2021	12	0–2	0.00–20.00	0–0	0.00–0.00
	May 3 to May 9, 2021	23	6–13	60.00–130.00	0–0	0.00–0.00
	May 10 to May 16, 2021	7	0–0	0.00–0.00	0–3	0.00–30.00
	May 17 to May 23, 2021	8	0–0	0.00–0.00	0–2	0.00–20.00
	May 24 to May 30, 2021	8	0–0	0.00–0.00	0–2	0.00–20.00
Aomori						
	Apr 5 to Apr 11, 2021	10	0–5	0.00–100.00	0–0	0.00–0.00
	Apr 12 to Apr 18, 2021	4	0–0	0.00–0.00	0–1	0.00–20.00
	Apr 19 to Apr 25, 2021	6	0–1	0.00–20.00	0–0	0.00–0.00
	Apr 26 to May 2, 2021	7	0–2	0.00–40.00	0–0	0.00–0.00
	May 3 to May 9, 2021	9	0–4	0.00–80.00	0–0	0.00–0.00
	May 10 to May 16, 2021	6	0–1	0.00–20.00	0–0	0.00–0.00
	May 17 to May 23, 2021	11	1–6	20.00–120.00	0–0	0.00–0.00
	May 24 to May 30, 2021	8	0–3	0.00–60.00	0–0	0.00–0.00
Iwate						
	Apr 5 to Apr 11, 2021	4	0–0	0.00–0.00	0–1	0.00–20.00
	Apr 12 to Apr 18, 2021	6	0–1	0.00–20.00	0–0	0.00–0.00
	Apr 19 to Apr 25, 2021	4	0–0	0.00–0.00	0–0	0.00–0.00
	Apr 26 to May 2, 2021	10	1–6	25.00–150.00	0–0	0.00–0.00
	May 3 to May 9, 2021	6	0–1	0.00–20.00	0–0	0.00–0.00
	May 10 to May 16, 2021	4	0–0	0.00–0.00	0–1	0.00–20.00
	May 17 to May 23, 2021	5	0–0	0.00–0.00	0–0	0.00–0.00
	May 24 to May 30, 2021	7	0–2	0.00–40.00	0–0	0.00–0.00
Miyagi						
	Apr 5 to Apr 11, 2021	9	0–3	0.00–50.00	0–0	0.00–0.00
	Apr 12 to Apr 18, 2021	10	0–4	0.00–66.67	0–0	0.00–0.00
	Apr 19 to Apr 25, 2021	9	0–2	0.00–28.57	0–0	0.00–0.00
	Apr 26 to May 2, 2021	8	0–1	0.00–14.29	0–0	0.00–0.00
	May 3 to May 9, 2021	13	0–6	0.00–85.71	0–0	0.00–0.00

	May 10 to May 16, 2021	11	0–4	0.00–57.14	0–0	0.00–0.00
	May 17 to May 23, 2021	4	0–0	0.00–0.00	0–3	0.00–42.86
	May 24 to May 30, 2021	8	0–1	0.00–14.29	0–0	0.00–0.00
Akita						
	Apr 5 to Apr 11, 2021	3	0–0	0.00–0.00	0–0	0.00–0.00
	Apr 12 to Apr 18, 2021	4	0–1	0.00–33.33	0–0	0.00–0.00
	Apr 19 to Apr 25, 2021	3	0–0	0.00–0.00	0–0	0.00–0.00
	Apr 26 to May 2, 2021	3	0–0	0.00–0.00	0–0	0.00–0.00
	May 3 to May 9, 2021	6	0–3	0.00–100.00	0–0	0.00–0.00
	May 10 to May 16, 2021	0	0–0	0.00–0.00	0–3	0.00–100.00
	May 17 to May 23, 2021	4	0–1	0.00–33.33	0–0	0.00–0.00
	May 24 to May 30, 2021	6	0–3	0.00–100.00	0–0	0.00–0.00
Yamagata						
	Apr 5 to Apr 11, 2021	6	0–0	0.00–0.00	0–0	0.00–0.00
	Apr 12 to Apr 18, 2021	5	0–0	0.00–0.00	0–1	0.00–16.67
	Apr 19 to Apr 25, 2021	2	0–0	0.00–0.00	0–3	0.00–60.00
	Apr 26 to May 2, 2021	6	0–1	0.00–20.00	0–0	0.00–0.00
	May 3 to May 9, 2021	10	0–5	0.00–100.00	0–0	0.00–0.00
	May 10 to May 16, 2021	10	1–5	20.00–100.00	0–0	0.00–0.00
	May 17 to May 23, 2021	9	0–4	0.00–80.00	0–0	0.00–0.00
	May 24 to May 30, 2021	6	0–2	0.00–50.00	0–0	0.00–0.00
Fukushima						
	Apr 5 to Apr 11, 2021	4	0–0	0.00–0.00	0–1	0.00–20.00
	Apr 12 to Apr 18, 2021	2	0–0	0.00–0.00	0–3	0.00–60.00
	Apr 19 to Apr 25, 2021	5	0–0	0.00–0.00	0–0	0.00–0.00
	Apr 26 to May 2, 2021	6	0–2	0.00–50.00	0–0	0.00–0.00
	May 3 to May 9, 2021	9	0–4	0.00–80.00	0–0	0.00–0.00
	May 10 to May 16, 2021	4	0–0	0.00–0.00	0–1	0.00–20.00
	May 17 to May 23, 2021	2	0–0	0.00–0.00	0–3	0.00–60.00
	May 24 to May 30, 2021	7	0–2	0.00–40.00	0–0	0.00–0.00
Ibaraki						
	Apr 5 to Apr 11, 2021	8	0–1	0.00–14.29	0–0	0.00–0.00
	Apr 12 to Apr 18, 2021	7	0–0	0.00–0.00	0–0	0.00–0.00
	Apr 19 to Apr 25, 2021	1	0–0	0.00–0.00	1–6	14.29–85.71
	Apr 26 to May 2, 2021	5	0–0	0.00–0.00	0–2	0.00–28.57
	May 3 to May 9, 2021	10	0–3	0.00–42.86	0–0	0.00–0.00
	May 10 to May 16, 2021	10	0–3	0.00–42.86	0–0	0.00–0.00
	May 17 to May 23, 2021	10	0–3	0.00–42.86	0–0	0.00–0.00
	May 24 to May 30, 2021	3	0–0	0.00–0.00	0–4	0.00–57.14
Tochigi						
	Apr 5 to Apr 11, 2021	2	0–0	0.00–0.00	1–5	14.29–71.43
	Apr 12 to Apr 18, 2021	7	0–0	0.00–0.00	0–0	0.00–0.00
	Apr 19 to Apr 25, 2021	7	0–0	0.00–0.00	0–0	0.00–0.00
	Apr 26 to May 2, 2021	5	0–0	0.00–0.00	0–2	0.00–28.57
	May 3 to May 9, 2021	7	0–0	0.00–0.00	0–0	0.00–0.00
	May 10 to May 16, 2021	9	0–2	0.00–28.57	0–0	0.00–0.00

	May 17 to May 23, 2021	8	0–1	0.00–14.29	0–0	0.00–0.00
	May 24 to May 30, 2021	5	0–0	0.00–0.00	0–1	0.00–16.67
Gunma						
	Apr 5 to Apr 11, 2021	13	0–4	0.00–44.44	0–0	0.00–0.00
	Apr 12 to Apr 18, 2021	18	2–8	20.00–80.00	0–0	0.00–0.00
	Apr 19 to Apr 25, 2021	10	0–0	0.00–0.00	0–0	0.00–0.00
	Apr 26 to May 2, 2021	9	0–0	0.00–0.00	0–1	0.00–10.00
	May 3 to May 9, 2021	13	0–3	0.00–30.00	0–0	0.00–0.00
	May 10 to May 16, 2021	8	0–0	0.00–0.00	0–2	0.00–20.00
	May 17 to May 23, 2021	6	0–0	0.00–0.00	0–3	0.00–33.33
	May 24 to May 30, 2021	10	0–1	0.00–11.11	0–0	0.00–0.00
Saitama						
	Apr 5 to Apr 11, 2021	30	0–7	0.00–30.43	0–0	0.00–0.00
	Apr 12 to Apr 18, 2021	24	0–1	0.00–4.35	0–0	0.00–0.00
	Apr 19 to Apr 25, 2021	20	0–0	0.00–0.00	0–2	0.00–9.09
	Apr 26 to May 2, 2021	37	4–14	17.39–60.87	0–0	0.00–0.00
	May 3 to May 9, 2021	25	0–2	0.00–8.70	0–0	0.00–0.00
	May 10 to May 16, 2021	26	0–2	0.00–8.33	0–0	0.00–0.00
	May 17 to May 23, 2021	37	1–12	4.00–48.00	0–0	0.00–0.00
	May 24 to May 30, 2021	31	0–7	0.00–29.17	0–0	0.00–0.00
Chiba						
	Apr 5 to Apr 11, 2021	14	0–0	0.00–0.00	0–0	0.00–0.00
	Apr 12 to Apr 18, 2021	12	0–0	0.00–0.00	0–2	0.00–14.29
	Apr 19 to Apr 25, 2021	23	0–8	0.00–53.33	0–0	0.00–0.00
	Apr 26 to May 2, 2021	17	0–2	0.00–13.33	0–0	0.00–0.00
	May 3 to May 9, 2021	14	0–0	0.00–0.00	0–1	0.00–6.67
	May 10 to May 16, 2021	14	0–0	0.00–0.00	0–0	0.00–0.00
	May 17 to May 23, 2021	16	0–1	0.00–6.67	0–0	0.00–0.00
	May 24 to May 30, 2021	14	0–0	0.00–0.00	0–1	0.00–6.67
Tokyo						
	Apr 5 to Apr 11, 2021	28	0–0	0.00–0.00	0–8	0.00–22.22
	Apr 12 to Apr 18, 2021	36	0–1	0.00–2.86	0–0	0.00–0.00
	Apr 19 to Apr 25, 2021	45	0–10	0.00–28.57	0–0	0.00–0.00
	Apr 26 to May 2, 2021	40	0–5	0.00–14.29	0–0	0.00–0.00
	May 3 to May 9, 2021	48	0–13	0.00–37.14	0–0	0.00–0.00
	May 10 to May 16, 2021	34	0–0	0.00–0.00	0–1	0.00–2.86
	May 17 to May 23, 2021	37	0–2	0.00–5.71	0–0	0.00–0.00
	May 24 to May 30, 2021	42	0–6	0.00–16.67	0–0	0.00–0.00
Kanagawa						
	Apr 5 to Apr 11, 2021	48	0–3	0.00–6.67	0–0	0.00–0.00
	Apr 12 to Apr 18, 2021	43	0–0	0.00–0.00	0–1	0.00–2.27
	Apr 19 to Apr 25, 2021	50	0–6	0.00–13.64	0–0	0.00–0.00
	Apr 26 to May 2, 2021	65	7–21	15.91–47.73	0–0	0.00–0.00
	May 3 to May 9, 2021	60	2–16	4.55–36.36	0–0	0.00–0.00
	May 10 to May 16, 2021	42	0–0	0.00–0.00	0–3	0.00–6.67
	May 17 to May 23, 2021	57	0–13	0.00–29.55	0–0	0.00–0.00

	May 24 to May 30, 2021	68	9–23	20.00–51.11	0–0	0.00–0.00
Niigata						
	Apr 5 to Apr 11, 2021	9	0–2	0.00–28.57	0–0	0.00–0.00
	Apr 12 to Apr 18, 2021	7	0–0	0.00–0.00	0–0	0.00–0.00
	Apr 19 to Apr 25, 2021	8	0–0	0.00–0.00	0–0	0.00–0.00
	Apr 26 to May 2, 2021	5	0–0	0.00–0.00	0–3	0.00–37.50
	May 3 to May 9, 2021	9	0–2	0.00–28.57	0–0	0.00–0.00
	May 10 to May 16, 2021	11	0–4	0.00–57.14	0–0	0.00–0.00
	May 17 to May 23, 2021	4	0–0	0.00–0.00	0–3	0.00–42.86
	May 24 to May 30, 2021	8	0–1	0.00–14.29	0–0	0.00–0.00
Toyama						
	Apr 5 to Apr 11, 2021	6	0–2	0.00–50.00	0–0	0.00–0.00
	Apr 12 to Apr 18, 2021	5	0–1	0.00–25.00	0–0	0.00–0.00
	Apr 19 to Apr 25, 2021	4	0–0	0.00–0.00	0–1	0.00–20.00
	Apr 26 to May 2, 2021	3	0–0	0.00–0.00	0–1	0.00–25.00
	May 3 to May 9, 2021	5	0–1	0.00–25.00	0–0	0.00–0.00
	May 10 to May 16, 2021	6	0–2	0.00–50.00	0–0	0.00–0.00
	May 17 to May 23, 2021	4	0–0	0.00–0.00	0–0	0.00–0.00
	May 24 to May 30, 2021	4	0–0	0.00–0.00	0–0	0.00–0.00
Ishikawa						
	Apr 5 to Apr 11, 2021	11	0–6	0.00–120.00	0–0	0.00–0.00
	Apr 12 to Apr 18, 2021	3	0–0	0.00–0.00	0–2	0.00–40.00
	Apr 19 to Apr 25, 2021	7	0–2	0.00–40.00	0–0	0.00–0.00
	Apr 26 to May 2, 2021	4	0–0	0.00–0.00	0–1	0.00–20.00
	May 3 to May 9, 2021	6	0–1	0.00–20.00	0–0	0.00–0.00
	May 10 to May 16, 2021	1	0–0	0.00–0.00	1–5	16.67–83.33
	May 17 to May 23, 2021	4	0–0	0.00–0.00	0–2	0.00–33.33
	May 24 to May 30, 2021	4	0–0	0.00–0.00	0–2	0.00–33.33
Fukui						
	Apr 5 to Apr 11, 2021	2	0–0	0.00–0.00	0–0	0.00–0.00
	Apr 12 to Apr 18, 2021	1	0–0	0.00–0.00	0–1	0.00–50.00
	Apr 19 to Apr 25, 2021	5	0–3	0.00–150.00	0–0	0.00–0.00
	Apr 26 to May 2, 2021	4	0–2	0.00–100.00	0–0	0.00–0.00
	May 3 to May 9, 2021	8	3–6	150.00–300.00	0–0	0.00–0.00
	May 10 to May 16, 2021	0	0–0	0.00–0.00	0–2	0.00–100.00
	May 17 to May 23, 2021	6	1–4	50.00–200.00	0–0	0.00–0.00
	May 24 to May 30, 2021	2	0–0	0.00–0.00	0–0	0.00–0.00
Yamanashi						
	Apr 5 to Apr 11, 2021	3	0–1	0.00–50.00	0–0	0.00–0.00
	Apr 12 to Apr 18, 2021	1	0–0	0.00–0.00	0–1	0.00–50.00
	Apr 19 to Apr 25, 2021	4	0–1	0.00–33.33	0–0	0.00–0.00
	Apr 26 to May 2, 2021	4	0–1	0.00–33.33	0–0	0.00–0.00
	May 3 to May 9, 2021	2	0–0	0.00–0.00	0–1	0.00–33.33
	May 10 to May 16, 2021	2	0–0	0.00–0.00	0–1	0.00–33.33
	May 17 to May 23, 2021	5	0–3	0.00–150.00	0–0	0.00–0.00
	May 24 to May 30, 2021	5	0–3	0.00–150.00	0–0	0.00–0.00

Nagano						
	Apr 5 to Apr 11, 2021	4	0–0	0.00–0.00	0–3	0.00–42.86
	Apr 12 to Apr 18, 2021	5	0–0	0.00–0.00	0–3	0.00–37.50
	Apr 19 to Apr 25, 2021	7	0–0	0.00–0.00	0–1	0.00–12.50
	Apr 26 to May 2, 2021	7	0–0	0.00–0.00	0–0	0.00–0.00
	May 3 to May 9, 2021	9	0–2	0.00–28.57	0–0	0.00–0.00
	May 10 to May 16, 2021	8	0–1	0.00–14.29	0–0	0.00–0.00
	May 17 to May 23, 2021	8	0–1	0.00–14.29	0–0	0.00–0.00
	May 24 to May 30, 2021	10	0–3	0.00–42.86	0–0	0.00–0.00
Gifu						
	Apr 5 to Apr 11, 2021	12	0–3	0.00–33.33	0–0	0.00–0.00
	Apr 12 to Apr 18, 2021	9	0–0	0.00–0.00	0–0	0.00–0.00
	Apr 19 to Apr 25, 2021	12	0–3	0.00–33.33	0–0	0.00–0.00
	Apr 26 to May 2, 2021	10	0–1	0.00–11.11	0–0	0.00–0.00
	May 3 to May 9, 2021	11	0–2	0.00–22.22	0–0	0.00–0.00
	May 10 to May 16, 2021	8	0–0	0.00–0.00	0–1	0.00–11.11
	May 17 to May 23, 2021	6	0–0	0.00–0.00	0–3	0.00–33.33
	May 24 to May 30, 2021	7	0–0	0.00–0.00	0–2	0.00–22.22
Shizuoka						
	Apr 5 to Apr 11, 2021	14	0–0	0.00–0.00	0–1	0.00–6.67
	Apr 12 to Apr 18, 2021	19	0–4	0.00–26.67	0–0	0.00–0.00
	Apr 19 to Apr 25, 2021	12	0–0	0.00–0.00	0–3	0.00–20.00
	Apr 26 to May 2, 2021	19	0–5	0.00–35.71	0–0	0.00–0.00
	May 3 to May 9, 2021	9	0–0	0.00–0.00	0–5	0.00–35.71
	May 10 to May 16, 2021	18	0–3	0.00–20.00	0–0	0.00–0.00
	May 17 to May 23, 2021	21	0–6	0.00–40.00	0–0	0.00–0.00
	May 24 to May 30, 2021	14	0–0	0.00–0.00	0–0	0.00–0.00
Aichi						
	Apr 5 to Apr 11, 2021	28	0–1	0.00–3.70	0–0	0.00–0.00
	Apr 12 to Apr 18, 2021	26	0–0	0.00–0.00	0–0	0.00–0.00
	Apr 19 to Apr 25, 2021	25	0–0	0.00–0.00	0–2	0.00–7.41
	Apr 26 to May 2, 2021	24	0–0	0.00–0.00	0–4	0.00–14.29
	May 3 to May 9, 2021	27	0–0	0.00–0.00	0–1	0.00–3.57
	May 10 to May 16, 2021	21	0–0	0.00–0.00	0–8	0.00–27.59
	May 17 to May 23, 2021	28	0–0	0.00–0.00	0–0	0.00–0.00
	May 24 to May 30, 2021	33	0–4	0.00–13.79	0–0	0.00–0.00
Mie						
	Apr 5 to Apr 11, 2021	4	0–0	0.00–0.00	0–3	0.00–42.86
	Apr 12 to Apr 18, 2021	11	0–5	0.00–83.33	0–0	0.00–0.00
	Apr 19 to Apr 25, 2021	7	0–1	0.00–16.67	0–0	0.00–0.00
	Apr 26 to May 2, 2021	6	0–0	0.00–0.00	0–0	0.00–0.00
	May 3 to May 9, 2021	8	0–2	0.00–33.33	0–0	0.00–0.00
	May 10 to May 16, 2021	13	1–6	14.29–85.71	0–0	0.00–0.00
	May 17 to May 23, 2021	12	0–6	0.00–100.00	0–0	0.00–0.00
	May 24 to May 30, 2021	8	0–1	0.00–14.29	0–0	0.00–0.00
Shiga						

	Apr 5 to Apr 11, 2021	6	0–3	0.00–100.00	0–0	0.00–0.00
	Apr 12 to Apr 18, 2021	5	0–2	0.00–66.67	0–0	0.00–0.00
	Apr 19 to Apr 25, 2021	3	0–0	0.00–0.00	0–0	0.00–0.00
	Apr 26 to May 2, 2021	5	0–2	0.00–66.67	0–0	0.00–0.00
	May 3 to May 9, 2021	0	0–0	0.00–0.00	0–3	0.00–100.00
	May 10 to May 16, 2021	4	0–2	0.00–100.00	0–0	0.00–0.00
	May 17 to May 23, 2021	2	0–0	0.00–0.00	0–0	0.00–0.00
	May 24 to May 30, 2021	5	0–3	0.00–150.00	0–0	0.00–0.00
Kyoto						
	Apr 5 to Apr 11, 2021	7	0–2	0.00–40.00	0–0	0.00–0.00
	Apr 12 to Apr 18, 2021	5	0–1	0.00–25.00	0–0	0.00–0.00
	Apr 19 to Apr 25, 2021	9	0–4	0.00–80.00	0–0	0.00–0.00
	Apr 26 to May 2, 2021	7	0–2	0.00–40.00	0–0	0.00–0.00
	May 3 to May 9, 2021	4	0–0	0.00–0.00	0–1	0.00–20.00
	May 10 to May 16, 2021	11	2–6	40.00–120.00	0–0	0.00–0.00
	May 17 to May 23, 2021	3	0–0	0.00–0.00	0–2	0.00–40.00
	May 24 to May 30, 2021	11	1–6	20.00–120.00	0–0	0.00–0.00
Osaka						
	Apr 5 to Apr 11, 2021	27	0–0	0.00–0.00	0–1	0.00–3.57
	Apr 12 to Apr 18, 2021	27	0–0	0.00–0.00	0–1	0.00–3.57
	Apr 19 to Apr 25, 2021	25	0–0	0.00–0.00	0–2	0.00–7.41
	Apr 26 to May 2, 2021	32	0–5	0.00–18.52	0–0	0.00–0.00
	May 3 to May 9, 2021	30	0–3	0.00–11.11	0–0	0.00–0.00
	May 10 to May 16, 2021	28	0–1	0.00–3.70	0–0	0.00–0.00
	May 17 to May 23, 2021	20	0–0	0.00–0.00	0–6	0.00–23.08
	May 24 to May 30, 2021	25	0–0	0.00–0.00	0–0	0.00–0.00
Hyogo						
	Apr 5 to Apr 11, 2021	11	0–0	0.00–0.00	0–1	0.00–8.33
	Apr 12 to Apr 18, 2021	20	0–8	0.00–66.67	0–0	0.00–0.00
	Apr 19 to Apr 25, 2021	9	0–0	0.00–0.00	0–3	0.00–25.00
	Apr 26 to May 2, 2021	13	0–1	0.00–8.33	0–0	0.00–0.00
	May 3 to May 9, 2021	12	0–1	0.00–9.09	0–0	0.00–0.00
	May 10 to May 16, 2021	16	0–4	0.00–33.33	0–0	0.00–0.00
	May 17 to May 23, 2021	16	0–4	0.00–33.33	0–0	0.00–0.00
	May 24 to May 30, 2021	16	0–4	0.00–33.33	0–0	0.00–0.00
Nara						
	Apr 5 to Apr 11, 2021	4	0–0	0.00–0.00	0–0	0.00–0.00
	Apr 12 to Apr 18, 2021	7	0–3	0.00–75.00	0–0	0.00–0.00
	Apr 19 to Apr 25, 2021	3	0–0	0.00–0.00	0–1	0.00–25.00
	Apr 26 to May 2, 2021	6	0–2	0.00–50.00	0–0	0.00–0.00
	May 3 to May 9, 2021	3	0–0	0.00–0.00	0–1	0.00–25.00
	May 10 to May 16, 2021	5	0–2	0.00–66.67	0–0	0.00–0.00
	May 17 to May 23, 2021	10	3–7	100.00–233.33	0–0	0.00–0.00
	May 24 to May 30, 2021	3	0–0	0.00–0.00	0–1	0.00–25.00
Wakayama						
	Apr 5 to Apr 11, 2021	8	0–4	0.00–100.00	0–0	0.00–0.00

	Apr 12 to Apr 18, 2021	3	0–0	0.00–0.00	0–1	0.00–25.00
	Apr 19 to Apr 25, 2021	5	0–1	0.00–25.00	0–0	0.00–0.00
	Apr 26 to May 2, 2021	6	0–2	0.00–50.00	0–0	0.00–0.00
	May 3 to May 9, 2021	8	0–4	0.00–100.00	0–0	0.00–0.00
	May 10 to May 16, 2021	8	0–4	0.00–100.00	0–0	0.00–0.00
	May 17 to May 23, 2021	7	0–3	0.00–75.00	0–0	0.00–0.00
	May 24 to May 30, 2021	2	0–0	0.00–0.00	0–2	0.00–50.00
Tottori						
	Apr 5 to Apr 11, 2021	3	0–0	0.00–0.00	0–0	0.00–0.00
	Apr 12 to Apr 18, 2021	3	0–0	0.00–0.00	0–0	0.00–0.00
	Apr 19 to Apr 25, 2021	3	0–1	0.00–50.00	0–0	0.00–0.00
	Apr 26 to May 2, 2021	3	0–1	0.00–50.00	0–0	0.00–0.00
	May 3 to May 9, 2021	4	0–2	0.00–100.00	0–0	0.00–0.00
	May 10 to May 16, 2021	0	0–0	0.00–0.00	0–2	0.00–100.00
	May 17 to May 23, 2021	0	0–0	0.00–0.00	0–2	0.00–100.00
	May 24 to May 30, 2021	6	0–4	0.00–200.00	0–0	0.00–0.00
Shimane						
	Apr 5 to Apr 11, 2021	2	0–0	0.00–0.00	0–1	0.00–33.33
	Apr 12 to Apr 18, 2021	0	0–0	0.00–0.00	0–3	0.00–100.00
	Apr 19 to Apr 25, 2021	7	0–4	0.00–133.33	0–0	0.00–0.00
	Apr 26 to May 2, 2021	2	0–0	0.00–0.00	0–1	0.00–33.33
	May 3 to May 9, 2021	4	0–1	0.00–33.33	0–0	0.00–0.00
	May 10 to May 16, 2021	3	0–0	0.00–0.00	0–0	0.00–0.00
	May 17 to May 23, 2021	3	0–0	0.00–0.00	0–0	0.00–0.00
	May 24 to May 30, 2021	4	0–1	0.00–33.33	0–0	0.00–0.00
Okayama						
	Apr 5 to Apr 11, 2021	1	0–0	0.00–0.00	0–4	0.00–80.00
	Apr 12 to Apr 18, 2021	3	0–0	0.00–0.00	0–2	0.00–40.00
	Apr 19 to Apr 25, 2021	4	0–0	0.00–0.00	0–2	0.00–33.33
	Apr 26 to May 2, 2021	8	0–2	0.00–33.33	0–0	0.00–0.00
	May 3 to May 9, 2021	10	0–4	0.00–66.67	0–0	0.00–0.00
	May 10 to May 16, 2021	7	0–1	0.00–16.67	0–0	0.00–0.00
	May 17 to May 23, 2021	8	0–3	0.00–60.00	0–0	0.00–0.00
	May 24 to May 30, 2021	6	0–1	0.00–20.00	0–0	0.00–0.00
Hiroshima						
	Apr 5 to Apr 11, 2021	6	0–0	0.00–0.00	0–1	0.00–14.29
	Apr 12 to Apr 18, 2021	11	0–5	0.00–83.33	0–0	0.00–0.00
	Apr 19 to Apr 25, 2021	8	0–2	0.00–33.33	0–0	0.00–0.00
	Apr 26 to May 2, 2021	7	0–1	0.00–16.67	0–0	0.00–0.00
	May 3 to May 9, 2021	6	0–0	0.00–0.00	0–0	0.00–0.00
	May 10 to May 16, 2021	7	0–1	0.00–16.67	0–0	0.00–0.00
	May 17 to May 23, 2021	10	0–4	0.00–66.67	0–0	0.00–0.00
	May 24 to May 30, 2021	5	0–0	0.00–0.00	0–1	0.00–16.67
Yamaguchi						
	Apr 5 to Apr 11, 2021	2	0–0	0.00–0.00	0–2	0.00–50.00
	Apr 12 to Apr 18, 2021	1	0–0	0.00–0.00	0–3	0.00–75.00

	Apr 19 to Apr 25, 2021	5	0–1	0.00–25.00	0–0	0.00–0.00
	Apr 26 to May 2, 2021	6	0–2	0.00–50.00	0–0	0.00–0.00
	May 3 to May 9, 2021	5	0–1	0.00–25.00	0–0	0.00–0.00
	May 10 to May 16, 2021	10	2–6	50.00–150.00	0–0	0.00–0.00
	May 17 to May 23, 2021	4	0–0	0.00–0.00	0–0	0.00–0.00
	May 24 to May 30, 2021	4	0–0	0.00–0.00	0–0	0.00–0.00
Tokushima						
	Apr 5 to Apr 11, 2021	2	0–0	0.00–0.00	0–0	0.00–0.00
	Apr 12 to Apr 18, 2021	0	NA	NA	NA	NA
	Apr 19 to Apr 25, 2021	2	NA	NA	NA	NA
	Apr 26 to May 2, 2021	2	0–0	0.00–0.00	0–0	0.00–0.00
	May 3 to May 9, 2021	0	NA	NA	NA	NA
	May 10 to May 16, 2021	1	0–0	0.00–0.00	0–1	0.00–50.00
	May 17 to May 23, 2021	1	NA	NA	NA	NA
	May 24 to May 30, 2021	2	NA	NA	NA	NA
Kagawa						
	Apr 5 to Apr 11, 2021	3	0–0	0.00–0.00	0–1	0.00–25.00
	Apr 12 to Apr 18, 2021	4	0–0	0.00–0.00	0–0	0.00–0.00
	Apr 19 to Apr 25, 2021	4	0–0	0.00–0.00	0–0	0.00–0.00
	Apr 26 to May 2, 2021	1	0–0	0.00–0.00	0–3	0.00–75.00
	May 3 to May 9, 2021	6	0–2	0.00–50.00	0–0	0.00–0.00
	May 10 to May 16, 2021	5	0–1	0.00–25.00	0–0	0.00–0.00
	May 17 to May 23, 2021	3	0–0	0.00–0.00	0–1	0.00–25.00
	May 24 to May 30, 2021	7	0–3	0.00–75.00	0–0	0.00–0.00
Ehime						
	Apr 5 to Apr 11, 2021	4	0–0	0.00–0.00	0–1	0.00–20.00
	Apr 12 to Apr 18, 2021	2	0–0	0.00–0.00	0–3	0.00–60.00
	Apr 19 to Apr 25, 2021	6	0–1	0.00–20.00	0–0	0.00–0.00
	Apr 26 to May 2, 2021	4	0–0	0.00–0.00	0–1	0.00–20.00
	May 3 to May 9, 2021	2	0–0	0.00–0.00	0–3	0.00–60.00
	May 10 to May 16, 2021	6	0–2	0.00–50.00	0–0	0.00–0.00
	May 17 to May 23, 2021	5	0–1	0.00–25.00	0–0	0.00–0.00
	May 24 to May 30, 2021	7	0–3	0.00–75.00	0–0	0.00–0.00
Kochi						
	Apr 5 to Apr 11, 2021	1	NA	NA	NA	NA
	Apr 12 to Apr 18, 2021	5	1–4	100.00–400.00	0–0	0.00–0.00
	Apr 19 to Apr 25, 2021	1	0–0	0.00–0.00	0–0	0.00–0.00
	Apr 26 to May 2, 2021	2	0–1	0.00–100.00	0–0	0.00–0.00
	May 3 to May 9, 2021	0	0–0	0.00–0.00	0–1	0.00–100.00
	May 10 to May 16, 2021	4	0–3	0.00–300.00	0–0	0.00–0.00
	May 17 to May 23, 2021	4	0–3	0.00–300.00	0–0	0.00–0.00
	May 24 to May 30, 2021	0	0–0	0.00–0.00	0–2	0.00–100.00
Fukuoka						
	Apr 5 to Apr 11, 2021	12	0–1	0.00–9.09	0–0	0.00–0.00
	Apr 12 to Apr 18, 2021	18	1–8	10.00–80.00	0–0	0.00–0.00
	Apr 19 to Apr 25, 2021	21	4–11	40.00–110.00	0–0	0.00–0.00

	Apr 26 to May 2, 2021	14	0–4	0.00–40.00	0–0	0.00–0.00
	May 3 to May 9, 2021	11	0–1	0.00–10.00	0–0	0.00–0.00
	May 10 to May 16, 2021	11	0–0	0.00–0.00	0–0	0.00–0.00
	May 17 to May 23, 2021	18	1–7	9.09–63.64	0–0	0.00–0.00
	May 24 to May 30, 2021	16	0–5	0.00–45.45	0–0	0.00–0.00
Saga						
	Apr 5 to Apr 11, 2021	1	0–0	0.00–0.00	0–2	0.00–66.67
	Apr 12 to Apr 18, 2021	2	0–0	0.00–0.00	0–1	0.00–33.33
	Apr 19 to Apr 25, 2021	3	0–0	0.00–0.00	0–0	0.00–0.00
	Apr 26 to May 2, 2021	4	0–1	0.00–33.33	0–0	0.00–0.00
	May 3 to May 9, 2021	5	0–2	0.00–66.67	0–0	0.00–0.00
	May 10 to May 16, 2021	2	0–0	0.00–0.00	0–1	0.00–33.33
	May 17 to May 23, 2021	4	0–1	0.00–33.33	0–0	0.00–0.00
	May 24 to May 30, 2021	6	0–4	0.00–200.00	0–0	0.00–0.00
Nagasaki						
	Apr 5 to Apr 11, 2021	3	0–0	0.00–0.00	0–0	0.00–0.00
	Apr 12 to Apr 18, 2021	2	0–0	0.00–0.00	0–1	0.00–33.33
	Apr 19 to Apr 25, 2021	4	0–1	0.00–33.33	0–0	0.00–0.00
	Apr 26 to May 2, 2021	5	0–1	0.00–25.00	0–0	0.00–0.00
	May 3 to May 9, 2021	2	0–0	0.00–0.00	0–2	0.00–50.00
	May 10 to May 16, 2021	7	0–3	0.00–75.00	0–0	0.00–0.00
	May 17 to May 23, 2021	4	0–0	0.00–0.00	0–0	0.00–0.00
	May 24 to May 30, 2021	7	0–3	0.00–75.00	0–0	0.00–0.00
Kumamoto						
	Apr 5 to Apr 11, 2021	5	0–1	0.00–25.00	0–0	0.00–0.00
	Apr 12 to Apr 18, 2021	5	0–1	0.00–25.00	0–0	0.00–0.00
	Apr 19 to Apr 25, 2021	4	0–0	0.00–0.00	0–0	0.00–0.00
	Apr 26 to May 2, 2021	9	1–5	25.00–125.00	0–0	0.00–0.00
	May 3 to May 9, 2021	6	0–2	0.00–50.00	0–0	0.00–0.00
	May 10 to May 16, 2021	6	0–2	0.00–50.00	0–0	0.00–0.00
	May 17 to May 23, 2021	2	0–0	0.00–0.00	0–2	0.00–50.00
	May 24 to May 30, 2021	6	0–2	0.00–50.00	0–0	0.00–0.00
Oita						
	Apr 5 to Apr 11, 2021	3	0–0	0.00–0.00	0–2	0.00–40.00
	Apr 12 to Apr 18, 2021	13	3–8	60.00–160.00	0–0	0.00–0.00
	Apr 19 to Apr 25, 2021	4	0–0	0.00–0.00	0–1	0.00–20.00
	Apr 26 to May 2, 2021	4	0–0	0.00–0.00	0–1	0.00–20.00
	May 3 to May 9, 2021	13	3–8	60.00–160.00	0–0	0.00–0.00
	May 10 to May 16, 2021	5	0–0	0.00–0.00	0–0	0.00–0.00
	May 17 to May 23, 2021	5	0–0	0.00–0.00	0–1	0.00–16.67
	May 24 to May 30, 2021	5	0–0	0.00–0.00	0–1	0.00–16.67
Miyazaki						
	Apr 5 to Apr 11, 2021	4	0–0	0.00–0.00	0–1	0.00–20.00
	Apr 12 to Apr 18, 2021	4	0–0	0.00–0.00	0–1	0.00–20.00
	Apr 19 to Apr 25, 2021	2	0–0	0.00–0.00	0–3	0.00–60.00
	Apr 26 to May 2, 2021	3	0–0	0.00–0.00	0–2	0.00–40.00

	May 3 to May 9, 2021	4	0–0	0.00–0.00	0–1	0.00–20.00
	May 10 to May 16, 2021	4	0–0	0.00–0.00	0–1	0.00–20.00
	May 17 to May 23, 2021	9	0–4	0.00–80.00	0–0	0.00–0.00
	May 24 to May 30, 2021	2	0–0	0.00–0.00	0–3	0.00–60.00
Kagoshima						
	Apr 5 to Apr 11, 2021	4	0–0	0.00–0.00	0–1	0.00–20.00
	Apr 12 to Apr 18, 2021	8	0–3	0.00–60.00	0–0	0.00–0.00
	Apr 19 to Apr 25, 2021	8	0–3	0.00–60.00	0–0	0.00–0.00
	Apr 26 to May 2, 2021	4	0–0	0.00–0.00	0–1	0.00–20.00
	May 3 to May 9, 2021	4	0–0	0.00–0.00	0–1	0.00–20.00
	May 10 to May 16, 2021	3	0–0	0.00–0.00	0–3	0.00–50.00
	May 17 to May 23, 2021	11	0–5	0.00–83.33	0–0	0.00–0.00
	May 24 to May 30, 2021	6	0–0	0.00–0.00	0–0	0.00–0.00
Okinawa						
	Apr 5 to Apr 11, 2021	3	0–0	0.00–0.00	0–0	0.00–0.00
	Apr 12 to Apr 18, 2021	3	0–0	0.00–0.00	0–0	0.00–0.00
	Apr 19 to Apr 25, 2021	2	0–0	0.00–0.00	0–1	0.00–33.33
	Apr 26 to May 2, 2021	2	0–0	0.00–0.00	0–1	0.00–33.33
	May 3 to May 9, 2021	5	0–2	0.00–66.67	0–0	0.00–0.00
	May 10 to May 16, 2021	2	0–0	0.00–0.00	0–1	0.00–33.33
	May 17 to May 23, 2021	2	0–0	0.00–0.00	0–1	0.00–33.33
	May 24 to May 30, 2021	4	0–0	0.00–0.00	0–0	0.00–0.00

Table A.16: Weekly number of observed and excess/exiguous deaths in Japan and 47 prefectures for malignant neoplasms-related deaths at homes from January 2020 through May 2021.

Prefecture	Week	Observed	Excess deaths	Percent excess	Exiguous deaths	Percent exiguous
Japan						
	Apr 5 to Apr 11, 2021	1531	162–369	13.94–31.76	0–0	0.00–0.00
	Apr 12 to Apr 18, 2021	1576	204–412	17.53–35.40	0–0	0.00–0.00
	Apr 19 to Apr 25, 2021	1617	234–442	19.91–37.62	0–0	0.00–0.00
	Apr 26 to May 2, 2021	1703	294–511	24.66–42.87	0–0	0.00–0.00
	May 3 to May 9, 2021	1602	187–405	15.62–33.83	0–0	0.00–0.00
	May 10 to May 16, 2021	1589	145–370	11.89–30.35	0–0	0.00–0.00
	May 17 to May 23, 2021	1490	39–267	3.19–21.83	0–0	0.00–0.00
	May 24 to May 30, 2021	1546	78–309	6.31–24.98	0–0	0.00–0.00
Hokkaido						
	Apr 5 to Apr 11, 2021	45	0–6	0.00–15.38	0–0	0.00–0.00
	Apr 12 to Apr 18, 2021	52	0–13	0.00–33.33	0–0	0.00–0.00
	Apr 19 to Apr 25, 2021	43	0–3	0.00–7.50	0–0	0.00–0.00
	Apr 26 to May 2, 2021	64	9–23	21.95–56.10	0–0	0.00–0.00
	May 3 to May 9, 2021	48	0–8	0.00–20.00	0–0	0.00–0.00
	May 10 to May 16, 2021	53	0–13	0.00–32.50	0–0	0.00–0.00
	May 17 to May 23, 2021	44	0–3	0.00–7.32	0–0	0.00–0.00
	May 24 to May 30, 2021	67	11–26	26.83–63.41	0–0	0.00–0.00
Aomori						
	Apr 5 to Apr 11, 2021	6	0–0	0.00–0.00	0–3	0.00–33.33
	Apr 12 to Apr 18, 2021	6	0–0	0.00–0.00	0–3	0.00–33.33
	Apr 19 to Apr 25, 2021	12	0–3	0.00–33.33	0–0	0.00–0.00
	Apr 26 to May 2, 2021	11	0–2	0.00–22.22	0–0	0.00–0.00
	May 3 to May 9, 2021	5	0–0	0.00–0.00	0–4	0.00–44.44
	May 10 to May 16, 2021	8	0–0	0.00–0.00	0–1	0.00–11.11
	May 17 to May 23, 2021	7	0–0	0.00–0.00	0–2	0.00–22.22
	May 24 to May 30, 2021	13	0–3	0.00–30.00	0–0	0.00–0.00
Iwate						
	Apr 5 to Apr 11, 2021	5	0–0	0.00–0.00	0–2	0.00–28.57
	Apr 12 to Apr 18, 2021	9	0–2	0.00–28.57	0–0	0.00–0.00
	Apr 19 to Apr 25, 2021	12	1–6	16.67–100.00	0–0	0.00–0.00
	Apr 26 to May 2, 2021	14	3–8	50.00–133.33	0–0	0.00–0.00
	May 3 to May 9, 2021	6	0–0	0.00–0.00	0–0	0.00–0.00
	May 10 to May 16, 2021	9	0–3	0.00–50.00	0–0	0.00–0.00
	May 17 to May 23, 2021	7	0–1	0.00–16.67	0–0	0.00–0.00
	May 24 to May 30, 2021	9	0–3	0.00–50.00	0–0	0.00–0.00
Miyagi						
	Apr 5 to Apr 11, 2021	32	0–10	0.00–45.45	0–0	0.00–0.00
	Apr 12 to Apr 18, 2021	34	2–12	9.09–54.55	0–0	0.00–0.00
	Apr 19 to Apr 25, 2021	32	1–10	4.55–45.45	0–0	0.00–0.00
	Apr 26 to May 2, 2021	36	4–14	18.18–63.64	0–0	0.00–0.00
	May 3 to May 9, 2021	29	0–6	0.00–26.09	0–0	0.00–0.00

	May 10 to May 16, 2021	23	0–1	0.00–4.55	0–0	0.00–0.00
	May 17 to May 23, 2021	24	0–2	0.00–9.09	0–0	0.00–0.00
	May 24 to May 30, 2021	28	0–5	0.00–21.74	0–0	0.00–0.00
Akita						
	Apr 5 to Apr 11, 2021	9	1–5	25.00–125.00	0–0	0.00–0.00
	Apr 12 to Apr 18, 2021	7	0–3	0.00–75.00	0–0	0.00–0.00
	Apr 19 to Apr 25, 2021	9	0–5	0.00–125.00	0–0	0.00–0.00
	Apr 26 to May 2, 2021	5	0–1	0.00–25.00	0–0	0.00–0.00
	May 3 to May 9, 2021	6	0–2	0.00–50.00	0–0	0.00–0.00
	May 10 to May 16, 2021	5	0–1	0.00–25.00	0–0	0.00–0.00
	May 17 to May 23, 2021	5	0–1	0.00–25.00	0–0	0.00–0.00
	May 24 to May 30, 2021	5	0–1	0.00–25.00	0–0	0.00–0.00
Yamagata						
	Apr 5 to Apr 11, 2021	11	0–0	0.00–0.00	0–1	0.00–8.33
	Apr 12 to Apr 18, 2021	10	0–0	0.00–0.00	0–3	0.00–23.08
	Apr 19 to Apr 25, 2021	21	0–8	0.00–61.54	0–0	0.00–0.00
	Apr 26 to May 2, 2021	28	7–15	53.85–115.38	0–0	0.00–0.00
	May 3 to May 9, 2021	15	0–2	0.00–15.38	0–0	0.00–0.00
	May 10 to May 16, 2021	21	0–7	0.00–50.00	0–0	0.00–0.00
	May 17 to May 23, 2021	20	0–6	0.00–42.86	0–0	0.00–0.00
	May 24 to May 30, 2021	17	0–4	0.00–30.77	0–0	0.00–0.00
Fukushima						
	Apr 5 to Apr 11, 2021	17	0–4	0.00–30.77	0–0	0.00–0.00
	Apr 12 to Apr 18, 2021	32	11–19	84.62–146.15	0–0	0.00–0.00
	Apr 19 to Apr 25, 2021	16	0–2	0.00–14.29	0–0	0.00–0.00
	Apr 26 to May 2, 2021	28	6–14	42.86–100.00	0–0	0.00–0.00
	May 3 to May 9, 2021	24	2–10	14.29–71.43	0–0	0.00–0.00
	May 10 to May 16, 2021	20	0–5	0.00–33.33	0–0	0.00–0.00
	May 17 to May 23, 2021	21	0–7	0.00–50.00	0–0	0.00–0.00
	May 24 to May 30, 2021	16	0–1	0.00–6.67	0–0	0.00–0.00
Ibaraki						
	Apr 5 to Apr 11, 2021	26	0–5	0.00–23.81	0–0	0.00–0.00
	Apr 12 to Apr 18, 2021	41	10–20	47.62–95.24	0–0	0.00–0.00
	Apr 19 to Apr 25, 2021	38	6–16	27.27–72.73	0–0	0.00–0.00
	Apr 26 to May 2, 2021	32	0–10	0.00–45.45	0–0	0.00–0.00
	May 3 to May 9, 2021	28	0–6	0.00–27.27	0–0	0.00–0.00
	May 10 to May 16, 2021	33	1–11	4.55–50.00	0–0	0.00–0.00
	May 17 to May 23, 2021	21	0–0	0.00–0.00	0–1	0.00–4.55
	May 24 to May 30, 2021	31	0–9	0.00–40.91	0–0	0.00–0.00
Tochigi						
	Apr 5 to Apr 11, 2021	22	0–3	0.00–15.79	0–0	0.00–0.00
	Apr 12 to Apr 18, 2021	22	0–3	0.00–15.79	0–0	0.00–0.00
	Apr 19 to Apr 25, 2021	20	0–1	0.00–5.26	0–0	0.00–0.00
	Apr 26 to May 2, 2021	34	7–16	38.89–88.89	0–0	0.00–0.00
	May 3 to May 9, 2021	31	4–13	22.22–72.22	0–0	0.00–0.00
	May 10 to May 16, 2021	20	0–1	0.00–5.26	0–0	0.00–0.00

	May 17 to May 23, 2021	13	0–0	0.00–0.00	0–6	0.00–31.58
	May 24 to May 30, 2021	30	1–10	5.00–50.00	0–0	0.00–0.00
Gunma						
	Apr 5 to Apr 11, 2021	20	0–1	0.00–5.26	0–0	0.00–0.00
	Apr 12 to Apr 18, 2021	24	0–4	0.00–20.00	0–0	0.00–0.00
	Apr 19 to Apr 25, 2021	25	0–5	0.00–25.00	0–0	0.00–0.00
	Apr 26 to May 2, 2021	21	0–0	0.00–0.00	0–0	0.00–0.00
	May 3 to May 9, 2021	17	0–0	0.00–0.00	0–4	0.00–19.05
	May 10 to May 16, 2021	24	0–2	0.00–9.09	0–0	0.00–0.00
	May 17 to May 23, 2021	15	0–0	0.00–0.00	0–7	0.00–31.82
	May 24 to May 30, 2021	19	0–0	0.00–0.00	0–3	0.00–13.64
Saitama						
	Apr 5 to Apr 11, 2021	97	1–23	1.35–31.08	0–0	0.00–0.00
	Apr 12 to Apr 18, 2021	93	0–20	0.00–27.40	0–0	0.00–0.00
	Apr 19 to Apr 25, 2021	93	0–19	0.00–25.68	0–0	0.00–0.00
	Apr 26 to May 2, 2021	88	0–10	0.00–12.82	0–0	0.00–0.00
	May 3 to May 9, 2021	93	0–13	0.00–16.25	0–0	0.00–0.00
	May 10 to May 16, 2021	97	0–17	0.00–21.25	0–0	0.00–0.00
	May 17 to May 23, 2021	106	4–28	5.13–35.90	0–0	0.00–0.00
	May 24 to May 30, 2021	85	0–6	0.00–7.59	0–0	0.00–0.00
Chiba						
	Apr 5 to Apr 11, 2021	86	11–29	19.30–50.88	0–0	0.00–0.00
	Apr 12 to Apr 18, 2021	101	25–43	43.10–74.14	0–0	0.00–0.00
	Apr 19 to Apr 25, 2021	81	5–23	8.62–39.66	0–0	0.00–0.00
	Apr 26 to May 2, 2021	61	0–2	0.00–3.39	0–0	0.00–0.00
	May 3 to May 9, 2021	87	8–28	13.56–47.46	0–0	0.00–0.00
	May 10 to May 16, 2021	68	0–8	0.00–13.33	0–0	0.00–0.00
	May 17 to May 23, 2021	72	0–12	0.00–20.00	0–0	0.00–0.00
	May 24 to May 30, 2021	69	0–10	0.00–16.95	0–0	0.00–0.00
Tokyo						
	Apr 5 to Apr 11, 2021	209	14–49	8.75–30.63	0–0	0.00–0.00
	Apr 12 to Apr 18, 2021	203	7–42	4.35–26.09	0–0	0.00–0.00
	Apr 19 to Apr 25, 2021	197	0–34	0.00–20.86	0–0	0.00–0.00
	Apr 26 to May 2, 2021	221	21–58	12.88–35.58	0–0	0.00–0.00
	May 3 to May 9, 2021	185	0–20	0.00–12.12	0–0	0.00–0.00
	May 10 to May 16, 2021	187	0–20	0.00–11.98	0–0	0.00–0.00
	May 17 to May 23, 2021	196	0–27	0.00–15.98	0–0	0.00–0.00
	May 24 to May 30, 2021	189	0–18	0.00–10.53	0–0	0.00–0.00
Kanagawa						
	Apr 5 to Apr 11, 2021	139	0–21	0.00–17.80	0–0	0.00–0.00
	Apr 12 to Apr 18, 2021	143	0–22	0.00–18.18	0–0	0.00–0.00
	Apr 19 to Apr 25, 2021	150	0–27	0.00–21.95	0–0	0.00–0.00
	Apr 26 to May 2, 2021	164	7–36	5.47–28.13	0–0	0.00–0.00
	May 3 to May 9, 2021	162	4–35	3.15–27.56	0–0	0.00–0.00
	May 10 to May 16, 2021	165	5–36	3.88–27.91	0–0	0.00–0.00
	May 17 to May 23, 2021	138	0–8	0.00–6.15	0–0	0.00–0.00

	May 24 to May 30, 2021	141	0–8	0.00–6.02	0–0	0.00–0.00
Niigata						
	Apr 5 to Apr 11, 2021	10	0–0	0.00–0.00	0–0	0.00–0.00
	Apr 12 to Apr 18, 2021	12	0–2	0.00–20.00	0–0	0.00–0.00
	Apr 19 to Apr 25, 2021	13	0–3	0.00–30.00	0–0	0.00–0.00
	Apr 26 to May 2, 2021	17	0–7	0.00–70.00	0–0	0.00–0.00
	May 3 to May 9, 2021	11	0–1	0.00–10.00	0–0	0.00–0.00
	May 10 to May 16, 2021	8	0–0	0.00–0.00	0–3	0.00–27.27
	May 17 to May 23, 2021	6	0–0	0.00–0.00	0–5	0.00–45.45
	May 24 to May 30, 2021	16	0–5	0.00–45.45	0–0	0.00–0.00
Toyama						
	Apr 5 to Apr 11, 2021	24	8–14	80.00–140.00	0–0	0.00–0.00
	Apr 12 to Apr 18, 2021	13	0–3	0.00–30.00	0–0	0.00–0.00
	Apr 19 to Apr 25, 2021	8	0–0	0.00–0.00	0–2	0.00–20.00
	Apr 26 to May 2, 2021	16	0–6	0.00–60.00	0–0	0.00–0.00
	May 3 to May 9, 2021	14	0–4	0.00–40.00	0–0	0.00–0.00
	May 10 to May 16, 2021	18	0–7	0.00–63.64	0–0	0.00–0.00
	May 17 to May 23, 2021	8	0–0	0.00–0.00	0–2	0.00–20.00
	May 24 to May 30, 2021	10	0–0	0.00–0.00	0–0	0.00–0.00
Ishikawa						
	Apr 5 to Apr 11, 2021	17	3–9	37.50–112.50	0–0	0.00–0.00
	Apr 12 to Apr 18, 2021	12	0–4	0.00–50.00	0–0	0.00–0.00
	Apr 19 to Apr 25, 2021	14	0–5	0.00–55.56	0–0	0.00–0.00
	Apr 26 to May 2, 2021	15	0–6	0.00–66.67	0–0	0.00–0.00
	May 3 to May 9, 2021	18	2–9	22.22–100.00	0–0	0.00–0.00
	May 10 to May 16, 2021	10	0–1	0.00–11.11	0–0	0.00–0.00
	May 17 to May 23, 2021	12	0–3	0.00–33.33	0–0	0.00–0.00
	May 24 to May 30, 2021	9	0–0	0.00–0.00	0–0	0.00–0.00
Fukui						
	Apr 5 to Apr 11, 2021	3	0–0	0.00–0.00	0–3	0.00–50.00
	Apr 12 to Apr 18, 2021	8	0–2	0.00–33.33	0–0	0.00–0.00
	Apr 19 to Apr 25, 2021	4	0–0	0.00–0.00	0–2	0.00–33.33
	Apr 26 to May 2, 2021	11	0–5	0.00–83.33	0–0	0.00–0.00
	May 3 to May 9, 2021	9	0–4	0.00–80.00	0–0	0.00–0.00
	May 10 to May 16, 2021	12	2–7	40.00–140.00	0–0	0.00–0.00
	May 17 to May 23, 2021	5	0–0	0.00–0.00	0–1	0.00–16.67
	May 24 to May 30, 2021	11	0–5	0.00–83.33	0–0	0.00–0.00
Yamanashi						
	Apr 5 to Apr 11, 2021	8	0–0	0.00–0.00	0–2	0.00–20.00
	Apr 12 to Apr 18, 2021	7	0–0	0.00–0.00	0–3	0.00–30.00
	Apr 19 to Apr 25, 2021	8	0–0	0.00–0.00	0–2	0.00–20.00
	Apr 26 to May 2, 2021	7	0–0	0.00–0.00	0–3	0.00–30.00
	May 3 to May 9, 2021	13	0–4	0.00–44.44	0–0	0.00–0.00
	May 10 to May 16, 2021	14	0–5	0.00–55.56	0–0	0.00–0.00
	May 17 to May 23, 2021	21	4–11	40.00–110.00	0–0	0.00–0.00
	May 24 to May 30, 2021	15	0–5	0.00–50.00	0–0	0.00–0.00

Nagano						
	Apr 5 to Apr 11, 2021	27	0–7	0.00–35.00	0–0	0.00–0.00
	Apr 12 to Apr 18, 2021	18	0–0	0.00–0.00	0–2	0.00–10.00
	Apr 19 to Apr 25, 2021	19	0–0	0.00–0.00	0–1	0.00–5.00
	Apr 26 to May 2, 2021	20	0–1	0.00–5.26	0–0	0.00–0.00
	May 3 to May 9, 2021	23	0–4	0.00–21.05	0–0	0.00–0.00
	May 10 to May 16, 2021	21	0–2	0.00–10.53	0–0	0.00–0.00
	May 17 to May 23, 2021	23	0–4	0.00–21.05	0–0	0.00–0.00
	May 24 to May 30, 2021	19	0–0	0.00–0.00	0–0	0.00–0.00
Gifu						
	Apr 5 to Apr 11, 2021	34	0–10	0.00–41.67	0–0	0.00–0.00
	Apr 12 to Apr 18, 2021	41	6–17	25.00–70.83	0–0	0.00–0.00
	Apr 19 to Apr 25, 2021	40	5–15	20.00–60.00	0–0	0.00–0.00
	Apr 26 to May 2, 2021	29	0–5	0.00–20.83	0–0	0.00–0.00
	May 3 to May 9, 2021	35	0–11	0.00–45.83	0–0	0.00–0.00
	May 10 to May 16, 2021	33	0–9	0.00–37.50	0–0	0.00–0.00
	May 17 to May 23, 2021	41	6–17	25.00–70.83	0–0	0.00–0.00
	May 24 to May 30, 2021	27	0–3	0.00–12.50	0–0	0.00–0.00
Shizuoka						
	Apr 5 to Apr 11, 2021	48	0–5	0.00–11.63	0–0	0.00–0.00
	Apr 12 to Apr 18, 2021	41	0–0	0.00–0.00	0–2	0.00–4.65
	Apr 19 to Apr 25, 2021	48	0–4	0.00–9.09	0–0	0.00–0.00
	Apr 26 to May 2, 2021	60	2–17	4.65–39.53	0–0	0.00–0.00
	May 3 to May 9, 2021	58	1–16	2.38–38.10	0–0	0.00–0.00
	May 10 to May 16, 2021	39	0–0	0.00–0.00	0–2	0.00–4.88
	May 17 to May 23, 2021	52	0–11	0.00–26.83	0–0	0.00–0.00
	May 24 to May 30, 2021	37	0–0	0.00–0.00	0–6	0.00–13.95
Aichi						
	Apr 5 to Apr 11, 2021	70	0–9	0.00–14.75	0–0	0.00–0.00
	Apr 12 to Apr 18, 2021	73	0–12	0.00–19.67	0–0	0.00–0.00
	Apr 19 to Apr 25, 2021	86	7–26	11.67–43.33	0–0	0.00–0.00
	Apr 26 to May 2, 2021	86	5–23	7.94–36.51	0–0	0.00–0.00
	May 3 to May 9, 2021	76	0–12	0.00–18.75	0–0	0.00–0.00
	May 10 to May 16, 2021	100	17–35	26.15–53.85	0–0	0.00–0.00
	May 17 to May 23, 2021	75	0–10	0.00–15.38	0–0	0.00–0.00
	May 24 to May 30, 2021	77	0–13	0.00–20.31	0–0	0.00–0.00
Mie						
	Apr 5 to Apr 11, 2021	24	0–5	0.00–26.32	0–0	0.00–0.00
	Apr 12 to Apr 18, 2021	22	0–3	0.00–15.79	0–0	0.00–0.00
	Apr 19 to Apr 25, 2021	23	0–4	0.00–21.05	0–0	0.00–0.00
	Apr 26 to May 2, 2021	23	0–4	0.00–21.05	0–0	0.00–0.00
	May 3 to May 9, 2021	22	0–4	0.00–22.22	0–0	0.00–0.00
	May 10 to May 16, 2021	20	0–2	0.00–11.11	0–0	0.00–0.00
	May 17 to May 23, 2021	26	0–9	0.00–52.94	0–0	0.00–0.00
	May 24 to May 30, 2021	24	0–6	0.00–33.33	0–0	0.00–0.00
Shiga						

	Apr 5 to Apr 11, 2021	14	0–5	0.00–55.56	0–0	0.00–0.00
	Apr 12 to Apr 18, 2021	14	0–5	0.00–55.56	0–0	0.00–0.00
	Apr 19 to Apr 25, 2021	18	3–9	33.33–100.00	0–0	0.00–0.00
	Apr 26 to May 2, 2021	17	1–7	10.00–70.00	0–0	0.00–0.00
	May 3 to May 9, 2021	16	0–7	0.00–77.78	0–0	0.00–0.00
	May 10 to May 16, 2021	22	6–13	66.67–144.44	0–0	0.00–0.00
	May 17 to May 23, 2021	14	0–5	0.00–55.56	0–0	0.00–0.00
	May 24 to May 30, 2021	19	2–9	20.00–90.00	0–0	0.00–0.00
Kyoto						
	Apr 5 to Apr 11, 2021	43	8–19	33.33–79.17	0–0	0.00–0.00
	Apr 12 to Apr 18, 2021	26	0–1	0.00–4.00	0–0	0.00–0.00
	Apr 19 to Apr 25, 2021	37	0–12	0.00–48.00	0–0	0.00–0.00
	Apr 26 to May 2, 2021	31	0–5	0.00–19.23	0–0	0.00–0.00
	May 3 to May 9, 2021	38	0–11	0.00–40.74	0–0	0.00–0.00
	May 10 to May 16, 2021	32	0–5	0.00–18.52	0–0	0.00–0.00
	May 17 to May 23, 2021	39	0–11	0.00–39.29	0–0	0.00–0.00
	May 24 to May 30, 2021	35	0–7	0.00–25.00	0–0	0.00–0.00
Osaka						
	Apr 5 to Apr 11, 2021	124	0–24	0.00–24.00	0–0	0.00–0.00
	Apr 12 to Apr 18, 2021	125	0–26	0.00–26.26	0–0	0.00–0.00
	Apr 19 to Apr 25, 2021	147	23–48	23.23–48.48	0–0	0.00–0.00
	Apr 26 to May 2, 2021	149	26–52	26.80–53.61	0–0	0.00–0.00
	May 3 to May 9, 2021	137	13–39	13.27–39.80	0–0	0.00–0.00
	May 10 to May 16, 2021	120	0–21	0.00–21.21	0–0	0.00–0.00
	May 17 to May 23, 2021	125	0–27	0.00–27.55	0–0	0.00–0.00
	May 24 to May 30, 2021	120	0–19	0.00–18.81	0–0	0.00–0.00
Hyogo						
	Apr 5 to Apr 11, 2021	73	0–12	0.00–19.67	0–0	0.00–0.00
	Apr 12 to Apr 18, 2021	100	19–38	30.65–61.29	0–0	0.00–0.00
	Apr 19 to Apr 25, 2021	95	13–32	20.63–50.79	0–0	0.00–0.00
	Apr 26 to May 2, 2021	111	31–50	50.82–81.97	0–0	0.00–0.00
	May 3 to May 9, 2021	93	10–30	15.87–47.62	0–0	0.00–0.00
	May 10 to May 16, 2021	97	14–34	22.22–53.97	0–0	0.00–0.00
	May 17 to May 23, 2021	81	0–18	0.00–28.57	0–0	0.00–0.00
	May 24 to May 30, 2021	91	6–26	9.23–40.00	0–0	0.00–0.00
Nara						
	Apr 5 to Apr 11, 2021	21	0–5	0.00–31.25	0–0	0.00–0.00
	Apr 12 to Apr 18, 2021	18	0–2	0.00–12.50	0–0	0.00–0.00
	Apr 19 to Apr 25, 2021	16	0–0	0.00–0.00	0–0	0.00–0.00
	Apr 26 to May 2, 2021	17	0–1	0.00–6.25	0–0	0.00–0.00
	May 3 to May 9, 2021	19	0–2	0.00–11.76	0–0	0.00–0.00
	May 10 to May 16, 2021	21	0–4	0.00–23.53	0–0	0.00–0.00
	May 17 to May 23, 2021	10	0–0	0.00–0.00	0–7	0.00–41.18
	May 24 to May 30, 2021	22	0–5	0.00–29.41	0–0	0.00–0.00
Wakayama						
	Apr 5 to Apr 11, 2021	9	0–0	0.00–0.00	0–2	0.00–18.18

	Apr 12 to Apr 18, 2021	14	0–3	0.00–27.27	0–0	0.00–0.00
	Apr 19 to Apr 25, 2021	11	0–0	0.00–0.00	0–0	0.00–0.00
	Apr 26 to May 2, 2021	12	0–1	0.00–9.09	0–0	0.00–0.00
	May 3 to May 9, 2021	23	4–12	36.36–109.09	0–0	0.00–0.00
	May 10 to May 16, 2021	15	0–5	0.00–50.00	0–0	0.00–0.00
	May 17 to May 23, 2021	16	0–5	0.00–45.45	0–0	0.00–0.00
	May 24 to May 30, 2021	16	0–5	0.00–45.45	0–0	0.00–0.00
Tottori						
	Apr 5 to Apr 11, 2021	5	0–1	0.00–25.00	0–0	0.00–0.00
	Apr 12 to Apr 18, 2021	5	0–1	0.00–25.00	0–0	0.00–0.00
	Apr 19 to Apr 25, 2021	4	0–0	0.00–0.00	0–0	0.00–0.00
	Apr 26 to May 2, 2021	8	0–4	0.00–100.00	0–0	0.00–0.00
	May 3 to May 9, 2021	4	0–0	0.00–0.00	0–0	0.00–0.00
	May 10 to May 16, 2021	8	0–3	0.00–60.00	0–0	0.00–0.00
	May 17 to May 23, 2021	9	0–4	0.00–80.00	0–0	0.00–0.00
	May 24 to May 30, 2021	11	1–6	20.00–120.00	0–0	0.00–0.00
Shimane						
	Apr 5 to Apr 11, 2021	6	0–1	0.00–20.00	0–0	0.00–0.00
	Apr 12 to Apr 18, 2021	9	0–4	0.00–80.00	0–0	0.00–0.00
	Apr 19 to Apr 25, 2021	3	0–0	0.00–0.00	0–2	0.00–40.00
	Apr 26 to May 2, 2021	6	0–1	0.00–20.00	0–0	0.00–0.00
	May 3 to May 9, 2021	6	0–1	0.00–20.00	0–0	0.00–0.00
	May 10 to May 16, 2021	2	0–0	0.00–0.00	0–2	0.00–50.00
	May 17 to May 23, 2021	2	0–0	0.00–0.00	0–3	0.00–60.00
	May 24 to May 30, 2021	4	0–0	0.00–0.00	0–1	0.00–20.00
Okayama						
	Apr 5 to Apr 11, 2021	16	0–4	0.00–33.33	0–0	0.00–0.00
	Apr 12 to Apr 18, 2021	15	0–2	0.00–15.38	0–0	0.00–0.00
	Apr 19 to Apr 25, 2021	21	0–7	0.00–50.00	0–0	0.00–0.00
	Apr 26 to May 2, 2021	22	0–7	0.00–46.67	0–0	0.00–0.00
	May 3 to May 9, 2021	16	0–1	0.00–6.67	0–0	0.00–0.00
	May 10 to May 16, 2021	27	4–12	26.67–80.00	0–0	0.00–0.00
	May 17 to May 23, 2021	20	0–5	0.00–33.33	0–0	0.00–0.00
	May 24 to May 30, 2021	25	2–10	13.33–66.67	0–0	0.00–0.00
Hiroshima						
	Apr 5 to Apr 11, 2021	24	0–6	0.00–33.33	0–0	0.00–0.00
	Apr 12 to Apr 18, 2021	26	0–8	0.00–44.44	0–0	0.00–0.00
	Apr 19 to Apr 25, 2021	33	6–15	33.33–83.33	0–0	0.00–0.00
	Apr 26 to May 2, 2021	20	0–2	0.00–11.11	0–0	0.00–0.00
	May 3 to May 9, 2021	36	9–18	50.00–100.00	0–0	0.00–0.00
	May 10 to May 16, 2021	25	0–7	0.00–38.89	0–0	0.00–0.00
	May 17 to May 23, 2021	33	6–15	33.33–83.33	0–0	0.00–0.00
	May 24 to May 30, 2021	25	0–6	0.00–31.58	0–0	0.00–0.00
Yamaguchi						
	Apr 5 to Apr 11, 2021	11	0–2	0.00–22.22	0–0	0.00–0.00
	Apr 12 to Apr 18, 2021	13	0–3	0.00–30.00	0–0	0.00–0.00

	Apr 19 to Apr 25, 2021	15	0–6	0.00–66.67	0–0	0.00–0.00
	Apr 26 to May 2, 2021	9	0–0	0.00–0.00	0–0	0.00–0.00
	May 3 to May 9, 2021	12	0–3	0.00–33.33	0–0	0.00–0.00
	May 10 to May 16, 2021	16	0–7	0.00–77.78	0–0	0.00–0.00
	May 17 to May 23, 2021	13	0–5	0.00–62.50	0–0	0.00–0.00
	May 24 to May 30, 2021	19	4–10	44.44–111.11	0–0	0.00–0.00
Tokushima						
	Apr 5 to Apr 11, 2021	10	0–5	0.00–100.00	0–0	0.00–0.00
	Apr 12 to Apr 18, 2021	7	0–2	0.00–40.00	0–0	0.00–0.00
	Apr 19 to Apr 25, 2021	7	0–2	0.00–40.00	0–0	0.00–0.00
	Apr 26 to May 2, 2021	12	2–7	40.00–140.00	0–0	0.00–0.00
	May 3 to May 9, 2021	11	1–6	20.00–120.00	0–0	0.00–0.00
	May 10 to May 16, 2021	7	0–2	0.00–40.00	0–0	0.00–0.00
	May 17 to May 23, 2021	6	0–1	0.00–20.00	0–0	0.00–0.00
	May 24 to May 30, 2021	7	0–2	0.00–40.00	0–0	0.00–0.00
Kagawa						
	Apr 5 to Apr 11, 2021	8	0–0	0.00–0.00	0–0	0.00–0.00
	Apr 12 to Apr 18, 2021	6	0–0	0.00–0.00	0–2	0.00–25.00
	Apr 19 to Apr 25, 2021	15	0–7	0.00–87.50	0–0	0.00–0.00
	Apr 26 to May 2, 2021	10	0–2	0.00–25.00	0–0	0.00–0.00
	May 3 to May 9, 2021	10	0–2	0.00–25.00	0–0	0.00–0.00
	May 10 to May 16, 2021	11	0–3	0.00–37.50	0–0	0.00–0.00
	May 17 to May 23, 2021	12	0–4	0.00–50.00	0–0	0.00–0.00
	May 24 to May 30, 2021	14	0–6	0.00–75.00	0–0	0.00–0.00
Ehime						
	Apr 5 to Apr 11, 2021	21	0–8	0.00–61.54	0–0	0.00–0.00
	Apr 12 to Apr 18, 2021	17	0–3	0.00–21.43	0–0	0.00–0.00
	Apr 19 to Apr 25, 2021	21	0–8	0.00–61.54	0–0	0.00–0.00
	Apr 26 to May 2, 2021	18	0–5	0.00–38.46	0–0	0.00–0.00
	May 3 to May 9, 2021	21	0–8	0.00–61.54	0–0	0.00–0.00
	May 10 to May 16, 2021	11	0–0	0.00–0.00	0–3	0.00–21.43
	May 17 to May 23, 2021	11	0–0	0.00–0.00	0–3	0.00–21.43
	May 24 to May 30, 2021	19	0–6	0.00–46.15	0–0	0.00–0.00
Kochi						
	Apr 5 to Apr 11, 2021	6	0–0	0.00–0.00	0–0	0.00–0.00
	Apr 12 to Apr 18, 2021	16	5–10	83.33–166.67	0–0	0.00–0.00
	Apr 19 to Apr 25, 2021	9	0–3	0.00–50.00	0–0	0.00–0.00
	Apr 26 to May 2, 2021	7	0–1	0.00–16.67	0–0	0.00–0.00
	May 3 to May 9, 2021	4	0–0	0.00–0.00	0–2	0.00–33.33
	May 10 to May 16, 2021	7	0–1	0.00–16.67	0–0	0.00–0.00
	May 17 to May 23, 2021	7	0–1	0.00–16.67	0–0	0.00–0.00
	May 24 to May 30, 2021	8	0–2	0.00–33.33	0–0	0.00–0.00
Fukuoka						
	Apr 5 to Apr 11, 2021	60	3–19	7.32–46.34	0–0	0.00–0.00
	Apr 12 to Apr 18, 2021	56	0–14	0.00–33.33	0–0	0.00–0.00
	Apr 19 to Apr 25, 2021	51	0–9	0.00–21.43	0–0	0.00–0.00

	Apr 26 to May 2, 2021	67	9–25	21.43–59.52	0–0	0.00–0.00
	May 3 to May 9, 2021	50	0–7	0.00–16.28	0–0	0.00–0.00
	May 10 to May 16, 2021	54	0–11	0.00–25.58	0–0	0.00–0.00
	May 17 to May 23, 2021	54	0–11	0.00–25.58	0–0	0.00–0.00
	May 24 to May 30, 2021	59	0–15	0.00–34.09	0–0	0.00–0.00
Saga						
	Apr 5 to Apr 11, 2021	5	0–0	0.00–0.00	0–1	0.00–16.67
	Apr 12 to Apr 18, 2021	11	0–5	0.00–83.33	0–0	0.00–0.00
	Apr 19 to Apr 25, 2021	12	0–6	0.00–100.00	0–0	0.00–0.00
	Apr 26 to May 2, 2021	7	0–1	0.00–16.67	0–0	0.00–0.00
	May 3 to May 9, 2021	14	2–8	33.33–133.33	0–0	0.00–0.00
	May 10 to May 16, 2021	4	0–0	0.00–0.00	0–2	0.00–33.33
	May 17 to May 23, 2021	14	2–8	33.33–133.33	0–0	0.00–0.00
	May 24 to May 30, 2021	8	0–2	0.00–33.33	0–0	0.00–0.00
Nagasaki						
	Apr 5 to Apr 11, 2021	19	0–5	0.00–35.71	0–0	0.00–0.00
	Apr 12 to Apr 18, 2021	18	0–4	0.00–28.57	0–0	0.00–0.00
	Apr 19 to Apr 25, 2021	19	0–5	0.00–35.71	0–0	0.00–0.00
	Apr 26 to May 2, 2021	12	0–0	0.00–0.00	0–2	0.00–14.29
	May 3 to May 9, 2021	13	0–0	0.00–0.00	0–0	0.00–0.00
	May 10 to May 16, 2021	21	1–8	7.69–61.54	0–0	0.00–0.00
	May 17 to May 23, 2021	10	0–0	0.00–0.00	0–2	0.00–16.67
	May 24 to May 30, 2021	21	2–9	16.67–75.00	0–0	0.00–0.00
Kumamoto						
	Apr 5 to Apr 11, 2021	18	0–7	0.00–63.64	0–0	0.00–0.00
	Apr 12 to Apr 18, 2021	17	0–6	0.00–54.55	0–0	0.00–0.00
	Apr 19 to Apr 25, 2021	20	2–9	18.18–81.82	0–0	0.00–0.00
	Apr 26 to May 2, 2021	17	0–5	0.00–41.67	0–0	0.00–0.00
	May 3 to May 9, 2021	14	0–2	0.00–16.67	0–0	0.00–0.00
	May 10 to May 16, 2021	20	1–8	8.33–66.67	0–0	0.00–0.00
	May 17 to May 23, 2021	14	0–2	0.00–16.67	0–0	0.00–0.00
	May 24 to May 30, 2021	14	0–1	0.00–7.69	0–0	0.00–0.00
Oita						
	Apr 5 to Apr 11, 2021	9	0–2	0.00–28.57	0–0	0.00–0.00
	Apr 12 to Apr 18, 2021	7	0–0	0.00–0.00	0–0	0.00–0.00
	Apr 19 to Apr 25, 2021	8	0–1	0.00–14.29	0–0	0.00–0.00
	Apr 26 to May 2, 2021	16	2–8	25.00–100.00	0–0	0.00–0.00
	May 3 to May 9, 2021	16	3–8	37.50–100.00	0–0	0.00–0.00
	May 10 to May 16, 2021	12	0–4	0.00–50.00	0–0	0.00–0.00
	May 17 to May 23, 2021	6	0–0	0.00–0.00	0–2	0.00–25.00
	May 24 to May 30, 2021	11	0–3	0.00–37.50	0–0	0.00–0.00
Miyazaki						
	Apr 5 to Apr 11, 2021	5	0–0	0.00–0.00	0–2	0.00–28.57
	Apr 12 to Apr 18, 2021	5	0–0	0.00–0.00	0–2	0.00–28.57
	Apr 19 to Apr 25, 2021	9	0–3	0.00–50.00	0–0	0.00–0.00
	Apr 26 to May 2, 2021	10	0–3	0.00–42.86	0–0	0.00–0.00

	May 3 to May 9, 2021	11	0–4	0.00–57.14	0–0	0.00–0.00
	May 10 to May 16, 2021	13	1–6	14.29–85.71	0–0	0.00–0.00
	May 17 to May 23, 2021	7	0–0	0.00–0.00	0–0	0.00–0.00
	May 24 to May 30, 2021	7	0–0	0.00–0.00	0–0	0.00–0.00
Kagoshima						
	Apr 5 to Apr 11, 2021	15	0–3	0.00–25.00	0–0	0.00–0.00
	Apr 12 to Apr 18, 2021	11	0–0	0.00–0.00	0–0	0.00–0.00
	Apr 19 to Apr 25, 2021	16	0–5	0.00–45.45	0–0	0.00–0.00
	Apr 26 to May 2, 2021	15	0–4	0.00–36.36	0–0	0.00–0.00
	May 3 to May 9, 2021	17	0–5	0.00–41.67	0–0	0.00–0.00
	May 10 to May 16, 2021	21	1–9	8.33–75.00	0–0	0.00–0.00
	May 17 to May 23, 2021	10	0–0	0.00–0.00	0–2	0.00–16.67
	May 24 to May 30, 2021	14	0–1	0.00–7.69	0–0	0.00–0.00
Okinawa						
	Apr 5 to Apr 11, 2021	20	4–10	40.00–100.00	0–0	0.00–0.00
	Apr 12 to Apr 18, 2021	16	0–6	0.00–60.00	0–0	0.00–0.00
	Apr 19 to Apr 25, 2021	18	1–8	10.00–80.00	0–0	0.00–0.00
	Apr 26 to May 2, 2021	19	1–8	9.09–72.73	0–0	0.00–0.00
	May 3 to May 9, 2021	6	0–0	0.00–0.00	0–4	0.00–40.00
	May 10 to May 16, 2021	15	0–5	0.00–50.00	0–0	0.00–0.00
	May 17 to May 23, 2021	19	2–9	20.00–90.00	0–0	0.00–0.00
	May 24 to May 30, 2021	13	0–3	0.00–30.00	0–0	0.00–0.00

Table A.17: Weekly number of observed and excess/exiguous deaths in Japan and 47 prefectures for senility-related deaths in all places from January 2020 through May 2021.

Prefecture	Week	Observed	Excess deaths	Percent excess	Exiguous deaths	Percent exiguous
Japan						
	Apr 5 to Apr 11, 2021	2750	0–58	0.00–2.15	0–0	0.00–0.00
	Apr 12 to Apr 18, 2021	3002	186–353	7.02–13.33	0–0	0.00–0.00
	Apr 19 to Apr 25, 2021	2860	90–265	3.47–10.21	0–0	0.00–0.00
	Apr 26 to May 2, 2021	2978	256–429	10.04–16.83	0–0	0.00–0.00
	May 3 to May 9, 2021	3002	329–487	13.08–19.36	0–0	0.00–0.00
	May 10 to May 16, 2021	2890	236–401	9.48–16.11	0–0	0.00–0.00
	May 17 to May 23, 2021	2781	164–321	6.67–13.05	0–0	0.00–0.00
	May 24 to May 30, 2021	2738	127–287	5.18–11.71	0–0	0.00–0.00
Hokkaido						
	Apr 5 to Apr 11, 2021	118	0–21	0.00–21.65	0–0	0.00–0.00
	Apr 12 to Apr 18, 2021	117	0–20	0.00–20.62	0–0	0.00–0.00
	Apr 19 to Apr 25, 2021	101	0–3	0.00–3.06	0–0	0.00–0.00
	Apr 26 to May 2, 2021	119	2–24	2.11–25.26	0–0	0.00–0.00
	May 3 to May 9, 2021	124	7–28	7.29–29.17	0–0	0.00–0.00
	May 10 to May 16, 2021	89	0–0	0.00–0.00	0–6	0.00–6.32
	May 17 to May 23, 2021	108	0–14	0.00–14.89	0–0	0.00–0.00
	May 24 to May 30, 2021	123	10–31	10.87–33.70	0–0	0.00–0.00
Aomori						
	Apr 5 to Apr 11, 2021	29	0–0	0.00–0.00	0–5	0.00–14.71
	Apr 12 to Apr 18, 2021	32	0–0	0.00–0.00	0–1	0.00–3.03
	Apr 19 to Apr 25, 2021	29	0–0	0.00–0.00	0–3	0.00–9.38
	Apr 26 to May 2, 2021	36	0–5	0.00–16.13	0–0	0.00–0.00
	May 3 to May 9, 2021	39	0–7	0.00–21.88	0–0	0.00–0.00
	May 10 to May 16, 2021	41	0–9	0.00–28.13	0–0	0.00–0.00
	May 17 to May 23, 2021	39	0–8	0.00–25.81	0–0	0.00–0.00
	May 24 to May 30, 2021	27	0–0	0.00–0.00	0–5	0.00–15.63
Iwate						
	Apr 5 to Apr 11, 2021	39	0–1	0.00–2.63	0–0	0.00–0.00
	Apr 12 to Apr 18, 2021	31	0–0	0.00–0.00	0–7	0.00–18.42
	Apr 19 to Apr 25, 2021	30	0–0	0.00–0.00	0–7	0.00–18.92
	Apr 26 to May 2, 2021	23	0–0	0.00–0.00	2–14	5.41–37.84
	May 3 to May 9, 2021	31	0–0	0.00–0.00	0–5	0.00–13.89
	May 10 to May 16, 2021	29	0–0	0.00–0.00	0–7	0.00–19.44
	May 17 to May 23, 2021	35	0–0	0.00–0.00	0–1	0.00–2.78
	May 24 to May 30, 2021	26	0–0	0.00–0.00	0–10	0.00–27.78
Miyagi						
	Apr 5 to Apr 11, 2021	54	0–4	0.00–8.00	0–0	0.00–0.00
	Apr 12 to Apr 18, 2021	76	9–24	17.31–46.15	0–0	0.00–0.00
	Apr 19 to Apr 25, 2021	60	0–8	0.00–15.38	0–0	0.00–0.00
	Apr 26 to May 2, 2021	53	0–0	0.00–0.00	0–0	0.00–0.00
	May 3 to May 9, 2021	57	0–5	0.00–9.62	0–0	0.00–0.00

	May 10 to May 16, 2021	58	0–8	0.00–16.00	0–0	0.00–0.00
	May 17 to May 23, 2021	50	0–2	0.00–4.17	0–0	0.00–0.00
	May 24 to May 30, 2021	54	0–7	0.00–14.89	0–0	0.00–0.00
Akita						
	Apr 5 to Apr 11, 2021	27	0–0	0.00–0.00	0–4	0.00–12.90
	Apr 12 to Apr 18, 2021	38	0–8	0.00–26.67	0–0	0.00–0.00
	Apr 19 to Apr 25, 2021	30	0–0	0.00–0.00	0–0	0.00–0.00
	Apr 26 to May 2, 2021	34	0–4	0.00–13.33	0–0	0.00–0.00
	May 3 to May 9, 2021	42	2–13	6.90–44.83	0–0	0.00–0.00
	May 10 to May 16, 2021	35	0–7	0.00–25.00	0–0	0.00–0.00
	May 17 to May 23, 2021	22	0–0	0.00–0.00	0–7	0.00–24.14
	May 24 to May 30, 2021	29	0–0	0.00–0.00	0–0	0.00–0.00
Yamagata						
	Apr 5 to Apr 11, 2021	32	0–0	0.00–0.00	0–7	0.00–17.95
	Apr 12 to Apr 18, 2021	34	0–0	0.00–0.00	0–4	0.00–10.53
	Apr 19 to Apr 25, 2021	34	0–0	0.00–0.00	0–3	0.00–8.11
	Apr 26 to May 2, 2021	42	0–5	0.00–13.51	0–0	0.00–0.00
	May 3 to May 9, 2021	46	0–9	0.00–24.32	0–0	0.00–0.00
	May 10 to May 16, 2021	47	0–10	0.00–27.03	0–0	0.00–0.00
	May 17 to May 23, 2021	49	0–12	0.00–32.43	0–0	0.00–0.00
	May 24 to May 30, 2021	56	7–19	18.92–51.35	0–0	0.00–0.00
Fukushima						
	Apr 5 to Apr 11, 2021	63	0–10	0.00–18.87	0–0	0.00–0.00
	Apr 12 to Apr 18, 2021	59	0–7	0.00–13.46	0–0	0.00–0.00
	Apr 19 to Apr 25, 2021	53	0–1	0.00–1.92	0–0	0.00–0.00
	Apr 26 to May 2, 2021	58	0–6	0.00–11.54	0–0	0.00–0.00
	May 3 to May 9, 2021	47	0–0	0.00–0.00	0–4	0.00–7.84
	May 10 to May 16, 2021	69	5–19	10.00–38.00	0–0	0.00–0.00
	May 17 to May 23, 2021	45	0–0	0.00–0.00	0–4	0.00–8.16
	May 24 to May 30, 2021	45	0–0	0.00–0.00	0–4	0.00–8.16
Ibaraki						
	Apr 5 to Apr 11, 2021	59	0–0	0.00–0.00	0–4	0.00–6.35
	Apr 12 to Apr 18, 2021	68	0–6	0.00–9.68	0–0	0.00–0.00
	Apr 19 to Apr 25, 2021	58	0–0	0.00–0.00	0–3	0.00–4.92
	Apr 26 to May 2, 2021	60	0–1	0.00–1.69	0–0	0.00–0.00
	May 3 to May 9, 2021	65	0–8	0.00–14.04	0–0	0.00–0.00
	May 10 to May 16, 2021	70	0–14	0.00–25.00	0–0	0.00–0.00
	May 17 to May 23, 2021	48	0–0	0.00–0.00	0–7	0.00–12.73
	May 24 to May 30, 2021	78	5–22	8.93–39.29	0–0	0.00–0.00
Tochigi						
	Apr 5 to Apr 11, 2021	50	0–6	0.00–13.64	0–0	0.00–0.00
	Apr 12 to Apr 18, 2021	54	0–10	0.00–22.73	0–0	0.00–0.00
	Apr 19 to Apr 25, 2021	52	0–9	0.00–20.93	0–0	0.00–0.00
	Apr 26 to May 2, 2021	43	0–2	0.00–4.88	0–0	0.00–0.00
	May 3 to May 9, 2021	34	0–0	0.00–0.00	0–6	0.00–15.00
	May 10 to May 16, 2021	41	0–2	0.00–5.13	0–0	0.00–0.00

	May 17 to May 23, 2021	35	0–0	0.00–0.00	0–4	0.00–10.26
	May 24 to May 30, 2021	42	0–4	0.00–10.53	0–0	0.00–0.00
Gunma						
	Apr 5 to Apr 11, 2021	35	0–0	0.00–0.00	0–5	0.00–12.50
	Apr 12 to Apr 18, 2021	49	0–10	0.00–25.64	0–0	0.00–0.00
	Apr 19 to Apr 25, 2021	58	8–21	21.62–56.76	0–0	0.00–0.00
	Apr 26 to May 2, 2021	46	0–9	0.00–24.32	0–0	0.00–0.00
	May 3 to May 9, 2021	52	2–15	5.41–40.54	0–0	0.00–0.00
	May 10 to May 16, 2021	28	0–0	0.00–0.00	0–7	0.00–20.00
	May 17 to May 23, 2021	32	0–0	0.00–0.00	0–3	0.00–8.57
	May 24 to May 30, 2021	43	0–6	0.00–16.22	0–0	0.00–0.00
Saitama						
	Apr 5 to Apr 11, 2021	126	0–8	0.00–6.78	0–0	0.00–0.00
	Apr 12 to Apr 18, 2021	157	19–40	16.24–34.19	0–0	0.00–0.00
	Apr 19 to Apr 25, 2021	127	0–12	0.00–10.43	0–0	0.00–0.00
	Apr 26 to May 2, 2021	144	9–31	7.96–27.43	0–0	0.00–0.00
	May 3 to May 9, 2021	123	0–10	0.00–8.85	0–0	0.00–0.00
	May 10 to May 16, 2021	130	0–20	0.00–18.18	0–0	0.00–0.00
	May 17 to May 23, 2021	102	0–0	0.00–0.00	0–5	0.00–4.67
	May 24 to May 30, 2021	116	0–9	0.00–8.41	0–0	0.00–0.00
Chiba						
	Apr 5 to Apr 11, 2021	121	0–5	0.00–4.31	0–0	0.00–0.00
	Apr 12 to Apr 18, 2021	110	0–0	0.00–0.00	0–5	0.00–4.35
	Apr 19 to Apr 25, 2021	116	0–3	0.00–2.65	0–0	0.00–0.00
	Apr 26 to May 2, 2021	128	0–19	0.00–17.43	0–0	0.00–0.00
	May 3 to May 9, 2021	126	0–17	0.00–15.60	0–0	0.00–0.00
	May 10 to May 16, 2021	127	0–17	0.00–15.45	0–0	0.00–0.00
	May 17 to May 23, 2021	112	0–2	0.00–1.82	0–0	0.00–0.00
	May 24 to May 30, 2021	110	0–0	0.00–0.00	0–0	0.00–0.00
Tokyo						
	Apr 5 to Apr 11, 2021	255	0–21	0.00–8.97	0–0	0.00–0.00
	Apr 12 to Apr 18, 2021	259	0–27	0.00–11.64	0–0	0.00–0.00
	Apr 19 to Apr 25, 2021	268	7–39	3.06–17.03	0–0	0.00–0.00
	Apr 26 to May 2, 2021	270	14–45	6.22–20.00	0–0	0.00–0.00
	May 3 to May 9, 2021	255	2–33	0.90–14.86	0–0	0.00–0.00
	May 10 to May 16, 2021	268	18–49	8.22–22.37	0–0	0.00–0.00
	May 17 to May 23, 2021	243	0–28	0.00–13.02	0–0	0.00–0.00
	May 24 to May 30, 2021	243	0–28	0.00–13.02	0–0	0.00–0.00
Kanagawa						
	Apr 5 to Apr 11, 2021	184	0–0	0.00–0.00	0–9	0.00–4.66
	Apr 12 to Apr 18, 2021	225	5–34	2.62–17.80	0–0	0.00–0.00
	Apr 19 to Apr 25, 2021	187	0–0	0.00–0.00	0–2	0.00–1.06
	Apr 26 to May 2, 2021	226	10–39	5.35–20.86	0–0	0.00–0.00
	May 3 to May 9, 2021	222	10–38	5.43–20.65	0–0	0.00–0.00
	May 10 to May 16, 2021	225	11–40	5.95–21.62	0–0	0.00–0.00
	May 17 to May 23, 2021	201	0–19	0.00–10.44	0–0	0.00–0.00

	May 24 to May 30, 2021	226	20–48	11.24–26.97	0–0	0.00–0.00
Niigata						
	Apr 5 to Apr 11, 2021	66	0–0	0.00–0.00	0–9	0.00–12.00
	Apr 12 to Apr 18, 2021	87	0–15	0.00–20.83	0–0	0.00–0.00
	Apr 19 to Apr 25, 2021	76	0–5	0.00–7.04	0–0	0.00–0.00
	Apr 26 to May 2, 2021	69	0–0	0.00–0.00	0–1	0.00–1.43
	May 3 to May 9, 2021	77	0–6	0.00–8.45	0–0	0.00–0.00
	May 10 to May 16, 2021	69	0–0	0.00–0.00	0–2	0.00–2.82
	May 17 to May 23, 2021	62	0–0	0.00–0.00	0–8	0.00–11.43
	May 24 to May 30, 2021	65	0–0	0.00–0.00	0–3	0.00–4.41
Toyama						
	Apr 5 to Apr 11, 2021	22	0–0	0.00–0.00	0–9	0.00–29.03
	Apr 12 to Apr 18, 2021	24	0–0	0.00–0.00	0–5	0.00–17.24
	Apr 19 to Apr 25, 2021	35	0–7	0.00–25.00	0–0	0.00–0.00
	Apr 26 to May 2, 2021	38	0–10	0.00–35.71	0–0	0.00–0.00
	May 3 to May 9, 2021	31	0–4	0.00–14.81	0–0	0.00–0.00
	May 10 to May 16, 2021	27	0–0	0.00–0.00	0–0	0.00–0.00
	May 17 to May 23, 2021	26	0–0	0.00–0.00	0–0	0.00–0.00
	May 24 to May 30, 2021	23	0–0	0.00–0.00	0–3	0.00–11.54
Ishikawa						
	Apr 5 to Apr 11, 2021	23	0–0	0.00–0.00	0–1	0.00–4.17
	Apr 12 to Apr 18, 2021	24	0–1	0.00–4.35	0–0	0.00–0.00
	Apr 19 to Apr 25, 2021	26	0–4	0.00–18.18	0–0	0.00–0.00
	Apr 26 to May 2, 2021	14	0–0	0.00–0.00	0–8	0.00–36.36
	May 3 to May 9, 2021	22	0–0	0.00–0.00	0–0	0.00–0.00
	May 10 to May 16, 2021	23	0–1	0.00–4.55	0–0	0.00–0.00
	May 17 to May 23, 2021	21	0–0	0.00–0.00	0–1	0.00–4.55
	May 24 to May 30, 2021	21	0–0	0.00–0.00	0–0	0.00–0.00
Fukui						
	Apr 5 to Apr 11, 2021	17	0–0	0.00–0.00	0–0	0.00–0.00
	Apr 12 to Apr 18, 2021	17	0–1	0.00–6.25	0–0	0.00–0.00
	Apr 19 to Apr 25, 2021	25	1–9	6.25–56.25	0–0	0.00–0.00
	Apr 26 to May 2, 2021	16	0–0	0.00–0.00	0–0	0.00–0.00
	May 3 to May 9, 2021	25	1–9	6.25–56.25	0–0	0.00–0.00
	May 10 to May 16, 2021	20	0–4	0.00–25.00	0–0	0.00–0.00
	May 17 to May 23, 2021	23	0–8	0.00–53.33	0–0	0.00–0.00
	May 24 to May 30, 2021	18	0–2	0.00–12.50	0–0	0.00–0.00
Yamanashi						
	Apr 5 to Apr 11, 2021	26	0–5	0.00–23.81	0–0	0.00–0.00
	Apr 12 to Apr 18, 2021	21	0–0	0.00–0.00	0–0	0.00–0.00
	Apr 19 to Apr 25, 2021	21	0–1	0.00–5.00	0–0	0.00–0.00
	Apr 26 to May 2, 2021	27	0–8	0.00–42.11	0–0	0.00–0.00
	May 3 to May 9, 2021	23	0–5	0.00–27.78	0–0	0.00–0.00
	May 10 to May 16, 2021	20	0–2	0.00–11.11	0–0	0.00–0.00
	May 17 to May 23, 2021	21	0–3	0.00–16.67	0–0	0.00–0.00
	May 24 to May 30, 2021	20	0–3	0.00–17.65	0–0	0.00–0.00

Nagano						
	Apr 5 to Apr 11, 2021	67	0–1	0.00–1.52	0–0	0.00–0.00
	Apr 12 to Apr 18, 2021	63	0–0	0.00–0.00	0–0	0.00–0.00
	Apr 19 to Apr 25, 2021	65	0–3	0.00–4.84	0–0	0.00–0.00
	Apr 26 to May 2, 2021	81	2–18	3.17–28.57	0–0	0.00–0.00
	May 3 to May 9, 2021	69	0–6	0.00–9.52	0–0	0.00–0.00
	May 10 to May 16, 2021	64	0–4	0.00–6.67	0–0	0.00–0.00
	May 17 to May 23, 2021	83	8–24	13.56–40.68	0–0	0.00–0.00
	May 24 to May 30, 2021	60	0–3	0.00–5.26	0–0	0.00–0.00
Gifu						
	Apr 5 to Apr 11, 2021	57	0–4	0.00–7.55	0–0	0.00–0.00
	Apr 12 to Apr 18, 2021	60	0–8	0.00–15.38	0–0	0.00–0.00
	Apr 19 to Apr 25, 2021	48	0–0	0.00–0.00	0–4	0.00–7.69
	Apr 26 to May 2, 2021	55	0–5	0.00–10.00	0–0	0.00–0.00
	May 3 to May 9, 2021	54	0–6	0.00–12.50	0–0	0.00–0.00
	May 10 to May 16, 2021	53	0–7	0.00–15.22	0–0	0.00–0.00
	May 17 to May 23, 2021	65	7–21	15.91–47.73	0–0	0.00–0.00
	May 24 to May 30, 2021	44	0–0	0.00–0.00	0–1	0.00–2.22
Shizuoka						
	Apr 5 to Apr 11, 2021	125	0–7	0.00–5.93	0–0	0.00–0.00
	Apr 12 to Apr 18, 2021	123	0–9	0.00–7.89	0–0	0.00–0.00
	Apr 19 to Apr 25, 2021	124	0–11	0.00–9.73	0–0	0.00–0.00
	Apr 26 to May 2, 2021	136	1–24	0.89–21.43	0–0	0.00–0.00
	May 3 to May 9, 2021	136	5–27	4.59–24.77	0–0	0.00–0.00
	May 10 to May 16, 2021	109	0–1	0.00–0.93	0–0	0.00–0.00
	May 17 to May 23, 2021	122	0–17	0.00–16.19	0–0	0.00–0.00
	May 24 to May 30, 2021	102	0–0	0.00–0.00	0–3	0.00–2.86
Aichi						
	Apr 5 to Apr 11, 2021	153	0–0	0.00–0.00	0–8	0.00–4.97
	Apr 12 to Apr 18, 2021	186	0–26	0.00–16.25	0–0	0.00–0.00
	Apr 19 to Apr 25, 2021	174	0–18	0.00–11.54	0–0	0.00–0.00
	Apr 26 to May 2, 2021	170	0–16	0.00–10.39	0–0	0.00–0.00
	May 3 to May 9, 2021	168	0–15	0.00–9.80	0–0	0.00–0.00
	May 10 to May 16, 2021	173	0–23	0.00–15.33	0–0	0.00–0.00
	May 17 to May 23, 2021	147	0–0	0.00–0.00	0–1	0.00–0.68
	May 24 to May 30, 2021	157	0–12	0.00–8.28	0–0	0.00–0.00
Mie						
	Apr 5 to Apr 11, 2021	52	0–0	0.00–0.00	0–3	0.00–5.45
	Apr 12 to Apr 18, 2021	50	0–0	0.00–0.00	0–3	0.00–5.66
	Apr 19 to Apr 25, 2021	60	0–8	0.00–15.38	0–0	0.00–0.00
	Apr 26 to May 2, 2021	56	0–4	0.00–7.69	0–0	0.00–0.00
	May 3 to May 9, 2021	65	0–14	0.00–27.45	0–0	0.00–0.00
	May 10 to May 16, 2021	57	0–5	0.00–9.62	0–0	0.00–0.00
	May 17 to May 23, 2021	76	10–25	19.61–49.02	0–0	0.00–0.00
	May 24 to May 30, 2021	52	0–2	0.00–4.00	0–0	0.00–0.00
Shiga						

	Apr 5 to Apr 11, 2021	23	0–0	0.00–0.00	0–2	0.00–8.00
	Apr 12 to Apr 18, 2021	28	0–3	0.00–12.00	0–0	0.00–0.00
	Apr 19 to Apr 25, 2021	24	0–0	0.00–0.00	0–1	0.00–4.00
	Apr 26 to May 2, 2021	36	2–12	8.33–50.00	0–0	0.00–0.00
	May 3 to May 9, 2021	42	9–19	39.13–82.61	0–0	0.00–0.00
	May 10 to May 16, 2021	33	0–10	0.00–43.48	0–0	0.00–0.00
	May 17 to May 23, 2021	21	0–0	0.00–0.00	0–3	0.00–12.50
	May 24 to May 30, 2021	32	0–8	0.00–33.33	0–0	0.00–0.00
Kyoto						
	Apr 5 to Apr 11, 2021	52	0–2	0.00–4.00	0–0	0.00–0.00
	Apr 12 to Apr 18, 2021	65	0–16	0.00–32.65	0–0	0.00–0.00
	Apr 19 to Apr 25, 2021	59	0–10	0.00–20.41	0–0	0.00–0.00
	Apr 26 to May 2, 2021	52	0–3	0.00–6.12	0–0	0.00–0.00
	May 3 to May 9, 2021	68	4–20	8.33–41.67	0–0	0.00–0.00
	May 10 to May 16, 2021	45	0–0	0.00–0.00	0–3	0.00–6.25
	May 17 to May 23, 2021	48	0–2	0.00–4.35	0–0	0.00–0.00
	May 24 to May 30, 2021	50	0–5	0.00–11.11	0–0	0.00–0.00
Osaka						
	Apr 5 to Apr 11, 2021	131	0–0	0.00–0.00	0–4	0.00–2.96
	Apr 12 to Apr 18, 2021	155	0–24	0.00–18.32	0–0	0.00–0.00
	Apr 19 to Apr 25, 2021	143	0–15	0.00–11.72	0–0	0.00–0.00
	Apr 26 to May 2, 2021	155	2–28	1.57–22.05	0–0	0.00–0.00
	May 3 to May 9, 2021	133	0–8	0.00–6.40	0–0	0.00–0.00
	May 10 to May 16, 2021	166	17–42	13.71–33.87	0–0	0.00–0.00
	May 17 to May 23, 2021	149	0–26	0.00–21.14	0–0	0.00–0.00
	May 24 to May 30, 2021	125	0–2	0.00–1.63	0–0	0.00–0.00
Hyogo						
	Apr 5 to Apr 11, 2021	113	0–5	0.00–4.63	0–0	0.00–0.00
	Apr 12 to Apr 18, 2021	114	0–7	0.00–6.54	0–0	0.00–0.00
	Apr 19 to Apr 25, 2021	133	8–29	7.69–27.88	0–0	0.00–0.00
	Apr 26 to May 2, 2021	130	6–27	5.83–26.21	0–0	0.00–0.00
	May 3 to May 9, 2021	128	5–26	4.90–25.49	0–0	0.00–0.00
	May 10 to May 16, 2021	120	0–18	0.00–17.65	0–0	0.00–0.00
	May 17 to May 23, 2021	130	9–30	9.00–30.00	0–0	0.00–0.00
	May 24 to May 30, 2021	96	0–0	0.00–0.00	0–2	0.00–2.04
Nara						
	Apr 5 to Apr 11, 2021	26	0–0	0.00–0.00	0–4	0.00–13.33
	Apr 12 to Apr 18, 2021	26	0–0	0.00–0.00	0–4	0.00–13.33
	Apr 19 to Apr 25, 2021	34	0–4	0.00–13.33	0–0	0.00–0.00
	Apr 26 to May 2, 2021	32	0–2	0.00–6.67	0–0	0.00–0.00
	May 3 to May 9, 2021	21	0–0	0.00–0.00	0–6	0.00–22.22
	May 10 to May 16, 2021	34	0–7	0.00–25.93	0–0	0.00–0.00
	May 17 to May 23, 2021	41	4–14	14.81–51.85	0–0	0.00–0.00
	May 24 to May 30, 2021	36	0–10	0.00–38.46	0–0	0.00–0.00
Wakayama						
	Apr 5 to Apr 11, 2021	23	0–0	0.00–0.00	0–7	0.00–23.33

	Apr 12 to Apr 18, 2021	35	0–6	0.00–20.69	0–0	0.00–0.00
	Apr 19 to Apr 25, 2021	31	0–3	0.00–10.71	0–0	0.00–0.00
	Apr 26 to May 2, 2021	24	0–0	0.00–0.00	0–3	0.00–11.11
	May 3 to May 9, 2021	31	0–3	0.00–10.71	0–0	0.00–0.00
	May 10 to May 16, 2021	31	0–3	0.00–10.71	0–0	0.00–0.00
	May 17 to May 23, 2021	30	0–2	0.00–7.14	0–0	0.00–0.00
	May 24 to May 30, 2021	29	0–0	0.00–0.00	0–0	0.00–0.00
Tottori						
	Apr 5 to Apr 11, 2021	17	0–0	0.00–0.00	0–3	0.00–15.00
	Apr 12 to Apr 18, 2021	23	0–4	0.00–21.05	0–0	0.00–0.00
	Apr 19 to Apr 25, 2021	18	0–0	0.00–0.00	0–0	0.00–0.00
	Apr 26 to May 2, 2021	12	0–0	0.00–0.00	0–6	0.00–33.33
	May 3 to May 9, 2021	25	0–7	0.00–38.89	0–0	0.00–0.00
	May 10 to May 16, 2021	22	0–3	0.00–15.79	0–0	0.00–0.00
	May 17 to May 23, 2021	26	0–8	0.00–44.44	0–0	0.00–0.00
	May 24 to May 30, 2021	23	0–5	0.00–27.78	0–0	0.00–0.00
Shimane						
	Apr 5 to Apr 11, 2021	24	0–2	0.00–9.09	0–0	0.00–0.00
	Apr 12 to Apr 18, 2021	29	0–7	0.00–31.82	0–0	0.00–0.00
	Apr 19 to Apr 25, 2021	24	0–3	0.00–14.29	0–0	0.00–0.00
	Apr 26 to May 2, 2021	24	0–3	0.00–14.29	0–0	0.00–0.00
	May 3 to May 9, 2021	19	0–0	0.00–0.00	0–0	0.00–0.00
	May 10 to May 16, 2021	22	0–3	0.00–15.79	0–0	0.00–0.00
	May 17 to May 23, 2021	22	0–4	0.00–22.22	0–0	0.00–0.00
	May 24 to May 30, 2021	22	0–4	0.00–22.22	0–0	0.00–0.00
Okayama						
	Apr 5 to Apr 11, 2021	41	0–0	0.00–0.00	0–0	0.00–0.00
	Apr 12 to Apr 18, 2021	48	0–9	0.00–23.08	0–0	0.00–0.00
	Apr 19 to Apr 25, 2021	37	0–0	0.00–0.00	0–1	0.00–2.63
	Apr 26 to May 2, 2021	40	0–2	0.00–5.26	0–0	0.00–0.00
	May 3 to May 9, 2021	40	0–3	0.00–8.11	0–0	0.00–0.00
	May 10 to May 16, 2021	42	0–5	0.00–13.51	0–0	0.00–0.00
	May 17 to May 23, 2021	43	0–6	0.00–16.22	0–0	0.00–0.00
	May 24 to May 30, 2021	44	0–7	0.00–18.92	0–0	0.00–0.00
Hiroshima						
	Apr 5 to Apr 11, 2021	59	0–0	0.00–0.00	0–4	0.00–6.35
	Apr 12 to Apr 18, 2021	56	0–0	0.00–0.00	0–5	0.00–8.20
	Apr 19 to Apr 25, 2021	58	0–0	0.00–0.00	0–1	0.00–1.69
	Apr 26 to May 2, 2021	75	2–18	3.51–31.58	0–0	0.00–0.00
	May 3 to May 9, 2021	76	6–21	10.91–38.18	0–0	0.00–0.00
	May 10 to May 16, 2021	60	0–7	0.00–13.21	0–0	0.00–0.00
	May 17 to May 23, 2021	63	0–11	0.00–21.15	0–0	0.00–0.00
	May 24 to May 30, 2021	56	0–5	0.00–9.80	0–0	0.00–0.00
Yamaguchi						
	Apr 5 to Apr 11, 2021	35	0–2	0.00–6.06	0–0	0.00–0.00
	Apr 12 to Apr 18, 2021	44	0–12	0.00–37.50	0–0	0.00–0.00

	Apr 19 to Apr 25, 2021	32	0–0	0.00–0.00	0–0	0.00–0.00
	Apr 26 to May 2, 2021	38	0–6	0.00–18.75	0–0	0.00–0.00
	May 3 to May 9, 2021	37	0–5	0.00–15.63	0–0	0.00–0.00
	May 10 to May 16, 2021	21	0–0	0.00–0.00	0–11	0.00–34.38
	May 17 to May 23, 2021	45	2–13	6.25–40.63	0–0	0.00–0.00
	May 24 to May 30, 2021	26	0–0	0.00–0.00	0–5	0.00–16.13
Tokushima						
	Apr 5 to Apr 11, 2021	19	0–0	0.00–0.00	0–1	0.00–5.00
	Apr 12 to Apr 18, 2021	25	0–5	0.00–25.00	0–0	0.00–0.00
	Apr 19 to Apr 25, 2021	19	0–0	0.00–0.00	0–0	0.00–0.00
	Apr 26 to May 2, 2021	15	0–0	0.00–0.00	0–4	0.00–21.05
	May 3 to May 9, 2021	21	0–1	0.00–5.00	0–0	0.00–0.00
	May 10 to May 16, 2021	16	0–0	0.00–0.00	0–4	0.00–20.00
	May 17 to May 23, 2021	23	0–4	0.00–21.05	0–0	0.00–0.00
	May 24 to May 30, 2021	16	0–0	0.00–0.00	0–4	0.00–20.00
Kagawa						
	Apr 5 to Apr 11, 2021	26	0–0	0.00–0.00	0–5	0.00–16.13
	Apr 12 to Apr 18, 2021	34	0–3	0.00–9.68	0–0	0.00–0.00
	Apr 19 to Apr 25, 2021	28	0–0	0.00–0.00	0–2	0.00–6.67
	Apr 26 to May 2, 2021	45	3–15	10.00–50.00	0–0	0.00–0.00
	May 3 to May 9, 2021	32	0–2	0.00–6.67	0–0	0.00–0.00
	May 10 to May 16, 2021	31	0–2	0.00–6.90	0–0	0.00–0.00
	May 17 to May 23, 2021	38	0–9	0.00–31.03	0–0	0.00–0.00
	May 24 to May 30, 2021	33	0–5	0.00–17.86	0–0	0.00–0.00
Ehime						
	Apr 5 to Apr 11, 2021	44	0–4	0.00–10.00	0–0	0.00–0.00
	Apr 12 to Apr 18, 2021	44	0–6	0.00–15.79	0–0	0.00–0.00
	Apr 19 to Apr 25, 2021	44	0–6	0.00–15.79	0–0	0.00–0.00
	Apr 26 to May 2, 2021	51	0–13	0.00–34.21	0–0	0.00–0.00
	May 3 to May 9, 2021	47	0–9	0.00–23.68	0–0	0.00–0.00
	May 10 to May 16, 2021	47	0–8	0.00–20.51	0–0	0.00–0.00
	May 17 to May 23, 2021	39	0–2	0.00–5.41	0–0	0.00–0.00
	May 24 to May 30, 2021	34	0–0	0.00–0.00	0–2	0.00–5.56
Kochi						
	Apr 5 to Apr 11, 2021	20	0–3	0.00–17.65	0–0	0.00–0.00
	Apr 12 to Apr 18, 2021	17	0–0	0.00–0.00	0–0	0.00–0.00
	Apr 19 to Apr 25, 2021	30	5–13	29.41–76.47	0–0	0.00–0.00
	Apr 26 to May 2, 2021	14	0–0	0.00–0.00	0–2	0.00–12.50
	May 3 to May 9, 2021	18	0–2	0.00–12.50	0–0	0.00–0.00
	May 10 to May 16, 2021	7	0–0	0.00–0.00	2–9	12.50–56.25
	May 17 to May 23, 2021	21	0–5	0.00–31.25	0–0	0.00–0.00
	May 24 to May 30, 2021	20	0–3	0.00–17.65	0–0	0.00–0.00
Fukuoka						
	Apr 5 to Apr 11, 2021	73	0–0	0.00–0.00	0–1	0.00–1.35
	Apr 12 to Apr 18, 2021	76	0–2	0.00–2.70	0–0	0.00–0.00
	Apr 19 to Apr 25, 2021	80	0–8	0.00–11.11	0–0	0.00–0.00

	Apr 26 to May 2, 2021	81	0–10	0.00–14.08	0–0	0.00–0.00
	May 3 to May 9, 2021	86	0–16	0.00–22.86	0–0	0.00–0.00
	May 10 to May 16, 2021	104	17–35	24.64–50.72	0–0	0.00–0.00
	May 17 to May 23, 2021	72	0–3	0.00–4.35	0–0	0.00–0.00
	May 24 to May 30, 2021	71	0–2	0.00–2.90	0–0	0.00–0.00
Saga						
	Apr 5 to Apr 11, 2021	21	0–3	0.00–16.67	0–0	0.00–0.00
	Apr 12 to Apr 18, 2021	20	0–3	0.00–17.65	0–0	0.00–0.00
	Apr 19 to Apr 25, 2021	12	0–0	0.00–0.00	0–5	0.00–29.41
	Apr 26 to May 2, 2021	14	0–0	0.00–0.00	0–3	0.00–17.65
	May 3 to May 9, 2021	24	0–7	0.00–41.18	0–0	0.00–0.00
	May 10 to May 16, 2021	16	0–0	0.00–0.00	0–1	0.00–5.88
	May 17 to May 23, 2021	16	0–0	0.00–0.00	0–0	0.00–0.00
	May 24 to May 30, 2021	19	0–3	0.00–18.75	0–0	0.00–0.00
Nagasaki						
	Apr 5 to Apr 11, 2021	38	0–10	0.00–35.71	0–0	0.00–0.00
	Apr 12 to Apr 18, 2021	28	0–1	0.00–3.70	0–0	0.00–0.00
	Apr 19 to Apr 25, 2021	35	0–9	0.00–34.62	0–0	0.00–0.00
	Apr 26 to May 2, 2021	34	0–9	0.00–36.00	0–0	0.00–0.00
	May 3 to May 9, 2021	39	5–15	20.83–62.50	0–0	0.00–0.00
	May 10 to May 16, 2021	31	0–6	0.00–24.00	0–0	0.00–0.00
	May 17 to May 23, 2021	32	0–8	0.00–33.33	0–0	0.00–0.00
	May 24 to May 30, 2021	46	12–22	50.00–91.67	0–0	0.00–0.00
Kumamoto						
	Apr 5 to Apr 11, 2021	40	0–0	0.00–0.00	0–0	0.00–0.00
	Apr 12 to Apr 18, 2021	50	0–11	0.00–28.21	0–0	0.00–0.00
	Apr 19 to Apr 25, 2021	39	0–1	0.00–2.63	0–0	0.00–0.00
	Apr 26 to May 2, 2021	39	0–1	0.00–2.63	0–0	0.00–0.00
	May 3 to May 9, 2021	63	12–25	31.58–65.79	0–0	0.00–0.00
	May 10 to May 16, 2021	45	0–7	0.00–18.42	0–0	0.00–0.00
	May 17 to May 23, 2021	42	0–4	0.00–10.53	0–0	0.00–0.00
	May 24 to May 30, 2021	50	0–11	0.00–28.21	0–0	0.00–0.00
Oita						
	Apr 5 to Apr 11, 2021	25	0–0	0.00–0.00	0–4	0.00–13.79
	Apr 12 to Apr 18, 2021	25	0–0	0.00–0.00	0–5	0.00–16.67
	Apr 19 to Apr 25, 2021	27	0–0	0.00–0.00	0–2	0.00–6.90
	Apr 26 to May 2, 2021	35	0–7	0.00–25.00	0–0	0.00–0.00
	May 3 to May 9, 2021	26	0–0	0.00–0.00	0–1	0.00–3.70
	May 10 to May 16, 2021	34	0–7	0.00–25.93	0–0	0.00–0.00
	May 17 to May 23, 2021	24	0–0	0.00–0.00	0–3	0.00–11.11
	May 24 to May 30, 2021	27	0–1	0.00–3.85	0–0	0.00–0.00
Miyazaki						
	Apr 5 to Apr 11, 2021	29	0–6	0.00–26.09	0–0	0.00–0.00
	Apr 12 to Apr 18, 2021	24	0–1	0.00–4.35	0–0	0.00–0.00
	Apr 19 to Apr 25, 2021	36	4–13	17.39–56.52	0–0	0.00–0.00
	Apr 26 to May 2, 2021	23	0–1	0.00–4.55	0–0	0.00–0.00

	May 3 to May 9, 2021	33	3–12	14.29–57.14	0–0	0.00–0.00
	May 10 to May 16, 2021	23	0–2	0.00–9.52	0–0	0.00–0.00
	May 17 to May 23, 2021	25	0–4	0.00–19.05	0–0	0.00–0.00
	May 24 to May 30, 2021	27	0–6	0.00–28.57	0–0	0.00–0.00
Kagoshima						
	Apr 5 to Apr 11, 2021	41	0–1	0.00–2.50	0–0	0.00–0.00
	Apr 12 to Apr 18, 2021	47	0–8	0.00–20.51	0–0	0.00–0.00
	Apr 19 to Apr 25, 2021	36	0–0	0.00–0.00	0–2	0.00–5.26
	Apr 26 to May 2, 2021	35	0–0	0.00–0.00	0–3	0.00–7.89
	May 3 to May 9, 2021	34	0–0	0.00–0.00	0–4	0.00–10.53
	May 10 to May 16, 2021	38	0–0	0.00–0.00	0–0	0.00–0.00
	May 17 to May 23, 2021	43	0–5	0.00–13.16	0–0	0.00–0.00
	May 24 to May 30, 2021	44	0–5	0.00–12.82	0–0	0.00–0.00
Okinawa						
	Apr 5 to Apr 11, 2021	30	0–7	0.00–30.43	0–0	0.00–0.00
	Apr 12 to Apr 18, 2021	25	0–3	0.00–13.64	0–0	0.00–0.00
	Apr 19 to Apr 25, 2021	24	0–3	0.00–14.29	0–0	0.00–0.00
	Apr 26 to May 2, 2021	30	0–9	0.00–42.86	0–0	0.00–0.00
	May 3 to May 9, 2021	26	0–6	0.00–30.00	0–0	0.00–0.00
	May 10 to May 16, 2021	30	0–9	0.00–42.86	0–0	0.00–0.00
	May 17 to May 23, 2021	21	0–1	0.00–5.00	0–0	0.00–0.00
	May 24 to May 30, 2021	32	2–12	10.00–60.00	0–0	0.00–0.00

Table A.18: Weekly number of observed and excess/exiguous deaths in Japan and 47 prefectures for senility-related deaths in hospitals and clinics from January 2020 through May 2021.

Prefecture	Week	Observed	Excess deaths	Percent excess	Exiguous deaths	Percent exiguous
Japan						
	Apr 5 to Apr 11, 2021	3565	0–47	0.00–1.34	0–0	0.00–0.00
	Apr 12 to Apr 18, 2021	3840	148–361	4.25–10.38	0–0	0.00–0.00
	Apr 19 to Apr 25, 2021	3725	48–274	1.39–7.94	0–0	0.00–0.00
	Apr 26 to May 2, 2021	3863	217–439	6.34–12.82	0–0	0.00–0.00
	May 3 to May 9, 2021	3856	278–485	8.25–14.39	0–0	0.00–0.00
	May 10 to May 16, 2021	3753	202–405	6.03–12.10	0–0	0.00–0.00
	May 17 to May 23, 2021	3641	133–328	4.01–9.90	0–0	0.00–0.00
	May 24 to May 30, 2021	3494	0–190	0.00–5.75	0–0	0.00–0.00
Hokkaido						
	Apr 5 to Apr 11, 2021	1278	0–76	0.00–6.32	0–0	0.00–0.00
	Apr 12 to Apr 18, 2021	1251	0–63	0.00–5.30	0–0	0.00–0.00
	Apr 19 to Apr 25, 2021	1198	0–23	0.00–1.96	0–0	0.00–0.00
	Apr 26 to May 2, 2021	1249	0–96	0.00–8.33	0–0	0.00–0.00
	May 3 to May 9, 2021	1230	5–100	0.44–8.85	0–0	0.00–0.00
	May 10 to May 16, 2021	1155	0–43	0.00–3.87	0–0	0.00–0.00
	May 17 to May 23, 2021	1083	0–0	0.00–0.00	0–18	0.00–1.63
	May 24 to May 30, 2021	1095	0–1	0.00–0.09	0–0	0.00–0.00
Aomori						
	Apr 5 to Apr 11, 2021	336	0–0	0.00–0.00	0–4	0.00–1.18
	Apr 12 to Apr 18, 2021	324	0–0	0.00–0.00	0–12	0.00–3.57
	Apr 19 to Apr 25, 2021	309	0–0	0.00–0.00	0–26	0.00–7.76
	Apr 26 to May 2, 2021	344	0–16	0.00–4.88	0–0	0.00–0.00
	May 3 to May 9, 2021	344	0–22	0.00–6.83	0–0	0.00–0.00
	May 10 to May 16, 2021	305	0–0	0.00–0.00	0–10	0.00–3.17
	May 17 to May 23, 2021	317	0–4	0.00–1.28	0–0	0.00–0.00
	May 24 to May 30, 2021	291	0–0	0.00–0.00	0–15	0.00–4.90
Iwate						
	Apr 5 to Apr 11, 2021	284	1–36	0.40–14.52	0–0	0.00–0.00
	Apr 12 to Apr 18, 2021	277	0–29	0.00–11.69	0–0	0.00–0.00
	Apr 19 to Apr 25, 2021	274	0–31	0.00–12.76	0–0	0.00–0.00
	Apr 26 to May 2, 2021	271	0–30	0.00–12.45	0–0	0.00–0.00
	May 3 to May 9, 2021	273	0–34	0.00–14.23	0–0	0.00–0.00
	May 10 to May 16, 2021	238	0–5	0.00–2.15	0–0	0.00–0.00
	May 17 to May 23, 2021	203	0–0	0.00–0.00	0–29	0.00–12.50
	May 24 to May 30, 2021	248	0–18	0.00–7.83	0–0	0.00–0.00
Miyagi						
	Apr 5 to Apr 11, 2021	401	0–0	0.00–0.00	0–6	0.00–1.47
	Apr 12 to Apr 18, 2021	418	0–19	0.00–4.76	0–0	0.00–0.00
	Apr 19 to Apr 25, 2021	409	0–21	0.00–5.41	0–0	0.00–0.00
	Apr 26 to May 2, 2021	429	5–49	1.32–12.89	0–0	0.00–0.00
	May 3 to May 9, 2021	400	0–26	0.00–6.95	0–0	0.00–0.00

	May 10 to May 16, 2021	410	0–40	0.00–10.81	0–0	0.00–0.00
	May 17 to May 23, 2021	417	10–55	2.76–15.19	0–0	0.00–0.00
	May 24 to May 30, 2021	356	0–0	0.00–0.00	0–4	0.00–1.11
Akita						
	Apr 5 to Apr 11, 2021	369	0–14	0.00–3.94	0–0	0.00–0.00
	Apr 12 to Apr 18, 2021	360	0–6	0.00–1.69	0–0	0.00–0.00
	Apr 19 to Apr 25, 2021	352	0–2	0.00–0.57	0–0	0.00–0.00
	Apr 26 to May 2, 2021	368	0–22	0.00–6.36	0–0	0.00–0.00
	May 3 to May 9, 2021	354	0–12	0.00–3.51	0–0	0.00–0.00
	May 10 to May 16, 2021	371	0–37	0.00–11.08	0–0	0.00–0.00
	May 17 to May 23, 2021	362	0–29	0.00–8.71	0–0	0.00–0.00
	May 24 to May 30, 2021	325	0–0	0.00–0.00	0–5	0.00–1.52
Yamagata						
	Apr 5 to Apr 11, 2021	271	0–6	0.00–2.26	0–0	0.00–0.00
	Apr 12 to Apr 18, 2021	262	0–0	0.00–0.00	0–2	0.00–0.76
	Apr 19 to Apr 25, 2021	307	16–48	6.18–18.53	0–0	0.00–0.00
	Apr 26 to May 2, 2021	271	0–13	0.00–5.04	0–0	0.00–0.00
	May 3 to May 9, 2021	288	0–32	0.00–12.50	0–0	0.00–0.00
	May 10 to May 16, 2021	264	0–12	0.00–4.76	0–0	0.00–0.00
	May 17 to May 23, 2021	263	0–13	0.00–5.20	0–0	0.00–0.00
	May 24 to May 30, 2021	248	0–1	0.00–0.40	0–0	0.00–0.00
Fukushima						
	Apr 5 to Apr 11, 2021	607	0–0	0.00–0.00	0–39	0.00–6.04
	Apr 12 to Apr 18, 2021	679	0–46	0.00–7.27	0–0	0.00–0.00
	Apr 19 to Apr 25, 2021	613	0–0	0.00–0.00	0–14	0.00–2.23
	Apr 26 to May 2, 2021	642	0–27	0.00–4.39	0–0	0.00–0.00
	May 3 to May 9, 2021	632	0–28	0.00–4.64	0–0	0.00–0.00
	May 10 to May 16, 2021	650	0–54	0.00–9.06	0–0	0.00–0.00
	May 17 to May 23, 2021	519	0–0	0.00–0.00	0–59	0.00–10.21
	May 24 to May 30, 2021	592	0–15	0.00–2.60	0–0	0.00–0.00
Ibaraki						
	Apr 5 to Apr 11, 2021	1666	0–40	0.00–2.46	0–0	0.00–0.00
	Apr 12 to Apr 18, 2021	1702	0–85	0.00–5.26	0–0	0.00–0.00
	Apr 19 to Apr 25, 2021	1675	0–81	0.00–5.08	0–0	0.00–0.00
	Apr 26 to May 2, 2021	1755	65–182	4.13–11.57	0–0	0.00–0.00
	May 3 to May 9, 2021	1722	59–174	3.81–11.24	0–0	0.00–0.00
	May 10 to May 16, 2021	1647	0–114	0.00–7.44	0–0	0.00–0.00
	May 17 to May 23, 2021	1634	6–120	0.40–7.93	0–0	0.00–0.00
	May 24 to May 30, 2021	1576	0–80	0.00–5.35	0–0	0.00–0.00
Tochigi						
	Apr 5 to Apr 11, 2021	193	0–5	0.00–2.66	0–0	0.00–0.00
	Apr 12 to Apr 18, 2021	199	0–12	0.00–6.42	0–0	0.00–0.00
	Apr 19 to Apr 25, 2021	209	0–25	0.00–13.59	0–0	0.00–0.00
	Apr 26 to May 2, 2021	196	0–14	0.00–7.69	0–0	0.00–0.00
	May 3 to May 9, 2021	177	0–0	0.00–0.00	0–1	0.00–0.56
	May 10 to May 16, 2021	193	0–16	0.00–9.04	0–0	0.00–0.00

	May 17 to May 23, 2021	194	0–20	0.00–11.49	0–0	0.00–0.00
	May 24 to May 30, 2021	203	1–31	0.58–18.02	0–0	0.00–0.00
Gunma						
	Apr 5 to Apr 11, 2021	447	0–0	0.00–0.00	0–2	0.00–0.45
	Apr 12 to Apr 18, 2021	523	31–84	7.06–19.13	0–0	0.00–0.00
	Apr 19 to Apr 25, 2021	452	0–17	0.00–3.91	0–0	0.00–0.00
	Apr 26 to May 2, 2021	454	0–29	0.00–6.82	0–0	0.00–0.00
	May 3 to May 9, 2021	450	0–26	0.00–6.13	0–0	0.00–0.00
	May 10 to May 16, 2021	453	0–39	0.00–9.42	0–0	0.00–0.00
	May 17 to May 23, 2021	385	0–0	0.00–0.00	0–24	0.00–5.87
	May 24 to May 30, 2021	443	0–35	0.00–8.58	0–0	0.00–0.00
Saitama						
	Apr 5 to Apr 11, 2021	346	0–0	0.00–0.00	0–15	0.00–4.16
	Apr 12 to Apr 18, 2021	382	0–28	0.00–7.91	0–0	0.00–0.00
	Apr 19 to Apr 25, 2021	395	2–45	0.57–12.86	0–0	0.00–0.00
	Apr 26 to May 2, 2021	390	2–43	0.58–12.39	0–0	0.00–0.00
	May 3 to May 9, 2021	369	0–28	0.00–8.21	0–0	0.00–0.00
	May 10 to May 16, 2021	325	0–0	0.00–0.00	0–13	0.00–3.85
	May 17 to May 23, 2021	363	0–28	0.00–8.36	0–0	0.00–0.00
	May 24 to May 30, 2021	363	0–36	0.00–11.01	0–0	0.00–0.00
Chiba						
	Apr 5 to Apr 11, 2021	499	0–17	0.00–3.53	0–0	0.00–0.00
	Apr 12 to Apr 18, 2021	551	22–72	4.59–15.03	0–0	0.00–0.00
	Apr 19 to Apr 25, 2021	469	0–0	0.00–0.00	0–3	0.00–0.64
	Apr 26 to May 2, 2021	492	0–21	0.00–4.46	0–0	0.00–0.00
	May 3 to May 9, 2021	489	0–25	0.00–5.39	0–0	0.00–0.00
	May 10 to May 16, 2021	436	0–0	0.00–0.00	0–21	0.00–4.60
	May 17 to May 23, 2021	434	0–0	0.00–0.00	0–15	0.00–3.34
	May 24 to May 30, 2021	457	0–17	0.00–3.86	0–0	0.00–0.00
Tokyo						
	Apr 5 to Apr 11, 2021	193	0–4	0.00–2.12	0–0	0.00–0.00
	Apr 12 to Apr 18, 2021	199	0–13	0.00–6.99	0–0	0.00–0.00
	Apr 19 to Apr 25, 2021	205	0–21	0.00–11.41	0–0	0.00–0.00
	Apr 26 to May 2, 2021	171	0–0	0.00–0.00	0–10	0.00–5.52
	May 3 to May 9, 2021	222	16–44	8.99–24.72	0–0	0.00–0.00
	May 10 to May 16, 2021	194	0–17	0.00–9.60	0–0	0.00–0.00
	May 17 to May 23, 2021	185	0–8	0.00–4.52	0–0	0.00–0.00
	May 24 to May 30, 2021	173	0–0	0.00–0.00	0–3	0.00–1.70
Kanagawa						
	Apr 5 to Apr 11, 2021	176	0–0	0.00–0.00	0–17	0.00–8.81
	Apr 12 to Apr 18, 2021	194	0–4	0.00–2.11	0–0	0.00–0.00
	Apr 19 to Apr 25, 2021	205	0–20	0.00–10.81	0–0	0.00–0.00
	Apr 26 to May 2, 2021	207	0–24	0.00–13.11	0–0	0.00–0.00
	May 3 to May 9, 2021	209	1–30	0.56–16.76	0–0	0.00–0.00
	May 10 to May 16, 2021	193	0–16	0.00–9.04	0–0	0.00–0.00
	May 17 to May 23, 2021	213	5–36	2.82–20.34	0–0	0.00–0.00

	May 24 to May 30, 2021	179	0–7	0.00–4.07	0–0	0.00–0.00
Niigata						
	Apr 5 to Apr 11, 2021	277	0–0	0.00–0.00	0–9	0.00–3.15
	Apr 12 to Apr 18, 2021	306	0–22	0.00–7.75	0–0	0.00–0.00
	Apr 19 to Apr 25, 2021	316	0–35	0.00–12.46	0–0	0.00–0.00
	Apr 26 to May 2, 2021	291	0–14	0.00–5.05	0–0	0.00–0.00
	May 3 to May 9, 2021	263	0–0	0.00–0.00	0–9	0.00–3.31
	May 10 to May 16, 2021	298	0–29	0.00–10.78	0–0	0.00–0.00
	May 17 to May 23, 2021	267	0–2	0.00–0.75	0–0	0.00–0.00
	May 24 to May 30, 2021	271	0–9	0.00–3.44	0–0	0.00–0.00
Toyama						
	Apr 5 to Apr 11, 2021	430	0–26	0.00–6.44	0–0	0.00–0.00
	Apr 12 to Apr 18, 2021	453	9–54	2.26–13.53	0–0	0.00–0.00
	Apr 19 to Apr 25, 2021	444	6–50	1.52–12.69	0–0	0.00–0.00
	Apr 26 to May 2, 2021	422	0–30	0.00–7.65	0–0	0.00–0.00
	May 3 to May 9, 2021	422	0–38	0.00–9.90	0–0	0.00–0.00
	May 10 to May 16, 2021	418	0–40	0.00–10.58	0–0	0.00–0.00
	May 17 to May 23, 2021	395	0–21	0.00–5.61	0–0	0.00–0.00
	May 24 to May 30, 2021	404	0–35	0.00–9.49	0–0	0.00–0.00
Ishikawa						
	Apr 5 to Apr 11, 2021	581	0–0	0.00–0.00	0–6	0.00–1.02
	Apr 12 to Apr 18, 2021	619	0–34	0.00–5.81	0–0	0.00–0.00
	Apr 19 to Apr 25, 2021	579	0–0	0.00–0.00	0–2	0.00–0.34
	Apr 26 to May 2, 2021	591	0–19	0.00–3.32	0–0	0.00–0.00
	May 3 to May 9, 2021	574	0–11	0.00–1.95	0–0	0.00–0.00
	May 10 to May 16, 2021	573	0–20	0.00–3.62	0–0	0.00–0.00
	May 17 to May 23, 2021	542	0–0	0.00–0.00	0–6	0.00–1.09
	May 24 to May 30, 2021	560	0–19	0.00–3.51	0–0	0.00–0.00
Fukui						
	Apr 5 to Apr 11, 2021	257	0–0	0.00–0.00	6–42	2.01–14.05
	Apr 12 to Apr 18, 2021	302	0–6	0.00–2.03	0–0	0.00–0.00
	Apr 19 to Apr 25, 2021	312	0–17	0.00–5.76	0–0	0.00–0.00
	Apr 26 to May 2, 2021	273	0–0	0.00–0.00	0–17	0.00–5.86
	May 3 to May 9, 2021	305	0–22	0.00–7.77	0–0	0.00–0.00
	May 10 to May 16, 2021	323	6–42	2.14–14.95	0–0	0.00–0.00
	May 17 to May 23, 2021	314	0–36	0.00–12.95	0–0	0.00–0.00
	May 24 to May 30, 2021	298	0–24	0.00–8.76	0–0	0.00–0.00
Yamanashi						
	Apr 5 to Apr 11, 2021	261	0–20	0.00–8.30	0–0	0.00–0.00
	Apr 12 to Apr 18, 2021	261	0–20	0.00–8.30	0–0	0.00–0.00
	Apr 19 to Apr 25, 2021	257	0–21	0.00–8.90	0–0	0.00–0.00
	Apr 26 to May 2, 2021	248	0–14	0.00–5.98	0–0	0.00–0.00
	May 3 to May 9, 2021	224	0–0	0.00–0.00	0–10	0.00–4.27
	May 10 to May 16, 2021	254	0–21	0.00–9.01	0–0	0.00–0.00
	May 17 to May 23, 2021	244	0–11	0.00–4.72	0–0	0.00–0.00
	May 24 to May 30, 2021	248	0–18	0.00–7.83	0–0	0.00–0.00

Nagano						
	Apr 5 to Apr 11, 2021	531	0–1	0.00–0.19	0–0	0.00–0.00
	Apr 12 to Apr 18, 2021	536	0–13	0.00–2.49	0–0	0.00–0.00
	Apr 19 to Apr 25, 2021	556	0–41	0.00–7.96	0–0	0.00–0.00
	Apr 26 to May 2, 2021	552	0–43	0.00–8.45	0–0	0.00–0.00
	May 3 to May 9, 2021	559	1–55	0.20–10.91	0–0	0.00–0.00
	May 10 to May 16, 2021	527	0–35	0.00–7.11	0–0	0.00–0.00
	May 17 to May 23, 2021	479	0–0	0.00–0.00	0–5	0.00–1.03
	May 24 to May 30, 2021	508	0–31	0.00–6.50	0–0	0.00–0.00
Gifu						
	Apr 5 to Apr 11, 2021	406	0–0	0.00–0.00	0–22	0.00–5.14
	Apr 12 to Apr 18, 2021	456	0–33	0.00–7.80	0–0	0.00–0.00
	Apr 19 to Apr 25, 2021	436	0–22	0.00–5.31	0–0	0.00–0.00
	Apr 26 to May 2, 2021	450	2–50	0.50–12.50	0–0	0.00–0.00
	May 3 to May 9, 2021	440	0–44	0.00–11.11	0–0	0.00–0.00
	May 10 to May 16, 2021	397	0–14	0.00–3.66	0–0	0.00–0.00
	May 17 to May 23, 2021	395	0–21	0.00–5.61	0–0	0.00–0.00
	May 24 to May 30, 2021	416	0–44	0.00–11.83	0–0	0.00–0.00
Shizuoka						
	Apr 5 to Apr 11, 2021	350	0–6	0.00–1.74	0–0	0.00–0.00
	Apr 12 to Apr 18, 2021	372	0–32	0.00–9.41	0–0	0.00–0.00
	Apr 19 to Apr 25, 2021	372	0–38	0.00–11.38	0–0	0.00–0.00
	Apr 26 to May 2, 2021	368	0–39	0.00–11.85	0–0	0.00–0.00
	May 3 to May 9, 2021	355	0–30	0.00–9.23	0–0	0.00–0.00
	May 10 to May 16, 2021	365	5–46	1.57–14.42	0–0	0.00–0.00
	May 17 to May 23, 2021	303	0–0	0.00–0.00	0–10	0.00–3.19
	May 24 to May 30, 2021	333	0–24	0.00–7.77	0–0	0.00–0.00
Aichi						
	Apr 5 to Apr 11, 2021	1257	0–8	0.00–0.64	0–0	0.00–0.00
	Apr 12 to Apr 18, 2021	1256	0–24	0.00–1.95	0–0	0.00–0.00
	Apr 19 to Apr 25, 2021	1274	0–46	0.00–3.75	0–0	0.00–0.00
	Apr 26 to May 2, 2021	1310	5–98	0.41–8.09	0–0	0.00–0.00
	May 3 to May 9, 2021	1300	3–95	0.25–7.88	0–0	0.00–0.00
	May 10 to May 16, 2021	1279	0–81	0.00–6.76	0–0	0.00–0.00
	May 17 to May 23, 2021	1322	32–126	2.68–10.54	0–0	0.00–0.00
	May 24 to May 30, 2021	1268	0–82	0.00–6.91	0–0	0.00–0.00
Mie						
	Apr 5 to Apr 11, 2021	1278	0–0	0.00–0.00	0–97	0.00–7.05
	Apr 12 to Apr 18, 2021	1368	0–11	0.00–0.81	0–0	0.00–0.00
	Apr 19 to Apr 25, 2021	1389	0–50	0.00–3.73	0–0	0.00–0.00
	Apr 26 to May 2, 2021	1405	0–83	0.00–6.28	0–0	0.00–0.00
	May 3 to May 9, 2021	1344	0–41	0.00–3.15	0–0	0.00–0.00
	May 10 to May 16, 2021	1422	40–152	3.15–11.97	0–0	0.00–0.00
	May 17 to May 23, 2021	1206	0–0	0.00–0.00	0–45	0.00–3.60
	May 24 to May 30, 2021	1274	0–39	0.00–3.16	0–0	0.00–0.00
Shiga						

	Apr 5 to Apr 11, 2021	206	0–15	0.00–7.85	0–0	0.00–0.00
	Apr 12 to Apr 18, 2021	188	0–0	0.00–0.00	0–1	0.00–0.53
	Apr 19 to Apr 25, 2021	242	28–58	15.22–31.52	0–0	0.00–0.00
	Apr 26 to May 2, 2021	190	0–11	0.00–6.15	0–0	0.00–0.00
	May 3 to May 9, 2021	209	3–32	1.69–18.08	0–0	0.00–0.00
	May 10 to May 16, 2021	215	8–37	4.49–20.79	0–0	0.00–0.00
	May 17 to May 23, 2021	173	0–1	0.00–0.58	0–0	0.00–0.00
	May 24 to May 30, 2021	176	0–6	0.00–3.53	0–0	0.00–0.00
Kyoto						
	Apr 5 to Apr 11, 2021	860	0–37	0.00–4.50	0–0	0.00–0.00
	Apr 12 to Apr 18, 2021	815	0–6	0.00–0.74	0–0	0.00–0.00
	Apr 19 to Apr 25, 2021	815	0–19	0.00–2.39	0–0	0.00–0.00
	Apr 26 to May 2, 2021	858	0–76	0.00–9.72	0–0	0.00–0.00
	May 3 to May 9, 2021	813	0–45	0.00–5.86	0–0	0.00–0.00
	May 10 to May 16, 2021	798	0–41	0.00–5.42	0–0	0.00–0.00
	May 17 to May 23, 2021	815	0–74	0.00–9.99	0–0	0.00–0.00
	May 24 to May 30, 2021	734	0–0	0.00–0.00	0–4	0.00–0.54
Osaka						
	Apr 5 to Apr 11, 2021	421	0–2	0.00–0.48	0–0	0.00–0.00
	Apr 12 to Apr 18, 2021	421	0–10	0.00–2.43	0–0	0.00–0.00
	Apr 19 to Apr 25, 2021	441	0–33	0.00–8.09	0–0	0.00–0.00
	Apr 26 to May 2, 2021	426	0–22	0.00–5.45	0–0	0.00–0.00
	May 3 to May 9, 2021	409	0–10	0.00–2.51	0–0	0.00–0.00
	May 10 to May 16, 2021	454	11–58	2.78–14.65	0–0	0.00–0.00
	May 17 to May 23, 2021	390	0–0	0.00–0.00	0–0	0.00–0.00
	May 24 to May 30, 2021	412	0–29	0.00–7.57	0–0	0.00–0.00
Hyogo						
	Apr 5 to Apr 11, 2021	2288	0–0	0.00–0.00	0–41	0.00–1.76
	Apr 12 to Apr 18, 2021	2463	1–166	0.04–7.23	0–0	0.00–0.00
	Apr 19 to Apr 25, 2021	2504	85–244	3.76–10.80	0–0	0.00–0.00
	Apr 26 to May 2, 2021	2459	88–246	3.98–11.12	0–0	0.00–0.00
	May 3 to May 9, 2021	2360	10–173	0.46–7.91	0–0	0.00–0.00
	May 10 to May 16, 2021	2227	0–58	0.00–2.67	0–0	0.00–0.00
	May 17 to May 23, 2021	2178	0–30	0.00–1.40	0–0	0.00–0.00
	May 24 to May 30, 2021	2162	0–37	0.00–1.74	0–0	0.00–0.00
Nara						
	Apr 5 to Apr 11, 2021	1145	0–11	0.00–0.97	0–0	0.00–0.00
	Apr 12 to Apr 18, 2021	1173	0–52	0.00–4.64	0–0	0.00–0.00
	Apr 19 to Apr 25, 2021	1185	0–86	0.00–7.83	0–0	0.00–0.00
	Apr 26 to May 2, 2021	1226	52–150	4.83–13.94	0–0	0.00–0.00
	May 3 to May 9, 2021	1186	26–123	2.45–11.57	0–0	0.00–0.00
	May 10 to May 16, 2021	1157	12–104	1.14–9.88	0–0	0.00–0.00
	May 17 to May 23, 2021	1144	5–100	0.48–9.58	0–0	0.00–0.00
	May 24 to May 30, 2021	1095	0–66	0.00–6.41	0–0	0.00–0.00
Wakayama						
	Apr 5 to Apr 11, 2021	257	0–0	0.00–0.00	0–18	0.00–6.55

	Apr 12 to Apr 18, 2021	309	1–37	0.37–13.60	0–0	0.00–0.00
	Apr 19 to Apr 25, 2021	326	24–58	8.96–21.64	0–0	0.00–0.00
	Apr 26 to May 2, 2021	279	0–12	0.00–4.49	0–0	0.00–0.00
	May 3 to May 9, 2021	272	0–11	0.00–4.21	0–0	0.00–0.00
	May 10 to May 16, 2021	309	15–49	5.77–18.85	0–0	0.00–0.00
	May 17 to May 23, 2021	245	0–0	0.00–0.00	0–13	0.00–5.04
	May 24 to May 30, 2021	252	0–0	0.00–0.00	0–0	0.00–0.00
Tottori						
	Apr 5 to Apr 11, 2021	350	0–7	0.00–2.04	0–0	0.00–0.00
	Apr 12 to Apr 18, 2021	331	0–0	0.00–0.00	0–9	0.00–2.65
	Apr 19 to Apr 25, 2021	369	0–34	0.00–10.15	0–0	0.00–0.00
	Apr 26 to May 2, 2021	328	0–2	0.00–0.61	0–0	0.00–0.00
	May 3 to May 9, 2021	373	12–50	3.72–15.48	0–0	0.00–0.00
	May 10 to May 16, 2021	361	4–42	1.25–13.17	0–0	0.00–0.00
	May 17 to May 23, 2021	299	0–0	0.00–0.00	0–14	0.00–4.47
	May 24 to May 30, 2021	378	33–73	10.82–23.93	0–0	0.00–0.00
Shimane						
	Apr 5 to Apr 11, 2021	233	0–0	0.00–0.00	0–8	0.00–3.32
	Apr 12 to Apr 18, 2021	256	0–18	0.00–7.56	0–0	0.00–0.00
	Apr 19 to Apr 25, 2021	227	0–0	0.00–0.00	0–7	0.00–2.99
	Apr 26 to May 2, 2021	254	0–25	0.00–10.92	0–0	0.00–0.00
	May 3 to May 9, 2021	232	0–7	0.00–3.11	0–0	0.00–0.00
	May 10 to May 16, 2021	223	0–5	0.00–2.29	0–0	0.00–0.00
	May 17 to May 23, 2021	221	0–5	0.00–2.31	0–0	0.00–0.00
	May 24 to May 30, 2021	223	0–15	0.00–7.21	0–0	0.00–0.00
Okayama						
	Apr 5 to Apr 11, 2021	144	0–4	0.00–2.86	0–0	0.00–0.00
	Apr 12 to Apr 18, 2021	158	0–20	0.00–14.49	0–0	0.00–0.00
	Apr 19 to Apr 25, 2021	147	0–12	0.00–8.89	0–0	0.00–0.00
	Apr 26 to May 2, 2021	132	0–0	0.00–0.00	0–0	0.00–0.00
	May 3 to May 9, 2021	156	1–24	0.76–18.18	0–0	0.00–0.00
	May 10 to May 16, 2021	162	9–32	6.92–24.62	0–0	0.00–0.00
	May 17 to May 23, 2021	137	0–9	0.00–7.03	0–0	0.00–0.00
	May 24 to May 30, 2021	137	0–12	0.00–9.60	0–0	0.00–0.00
Hiroshima						
	Apr 5 to Apr 11, 2021	1358	0–0	0.00–0.00	0–10	0.00–0.73
	Apr 12 to Apr 18, 2021	1408	0–60	0.00–4.45	0–0	0.00–0.00
	Apr 19 to Apr 25, 2021	1401	0–73	0.00–5.50	0–0	0.00–0.00
	Apr 26 to May 2, 2021	1508	84–198	6.41–15.11	0–0	0.00–0.00
	May 3 to May 9, 2021	1435	39–146	3.03–11.33	0–0	0.00–0.00
	May 10 to May 16, 2021	1371	0–104	0.00–8.21	0–0	0.00–0.00
	May 17 to May 23, 2021	1319	0–71	0.00–5.69	0–0	0.00–0.00
	May 24 to May 30, 2021	1293	0–57	0.00–4.61	0–0	0.00–0.00
Yamaguchi						
	Apr 5 to Apr 11, 2021	443	0–41	0.00–10.20	0–0	0.00–0.00
	Apr 12 to Apr 18, 2021	393	0–0	0.00–0.00	0–3	0.00–0.76

	Apr 19 to Apr 25, 2021	444	8–55	2.06–14.14	0–0	0.00–0.00
	Apr 26 to May 2, 2021	384	0–1	0.00–0.26	0–0	0.00–0.00
	May 3 to May 9, 2021	401	0–20	0.00–5.25	0–0	0.00–0.00
	May 10 to May 16, 2021	377	0–0	0.00–0.00	0–0	0.00–0.00
	May 17 to May 23, 2021	393	0–19	0.00–5.08	0–0	0.00–0.00
	May 24 to May 30, 2021	400	0–32	0.00–8.70	0–0	0.00–0.00
Tokushima						
	Apr 5 to Apr 11, 2021	1001	0–0	0.00–0.00	0–44	0.00–4.21
	Apr 12 to Apr 18, 2021	1058	0–25	0.00–2.42	0–0	0.00–0.00
	Apr 19 to Apr 25, 2021	1161	54–142	5.30–13.94	0–0	0.00–0.00
	Apr 26 to May 2, 2021	1087	0–82	0.00–8.16	0–0	0.00–0.00
	May 3 to May 9, 2021	1068	3–82	0.30–8.32	0–0	0.00–0.00
	May 10 to May 16, 2021	1082	19–105	1.94–10.75	0–0	0.00–0.00
	May 17 to May 23, 2021	991	0–30	0.00–3.12	0–0	0.00–0.00
	May 24 to May 30, 2021	1010	0–58	0.00–6.09	0–0	0.00–0.00
Kagawa						
	Apr 5 to Apr 11, 2021	268	0–22	0.00–8.94	0–0	0.00–0.00
	Apr 12 to Apr 18, 2021	285	9–40	3.67–16.33	0–0	0.00–0.00
	Apr 19 to Apr 25, 2021	257	0–14	0.00–5.76	0–0	0.00–0.00
	Apr 26 to May 2, 2021	254	0–16	0.00–6.72	0–0	0.00–0.00
	May 3 to May 9, 2021	280	13–45	5.53–19.15	0–0	0.00–0.00
	May 10 to May 16, 2021	261	0–28	0.00–12.02	0–0	0.00–0.00
	May 17 to May 23, 2021	242	0–13	0.00–5.68	0–0	0.00–0.00
	May 24 to May 30, 2021	220	0–0	0.00–0.00	0–7	0.00–3.08
Ehime						
	Apr 5 to Apr 11, 2021	309	0–6	0.00–1.98	0–0	0.00–0.00
	Apr 12 to Apr 18, 2021	325	0–21	0.00–6.91	0–0	0.00–0.00
	Apr 19 to Apr 25, 2021	288	0–0	0.00–0.00	0–14	0.00–4.64
	Apr 26 to May 2, 2021	335	5–41	1.70–13.95	0–0	0.00–0.00
	May 3 to May 9, 2021	338	14–49	4.84–16.96	0–0	0.00–0.00
	May 10 to May 16, 2021	286	0–2	0.00–0.70	0–0	0.00–0.00
	May 17 to May 23, 2021	306	0–26	0.00–9.29	0–0	0.00–0.00
	May 24 to May 30, 2021	272	0–0	0.00–0.00	0–4	0.00–1.45
Kochi						
	Apr 5 to Apr 11, 2021	495	0–0	0.00–0.00	0–5	0.00–1.00
	Apr 12 to Apr 18, 2021	508	0–15	0.00–3.04	0–0	0.00–0.00
	Apr 19 to Apr 25, 2021	490	0–3	0.00–0.62	0–0	0.00–0.00
	Apr 26 to May 2, 2021	525	0–44	0.00–9.15	0–0	0.00–0.00
	May 3 to May 9, 2021	459	0–0	0.00–0.00	0–10	0.00–2.13
	May 10 to May 16, 2021	497	0–32	0.00–6.88	0–0	0.00–0.00
	May 17 to May 23, 2021	475	0–16	0.00–3.49	0–0	0.00–0.00
	May 24 to May 30, 2021	415	0–0	0.00–0.00	0–35	0.00–7.78
Fukuoka						
	Apr 5 to Apr 11, 2021	463	0–0	0.00–0.00	0–9	0.00–1.91
	Apr 12 to Apr 18, 2021	492	0–24	0.00–5.13	0–0	0.00–0.00
	Apr 19 to Apr 25, 2021	468	0–8	0.00–1.74	0–0	0.00–0.00

	Apr 26 to May 2, 2021	491	0–40	0.00–8.87	0–0	0.00–0.00
	May 3 to May 9, 2021	478	0–33	0.00–7.42	0–0	0.00–0.00
	May 10 to May 16, 2021	472	0–32	0.00–7.27	0–0	0.00–0.00
	May 17 to May 23, 2021	451	0–17	0.00–3.92	0–0	0.00–0.00
	May 24 to May 30, 2021	431	0–0	0.00–0.00	0–0	0.00–0.00
Saga						
	Apr 5 to Apr 11, 2021	617	0–26	0.00–4.40	0–0	0.00–0.00
	Apr 12 to Apr 18, 2021	599	0–15	0.00–2.57	0–0	0.00–0.00
	Apr 19 to Apr 25, 2021	635	0–63	0.00–11.01	0–0	0.00–0.00
	Apr 26 to May 2, 2021	614	0–49	0.00–8.67	0–0	0.00–0.00
	May 3 to May 9, 2021	636	20–82	3.61–14.80	0–0	0.00–0.00
	May 10 to May 16, 2021	611	1–62	0.18–11.29	0–0	0.00–0.00
	May 17 to May 23, 2021	584	0–46	0.00–8.55	0–0	0.00–0.00
	May 24 to May 30, 2021	541	0–12	0.00–2.27	0–0	0.00–0.00
Nagasaki						
	Apr 5 to Apr 11, 2021	1830	0–35	0.00–1.95	0–0	0.00–0.00
	Apr 12 to Apr 18, 2021	1792	0–13	0.00–0.73	0–0	0.00–0.00
	Apr 19 to Apr 25, 2021	1946	55–194	3.14–11.07	0–0	0.00–0.00
	Apr 26 to May 2, 2021	1853	0–131	0.00–7.61	0–0	0.00–0.00
	May 3 to May 9, 2021	1910	80–208	4.70–12.22	0–0	0.00–0.00
	May 10 to May 16, 2021	1838	23–155	1.37–9.21	0–0	0.00–0.00
	May 17 to May 23, 2021	1735	0–63	0.00–3.77	0–0	0.00–0.00
	May 24 to May 30, 2021	1707	0–58	0.00–3.52	0–0	0.00–0.00
Kumamoto						
	Apr 5 to Apr 11, 2021	438	0–0	0.00–0.00	0–12	0.00–2.67
	Apr 12 to Apr 18, 2021	449	0–14	0.00–3.22	0–0	0.00–0.00
	Apr 19 to Apr 25, 2021	466	0–37	0.00–8.62	0–0	0.00–0.00
	Apr 26 to May 2, 2021	435	0–17	0.00–4.07	0–0	0.00–0.00
	May 3 to May 9, 2021	451	0–41	0.00–10.00	0–0	0.00–0.00
	May 10 to May 16, 2021	457	2–51	0.49–12.56	0–0	0.00–0.00
	May 17 to May 23, 2021	465	15–65	3.75–16.25	0–0	0.00–0.00
	May 24 to May 30, 2021	384	0–0	0.00–0.00	0–13	0.00–3.27
Oita						
	Apr 5 to Apr 11, 2021	241	0–0	0.00–0.00	0–4	0.00–1.63
	Apr 12 to Apr 18, 2021	254	0–11	0.00–4.53	0–0	0.00–0.00
	Apr 19 to Apr 25, 2021	263	0–25	0.00–10.50	0–0	0.00–0.00
	Apr 26 to May 2, 2021	239	0–4	0.00–1.70	0–0	0.00–0.00
	May 3 to May 9, 2021	250	0–22	0.00–9.65	0–0	0.00–0.00
	May 10 to May 16, 2021	230	0–9	0.00–4.07	0–0	0.00–0.00
	May 17 to May 23, 2021	210	0–0	0.00–0.00	0–8	0.00–3.67
	May 24 to May 30, 2021	219	0–3	0.00–1.39	0–0	0.00–0.00
Miyazaki						
	Apr 5 to Apr 11, 2021	289	0–34	0.00–13.33	0–0	0.00–0.00
	Apr 12 to Apr 18, 2021	285	0–32	0.00–12.65	0–0	0.00–0.00
	Apr 19 to Apr 25, 2021	250	0–2	0.00–0.81	0–0	0.00–0.00
	Apr 26 to May 2, 2021	286	7–42	2.87–17.21	0–0	0.00–0.00

	May 3 to May 9, 2021	273	0–33	0.00–13.75	0–0	0.00–0.00
	May 10 to May 16, 2021	256	0–23	0.00–9.87	0–0	0.00–0.00
	May 17 to May 23, 2021	252	0–22	0.00–9.57	0–0	0.00–0.00
	May 24 to May 30, 2021	261	0–34	0.00–14.98	0–0	0.00–0.00
Kagoshima						
	Apr 5 to Apr 11, 2021	193	0–14	0.00–7.82	0–0	0.00–0.00
	Apr 12 to Apr 18, 2021	194	0–15	0.00–8.38	0–0	0.00–0.00
	Apr 19 to Apr 25, 2021	189	0–12	0.00–6.78	0–0	0.00–0.00
	Apr 26 to May 2, 2021	204	3–33	1.75–19.30	0–0	0.00–0.00
	May 3 to May 9, 2021	217	21–50	12.57–29.94	0–0	0.00–0.00
	May 10 to May 16, 2021	181	0–16	0.00–9.70	0–0	0.00–0.00
	May 17 to May 23, 2021	193	0–29	0.00–17.68	0–0	0.00–0.00
	May 24 to May 30, 2021	172	0–11	0.00–6.83	0–0	0.00–0.00
Okinawa						
	Apr 5 to Apr 11, 2021	160	0–0	0.00–0.00	3–30	1.58–15.79
	Apr 12 to Apr 18, 2021	220	5–34	2.69–18.28	0–0	0.00–0.00
	Apr 19 to Apr 25, 2021	189	0–4	0.00–2.16	0–0	0.00–0.00
	Apr 26 to May 2, 2021	194	0–14	0.00–7.78	0–0	0.00–0.00
	May 3 to May 9, 2021	202	0–27	0.00–15.43	0–0	0.00–0.00
	May 10 to May 16, 2021	183	0–12	0.00–7.02	0–0	0.00–0.00
	May 17 to May 23, 2021	186	0–19	0.00–11.38	0–0	0.00–0.00
	May 24 to May 30, 2021	178	0–13	0.00–7.88	0–0	0.00–0.00

Table A.19: Weekly number of observed and excess/exiguous deaths in Japan and 47 prefectures for senility-related deaths in nursing homes and elderly care facilities from January 2020 through May 2021.

Prefecture	Week	Observed	Excess deaths	Percent excess	Exiguous deaths	Percent exiguous
Japan						
	Apr 5 to Apr 11, 2021	1296	0–6	0.00–0.47	0–0	0.00–0.00
	Apr 12 to Apr 18, 2021	1438	68–160	5.32–12.52	0–0	0.00–0.00
	Apr 19 to Apr 25, 2021	1327	0–67	0.00–5.32	0–0	0.00–0.00
	Apr 26 to May 2, 2021	1489	142–240	11.37–19.22	0–0	0.00–0.00
	May 3 to May 9, 2021	1444	112–202	9.02–16.26	0–0	0.00–0.00
	May 10 to May 16, 2021	1400	84–169	6.82–13.73	0–0	0.00–0.00
	May 17 to May 23, 2021	1317	2–91	0.16–7.42	0–0	0.00–0.00
	May 24 to May 30, 2021	1317	0–95	0.00–7.77	0–0	0.00–0.00
Hokkaido						
	Apr 5 to Apr 11, 2021	35	0–5	0.00–16.67	0–0	0.00–0.00
	Apr 12 to Apr 18, 2021	44	2–14	6.67–46.67	0–0	0.00–0.00
	Apr 19 to Apr 25, 2021	33	0–3	0.00–10.00	0–0	0.00–0.00
	Apr 26 to May 2, 2021	44	2–14	6.67–46.67	0–0	0.00–0.00
	May 3 to May 9, 2021	41	0–11	0.00–36.67	0–0	0.00–0.00
	May 10 to May 16, 2021	28	0–0	0.00–0.00	0–2	0.00–6.67
	May 17 to May 23, 2021	31	0–1	0.00–3.33	0–0	0.00–0.00
	May 24 to May 30, 2021	43	0–13	0.00–43.33	0–0	0.00–0.00
Aomori						
	Apr 5 to Apr 11, 2021	15	0–0	0.00–0.00	0–3	0.00–16.67
	Apr 12 to Apr 18, 2021	17	0–0	0.00–0.00	0–1	0.00–5.56
	Apr 19 to Apr 25, 2021	17	0–0	0.00–0.00	0–1	0.00–5.56
	Apr 26 to May 2, 2021	20	0–3	0.00–17.65	0–0	0.00–0.00
	May 3 to May 9, 2021	19	0–2	0.00–11.76	0–0	0.00–0.00
	May 10 to May 16, 2021	19	0–2	0.00–11.76	0–0	0.00–0.00
	May 17 to May 23, 2021	15	0–0	0.00–0.00	0–1	0.00–6.25
	May 24 to May 30, 2021	19	0–3	0.00–18.75	0–0	0.00–0.00
Iwate						
	Apr 5 to Apr 11, 2021	17	0–1	0.00–6.25	0–0	0.00–0.00
	Apr 12 to Apr 18, 2021	15	0–0	0.00–0.00	0–1	0.00–6.25
	Apr 19 to Apr 25, 2021	11	0–0	0.00–0.00	0–5	0.00–31.25
	Apr 26 to May 2, 2021	12	0–0	0.00–0.00	0–4	0.00–25.00
	May 3 to May 9, 2021	18	0–2	0.00–12.50	0–0	0.00–0.00
	May 10 to May 16, 2021	14	0–0	0.00–0.00	0–2	0.00–12.50
	May 17 to May 23, 2021	12	0–0	0.00–0.00	0–4	0.00–25.00
	May 24 to May 30, 2021	12	0–0	0.00–0.00	0–4	0.00–25.00
Miyagi						
	Apr 5 to Apr 11, 2021	29	0–7	0.00–31.82	0–0	0.00–0.00
	Apr 12 to Apr 18, 2021	34	1–11	4.35–47.83	0–0	0.00–0.00
	Apr 19 to Apr 25, 2021	26	0–3	0.00–13.04	0–0	0.00–0.00
	Apr 26 to May 2, 2021	28	0–4	0.00–16.67	0–0	0.00–0.00
	May 3 to May 9, 2021	24	0–0	0.00–0.00	0–0	0.00–0.00

	May 10 to May 16, 2021	31	0–7	0.00–29.17	0–0	0.00–0.00
	May 17 to May 23, 2021	20	0–0	0.00–0.00	0–3	0.00–13.04
	May 24 to May 30, 2021	20	0–0	0.00–0.00	0–4	0.00–16.67
Akita						
	Apr 5 to Apr 11, 2021	10	0–0	0.00–0.00	0–2	0.00–16.67
	Apr 12 to Apr 18, 2021	17	0–5	0.00–41.67	0–0	0.00–0.00
	Apr 19 to Apr 25, 2021	9	0–0	0.00–0.00	0–3	0.00–25.00
	Apr 26 to May 2, 2021	12	0–0	0.00–0.00	0–0	0.00–0.00
	May 3 to May 9, 2021	20	2–9	18.18–81.82	0–0	0.00–0.00
	May 10 to May 16, 2021	13	0–3	0.00–30.00	0–0	0.00–0.00
	May 17 to May 23, 2021	6	0–0	0.00–0.00	0–5	0.00–45.45
	May 24 to May 30, 2021	12	0–1	0.00–9.09	0–0	0.00–0.00
Yamagata						
	Apr 5 to Apr 11, 2021	10	0–0	0.00–0.00	4–12	18.18–54.55
	Apr 12 to Apr 18, 2021	24	0–3	0.00–14.29	0–0	0.00–0.00
	Apr 19 to Apr 25, 2021	18	0–0	0.00–0.00	0–2	0.00–10.00
	Apr 26 to May 2, 2021	27	0–7	0.00–35.00	0–0	0.00–0.00
	May 3 to May 9, 2021	28	0–8	0.00–40.00	0–0	0.00–0.00
	May 10 to May 16, 2021	23	0–3	0.00–15.00	0–0	0.00–0.00
	May 17 to May 23, 2021	27	0–7	0.00–35.00	0–0	0.00–0.00
	May 24 to May 30, 2021	33	4–14	21.05–73.68	0–0	0.00–0.00
Fukushima						
	Apr 5 to Apr 11, 2021	26	0–5	0.00–23.81	0–0	0.00–0.00
	Apr 12 to Apr 18, 2021	24	0–3	0.00–14.29	0–0	0.00–0.00
	Apr 19 to Apr 25, 2021	23	0–2	0.00–9.52	0–0	0.00–0.00
	Apr 26 to May 2, 2021	24	0–3	0.00–14.29	0–0	0.00–0.00
	May 3 to May 9, 2021	20	0–0	0.00–0.00	0–1	0.00–4.76
	May 10 to May 16, 2021	30	0–10	0.00–50.00	0–0	0.00–0.00
	May 17 to May 23, 2021	22	0–1	0.00–4.76	0–0	0.00–0.00
	May 24 to May 30, 2021	19	0–0	0.00–0.00	0–1	0.00–5.00
Ibaraki						
	Apr 5 to Apr 11, 2021	21	0–0	0.00–0.00	0–7	0.00–25.00
	Apr 12 to Apr 18, 2021	35	0–7	0.00–25.00	0–0	0.00–0.00
	Apr 19 to Apr 25, 2021	16	0–0	0.00–0.00	1–11	3.70–40.74
	Apr 26 to May 2, 2021	23	0–0	0.00–0.00	0–3	0.00–11.54
	May 3 to May 9, 2021	30	0–4	0.00–15.38	0–0	0.00–0.00
	May 10 to May 16, 2021	32	0–7	0.00–28.00	0–0	0.00–0.00
	May 17 to May 23, 2021	23	0–0	0.00–0.00	0–2	0.00–8.00
	May 24 to May 30, 2021	40	4–15	16.00–60.00	0–0	0.00–0.00
Tochigi						
	Apr 5 to Apr 11, 2021	28	0–9	0.00–47.37	0–0	0.00–0.00
	Apr 12 to Apr 18, 2021	30	1–10	5.00–50.00	0–0	0.00–0.00
	Apr 19 to Apr 25, 2021	26	0–7	0.00–36.84	0–0	0.00–0.00
	Apr 26 to May 2, 2021	24	0–5	0.00–26.32	0–0	0.00–0.00
	May 3 to May 9, 2021	17	0–0	0.00–0.00	0–1	0.00–5.56
	May 10 to May 16, 2021	12	0–0	0.00–0.00	0–5	0.00–29.41

	May 17 to May 23, 2021	13	0–0	0.00–0.00	0–4	0.00–23.53
	May 24 to May 30, 2021	17	0–0	0.00–0.00	0–0	0.00–0.00
Gunma						
	Apr 5 to Apr 11, 2021	20	0–0	0.00–0.00	0–2	0.00–9.09
	Apr 12 to Apr 18, 2021	25	0–3	0.00–13.64	0–0	0.00–0.00
	Apr 19 to Apr 25, 2021	40	10–19	47.62–90.48	0–0	0.00–0.00
	Apr 26 to May 2, 2021	28	0–7	0.00–33.33	0–0	0.00–0.00
	May 3 to May 9, 2021	24	0–3	0.00–14.29	0–0	0.00–0.00
	May 10 to May 16, 2021	12	0–0	0.00–0.00	0–8	0.00–40.00
	May 17 to May 23, 2021	17	0–0	0.00–0.00	0–3	0.00–15.00
	May 24 to May 30, 2021	25	0–5	0.00–25.00	0–0	0.00–0.00
Saitama						
	Apr 5 to Apr 11, 2021	62	0–2	0.00–3.33	0–0	0.00–0.00
	Apr 12 to Apr 18, 2021	71	0–13	0.00–22.41	0–0	0.00–0.00
	Apr 19 to Apr 25, 2021	63	0–6	0.00–10.53	0–0	0.00–0.00
	Apr 26 to May 2, 2021	70	0–14	0.00–25.00	0–0	0.00–0.00
	May 3 to May 9, 2021	62	0–5	0.00–8.77	0–0	0.00–0.00
	May 10 to May 16, 2021	65	0–9	0.00–16.07	0–0	0.00–0.00
	May 17 to May 23, 2021	50	0–0	0.00–0.00	0–6	0.00–10.71
	May 24 to May 30, 2021	57	0–1	0.00–1.79	0–0	0.00–0.00
Chiba						
	Apr 5 to Apr 11, 2021	53	0–4	0.00–8.16	0–0	0.00–0.00
	Apr 12 to Apr 18, 2021	43	0–0	0.00–0.00	0–4	0.00–8.51
	Apr 19 to Apr 25, 2021	52	0–4	0.00–8.33	0–0	0.00–0.00
	Apr 26 to May 2, 2021	42	0–0	0.00–0.00	0–6	0.00–12.50
	May 3 to May 9, 2021	55	0–5	0.00–10.00	0–0	0.00–0.00
	May 10 to May 16, 2021	50	0–0	0.00–0.00	0–0	0.00–0.00
	May 17 to May 23, 2021	49	0–0	0.00–0.00	0–1	0.00–2.00
	May 24 to May 30, 2021	50	0–0	0.00–0.00	0–1	0.00–1.96
Tokyo						
	Apr 5 to Apr 11, 2021	129	0–8	0.00–6.61	0–0	0.00–0.00
	Apr 12 to Apr 18, 2021	129	0–7	0.00–5.74	0–0	0.00–0.00
	Apr 19 to Apr 25, 2021	121	0–0	0.00–0.00	0–0	0.00–0.00
	Apr 26 to May 2, 2021	136	0–15	0.00–12.40	0–0	0.00–0.00
	May 3 to May 9, 2021	135	0–16	0.00–13.45	0–0	0.00–0.00
	May 10 to May 16, 2021	145	7–29	6.03–25.00	0–0	0.00–0.00
	May 17 to May 23, 2021	129	0–14	0.00–12.17	0–0	0.00–0.00
	May 24 to May 30, 2021	109	0–0	0.00–0.00	0–4	0.00–3.54
Kanagawa						
	Apr 5 to Apr 11, 2021	98	0–0	0.00–0.00	0–2	0.00–2.00
	Apr 12 to Apr 18, 2021	108	0–8	0.00–8.00	0–0	0.00–0.00
	Apr 19 to Apr 25, 2021	83	0–0	0.00–0.00	0–17	0.00–17.00
	Apr 26 to May 2, 2021	125	5–25	5.00–25.00	0–0	0.00–0.00
	May 3 to May 9, 2021	123	3–23	3.00–23.00	0–0	0.00–0.00
	May 10 to May 16, 2021	125	3–24	2.97–23.76	0–0	0.00–0.00
	May 17 to May 23, 2021	102	0–1	0.00–0.99	0–0	0.00–0.00

	May 24 to May 30, 2021	104	0–3	0.00–2.97	0–0	0.00–0.00
Niigata						
	Apr 5 to Apr 11, 2021	29	0–0	0.00–0.00	0–9	0.00–23.68
	Apr 12 to Apr 18, 2021	40	0–3	0.00–8.11	0–0	0.00–0.00
	Apr 19 to Apr 25, 2021	35	0–0	0.00–0.00	0–1	0.00–2.78
	Apr 26 to May 2, 2021	33	0–0	0.00–0.00	0–3	0.00–8.33
	May 3 to May 9, 2021	40	0–4	0.00–11.11	0–0	0.00–0.00
	May 10 to May 16, 2021	39	0–2	0.00–5.41	0–0	0.00–0.00
	May 17 to May 23, 2021	40	0–5	0.00–14.29	0–0	0.00–0.00
	May 24 to May 30, 2021	36	0–1	0.00–2.86	0–0	0.00–0.00
Toyama						
	Apr 5 to Apr 11, 2021	16	0–2	0.00–14.29	0–0	0.00–0.00
	Apr 12 to Apr 18, 2021	12	0–0	0.00–0.00	0–2	0.00–14.29
	Apr 19 to Apr 25, 2021	16	0–2	0.00–14.29	0–0	0.00–0.00
	Apr 26 to May 2, 2021	18	0–5	0.00–38.46	0–0	0.00–0.00
	May 3 to May 9, 2021	13	0–0	0.00–0.00	0–1	0.00–7.14
	May 10 to May 16, 2021	13	0–0	0.00–0.00	0–0	0.00–0.00
	May 17 to May 23, 2021	14	0–1	0.00–7.69	0–0	0.00–0.00
	May 24 to May 30, 2021	9	0–0	0.00–0.00	0–3	0.00–25.00
Ishikawa						
	Apr 5 to Apr 11, 2021	11	0–0	0.00–0.00	0–3	0.00–21.43
	Apr 12 to Apr 18, 2021	11	0–0	0.00–0.00	0–2	0.00–15.38
	Apr 19 to Apr 25, 2021	14	0–2	0.00–16.67	0–0	0.00–0.00
	Apr 26 to May 2, 2021	9	0–0	0.00–0.00	0–4	0.00–30.77
	May 3 to May 9, 2021	11	0–0	0.00–0.00	0–2	0.00–15.38
	May 10 to May 16, 2021	9	0–0	0.00–0.00	0–4	0.00–30.77
	May 17 to May 23, 2021	8	0–0	0.00–0.00	0–4	0.00–33.33
	May 24 to May 30, 2021	14	0–2	0.00–16.67	0–0	0.00–0.00
Fukui						
	Apr 5 to Apr 11, 2021	7	0–0	0.00–0.00	0–2	0.00–22.22
	Apr 12 to Apr 18, 2021	12	0–3	0.00–33.33	0–0	0.00–0.00
	Apr 19 to Apr 25, 2021	11	0–2	0.00–22.22	0–0	0.00–0.00
	Apr 26 to May 2, 2021	8	0–0	0.00–0.00	0–1	0.00–11.11
	May 3 to May 9, 2021	10	0–1	0.00–11.11	0–0	0.00–0.00
	May 10 to May 16, 2021	11	0–3	0.00–37.50	0–0	0.00–0.00
	May 17 to May 23, 2021	12	0–4	0.00–50.00	0–0	0.00–0.00
	May 24 to May 30, 2021	11	0–3	0.00–37.50	0–0	0.00–0.00
Yamanashi						
	Apr 5 to Apr 11, 2021	12	0–1	0.00–9.09	0–0	0.00–0.00
	Apr 12 to Apr 18, 2021	10	0–0	0.00–0.00	0–0	0.00–0.00
	Apr 19 to Apr 25, 2021	9	0–0	0.00–0.00	0–1	0.00–10.00
	Apr 26 to May 2, 2021	13	0–3	0.00–30.00	0–0	0.00–0.00
	May 3 to May 9, 2021	10	0–0	0.00–0.00	0–0	0.00–0.00
	May 10 to May 16, 2021	10	0–1	0.00–11.11	0–0	0.00–0.00
	May 17 to May 23, 2021	10	0–1	0.00–11.11	0–0	0.00–0.00
	May 24 to May 30, 2021	10	0–1	0.00–11.11	0–0	0.00–0.00

Nagano						
	Apr 5 to Apr 11, 2021	30	0–0	0.00–0.00	0–3	0.00–9.09
	Apr 12 to Apr 18, 2021	29	0–0	0.00–0.00	0–4	0.00–12.12
	Apr 19 to Apr 25, 2021	36	0–3	0.00–9.09	0–0	0.00–0.00
	Apr 26 to May 2, 2021	44	0–10	0.00–29.41	0–0	0.00–0.00
	May 3 to May 9, 2021	31	0–0	0.00–0.00	0–2	0.00–6.06
	May 10 to May 16, 2021	37	0–4	0.00–12.12	0–0	0.00–0.00
	May 17 to May 23, 2021	40	0–7	0.00–21.21	0–0	0.00–0.00
	May 24 to May 30, 2021	26	0–0	0.00–0.00	0–6	0.00–18.75
Gifu						
	Apr 5 to Apr 11, 2021	30	0–3	0.00–11.11	0–0	0.00–0.00
	Apr 12 to Apr 18, 2021	31	0–6	0.00–24.00	0–0	0.00–0.00
	Apr 19 to Apr 25, 2021	25	0–0	0.00–0.00	0–1	0.00–3.85
	Apr 26 to May 2, 2021	29	0–3	0.00–11.54	0–0	0.00–0.00
	May 3 to May 9, 2021	30	0–5	0.00–20.00	0–0	0.00–0.00
	May 10 to May 16, 2021	26	0–2	0.00–8.33	0–0	0.00–0.00
	May 17 to May 23, 2021	38	4–14	16.67–58.33	0–0	0.00–0.00
	May 24 to May 30, 2021	17	0–0	0.00–0.00	0–8	0.00–32.00
Shizuoka						
	Apr 5 to Apr 11, 2021	63	0–0	0.00–0.00	0–7	0.00–10.00
	Apr 12 to Apr 18, 2021	65	0–0	0.00–0.00	0–5	0.00–7.14
	Apr 19 to Apr 25, 2021	65	0–0	0.00–0.00	0–4	0.00–5.80
	Apr 26 to May 2, 2021	75	0–7	0.00–10.29	0–0	0.00–0.00
	May 3 to May 9, 2021	66	0–0	0.00–0.00	0–0	0.00–0.00
	May 10 to May 16, 2021	64	0–0	0.00–0.00	0–1	0.00–1.54
	May 17 to May 23, 2021	68	0–3	0.00–4.62	0–0	0.00–0.00
	May 24 to May 30, 2021	65	0–0	0.00–0.00	0–1	0.00–1.52
Aichi						
	Apr 5 to Apr 11, 2021	77	0–0	0.00–0.00	0–9	0.00–10.47
	Apr 12 to Apr 18, 2021	99	0–14	0.00–16.47	0–0	0.00–0.00
	Apr 19 to Apr 25, 2021	82	0–0	0.00–0.00	0–2	0.00–2.38
	Apr 26 to May 2, 2021	99	0–16	0.00–19.28	0–0	0.00–0.00
	May 3 to May 9, 2021	81	0–1	0.00–1.25	0–0	0.00–0.00
	May 10 to May 16, 2021	98	0–18	0.00–22.50	0–0	0.00–0.00
	May 17 to May 23, 2021	69	0–0	0.00–0.00	0–12	0.00–14.81
	May 24 to May 30, 2021	91	0–11	0.00–13.75	0–0	0.00–0.00
Mie						
	Apr 5 to Apr 11, 2021	25	0–0	0.00–0.00	0–3	0.00–10.71
	Apr 12 to Apr 18, 2021	26	0–0	0.00–0.00	0–1	0.00–3.70
	Apr 19 to Apr 25, 2021	32	0–5	0.00–18.52	0–0	0.00–0.00
	Apr 26 to May 2, 2021	27	0–0	0.00–0.00	0–0	0.00–0.00
	May 3 to May 9, 2021	32	0–6	0.00–23.08	0–0	0.00–0.00
	May 10 to May 16, 2021	25	0–0	0.00–0.00	0–1	0.00–3.85
	May 17 to May 23, 2021	39	2–13	7.69–50.00	0–0	0.00–0.00
	May 24 to May 30, 2021	28	0–2	0.00–7.69	0–0	0.00–0.00
Shiga						

	Apr 5 to Apr 11, 2021	11	0–0	0.00–0.00	0–0	0.00–0.00
	Apr 12 to Apr 18, 2021	11	0–0	0.00–0.00	0–0	0.00–0.00
	Apr 19 to Apr 25, 2021	10	0–0	0.00–0.00	0–2	0.00–16.67
	Apr 26 to May 2, 2021	16	0–4	0.00–33.33	0–0	0.00–0.00
	May 3 to May 9, 2021	19	0–8	0.00–72.73	0–0	0.00–0.00
	May 10 to May 16, 2021	15	0–3	0.00–25.00	0–0	0.00–0.00
	May 17 to May 23, 2021	11	0–0	0.00–0.00	0–1	0.00–8.33
	May 24 to May 30, 2021	18	0–6	0.00–50.00	0–0	0.00–0.00
Kyoto						
	Apr 5 to Apr 11, 2021	24	0–0	0.00–0.00	0–1	0.00–4.00
	Apr 12 to Apr 18, 2021	28	0–4	0.00–16.67	0–0	0.00–0.00
	Apr 19 to Apr 25, 2021	26	0–2	0.00–8.33	0–0	0.00–0.00
	Apr 26 to May 2, 2021	26	0–1	0.00–4.00	0–0	0.00–0.00
	May 3 to May 9, 2021	37	3–14	13.04–60.87	0–0	0.00–0.00
	May 10 to May 16, 2021	19	0–0	0.00–0.00	0–5	0.00–20.83
	May 17 to May 23, 2021	26	0–2	0.00–8.33	0–0	0.00–0.00
	May 24 to May 30, 2021	29	0–6	0.00–26.09	0–0	0.00–0.00
Osaka						
	Apr 5 to Apr 11, 2021	66	0–3	0.00–4.76	0–0	0.00–0.00
	Apr 12 to Apr 18, 2021	67	0–6	0.00–9.84	0–0	0.00–0.00
	Apr 19 to Apr 25, 2021	69	0–8	0.00–13.11	0–0	0.00–0.00
	Apr 26 to May 2, 2021	75	0–14	0.00–22.95	0–0	0.00–0.00
	May 3 to May 9, 2021	58	0–0	0.00–0.00	0–1	0.00–1.69
	May 10 to May 16, 2021	78	1–18	1.67–30.00	0–0	0.00–0.00
	May 17 to May 23, 2021	67	0–8	0.00–13.56	0–0	0.00–0.00
	May 24 to May 30, 2021	54	0–0	0.00–0.00	0–4	0.00–6.90
Hyogo						
	Apr 5 to Apr 11, 2021	49	0–0	0.00–0.00	0–8	0.00–14.04
	Apr 12 to Apr 18, 2021	50	0–0	0.00–0.00	0–8	0.00–13.79
	Apr 19 to Apr 25, 2021	67	0–9	0.00–15.52	0–0	0.00–0.00
	Apr 26 to May 2, 2021	72	0–14	0.00–24.14	0–0	0.00–0.00
	May 3 to May 9, 2021	72	0–15	0.00–26.32	0–0	0.00–0.00
	May 10 to May 16, 2021	55	0–0	0.00–0.00	0–2	0.00–3.51
	May 17 to May 23, 2021	68	0–12	0.00–21.43	0–0	0.00–0.00
	May 24 to May 30, 2021	41	0–0	0.00–0.00	0–14	0.00–25.45
Nara						
	Apr 5 to Apr 11, 2021	15	0–0	0.00–0.00	0–0	0.00–0.00
	Apr 12 to Apr 18, 2021	18	0–3	0.00–20.00	0–0	0.00–0.00
	Apr 19 to Apr 25, 2021	16	0–1	0.00–6.67	0–0	0.00–0.00
	Apr 26 to May 2, 2021	19	0–4	0.00–26.67	0–0	0.00–0.00
	May 3 to May 9, 2021	11	0–0	0.00–0.00	0–4	0.00–26.67
	May 10 to May 16, 2021	18	0–3	0.00–20.00	0–0	0.00–0.00
	May 17 to May 23, 2021	23	0–8	0.00–53.33	0–0	0.00–0.00
	May 24 to May 30, 2021	19	0–4	0.00–26.67	0–0	0.00–0.00
Wakayama						
	Apr 5 to Apr 11, 2021	9	0–0	0.00–0.00	0–4	0.00–30.77

	Apr 12 to Apr 18, 2021	16	0–3	0.00–23.08	0–0	0.00–0.00
	Apr 19 to Apr 25, 2021	18	0–5	0.00–38.46	0–0	0.00–0.00
	Apr 26 to May 2, 2021	10	0–0	0.00–0.00	0–3	0.00–23.08
	May 3 to May 9, 2021	14	0–1	0.00–7.69	0–0	0.00–0.00
	May 10 to May 16, 2021	16	0–2	0.00–14.29	0–0	0.00–0.00
	May 17 to May 23, 2021	11	0–0	0.00–0.00	0–3	0.00–21.43
	May 24 to May 30, 2021	18	0–4	0.00–28.57	0–0	0.00–0.00
Tottori						
	Apr 5 to Apr 11, 2021	6	0–0	0.00–0.00	0–4	0.00–40.00
	Apr 12 to Apr 18, 2021	13	0–4	0.00–44.44	0–0	0.00–0.00
	Apr 19 to Apr 25, 2021	9	0–0	0.00–0.00	0–0	0.00–0.00
	Apr 26 to May 2, 2021	10	0–0	0.00–0.00	0–0	0.00–0.00
	May 3 to May 9, 2021	14	0–4	0.00–40.00	0–0	0.00–0.00
	May 10 to May 16, 2021	6	0–0	0.00–0.00	0–5	0.00–45.45
	May 17 to May 23, 2021	12	0–2	0.00–20.00	0–0	0.00–0.00
	May 24 to May 30, 2021	11	0–1	0.00–10.00	0–0	0.00–0.00
Shimane						
	Apr 5 to Apr 11, 2021	13	0–1	0.00–8.33	0–0	0.00–0.00
	Apr 12 to Apr 18, 2021	12	0–0	0.00–0.00	0–0	0.00–0.00
	Apr 19 to Apr 25, 2021	13	0–1	0.00–8.33	0–0	0.00–0.00
	Apr 26 to May 2, 2021	14	0–2	0.00–16.67	0–0	0.00–0.00
	May 3 to May 9, 2021	10	0–0	0.00–0.00	0–1	0.00–9.09
	May 10 to May 16, 2021	12	0–2	0.00–20.00	0–0	0.00–0.00
	May 17 to May 23, 2021	13	0–3	0.00–30.00	0–0	0.00–0.00
	May 24 to May 30, 2021	14	0–4	0.00–40.00	0–0	0.00–0.00
Okayama						
	Apr 5 to Apr 11, 2021	23	0–3	0.00–15.00	0–0	0.00–0.00
	Apr 12 to Apr 18, 2021	22	0–2	0.00–10.00	0–0	0.00–0.00
	Apr 19 to Apr 25, 2021	14	0–0	0.00–0.00	0–5	0.00–26.32
	Apr 26 to May 2, 2021	23	0–4	0.00–21.05	0–0	0.00–0.00
	May 3 to May 9, 2021	16	0–0	0.00–0.00	0–3	0.00–15.79
	May 10 to May 16, 2021	21	0–2	0.00–10.53	0–0	0.00–0.00
	May 17 to May 23, 2021	20	0–1	0.00–5.26	0–0	0.00–0.00
	May 24 to May 30, 2021	19	0–0	0.00–0.00	0–0	0.00–0.00
Hiroshima						
	Apr 5 to Apr 11, 2021	28	0–0	0.00–0.00	0–0	0.00–0.00
	Apr 12 to Apr 18, 2021	27	0–0	0.00–0.00	0–1	0.00–3.57
	Apr 19 to Apr 25, 2021	25	0–0	0.00–0.00	0–3	0.00–10.71
	Apr 26 to May 2, 2021	32	0–5	0.00–18.52	0–0	0.00–0.00
	May 3 to May 9, 2021	36	0–10	0.00–38.46	0–0	0.00–0.00
	May 10 to May 16, 2021	28	0–3	0.00–12.00	0–0	0.00–0.00
	May 17 to May 23, 2021	30	0–5	0.00–20.00	0–0	0.00–0.00
	May 24 to May 30, 2021	23	0–0	0.00–0.00	0–1	0.00–4.17
Yamaguchi						
	Apr 5 to Apr 11, 2021	14	0–0	0.00–0.00	0–2	0.00–12.50
	Apr 12 to Apr 18, 2021	23	0–8	0.00–53.33	0–0	0.00–0.00

	Apr 19 to Apr 25, 2021	15	0–0	0.00–0.00	0–1	0.00–6.25
	Apr 26 to May 2, 2021	25	1–9	6.25–56.25	0–0	0.00–0.00
	May 3 to May 9, 2021	19	0–3	0.00–18.75	0–0	0.00–0.00
	May 10 to May 16, 2021	7	0–0	0.00–0.00	2–9	12.50–56.25
	May 17 to May 23, 2021	19	0–2	0.00–11.76	0–0	0.00–0.00
	May 24 to May 30, 2021	11	0–0	0.00–0.00	0–6	0.00–35.29
Tokushima						
	Apr 5 to Apr 11, 2021	9	0–0	0.00–0.00	0–1	0.00–10.00
	Apr 12 to Apr 18, 2021	12	0–2	0.00–20.00	0–0	0.00–0.00
	Apr 19 to Apr 25, 2021	10	0–1	0.00–11.11	0–0	0.00–0.00
	Apr 26 to May 2, 2021	5	0–0	0.00–0.00	0–4	0.00–44.44
	May 3 to May 9, 2021	7	0–0	0.00–0.00	0–2	0.00–22.22
	May 10 to May 16, 2021	10	0–0	0.00–0.00	0–0	0.00–0.00
	May 17 to May 23, 2021	12	0–3	0.00–33.33	0–0	0.00–0.00
	May 24 to May 30, 2021	6	0–0	0.00–0.00	0–4	0.00–40.00
Kagawa						
	Apr 5 to Apr 11, 2021	11	0–0	0.00–0.00	0–4	0.00–26.67
	Apr 12 to Apr 18, 2021	21	0–6	0.00–40.00	0–0	0.00–0.00
	Apr 19 to Apr 25, 2021	15	0–1	0.00–7.14	0–0	0.00–0.00
	Apr 26 to May 2, 2021	25	2–10	13.33–66.67	0–0	0.00–0.00
	May 3 to May 9, 2021	13	0–0	0.00–0.00	0–2	0.00–13.33
	May 10 to May 16, 2021	16	0–1	0.00–6.67	0–0	0.00–0.00
	May 17 to May 23, 2021	15	0–0	0.00–0.00	0–0	0.00–0.00
	May 24 to May 30, 2021	17	0–2	0.00–13.33	0–0	0.00–0.00
Ehime						
	Apr 5 to Apr 11, 2021	24	0–7	0.00–41.18	0–0	0.00–0.00
	Apr 12 to Apr 18, 2021	16	0–0	0.00–0.00	0–0	0.00–0.00
	Apr 19 to Apr 25, 2021	20	0–4	0.00–25.00	0–0	0.00–0.00
	Apr 26 to May 2, 2021	21	0–5	0.00–31.25	0–0	0.00–0.00
	May 3 to May 9, 2021	20	0–4	0.00–25.00	0–0	0.00–0.00
	May 10 to May 16, 2021	19	0–3	0.00–18.75	0–0	0.00–0.00
	May 17 to May 23, 2021	14	0–0	0.00–0.00	0–1	0.00–6.67
	May 24 to May 30, 2021	14	0–0	0.00–0.00	0–0	0.00–0.00
Kochi						
	Apr 5 to Apr 11, 2021	6	0–1	0.00–20.00	0–0	0.00–0.00
	Apr 12 to Apr 18, 2021	9	0–4	0.00–80.00	0–0	0.00–0.00
	Apr 19 to Apr 25, 2021	8	0–3	0.00–60.00	0–0	0.00–0.00
	Apr 26 to May 2, 2021	5	0–0	0.00–0.00	0–1	0.00–16.67
	May 3 to May 9, 2021	7	0–1	0.00–16.67	0–0	0.00–0.00
	May 10 to May 16, 2021	3	0–0	0.00–0.00	0–3	0.00–50.00
	May 17 to May 23, 2021	6	0–0	0.00–0.00	0–0	0.00–0.00
	May 24 to May 30, 2021	7	0–0	0.00–0.00	0–0	0.00–0.00
Fukuoka						
	Apr 5 to Apr 11, 2021	27	0–0	0.00–0.00	0–6	0.00–18.18
	Apr 12 to Apr 18, 2021	33	0–0	0.00–0.00	0–1	0.00–2.94
	Apr 19 to Apr 25, 2021	29	0–0	0.00–0.00	0–5	0.00–14.71

	Apr 26 to May 2, 2021	37	0–4	0.00–12.12	0–0	0.00–0.00
	May 3 to May 9, 2021	35	0–3	0.00–9.38	0–0	0.00–0.00
	May 10 to May 16, 2021	46	2–14	6.25–43.75	0–0	0.00–0.00
	May 17 to May 23, 2021	28	0–0	0.00–0.00	0–3	0.00–9.68
	May 24 to May 30, 2021	37	0–6	0.00–19.35	0–0	0.00–0.00
Saga						
	Apr 5 to Apr 11, 2021	8	0–1	0.00–14.29	0–0	0.00–0.00
	Apr 12 to Apr 18, 2021	7	0–0	0.00–0.00	0–0	0.00–0.00
	Apr 19 to Apr 25, 2021	6	0–0	0.00–0.00	0–1	0.00–14.29
	Apr 26 to May 2, 2021	7	0–0	0.00–0.00	0–0	0.00–0.00
	May 3 to May 9, 2021	15	3–8	42.86–114.29	0–0	0.00–0.00
	May 10 to May 16, 2021	6	0–0	0.00–0.00	0–1	0.00–14.29
	May 17 to May 23, 2021	9	0–2	0.00–28.57	0–0	0.00–0.00
	May 24 to May 30, 2021	5	0–0	0.00–0.00	0–2	0.00–28.57
Nagasaki						
	Apr 5 to Apr 11, 2021	20	3–10	30.00–100.00	0–0	0.00–0.00
	Apr 12 to Apr 18, 2021	14	0–4	0.00–40.00	0–0	0.00–0.00
	Apr 19 to Apr 25, 2021	17	1–7	10.00–70.00	0–0	0.00–0.00
	Apr 26 to May 2, 2021	10	0–0	0.00–0.00	0–0	0.00–0.00
	May 3 to May 9, 2021	20	5–11	55.56–122.22	0–0	0.00–0.00
	May 10 to May 16, 2021	14	0–5	0.00–55.56	0–0	0.00–0.00
	May 17 to May 23, 2021	9	0–0	0.00–0.00	0–0	0.00–0.00
	May 24 to May 30, 2021	17	2–8	22.22–88.89	0–0	0.00–0.00
Kumamoto						
	Apr 5 to Apr 11, 2021	24	0–7	0.00–41.18	0–0	0.00–0.00
	Apr 12 to Apr 18, 2021	22	0–6	0.00–37.50	0–0	0.00–0.00
	Apr 19 to Apr 25, 2021	17	0–1	0.00–6.25	0–0	0.00–0.00
	Apr 26 to May 2, 2021	22	0–6	0.00–37.50	0–0	0.00–0.00
	May 3 to May 9, 2021	25	0–8	0.00–47.06	0–0	0.00–0.00
	May 10 to May 16, 2021	22	0–5	0.00–29.41	0–0	0.00–0.00
	May 17 to May 23, 2021	18	0–1	0.00–5.88	0–0	0.00–0.00
	May 24 to May 30, 2021	24	0–7	0.00–41.18	0–0	0.00–0.00
Oita						
	Apr 5 to Apr 11, 2021	11	0–0	0.00–0.00	0–6	0.00–35.29
	Apr 12 to Apr 18, 2021	12	0–0	0.00–0.00	0–6	0.00–33.33
	Apr 19 to Apr 25, 2021	19	0–2	0.00–11.76	0–0	0.00–0.00
	Apr 26 to May 2, 2021	20	0–4	0.00–25.00	0–0	0.00–0.00
	May 3 to May 9, 2021	11	0–0	0.00–0.00	0–5	0.00–31.25
	May 10 to May 16, 2021	15	0–0	0.00–0.00	0–1	0.00–6.25
	May 17 to May 23, 2021	12	0–0	0.00–0.00	0–4	0.00–25.00
	May 24 to May 30, 2021	14	0–0	0.00–0.00	0–1	0.00–6.67
Miyazaki						
	Apr 5 to Apr 11, 2021	12	0–0	0.00–0.00	0–1	0.00–7.69
	Apr 12 to Apr 18, 2021	13	0–1	0.00–8.33	0–0	0.00–0.00
	Apr 19 to Apr 25, 2021	17	0–5	0.00–41.67	0–0	0.00–0.00
	Apr 26 to May 2, 2021	10	0–0	0.00–0.00	0–2	0.00–16.67

	May 3 to May 9, 2021	17	0–6	0.00–54.55	0–0	0.00–0.00
	May 10 to May 16, 2021	9	0–0	0.00–0.00	0–2	0.00–18.18
	May 17 to May 23, 2021	14	0–3	0.00–27.27	0–0	0.00–0.00
	May 24 to May 30, 2021	14	0–3	0.00–27.27	0–0	0.00–0.00
Kagoshima						
	Apr 5 to Apr 11, 2021	20	0–3	0.00–17.65	0–0	0.00–0.00
	Apr 12 to Apr 18, 2021	25	0–8	0.00–47.06	0–0	0.00–0.00
	Apr 19 to Apr 25, 2021	16	0–0	0.00–0.00	0–1	0.00–5.88
	Apr 26 to May 2, 2021	22	0–5	0.00–29.41	0–0	0.00–0.00
	May 3 to May 9, 2021	18	0–1	0.00–5.88	0–0	0.00–0.00
	May 10 to May 16, 2021	21	0–4	0.00–23.53	0–0	0.00–0.00
	May 17 to May 23, 2021	19	0–2	0.00–11.76	0–0	0.00–0.00
	May 24 to May 30, 2021	20	0–3	0.00–17.65	0–0	0.00–0.00
Okinawa						
	Apr 5 to Apr 11, 2021	13	0–2	0.00–18.18	0–0	0.00–0.00
	Apr 12 to Apr 18, 2021	16	0–5	0.00–45.45	0–0	0.00–0.00
	Apr 19 to Apr 25, 2021	8	0–0	0.00–0.00	0–2	0.00–20.00
	Apr 26 to May 2, 2021	13	0–2	0.00–18.18	0–0	0.00–0.00
	May 3 to May 9, 2021	10	0–0	0.00–0.00	0–0	0.00–0.00
	May 10 to May 16, 2021	13	0–3	0.00–30.00	0–0	0.00–0.00
	May 17 to May 23, 2021	7	0–0	0.00–0.00	0–3	0.00–30.00
	May 24 to May 30, 2021	18	1–8	10.00–80.00	0–0	0.00–0.00

Table A.20: Weekly number of observed and excess/exiguous deaths in Japan and 47 prefectures for senility-related deaths at homes from January 2020 through May 2021.

Prefecture	Week	Observed	Excess deaths	Percent excess	Exiguous deaths	Percent exiguous
Japan						
	Apr 5 to Apr 11, 2021	432	0–34	0.00–8.54	0–0	0.00–0.00
	Apr 12 to Apr 18, 2021	477	24–85	6.12–21.68	0–0	0.00–0.00
	Apr 19 to Apr 25, 2021	503	63–124	16.62–32.72	0–0	0.00–0.00
	Apr 26 to May 2, 2021	501	71–132	19.24–35.77	0–0	0.00–0.00
	May 3 to May 9, 2021	477	51–113	14.01–31.04	0–0	0.00–0.00
	May 10 to May 16, 2021	498	78–139	21.73–38.72	0–0	0.00–0.00
	May 17 to May 23, 2021	479	67–125	18.93–35.31	0–0	0.00–0.00
	May 24 to May 30, 2021	435	17–78	4.76–21.85	0–0	0.00–0.00
Hokkaido						
	Apr 5 to Apr 11, 2021	20	0–6	0.00–42.86	0–0	0.00–0.00
	Apr 12 to Apr 18, 2021	13	0–0	0.00–0.00	0–1	0.00–7.14
	Apr 19 to Apr 25, 2021	17	0–3	0.00–21.43	0–0	0.00–0.00
	Apr 26 to May 2, 2021	21	0–7	0.00–50.00	0–0	0.00–0.00
	May 3 to May 9, 2021	12	0–0	0.00–0.00	0–2	0.00–14.29
	May 10 to May 16, 2021	12	0–0	0.00–0.00	0–2	0.00–14.29
	May 17 to May 23, 2021	18	0–4	0.00–28.57	0–0	0.00–0.00
	May 24 to May 30, 2021	13	0–0	0.00–0.00	0–2	0.00–13.33
Aomori						
	Apr 5 to Apr 11, 2021	4	0–0	0.00–0.00	0–1	0.00–20.00
	Apr 12 to Apr 18, 2021	2	0–0	0.00–0.00	0–2	0.00–50.00
	Apr 19 to Apr 25, 2021	3	0–0	0.00–0.00	0–1	0.00–25.00
	Apr 26 to May 2, 2021	2	0–0	0.00–0.00	0–2	0.00–50.00
	May 3 to May 9, 2021	6	0–1	0.00–20.00	0–0	0.00–0.00
	May 10 to May 16, 2021	7	0–2	0.00–40.00	0–0	0.00–0.00
	May 17 to May 23, 2021	7	0–2	0.00–40.00	0–0	0.00–0.00
	May 24 to May 30, 2021	2	0–0	0.00–0.00	0–3	0.00–60.00
Iwate						
	Apr 5 to Apr 11, 2021	7	0–3	0.00–75.00	0–0	0.00–0.00
	Apr 12 to Apr 18, 2021	4	0–0	0.00–0.00	0–0	0.00–0.00
	Apr 19 to Apr 25, 2021	2	0–0	0.00–0.00	0–2	0.00–50.00
	Apr 26 to May 2, 2021	2	0–0	0.00–0.00	0–2	0.00–50.00
	May 3 to May 9, 2021	4	0–0	0.00–0.00	0–1	0.00–20.00
	May 10 to May 16, 2021	6	0–2	0.00–50.00	0–0	0.00–0.00
	May 17 to May 23, 2021	6	0–1	0.00–20.00	0–0	0.00–0.00
	May 24 to May 30, 2021	3	0–0	0.00–0.00	0–2	0.00–40.00
Miyagi						
	Apr 5 to Apr 11, 2021	12	0–2	0.00–20.00	0–0	0.00–0.00
	Apr 12 to Apr 18, 2021	15	0–5	0.00–50.00	0–0	0.00–0.00
	Apr 19 to Apr 25, 2021	12	0–2	0.00–20.00	0–0	0.00–0.00
	Apr 26 to May 2, 2021	7	0–0	0.00–0.00	0–3	0.00–30.00
	May 3 to May 9, 2021	9	0–0	0.00–0.00	0–0	0.00–0.00

	May 10 to May 16, 2021	13	0–4	0.00–44.44	0–0	0.00–0.00
	May 17 to May 23, 2021	9	0–0	0.00–0.00	0–0	0.00–0.00
	May 24 to May 30, 2021	11	0–3	0.00–37.50	0–0	0.00–0.00
Akita						
	Apr 5 to Apr 11, 2021	2	0–0	0.00–0.00	0–1	0.00–33.33
	Apr 12 to Apr 18, 2021	4	0–1	0.00–33.33	0–0	0.00–0.00
	Apr 19 to Apr 25, 2021	3	0–0	0.00–0.00	0–0	0.00–0.00
	Apr 26 to May 2, 2021	2	0–0	0.00–0.00	0–1	0.00–33.33
	May 3 to May 9, 2021	4	0–1	0.00–33.33	0–0	0.00–0.00
	May 10 to May 16, 2021	4	0–1	0.00–33.33	0–0	0.00–0.00
	May 17 to May 23, 2021	2	0–0	0.00–0.00	0–1	0.00–33.33
	May 24 to May 30, 2021	2	0–0	0.00–0.00	0–1	0.00–33.33
Yamagata						
	Apr 5 to Apr 11, 2021	4	0–0	0.00–0.00	0–1	0.00–20.00
	Apr 12 to Apr 18, 2021	1	0–0	0.00–0.00	0–4	0.00–80.00
	Apr 19 to Apr 25, 2021	7	0–2	0.00–40.00	0–0	0.00–0.00
	Apr 26 to May 2, 2021	6	0–2	0.00–50.00	0–0	0.00–0.00
	May 3 to May 9, 2021	3	0–0	0.00–0.00	0–1	0.00–25.00
	May 10 to May 16, 2021	8	0–4	0.00–100.00	0–0	0.00–0.00
	May 17 to May 23, 2021	3	0–0	0.00–0.00	0–1	0.00–25.00
	May 24 to May 30, 2021	10	2–6	50.00–150.00	0–0	0.00–0.00
Fukushima						
	Apr 5 to Apr 11, 2021	12	0–2	0.00–20.00	0–0	0.00–0.00
	Apr 12 to Apr 18, 2021	10	0–0	0.00–0.00	0–0	0.00–0.00
	Apr 19 to Apr 25, 2021	5	0–0	0.00–0.00	0–4	0.00–44.44
	Apr 26 to May 2, 2021	10	0–1	0.00–11.11	0–0	0.00–0.00
	May 3 to May 9, 2021	8	0–0	0.00–0.00	0–1	0.00–11.11
	May 10 to May 16, 2021	9	0–0	0.00–0.00	0–0	0.00–0.00
	May 17 to May 23, 2021	7	0–0	0.00–0.00	0–1	0.00–12.50
	May 24 to May 30, 2021	6	0–0	0.00–0.00	0–2	0.00–25.00
Ibaraki						
	Apr 5 to Apr 11, 2021	7	0–0	0.00–0.00	0–4	0.00–36.36
	Apr 12 to Apr 18, 2021	8	0–0	0.00–0.00	0–3	0.00–27.27
	Apr 19 to Apr 25, 2021	11	0–1	0.00–10.00	0–0	0.00–0.00
	Apr 26 to May 2, 2021	15	0–5	0.00–50.00	0–0	0.00–0.00
	May 3 to May 9, 2021	8	0–0	0.00–0.00	0–1	0.00–11.11
	May 10 to May 16, 2021	10	0–1	0.00–11.11	0–0	0.00–0.00
	May 17 to May 23, 2021	10	0–1	0.00–11.11	0–0	0.00–0.00
	May 24 to May 30, 2021	10	0–1	0.00–11.11	0–0	0.00–0.00
Tochigi						
	Apr 5 to Apr 11, 2021	8	0–0	0.00–0.00	0–1	0.00–11.11
	Apr 12 to Apr 18, 2021	9	0–0	0.00–0.00	0–0	0.00–0.00
	Apr 19 to Apr 25, 2021	14	0–5	0.00–55.56	0–0	0.00–0.00
	Apr 26 to May 2, 2021	3	0–0	0.00–0.00	0–5	0.00–62.50
	May 3 to May 9, 2021	6	0–0	0.00–0.00	0–2	0.00–25.00
	May 10 to May 16, 2021	13	0–5	0.00–62.50	0–0	0.00–0.00

	May 17 to May 23, 2021	8	0–0	0.00–0.00	0–0	0.00–0.00
	May 24 to May 30, 2021	13	0–5	0.00–62.50	0–0	0.00–0.00
Gunma						
	Apr 5 to Apr 11, 2021	3	0–0	0.00–0.00	0–4	0.00–57.14
	Apr 12 to Apr 18, 2021	11	0–4	0.00–57.14	0–0	0.00–0.00
	Apr 19 to Apr 25, 2021	7	0–0	0.00–0.00	0–0	0.00–0.00
	Apr 26 to May 2, 2021	7	0–1	0.00–16.67	0–0	0.00–0.00
	May 3 to May 9, 2021	7	0–1	0.00–16.67	0–0	0.00–0.00
	May 10 to May 16, 2021	4	0–0	0.00–0.00	0–2	0.00–33.33
	May 17 to May 23, 2021	6	0–0	0.00–0.00	0–0	0.00–0.00
	May 24 to May 30, 2021	5	0–0	0.00–0.00	0–1	0.00–16.67
Saitama						
	Apr 5 to Apr 11, 2021	22	0–1	0.00–4.76	0–0	0.00–0.00
	Apr 12 to Apr 18, 2021	29	0–9	0.00–45.00	0–0	0.00–0.00
	Apr 19 to Apr 25, 2021	24	0–4	0.00–20.00	0–0	0.00–0.00
	Apr 26 to May 2, 2021	32	3–13	15.79–68.42	0–0	0.00–0.00
	May 3 to May 9, 2021	23	0–4	0.00–21.05	0–0	0.00–0.00
	May 10 to May 16, 2021	29	0–9	0.00–45.00	0–0	0.00–0.00
	May 17 to May 23, 2021	16	0–0	0.00–0.00	0–3	0.00–15.79
	May 24 to May 30, 2021	17	0–0	0.00–0.00	0–2	0.00–10.53
Chiba						
	Apr 5 to Apr 11, 2021	17	0–0	0.00–0.00	0–3	0.00–15.00
	Apr 12 to Apr 18, 2021	20	0–0	0.00–0.00	0–1	0.00–4.76
	Apr 19 to Apr 25, 2021	21	0–0	0.00–0.00	0–0	0.00–0.00
	Apr 26 to May 2, 2021	28	0–8	0.00–40.00	0–0	0.00–0.00
	May 3 to May 9, 2021	23	0–3	0.00–15.00	0–0	0.00–0.00
	May 10 to May 16, 2021	25	0–6	0.00–31.58	0–0	0.00–0.00
	May 17 to May 23, 2021	22	0–3	0.00–15.79	0–0	0.00–0.00
	May 24 to May 30, 2021	15	0–0	0.00–0.00	0–4	0.00–21.05
Tokyo						
	Apr 5 to Apr 11, 2021	48	0–0	0.00–0.00	0–3	0.00–5.88
	Apr 12 to Apr 18, 2021	60	0–10	0.00–20.00	0–0	0.00–0.00
	Apr 19 to Apr 25, 2021	66	3–18	6.25–37.50	0–0	0.00–0.00
	Apr 26 to May 2, 2021	63	5–19	11.36–43.18	0–0	0.00–0.00
	May 3 to May 9, 2021	59	1–14	2.22–31.11	0–0	0.00–0.00
	May 10 to May 16, 2021	55	0–11	0.00–25.00	0–0	0.00–0.00
	May 17 to May 23, 2021	52	0–8	0.00–18.18	0–0	0.00–0.00
	May 24 to May 30, 2021	49	0–5	0.00–11.36	0–0	0.00–0.00
Kanagawa						
	Apr 5 to Apr 11, 2021	46	0–3	0.00–6.98	0–0	0.00–0.00
	Apr 12 to Apr 18, 2021	49	0–6	0.00–13.95	0–0	0.00–0.00
	Apr 19 to Apr 25, 2021	43	0–0	0.00–0.00	0–1	0.00–2.27
	Apr 26 to May 2, 2021	56	0–13	0.00–30.23	0–0	0.00–0.00
	May 3 to May 9, 2021	42	0–1	0.00–2.44	0–0	0.00–0.00
	May 10 to May 16, 2021	55	0–14	0.00–34.15	0–0	0.00–0.00
	May 17 to May 23, 2021	37	0–0	0.00–0.00	0–3	0.00–7.50

	May 24 to May 30, 2021	54	1–16	2.63–42.11	0–0	0.00–0.00
Niigata						
	Apr 5 to Apr 11, 2021	8	0–1	0.00–14.29	0–0	0.00–0.00
	Apr 12 to Apr 18, 2021	6	0–0	0.00–0.00	0–1	0.00–14.29
	Apr 19 to Apr 25, 2021	8	0–1	0.00–14.29	0–0	0.00–0.00
	Apr 26 to May 2, 2021	7	0–0	0.00–0.00	0–0	0.00–0.00
	May 3 to May 9, 2021	12	0–5	0.00–71.43	0–0	0.00–0.00
	May 10 to May 16, 2021	7	0–0	0.00–0.00	0–0	0.00–0.00
	May 17 to May 23, 2021	3	0–0	0.00–0.00	0–4	0.00–57.14
	May 24 to May 30, 2021	9	0–2	0.00–28.57	0–0	0.00–0.00
Toyama						
	Apr 5 to Apr 11, 2021	1	0–0	0.00–0.00	0–2	0.00–66.67
	Apr 12 to Apr 18, 2021	2	0–0	0.00–0.00	0–1	0.00–33.33
	Apr 19 to Apr 25, 2021	4	0–1	0.00–33.33	0–0	0.00–0.00
	Apr 26 to May 2, 2021	10	4–7	133.33–233.33	0–0	0.00–0.00
	May 3 to May 9, 2021	3	0–1	0.00–50.00	0–0	0.00–0.00
	May 10 to May 16, 2021	3	0–1	0.00–50.00	0–0	0.00–0.00
	May 17 to May 23, 2021	3	0–1	0.00–50.00	0–0	0.00–0.00
	May 24 to May 30, 2021	2	0–0	0.00–0.00	0–1	0.00–33.33
Ishikawa						
	Apr 5 to Apr 11, 2021	2	0–0	0.00–0.00	0–1	0.00–33.33
	Apr 12 to Apr 18, 2021	5	0–2	0.00–66.67	0–0	0.00–0.00
	Apr 19 to Apr 25, 2021	4	0–1	0.00–33.33	0–0	0.00–0.00
	Apr 26 to May 2, 2021	1	0–0	0.00–0.00	0–2	0.00–66.67
	May 3 to May 9, 2021	2	0–0	0.00–0.00	0–1	0.00–33.33
	May 10 to May 16, 2021	3	0–0	0.00–0.00	0–0	0.00–0.00
	May 17 to May 23, 2021	2	0–0	0.00–0.00	0–1	0.00–33.33
	May 24 to May 30, 2021	4	0–1	0.00–33.33	0–0	0.00–0.00
Fukui						
	Apr 5 to Apr 11, 2021	1	0–0	0.00–0.00	0–2	0.00–66.67
	Apr 12 to Apr 18, 2021	1	0–0	0.00–0.00	0–2	0.00–66.67
	Apr 19 to Apr 25, 2021	4	0–1	0.00–33.33	0–0	0.00–0.00
	Apr 26 to May 2, 2021	2	0–0	0.00–0.00	0–0	0.00–0.00
	May 3 to May 9, 2021	3	0–0	0.00–0.00	0–0	0.00–0.00
	May 10 to May 16, 2021	3	0–0	0.00–0.00	0–0	0.00–0.00
	May 17 to May 23, 2021	0	0–0	0.00–0.00	0–3	0.00–100.00
	May 24 to May 30, 2021	3	0–0	0.00–0.00	0–0	0.00–0.00
Yamanashi						
	Apr 5 to Apr 11, 2021	6	0–3	0.00–100.00	0–0	0.00–0.00
	Apr 12 to Apr 18, 2021	2	0–0	0.00–0.00	0–1	0.00–33.33
	Apr 19 to Apr 25, 2021	2	0–0	0.00–0.00	0–1	0.00–33.33
	Apr 26 to May 2, 2021	6	0–3	0.00–100.00	0–0	0.00–0.00
	May 3 to May 9, 2021	4	0–2	0.00–100.00	0–0	0.00–0.00
	May 10 to May 16, 2021	3	0–1	0.00–50.00	0–0	0.00–0.00
	May 17 to May 23, 2021	4	0–2	0.00–100.00	0–0	0.00–0.00
	May 24 to May 30, 2021	2	0–0	0.00–0.00	0–0	0.00–0.00

Nagano					
	Apr 5 to Apr 11, 2021	13	0–4	0.00–44.44	0–0	0.00–0.00
	Apr 12 to Apr 18, 2021	11	0–2	0.00–22.22	0–0	0.00–0.00
	Apr 19 to Apr 25, 2021	12	0–3	0.00–33.33	0–0	0.00–0.00
	Apr 26 to May 2, 2021	18	3–9	33.33–100.00	0–0	0.00–0.00
	May 3 to May 9, 2021	9	0–0	0.00–0.00	0–0	0.00–0.00
	May 10 to May 16, 2021	4	0–0	0.00–0.00	0–4	0.00–50.00
	May 17 to May 23, 2021	19	5–11	62.50–137.50	0–0	0.00–0.00
	May 24 to May 30, 2021	15	1–7	12.50–87.50	0–0	0.00–0.00
Gifu					
	Apr 5 to Apr 11, 2021	8	0–0	0.00–0.00	0–0	0.00–0.00
	Apr 12 to Apr 18, 2021	9	0–2	0.00–28.57	0–0	0.00–0.00
	Apr 19 to Apr 25, 2021	11	0–4	0.00–57.14	0–0	0.00–0.00
	Apr 26 to May 2, 2021	9	0–2	0.00–28.57	0–0	0.00–0.00
	May 3 to May 9, 2021	5	0–0	0.00–0.00	0–2	0.00–28.57
	May 10 to May 16, 2021	7	0–0	0.00–0.00	0–0	0.00–0.00
	May 17 to May 23, 2021	9	0–2	0.00–28.57	0–0	0.00–0.00
	May 24 to May 30, 2021	7	0–0	0.00–0.00	0–0	0.00–0.00
Shizuoka					
	Apr 5 to Apr 11, 2021	24	0–7	0.00–41.18	0–0	0.00–0.00
	Apr 12 to Apr 18, 2021	22	0–6	0.00–37.50	0–0	0.00–0.00
	Apr 19 to Apr 25, 2021	25	0–9	0.00–56.25	0–0	0.00–0.00
	Apr 26 to May 2, 2021	23	0–7	0.00–43.75	0–0	0.00–0.00
	May 3 to May 9, 2021	21	0–6	0.00–40.00	0–0	0.00–0.00
	May 10 to May 16, 2021	23	0–9	0.00–64.29	0–0	0.00–0.00
	May 17 to May 23, 2021	29	6–15	42.86–107.14	0–0	0.00–0.00
	May 24 to May 30, 2021	19	0–4	0.00–26.67	0–0	0.00–0.00
Aichi					
	Apr 5 to Apr 11, 2021	20	0–0	0.00–0.00	0–2	0.00–9.09
	Apr 12 to Apr 18, 2021	25	0–3	0.00–13.64	0–0	0.00–0.00
	Apr 19 to Apr 25, 2021	33	2–11	9.09–50.00	0–0	0.00–0.00
	Apr 26 to May 2, 2021	21	0–0	0.00–0.00	0–0	0.00–0.00
	May 3 to May 9, 2021	26	0–5	0.00–23.81	0–0	0.00–0.00
	May 10 to May 16, 2021	23	0–2	0.00–9.52	0–0	0.00–0.00
	May 17 to May 23, 2021	27	0–7	0.00–35.00	0–0	0.00–0.00
	May 24 to May 30, 2021	22	0–3	0.00–15.79	0–0	0.00–0.00
Mie					
	Apr 5 to Apr 11, 2021	5	0–0	0.00–0.00	0–2	0.00–28.57
	Apr 12 to Apr 18, 2021	7	0–0	0.00–0.00	0–0	0.00–0.00
	Apr 19 to Apr 25, 2021	8	0–1	0.00–14.29	0–0	0.00–0.00
	Apr 26 to May 2, 2021	8	0–1	0.00–14.29	0–0	0.00–0.00
	May 3 to May 9, 2021	10	0–3	0.00–42.86	0–0	0.00–0.00
	May 10 to May 16, 2021	14	1–7	14.29–100.00	0–0	0.00–0.00
	May 17 to May 23, 2021	11	0–4	0.00–57.14	0–0	0.00–0.00
	May 24 to May 30, 2021	7	0–0	0.00–0.00	0–0	0.00–0.00
Shiga					

	Apr 5 to Apr 11, 2021	4	0–0	0.00–0.00	0–0	0.00–0.00
	Apr 12 to Apr 18, 2021	4	0–0	0.00–0.00	0–0	0.00–0.00
	Apr 19 to Apr 25, 2021	6	0–2	0.00–50.00	0–0	0.00–0.00
	Apr 26 to May 2, 2021	4	0–0	0.00–0.00	0–0	0.00–0.00
	May 3 to May 9, 2021	6	0–2	0.00–50.00	0–0	0.00–0.00
	May 10 to May 16, 2021	5	0–1	0.00–25.00	0–0	0.00–0.00
	May 17 to May 23, 2021	4	0–0	0.00–0.00	0–0	0.00–0.00
	May 24 to May 30, 2021	6	0–2	0.00–50.00	0–0	0.00–0.00
Kyoto						
	Apr 5 to Apr 11, 2021	12	0–4	0.00–50.00	0–0	0.00–0.00
	Apr 12 to Apr 18, 2021	11	0–3	0.00–37.50	0–0	0.00–0.00
	Apr 19 to Apr 25, 2021	13	0–5	0.00–62.50	0–0	0.00–0.00
	Apr 26 to May 2, 2021	13	0–6	0.00–85.71	0–0	0.00–0.00
	May 3 to May 9, 2021	14	1–7	14.29–100.00	0–0	0.00–0.00
	May 10 to May 16, 2021	9	0–2	0.00–28.57	0–0	0.00–0.00
	May 17 to May 23, 2021	11	0–4	0.00–57.14	0–0	0.00–0.00
	May 24 to May 30, 2021	4	0–0	0.00–0.00	0–3	0.00–42.86
Osaka						
	Apr 5 to Apr 11, 2021	17	0–0	0.00–0.00	0–5	0.00–22.73
	Apr 12 to Apr 18, 2021	28	0–7	0.00–33.33	0–0	0.00–0.00
	Apr 19 to Apr 25, 2021	27	0–6	0.00–28.57	0–0	0.00–0.00
	Apr 26 to May 2, 2021	29	0–9	0.00–45.00	0–0	0.00–0.00
	May 3 to May 9, 2021	31	2–12	10.53–63.16	0–0	0.00–0.00
	May 10 to May 16, 2021	33	4–14	21.05–73.68	0–0	0.00–0.00
	May 17 to May 23, 2021	30	0–10	0.00–50.00	0–0	0.00–0.00
	May 24 to May 30, 2021	25	0–5	0.00–25.00	0–0	0.00–0.00
Hyogo						
	Apr 5 to Apr 11, 2021	31	4–13	22.22–72.22	0–0	0.00–0.00
	Apr 12 to Apr 18, 2021	31	5–13	27.78–72.22	0–0	0.00–0.00
	Apr 19 to Apr 25, 2021	27	2–10	11.76–58.82	0–0	0.00–0.00
	Apr 26 to May 2, 2021	25	0–8	0.00–47.06	0–0	0.00–0.00
	May 3 to May 9, 2021	26	1–10	6.25–62.50	0–0	0.00–0.00
	May 10 to May 16, 2021	25	0–9	0.00–56.25	0–0	0.00–0.00
	May 17 to May 23, 2021	30	5–14	31.25–87.50	0–0	0.00–0.00
	May 24 to May 30, 2021	28	4–12	25.00–75.00	0–0	0.00–0.00
Nara						
	Apr 5 to Apr 11, 2021	6	0–1	0.00–20.00	0–0	0.00–0.00
	Apr 12 to Apr 18, 2021	3	0–0	0.00–0.00	0–2	0.00–40.00
	Apr 19 to Apr 25, 2021	7	0–2	0.00–40.00	0–0	0.00–0.00
	Apr 26 to May 2, 2021	5	0–0	0.00–0.00	0–0	0.00–0.00
	May 3 to May 9, 2021	4	0–0	0.00–0.00	0–0	0.00–0.00
	May 10 to May 16, 2021	7	0–2	0.00–40.00	0–0	0.00–0.00
	May 17 to May 23, 2021	8	0–4	0.00–100.00	0–0	0.00–0.00
	May 24 to May 30, 2021	6	0–2	0.00–50.00	0–0	0.00–0.00
Wakayama						
	Apr 5 to Apr 11, 2021	3	0–0	0.00–0.00	0–1	0.00–25.00

	Apr 12 to Apr 18, 2021	4	0–0	0.00–0.00	0–0	0.00–0.00
	Apr 19 to Apr 25, 2021	4	0–1	0.00–33.33	0–0	0.00–0.00
	Apr 26 to May 2, 2021	2	0–0	0.00–0.00	0–1	0.00–33.33
	May 3 to May 9, 2021	5	0–1	0.00–25.00	0–0	0.00–0.00
	May 10 to May 16, 2021	8	1–5	33.33–166.67	0–0	0.00–0.00
	May 17 to May 23, 2021	5	0–1	0.00–25.00	0–0	0.00–0.00
	May 24 to May 30, 2021	2	0–0	0.00–0.00	0–2	0.00–50.00
Tottori						
	Apr 5 to Apr 11, 2021	2	0–0	0.00–0.00	0–1	0.00–33.33
	Apr 12 to Apr 18, 2021	3	0–1	0.00–50.00	0–0	0.00–0.00
	Apr 19 to Apr 25, 2021	2	0–0	0.00–0.00	0–0	0.00–0.00
	Apr 26 to May 2, 2021	0	0–0	0.00–0.00	0–2	0.00–100.00
	May 3 to May 9, 2021	3	0–1	0.00–50.00	0–0	0.00–0.00
	May 10 to May 16, 2021	4	0–2	0.00–100.00	0–0	0.00–0.00
	May 17 to May 23, 2021	3	0–1	0.00–50.00	0–0	0.00–0.00
	May 24 to May 30, 2021	2	0–0	0.00–0.00	0–0	0.00–0.00
Shimane						
	Apr 5 to Apr 11, 2021	2	0–0	0.00–0.00	0–1	0.00–33.33
	Apr 12 to Apr 18, 2021	4	0–1	0.00–33.33	0–0	0.00–0.00
	Apr 19 to Apr 25, 2021	1	0–0	0.00–0.00	0–1	0.00–50.00
	Apr 26 to May 2, 2021	0	0–0	0.00–0.00	0–2	0.00–100.00
	May 3 to May 9, 2021	4	0–2	0.00–100.00	0–0	0.00–0.00
	May 10 to May 16, 2021	5	0–3	0.00–150.00	0–0	0.00–0.00
	May 17 to May 23, 2021	4	0–2	0.00–100.00	0–0	0.00–0.00
	May 24 to May 30, 2021	1	0–0	0.00–0.00	0–1	0.00–50.00
Okayama						
	Apr 5 to Apr 11, 2021	8	0–4	0.00–100.00	0–0	0.00–0.00
	Apr 12 to Apr 18, 2021	7	0–3	0.00–75.00	0–0	0.00–0.00
	Apr 19 to Apr 25, 2021	4	0–0	0.00–0.00	0–0	0.00–0.00
	Apr 26 to May 2, 2021	4	0–0	0.00–0.00	0–0	0.00–0.00
	May 3 to May 9, 2021	7	0–3	0.00–75.00	0–0	0.00–0.00
	May 10 to May 16, 2021	4	0–0	0.00–0.00	0–0	0.00–0.00
	May 17 to May 23, 2021	6	0–2	0.00–50.00	0–0	0.00–0.00
	May 24 to May 30, 2021	9	1–5	25.00–125.00	0–0	0.00–0.00
Hiroshima						
	Apr 5 to Apr 11, 2021	7	0–0	0.00–0.00	0–0	0.00–0.00
	Apr 12 to Apr 18, 2021	5	0–0	0.00–0.00	0–1	0.00–16.67
	Apr 19 to Apr 25, 2021	4	0–0	0.00–0.00	0–2	0.00–33.33
	Apr 26 to May 2, 2021	7	0–1	0.00–16.67	0–0	0.00–0.00
	May 3 to May 9, 2021	11	0–5	0.00–83.33	0–0	0.00–0.00
	May 10 to May 16, 2021	6	0–0	0.00–0.00	0–0	0.00–0.00
	May 17 to May 23, 2021	7	0–1	0.00–16.67	0–0	0.00–0.00
	May 24 to May 30, 2021	5	0–0	0.00–0.00	0–1	0.00–16.67
Yamaguchi						
	Apr 5 to Apr 11, 2021	1	0–0	0.00–0.00	0–3	0.00–75.00
	Apr 12 to Apr 18, 2021	2	0–0	0.00–0.00	0–2	0.00–50.00

	Apr 19 to Apr 25, 2021	5	0–1	0.00–25.00	0–0	0.00–0.00
	Apr 26 to May 2, 2021	3	0–0	0.00–0.00	0–1	0.00–25.00
	May 3 to May 9, 2021	3	0–0	0.00–0.00	0–0	0.00–0.00
	May 10 to May 16, 2021	1	0–0	0.00–0.00	0–2	0.00–66.67
	May 17 to May 23, 2021	6	0–3	0.00–100.00	0–0	0.00–0.00
	May 24 to May 30, 2021	1	0–0	0.00–0.00	0–2	0.00–66.67
Tokushima						
	Apr 5 to Apr 11, 2021	1	0–0	0.00–0.00	0–1	0.00–50.00
	Apr 12 to Apr 18, 2021	3	0–1	0.00–50.00	0–0	0.00–0.00
	Apr 19 to Apr 25, 2021	1	0–0	0.00–0.00	0–1	0.00–50.00
	Apr 26 to May 2, 2021	2	0–0	0.00–0.00	0–0	0.00–0.00
	May 3 to May 9, 2021	1	0–0	0.00–0.00	0–1	0.00–50.00
	May 10 to May 16, 2021	2	0–0	0.00–0.00	0–0	0.00–0.00
	May 17 to May 23, 2021	2	0–0	0.00–0.00	0–0	0.00–0.00
	May 24 to May 30, 2021	0	0–0	0.00–0.00	0–2	0.00–100.00
Kagawa						
	Apr 5 to Apr 11, 2021	2	0–0	0.00–0.00	0–2	0.00–50.00
	Apr 12 to Apr 18, 2021	5	0–0	0.00–0.00	0–0	0.00–0.00
	Apr 19 to Apr 25, 2021	6	0–2	0.00–50.00	0–0	0.00–0.00
	Apr 26 to May 2, 2021	6	0–2	0.00–50.00	0–0	0.00–0.00
	May 3 to May 9, 2021	3	0–0	0.00–0.00	0–1	0.00–25.00
	May 10 to May 16, 2021	3	0–0	0.00–0.00	0–1	0.00–25.00
	May 17 to May 23, 2021	8	0–4	0.00–100.00	0–0	0.00–0.00
	May 24 to May 30, 2021	3	0–0	0.00–0.00	0–1	0.00–25.00
Ehime						
	Apr 5 to Apr 11, 2021	6	0–0	0.00–0.00	0–2	0.00–25.00
	Apr 12 to Apr 18, 2021	8	0–1	0.00–14.29	0–0	0.00–0.00
	Apr 19 to Apr 25, 2021	8	0–1	0.00–14.29	0–0	0.00–0.00
	Apr 26 to May 2, 2021	10	0–3	0.00–42.86	0–0	0.00–0.00
	May 3 to May 9, 2021	11	0–4	0.00–57.14	0–0	0.00–0.00
	May 10 to May 16, 2021	7	0–0	0.00–0.00	0–0	0.00–0.00
	May 17 to May 23, 2021	5	0–0	0.00–0.00	0–2	0.00–28.57
	May 24 to May 30, 2021	9	0–2	0.00–28.57	0–0	0.00–0.00
Kochi						
	Apr 5 to Apr 11, 2021	2	0–1	0.00–100.00	0–0	0.00–0.00
	Apr 12 to Apr 18, 2021	0	0–0	0.00–0.00	0–1	0.00–100.00
	Apr 19 to Apr 25, 2021	5	1–4	100.00–400.00	0–0	0.00–0.00
	Apr 26 to May 2, 2021	0	0–0	0.00–0.00	0–1	0.00–100.00
	May 3 to May 9, 2021	3	0–2	0.00–200.00	0–0	0.00–0.00
	May 10 to May 16, 2021	0	0–0	0.00–0.00	0–1	0.00–100.00
	May 17 to May 23, 2021	0	NA	NA	NA	NA
	May 24 to May 30, 2021	1	NA	NA	NA	NA
Fukuoka						
	Apr 5 to Apr 11, 2021	7	0–0	0.00–0.00	0–2	0.00–22.22
	Apr 12 to Apr 18, 2021	8	0–0	0.00–0.00	0–1	0.00–11.11
	Apr 19 to Apr 25, 2021	10	0–2	0.00–25.00	0–0	0.00–0.00

	Apr 26 to May 2, 2021	14	0–6	0.00–75.00	0–0	0.00–0.00
	May 3 to May 9, 2021	11	0–3	0.00–37.50	0–0	0.00–0.00
	May 10 to May 16, 2021	16	2–8	25.00–100.00	0–0	0.00–0.00
	May 17 to May 23, 2021	12	0–4	0.00–50.00	0–0	0.00–0.00
	May 24 to May 30, 2021	7	0–0	0.00–0.00	0–1	0.00–12.50
Saga						
	Apr 5 to Apr 11, 2021	4	0–1	0.00–33.33	0–0	0.00–0.00
	Apr 12 to Apr 18, 2021	4	0–1	0.00–33.33	0–0	0.00–0.00
	Apr 19 to Apr 25, 2021	1	0–0	0.00–0.00	0–2	0.00–66.67
	Apr 26 to May 2, 2021	1	0–0	0.00–0.00	0–2	0.00–66.67
	May 3 to May 9, 2021	1	0–0	0.00–0.00	0–2	0.00–66.67
	May 10 to May 16, 2021	0	NA	NA	NA	NA
	May 17 to May 23, 2021	3	0–0	0.00–0.00	0–0	0.00–0.00
	May 24 to May 30, 2021	3	0–0	0.00–0.00	0–0	0.00–0.00
Nagasaki						
	Apr 5 to Apr 11, 2021	3	0–0	0.00–0.00	0–1	0.00–25.00
	Apr 12 to Apr 18, 2021	2	0–0	0.00–0.00	0–2	0.00–50.00
	Apr 19 to Apr 25, 2021	6	0–2	0.00–50.00	0–0	0.00–0.00
	Apr 26 to May 2, 2021	3	0–0	0.00–0.00	0–1	0.00–25.00
	May 3 to May 9, 2021	6	0–2	0.00–50.00	0–0	0.00–0.00
	May 10 to May 16, 2021	2	0–0	0.00–0.00	0–3	0.00–60.00
	May 17 to May 23, 2021	4	0–0	0.00–0.00	0–1	0.00–20.00
	May 24 to May 30, 2021	4	0–0	0.00–0.00	0–0	0.00–0.00
Kumamoto						
	Apr 5 to Apr 11, 2021	3	0–0	0.00–0.00	0–2	0.00–40.00
	Apr 12 to Apr 18, 2021	7	0–2	0.00–40.00	0–0	0.00–0.00
	Apr 19 to Apr 25, 2021	5	0–0	0.00–0.00	0–0	0.00–0.00
	Apr 26 to May 2, 2021	3	0–0	0.00–0.00	0–2	0.00–40.00
	May 3 to May 9, 2021	5	0–0	0.00–0.00	0–0	0.00–0.00
	May 10 to May 16, 2021	4	0–0	0.00–0.00	0–1	0.00–20.00
	May 17 to May 23, 2021	5	0–0	0.00–0.00	0–0	0.00–0.00
	May 24 to May 30, 2021	5	0–0	0.00–0.00	0–0	0.00–0.00
Oita						
	Apr 5 to Apr 11, 2021	1	0–0	0.00–0.00	0–2	0.00–66.67
	Apr 12 to Apr 18, 2021	3	0–0	0.00–0.00	0–0	0.00–0.00
	Apr 19 to Apr 25, 2021	3	0–1	0.00–50.00	0–0	0.00–0.00
	Apr 26 to May 2, 2021	0	0–0	0.00–0.00	0–2	0.00–100.00
	May 3 to May 9, 2021	4	0–2	0.00–100.00	0–0	0.00–0.00
	May 10 to May 16, 2021	5	0–3	0.00–150.00	0–0	0.00–0.00
	May 17 to May 23, 2021	3	0–1	0.00–50.00	0–0	0.00–0.00
	May 24 to May 30, 2021	2	0–0	0.00–0.00	0–0	0.00–0.00
Miyazaki						
	Apr 5 to Apr 11, 2021	4	0–1	0.00–33.33	0–0	0.00–0.00
	Apr 12 to Apr 18, 2021	2	0–0	0.00–0.00	0–1	0.00–33.33
	Apr 19 to Apr 25, 2021	0	0–0	0.00–0.00	0–2	0.00–100.00
	Apr 26 to May 2, 2021	4	0–2	0.00–100.00	0–0	0.00–0.00

	May 3 to May 9, 2021	2	0–0	0.00–0.00	0–0	0.00–0.00
	May 10 to May 16, 2021	5	0–3	0.00–150.00	0–0	0.00–0.00
	May 17 to May 23, 2021	2	0–0	0.00–0.00	0–0	0.00–0.00
	May 24 to May 30, 2021	3	0–1	0.00–50.00	0–0	0.00–0.00
Kagoshima						
	Apr 5 to Apr 11, 2021	5	0–0	0.00–0.00	0–0	0.00–0.00
	Apr 12 to Apr 18, 2021	5	0–0	0.00–0.00	0–0	0.00–0.00
	Apr 19 to Apr 25, 2021	5	0–0	0.00–0.00	0–0	0.00–0.00
	Apr 26 to May 2, 2021	2	0–0	0.00–0.00	0–3	0.00–60.00
	May 3 to May 9, 2021	4	0–0	0.00–0.00	0–1	0.00–20.00
	May 10 to May 16, 2021	3	0–0	0.00–0.00	0–3	0.00–50.00
	May 17 to May 23, 2021	5	0–0	0.00–0.00	0–1	0.00–16.67
	May 24 to May 30, 2021	4	0–0	0.00–0.00	0–2	0.00–33.33
Okinawa						
	Apr 5 to Apr 11, 2021	1	0–0	0.00–0.00	0–2	0.00–66.67
	Apr 12 to Apr 18, 2021	3	0–0	0.00–0.00	0–0	0.00–0.00
	Apr 19 to Apr 25, 2021	7	0–4	0.00–133.33	0–0	0.00–0.00
	Apr 26 to May 2, 2021	5	0–3	0.00–150.00	0–0	0.00–0.00
	May 3 to May 9, 2021	3	0–1	0.00–50.00	0–0	0.00–0.00
	May 10 to May 16, 2021	4	0–2	0.00–100.00	0–0	0.00–0.00
	May 17 to May 23, 2021	6	0–4	0.00–200.00	0–0	0.00–0.00
	May 24 to May 30, 2021	178	0–13	0.00–7.88	0–0	0.00–0.00
